# 2 deoxy-D-glucose augments the mitochondrial respiratory chain in heart

**DOI:** 10.1038/s41598-022-10168-1

**Published:** 2022-04-27

**Authors:** Irati Aiestaran-Zelaia, María Jesús Sánchez-Guisado, Marina Villar-Fernandez, Mikel Azkargorta, Lucia Fadon-Padilla, Uxoa Fernandez-Pelayo, Diego Perez-Rodriguez, Pedro Ramos-Cabrer, Antonella Spinazzola, Félix Elortza, Jésus Ruíz-Cabello, Ian J. Holt

**Affiliations:** 1 Biodonostia Health Research Institute, 20014 San Sebastián, Spain; 2 Center for Cooperative Research in Biomaterials (CIC biomaGUNE), Basque Research and Technology Alliance (BRTA), Paseo de Miramón 182, 20014 San Sebastián, Spain; 3 Centro de Investigación Biomédica en Red de Enfermedades Respiratorias (CIBERES), Madrid, Spain; 4 Proteomics Platform, CIC bioGUNE, Basque Research and Technology Alliance (BRTA), CIBERehd, ProteoRed-ISCIII, Bizkaia Science and Technology Park, 48160 Derio, Spain; 5 Department of Clinical and Movement Neurosciences, UCL Queen Square Institute of Neurology, Royal Free Campus, London, NW3 2PF UK; 6 IKERBASQUE, Basque Foundation for Science, 48013 Bilbao, Spain; 7 Departamento de Química en Ciencias Farmacéuticas, Facultad de Farmacia, Universidad Complutense de Madrid (UCM), Madrid, Spain; 8 CIBERNED (Center for Networked Biomedical Research On Neurodegenerative Diseases, Ministry of Economy and Competitiveness, Institute Carlos III), 28031 Madrid, Spain; 9 Universidad de País Vasco, Barrio Sarriena S/N, 48940 Leioa, Bilbao, Spain

**Keywords:** Cardiovascular biology, Proteomics

## Abstract

2-Deoxy-D-glucose (2DG) has recently received emergency approval for the treatment of COVID-19 in India, after a successful clinical trial. SARS-CoV-2 infection of cultured cells is accompanied by elevated glycolysis and decreased mitochondrial function, whereas 2DG represses glycolysis and stimulates respiration, and restricts viral replication. While 2DG has pleiotropic effects on cell metabolism in cultured cells it is not known which of these manifests in vivo. On the other hand, it is known that 2DG given continuously can have severe detrimental effects on the rodent heart. Here, we show that the principal effect of an extended, intermittent 2DG treatment on mice is to augment the mitochondrial respiratory chain proteome in the heart; importantly, this occurs without vacuolization, hypertrophy or fibrosis. The increase in the heart respiratory chain proteome suggests an increase in mitochondrial oxidative capacity, which could compensate for the energy deficit caused by the inhibition of glycolysis. Thus, 2DG in the murine heart appears to induce a metabolic configuration that is the opposite of SARS-CoV-2 infected cells, which could explain the compound’s ability to restrict the propagation of the virus to the benefit of patients with COVID-19 disease.

## Introduction

2 deoxy-D-glucose (2DG) reconfigures cell metabolism by restricting glucose consumption in cells and tissues owing to inhibition of glycolysis^1^; as such, there is considerable interest in its potential as an anti-cancer agent, given the highly glycolytic nature of many cancer cells^2^. Recently, 2DG has attracted further interest as it restricts SARS-CoV-2 viral replication in infected human cultured cells^3^, and it has recently received emergency approval for the treatment of COVID-19 in India, after a successful clinical trial^4^.

Following infection, viruses usurp control of the host cell and remodel processes and pathways to optimize viral production. Hence, configuring cell metabolism opposite to the preferences of the virus represents a potentially powerful anti-viral strategy. Previous studies have shown that glycolysis is upregulated in response to viral infection, and that 2DG restricts the replication of viruses as diverse as HSV, Rhinovirus, Norovirus and SARS-CoV-2^3,5,6^. However, inhibition of glycolysis is merely the most prominent of a number of pleiotropic effects of 2DG on cell metabolism. Deciphering 2DG’s effects in vivo is important to understand how it might restrict viral infections, such as SARS-CoV-2, and any complications that might segue, especially those affecting the heart where 2DG has been reported to have serious detrimental effects in rodents^7^.

Several intersecting cellular pathways and processes are linked to SARS-CoV-2 replication and propagation, including TORC1, HIF1α and one-carbon metabolism^8–10^, although the importance of each of these is still the subject of debate. Nevertheless, the chief rationale is that glycolysis is a major pathway to generate biosynthetic precursors that is hijacked by the viruses, and so 2DG would limit the availability of precursor molecules for viral construction. Glycolysis is also used to generate energy in the form of ATP and the energy yield depends on the fate of the final product, pyruvate: it is low when converted to lactate, but much higher when pyruvate is oxidized in the mitochondria, via the respiratory chain. Notably, the former does not require oxygen, whereas mitochondrial energy production is dependent on oxygen. Hence, the increase in glycolysis associated with SARS-CoV-2 infection^3^ might explain why many patients tolerate very low oxygen levels—presumably they are using glycolysis heavily and barely utilizing their mitochondria. In support of this hypothesis, perturbation of mitochondrial function is a feature of SARS-CoV-2 infected cells^8,11^. Thus, decreasing glycolysis and augmenting mitochondrial function could be beneficial to tackle the disease. As 2DG achieves both these effects in cultured cells^12^ we sought to determine whether this occurred in vivo in the heart, which could explain its reported anti-SARS.CoV-2 effects.

## Results

### Proteomic analysis accurately reflects mitochondrial respiratory chain abundance

A new generation of mass spectrometers has considerably enhanced the capacity of proteomic analysis to yield information on relative protein abundance. Because it samples thousands of proteins it is highly sensitive to changes in diverse aspects of cell and organ metabolism^13,14^. Of particular importance to our investigation was the mitochondrion, and so we compared heart and lung from C57BL/6J mice to determine whether the higher respiratory capacity of the heart^15^ was evident at the protein level. A mass spectrometry-based differential protein abundance analysis indicated that many proteins of the respiratory chain, TCA cycle and pyruvate dehydrogenase were detected at much higher frequency in heart than lung (Table S1), which establishes that this proteomic approach accurately reflects the abundance of mitochondrial energy producing machinery and allied enzymes and pathways.

### 2DG augments the mitochondrial respiratory chain in heart

To determine whether a high dose of 2DG could be well tolerated and induce changes in energy metabolism, we treated two groups of mice (B and C) intermittently for 8 months with 2DG and compared to a control group (A). Group B received 0.4% (w/v) 2DG in drinking water, 3 days per week, while group C received the same dose for 3 days every 2 weeks. Two days after the final dose mice were sacrificed and heart and lung proteins were analyzed by quantitative mass spectrometry. In the heart, among the 1227 identified proteins, 127 were significantly differentially expressed between controls and 2DG treated mice, almost all of which were more abundant in the 2DG treated animals. Irrespective of whether the two groups were considered individually (Tables 1, S2-S3) or together (Tables 2, S4), the most significantly altered pathways were related to the mitochondrial respiratory chain and ATP metabolism, based on Gene Ontology (GO) analysis. When the treated groups, were combined and compared to the controls, mitochondrial ATP synthesis coupled electron transport (GO:0042775) had a fold enrichment score of 28 and was the most significantly altered process (FDR 3.34E-06), among those with fewer than 100 members (Table 2). The larger category of ATP metabolic process (GO:0046034) was the most significantly altered process of all, FDR = 6.52E-12.

**Table 1:** Gene Ontology analysis of significantly altered biological processes in 2DG treated murine heart indicates that the principal effects of the compound are to augment the expression of proteins related to the respiratory chain and ATP synthesis.

GO biological process complete	Fold enrichment	Raw *P*-value	FDR < 0.05
Analysis type: B/A			
**Mitochondrial electron transport, NADH to ubiquinone (GO:0006120)**	76.35	4.54E-07	3.57E-03
**Purine ribonucleoside biosynthetic process (GO:0046129)**	51.53	3.96E-05	4.45E-02
**Nucleoside biosynthetic process (GO:0009163)**	51.53	3.96E-05	4.16E-02
**Ribonucleoside biosynthetic process (GO:0042455)**	51.53	3.96E-05	3.90E-02
**Purine nucleoside biosynthetic process (GO:0042451)**	51.53	3.96E-05	3.67E-02
Alternative mRNA splicing, via spliceosome (GO:0000380)	46.85	5.12E-05	4.48E-02
Glycosyl compound biosynthetic process (GO:1901659)	44.81	5.78E-05	4.79E-02
**Mitochondrial ATP synthesis coupled electron transport (GO:0042775)**	33.68	5.98E-07	3.14E-03
**ATP synthesis coupled electron transport (GO:0042773)**	31.23	8.48E-07	3.34E-03
**Oxidative phosphorylation (GO:0006119)**	23.53	3.15E-06	7.08E-03
**Respiratory electron transport chain (GO:0022904)**	23.53	3.15E-06	6.20E-03
**Electron transport chain (GO:0022900)**	22.31	4.03E-06	7.06E-03
**ATP metabolic process (GO:0046034)**	18.08	2.46E-09	3.88E-05
**Cellular respiration (GO:0045333)**	13.63	3.94E-05	4.78E-02
**Energy derivation by oxidation of organic compounds (GO:0015980)**	11.02	2.04E-05	2.68E-02

Analysis type: C/A			
Intracellular distribution of mitochondria (GO:0048312)	67.86	2.10E-05	2.07E-02
**Mitochondrial electron transport, NADH to ubiquinone (GO:0006120)**	45.24	6.04E-05	4.75E-02
**NADH dehydrogenase complex assembly (GO:0010257)**	22.16	4.30E-05	3.98E-02
**Mitochondrial respiratory chain complex I assembly (GO:0032981)**	22.16	4.30E-05	3.76E-02
Regulation of protein import (GO:1904589)	16.76	1.62E-05	1.70E-02
**Cellular respiration (GO:0045333)**	12.93	9.18E-06	1.31E-02
**Energy derivation by oxidation of organic compounds (GO:0015980)**	10.16	7.31E-06	1.28E-02

Upper section 2DG treated group B vs. controls (A), based on 64 significantly altered proteins. Lower section, 2DG treated group C vs. A (81 proteins). The peptide abundances were converted to log2 and a t-test applied and filtered, criteria for inclusion: FDR < 0.05; > tenfold enrichment. GO processes in bold relate to mitochondrial energy production and ATP metabolism.

**Table 2:** Gene Ontology analysis of significantly altered biological processes in 2DG treated murine heart indicates altered glycolysis/carbohydrate metabolism, as well as augmented respiratory chain capacity.

GO biological process complete	Fold Enrichment	Raw *P*-value	FDR < 0.001
Analysis type: (B + C) vs. A			
Cardiac myofibril assembly (GO:0055003)	49.66	1.52E-07	7.06E-05
**Mitochondrial ATP synthesis coupled electron transport (GO:0042775)**	28.04	1.27E-09	3.34E-06
**ATP synthesis coupled electron transport (GO:0042773)**	26.00	2.18E-09	3.82E-06
**Glycolytic process (GO:0006096)**	23.32	4.33E-07	1.75E-04
**ATP generation from ADP (GO:0006757)**	22.82	4.86E-07	1.87E-04
**ADP metabolic process (GO:0046031)**	22.75	5.30E-08	3.79E-05
**NADH dehydrogenase complex assembly (GO:0010257)**	21.89	6.08E-07	2.18E-04
**Mitochondrial respiratory chain complex I assembly (GO:0032981)**	21.89	6.08E-07	2.13E-04
**Purine ribonucleoside diphosphate metabolic process (GO:0009179)**	21.21	8.23E-08	4.99E-05
**Purine nucleoside diphosphate metabolic process (GO:0009135)**	21.21	8.23E-08	4.80E-05
**Nucleoside diphosphate phosphorylation (GO:0006165)**	20.86	9.15E-08	4.97E-05
**Nucleotide phosphorylation (GO:0046939)**	19.86	1.24E-07	6.12E-05
**Oxidative phosphorylation (GO:0006119)**	19.59	1.67E-08	1.55E-05
**Respiratory electron transport chain (GO:0022904)**	19.59	1.67E-08	1.46E-05
**Ribonucleoside diphosphate metabolic process (GO:0009185)**	19.55	1.37E-07	6.56E-05
**Electron transport chain (GO:0022900)**	18.57	2.45E-08	2.03E-05
**Pyruvate metabolic process (GO:0006090)**	17.88	2.42E-07	1.09E-04
**ATP metabolic process (GO:0046034)**	17.77	4.14E-16	6.52E-12
**Carbohydrate catabolic process (GO:0016052)**	16.82	5.00E-08	3.75E-05
**Nucleoside diphosphate metabolic process (GO:0009132)**	15.45	6.07E-07	2.22E-04
**Cellular respiration (GO:0045333)**	12.77	6.59E-08	4.32E-05
**Energy derivation by oxidation of organic compounds (GO:0015980)**	12.43	9.43E-11	4.95E-07
**Generation of precursor metabolites and energy (GO:0006091)**	11.92	6.46E-15	5.09E-11

GO biological processes identified as significantly altered in murine heart by 2DG treatment combining the data from two groups treated with 2DG (B and C), vs. controls (A) (127 proteins). The peptide abundances were converted to log2 and a t-test applied and filtered, criteria for inclusion: FDR < 0.001; > tenfold enrichment. GO processes in bold relate to mitochondrial energy production and ATP metabolism, or glycolysis/carbohydrate metabolism.

**Figure 1:**
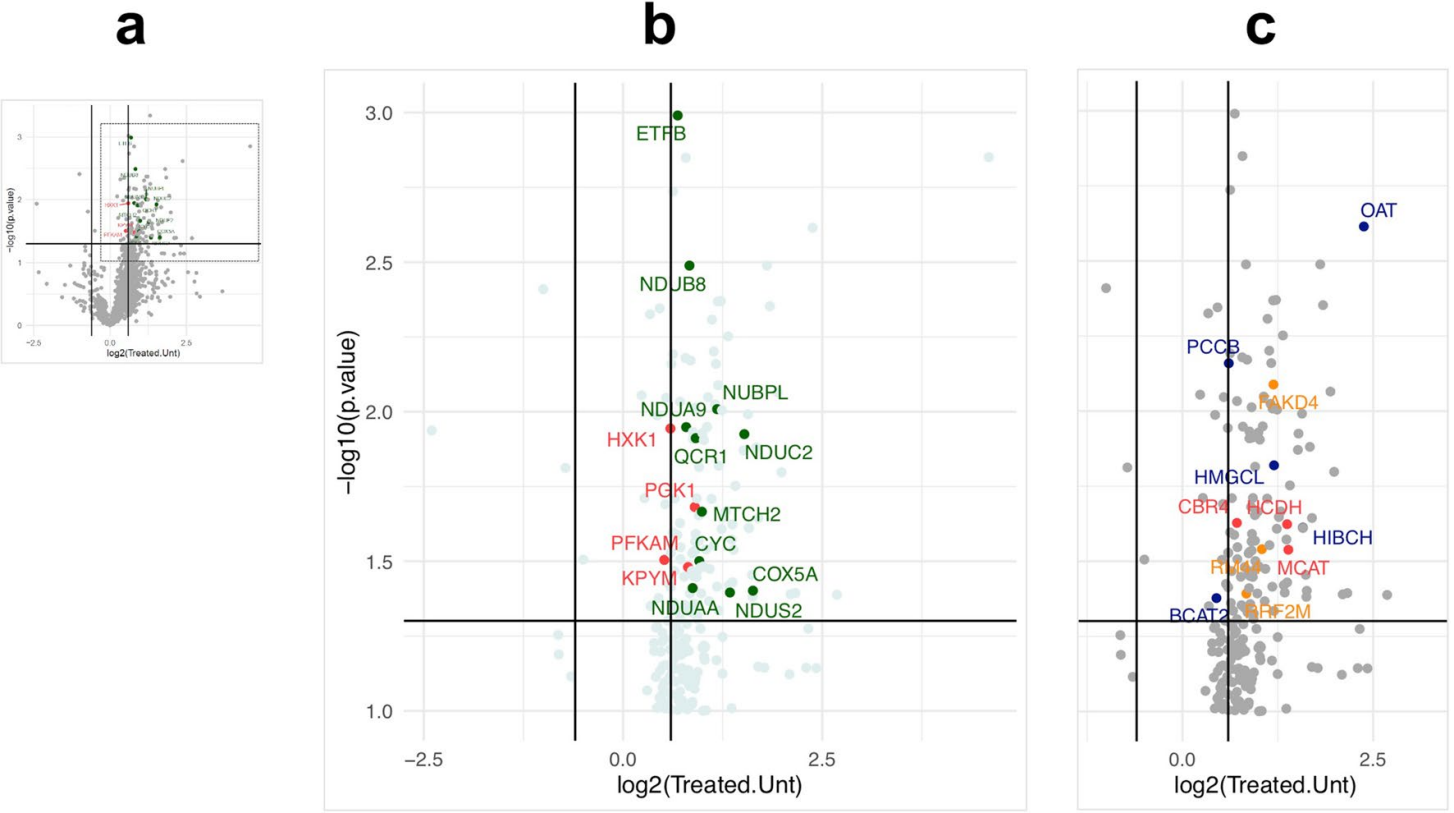
Volcano plots of quantitative proteomic analysis of hearts of control and 2DG treated mice. (**a**) In miniature, fold-change and significance of all 1237 proteins identified in hearts of 2DG-treated mice versus controls. (**b**) Magnified region showing the significantly altered individual proteins related to the respiratory chain (green) and glycolysis (HXK1, PFKAM, PGK1, TPIS and KPYM) (red) (see Table S4 for the complete list and individual scores). (**c**) Magnified region of the volcano plot showing the significantly altered proteins related to mitochondrial branched-chain amino acid catabolism BCAT2, HMGCL, HIBCH, and PCCB, in blue, and OAT (which is involved in amino acid inter-conversion, as well as synthesis); 3 proteins in orange related to mtDNA expression (RRF2M, RM44, FAKD4), whereas three proteins of fatty acid metabolism, HCDH, MCAT and CBR4, are marked in red. Other components of the mitochondrial proteome significantly altered by the 2DG treatment (not highlighted) were two proteins related to mitochondrial morphology and division MTFP1, ARMC1, the mitochondrial transporter protein ABCB10; TOM70 a mitochondrial protein import factor; CLYBL (putative malate synthase); CDS2 (cardiolipin synthesis); GSHR and TRXR2 (redox homeostasis) and OXNAD1 (unknown function).

Volcano plot analysis of the individual proteins also indicated a pronounced skew to the respiratory chain (10 structural components, 1 assembly factor and MTCH2 that is annotated as ‘respiratory chain’ in Gene Ontology) (Fig. 1a). Of the 19 other significant mitochondrial proteins four are linked to branch-chain amino acid (BCAA) metabolism: BCAT2 (ILV catabolism), HMGCL (leucine catabolism), HIBCH (Valine catabolism) and PCCB (Catabolism of BCAAs and other metabolites). Because glucose represses the expression of factors involved in BCAA degradation in cultured cardiomyocytes^16^, increased levels of BCAA catabolic enzymes imply restricted glucose metabolism.

Three other mitochondrial factors increased by 2DG treatment are related to the expression of mtDNA (RRF2M, RM44, FAKD4), and two to mitochondrial distribution (MTFP1, ARMC1) (Fig. 1b). The other significantly altered mitochondrial proteins (Fig. 1, legend) do not fit a particular pathway or process in the organelle, nor do they suggest a wholesale increase in mitochondrial biogenesis, which would have given rise to higher levels of a much broader range of protein components of the mitochondria. Instead, the proteomic analysis indicates that 2DG primarily increases the abundance of mitochondrial respiratory chain factors, and enhances branched-chain amino acid catabolism (Tables 1, 2 and S4, and Fig. 1); both of which are concordant with decreased glucose metabolism. In contrast to the heart, there were no significantly altered pathways of any description, between 2DG treated and control lung, based on Gene Ontology analysis (Table S5).

### Altered glycolysis in 2DG-treated heart

The second most highly ranked process in the combined comparison (Table 2) was the ‘generation of precursor metabolites and energy’ (GO:0006091) (FDR = 5.63E-11). This is notable because it encompasses glycolysis, which 2DG inhibits in cultured cells and *in vivo*^17^. Direct evidence that 2DG has a major impact on glycolysis in the heart comes from the significant changes in five glycolytic enzymes induced by the 2DG treatments, including Hexokinase I that is a direct target of 2DG (Fig. 1b, Table S4). GO analysis indicated a 23-fold enrichment of proteins related to the glycolytic process (GO:0006096) with a highly significant FDR score of 1.75E-04.

In contrast to the changes in mitochondrial respiration and glycolysis, there was no striking evidence of ER-stress (aka the unfolded protein response (UPR)), which is another established effect of 2DG in cultured cells^18^. No GO process related to UPR achieved an FDR < 0.05, even when considering those with enrichments scores below the cut-off of 10 applied to the data in Tables 1 and 2, and while there was a 1.4 fold increase in the canonical UPR marker BiP/GRP78 in the hearts of the 2DG treated mice, it was not significant (*p* = 0.213). UPR induction may be limited by enhanced HSP70 and HSP90 coupling, as the protein that mediates this connection, STIP1, was significantly increased in the 2DG treated mice (Table S4). More recently 2DG treatment has been shown to restrict glutamine, as well as glucose, utilization in cultured cells^19^. Although there was no significant alteration to any GO process related to glutamine metabolism (Table 2), glutamine-tRNA ligase and cytoplasmic aspartate-tRNA ligase were among the significantly increased proteins, as was a component of the glutamate-aspartate shuttle, GOT1 (cytoplasmic aspartate aminotransferase) (Table S4), which might reflect changes in glutamine and aspartate metabolism. Notwithstanding these considerations, the prominent changes in the respiratory chain coupled with those of glycolysis and the allied pathways of carbohydrate metabolism strongly indicate that the major effect of 2DG in the heart is altered glucose metabolism, and that this is over and above any other effects, at least from a proteomic perspective.

### Intermittent 2DG treatment does not cause overt cardiac toxicity

**Figure 2:**
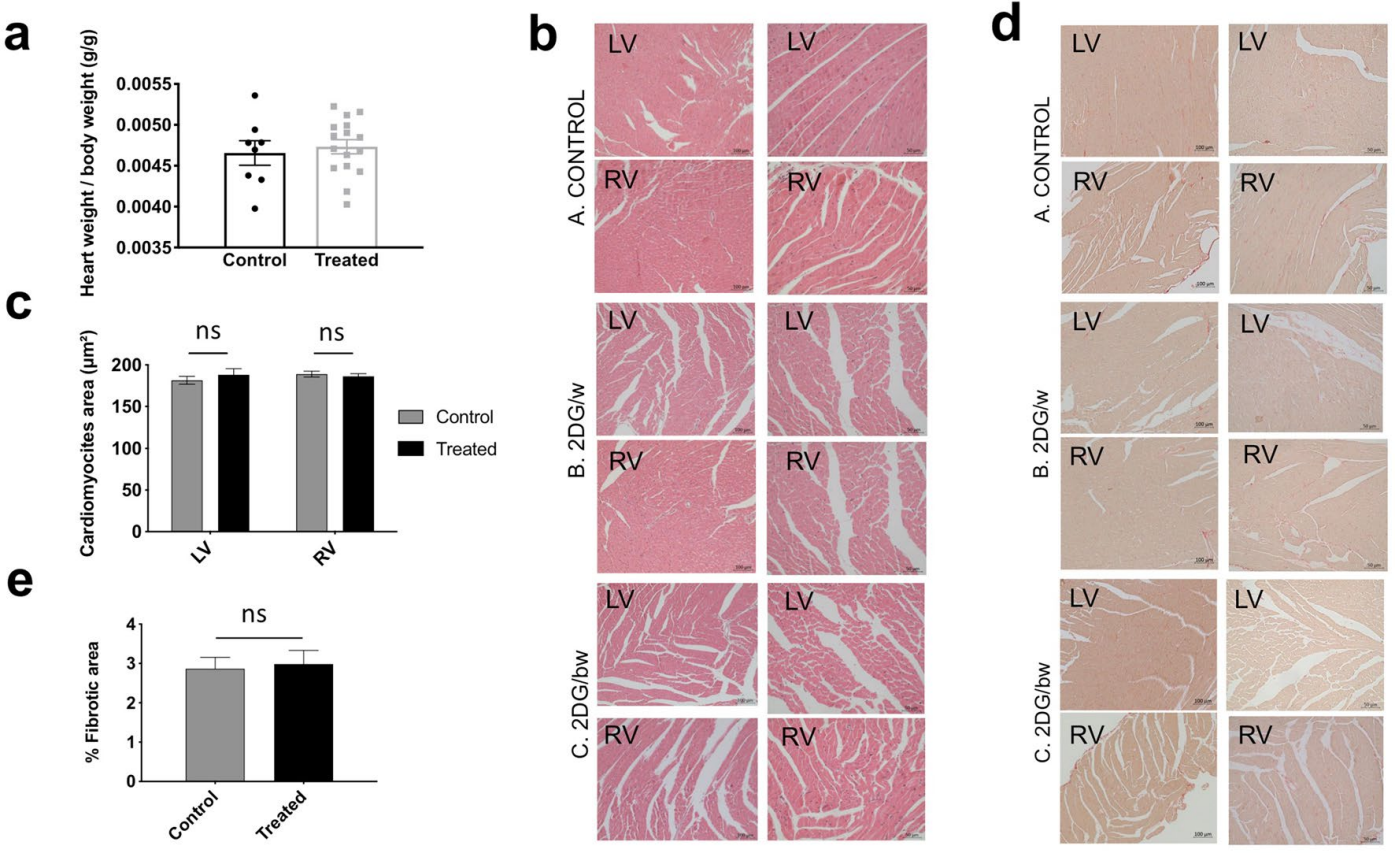
Histological staining indicates normal heart structure in controls and 2DG treated animals with no evidence of fibrosis. (**a)** Heart body weight ratio in 8 month old mice treated without (controls) or with 2DG (treated) for 8 months (see methods for details); *n* = 8 controls; *n* = 16 treated mice. **(b)** Heart sections of 4 µm stained with hematoxylin eosin; LV and RV, left and right ventricle respectively. Objective lens 20 × for the left column, 40 × for the right; NA, 0.30. (**c)** Quantitative analysis of the longitudinal area of ~ 100 cardiomyocytes from 3 mice per group revealed no significant difference (ns) in fibre size between treated and control animals in either the left (LV) or right (RV) ventricle. (**d)** As (**b**) except that the heart sections were stained with picrosirius red to highlight fibrotic fibers. (**e)** Quantitative analysis of the fibrotic area of the heart from sections such as those shown in panel (**d)** showing no significant difference between control and 2DG treated mice.

**Figure 3:**
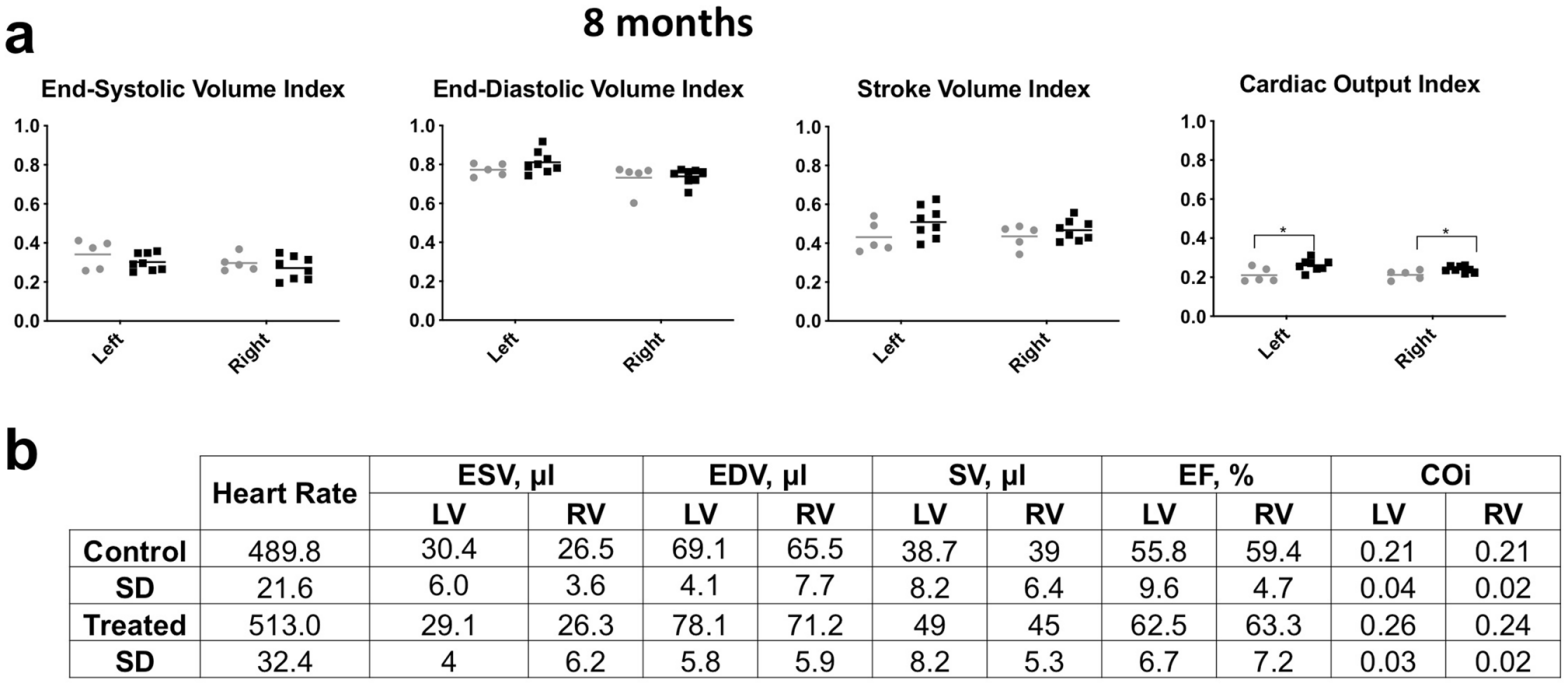
Heart function is similar in control and 2-DG treated mice. **(a)** Heart capacity was measured by MRI. Right and left ventricle End-Systolic and End-Diastolic Volume index, Stroke Volume index and Cardiac Output index, adjusted for body surface area (BSA = 20 × body weight (g) ^ 0.42). There was no significant difference in the Ejection Fraction (EF) (amount, or percentage, of blood that is ejected from the ventricles with each contraction (EF = (SV/EDV) × 100)), or the volume remaining after contraction (End-Systolic Volume (ESV) or systole). There was a small increase in the cardiac output index in the 2DG treated animals (*n* = 8) versus controls (*n* = 6) which was significant, *p* = 0.03 for the right ventricle and *p* = 0.02 for the left. (**b)** Selected parameters measured to assess cardiac function. Heart rate (beats per minute), End-Systolic volume (ESV), End-Diastolic volume (EDV): volume of blood in a ventricle at the end of diastole, just before systole starts, Stroke volume (SV): volume of blood pumped from the left ventricle per beat (SV = EDV-ESV), Ejection Fraction (EF) (amount, or percentage, of blood that is ejected from the ventricles with each contraction (EF = (SV/EDV) × 100)), and Cardiac Output index (COi). The last was derived from Cardiac Output (CO): amount of blood pumped by the heart per minute (CO = (Heart rate x SV)/1000) and the Cardiac Index (CI), which relates the cardiac output (CO) from left ventricle in one minute to body surface area (CI = (CO/BSA) × 10,000)). There were no significant differences in the EF, ESV, CO or CI.

The 0.4% (w/v) 2DG in drinking water equated to 320 mg/Kg/day, which, at both 3 days per week and 3 days every two weeks, increased the mitochondrial respiratory chain proteome (Table 2). Hence, these molecular changes do not require a continuous 2DG treatment and doses can be spaced at least 11 days apart. The intermittent dosing regimes were designed primarily to avoid the severe cardiac abnormalities and premature death associated with a chronic, continuous 2DG treatment of rodents at the same dose^7^. After 8 months of intermittent 2DG treatment, the mice had reached approximately 1 year old, and there were no deaths among any of the three groups. Moreover, the heart to body mass ratio, and individual myofibre size were essentially the same for controls and 2DG treated mice with no evidence of fibrosis or vacuolization based on hematoxylin eosin and picrosirius red staining (Fig. 2). The only difference in heart performance, revealed by magnetic resonance imaging, was a small increase in the cardiac output index after eight months of treatment (Fig. 3). At the proteomic level, cardiac myofibril assembly factors (GO:0055003) were 50-fold enriched (FDR = 7.06E-05), suggesting that myofibril assembly is augmented by the 2DG treatments. A likely contributing factor to this change is the most significantly altered protein mimecan/osteoglycin (Table S4), as it is implicated in the regulation of left ventricular mass^20^.

## Discussion

2DG restricts glucose consumption by inhibiting glycolysis, and in cultured cells curtailed glycolytic ATP production can be compensated by increased respiration (e.g.^12^). The elevated mitochondrial respiratory chain proteome (Tables 1 and 2, and Fig. 1) strongly suggests 2DG induces the same changes in the heart. That 2DG is effective in vivo as well as in vitro boosts its prospects for clinical development. The therapeutic potential of 2DG extends from viruses^3–6^ to cancer^2^, and it holds considerable promise for autosomal dominant polycystic disease (ADPKD) and some mitochondrial DNA diseases^12,17^. While these disorders have different features, the expectation is that all will benefit from a switch from glycolytic to mitochondrial ATP production. As the treatments are likely to endure months or years, an important facet of the new data is that they suggest extended, intermittent 2DG treatment regimes can be devised that alter energy metabolism without causing cardiac toxicity or premature death. The new findings complement those in the rat, where continuous exposure to 0.25% (w/v) 2DG (approximately 200 mg/Kg/day) was not cardiotoxic^7^, and only beneficial effects were detected from a 10 week treatment of ADPKD mice with 100 mg/Kg/day 2DG, 5 days per week^17^. Hence, intermittent treatments at doses lower than the one used in this study, such as 100 mg/Kg/day 2DG, could augment mitochondrial function with minimal or no detrimental effect on the heart.

**Figure 4:**
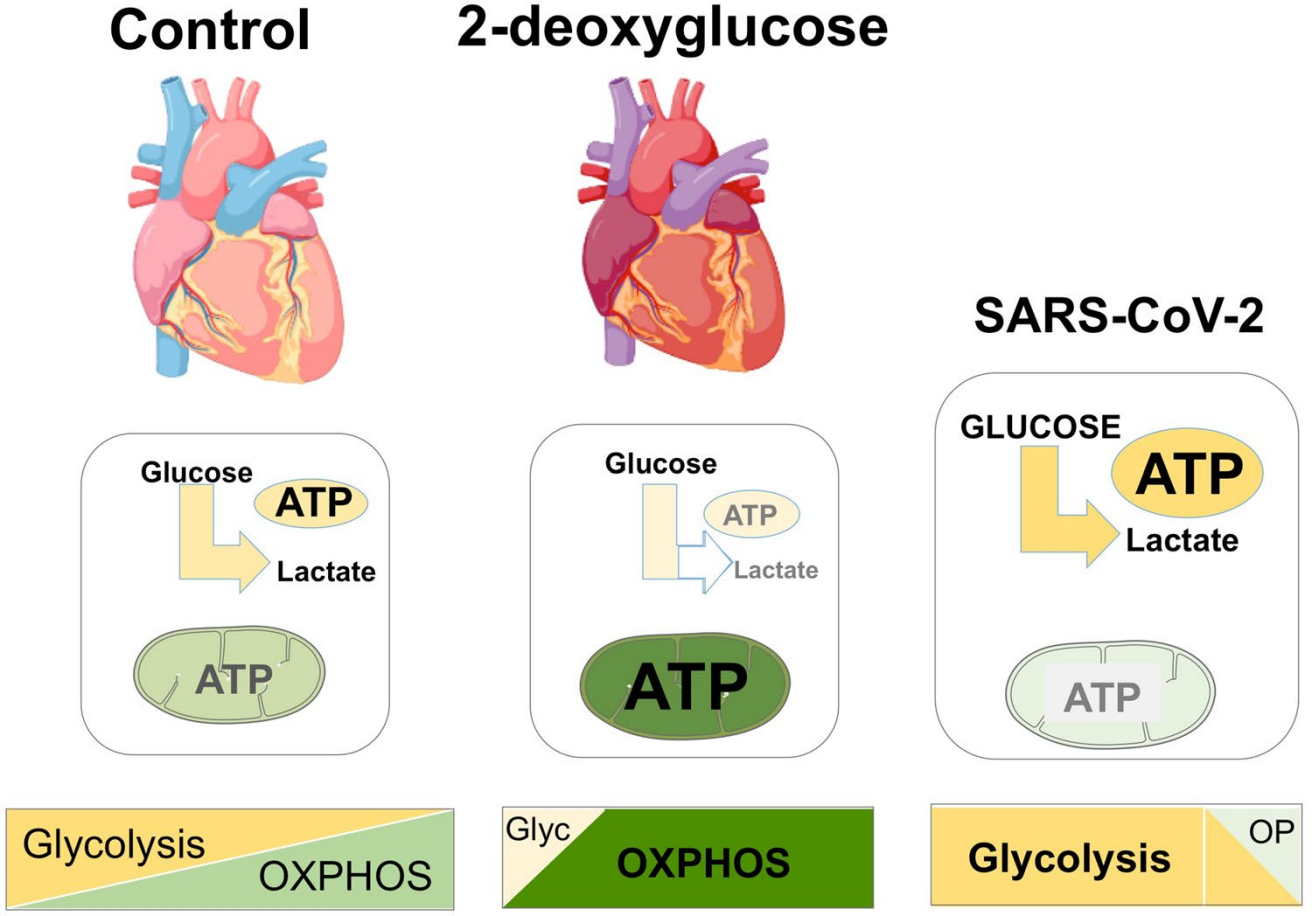
The effect of 2DG on the balance between glycolytic and mitochondrial energy production contrasts with that of SARS-CoV2. The proteomic signature found in this study suggests that the principle effect of 2-deoxyglucose (2DG) in the heart is to shift the equilibrium from glycolytic to mitochondrial (OXPHOS/OP) ATP production, as occurs in cultured cells. These effects are opposite to those of SARS-CoV2 infection in cells (illustrated to the right), and so support the idea that 2-deoxyglucose could have anti-viral properties in vivo as well as in cultured cells^3^.

In the particular case of SARS-CoV-2, the current study suggests 2DG could have the same anti-viral properties in major organs, such as the heart, as those reported in cultured cells^3^. In vitro the virus increases glycolysis and represses mitochondrial function^3,8,11^ and 2DG is diametrically opposed to these changes, in that the increase in the respiratory chain predicts a switch in energy producing pathways, from glycolytic to mitochondrial energy production (Fig. 4). Thus, while we do not yet know whether the heart responds the same to 2DG when an individual is infected with SARS-CoV-2, the new findings offer a credible explanation for the reported positive outcomes of 2DG treatment of patients in India with COVID19.

## Methods

### Ethics statement

This study (PRO-AE-SS-185) was approved by the Ethical Committee of CIC biomaGUNE and local authorities of Gizpukoa. Animal maintenance and handling was conducted in accordance with the European Council Directive 2010/63/UE.

### Animal maintenance

C57BL/6 J mice of three months of age were purchased from Charles River laboratories and were fed a standard chow, and normal unadulterated drinking water (group A), or drinking water with 0.4% (w/v) 2DG three days per week (group B), or three days every two weeks (group C).

### Mass spectrometry analysis

Hearts were homogenized in RIPA buffer with an ultra-turrax, 2 bursts of 10 s on ice. Homogenized heart was solubilized with 7 M Urea, 2 M Tiourea, and 4% CHAPS and in-solution protein digestion was performed following the FASP protocol^21^, using Amicon Ultra 30 K devices (Merck-Millipore). Proteins were quantified with Bradford assays (Bio-Rad), prior to overnight incubation with trypsin at 37ºC (at the relatively high trypsin: protein ratio of 1:10 to maximize cleavages). The resulting peptides were dried and resuspended in 0.1% FA, and sonicated for 5 min prior to analysis in an LC MS/MS system: EVOSEP ONE (EVOSEP) coupled on-line to a hybrid trapped ion mobility spectrometry – quadrupole time of flight mass spectrometer (timsTOF Pro with PASEF, Bruker Daltonics)^13^. Raw MS files were analysed using PEAKS Pro (Bioinformatics Solutions Inc.). Proteins were identified matching to a mouse (Uniprot/Swissprot Mus musculus) with a maximum of 2 missed cleavages and with precursor and fragment tolerances of 20 ppm and 0.05 Da. A false discovery rate estimation procedure was applied for peptide identification (FDR < 1%) and only proteins detected with at least 2 peptides were considered for the quantitative analysis. Duplicates were acquired for each sample, and protein abundances were averaged before analysis. Data was analysed using the Perseus platform^22^, protein abundances were log_2_ transformed and imputed, then compared using a student’s t-test (*p* < 0.05 considered as significant).

### Magnetic resonance imaging

Anesthesia was induced with 3% isoflurane in 30% oxygen and maintained at 1–2% isoflurane during the experiments. Heart analysis was performed on a 7 Tesla Bruker Biospec 70/30 USR MRI system (Bruker Biospin GmbH, Ettlingen, Germany), interfaced to an AVANCE III console and with a BGA12-S imaging gradient insert (maximal gradient strength 400 mT/m, switchable within 80 µs). Measurements were performed with a 72 mm volumetric quadrature coil for excitation and a 20 mm rat brain surface coil for reception. Short axis images were acquired from the apex covering both ventricles. 11–12 0.8 mm slices were acquired using a Bruker cine IntraGate FLASH sequence using the following parameters: Effective TE = 2.82 ms; TR = 6.87 ms; Matrix = 256 × 128 points; FA = 30; FOV = 25 × 25 mm. Images were reconstructed in 15 phases of a single cardiac cycle.

### Histology

Paraffin-embedded section (4 µm) of the heart were mounted on glass slides. Subsequently, slides of the heart were stained with hematoxylin eosin or Picrosirius red to assess the structure and fibrosis by imaging software (ImageJ, 1.52P). Image acquisition was performed with an inverted epifluorescence microscope (Axio Observer Z1, Zeiss).

### Statistical analysis

Analyses were performed using GraphPad Prism version 8, San Diego, California USA. Data are expressed as mean ± s.e.m., unless otherwise indicated. Statistical comparisons were made in Prism software using a Student’s t-test (for two groups meeting the normal distribution criteria, according to the Shapiro–Wilk normality test), Mann–Whitney U-test (for two groups not meeting the normal distribution criteria) or ANOVA with Tukey’s multiple comparison test (for groups across variables, with multiple comparisons between groups). For all tests, *P* < 0.05 was considered significant.

## Ethics declarations

The study is reported in accordance with ARRIVE guidelines (https://arriveguidelines.org).

## Supplementary Information

Supplementary Information.

## Data Availability

In addition to the data displayed in the figures of the main article, further details of the individual proteins significantly differently expressed in hearts treated with 2DG vs. controls are listed in Supplementary Tables 1–3. The full set of proteomic data have been deposited in PRIDE (PXD027974).
