# Supplementary material for: 2 deoxy-D-glucose augments the mitochondrial respiratory chain in heart
Source: Sci Rep. 2022 Apr 27;12:6890. doi: 10.1038/s41598-022-10168-1 (PMC9044724; doi:10.1038/s41598-022-10168-1)
Supplement: Supplementary file 1 — Supplementary Information. [file 41598_2022_10168_MOESM1_ESM.pdf]

Supplementary Table Legends.

Table S1. Mitochondrial proteins more highly expressed in heart than lung or vice-versa. ND - not detected, VL - very low expression <0.01, L - low expression, <0.1, see Table S5 for specific values.

Table S2. List of quantified proteins significantly different between group B vs. A, from which GO analysis in the upper part of Table 1 is derived.

Table S3. List of quantified proteins significantly different between group C vs. A, from which GO analysis in the lower part of Table 1 is derived.

Table S4. List of quantified proteins significantly different between hearts of mice treated with (B and C) or without (A) 2DG intermittently for 8 months. Data used for GO analysis of biological processes and volcano plots, Table 2 and Figure 1, respectively.

Table S5. Relative abundance of proteins in murine heart and lung. Total protein from heart or lung homogenates was analysed by mass spectrometry. The values are relative abundance; A - controls, B and C - mice treated with 2DG. The data in Table S1 are derived from this complete list.

Function	Protein	Heart > Lung	Lung > Heart	Protein ID	Function	Protein	Heart > Lung	Lung > Heart	Protein ID
OXPHOS									
Complex I	NDUAC	28 fold		Q7TMF3	ADP/ATP exchange	ADT1	4 fold		P48962
	NDUS1	15 fold		Q91VD9					
	NDUAA	14 fold		Q99LC3	TCA cycle	IDHP	5 fold		P54071
	NDUV1	15 fold		Q91YT0		FUMH	7 fold		P97807
	NDUB9	16 fold		Q9CQJ8		ACON	25 fold		Q99KI0
	NDUA6	5 fold		Q9CQZ5		CISY	5 fold		Q9CZU6
	NDUB8	10 fold		Q9D6J5		ODO1	16 fold		Q60597
	NDUV2	15 fold		Q9D6J6		ODO2		12 fold	Q9D2G2
	NDUA9	40 fold		Q9DC69		SUCA	11 fold		Q9WUM5
	NDUBA	8 fold		Q9DCS9		SUCB1	10 fold		Q9Z2I9
	NDUS3	16 fold		Q9DCT2					
	NDUAD	15 fold		Q9ERS2	PDH	ODP2	100 fold		Q8BMF4
	NDUB6	ND	L	Q3UIU2		ODPB	5 fold		Q9D051
	NDUA2	ND	VL	Q9CQ75		PDK2	6 fold		Q9JK42
	NDUA3	ND	VL	Q9CQ91		PDP1	4 fold		Q3UV70
	NDUB4	ND	L	Q9CQC7					
	NDUB5	ND	L	Q9CQH3	Beta-oxidation	ACADV	10 fold		P50544
	NDUB3	ND	VL	Q9CQZ6		ACADL	10 fold		P51174
	NDUB7	5 fold		Q9CR61		ECHA	30 fold		Q8BMS1
						ECHB	9 fold		Q99JY0
Complex II	SDHA	25 fold		Q8K2B3		THIL	10 fold		Q8QZT1
	SDHB	3 fold		Q9CQA3					
Complex III	QCR1	9 fold		Q9CZ13	Morphology	OPA1	7.5 fold		P58281
	QCR2	20 fold		Q9DB77					
	UQCC1	ND	VL	Q9CWU6	RNA metabolism	LRPPRC	8 fold		Q6PB66
Complex IV	COX2	4 fold		P00405		VWA8	7.5 fold		Q8CC88
	CX6B1	24 fold		P56391	Carrier	CMC1	10 fold		Q8BH59
	COX20	L	ND	Q9D7J4					
Cytochrome c	CY1	40 fold		Q9D0M3	Protease	AFG32		7 fold	Q8JZQ2
ATP synthase	ATP5B	20 fold		P56480	Fatty Acetyl CoA	ACOT2		5 fold	Q9QYR9
	ATP5J	13 fold		P97450					
	ATP5A	12 fold		Q03265					
	ATP5I (e)	ND	L	Q06185					
	ATPMD (k)	ND	L	Q78IK2					
	ATPO	6 fold		Q9DB20					
	ATP5H	30 fold		Q9DCX2					

Table S1. Mitochondrial proteins more highly expressed in heart than lung or vice-versa.

Table S2. List of quantified proteins significantly different between group B vs. A from which GO analysis in the upper part of Table 1 is derived.

T: Accession	T: Entry	T: Description B/A	T: #Peptides	T: #Unique	Student's T-test Significant A_B	Student's T-test significant	N: -Log Student's T-test p-value A_B	N: Student's T-test Difference A_B	p value	B/A	N: Student's T-test Test statistic A_B	A4	A6	B21	B20	C34	C35
Q61081	CDC37_MOUSE	Hsp90 co-chaperone Cdc37 OS=Mus musculus OX=10090 GN=Cdc37 PE=1 SV=1	2	2	+	A_B	3.10671	0.672746	0.00078215	0.627311537	35.7357	10.6707	10.7056	10.0224	10.0084	10.6348	11.4147
Q9ES46	PARVB_MOUSE	Beta-parvin OS=Mus musculus OX=10090 GN=Parvb PE=1 SV=1	3	3	+	A_B	3.08095	-0.756808	0.000829946	1.689747875	-34.69	12.2015	12.2198	12.9476	12.9873	12.4409	13.4767
Q791V5	MTCH2_MOUSE	Mitochondrial carrier homolog 2 OS=Mus musculus OX=10090 GN=Mtch2 PE=1 SV=1	5	5	+	A_B	2.69117	-0.829558	0.002036245	1.777140814	-22.1269	13.0189	13.0668	13.8435	13.9012	13.8138	14.5687
Q62000	MIME_MOUSE	Mimecan OS=Mus musculus OX=10090 GN=Ogn PE=1 SV=1	2	2	+	A_B	2.50541	-4.01523	0.00312313	16.16980079	-17.852	5.35403	4.97728	9.05799	9.30378	9.88722	10.7731
Q99JY3	GIMA4_MOUSE	GTPase IMAP family member 4 OS=Mus musculus OX=10090 GN=Gimap4 PE=1 SV=2	2	2	+	A_B	2.49148	-1.34826	0.003224928	2.546048674	-17.5665	11.0084	11.1611	12.4409	12.4252	13.1098	11.8178
Q9ES97	RTN3_MOUSE	Reticulon-3 OS=Mus musculus OX=10090 GN=Rtn3 PE=1 SV=2	2	2	+	A_B	2.4667	3.73875	0.003414287	0.074907293	17.07	11.5507	11.3049	7.87036	7.50779	11.1163	9.88264
Q99L43	CDS2_MOUSE	Phosphatidate cytidylyltransferase 2 OS=Mus musculus OX=10090 GN=Cds2 PE=1 SV=1	2	2	+	A_B	2.46579	-1.10738	0.003421448	2.154540175	-17.0522	10.388	10.388	11.5603	11.4305	10.944	11.6027
Q9EP89	LACTB_MOUSE	Serine beta-lactamase-like protein LACTB mitochondrial OS=Mus musculus OX=10090 GN=Lactb PE=1 SV=1	2	2	+	A_B	2.22779	-0.449347	0.005918477	1.365422093	-12.9408	9.5216	9.50184	9.99435	9.92778	9.87805	8.98584
P55264	ADK_MOUSE	Adenosine kinase OS=Mus musculus OX=10090 GN=Adk PE=1 SV=2	3	3	+	A_B	2.12615	-1.18322	0.007479111	2.270830462	-11.4982	11.7398	11.8803	12.9181	13.0684	12.7855	11.3332
Q9D6J5	NDUB8_MOUSE	NADH dehydrogenase [ubiquinone] 1 beta subcomplex subunit 8 mitochondrial OS=Mus musculus OX=10090 GN=Ndufb8 PE=1 SV=1	5	5	+	A_B	2.10517	-0.93843	0.007849283	1.916441553	-11.2207	14.8337	14.8386	15.691	15.8582	15.4318	15.6964
Q8K3J1	NDUS8_MOUSE	NADH dehydrogenase [ubiquinone] iron-sulfur protein 8 mitochondrial OS=Mus musculus OX=10090 GN=Ndufs8 PE=1 SV=1	2	2	+	A_B	2.02313	1.63995	0.009481346	0.320867595	10.1967	13.4893	13.7207	12.0768	11.8533	12.7936	13.8823
P26043	RADI_MOUSE	Radixin OS=Mus musculus OX=10090 GN=Rdx PE=1 SV=3	4	3	+	A_B	2.02094	-1.01052	0.009529278	2.014637118	-10.1706	11.3498	11.1548	12.2819	12.2438	11.744	12.2527
P53994	RAB2A_MOUSE	Ras-related protein Rab-2A OS=Mus musculus OX=10090 GN=Rab2a PE=1 SV=1	4	4	+	A_B	1.92248	-0.341427	0.011954186	1.2670092	-9.064	11.9256	11.8727	12.2137	12.2674	12.3134	12.7524
Q8K310	MATR3_MOUSE	Matrin-3 OS=Mus musculus OX=10090 GN=Matr3 PE=1 SV=1	3	3	+	A_B	1.91693	-0.901247	0.012107933	1.867679623	-9.0052	9.98014	9.89482	10.7482	10.9293	10.7313	11.3987
Q8R4N0	CLYBL_MOUSE	Citramalyl-CoA lyase mitochondrial OS=Mus musculus OX=10090 GN=Clybl PE=1 SV=2	4	4	+	A_B	1.87364	-0.978233	0.013377039	1.97005103	-8.55909	11.5265	11.5216	12.6165	12.388	12.7711	11.9658
A2AN08	UBR4_MOUSE	E3 ubiquitin-protein ligase UBR4 OS=Mus musculus OX=10090 GN=Ubr4 PE=1 SV=1	4	4	+	A_B	1.87356	0.978334	0.013379504	0.50756553	8.5583	11.0224	11.0154	10.1548	9.9263	9.74987	10.2408
P47791	GSHR_MOUSE	Glutathione reductase mitochondrial OS=Mus musculus OX=10090 GN=Gsr PE=1 SV=3	2	2	+	A_B	1.85936	-0.644831	0.0138242	1.563556118	-8.4167	9.40514	9.36413	9.95565	10.1033	10.1674	9.81218
P18242	CATD_MOUSE	Cathepsin D OS=Mus musculus OX=10090 GN=Ctsd PE=1 SV=1	3	3	+	A_B	1.80448	-0.649932	0.015686281	1.569094236	-7.89014	12.7628	12.5981	13.3304	13.3304	14.1115	13.4893
Q91VH6	MEMO1_MOUSI	Protein MEMO1 OS=Mus musculus OX=10090 GN=Memo1 PE=1 SV=1	2	2	+	A_B	1.79356	-0.597191	0.016085701	1.512768261	-7.78916	8.52748	8.66178	9.22882	9.15482	9.35755	9.13955
P09055	ITB1_MOUSE	Integrin beta-1 OS=Mus musculus OX=10090 GN=Itgb1 PE=1 SV=1	3	3	+	A_B	1.75813	-0.606489	0.017452996	1.52254936	-7.46997	7.45121	7.53916	8.03342	8.16992	8.2384	7.2854
Q9DCT1	AKCL2_MOUSE	1 5-anhydro-D-fructose reductase OS=Mus musculus OX=10090 GN=Akr1e2 PE=1 SV=1	2	2	+	A_B	1.73776	-0.846251	0.018291107	1.797823011	-7.29214	9.7211	9.87805	10.7313	10.5603	9.83131	10.6439
Q91V61	SFXN3_MOUSE	Sideroflexin-3 OS=Mus musculus OX=10090 GN=Sfxn3 PE=1 SV=1	3	3	+	A_B	1.73335	1.28717	0.018477789	0.409754018	7.25423	9.47167	9.82655	8.36194	8.36194	9.16992	9.36194
Q9R0Y5	KAD1_MOUSE	Adenylate kinase isoenzyme 1 OS=Mus musculus OX=10090 GN=Ak1 PE=1 SV=1	5	5	+	A_B	1.72903	-1.42676	0.018662508	2.688422718	-7.21717	15.1908	14.887	16.3391	16.5922	15.4383	16.5893
Q3TZZ7	ESYT2_MOUSE	Extended synaptotagmin-2 OS=Mus musculus OX=10090 GN=Esyt2 PE=1 SV=1	2	2	+	A_B	1.70486	-0.907253	0.019730587	1.875471058	-7.01337	7.7211	7.46761	8.47573	8.52748	8.92184	9.00562
P63017	HSP7C_MOUSE	Heat shock cognate 71 kDa protein OS=Mus musculus OX=10090 GN=Hspa8 PE=1 SV=1	11	7	+	A_B	1.68549	-0.389038	0.020630512	1.309519915	-6.85397	15.4318	15.3233	15.7498	15.7834	15.6068	16.68
P98192	GNPAT_MOUSE	Dihydroxyacetone phosphate acyltransferase OS=Mus musculus OX=10090 GN=Gnpat PE=1 SV=1	5	5	+	A_B	1.66998	-1.33806	0.021380605	2.528111336	-6.7288	9.40301	9.21432	10.8218	10.4717	10.5793	10.6073
A2AGL3	RYR3_MOUSE	Ryanodine receptor 3 OS=Mus musculus OX=10090 GN=Ryr3 PE=1 SV=1	2	1	+	A_B	1.61814	-0.278427	0.024091287	1.212871744	-6.32573	8.53138	8.45121	8.7879	8.75154	9.41574	9.41363
Q9Z1Z2	STRAP_MOUSE	OX=10090 GN=Strap PE=1 SV=2	3	3	+	A_B	1.60892	0.376356	0.024608209	0.770380985	6.25646	11.2107	11.1674	10.7566	10.8688	12.0224	11.3663
Q91ZX7	LRP1_MOUSE	OX=10090 GN=Lrp1 PE=1 SV=1	2	2	+	A_B	1.57932	-1.19163	0.026343896	2.284106626	-6.03868	6.46271	6.57847	7.52356	7.90087	7.21917	5.97499
Q9WTX5	SKP1_MOUSE	S-phase kinase-associated protein 1 OS=Mus musculus OX=10090 GN=Skp1 PE=1 SV=3	3	3	+	A_B	1.5782	-1.12365	0.026411922	2.178975538	-6.03066	11.043	10.6795	11.944	12.0258	11.2935	11.6393
P23492	PNPH_MOUSE	Purine nucleoside phosphorylase OS=Mus musculus OX=10090 GN=Pnp PE=1 SV=2	3	3	+	A_B	1.56876	-0.986352	0.026992307	1.981169066	-5.96274	10.0224	10.1421	11.2228	10.9144	11.0566	10.3106
P23591	FCL_MOUSE	GDP-L-fucose synthase OS=Mus musculus OX=10090 GN=Tsta3 PE=1 SV=3	3	3	+	A_B	1.56125	-0.3153	0.027463128	1.244270365	-5.90925	9.84235	9.94837	10.2046	10.2167	9.80252	10.4409
Q9D0E1	HNRPM_MOUSE	Heterogeneous nuclear ribonucleoprotein M OS=Mus musculus OX=10090 GN=Hnrnpm PE=1 SV=3	8	8	+	A_B	1.54878	-0.574329	0.028263113	1.488984773	-5.82143	11.8803	11.9873	12.4252	12.5911	13.0549	13.1357
P08032	SPTA1_MOUSE	Spectrin alpha chain erythrocytic 1 OS=Mus musculus OX=10090 GN=Spta1 PE=1 SV=3	15	15	+	A_B	1.54613	-0.565808	0.028436098	1.480216291	-5.80291	11.5216	11.6926	12.1261	12.2198	12.2703	12.2137
P51667	MLRV_MOUSE	Myosin regulatory light chain 2 ventricular/cardiac muscle isoform OS=Mus musculus OX=10090 GN=Myl2 PE=1 SV=3	8	8	+	A_B	1.53185	-1.47235	0.029386645	2.774735012	-5.70406	12.5507	12.0396	13.7313	13.8037	12.905	13.6883
Q8VIJ6	SFPQ_MOUSE	Splicing factor proline- and glutamine-rich OS=Mus musculus OX=10090 GN=Sfpq PE=1 SV=1	2	2	+	A_B	1.52527	0.361922	0.029835272	0.778127245	5.65906	10.1033	10.1293	9.81698	9.69174	10.3106	10.3987
Q9QYG0	NDRG2_MOUSE	Protein NDRG2 OS=Mus musculus OX=10090 GN=Ndrg2 PE=1 SV=1	7	7	+	A_B	1.51658	-0.883662	0.030438272	1.845052669	-5.60008	15.2622	15.1596	16.2438	15.9454	15.8113	16.2344
Q99MN9	PCCB_MOUSE	Propionyl-CoA carboxylase beta chain mitochondrial OS=Mus musculus OX=10090 GN=Pccb PE=1 SV=2	11	11	+	A_B	1.51447	-0.4984	0.030586515	1.412646019	-5.58588	14.3512	14.3092	14.9153	14.7419	14.9703	15.1236

Q9DCW4	ETFB_MOUSE	Electron transfer flavoprotein subunit beta OS=Mus musculus OX=10090 GN=Etfb PE=1 SV=3	4	4	+	A_B	1.5138	-0.700689	0.030633738	1.625280806	-5.58133	17.4576	17.7073	18.2967	18.2696	18.2232	18.2832
Q8VDM4	PSMD2_MOUSE	26S proteasome non-ATPase regulatory subunit 2 OS=Mus musculus OX=10090 GN=Psmd2 PE=1 SV=1	6	6	+	A_B	1.50117	-1.12808	0.031537699	2.185676674	-5.49695	9.53528	9.66178	10.5314	10.9218	10.0084	9.82177
A2AAJ9	OBSCN_MOUSE	Obscurin OS=Mus musculus OX=10090 GN=Obscn PE=1 SV=3	50	50	+	A_B	1.48455	-0.251754	0.032768005	1.190653809	-5.38759	15.5111	15.4186	15.7233	15.7099	16.0868	16.0575
P17710	HXK1_MOUSE	Hexokinase-1 OS=Mus musculus OX=10090 GN=Hk1 PE=1 SV=3	7	7	+	A_B	1.47399	-0.530436	0.033574534	1.444365634	-5.31912	12.2408	12.4357	12.8899	12.8474	12.8238	13.169
P56399	UBP5_MOUSE	Ubiquitin carboxyl-terminal hydrolase 5 OS=Mus musculus OX=10090 GN=Usp5	7	7	+	A_B	1.46269	-0.736024	0.034459582	1.665579249	-5.24677	13.6096	13.3443	14.1674	14.2586	14.3581	14.2732
Q91YM4	FAKD4_MOUSE	FAST kinase domain-containing protein 4 OS=Mus musculus OX=10090	2	2	+	A_B	1.4568	-1.38703	0.034930114	2.615397082	-5.20937	8.26209	8.41363	9.46964	9.98014	9.22159	9.45327
Q9DCV4	RMD1_MOUSE	Regulator of microtubule dynamics protein 1 OS=Mus musculus OX=10090	2	2	+	A_B	1.44448	0.581348	0.035935195	0.668339015	5.13199	12.9961	12.8553	12.2556	12.4331	13.1	13.7524
P56501	UCP3_MOUSE	Mitochondrial uncoupling protein 3 OS=Mus musculus OX=10090 GN=Ucp3	3	3	+	A_B	1.418	-1.066	0.038194427	2.093620564	-4.96907	12.0014	12.4305	13.2834	13.2805	12.577	11.4818
Q91VT4	CBR4_MOUSE	Carbonyl reductase family member 4 OS=Mus musculus OX=10090 GN=Cbr4	5	5	+	A_B	1.40742	-0.763546	0.039136321	1.697658175	-4.90528	11.5068	11.5887	12.4615	12.1611	11.933	12.4919
P35564	CALX_MOUSE	Calnexin OS=Mus musculus OX=10090 GN=Canx PE=1 SV=1	4	4	+	A_B	1.3923	-0.936227	0.040522852	1.913517374	-4.81539	12.4331	12.5093	13.5981	13.2167	13.7313	13.0345
Q60864	STIP1_MOUSE	Stress-induced-phosphoprotein 1 OS=Mus musculus OX=10090 GN=Stip1 PE=1 SV=1	5	5	+	A_B	1.38785	-0.928177	0.040940204	1.902869996	-4.78917	11.2228	10.8376	11.9366	11.9801	11.4666	11.6165
P00920	CAH2_MOUSE	Carbonic anhydrase 2 OS=Mus musculus OX=10090 GN=Ca2 PE=1 SV=4	4	4	+	A_B	1.38773	-2.21398	0.040951518	4.6395343	-4.78848	11.4409	10.6073	13.0379	13.4383	12.8975	10.7649
P70168	IMB1_MOUSE	Importin subunit beta-1 OS=Mus musculus OX=10090 GN=Kpnb1 PE=1 SV=2	6	6	+	A_B	1.38759	-1.2172	0.040964721	2.324950499	-4.78769	11.9144	11.4094	12.8494	12.9088	12.4226	12.7313
Q9JI39	ABCBA_MOUSE	ATP-binding cassette sub-family B member 10 mitochondrial OS=Mus musculus OX=10090 GN=Abcb10 PE=1 SV=1	4	4	+	A_B	1.38014	-1.62376	0.041673502	3.081771722	-4.74411	9.65105	10.1799	11.7566	11.3219	12.5142	11.8297
Q62165	DAG1_MOUSE	Dystroglycan OS=Mus musculus OX=10090 GN=Dag1 PE=1 SV=4	3	3	+	A_B	1.37991	-0.98923	0.041695578	1.9851252	-4.7428	10.9218	10.7649	12.0258	11.6393	12.158	11.3554
P12382	PFKAL_MOUSE	GN=Pfkl PE=1 SV=4	4	4	+	A_B	1.37366	-0.626094	0.042299964	1.543380732	-4.70656	11.6073	11.3826	12.1923	12.0498	11.9979	11.3106
Q8K2I3	FMO2_MOUSE	Dimethylaniline monooxygenase [N-oxide-forming] 2 OS=Mus musculus OX=10090 GN=Fmo2 PE=1 SV=3	2	2	+	A_B	1.37079	0.875666	0.042580426	0.545002216	4.68996	11.0901	11.2877	10.1548	10.4717	10.5699	10.6707
O09174	AMACR_MOUSE	Alpha-methylacyl-CoA racemase OS=Mus musculus OX=10090 GN=Amacr PE=1 SV=4	5	5	+	A_B	1.35218	-0.237444	0.044444702	1.178902168	-4.58383	12.1066	12.158	12.4147	12.3247	11.9403	12.1066
P40336	VP26A_MOUSE	Vacuolar protein sorting-associated protein 26A OS=Mus musculus OX=10090	5	5	+	A_B	1.34156	-1.05359	0.045544926	2.075688567	-4.52416	10.9069	11.1163	12.2732	11.8572	11.7056	11.8376
Q91WD5	NDUS2_MOUSE	NADH dehydrogenase [ubiquinone] iron-sulfur protein 2 mitochondrial OS=Mus musculus OX=10090 GN=Ndufs2 PE=1 SV=1	6	6	+	A_B	1.33191	-0.84458	0.046568259	1.79574189	-4.47056	11.5934	11.2586	12.3581	12.183	13.3443	13.183
Q9CWX2	CIA30_MOUSE	Complex I intermediate-associated protein 30 mitochondrial OS=Mus musculus OX=10090 GN=Ndufaf1 PE=1 SV=2	3	3	+	A_B	1.32012	-1.02511	0.047849786	2.035114548	-4.40579	9.8009	9.35975	10.6795	10.5314	9.96434	9.84706
Q99K41	EMIL1_MOUSE	EMILIN-1 OS=Mus musculus OX=10090 GN=Emilin1 PE=1 SV=1	2	2	+	A_B	1.31195	-0.760081	0.048758462	1.693585708	-4.36141	6.63372	6.6865	7.24793	7.59246	6.17393	6.80872
Q9D1D4	TMEDA_MOUSE	Transmembrane emp24 domain-containing protein 10 OS=Mus musculus OX=10090 GN=Tmed10 PE=1 SV=1	2	2	+	A_B	1.31161	-1.84454	0.048796649	3.591384204	-4.35953	8.14975	7.52356	9.96578	9.3966	9.98014	9.37504
Q9D7A8	ARMC1_MOUSE	Armadillo repeat-containing protein 1 OS=Mus musculus OX=10090 GN=Armc1 PE=1 SV=1	3	3	+	A_B	1.30921	-1.60271	0.049067056	3.037132816	-4.3466	9.44708	9.62571	10.7814	11.4969	11.5699	11.5216
P48722	HS74L_MOUSE	Heat shock 70 kDa protein 4L OS=Mus musculus OX=10090 GN=Hspa4l PE=1 SV=2	4	4	+	A_B	1.30759	-0.464736	0.049250427	1.380064791	-4.33785	10.4615	10.4818	11.043	10.8297	10.8842	11.2527
Q9D6Y9	GLGB_MOUSE	1 4-alpha-glucan-branching enzyme OS=Mus musculus OX=10090 GN=Gbe1 PE=1 SV=1	7	7	+	A_B	1.3055	-0.938919	0.049488011	1.917091239	-4.32659	10.4512	10.3987	11.5793	11.1485	11.1799	10.42

Table S3. List of quantified proteins significantly different between group C vs. A from which GO analysis in the lower part of Table 1 is derived.

T: Accession	T: Entry	T: Description C/A	T: #Peptides	T: #Unique	C: Student's T-test Significant A_C	C: Student's T-test significant	N: -Log Student's T-test p-value A_C	N: Student's T-test Difference A_C	p value	C/A	N: Student's T-test Test statistic A_C	A4	A6	B21	B20	C34	C35
Q8CDN6	TXNL1_MOUS	Thioredoxin-like protein 1 OS=Mus musculus OX=10090 GN=Txnl1 PE=1 SV=3	2	2	+	A_C	4.00278	-1.46047	9.93619E-05	2.751980028	-100.313	7.98868	8.01123	8.3837	8.79766	9.46964	9.45121
A2ASS6	TITIN_MOUSE	Titin OS=Mus musculus OX=10090 GN=Ttn PE=1 SV=1	516	516	+	A_C	3.52091	-0.37571	0.000301363	1.297477931	-57.5913	21.1825	21.1704	21.5592	21.3962	21.5545	21.5498
Q9QZE5	COPG1_MOUS	Coatomer subunit gamma-1 OS=Mus musculus OX=10090 GN=Copg1 PE=1 SV=1	2	2	+	A_C	3.29336	-2.51828	0.000508909	5.728986746	-44.3114	6.54844	6.53957	6.78136	4.66107	9.00562	9.11894
A2AGL3	RYR3_MOUSE	Ryanodine receptor 3 OS=Mus musculus OX=10090 GN=Ryr3 PE=1 SV=1	2	1	+	A_C	2.72572	-0.923388	0.001880529	1.896563926	-23.0276	8.53138	8.45121	8.7879	8.75154	9.41574	9.41363
Q9D7A8	ARMC1_MOU	Armadillo repeat-containing protein 1 OS=Mus musculus OX=10090 GN=Armc1 PE=1 SV=1	3	3	+	A_C	2.67507	-2.00933	0.002113148	4.025952079	-21.7192	9.44708	9.62571	10.7814	11.4969	11.5699	11.5216
Q9D0E1	HNRPM_MOU	Heterogeneous nuclear ribonucleoprotein M OS=Mus musculus OX=10090 GN=Hnrnpm PE=1 SV=3	8	8	+	A_C	2.48011	-1.16152	0.003310473	2.236929828	-17.3371	11.8803	11.9873	12.4252	12.5911	13.0549	13.1357
Q8K1M6	DNM1L_MOU	Dynamin-1-like protein OS=Mus musculus OX=10090 GN=Dnm1l PE=1 SV=2	12	12	+	A_C	2.44455	-1.25832	0.00359294	2.392170135	-16.638	12.0293	11.9549	13.2061	12.7228	13.1846	13.3163
Q8CC88	VWA8_MOUS	von Willebrand factor A domain-containing protein 8 OS=Mus musculus OX=10090 GN=Vwa8 PE=1 SV=2	24	24	+	A_C	2.42153	-0.618814	0.003788524	1.535612278	-16.2004	14.4383	14.5142	14.8582	14.4186	15.0909	15.0992
P46935	NEDD4_MOU	E3 ubiquitin-protein ligase NEDD4 OS=Mus musculus OX=10090 GN=Nedd4 PE=1 SV=3	5	5	+	A_C	2.33593	-0.568904	0.004613919	1.483396222	-14.671	10.6073	10.5887	11.0293	10.5018	11.2046	11.1293
P98192	GNPAT_MOU	Dihydroxyacetone phosphate acyltransferase OS=Mus musculus OX=10090 GN=Gnpat PE=1 SV=1	5	5	+	A_C	2.26223	-1.28466	0.005467263	2.436246309	-13.4687	9.40301	9.21432	10.8218	10.4717	10.5793	10.6073
Q9EQK5	MVP_MOUSE	Major vault protein OS=Mus musculus OX=10090 GN=Mvp PE=1 SV=4	7	7	+	A_C	2.22121	-0.712732	0.006008831	1.638904738	-12.8422	11.5314	11.4305	12.2408	11.6926	12.2167	12.1706
A2AAJ9	OBSCN_MOU	Obscurin OS=Mus musculus OX=10090 GN=Obscn PE=1 SV=3	50	50	+	A_C	2.19915	-0.607253	0.006321935	1.523355861	-12.5172	15.5111	15.4186	15.7233	15.7099	16.0868	16.0575
P50396	GDIA_MOUSE	Rab GDP dissociation inhibitor alpha OS=Mus musculus OX=10090 GN=Gdi1 PE=1 SV=3	5	4	+	A_C	2.11028	-0.748717	0.007757468	1.680297864	-11.2876	12.1357	12.1131	12.2258	11.5459	12.8078	12.9385
Q62188	DPYL3_MOUS	Dihydropyrimidinase-related protein 3 OS=Mus musculus OX=10090 GN=Dpysl3 PE=1 SV=1	5	4	+	A_C	2.07023	-0.855741	0.008506874	1.809688012	-10.7728	9.76321	9.90989	10.5216	10.1548	10.7228	10.6618
Q62000	MIME_MOUS	Mimecan OS=Mus musculus OX=10090 GN=Ogn PE=1 SV=1	2	2	+	A_C	2.06675	-5.16453	0.008575313	35.86562832	-10.7292	5.35403	4.97728	9.05799	9.30378	9.88722	10.7731
Q8QZS1	HIBCH_MOUS	3-hydroxyisobutyryl-CoA hydrolase mitochondrial OS=Mus musculus OX=10090 GN=Hibch PE=1 SV=1	3	3	+	A_C	2.04536	-2.02525	0.009008241	4.070624093	-10.4648	12.5887	12.8803	14.183	13.5507	14.887	14.6325
Q3TZZ7	ESYT2_MOUS	Extended synaptotagmin-2 OS=Mus musculus OX=10090 GN=Esyt2 PE=1 SV=1	2	2	+	A_C	2.02828	-1.36938	0.009369577	2.583595119	-10.2583	7.7211	7.46761	8.47573	8.52748	8.92184	9.00562
Q91WD5	NDUS2_MOU	NADH dehydrogenase [ubiquinone] iron-sulfur protein 2 mitochondrial OS=Mus musculus OX=10090 GN=Ndufs2 PE=1 SV=1	6	6	+	A_C	1.99694	-1.83768	0.010070708	3.574347746	-9.88943	11.5934	11.2586	12.3581	12.183	13.3443	13.183
Q9CZW5	TOM70_MOU	Mitochondrial import receptor subunit TOM70 OS=Mus musculus OX=10090 GN=Tomm70 PE=1 SV=2	5	5	+	A_C	1.97585	-1.25983	0.010571826	2.394675216	-9.64854	10.0901	9.87958	10.7895	11.6257	11.1674	11.3219
Q99MN9	PCCB_MOUSE	Propionyl-CoA carboxylase beta chain mitochondrial OS=Mus musculus OX=10090 GN=Pccb PE=1 SV=2	11	11	+	A_C	1.91791	-0.716757	0.012080642	1.643483532	-9.01555	14.3512	14.3092	14.9153	14.7419	14.9703	15.1236
Q99KF1	TMED9_MOU	Transmembrane emp24 domain-containing protein 9 OS=Mus musculus OX=10090 GN=Tmed9 PE=1 SV=2	2	2	+	A_C	1.91358	-0.932148	0.01220169	1.908114837	-8.96985	10.0634	9.88417	9.67948	10.5699	10.8533	10.9586
P54071	IDHP_MOUSE	Isocitrate dehydrogenase [NADP] mitochondrial OS=Mus musculus OX=10090 GN=Idh2 PE=1 SV=3	10	10	+	A_C	1.90374	-0.427423	0.012481305	1.344829242	-8.86693	17.68	17.7406	18.1801	17.6937	18.1002	18.1752
Q99MN1	SYK_MOUSE	Lysine--tRNA ligase OS=Mus musculus OX=10090 GN=Kars1 PE=1 SV=1	3	3	+	A_C	1.90346	-0.404115	0.012489355	1.323276917	-8.86406	10.6795	10.5981	11.4666	10.8994	11.0224	11.0634
O54724	CAVN1_MOU	Caveolae-associated protein 1 OS=Mus musculus OX=10090 GN=Cavin1 PE=1 SV=1	6	6	+	A_C	1.86694	-1.01323	0.013585011	2.018425027	-8.49201	15.5356	15.435	15.6828	15.2805	16.3904	16.6068
Q9DC69	NDUA9_MOU	NADH dehydrogenase [ubiquinone] 1 alpha subcomplex subunit 9 mitochondrial OS=Mus musculus OX=10090 GN=Ndufa9 PE=1 SV=2	6	6	+	A_C	1.85234	-0.952422	0.014049472	1.935118614	-8.34745	16.5507	16.6382	17.3667	17.0847	17.6523	17.4415
Q5SW19	CLU_MOUSE	Clustered mitochondria protein homolog OS=Mus musculus OX=10090 GN=Cluh PE=1 SV=2	6	6	+	A_C	1.82675	-1.02027	0.014902187	2.02829852	-8.09985	11.4409	11.4969	11.5411	11.5507	12.6119	12.3663
Q9CWD8	NUBPL_MOU	Iron-sulfur protein NUBPL OS=Mus musculus OX=10090 GN=Nubpl PE=1 SV=2	2	2	+	A_C	1.806	-1.35197	0.015631476	2.552604457	-7.90427	8.29002	8.11374	9.50184	8.91886	9.70044	9.40727
O55106	STRN_MOUSE	Striatin OS=Mus musculus OX=10090 GN=Strn PE=1 SV=2	3	3	+	A_C	1.80491	-1.05294	0.015670758	2.074753585	-7.8941	8.73132	8.69697	8.84862	8.54689	9.63481	9.89936
Q02788	CO6A2_MOU	Collagen alpha-2(VI) chain OS=Mus musculus OX=10090 GN=Col6a2 PE=1 SV=3	8	8	+	A_C	1.78732	0.252648	0.016318491	0.839354404	7.73202	13.92	13.8727	13.2423	14.0701	13.6211	13.6662
P51125	ICAL_MOUSE	Calpastatin OS=Mus musculus OX=10090 GN=Cast PE=1 SV=2	3	3	+	A_C	1.76777	0.661633	0.017069862	0.632162341	7.55564	10.6348	10.6707	10.4512	9.57175	9.90539	10.0768
Q9DB20	ATPO_MOUSE	ATP synthase subunit O mitochondrial OS=Mus musculus OX=10090 GN=Atp5po PE=1 SV=1	2	2	+	A_C	1.75214	-0.802829	0.017695384	1.744518625	-7.41726	14.726	14.8533	16.9083	16.0617	15.68	15.5049

Q91YM4	FAKD4_MOUS	FAST kinase domain-containing protein 4 OS=Mus musculus OX=10090 GN=Tbrg4 PE=1 SV=1	2	2	+	A_C	1.72951	-0.999568	0.018641893	1.99940121	-7.2213	8.26209	8.41363	9.46964	9.98014	9.22159	9.45327
G5E8K5	ANK3_MOUSE	Ankyrin-3 OS=Mus musculus OX=10090 GN=Ank3 PE=1 SV=1	3	1	+	A_C	1.727	-1.80523	0.018749945	3.494848701	-7.19991	6.75489	6.54998	8.74819	7.62936	8.6865	8.22882
P08032	SPTA1_MOUS	Spectrin alpha chain erythrocytic 1 OS=Mus musculus OX=10090 GN=Spta1 PE=1 SV=3	15	15	+	A_C	1.70915	-0.634895	0.019536646	1.552824731	-7.0492	11.5216	11.6926	12.1261	12.2198	12.2703	12.2137
Q9JIQ3	DBLOH_MOU	Diablo homolog mitochondrial OS=Mus musculus OX=10090 GN=Diablo PE=1 SV=2	3	3	+	A_C	1.67739	-0.757741	0.021018901	1.690840999	-6.78833	8.81378	9.01959	9.53916	8.41785	9.71768	9.63118
P49442	INPP_MOUSE	Inositol polyphosphate 1-phosphatase OS=Mus musculus OX=10090 GN=Inpp1 PE=1 SV=2	3	3	+	A_C	1.66791	-0.58572	0.021482756	1.500787795	-6.71228	9.9542	9.95128	10.8533	10.2877	10.6257	10.4512
P29758	OAT_MOUSE	Ornithine aminotransferase mitochondrial OS=Mus musculus OX=10090 GN=Oat PE=1 SV=1	7	7	+	A_C	1.65396	-2.48523	0.022184007	5.599236043	-6.60177	13.6883	12.944	15.9882	15.1869	15.8582	15.7445
P27546	MAP4_MOUS	Microtubule-associated protein 4 OS=Mus musculus OX=10090 GN=Map4 PE=1 SV=3	7	7	+	A_C	1.65392	-0.831233	0.022186051	1.779205311	-6.6014	11.7356	11.6027	12.1033	11.9293	12.6073	12.3934
P54116	STOM_MOUS	Erythrocyte band 7 integral membrane protein OS=Mus musculus OX=10090 GN=Stom PE=1 SV=3	3	3	+	A_C	1.64148	-0.37578	0.022830741	1.297540887	-6.50437	12.5911	12.4767	12.4486	11.6073	12.9012	12.9181
P40336	VP26A_MOUS	Vacuolar protein sorting-associated protein 26A OS=Mus musculus OX=10090 GN=Vps26a PE=1 SV=1	5	5	+	A_C	1.59316	-0.760013	0.02551761	1.693505885	-6.13966	10.9069	11.1163	12.2732	11.8572	11.7056	11.8376
P98156	VLDLR_MOUS	Very low-density lipoprotein receptor OS=Mus musculus OX=10090 GN=Vldlr PE=1 SV=1	3	3	+	A_C	1.58565	-0.767229	0.025962709	1.701997597	-6.08465	9.16992	9.23122	9.29692	9.00562	9.84549	10.0901
Q68FD5	CLH1_MOUSE	Clathrin heavy chain 1 OS=Mus musculus OX=10090 GN=Cltc PE=1 SV=3	23	23	+	A_C	1.58553	-0.521711	0.025969883	1.435656892	-6.08383	13.5507	13.3987	14.1984	13.8727	13.9567	14.0362
Q8CI94	PYGB_MOUSE	Glycogen phosphorylase brain form OS=Mus musculus OX=10090 GN=Pygb PE=1 SV=3	18	14	+	A_C	1.57943	-1.14986	0.026337224	2.218923608	-6.03948	15.5204	15.1397	16.8965	16.3701	16.4752	16.4846
P56399	UBP5_MOUSE	Ubiquitin carboxyl-terminal hydrolase 5 OS=Mus musculus OX=10090 GN=Usp5 PE=1 SV=1	7	7	+	A_C	1.57685	-0.838689	0.026494151	1.788424234	-6.0209	13.6096	13.3443	14.1674	14.2586	14.3581	14.2732
Q02257	PLAK_MOUSE	Junction plakoglobin OS=Mus musculus OX=10090 GN=Jup PE=1 SV=3	7	7	+	A_C	1.57127	-1.25751	0.026836755	2.39082743	-5.98076	10.6528	11.0634	11.5603	11.8533	12.1611	12.0701
Q8BND5	QSOX1_MOU	Sulfhydryl oxidase 1 OS=Mus musculus OX=10090 GN=Qsox1 PE=1 SV=1	2	2	+	A_C	1.56872	-0.804775	0.026994793	1.746873332	-5.96247	9.46964	9.20457	9.8933	10.6883	10.1674	10.1163
Q9JJW5	MYOZ2_MOU	Myozenin-2 OS=Mus musculus OX=10090 GN=Myoz2 PE=1 SV=1	6	6	+	A_C	1.55578	-0.903382	0.027811217	1.870445591	-5.87059	15.8942	15.5893	16.3127	16.0232	16.624	16.6662
Q64310	SURF4_MOUS	Surfeit locus protein 4 OS=Mus musculus OX=10090 GN=Surf4 PE=1 SV=1	2	2	+	A_C	1.53466	-1.24603	0.029197119	2.37187832	-5.72343	10.2288	10.6439	11.9586	10.9873	11.7482	11.6165
Q9D0K2	SCOT1_MOUS	Succinyl-CoA:3-ketoacid coenzyme A transferase 1 mitochondrial OS=Mus musculus OX=10090 GN=Oxct1 PE=1 SV=1	6	6	+	A_C	1.5123	-0.943067	0.030739727	1.922611142	-5.57125	15.8533	15.5805	16.5597	15.5049	16.7602	16.5597
P01867	IGG2B_MOUS	Ig gamma-2B chain C region OS=Mus musculus OX=10090 GN=Igh-3 PE=1 SV=3	3	3	+	A_C	1.50181	-2.38273	0.031491257	5.215226821	-5.50117	10.6348	10.3106	10.0634	11.8494	13.2571	12.4538
Q9D6J5	NDUB8_MOU	NADH dehydrogenase [ubiquinone] 1 beta subcomplex subunit 8 mitochondrial OS=Mus musculus OX=10090 GN=Ndufb8 PE=1 SV=1	5	5	+	A_C	1.50162	-0.727944	0.031505037	1.656277031	-5.49991	14.8337	14.8386	15.691	15.8582	15.4318	15.6964
P62702	RS4X_MOUSE	40S ribosomal protein S4 X isoform OS=Mus musculus OX=10090 GN=Rps4x PE=1 SV=2	2	2	+	A_C	1.49941	-1.38552	0.031665766	2.612661103	-5.48523	11.3554	11.7731	12.664	12.0049	12.8078	13.0918
P47811	MK14_MOUS	Mitogen-activated protein kinase 14 OS=Mus musculus OX=10090 GN=Mapk14 PE=1 SV=3	3	3	+	A_C	1.4956	-0.510536	0.031944787	1.424579367	-5.46008	8.37504	8.30834	9.66711	8.42626	8.76487	8.93958
O88844	IDHC_MOUSE	Isocitrate dehydrogenase [NADP] cytoplasmic OS=Mus musculus OX=10090 GN=Idh1 PE=1 SV=2	4	4	+	A_C	1.49431	-1.19678	0.032039815	2.29227479	-5.45155	11.9293	11.6303	12.6684	11.8572	12.8158	13.1373
Q9WUR2	ECI2_MOUSE	Enoyl-CoA delta isomerase 2 OS=Mus musculus OX=10090 GN=Eci2 PE=1 SV=2	3	3	+	A_C	1.49005	-0.922753	0.03235564	1.89572934	-5.42352	14.2732	13.9476	14.3373	14.7834	15.0826	14.9837
Q61937	NPM_MOUSE	Nucleophosmin OS=Mus musculus OX=10090 GN=Npm1 PE=1 SV=1	2	2	+	A_C	1.47736	-0.878369	0.033315014	1.83829589	-5.3409	10.4919	10.7313	11.4252	11.1737	11.6027	11.3772
Q9DCW4	ETFB_MOUSE	Electron transfer flavoprotein subunit beta OS=Mus musculus OX=10090 GN=Etfb PE=1 SV=3	4	4	+	A_C	1.45931	-0.67074	0.034728818	1.591889284	-5.22528	17.4576	17.7073	18.2967	18.2696	18.2232	18.2832
Q9JI39	ABCBA_MOUS	ATP-binding cassette sub-family B member 10 mitochondrial OS=Mus musculus OX=10090 GN=Abcb10 PE=1 SV=1	4	4	+	A_C	1.45806	-2.25649	0.034828919	4.778275376	-5.21731	9.65105	10.1799	11.7566	11.3219	12.5142	11.8297
Q8VIJ6	SFPQ_MOUSE	Splicing factor proline- and glutamine-rich OS=Mus musculus OX=10090 GN=Sfpq PE=1 SV=1	2	2	+	A_C	1.45357	-0.238393	0.03519087	1.179677901	-5.18895	10.1033	10.1293	9.81698	9.69174	10.3106	10.3987
Q6PER3	MARE3_MOU	Microtubule-associated protein RP/EB family member 3 OS=Mus musculus OX=10090 GN=Mapre3 PE=1 SV=1	2	2	+	A_C	1.45132	-1.09492	0.03537366	2.136012367	-5.1748	9.0634	8.71425	9.19229	9.28309	10.1033	9.86419
Q8K2I3	FMO2_MOUS	Dimethylaniline monooxygenase [N-oxide-forming] 2 OS=Mus musculus OX=10090 GN=Fmo2 PE=1 SV=3	2	2	+	A_C	1.4437	0.568656	0.035999793	0.674244615	5.12708	11.0901	11.2877	10.1548	10.4717	10.5699	10.6707
Q91VH6	MEMO1_MOU	Protein MEMO1 OS=Mus musculus OX=10090 GN=Memo1 PE=1 SV=1	2	2	+	A_C	1.4406	-0.653924	0.036257679	1.573442001	-5.1078	8.52748	8.66178	9.22882	9.15482	9.35755	9.13955
P63330	PP2AA_MOUS	Serine/threonine-protein phosphatase 2A catalytic subunit alpha isoform OS=Mus musculus OX=10090 GN=Ppp2ca PE=1 SV=1	6	1	+	A_C	1.43772	-0.944699	0.036498919	1.924787262	-5.0899	11.6393	11.9476	9.44087	11.446	12.6348	12.8416
Q8R1B4	EIF3C_MOUSE	Eukaryotic translation initiation factor 3 subunit C OS=Mus musculus OX=10090 GN=Eif3c PE=1 SV=1	2	2	+	A_C	1.42567	-0.402625	0.037525803	1.321910956	-5.01575	10.1293	10.0362	11.2703	10.7228	10.5507	10.42
Q9CZS1	AL1B1_MOUS	Aldehyde dehydrogenase X mitochondrial OS=Mus musculus OX=10090 GN=Aldh1b1 PE=1 SV=1	6	6	+	A_C	1.41644	-0.959572	0.038331869	1.944732871	-4.95962	11.4512	11.1485	11.3663	11.6883	12.1389	12.3799

Q99MI1	RB6I2_MOUSI	ELKS/Rab6-interacting/CAST family member 1 OS=Mus musculus OX=10090 GN=Erc1 PE=1 SV=1	2	2	+	A_C	1.41132	-0.764175	0.038786447	1.698398497	-4.92872	9	8.69	9.11634	8.69697	9.60548	9.61287
P80317	TCPZ_MOUSE	T-complex protein 1 subunit zeta OS=Mus musculus OX=10090 GN=Cct6a PE=1 SV=3	3	3	+	A_C	1.40763	-0.451151	0.039117402	1.367130535	-4.90654	10.0362	10.1799	11.1033	9.15228	10.5018	10.6165
P70333	HNRH2_MOU:	Heterogeneous nuclear ribonucleoprotein H2 OS=Mus musculus OX=10090 GN=Hnrnph2 PE=1 SV=1	3	1	+	A_C	1.38931	-1.32563	0.040802803	2.50642315	-4.79774	9.05257	8.53528	9.89785	9.69697	10.0224	10.2167
Q91YE8	SYNP2_MOUS	Synaptopodin-2 OS=Mus musculus OX=10090 GN=Synpo2 PE=1 SV=2	9	9	+	A_C	1.38441	-0.484961	0.041265774	1.399548031	-4.76901	12.8688	12.6707	12.7875	12.5191	13.2318	13.2776
Q80XB4	NRAP_MOUSI	Nebulin-related-anchoring protein OS=Mus musculus OX=10090 GN=Nrap PE=1 SV=3	9	9	+	A_C	1.38179	-0.763886	0.041515474	1.698058309	-4.75374	12.9819	13.3021	13.6662	13.4893	13.8918	13.92
Q60854	SPB6_MOUSE	Serpin B6 OS=Mus musculus OX=10090 GN=Serpinb6 PE=1 SV=1	6	6	+	A_C	1.37591	-0.776038	0.042081383	1.712421673	-4.71954	12.2408	11.973	12.1453	12.3853	12.7875	12.9784
Q9CQ54	NDUC2_MOU:	NADH dehydrogenase [ubiquinone] 1 subunit C2 OS=Mus musculus OX=10090 GN=Ndufc2 PE=1 SV=1	2	2	+	A_C	1.3662	-1.67183	0.043032839	3.186184919	-4.66362	12.2288	12.1706	13.1861	13.9567	13.5142	14.2288
P48036	ANXA5_MOU:	Annexin A5 OS=Mus musculus OX=10090 GN=Anxa5 PE=1 SV=1	4	4	+	A_C	1.36488	-0.8661	0.043163833	1.822728899	-4.65605	13.6883	13.3163	14.0102	13.6439	14.365	14.3718
Q5SRX1	TM1L2_MOU:	TOM1-like protein 2 OS=Mus musculus OX=10090 GN=Tom1l2 PE=1 SV=1	2	2	+	A_C	1.36215	-0.779094	0.043436018	1.716052868	-4.64042	7.40939	7.72792	8.24317	7.35755	8.29462	8.40088
Q11011	PSA_MOUSE	Puromycin-sensitive aminopeptidase OS=Mus musculus OX=10090 GN=Npepps PE=1 SV=2	6	6	+	A_C	1.35549	-0.654981	0.044107252	1.574595216	-4.60256	9.96578	9.75154	11.0084	10.2167	10.42	10.6073
Q8BKE9	IFT74_MOUSI	Intraflagellar transport protein 74 homolog OS=Mus musculus OX=10090 GN=Ift74 PE=1 SV=2	2	2	+	A_C	1.34424	-0.870433	0.045264737	1.828211524	-4.53915	8.77149	9.07146	9.09276	8.50779	9.91139	9.67243
Q9WVJ2	PSD13_MOUS	26S proteasome non-ATPase regulatory subunit 13 OS=Mus musculus OX=10090 GN=Psmd13 PE=1 SV=1	5	5	+	A_C	1.33625	-0.497382	0.04610521	1.411649574	-4.49456	12.2732	12.0532	12.4818	12.5627	12.6484	12.6729
Q9CXI0	COQ5_MOUSI	2-methoxy-6-polyprenyl-1 4-benzoquinol methylase mitochondrial OS=Mus musculus OX=10090 GN=Coq5 PE=1 SV=2	4	4	+	A_C	1.32917	-0.623242	0.046862991	1.540332705	-4.45543	11.8138	12.0154	13.1611	12.1674	12.6348	12.4409
Q9QXS1	PLEC_MOUSE	Plectin OS=Mus musculus OX=10090 GN=Plec PE=1 SV=3	46	46	+	A_C	1.3256	-0.320806	0.047249803	1.249028157	-4.43581	16.1965	16.0868	16.4087	16.3056	16.4153	16.5096
P35486	ODPA_MOUSI	Pyruvate dehydrogenase E1 component subunit alpha somatic form mitochondrial OS=Mus musculus OX=10090 GN=Pdha1 PE=1 SV=1	6	6	+	A_C	1.30857	-0.963436	0.049139417	1.949948471	-4.34316	15.3443	14.9703	16.0102	15.3512	16.0014	16.24
P09541	MYL4_MOUSI	Myosin light chain 4 OS=Mus musculus OX=10090 GN=Myl4 PE=1 SV=3	4	4	+	A_C	1.30608	-1.24452	0.049421964	2.369397087	-4.32973	12.0327	11.5887	11.8727	12.1984	12.8727	13.2378

Table S4. List of quantified proteins significantly different between hearts of mice treated with (B and C) or without (A) 2DG intermittently for 8 months. Data used for GO analysis of biological processes and volcano plots, Table 2 and Figure 1, respectively.

T: Accession	T: Entry	T: Description (B + C)/A	#Peptides	#Unique	C: Student's T-test <0.05	C: Student's T	N: -Log Student's T-test p-value Unt_Treated	N: Student's T-test Difference Unt_Treated	p value	Treated/Ut	N: Student's T-test Test statistic Unt_Treated
Q91VR5	DDX1_M	ATP-dependent RNA helicase DDX1 OS=Mus musculus OX=10090 GN=Ddx1 PE=1 SV=1	3	3	+	Unt_Treated	1.9373	2.40136	0.01155314	0.18928605	4.41528
A2AN08	UBR4_M	E3 ubiquitin-protein ligase UBR4 OS=Mus musculus OX=10090 GN=Ubr4 PE=1	4	4	+	Unt_Treated	2.40953	1.00095	0.00389466	0.499670863	5.99489
Q8K2I3	FMO2_M	Dimethylaniline monooxygenase [N-oxide-forming] 2 OS=Mus musculus OX=10090 GN=Fmo2 PE=1 SV=3	2	2	+	Unt_Treated	1.81256	0.722161	0.01539714	0.606188757	4.05617
Q9DBG3	AP2B1_M	AP-2 complex subunit beta OS=Mus musculus OX=10090 GN=Ap2b1 PE=1 SV=1	3	1	+	Unt_Treated	1.50585	0.499063	0.03119967	0.707566181	3.25594
Q8R081	HNRPL_M	Heterogeneous nuclear ribonucleoprotein L OS=Mus musculus OX=10090 GN=Hnrnpl PE=1 SV=2	4	4	+	Unt_Treated	2.05482	-0.232103	0.00881414	1.174545824	-4.77401
Q9QXS1	PLEC_MO	Plectin OS=Mus musculus OX=10090 GN=Plec PE=1 SV=3	46	46	+	Unt_Treated	1.7109	-0.268162	0.01945808	1.204272601	-3.77867
A2ASS6	TITIN_MO	Titin OS=Mus musculus OX=10090 GN=Ttn PE=1 SV=1	516	516	+	Unt_Treated	2.32564	-0.338487	0.00472455	1.264429851	-5.68575
Q64471	GSTT1_M	Glutathione S-transferase theta-1 OS=Mus musculus OX=10090 GN=Gstt1	2	2	+	Unt_Treated	1.35069	-0.346004	0.04459745	1.271035213	-2.88924
Q9WVJ2	PSD13_M	26S proteasome non-ATPase regulatory subunit 13 OS=Mus musculus OX=10090 GN=Psmd13 PE=1 SV=1	5	5	+	Unt_Treated	1.98748	-0.428206	0.01029248	1.345559325	-4.56592
O35855	BCAT2_M	Branched-chain-amino-acid aminotransferase mitochondrial OS=Mus musculus OX=10090 GN=Bcat2 PE=1 SV=2	4	4	+	Unt_Treated	1.3774	-0.446025	0.04193725	1.362281641	-2.95076
P70670	NACAM_	Nascent polypeptide-associated complex subunit alpha muscle-specific form OS=Mus musculus OX=10090 GN=Naca PE=1 SV=2	19	19	+	Unt_Treated	2.34553	-0.459993	0.00451305	1.375535144	-5.75781
P47857	PFKAM_I	ATP-dependent 6-phosphofructokinase muscle type OS=Mus musculus OX=10090 GN=Pfkm PE=1 SV=3	9	9	+	Unt_Treated	1.50475	-0.517613	0.03127879	1.43158467	-3.25328
P48722	HS74L_M	Heat shock 70 kDa protein 4L OS=Mus musculus OX=10090 GN=Hspa4l PE=1 SV=2	4	4	+	Unt_Treated	1.69063	-0.530757	0.02038778	1.444687042	-3.72486
Q68FD5	CLH1_MO	Clathrin heavy chain 1 OS=Mus musculus OX=10090 GN=Cltc PE=1 SV=3	23	23	+	Unt_Treated	2.04664	-0.541262	0.00898173	1.455244939	-4.74835
Q61702	ITIH1_MO	Inter-alpha-trypsin inhibitor heavy chain H1 OS=Mus musculus OX=10090 GN=Itih1 PE=1 SV=2	3	3	+	Unt_Treated	1.3017	-0.56004	0.04992292	1.474310093	-2.77795
P14152	MDHC_M	Malate dehydrogenase cytoplasmic OS=Mus musculus OX=10090 GN=Mdh1 PE=1 SV=3	7	7	+	Unt_Treated	1.42471	-0.571765	0.03760885	1.486340855	-3.06136
P17710	HXK1_MO	Hexokinase-1 OS=Mus musculus OX=10090 GN=Hk1 PE=1 SV=3	7	7	+	Unt_Treated	1.94377	-0.59429	0.0113823	1.509729413	-4.43448
Q9EQK5	MVP_MO	Major vault protein OS=Mus musculus OX=10090 GN=Mvp PE=1 SV=4	7	7	+	Unt_Treated	1.41356	-0.599259	0.03858691	1.514938261	-3.0351
Q80XB4	NRAP_M	Nebulin-related-anchoring protein OS=Mus musculus OX=10090 GN=Nrap PE=1 SV=3	9	9	+	Unt_Treated	1.52873	-0.599837	0.02959852	1.515545326	-3.31203
P08032	SPTA1_M	Spectrin alpha chain erythrocytic 1 OS=Mus musculus OX=10090 GN=Spta1 PE=1 SV=3	15	15	+	Unt_Treated	3.01751	-0.600352	0.00096048	1.516086428	-8.70139

P49442	INPP_MO	Inositol polyphosphate 1-phosphatase OS=Mus musculus OX=10090 GN=Inpp1 PE=1 SV=2	3	3	+	Unt_Treated	1.52814	-0.601745	0.02963876	1.517550998	-3.31056
Q99MN9	PCCB_MO	Propionyl-CoA carboxylase beta chain mitochondrial OS=Mus musculus OX=10090 GN=Pccb PE=1 SV=2	11	11	+	Unt_Treated	2.1594	-0.607578	0.00692787	1.523699071	-5.11129
Q8BH61	F13A_MO	Coagulation factor XIII A chain OS=Mus musculus OX=10090 GN=F13a1 PE=1	4	4	+	Unt_Treated	1.59621	-0.623968	0.02533903	1.541108033	-3.48057
P47791	GSHR_M	Glutathione reductase mitochondrial OS=Mus musculus OX=10090 GN=Gsr PE=1 SV=3	2	2	+	Unt_Treated	2.19242	-0.624995	0.00642066	1.542205481	-5.22154
Q91VH6	MEMO1_	Protein MEMO1 OS=Mus musculus OX=10090 GN=Memo1 PE=1 SV=1	2	2	+	Unt_Treated	2.73657	-0.625557	0.00183413	1.542806362	-7.34011
P19123	TNNC1_M	Troponin C slow skeletal and cardiac muscles OS=Mus musculus OX=10090 GN=Tnnc1 PE=1 SV=1	2	2	+	Unt_Treated	1.36115	-0.634947	0.04353615	1.552880701	-2.91325
Q8CGY6	UN45B_M	Protein unc-45 homolog B OS=Mus musculus OX=10090 GN=Unc45b PE=1	5	5	+	Unt_Treated	1.46726	-0.645688	0.03409887	1.564485188	-3.16259
Q8BG32	PSD11_M	26S proteasome non-ATPase regulatory subunit 11 OS=Mus musculus OX=10090 GN=Psmd11 PE=1 SV=3	4	4	+	Unt_Treated	1.71035	-0.647996	0.01948274	1.566990029	-3.7772
P05201	AATC_MO	Aspartate aminotransferase cytoplasmic OS=Mus musculus OX=10090 GN=Got1 PE=1 SV=3	5	5	+	Unt_Treated	1.33517	-0.664185	0.04622001	1.584672815	-2.85376
Q62188	DPYL3_M	Dihydropyrimidinase-related protein 3 OS=Mus musculus OX=10090 GN=Dpysl3 PE=1 SV=1	5	4	+	Unt_Treated	1.58792	-0.678698	0.02582736	1.600694512	-3.45959
Q9DCW4	ETFB_MO	Electron transfer flavoprotein subunit beta OS=Mus musculus OX=10090 GN=Etfb PE=1 SV=3	4	4	+	Unt_Treated	2.99038	-0.685715	0.0010224	1.608498956	-8.5606
Q91VT4	CBR4_MO	Carbonyl reductase family member 4 OS=Mus musculus OX=10090 GN=Cbr4 PE=1 SV=2	5	5	+	Unt_Treated	1.62788	-0.714096	0.023557	1.640454978	-3.56137
Q61207	SAP_MO	Prosaposin OS=Mus musculus OX=10090 GN=Psap PE=1 SV=2	2	2	+	Unt_Treated	2.03361	-0.715453	0.00925529	1.641998717	-4.70773
P13541	MYH3_M	Myosin-3 OS=Mus musculus OX=10090 GN=Myh3 PE=2 SV=2	12	3	+	Unt_Treated	1.30754	-0.715562	0.0492561	1.64212278	-2.79111
Q60864	STIP1_M	Stress-induced-phosphoprotein 1 OS=Mus musculus OX=10090 GN=Stip1	5	5	+	Unt_Treated	1.50719	-0.719767	0.03110355	1.646916031	-3.25923
Q9DBR7	MYPT1_M	Protein phosphatase 1 regulatory subunit 12A OS=Mus musculus OX=10090 GN=Ppp1r12a PE=1 SV=2	3	2	+	Unt_Treated	1.5469	-0.721128	0.02838573	1.64847042	-3.35693
P05132	KAPCA_M	cAMP-dependent protein kinase catalytic subunit alpha OS=Mus musculus OX=10090 GN=Prkaca PE=1 SV=3	3	3	+	Unt_Treated	1.4485	-0.765817	0.0356041	1.700332626	-3.11776
P68040	RACK1_M	Receptor of activated protein C kinase 1 OS=Mus musculus OX=10090 GN=Rack1 PE=1 SV=3	4	4	+	Unt_Treated	1.35387	-0.774983	0.04427209	1.711169888	-2.89652
Q61937	NPM_MO	Nucleophosmin OS=Mus musculus OX=10090 GN=Npm1 PE=1 SV=1	2	2	+	Unt_Treated	2.17923	-0.783114	0.00661866	1.720841235	-5.17729
P56399	UBP5_MO	Ubiquitin carboxyl-terminal hydrolase 5 OS=Mus musculus OX=10090 GN=Usp5 PE=1 SV=1	7	7	+	Unt_Treated	2.84926	-0.787357	0.00141495	1.725909719	-7.86119
Q9DC69	NDUA9_I	NADH dehydrogenase [ubiquinone] 1 alpha subcomplex subunit 9 mitochondrial OS=Mus musculus OX=10090 GN=Ndufa9 PE=1 SV=2	6	6	+	Unt_Treated	1.94857	-0.79182	0.01125719	1.731257115	-4.44878
P61202	CSN2_MO	COP9 signalosome complex subunit 2 OS=Mus musculus OX=10090 GN=Cops2 PE=1 SV=1	3	3	+	Unt_Treated	1.34516	-0.814705	0.04516895	1.75893845	-2.87657
P52480	KPYM_M	Pyruvate kinase PKM OS=Mus musculus OX=10090 GN=Pkm PE=1 SV=4	13	13	+	Unt_Treated	1.4807	-0.815856	0.03305978	1.760342313	-3.19495
Q9D6J5	NDUB8_I	NADH dehydrogenase [ubiquinone] 1 beta subcomplex subunit 8 mitochondrial OS=Mus musculus OX=10090 GN=Ndufb8 PE=1 SV=1	5	5	+	Unt_Treated	2.48856	-0.833188	0.00324668	1.781617952	-6.29884
Q8R2Q4	RRF2M_I	Ribosome-releasing factor 2 mitochondrial OS=Mus musculus OX=10090 GN=Gfm2 PE=1 SV=2	6	6	+	Unt_Treated	1.39239	-0.83823	0.04051445	1.787855329	-2.98559
Q9QYG0	NDRG2_I	Protein NDRG2 OS=Mus musculus OX=10090 GN=Ndrg2 PE=1 SV=1	7	7	+	Unt_Treated	2.17139	-0.847811	0.00673923	1.799768066	-5.15111

Q922B2	SYDC_MO	Aspartate--tRNA ligase cytoplasmic OS=Mus musculus OX=10090 GN=Dars1 PE=1 SV=2	6	6	+	Unt_Treated	1.32992	-0.853759	0.04678213	1.807203538	-2.8418
Q9WTX5	SKP1_MO	S-phase kinase-associated protein 1 OS=Mus musculus OX=10090 GN=Skp1 PE=1 SV=3	3	3	+	Unt_Treated	1.46781	-0.864402	0.03405571	1.820584875	-3.16393
Q9D0E1	HNRPM_	Heterogeneous nuclear ribonucleoprotein M OS=Mus musculus OX=10090 GN=Hnrnpm PE=1 SV=3	8	8	+	Unt_Treated	1.5264	-0.867925	0.02975774	1.825036098	-3.30628
Q99LC3	NDUAA_	NADH dehydrogenase [ubiquinone] 1 alpha subcomplex subunit 10 mitochondrial OS=Mus musculus OX=10090 GN=Ndufa10 PE=1 SV=1	7	7	+	Unt_Treated	1.41059	-0.873585	0.0388517	1.832210167	-3.02814
Q9D1X0	NOL3_M	Nucleolar protein 3 OS=Mus musculus OX=10090 GN=Nol3 PE=1 SV=1	3	3	+	Unt_Treated	1.93249	-0.876691	0.01168181	1.836159009	-4.40103
P26043	RADI_MO	Radixin OS=Mus musculus OX=10090 GN=Rdx PE=1 SV=3	4	3	+	Unt_Treated	1.90946	-0.878264	0.01231799	1.838162103	-4.33326
Q8BND5	QSOX1_M	Sulfhydryl oxidase 1 OS=Mus musculus OX=10090 GN=Qsox1 PE=1 SV=1	2	2	+	Unt_Treated	1.53101	-0.879221	0.02944354	1.839381837	-3.31762
P18242	CATD_MO	Cathepsin D OS=Mus musculus OX=10090 GN=Ctsd PE=1 SV=1	3	3	+	Unt_Treated	1.45104	-0.884954	0.03539647	1.846705739	-3.1238
Q8BML9	SYQ_MO	Glutamine--tRNA ligase OS=Mus musculus OX=10090 GN=Qars1 PE=1 SV=1	3	3	+	Unt_Treated	1.35759	-0.889644	0.04389449	1.85271889	-2.90506
P09411	PGK1_MO	Phosphoglycerate kinase 1 OS=Mus musculus OX=10090 GN=Pgk1 PE=1 SV=4	6	6	+	Unt_Treated	1.68153	-0.900005	0.02081949	1.86607245	-3.70087
Q80W93	HYDIN_M	Hydrocephalus-inducing protein OS=Mus musculus OX=10090 GN=Hydin PE=1 SV=2	2	2	+	Unt_Treated	1.56595	-0.905824	0.02716752	1.87361431	-3.40438
P40336	VP26A_M	Vacuolar protein sorting-associated protein 26A OS=Mus musculus OX=10090 GN=Vps26a PE=1 SV=1	5	5	+	Unt_Treated	2.01294	-0.906802	0.00970644	1.87488486	-4.64379
Q61879	MYH10_I	Myosin-10 OS=Mus musculus OX=10090 GN=Myh10 PE=1 SV=2	3	2	+	Unt_Treated	1.53568	-0.907629	0.02912863	1.875959914	-3.32916
Q8R4N0	CLYBL_M	Citramalyl-CoA lyase mitochondrial OS=Mus musculus OX=10090 GN=Clybl PE=1 SV=2	4	4	+	Unt_Treated	1.59122	-0.911307	0.02563185	1.880748579	-3.46792
Q9CZ13	QCR1_M	Cytochrome b-c1 complex subunit 1 mitochondrial OS=Mus musculus OX=10090 GN=Uqcrc1 PE=1 SV=2	7	7	+	Unt_Treated	1.9117	-0.91255	0.01225462	1.882369696	-4.33982
P63085	MK01_M	Mitogen-activated protein kinase 1 OS=Mus musculus OX=10090 GN=Mapk1 PE=1 SV=3	2	2	+	Unt_Treated	1.45998	-0.917703	0.03467528	1.889105142	-3.14517
P62334	PRS10_M	26S proteasome regulatory subunit 10B OS=Mus musculus OX=10090 GN=Psmc6 PE=1 SV=1	4	4	+	Unt_Treated	1.44803	-0.922661	0.03564265	1.895608454	-3.11662
P35564	CALX_MO	Calnexin OS=Mus musculus OX=10090 GN=Canx PE=1 SV=1	4	4	+	Unt_Treated	1.71062	-0.923971	0.01947063	1.897330491	-3.77792
P97449	AMPN_M	Aminopeptidase N OS=Mus musculus OX=10090 GN=Anpep PE=1 SV=4	3	3	+	Unt_Treated	1.30814	-0.932181	0.04918809	1.908158483	-2.79247
Q9CRB8	MTFP1_M	Mitochondrial fission process protein 1 OS=Mus musculus OX=10090 GN=Mtfp1 PE=1 SV=1	2	2	+	Unt_Treated	1.32576	-0.935766	0.0472324	1.912906024	-2.83236
Q3UTJ2	SRBS2_M	Sorbin and SH3 domain-containing protein 2 OS=Mus musculus OX=10090 GN=Sorbs2 PE=1 SV=2	2	2	+	Unt_Treated	1.65366	-0.949986	0.02219934	1.931853911	-3.62799
Q62165	DAG1_M	Dystroglycan OS=Mus musculus OX=10090 GN=Dag1 PE=1 SV=4	3	3	+	Unt_Treated	1.56847	-0.951269	0.02701034	1.933572688	-3.4107
P06151	LDHA_M	L-lactate dehydrogenase A chain OS=Mus musculus OX=10090 GN=Ldha PE=1 SV=3	5	5	+	Unt_Treated	1.81496	-0.953271	0.01531228	1.936257732	-4.06289
P62897	CYC_MO	Cytochrome c somatic OS=Mus musculus OX=10090 GN=Cycs PE=1 SV=2	2	2	+	Unt_Treated	1.50073	-0.956752	0.03156967	1.940935263	-3.24348
P27773	PDIA3_M	Protein disulfide-isomerase A3 OS=Mus musculus OX=10090 GN=Pdia3 PE=1 SV=2	4	4	+	Unt_Treated	1.65934	-0.964516	0.02191089	1.951408747	-3.64278
Q8BU30	SYIC_MO	Isoleucine--tRNA ligase cytoplasmic OS=Mus musculus OX=10090 GN=Iars1 PE=1 SV=2	2	2	+	Unt_Treated	1.47866	-0.968565	0.03321544	1.956893171	-3.19002
Q9JLT4	TRXR2_M	Thioredoxin reductase 2 mitochondrial OS=Mus musculus OX=10090 GN=Txnrd2 PE=1 SV=4	6	5	+	Unt_Treated	1.39281	-0.981322	0.04047529	1.974273687	-2.98656

Q791V5	MTCH2_I	Mitochondrial carrier homolog 2 OS=Mus musculus OX=10090 GN=Mtch2 PE=1 SV=1	5	5	+	Unt_Treated	1.66597	-0.988976	0.02157893	1.984775731	-3.66007
Q99L43	CDS2_M(	Phosphatidate cytidylyltransferase 2 OS=Mus musculus OX=10090 GN=Cds2 PE=1 SV=1	2	2	+	Unt_Treated	1.92882	-0.996348	0.01178094	1.994943655	-4.39018
P47802	MTX1_M	Metaxin-1 OS=Mus musculus OX=10090 GN=Mtx1 PE=1 SV=1	3	3	+	Unt_Treated	1.48237	-1.00348	0.0329329	2.004830128	-3.19898
Q8K310	MATR3_I	Matrin-3 OS=Mus musculus OX=10090 GN=Matr3 PE=1 SV=1	3	3	+	Unt_Treated	1.90516	-1.0144	0.01244056	2.020062598	-4.3207
Q9CY73	RM44_M	39S ribosomal protein L44 mitochondrial OS=Mus musculus OX=10090 GN=Mrpl44 PE=1 SV=3	2	2	+	Unt_Treated	1.54022	-1.04184	0.02882571	2.05885182	-3.34038
Q02257	PLAK_M(	Junction plakoglobin OS=Mus musculus OX=10090 GN=Jup PE=1 SV=3	7	7	+	Unt_Treated	1.94956	-1.0531	0.01123156	2.074983695	-4.45174
P70168	IMB1_M(	Importin subunit beta-1 OS=Mus musculus OX=10090 GN=Kpnb1 PE=1 SV=2	6	6	+	Unt_Treated	2.04849	-1.06613	0.00894355	2.093809227	-4.75414
P62702	RS4X_M(	40S ribosomal protein S4 X isoform OS=Mus musculus OX=10090 GN=Rps4x PE=1 SV=2	2	2	+	Unt_Treated	1.36699	-1.07787	0.04295463	2.110917214	-2.9267
Q9Z1E4	GYS1_M(	Glycogen [starch] synthase muscle OS=Mus musculus OX=10090 GN=Gys1 PE=1 SV=2	8	8	+	Unt_Treated	1.47467	-1.08651	0.03352201	2.123596988	-3.18042
Q76LL6	FHOD3_I	FH1/FH2 domain-containing protein 3 OS=Mus musculus OX=10090 GN=Fhod3 PE=1 SV=1	4	4	+	Unt_Treated	1.70879	-1.11066	0.01955285	2.159444143	-3.77304
Q8K1M6	DNM1L_I	Dynamin-1-like protein OS=Mus musculus OX=10090 GN=Dnm1l PE=1 SV=2	12	12	+	Unt_Treated	2.30751	-1.11533	0.00492595	2.166445584	-5.62069
Q3TZZ7	ESYT2_M	Extended synaptotagmin-2 OS=Mus musculus OX=10090 GN=Esyt2 PE=1 SV=1	2	2	+	Unt_Treated	2.20144	-1.13832	0.00628869	2.201245416	-5.252
Q64310	SURF4_N	Surfeit locus protein 4 OS=Mus musculus OX=10090 GN=Surf4 PE=1 SV=1	2	2	+	Unt_Treated	1.55364	-1.1413	0.0279486	2.205796961	-3.37368
Q61171	PRDX2_I	Peroxiredoxin-2 OS=Mus musculus OX=10090 GN=Prdx2 PE=1 SV=3	5	5	+	Unt_Treated	2.02449	-1.15458	0.0094517	2.226195048	-4.67944
P70333	HNRH2_I	Heterogeneous nuclear ribonucleoprotein H2 OS=Mus musculus OX=10090 GN=Hnrnph2 PE=1 SV=1	3	1	+	Unt_Treated	2.16	-1.16456	0.00691831	2.241648383	-5.1133
Q8R010	AIMP2_I	Aminoacyl tRNA synthase complex-interacting multifunctional protein 2 OS=Mus musculus OX=10090 GN=Aimp2 PE=1 SV=2	3	3	+	Unt_Treated	1.41534	-1.17815	0.03842908	2.262864188	-3.03928
Q9CWD8	NUBPL_I	Iron-sulfur protein NUBPL OS=Mus musculus OX=10090 GN=Nubpl PE=1 SV=2	2	2	+	Unt_Treated	2.0087	-1.18022	0.00980167	2.266113309	-4.63075
P62259	1433E_I	14-3-3 protein epsilon OS=Mus musculus OX=10090 GN=Ywhae PE=1 SV=1	5	5	+	Unt_Treated	2.36862	-1.18959	0.00427937	2.280879136	-5.84245
Q91YM4	FAKD4_I	FAST kinase domain-containing protein 4 OS=Mus musculus OX=10090 GN=Tbrg4 PE=1 SV=1	2	2	+	Unt_Treated	2.08864	-1.1933	0.0081538	2.286752138	-4.88117
P38060	HMGCL_	Hydroxymethylglutaryl-CoA lyase mitochondrial OS=Mus musculus OX=10090 GN=Hmgcl PE=1 SV=2	3	3	+	Unt_Treated	1.81956	-1.20072	0.01515095	2.298543549	-4.07576
Q9R0Y5	KAD1_M(	Adenylate kinase isoenzyme 1 OS=Mus musculus OX=10090 GN=Ak1 PE=1 SV=1	5	5	+	Unt_Treated	1.33488	-1.20083	0.04625088	2.298718811	-2.85309
Q8CI94	PYGB_M(	Glycogen phosphorylase brain form OS=Mus musculus OX=10090 GN=Pygb PE=1 SV=3	18	14	+	Unt_Treated	2.37018	-1.22656	0.00426403	2.340083484	-5.8482
P51667	MLRV_M	Myosin regulatory light chain 2 ventricular/cardiac muscle isoform OS=Mus musculus OX=10090 GN=Myl2 PE=1 SV=3	8	8	+	Unt_Treated	1.60839	-1.2369	0.02463826	2.356915441	-3.51152
Q9CZW5	TOM70_I	Mitochondrial import receptor subunit TOM70 OS=Mus musculus OX=10090 GN=Tomm70 PE=1 SV=2	5	5	+	Unt_Treated	2.00508	-1.2413	0.00988371	2.364114647	-4.61964
Q59J78	NDUF2_I	NADH dehydrogenase [ubiquinone] 1 alpha subcomplex assembly factor 2 OS=Mus musculus OX=10090 GN=Ndufaf2 PE=1 SV=1	2	2	+	Unt_Treated	1.64751	-1.26449	0.02251594	2.40242268	-3.61202

P80316	TCPE_MO	T-complex protein 1 subunit epsilon OS=Mus musculus OX=10090 GN=Cct5 PE=1 SV=1	4	4	+	Unt_Treated	1.66613	-1.28913	0.02157099	2.443806403	-3.66049
P98192	GNPAT_M	Dihydroxyacetone phosphate acyltransferase OS=Mus musculus OX=10090 GN=Gnpat PE=1 SV=1	5	5	+	Unt_Treated	3.34116	-1.31136	0.00045587	2.481753798	-10.5546
P17751	TPIS_MO	Triosephosphate isomerase OS=Mus musculus OX=10090 GN=Tpi1 PE=1 SV=4	8	8	+	Unt_Treated	2.25198	-1.31735	0.00559783	2.49207934	-5.42515
P61211	ARL1_MO	ADP-ribosylation factor-like protein 1 OS=Mus musculus OX=10090 GN=Arl1 PE=1 SV=1	2	2	+	Unt_Treated	1.42052	-1.32873	0.03797345	2.511814633	-3.05147
Q91WD5	NDUS2_M	NADH dehydrogenase [ubiquinone] iron-sulfur protein 2 mitochondrial OS=Mus musculus OX=10090 GN=Ndufs2 PE=1 SV=1	6	6	+	Unt_Treated	1.3957	-1.34113	0.04020685	2.533496788	-2.9933
Q99JY3	GIMA4_M	GTPase IMAP family member 4 OS=Mus musculus OX=10090 GN=Gimap4 PE=1 SV=2	2	2	+	Unt_Treated	1.57258	-1.36364	0.02675593	2.573336282	-3.42098
Q61425	HCDH_M	Hydroxyacyl-coenzyme A dehydrogenase mitochondrial OS=Mus musculus OX=10090 GN=Hadh PE=1 SV=2	5	5	+	Unt_Treated	1.62343	-1.3725	0.02379962	2.589188498	-3.54996
P82198	BGH3_M	Transforming growth factor-beta-induced protein ig-h3 OS=Mus musculus OX=10090 GN=Tgfbi PE=1 SV=1	3	3	+	Unt_Treated	1.42974	-1.37338	0.03717577	2.590768306	-3.07323
Q9Z2Z6	MCAT_M	Mitochondrial carnitine/acylcarnitine carrier protein OS=Mus musculus OX=10090 GN=Slc25a20 PE=1 SV=1	2	2	+	Unt_Treated	1.53844	-1.38817	0.0289441	2.617464554	-3.33596
P21550	ENOB_M	Beta-enolase OS=Mus musculus OX=10090 GN=Eno3 PE=1 SV=3	9	5	+	Unt_Treated	1.75261	-1.40937	0.01767624	2.656211453	-3.89095
P62821	RAB1A_M	Ras-related protein Rab-1A OS=Mus musculus OX=10090 GN=Rab1A PE=1 SV=3	2	2	+	Unt_Treated	1.87107	-1.51367	0.01345643	2.855354755	-4.22196
Q9CQ54	NDUC2_I	NADH dehydrogenase [ubiquinone] 1 subunit C2 OS=Mus musculus OX=10090 GN=Ndufc2 PE=1 SV=1	2	2	+	Unt_Treated	1.92514	-1.52179	0.01188119	2.871471017	-4.37933
Q8VE38	OXND1_I	Oxidoreductase NAD-binding domain-containing protein 1 OS=Mus musculus OX=10090 GN=Oxnad1 PE=1 SV=2	2	2	+	Unt_Treated	1.99151	-1.56732	0.01019741	2.963536857	-4.57818
Q8QZS1	HIBCH_M	3-hydroxyisobutyryl-CoA hydrolase mitochondrial OS=Mus musculus OX=10090 GN=Hibch PE=1 SV=1	3	3	+	Unt_Treated	1.61227	-1.5788	0.02441912	2.98721277	-3.52141
O89020	AFAM_M	Afamin OS=Mus musculus OX=10090 GN=Afm PE=1 SV=2	4	4	+	Unt_Treated	1.61109	-1.57911	0.02448556	2.987854718	-3.51839
Q8BG95	MYPT2_M	Protein phosphatase 1 regulatory subunit 12B OS=Mus musculus OX=10090 GN=Ppp1r12b PE=1 SV=2	4	3	+	Unt_Treated	1.45481	-1.617	0.03509054	3.067365319	-3.1328
Q9R0Q6	ARC1A_M	Actin-related protein 2/3 complex subunit 1A OS=Mus musculus OX=10090 GN=Arpc1a PE=1 SV=1	3	3	+	Unt_Treated	1.38159	-1.62469	0.0415346	3.083758955	-2.96048
P12787	COX5A_M	Cytochrome c oxidase subunit 5A mitochondrial OS=Mus musculus OX=10090 GN=Cox5a PE=1 SV=2	4	4	+	Unt_Treated	1.40199	-1.62997	0.03962872	3.095065626	-3.00799
G5E8K5	ANK3_M	Ankyrin-3 OS=Mus musculus OX=10090 GN=Ank3 PE=1 SV=1	3	1	+	Unt_Treated	1.8811	-1.67078	0.01314922	3.183866843	-4.25085
Q62448	IF4G2_M	Eukaryotic translation initiation factor 4 gamma 2 OS=Mus musculus OX=10090 GN=Eif4g2 PE=1 SV=2	2	2	+	Unt_Treated	1.64417	-1.69847	0.02268977	3.245565788	-3.60338
Q9D7A8	ARMC1_I	Armadillo repeat-containing protein 1 OS=Mus musculus OX=10090 GN=Armc1 PE=1 SV=1	3	3	+	Unt_Treated	2.48861	-1.80602	0.00324631	3.496762957	-6.29905
Q9D1D4	TMEDA_I	Transmembrane emp24 domain-containing protein 10 OS=Mus musculus OX=10090 GN=Tmed10 PE=1 SV=1	2	2	+	Unt_Treated	2.35302	-1.84274	0.00443588	3.586906154	-5.78516
Q9JI39	ABCBA_M	ATP-binding cassette sub-family B member 10 mitochondrial OS=Mus musculus OX=10090 GN=Abcb10 PE=1 SV=1	4	4	+	Unt_Treated	2.06518	-1.94013	0.00860637	3.837402247	-4.80664

P10518	HEM2_M	Delta-aminolevulinic acid dehydratase OS=Mus musculus OX=10090 GN=Alad PE=1 SV=1	3	3	+	Unt_Treated	1.79795	-1.98887	0.01592392	3.969259816	-4.01548
Q3V1L4	5NTC_M	Cytosolic purine 5'-nucleotidase OS=Mus musculus OX=10090 GN=Nt5c2 PE=1 SV=2	2	2	+	Unt_Treated	1.39003	-2.10105	0.04073521	4.290215152	-2.98009
Q3V3R4	ITA1_MC	Integrin alpha-1 OS=Mus musculus OX=10090 GN=Itga1 PE=1 SV=2	3	3	+	Unt_Treated	1.39411	-2.16157	0.04035432	4.474014711	-2.98958
P29758	OAT_MO	Ornithine aminotransferase mitochondrial OS=Mus musculus OX=10090 GN=Oat PE=1 SV=1	7	7	+	Unt_Treated	2.61484	-2.37832	0.0024275	5.199309366	-6.81175
O35344	IMA4_M	Importin subunit alpha-4 OS=Mus musculus OX=10090 GN=Kpna3 PE=1 SV=1	3	3	+	Unt_Treated	1.3881	-2.68357	0.04091664	6.424436864	-2.97559
Q62000	MIME_M	Mimecan OS=Mus musculus OX=10090 GN=Ogn PE=1 SV=1	2	2	+	Unt_Treated	2.85092	-4.58988	0.00140955	24.0819448	-7.86909

Table S5. Relative abundance of proteins in murine heart and lung. Total protein from heart or lung homogenates was analysed by mass spectrometry. The values are relative abundance; A - controls, B and C - mice treated with 2DG. The data in Table S1 are derived from this complete list.

		Heart						Lung					
Accession	Description	A4	A6	B21	B20	C34	C35	A1	A2	B17	B19	C33	C34
Q3TCH7	CUL4A_MOUSE	0.00115	0.00088	0.00076	0.00052	0.00058	0.00134	0.02897	0.01662	0.02827	0.03124	0.03320	0.02640
A2A5R2	BIG2_MOUSE	0.00000	0.00000	0.00000	0.00000	0.00000	0.00000	0.00484	0.00281	0.00310	0.00295	0.00655	0.00539
A2A8L5	PTPRF_MOUSE	0.00000	0.00000	0.00000	0.00000	0.00000	0.00000	0.00000	0.00045	0.00046	0.00031	0.00000	0.00000
A2A8Z1	OSBL9_MOUSE	0.00000	0.00000	0.00000	0.00000	0.00000	0.00000	0.00564	0.00848	0.00673	0.00757	0.00880	0.00819
A2AAJ9	OBSCN_MOUSE	0.13668	0.17473	0.13010	0.15088	0.16738	0.15547	0.00051	0.00098	0.00000	0.00407	0.00190	0.00166
A2ABU4	MYOM3_MOUSE	0.00000	0.00000	0.00000	0.00000	0.00000	0.00000	0.01859	0.00000	0.00147	0.00000	0.00261	0.00321
A2ADY9	DDI2_MOUSE	0.00000	0.00000	0.00000	0.00000	0.00000	0.00000	0.00772	0.01003	0.01647	0.01220	0.01982	0.01610
A2AGT5	CKAP5_MOUSE	0.00000	0.00000	0.00000	0.00000	0.00000	0.00000	0.04235	0.09712	0.07070	0.07052	0.06654	0.03975
A2AJI0	MA7D1_MOUSE	0.00000	0.00000	0.00000	0.00000	0.00000	0.00000	0.00354	0.00755	0.00328	0.00176	0.00271	0.00574
A2AJL3	FGGY_MOUSE	0.00000	0.00000	0.00000	0.00000	0.00000	0.00000	0.00000	0.00000	0.00024	0.00016	0.00000	0.00000
A2AKK5	ACNT1_MOUSE	0.00000	0.00000	0.00000	0.00000	0.00000	0.00000	0.00000	0.00000	0.00818	0.00000	0.00000	0.01196
A2AMM0	CAVN4_MOUSE	0.00669	0.01315	0.00772	0.00713	0.01356	0.00567	0.00612	0.00000	0.00199	0.00271	0.00361	0.00092
A2AN08	UBR4_MOUSE	0.00610	0.00825	0.00275	0.00274	0.00207	0.00276	0.01099	0.00629	0.01190	0.00921	0.01131	0.00759
A2APV2	FMNL2_MOUSE	0.00000	0.00000	0.00000	0.00000	0.00000	0.00000	0.01326	0.01099	0.01069	0.00970	0.00911	0.01075
A2AQ25	SKT_MOUSE	0.00000	0.00000	0.00000	0.00000	0.00000	0.00000	0.00000	0.00176	0.00160	0.00126	0.00315	0.00085
A2ARA8	ITA8_MOUSE	0.00000	0.00000	0.00000	0.00000	0.00000	0.00000	0.01668	0.03794	0.04194	0.03432	0.04470	0.04153
A2ARV4	LRP2_MOUSE	0.00000	0.00000	0.00000	0.00000	0.00000	0.00000	0.00030	0.00002	0.00047	0.00007	0.00031	0.00015
A2ASQ1	AGRIN_MOUSE	0.00889	0.00884	0.00763	0.00920	0.00675	0.00567	0.02745	0.01960	0.02738	0.01835	0.03019	0.03063
A2ASS6	TITIN_MOUSE	6.96078	9.42445	7.41544	7.76892	7.41862	7.00918	0.23722	0.03060	0.02915	0.04019	0.02329	0.02295
A2ATU0	DHTK1_MOUSE	0.00000	0.00000	0.00000	0.00000	0.00000	0.00000	0.00000	0.00000	0.00000	0.00000	0.00000	0.00000
A2AUC9	KLH41_MOUSE	0.00000	0.00000	0.00000	0.00000	0.00000	0.00000	0.01005	0.00000	0.00000	0.00000	0.00000	0.00000
A2AVZ9	S43A3_MOUSE	0.00000	0.00000	0.00000	0.00000	0.00000	0.00000	0.01555	0.01817	0.01316	0.01458	0.01256	0.01840
A2AWA9	RBGP1_MOUSE	0.00000	0.00000	0.00000	0.00000	0.00000	0.00000	0.00394	0.00580	0.00555	0.00625	0.00513	0.00488
P11588	MUP1_MOUSE	0.00000	0.00000	0.00000	0.00000	0.00000	0.00000	0.00000	0.00000	0.00000	0.00000	0.00000	0.00000
A3KGV1	ODFP2_MOUSE	0.00000	0.00000	0.00000	0.00000	0.00000	0.00000	0.00000	0.00000	0.00199	0.00000	0.00000	0.00000
A3KMP2	TTC38_MOUSE	0.00884	0.00843	0.00940	0.00332	0.02627	0.00799	0.01135	0.01560	0.01872	0.01608	0.01896	0.01773
A6H6E2	MMRN2_MOUSE	0.00189	0.00089	0.00150	0.00103	0.00189	0.00245	0.00492	0.00461	0.00956	0.00372	0.00924	0.00526
A6PWY4	WDR76_MOUSE	0.00000	0.00000	0.00000	0.00000	0.00000	0.00000	0.03020	0.02352	0.01219	0.02059	0.01791	0.01224
A6X8Z5	RHG31_MOUSE	0.00000	0.00000	0.00000	0.00000	0.00000	0.00000	0.00056	0.00540	0.00910	0.00478	0.00429	0.00587
A6X935	ITIH4_MOUSE	0.14253	0.16484	0.09039	0.13209	0.09136	0.18981	0.01045	0.00776	0.00814	0.01091	0.01053	0.00523
B1AVD1	XPP2_MOUSE	0.00000	0.00000	0.00000	0.00000	0.00000	0.00000	0.00187	0.00464	0.00281	0.00188	0.00000	0.00356
B1AY13	UBP24_MOUSE	0.00000	0.00000	0.00000	0.00000	0.00000	0.00000	0.01524	0.01277	0.02213	0.01499	0.01852	0.01694

B2RQC6	PYR1_MOUSE	0.00000	0.00000	0.00000	0.00000	0.00000	0.00000	0.00451	0.00550	0.00403	0.00424	0.00411	0.00392
B2RSH2	GNAI1_MOUSE	0.00000	0.00000	0.00000	0.00000	0.00000	0.00000	0.00904	0.00821	0.00628	0.00680	0.00783	0.00933
B2RUR8	OTU7B_MOUSE	0.00000	0.00000	0.00000	0.00000	0.00000	0.00000	0.00067	0.00303	0.00215	0.00178	0.00000	0.00081
B2RX12	MRP3_MOUSE	0.00000	0.00000	0.00000	0.00000	0.00000	0.00000	0.00773	0.00460	0.00598	0.00598	0.00630	0.00707
B2RXC1	TPC11_MOUSE	0.00000	0.00000	0.00000	0.00000	0.00000	0.00000	0.00000	0.00000	0.00000	0.00000	0.00000	0.00000
B2RXR6	ANR44_MOUSE	0.00000	0.00000	0.00000	0.00000	0.00000	0.00000	0.00913	0.00949	0.00605	0.00845	0.00372	0.00889
B2RXS4	PLXB2_MOUSE	0.00179	0.00150	0.00080	0.00089	0.00092	0.00091	0.00552	0.00622	0.01192	0.00800	0.00872	0.00774
B2RY56	RBM25_MOUSE	0.00000	0.00000	0.00000	0.00000	0.00000	0.00000	0.00064	0.00344	0.00695	0.00608	0.00207	0.00182
B8JK39	ITA9_MOUSE	0.00000	0.00000	0.00000	0.00000	0.00000	0.00000	0.00000	0.00000	0.00000	0.00000	0.00000	0.00000
B9EJ86	OSBL8_MOUSE	0.00000	0.00000	0.00000	0.00000	0.00000	0.00000	0.01506	0.01443	0.01868	0.00994	0.01707	0.01743
C0HKG6	RNT2B_MOUSE	0.00000	0.00000	0.00000	0.00000	0.00000	0.00000	0.00000	0.00037	0.00073	0.00000	0.00060	0.00075
D3YVF0	AKAP5_MOUSE	0.00000	0.00000	0.00000	0.00000	0.00000	0.00000	0.04282	0.06631	0.06092	0.05954	0.08001	0.05142
D3YXK2	SAFB1_MOUSE	0.00000	0.00000	0.00000	0.00000	0.00000	0.00000	0.00380	0.00533	0.00442	0.00530	0.00662	0.00689
D3YZP9	CCDC6_MOUSE	0.00000	0.00000	0.00000	0.00000	0.00000	0.00000	0.00094	0.00497	0.00065	0.00336	0.00034	0.00195
D3Z6Q9	BIN2_MOUSE	0.00000	0.00000	0.00000	0.00000	0.00000	0.00000	0.01147	0.00567	0.01869	0.02052	0.01230	0.01476
D3Z7P3	GLSK_MOUSE	0.01341	0.01334	0.00887	0.00898	0.00913	0.00858	0.00516	0.00776	0.01116	0.00788	0.00535	0.00849
Q9JIY8	NT8F3_MOUSE	0.00000	0.00000	0.00000	0.00000	0.00000	0.00000	0.00000	0.00000	0.00000	0.00000	0.00000	0.00000
E9PV24	FIBA_MOUSE	0.00734	0.00170	0.00168	0.00696	0.00217	0.00075	0.08094	0.02839	0.04144	0.05326	0.07907	0.01924
E9PVA8	GCN1_MOUSE	0.00000	0.00000	0.00000	0.00000	0.00000	0.00000	0.01297	0.00507	0.01050	0.00920	0.01271	0.01043
E9PYK3	PARP4_MOUSE	0.00000	0.00000	0.00000	0.00000	0.00000	0.00000	0.00072	0.00189	0.00053	0.00198	0.00102	0.00189
E9Q1P8	I2BP2_MOUSE	0.00000	0.00000	0.00000	0.00000	0.00000	0.00000	0.00100	0.00184	0.00578	0.00363	0.00187	0.00398
E9Q394	AKP13_MOUSE	0.00000	0.00000	0.00000	0.00000	0.00000	0.00000	0.00736	0.00447	0.00843	0.00838	0.00356	0.00836
E9Q3S4	M3K19_MOUSE	0.00000	0.00000	0.00000	0.00000	0.00000	0.00000	0.00000	0.00000	0.00000	0.00107	0.00000	0.00000
E9Q401	RYR2_MOUSE	0.23512	0.26786	0.21991	0.22102	0.23265	0.22091	0.00000	0.00000	0.00000	0.00021	0.00000	0.00000
E9Q557	DESP_MOUSE	0.13817	0.14500	0.23015	0.15905	0.22304	0.12961	0.03870	0.00858	0.01416	0.01420	0.02064	0.01636
E9Q5C9	NOLC1_MOUSE	0.00000	0.00000	0.00000	0.00000	0.00000	0.00000	0.00000	0.00146	0.00241	0.00083	0.00077	0.00093
E9Q634	MYO1E_MOUSE	0.00000	0.00000	0.00000	0.00000	0.00000	0.00000	0.01976	0.01390	0.01101	0.00446	0.00000	0.00000
E9Q6P5	TTC7B_MOUSE	0.00000	0.00000	0.00000	0.00000	0.00000	0.00000	0.00317	0.00439	0.00439	0.00282	0.00324	0.00442
E9Q735	UBE4A_MOUSE	0.00000	0.00000	0.00000	0.00000	0.00000	0.00000	0.00000	0.00115	0.00000	0.00000	0.00000	0.00000
E9Q7G0	NUMA1_MOUSE	0.00000	0.00000	0.00000	0.00000	0.00000	0.00000	0.03600	0.02977	0.04384	0.03729	0.03921	0.04444
E9QAT4	SC16A_MOUSE	0.00000	0.00000	0.00000	0.00000	0.00000	0.00000	0.00126	0.00167	0.00201	0.00195	0.00197	0.00266
F6ZDS4	TPR_MOUSE	0.00039	0.00116	0.00121	0.00067	0.00098	0.00129	0.00475	0.00651	0.00761	0.00606	0.00650	0.00776
F7BWT7	TSN15_MOUSE	0.00000	0.00000	0.00000	0.00000	0.00000	0.00000	0.00330	0.00692	0.00572	0.00566	0.00440	0.00484
F8VPU2	FARP1_MOUSE	0.00000	0.00000	0.00000	0.00000	0.00000	0.00000	0.01307	0.01297	0.01822	0.01243	0.01144	0.01093

G3X982	AOXC_MOUSE	0.00000	0.00000	0.00000	0.00000	0.00000	0.00000	0.00943	0.03049	0.00789	0.00760	0.01126	0.01477
G3X9C2	FBX50_MOUSE	0.00000	0.00000	0.00000	0.00000	0.00000	0.00000	0.00093	0.00000	0.00000	0.00000	0.00234	0.00135
G3X9K3	BIG1_MOUSE	0.00000	0.00000	0.00000	0.00000	0.00000	0.00000	0.00000	0.00166	0.00000	0.00000	0.00000	0.00000
G5E829	AT2B1_MOUSE	0.00000	0.00000	0.00000	0.00000	0.00000	0.00000	0.03086	0.03305	0.04460	0.03630	0.04142	0.03777
G5E897	PLGT3_MOUSE	0.00000	0.00000	0.00000	0.00000	0.00000	0.00000	0.00502	0.00866	0.01041	0.00530	0.00814	0.01120
G5E8K5	ANK3_MOUSE	0.00032	0.00037	0.00103	0.00056	0.00099	0.00068	0.00483	0.00417	0.00606	0.00535	0.00562	0.00210
G5E8Q8	AGRF5_MOUSE	0.00000	0.00000	0.00000	0.00000	0.00000	0.00000	0.00000	0.00366	0.00423	0.00253	0.00216	0.00360
O08528	HXK2_MOUSE	0.07370	0.04871	0.04136	0.05364	0.04806	0.04085	0.02347	0.00581	0.00557	0.00672	0.00838	0.00918
O08529	CAN2_MOUSE	0.00575	0.00767	0.00532	0.00707	0.00835	0.00598	0.03279	0.04555	0.04955	0.04542	0.04438	0.04610
O08530	S1PR1_MOUSE	0.00000	0.00000	0.00000	0.00000	0.00000	0.00000	0.01198	0.00906	0.00308	0.00397	0.00128	0.00382
O08532	CA2D1_MOUSE	0.01905	0.02513	0.01957	0.01347	0.01801	0.02337	0.00862	0.01097	0.00641	0.01213	0.00317	0.00821
O08539	BIN1_MOUSE	0.00000	0.00000	0.00000	0.00000	0.00000	0.00000	0.03009	0.01028	0.01572	0.00862	0.01430	0.01357
O08547	SC22B_MOUSE	0.00000	0.00000	0.00000	0.00000	0.00000	0.00000	0.02236	0.01613	0.02184	0.02103	0.02329	0.01990
O08553	DPYL2_MOUSE	0.04573	0.04906	0.03826	0.03237	0.05196	0.05024	0.37317	0.42833	0.38840	0.42375	0.40060	0.46678
O08573	LEG9_MOUSE	0.00000	0.00000	0.00000	0.00000	0.00000	0.00000	0.01324	0.00765	0.00978	0.01138	0.00617	0.01625
O08579	EMD_MOUSE	0.00000	0.00000	0.00000	0.00000	0.00000	0.00000	0.01001	0.00943	0.00975	0.00969	0.00903	0.01000
O08582	GTPB1_MOUSE	0.00000	0.00000	0.00000	0.00000	0.00000	0.00000	0.00383	0.00328	0.00446	0.00424	0.00413	0.00611
O08583	THOC4_MOUSE	0.00000	0.00000	0.00000	0.00000	0.00000	0.00000	0.02296	0.02147	0.01762	0.01861	0.01921	0.02194
O08585	CLCA_MOUSE	0.00000	0.00000	0.00000	0.00000	0.00000	0.00000	0.00000	0.01247	0.01808	0.01871	0.00878	0.00716
O08599	STXB1_MOUSE	0.00000	0.00000	0.00000	0.00000	0.00000	0.00000	0.01132	0.01520	0.01052	0.00891	0.01063	0.01343
O08600	NUCG_MOUSE	0.00485	0.00293	0.00347	0.00315	0.00258	0.00324	0.00000	0.00092	0.00000	0.00089	0.00000	0.00225
O08638	MYH11_MOUSE	0.10092	0.11043	0.07022	0.07083	0.07979	0.10797	0.15945	0.12973	0.10320	0.11913	0.12469	0.11664
O08663	MAP2_MOUSE	0.00465	0.00609	0.00296	0.00129	0.00396	0.00514	0.07911	0.03639	0.06187	0.06650	0.09240	0.08536
O08677	KNG1_MOUSE	0.00011	0.00000	0.00068	0.00297	0.00000	0.00000	0.03469	0.02362	0.02858	0.04418	0.04126	0.01855
O08692	NGP_MOUSE	0.00000	0.00000	0.00000	0.00000	0.00000	0.00000	0.06845	0.06355	0.03615	0.05291	0.07734	0.10226
O08709	PRDX6_MOUSE	0.01551	0.01542	0.03326	0.02340	0.02985	0.01043	1.22585	1.14145	1.02518	0.99616	1.16888	1.12801
O08738	CASP6_MOUSE	0.00000	0.00000	0.00000	0.00000	0.00000	0.00000	0.00644	0.00610	0.00854	0.00626	0.00554	0.00636
O08739	AMPD3_MOUSE	0.00000	0.00000	0.00000	0.00000	0.00000	0.00000	0.00000	0.00245	0.00191	0.00094	0.00219	0.00207
O08749	DLDH_MOUSE	0.77387	0.55528	0.71460	0.47179	0.70298	0.43546	0.04053	0.03729	0.04053	0.03132	0.04180	0.03876
O08756	HCD2_MOUSE	0.10052	0.06572	0.10198	0.06212	0.08187	0.06449	0.00879	0.02408	0.03821	0.02461	0.02093	0.02278
O08759	UBE3A_MOUSE	0.00334	0.00325	0.00217	0.00174	0.00225	0.00274	0.00466	0.00351	0.00320	0.00403	0.00183	0.00563
O08784	TCOF_MOUSE	0.00000	0.00000	0.00000	0.00000	0.00000	0.00000	0.00258	0.00190	0.00448	0.00270	0.00481	0.00239
O08788	DCTN1_MOUSE	0.00880	0.01902	0.01350	0.01072	0.00927	0.01401	0.01718	0.03388	0.04459	0.04118	0.03264	0.03745
O08795	GLU2B_MOUSE	0.00000	0.00000	0.00000	0.00000	0.00000	0.00000	0.01763	0.02734	0.03484	0.02985	0.03253	0.02985

O08800	SPB8_MOUSE	0.00000	0.00000	0.00000	0.00000	0.00000	0.00000	0.00000	0.00320	0.00621	0.00000	0.00459	0.00515
O08807	PRDX4_MOUSE	0.00000	0.00000	0.00000	0.00000	0.00000	0.00000	0.05053	0.04342	0.07067	0.05914	0.05811	0.05066
O08808	DIAP1_MOUSE	0.00000	0.00000	0.00000	0.00000	0.00000	0.00000	0.00691	0.00658	0.00891	0.00756	0.00542	0.00616
O08810	U5S1_MOUSE	0.00000	0.00000	0.00000	0.00000	0.00000	0.00000	0.01376	0.01507	0.01804	0.01532	0.01439	0.01929
O08900	IKZF3_MOUSE	0.00000	0.00000	0.00000	0.00000	0.00000	0.00000	0.00000	0.00000	0.00085	0.00000	0.00000	0.00153
O08911	MK12_MOUSE	0.00000	0.00000	0.00000	0.00000	0.00000	0.00000	0.00175	0.00124	0.00409	0.00222	0.00420	0.00296
O08914	FAAH1_MOUSE	0.00000	0.00000	0.00000	0.00000	0.00000	0.00000	0.00095	0.00640	0.00630	0.00340	0.00454	0.00329
O08915	AIP_MOUSE	0.00000	0.00000	0.00000	0.00000	0.00000	0.00000	0.00464	0.00419	0.00771	0.00447	0.00689	0.00472
O08917	FLOT1_MOUSE	0.00190	0.00593	0.01720	0.00609	0.02566	0.00810	0.01992	0.01971	0.02667	0.02201	0.02564	0.02476
O08966	S22A1_MOUSE	0.00000	0.00000	0.00000	0.00000	0.00000	0.00000	0.00129	0.01745	0.00413	0.01282	0.00333	0.00799
O08967	CYH3_MOUSE	0.00000	0.00000	0.00000	0.00000	0.00000	0.00000	0.00492	0.00638	0.00577	0.00599	0.00525	0.00615
O08992	SDCB1_MOUSE	0.00000	0.00000	0.00000	0.00000	0.00000	0.00000	0.00532	0.00812	0.00841	0.00621	0.01102	0.00977
O08997	ATOX1_MOUSE	0.00000	0.00000	0.00000	0.00000	0.00000	0.00000	0.01035	0.00427	0.00471	0.00413	0.00404	0.00402
O09005	DEGS1_MOUSE	0.00000	0.00000	0.00000	0.00000	0.00000	0.00000	0.00474	0.00402	0.00306	0.00000	0.00467	0.00581
O09043	NAPSA_MOUSE	0.00000	0.00000	0.00000	0.00000	0.00000	0.00000	0.00000	0.01786	0.00392	0.00427	0.00080	0.00211
O09044	SNP23_MOUSE	0.00111	0.00089	0.00047	0.00049	0.00041	0.00029	0.01434	0.02049	0.02288	0.01961	0.02333	0.02457
O09061	PSB1_MOUSE	0.00609	0.00735	0.00683	0.00587	0.00752	0.00253	0.01799	0.01438	0.02886	0.02135	0.01534	0.02236
O09106	HDAC1_MOUSE	0.00000	0.00000	0.00000	0.00000	0.00000	0.00000	0.00991	0.01327	0.01100	0.01156	0.00638	0.01336
O09110	MP2K3_MOUSE	0.00000	0.00000	0.00000	0.00000	0.00000	0.00000	0.00000	0.00000	0.00079	0.00000	0.00000	0.00000
O09111	NDUBB_MOUSE	0.00000	0.00000	0.00000	0.00000	0.00000	0.00000	0.00280	0.00569	0.00128	0.00471	0.00078	0.00186
O09117	SYPL1_MOUSE	0.00000	0.00000	0.00000	0.00000	0.00000	0.00000	0.00000	0.02038	0.03396	0.02178	0.01684	0.01193
O09131	GSTO1_MOUSE	0.00746	0.00244	0.00789	0.00817	0.00670	0.00527	0.04306	0.06058	0.08070	0.06126	0.04261	0.06757
O09159	MA2B1_MOUSE	0.00000	0.00000	0.00000	0.00000	0.00000	0.00000	0.01193	0.00465	0.00549	0.00486	0.00681	0.00473
O09161	CASQ2_MOUSE	0.09549	0.08736	0.11971	0.08586	0.14736	0.13954	0.03929	0.01654	0.02129	0.02452	0.02862	0.01742
O09164	SODE_MOUSE	0.00000	0.00000	0.00000	0.00000	0.00000	0.00000	0.11280	0.08435	0.08443	0.07034	0.08041	0.07238
O09165	CASQ1_MOUSE	0.00000	0.00000	0.00000	0.00000	0.00000	0.00000	0.06731	0.00000	0.00008	0.00005	0.00000	0.00003
O09167	RL21_MOUSE	0.00000	0.00000	0.00000	0.00000	0.00000	0.00000	0.01676	0.01502	0.01759	0.01381	0.01667	0.01391
O09172	GSH0_MOUSE	0.00443	0.00748	0.00507	0.00304	0.00306	0.00261	0.00952	0.00511	0.00485	0.00623	0.00713	0.00325
O09173	HGD_MOUSE	0.00000	0.00000	0.00000	0.00000	0.00000	0.00000	0.00000	0.00000	0.00000	0.00000	0.00162	0.00000
O09174	AMACR_MOUSE	0.01291	0.01822	0.01312	0.01443	0.00946	0.01005	0.00000	0.00018	0.00006	0.00004	0.00007	0.00011
O35074	PTGIS_MOUSE	0.00000	0.00000	0.00000	0.00000	0.00000	0.00000	0.05379	0.04311	0.04112	0.04430	0.05626	0.04836
O35075	VP26C_MOUSE	0.00000	0.00000	0.00000	0.00000	0.00000	0.00000	0.00000	0.00000	0.02325	0.00000	0.00000	0.03618
O35083	PLCA_MOUSE	0.00000	0.00000	0.00000	0.00000	0.00000	0.00000	0.00000	0.00391	0.00000	0.00084	0.00297	0.00189
O35098	DPYL4_MOUSE	0.00000	0.00000	0.00000	0.00000	0.00000	0.00000	0.00607	0.00800	0.00926	0.00733	0.00640	0.00000

O35114	SCRB2_MOUSE	0.00000	0.00000	0.00000	0.00000	0.00000	0.00000	0.00686	0.01404	0.01990	0.00946	0.00597	0.00473
O35129	PHB2_MOUSE	0.00395	0.00178	0.00333	0.00374	0.00121	0.00575	0.04986	0.04028	0.03487	0.03669	0.04975	0.04528
O35206	COFA1_MOUSE	0.01260	0.01063	0.01266	0.01123	0.01385	0.01568	0.00566	0.00024	0.00127	0.00000	0.00171	0.00051
O35215	DOPD_MOUSE	0.00232	0.00065	0.00186	0.00363	0.00268	0.00111	0.05390	0.03215	0.03357	0.02949	0.03076	0.03385
O35226	PSMD4_MOUSE	0.00957	0.01392	0.00677	0.00728	0.00839	0.00792	0.01186	0.00951	0.01336	0.01179	0.01655	0.01234
O35250	EXOC7_MOUSE	0.00000	0.00000	0.00000	0.00000	0.00000	0.00000	0.00000	0.00304	0.00633	0.00533	0.00342	0.00416
O35286	DHX15_MOUSE	0.00000	0.00000	0.00000	0.00000	0.00000	0.00000	0.00546	0.00577	0.00594	0.00678	0.00883	0.00595
O35295	PURB_MOUSE	0.00000	0.00000	0.00000	0.00000	0.00000	0.00000	0.00590	0.00794	0.02051	0.01174	0.01591	0.01709
O35309	NMI_MOUSE	0.00000	0.00000	0.00000	0.00000	0.00000	0.00000	0.00181	0.00733	0.00839	0.00740	0.00231	0.00920
O35326	SRSF5_MOUSE	0.00000	0.00000	0.00000	0.00000	0.00000	0.00000	0.00569	0.01090	0.00795	0.00823	0.00683	0.01037
O35343	IMA3_MOUSE	0.00206	0.00064	0.00257	0.00106	0.00198	0.00058	0.00000	0.00000	0.00726	0.00428	0.00317	0.00241
O35344	IMA4_MOUSE	0.00044	0.00009	0.00118	0.00110	0.00114	0.00052	0.00742	0.00551	0.00537	0.00556	0.00450	0.00607
O35345	IMA7_MOUSE	0.00510	0.00630	0.00601	0.00164	0.00169	0.00519	0.00204	0.00599	0.00310	0.00422	0.00353	0.00501
O35350	CAN1_MOUSE	0.00390	0.00275	0.00190	0.00205	0.00256	0.00322	0.02847	0.02411	0.02771	0.02712	0.03143	0.02723
O35379	MRP1_MOUSE	0.00000	0.00000	0.00000	0.00000	0.00000	0.00000	0.00313	0.00233	0.00219	0.00227	0.00210	0.00197
O35381	AN32A_MOUSE	0.02036	0.02002	0.01946	0.01511	0.01652	0.01952	0.02870	0.03142	0.04752	0.03665	0.02898	0.03694
O35382	EXOC4_MOUSE	0.00000	0.00000	0.00000	0.00000	0.00000	0.00000	0.00160	0.00445	0.00564	0.00425	0.00600	0.00385
O35386	PAHX_MOUSE	0.00000	0.00000	0.00000	0.00000	0.00000	0.00000	0.00000	0.00000	0.00000	0.00000	0.00000	0.00000
O35387	HAX1_MOUSE	0.00000	0.00000	0.00000	0.00000	0.00000	0.00000	0.00000	0.00000	0.00000	0.00000	0.00000	0.00000
O35409	FOLH1_MOUSE	0.00000	0.00000	0.00000	0.00000	0.00000	0.00000	0.02353	0.02650	0.02296	0.01670	0.02433	0.02395
O35435	PYRD_MOUSE	0.00122	0.00509	0.00144	0.00189	0.00592	0.00167	0.00000	0.00132	0.00674	0.00330	0.00088	0.00148
O35457	CCRL2_MOUSE	0.00000	0.00000	0.00000	0.00000	0.00000	0.00000	0.00000	0.00000	0.00000	0.00000	0.00000	0.00000
O35459	ECH1_MOUSE	0.06631	0.06353	0.06182	0.05916	0.06337	0.09725	0.00801	0.01021	0.01092	0.01082	0.01192	0.01261
O35465	FKBP8_MOUSE	0.00127	0.00144	0.00089	0.00858	0.00097	0.00109	0.00075	0.00186	0.00057	0.00225	0.00000	0.00283
O35488	S27A2_MOUSE	0.00000	0.00000	0.00000	0.00000	0.00000	0.00000	0.00005	0.00002	0.00006	0.00009	0.00002	0.00007
O35566	CD151_MOUSE	0.00000	0.00000	0.00000	0.00000	0.00000	0.00000	0.00554	0.01834	0.02392	0.01846	0.01360	0.01587
O35593	PSDE_MOUSE	0.00000	0.00000	0.00000	0.00000	0.00000	0.00000	0.00950	0.01718	0.01305	0.01158	0.01836	0.00696
O35598	ADA10_MOUSE	0.00000	0.00000	0.00000	0.00000	0.00000	0.00000	0.00731	0.01119	0.00851	0.00959	0.00603	0.00870
O35604	NPC1_MOUSE	0.00000	0.00000	0.00000	0.00000	0.00000	0.00000	0.00000	0.00182	0.00128	0.00300	0.00000	0.00176
O35609	SCAM3_MOUSE	0.00000	0.00000	0.00000	0.00000	0.00000	0.00000	0.01282	0.01492	0.02090	0.01677	0.01392	0.01576
O35623	BET1_MOUSE	0.00000	0.00000	0.00000	0.00000	0.00000	0.00000	0.00788	0.00194	0.00621	0.00388	0.00479	0.00618
O35632	HYAL2_MOUSE	0.00000	0.00000	0.00000	0.00000	0.00000	0.00000	0.00147	0.00517	0.00244	0.00305	0.00120	0.00325
O35638	STAG2_MOUSE	0.00000	0.00000	0.00000	0.00000	0.00000	0.00000	0.00084	0.00169	0.00673	0.00341	0.00000	0.00694
O35639	ANXA3_MOUSE	0.00163	0.00652	0.00298	0.00273	0.00730	0.00407	0.10451	0.12614	0.13383	0.12980	0.13367	0.11457

O35640	ANXA8_MOUSE	0.00000	0.00000	0.00000	0.00000	0.00000	0.00000	0.00345	0.00185	0.00241	0.00317	0.00217	0.00488
O35643	AP1B1_MOUSE	0.00000	0.00000	0.00000	0.00017	0.00000	0.00000	0.00951	0.01073	0.01500	0.01178	0.01364	0.01272
O35648	CETN3_MOUSE	0.00000	0.00000	0.00000	0.00000	0.00000	0.00000	0.00539	0.00444	0.00479	0.00472	0.00666	0.00498
O35658	C1QBP_MOUSE	0.16297	0.12705	0.11578	0.11082	0.14698	0.10801	0.04582	0.05578	0.03047	0.04158	0.05122	0.04194
O35678	MGLL_MOUSE	0.00384	0.00561	0.00115	0.00369	0.00336	0.00240	0.00545	0.00105	0.00724	0.00408	0.00261	0.00232
O35682	MYADM_MOUSE	0.00000	0.00000	0.00000	0.00000	0.00000	0.00000	0.00904	0.03239	0.04020	0.03422	0.01961	0.02132
O35683	NDUA1_MOUSE	0.00000	0.00000	0.00000	0.00000	0.00000	0.00000	0.00090	0.00284	0.00066	0.00084	0.00057	0.00065
O35685	NUDC_MOUSE	0.00000	0.00000	0.00000	0.00000	0.00000	0.00000	0.00485	0.01559	0.02004	0.01585	0.00713	0.01417
O35691	PININ_MOUSE	0.00000	0.00000	0.00000	0.00000	0.00000	0.00000	0.00000	0.00311	0.00526	0.00502	0.00458	0.00511
O35704	SPTC1_MOUSE	0.00000	0.00000	0.00000	0.00000	0.00000	0.00000	0.00000	0.00000	0.00000	0.00000	0.00000	0.00000
O35737	HNRH1_MOUSE	0.00000	0.00000	0.00000	0.00000	0.00000	0.00000	0.11289	0.08273	0.11390	0.11053	0.12787	0.13315
O35744	CHIL3_MOUSE	0.00000	0.00000	0.00000	0.00000	0.00000	0.00000	0.03860	0.05774	0.03127	0.03666	0.04400	0.04433
O35841	API5_MOUSE	0.00000	0.00000	0.00000	0.00000	0.00000	0.00000	0.00759	0.01446	0.01453	0.01350	0.01264	0.01322
O35855	BCAT2_MOUSE	0.05432	0.06610	0.07002	0.06374	0.05235	0.05190	0.00704	0.01541	0.00876	0.00526	0.00990	0.00597
O35857	TIM44_MOUSE	0.00875	0.01016	0.00756	0.00469	0.00826	0.00494	0.00631	0.00657	0.00590	0.00609	0.00633	0.00565
O35864	CSN5_MOUSE	0.00000	0.00000	0.00000	0.00000	0.00000	0.00000	0.00754	0.00852	0.00797	0.00753	0.01019	0.00711
O35887	CALU_MOUSE	0.00651	0.00411	0.00591	0.00321	0.00386	0.00481	0.03574	0.03758	0.05232	0.04825	0.05219	0.04625
O35900	LSM2_MOUSE	0.00000	0.00000	0.00000	0.00000	0.00000	0.00000	0.00000	0.00000	0.01848	0.02699	0.03479	0.00000
O35926	CD5R2_MOUSE	0.00000	0.00000	0.00000	0.00000	0.00000	0.00000	0.06847	0.04853	0.10252	0.08332	0.14763	0.11455
O35945	AL1A7_MOUSE	0.00000	0.00000	0.00000	0.00000	0.00000	0.00000	0.04576	0.05789	0.03293	0.02825	0.05113	0.03635
O35955	PSB10_MOUSE	0.00000	0.00000	0.00000	0.00000	0.00000	0.00000	0.00736	0.00762	0.02942	0.02154	0.02527	0.02167
O35969	GAMT_MOUSE	0.00000	0.00000	0.00000	0.00000	0.00000	0.00000	0.00000	0.00000	0.00058	0.00022	0.00000	0.00024
O35988	SDC4_MOUSE	0.00000	0.00000	0.00000	0.00000	0.00000	0.00000	0.00000	0.00389	0.00000	0.00106	0.00139	0.00000
O54724	CAVN1_MOUSE	0.13900	0.17640	0.12648	0.11197	0.20667	0.22773	0.39554	0.47044	0.58507	0.50765	0.42552	0.54545
O54734	OST48_MOUSE	0.00292	0.00635	0.00191	0.00191	0.00502	0.00300	0.03443	0.03277	0.03139	0.03046	0.03169	0.03610
Q61080	FOXF1_MOUSE	0.00000	0.00000	0.00000	0.00000	0.00000	0.00000	0.00000	0.00464	0.00024	0.00000	0.00157	0.00150
O54749	CP2J5_MOUSE	0.00000	0.00000	0.00000	0.00000	0.00000	0.00000	0.00061	0.00094	0.00099	0.00072	0.00053	0.00000
O54774	AP3D1_MOUSE	0.00000	0.00000	0.00000	0.00000	0.00000	0.00000	0.00700	0.00950	0.00420	0.00595	0.00551	0.00973
O54824	IL16_MOUSE	0.00000	0.00000	0.00000	0.00000	0.00000	0.00000	0.00000	0.00524	0.00386	0.00482	0.00231	0.00504
O54833	CSK22_MOUSE	0.00000	0.00000	0.00000	0.00000	0.00000	0.00000	0.00511	0.00491	0.00412	0.00301	0.00264	0.00404
O54834	RHG06_MOUSE	0.00000	0.00000	0.00000	0.00000	0.00000	0.00000	0.00000	0.00000	0.00106	0.00000	0.00051	0.00016
O54865	GCYB1_MOUSE	0.00000	0.00000	0.00000	0.00000	0.00000	0.00000	0.01501	0.02441	0.02147	0.02384	0.02149	0.02510
O54890	ITB3_MOUSE	0.00000	0.00000	0.00000	0.00000	0.00000	0.00000	0.00099	0.00000	0.00691	0.00620	0.00303	0.00142
O54901	OX2G_MOUSE	0.00000	0.00000	0.00000	0.00000	0.00000	0.00000	0.00359	0.01524	0.01049	0.01081	0.00489	0.00739

O54909	RDH16_MOUSE	0.00000	0.00000	0.00000	0.00000	0.00000	0.00000	0.00000	0.00000	0.00000	0.00000	0.00000	0.00000
O54931	AKAP2_MOUSE	0.00123	0.00136	0.00095	0.00134	0.00095	0.00137	0.11331	0.14680	0.18254	0.15018	0.15529	0.14960
O54940	BNIP2_MOUSE	0.00000	0.00000	0.00000	0.00000	0.00000	0.00000	0.00336	0.00069	0.00347	0.00169	0.00199	0.00225
O54941	SMCE1_MOUSE	0.00000	0.00000	0.00000	0.00000	0.00000	0.00000	0.00093	0.00494	0.00114	0.00202	0.00000	0.00335
O54942	CLD5_MOUSE	0.00000	0.00000	0.00000	0.00000	0.00000	0.00000	0.00809	0.03377	0.02348	0.01747	0.02316	0.03312
O54950	AAKG1_MOUSE	0.00000	0.00000	0.00000	0.00000	0.00000	0.00000	0.01998	0.01891	0.02388	0.01763	0.02668	0.01686
O54962	BAF_MOUSE	0.00000	0.00000	0.00000	0.00000	0.00000	0.00000	0.01499	0.01966	0.00681	0.01581	0.00448	0.02591
O54974	LEG7_MOUSE	0.00000	0.00000	0.00000	0.00000	0.00000	0.00000	0.00279	0.00000	0.00000	0.00000	0.00000	0.00000
O54984	GET3_MOUSE	0.00000	0.00000	0.00000	0.00000	0.00000	0.00000	0.01381	0.01385	0.01922	0.01706	0.02080	0.01720
O54988	SLK_MOUSE	0.00165	0.00106	0.00056	0.00090	0.00066	0.00113	0.00727	0.01671	0.01623	0.01898	0.00852	0.01736
O55013	TPPC3_MOUSE	0.00000	0.00000	0.00000	0.00000	0.00000	0.00000	0.00247	0.00377	0.00863	0.00655	0.00424	0.00716
O55022	PGRC1_MOUSE	0.00000	0.00000	0.00000	0.00000	0.00000	0.00000	0.04640	0.06441	0.08077	0.07285	0.09062	0.08864
O55023	IMPA1_MOUSE	0.00000	0.00000	0.00000	0.00000	0.00000	0.00000	0.00960	0.02307	0.02159	0.01752	0.01852	0.01497
O55026	ENTP2_MOUSE	0.00000	0.00000	0.00000	0.00000	0.00000	0.00000	0.00000	0.00472	0.00438	0.00335	0.00141	0.00154
O55029	COPB2_MOUSE	0.00000	0.00000	0.00000	0.00000	0.00000	0.00000	0.04316	0.02711	0.03781	0.03567	0.04158	0.03221
O55042	SYUA_MOUSE	0.00000	0.00000	0.00000	0.00000	0.00000	0.00000	0.00278	0.00099	0.00187	0.00341	0.00393	0.00134
O55060	TPMT_MOUSE	0.00000	0.00000	0.00000	0.00000	0.00000	0.00000	0.00078	0.00138	0.00472	0.00326	0.00356	0.00354
O55071	CP2BJ_MOUSE	0.00000	0.00000	0.00000	0.00000	0.00000	0.00000	0.04363	0.02015	0.04170	0.03336	0.04914	0.03558
O55098	STK10_MOUSE	0.00000	0.00000	0.00000	0.00000	0.00000	0.00000	0.00000	0.00000	0.00072	0.00050	0.00153	0.00022
O55101	SNG2_MOUSE	0.00000	0.00000	0.00000	0.00000	0.00000	0.00000	0.01695	0.01142	0.00834	0.00756	0.01165	0.01363
O55103	PRAX_MOUSE	0.00000	0.00000	0.00000	0.00000	0.00000	0.00000	0.11240	0.08991	0.10913	0.09316	0.14096	0.12137
O55106	STRN_MOUSE	0.00125	0.00166	0.00111	0.00105	0.00191	0.00218	0.00610	0.00876	0.00873	0.00581	0.01105	0.01164
O55125	NIPS1_MOUSE	0.00000	0.00000	0.00000	0.00000	0.00000	0.00000	0.00275	0.00668	0.00026	0.00289	0.00000	0.00155
O55126	NIPS2_MOUSE	0.14554	0.08151	0.14317	0.13923	0.11131	0.16624	0.03907	0.03334	0.03961	0.03453	0.03785	0.03468
O55128	SAP18_MOUSE	0.00000	0.00000	0.00000	0.00000	0.00000	0.00000	0.00123	0.00089	0.00240	0.00248	0.00393	0.00351
O55131	SEPT7_MOUSE	0.00540	0.00598	0.00451	0.00468	0.00406	0.00246	0.05108	0.04788	0.07034	0.05322	0.07844	0.05986
O55135	IF6_MOUSE	0.00000	0.00000	0.00000	0.00000	0.00000	0.00000	0.00580	0.00358	0.00879	0.00613	0.00904	0.00472
O55137	ACOT1_MOUSE	0.00000	0.00000	0.00000	0.00000	0.00000	0.00000	0.00792	0.00494	0.00892	0.00718	0.01156	0.00854
O55142	RL35A_MOUSE	0.00000	0.00000	0.00000	0.00000	0.00000	0.00000	0.00000	0.00651	0.00144	0.00388	0.00080	0.00234
O55143	AT2A2_MOUSE	1.44816	1.65616	1.50177	1.49384	1.59797	1.89910	0.09990	0.08486	0.08843	0.11060	0.10149	0.08683
O55192	SC6A2_MOUSE	0.00000	0.00000	0.00000	0.00000	0.00000	0.00000	0.00000	0.00000	0.00278	0.00176	0.00135	0.00127
O55201	SPT5H_MOUSE	0.00000	0.00000	0.00000	0.00000	0.00000	0.00000	0.00426	0.00644	0.00660	0.00479	0.00866	0.00789
O55222	ILK_MOUSE	0.00635	0.00300	0.00604	0.00350	0.00440	0.00368	0.07372	0.12673	0.11869	0.11803	0.07829	0.14137
O55226	CHAD_MOUSE	0.00000	0.00000	0.00000	0.00000	0.00000	0.00000	0.02448	0.00154	0.00333	0.00000	0.00513	0.00000

O55229	CHKB_MOUSE	0.00000	0.00000	0.00000	0.00000	0.00000	0.00000	0.00000	0.00205	0.00358	0.00213	0.00176	0.00354
O55234	PSB5_MOUSE	0.00326	0.00309	0.00678	0.00594	0.00408	0.00289	0.00529	0.00642	0.01037	0.00939	0.00708	0.00583
O55242	SGMR1_MOUSE	0.00000	0.00000	0.00000	0.00000	0.00000	0.00000	0.00000	0.00000	0.00000	0.00000	0.00000	0.00000
O70133	DHX9_MOUSE	0.00000	0.00000	0.00000	0.00000	0.00000	0.00000	0.02764	0.03321	0.04498	0.04175	0.04757	0.03875
O70138	MMP8_MOUSE	0.00000	0.00000	0.00000	0.00000	0.00000	0.00000	0.00000	0.00000	0.00000	0.00000	0.00028	0.00191
O70152	DPM1_MOUSE	0.00000	0.00000	0.00000	0.00000	0.00000	0.00000	0.00305	0.00442	0.00495	0.00580	0.00581	0.00169
O70172	PI42A_MOUSE	0.00000	0.00000	0.00000	0.00000	0.00000	0.00000	0.00000	0.00000	0.00000	0.00000	0.00297	0.00000
O70194	EIF3D_MOUSE	0.00000	0.00000	0.00000	0.00000	0.00000	0.00000	0.01004	0.01138	0.01076	0.01157	0.01460	0.01114
O70209	PDLI3_MOUSE	0.00000	0.00000	0.00000	0.00000	0.00000	0.00000	0.01826	0.00219	0.00393	0.00454	0.00355	0.00586
O70250	PGAM2_MOUSE	0.00000	0.00000	0.00000	0.00000	0.00000	0.00000	0.02709	0.01132	0.01688	0.02160	0.00425	0.01163
O70251	EF1B_MOUSE	0.03693	0.01284	0.03034	0.01865	0.02797	0.01892	0.05159	0.03728	0.06146	0.04111	0.06095	0.05715
O70252	HMOX2_MOUSE	0.00495	0.00291	0.00232	0.00359	0.00448	0.00447	0.00922	0.00802	0.01270	0.00940	0.01300	0.00959
O70274	TP4A2_MOUSE	0.00000	0.00000	0.00000	0.00000	0.00000	0.00000	0.00000	0.00141	0.00337	0.00126	0.00141	0.00106
O70305	ATX2_MOUSE	0.01129	0.00940	0.01062	0.01029	0.00885	0.00474	0.00235	0.00347	0.00411	0.00087	0.00222	0.00211
O70309	ITB5_MOUSE	0.00000	0.00000	0.00000	0.00000	0.00000	0.00000	0.00253	0.00573	0.00185	0.00094	0.00269	0.00476
O70310	NMT1_MOUSE	0.00069	0.00221	0.00104	0.00092	0.00126	0.00142	0.01422	0.01484	0.01308	0.01379	0.01682	0.01744
O70318	E41L2_MOUSE	0.00786	0.01790	0.00668	0.00377	0.00755	0.00938	0.02855	0.04032	0.05284	0.04225	0.04893	0.03996
O70324	MOT8_MOUSE	0.00000	0.00000	0.00000	0.00000	0.00000	0.00000	0.00000	0.00000	0.00000	0.00000	0.00000	0.00000
O70325	GPX4_MOUSE	0.00000	0.00000	0.00000	0.00000	0.00000	0.00000	0.01576	0.02008	0.02397	0.02281	0.01984	0.02206
O70370	CATS_MOUSE	0.00000	0.00000	0.00000	0.00000	0.00000	0.00000	0.01102	0.00636	0.00440	0.00826	0.00799	0.01226
O70378	EMC8_MOUSE	0.00000	0.00000	0.00000	0.00000	0.00000	0.00000	0.00226	0.00134	0.00490	0.00319	0.00623	0.00339
O70400	PDLI1_MOUSE	0.00449	0.00337	0.00254	0.00402	0.00394	0.00596	0.01357	0.01368	0.01684	0.01462	0.01441	0.01503
O70404	VAMP8_MOUSE	0.00000	0.00000	0.00000	0.00000	0.00000	0.00000	0.00000	0.01206	0.01871	0.01541	0.00844	0.00854
O70423	AOC3_MOUSE	0.00417	0.00444	0.00324	0.00256	0.00374	0.00655	0.01180	0.00809	0.02303	0.00814	0.01101	0.00714
O70435	PSA3_MOUSE	0.00000	0.00000	0.00000	0.00000	0.00000	0.00000	0.02213	0.02944	0.01980	0.03104	0.01474	0.02217
O70439	STX7_MOUSE	0.00217	0.00393	0.00279	0.00270	0.00348	0.00325	0.02469	0.03224	0.02803	0.04151	0.02294	0.03447
O70456	1433S_MOUSE	0.00000	0.00000	0.00000	0.00000	0.00000	0.00000	0.05464	0.01776	0.01848	0.01507	0.01852	0.01366
O70468	MYPC3_MOUSE	1.14491	1.46768	1.01573	1.28842	1.17675	1.42079	0.01626	0.01509	0.01771	0.02364	0.01591	0.01365
O70475	UGDH_MOUSE	0.00000	0.00000	0.00000	0.00000	0.00000	0.00000	0.00338	0.00847	0.00909	0.00847	0.00660	0.00464
O70492	SNX3_MOUSE	0.00000	0.00000	0.00000	0.00000	0.00000	0.00000	0.01709	0.01691	0.00836	0.01212	0.00772	0.01313
O70493	SNX12_MOUSE	0.00000	0.00000	0.00000	0.00000	0.00000	0.00000	0.00804	0.01290	0.00442	0.00669	0.00123	0.00922
O70503	DHB12_MOUSE	0.00373	0.00296	0.00191	0.00165	0.00193	0.00289	0.02274	0.00789	0.01741	0.01017	0.00426	0.01818
O70551	SRPK1_MOUSE	0.00000	0.00000	0.00000	0.00000	0.00000	0.00000	0.00000	0.00000	0.00105	0.00000	0.00000	0.00000
O70566	DIAP2_MOUSE	0.00000	0.00000	0.00000	0.00000	0.00000	0.00000	0.00000	0.00000	0.00347	0.00114	0.00000	0.00087

O70572	NSMA_MOUSE	0.00000	0.00000	0.00000	0.00000	0.00000	0.00000	0.00116	0.00000	0.00085	0.00055	0.00000	0.00103
O70577	S22A2_MOUSE	0.00000	0.00000	0.00000	0.00000	0.00000	0.00000	0.00365	0.00143	0.00667	0.00134	0.00957	0.00335
O70589	CSKP_MOUSE	0.00000	0.00000	0.00000	0.00000	0.00000	0.00000	0.00095	0.00231	0.00167	0.00095	0.00213	0.00133
O70591	PFD2_MOUSE	0.00000	0.00000	0.00000	0.00000	0.00000	0.00000	0.00000	0.00000	0.00142	0.00000	0.00163	0.00234
O70622	RTN2_MOUSE	0.04400	0.02521	0.03992	0.02684	0.02929	0.01523	0.01080	0.00415	0.00197	0.00476	0.00455	0.00214
O88207	CO5A1_MOUSE	0.00000	0.00000	0.00000	0.00000	0.00000	0.00000	0.01688	0.01349	0.01307	0.01122	0.01340	0.01403
O88271	CFDP1_MOUSE	0.00000	0.00000	0.00000	0.00000	0.00000	0.00000	0.00000	0.00172	0.00319	0.00163	0.00372	0.00220
O88307	SORL_MOUSE	0.00000	0.00000	0.00000	0.00000	0.00000	0.00000	0.00106	0.00309	0.00306	0.00319	0.00265	0.00000
O88322	NID2_MOUSE	0.00593	0.00923	0.00490	0.00435	0.00514	0.00492	0.01956	0.01854	0.02892	0.01934	0.02512	0.02575
O88338	CAD16_MOUSE	0.00000	0.00000	0.00000	0.00000	0.00000	0.00000	0.00000	0.00000	0.00000	0.00000	0.00000	0.00000
O88342	WDR1_MOUSE	0.04328	0.02247	0.04284	0.03179	0.04067	0.03205	0.06474	0.05616	0.06805	0.06141	0.07584	0.05307
O88343	S4A4_MOUSE	0.00000	0.00000	0.00000	0.00000	0.00000	0.00000	0.00000	0.00000	0.00029	0.00016	0.00000	0.00000
O88384	VTI1B_MOUSE	0.00000	0.00000	0.00000	0.00000	0.00000	0.00000	0.00141	0.00248	0.00606	0.00558	0.00502	0.00364
O88428	PAPS2_MOUSE	0.00000	0.00000	0.00000	0.00000	0.00000	0.00000	0.00624	0.01214	0.00952	0.01141	0.01395	0.01311
O88441	MTX2_MOUSE	0.00392	0.00400	0.00746	0.00326	0.00372	0.00136	0.00140	0.00327	0.00651	0.00410	0.00174	0.00236
O88447	KLC1_MOUSE	0.00000	0.00000	0.00000	0.00000	0.00000	0.00000	0.01153	0.01513	0.01621	0.01479	0.01749	0.01164
O88456	CPNS1_MOUSE	0.00000	0.00000	0.00000	0.00000	0.00000	0.00000	0.02766	0.03228	0.03039	0.03091	0.03625	0.02206
O88487	DC1I2_MOUSE	0.00356	0.00222	0.00561	0.00534	0.00312	0.00092	0.02317	0.02768	0.04949	0.03872	0.05020	0.03595
O88492	PLIN4_MOUSE	0.01024	0.08756	0.01048	0.03585	0.01015	0.00978	0.00178	0.00550	0.00777	0.00497	0.00249	0.00191
O88531	PPT1_MOUSE	0.00752	0.00508	0.01047	0.00547	0.00613	0.00470	0.01687	0.01366	0.01171	0.00973	0.00629	0.00905
O88532	ZFR_MOUSE	0.00000	0.00000	0.00000	0.00000	0.00000	0.00000	0.00000	0.00000	0.00000	0.00000	0.00000	0.00000
O88533	DDC_MOUSE	0.00000	0.00000	0.00000	0.00000	0.00000	0.00000	0.00000	0.00000	0.00000	0.00000	0.00000	0.00000
O88543	CSN3_MOUSE	0.00559	0.01012	0.00601	0.00514	0.01378	0.00442	0.00297	0.00948	0.00775	0.00770	0.01022	0.00642
O88544	CSN4_MOUSE	0.00248	0.00318	0.00229	0.00388	0.00292	0.00364	0.00728	0.01229	0.01100	0.00972	0.00775	0.01245
O88545	CSN6_MOUSE	0.00278	0.00307	0.00369	0.00291	0.00377	0.00239	0.00687	0.00592	0.00973	0.00710	0.00914	0.00698
O88552	CLD2_MOUSE	0.00000	0.00000	0.00000	0.00000	0.00000	0.00000	0.00000	0.00000	0.00000	0.00000	0.00000	0.00000
O88569	ROA2_MOUSE	0.01413	0.01177	0.00778	0.01254	0.01564	0.01288	0.17440	0.20151	0.21064	0.19423	0.22259	0.21060
O88572	LRP6_MOUSE	0.00000	0.00000	0.00000	0.00000	0.00000	0.00000	0.00649	0.00964	0.01409	0.01385	0.01539	0.00972
O88576	S6A18_MOUSE	0.00000	0.00000	0.00000	0.00000	0.00000	0.00000	0.00000	0.00000	0.00000	0.00091	0.00000	0.00082
O88587	COMT_MOUSE	0.00000	0.00000	0.00000	0.00000	0.00000	0.00000	0.00471	0.00969	0.01161	0.00907	0.01006	0.00786
O88630	GOSR1_MOUSE	0.00000	0.00000	0.00000	0.00000	0.00000	0.00000	0.00296	0.00252	0.00492	0.00393	0.00353	0.00326
O88668	CREG1_MOUSE	0.00000	0.00000	0.00000	0.00000	0.00000	0.00000	0.00690	0.00323	0.00658	0.00573	0.00758	0.00413
O88685	PRS6A_MOUSE	0.00574	0.00569	0.00630	0.00419	0.00475	0.00556	0.02804	0.03096	0.02917	0.02870	0.03665	0.03379
O88696	CLPP_MOUSE	0.02316	0.01075	0.02732	0.01307	0.01607	0.01530	0.01546	0.00495	0.00818	0.00908	0.00712	0.01029

O88712	CTBP1_MOUSE	0.00000	0.00000	0.00000	0.00000	0.00000	0.00000	0.00625	0.00735	0.00981	0.00797	0.00882	0.00903
O88746	TOM1_MOUSE	0.00577	0.00510	0.00289	0.00282	0.00321	0.00293	0.00580	0.00598	0.00612	0.00524	0.00590	0.00669
O88792	JAM1_MOUSE	0.00000	0.00000	0.00000	0.00000	0.00000	0.00000	0.02303	0.01940	0.02352	0.01596	0.01766	0.02398
O88811	STAM2_MOUSE	0.00000	0.00000	0.00000	0.00000	0.00000	0.00000	0.00472	0.00153	0.00553	0.00472	0.00391	0.00473
O88844	IDHC_MOUSE	0.01141	0.01263	0.01563	0.01043	0.01735	0.02056	0.03919	0.03230	0.03478	0.03018	0.03284	0.03428
O88848	ARL6_MOUSE	0.00000	0.00000	0.00000	0.00000	0.00000	0.00000	0.00000	0.00000	0.00052	0.00076	0.00000	0.00054
O88851	RBBP9_MOUSE	0.00000	0.00000	0.00000	0.00000	0.00000	0.00000	0.01123	0.00901	0.01791	0.01729	0.02080	0.01496
O88909	S22A8_MOUSE	0.00000	0.00000	0.00000	0.00000	0.00000	0.00000	0.00000	0.00000	0.00000	0.00000	0.00000	0.00000
O88952	LIN7C_MOUSE	0.00000	0.00000	0.00000	0.00000	0.00000	0.00000	0.00000	0.00751	0.00771	0.00667	0.00318	0.00808
O88958	GNPI1_MOUSE	0.00000	0.00000	0.00000	0.00000	0.00000	0.00000	0.00082	0.00267	0.00792	0.00967	0.00532	0.00766
O88968	TCO2_MOUSE	0.00000	0.00000	0.00000	0.00000	0.00000	0.00000	0.00000	0.00000	0.00266	0.00134	0.00152	0.00228
O88983	STX8_MOUSE	0.00000	0.00000	0.00000	0.00000	0.00000	0.00000	0.00412	0.00223	0.00839	0.00501	0.00876	0.00765
O88986	KBL_MOUSE	0.00000	0.00000	0.00000	0.00000	0.00000	0.00000	0.00000	0.00134	0.00000	0.00000	0.00000	0.00000
O88990	ACTN3_MOUSE	0.00000	0.00000	0.00000	0.00000	0.00000	0.00000	0.02413	0.00001	0.00012	0.00023	0.00004	0.00010
O89017	LGMN_MOUSE	0.00000	0.00000	0.00000	0.00000	0.00000	0.00000	0.00000	0.00146	0.00000	0.00063	0.00000	0.00053
O89023	TPP1_MOUSE	0.00000	0.00000	0.00000	0.00000	0.00000	0.00000	0.00759	0.01135	0.01623	0.01049	0.01056	0.00762
O89051	ITM2B_MOUSE	0.00000	0.00000	0.00000	0.00000	0.00000	0.00000	0.01654	0.01291	0.01548	0.01401	0.01589	0.01587
O89053	COR1A_MOUSE	0.00000	0.00000	0.00000	0.00000	0.00000	0.00000	0.00820	0.02426	0.04965	0.05185	0.03731	0.04082
O89079	COPE_MOUSE	0.00699	0.00794	0.00699	0.00632	0.00630	0.00639	0.03847	0.02991	0.04390	0.03961	0.04883	0.04044
O89086	RBM3_MOUSE	0.00000	0.00000	0.00000	0.00000	0.00000	0.00000	0.00599	0.00887	0.00740	0.00917	0.00372	0.00885
O89090	SP1_MOUSE	0.00000	0.00000	0.00000	0.00000	0.00000	0.00000	0.00153	0.00000	0.00583	0.00414	0.00601	0.00129
O89103	C1QR1_MOUSE	0.00000	0.00000	0.00000	0.00000	0.00000	0.00000	0.02150	0.02915	0.02473	0.02446	0.02137	0.02275
O89104	SYPL2_MOUSE	0.00000	0.00000	0.00000	0.00000	0.00000	0.00000	0.00000	0.00000	0.00000	0.00000	0.00000	0.00000
O89106	FHIT_MOUSE	0.00000	0.00000	0.00000	0.00000	0.00000	0.00000	0.00000	0.00000	0.00000	0.00000	0.00000	0.00000
O89110	CASP8_MOUSE	0.00000	0.00000	0.00000	0.00000	0.00000	0.00000	0.01183	0.00173	0.00348	0.00336	0.00408	0.00528
O89112	LANC1_MOUSE	0.00000	0.00000	0.00000	0.00000	0.00000	0.00000	0.00122	0.00488	0.00563	0.00546	0.00205	0.00448
P00329	ADH1_MOUSE	0.00000	0.00000	0.00000	0.00000	0.00000	0.00000	0.13367	0.08024	0.16402	0.12358	0.17508	0.13153
P00375	DYR_MOUSE	0.00000	0.00000	0.00000	0.00000	0.00000	0.00000	0.00011	0.00010	0.00000	0.00000	0.00000	0.00092
P00397	COX1_MOUSE	0.01005	0.00577	0.01046	0.01216	0.00735	0.01099	0.00311	0.00877	0.01007	0.00955	0.00389	0.00474
P00405	COX2_MOUSE	0.47796	0.29714	0.34847	0.26250	0.30274	0.36478	0.10900	0.06875	0.07307	0.07417	0.06895	0.06725
P00416	COX3_MOUSE	0.00000	0.00000	0.00000	0.00000	0.00000	0.00000	0.00000	0.01490	0.03080	0.02341	0.01058	0.00991
P00493	HPRT_MOUSE	0.00000	0.00000	0.00000	0.00000	0.00000	0.00000	0.00482	0.00466	0.00315	0.00520	0.00000	0.00553
P00848	ATP6_MOUSE	0.00000	0.00000	0.00000	0.00000	0.00000	0.00000	0.00068	0.00334	0.00642	0.00474	0.00167	0.00210
P00920	CAH2_MOUSE	0.00813	0.00622	0.02021	0.03125	0.01835	0.00396	0.11793	0.07967	0.08465	0.16395	0.19844	0.05913

P01027	CO3_MOUSE	0.03792	0.02121	0.01650	0.04202	0.03691	0.01949	0.07329	0.04394	0.06791	0.08972	0.11542	0.06382
P01029	CO4B_MOUSE	0.00238	0.00030	0.00301	0.00219	0.00200	0.00121	0.03848	0.02524	0.03459	0.02464	0.04049	0.02741
P01592	IGJ_MOUSE	0.00000	0.00000	0.00000	0.00000	0.00000	0.00000	0.01022	0.00359	0.00685	0.01320	0.00861	0.00000
P01631	KV2A7_MOUSE	0.00000	0.00000	0.00000	0.00000	0.00000	0.00000	0.00944	0.00559	0.01272	0.01086	0.00717	0.00447
P01635	KV5A3_MOUSE	0.00000	0.00000	0.00000	0.00000	0.00000	0.00000	0.00000	0.00000	0.00000	0.00000	0.00000	0.00000
P01638	KV5A6_MOUSE	0.00000	0.00000	0.00000	0.00000	0.00000	0.00000	0.00000	0.00000	0.00000	0.00000	0.01334	0.00000
P01645	KV5AC_MOUSE	0.00000	0.00000	0.00000	0.00000	0.00000	0.00000	0.00731	0.01419	0.01212	0.00502	0.01362	0.02006
P01655	KV3A2_MOUSE	0.00000	0.00000	0.00000	0.00000	0.00000	0.00000	0.01540	0.00343	0.00353	0.00155	0.00271	0.00000
P01786	HVM17_MOUSE	0.00000	0.00000	0.00000	0.00000	0.00000	0.00000	0.00000	0.00000	0.00069	0.00024	0.00000	0.00000
P01800	HVM31_MOUSE	0.00000	0.00000	0.00000	0.00000	0.00000	0.00000	0.00000	0.00000	0.00321	0.00298	0.00386	0.00000
P01831	THY1_MOUSE	0.00000	0.00000	0.00000	0.00000	0.00000	0.00000	0.00488	0.00429	0.00843	0.00553	0.00656	0.00600
P01837	IGKC_MOUSE	0.00000	0.00000	0.00000	0.00000	0.00000	0.00000	0.03834	0.05182	0.02811	0.02860	0.02662	0.03359
P01843	LAC1_MOUSE	0.00000	0.00000	0.00000	0.00000	0.00000	0.00000	0.00000	0.00000	0.01123	0.00247	0.00246	0.00000
P01864	GCAB_MOUSE	0.00287	0.00661	0.00365	0.01139	0.00953	0.00397	0.04358	0.01070	0.03061	0.00925	0.01632	0.02068
P01867	IGG2B_MOUSE	0.00464	0.00508	0.00257	0.01038	0.02356	0.01281	0.01579	0.00522	0.00613	0.00359	0.00000	0.01415
P01868	IGHG1_MOUSE	0.00512	0.00112	0.00323	0.00191	0.00188	0.00226	0.00837	0.02026	0.02346	0.03575	0.03036	0.02120
P01872	IGHM_MOUSE	0.04556	0.02037	0.02000	0.03054	0.02326	0.02160	0.06903	0.04198	0.06036	0.04591	0.06671	0.02297
P01898	HA10_MOUSE	0.00740	0.00385	0.00571	0.00601	0.00514	0.00389	0.00037	0.00496	0.00367	0.01713	0.00940	0.00099
P01899	HA11_MOUSE	0.01613	0.01514	0.00715	0.00574	0.00596	0.00956	0.03277	0.04500	0.04456	0.04107	0.03375	0.04191
P01901	HA1B_MOUSE	0.00000	0.00000	0.00000	0.00000	0.00000	0.00000	0.02956	0.03680	0.03262	0.03659	0.02014	0.03483
P01942	HBA_MOUSE	0.30719	0.17303	0.40813	0.84134	0.39810	0.13502	3.08705	2.09247	1.18206	2.65642	2.84898	1.48933
P02088	HBB1_MOUSE	0.86314	0.49864	0.64415	1.55827	0.93174	0.42266	3.10491	1.66747	1.53052	2.90289	3.23936	1.53755
P02089	HBB2_MOUSE	0.00000	0.00000	0.00000	0.00000	0.00000	0.00000	0.01182	0.00174	0.00202	0.01123	0.00140	0.00016
P84244	H33_MOUSE	0.00000	0.00000	0.00000	0.00000	0.00000	0.00000	0.00363	0.44305	0.44043	0.42918	0.42183	0.68807
P02463	CO4A1_MOUSE	0.00000	0.00000	0.00000	0.00000	0.00000	0.00000	0.10389	0.05505	0.05345	0.03295	0.13577	0.06042
P02468	LAMC1_MOUSE	0.07037	0.14064	0.06665	0.08988	0.06984	0.09869	0.24591	0.16723	0.19359	0.12873	0.27580	0.22811
P02469	LAMB1_MOUSE	0.01252	0.02321	0.02420	0.02306	0.02982	0.01375	0.03159	0.02562	0.02400	0.02151	0.02437	0.02941
P03888	NU1M_MOUSE	0.00674	0.00251	0.00580	0.00397	0.00552	0.00438	0.00334	0.00986	0.00744	0.00779	0.00645	0.00753
P03911	NU4M_MOUSE	0.00998	0.00247	0.01284	0.01173	0.00304	0.00350	0.00000	0.00141	0.00000	0.00133	0.00000	0.00114
P03921	NU5M_MOUSE	0.03616	0.02648	0.06620	0.05066	0.03110	0.03293	0.00475	0.00000	0.00000	0.00346	0.00000	0.00114
P03930	ATP8_MOUSE	0.00000	0.00000	0.00000	0.00000	0.00000	0.00000	0.02442	0.01074	0.00370	0.01509	0.00000	0.01675
P03953	CFAD_MOUSE	0.00000	0.00000	0.00000	0.00000	0.00000	0.00000	0.00375	0.00355	0.02006	0.00402	0.01650	0.00132
P03958	ADA_MOUSE	0.00000	0.00000	0.00000	0.00000	0.00000	0.00000	0.03126	0.00033	0.00028	0.00020	0.00022	0.00040
P03987	IGHG3_MOUSE	0.02288	0.00396	0.00207	0.00557	0.00566	0.00221	0.00570	0.03472	0.03505	0.04252	0.00349	0.00049

P04117	FABP4_MOUSE	0.00000	0.00000	0.00000	0.00000	0.00000	0.00000	0.10977	0.02073	0.09291	0.02080	0.01436	0.01968
P04186	CFAB_MOUSE	0.00760	0.00251	0.00513	0.00820	0.00787	0.00322	0.00960	0.00623	0.00825	0.01100	0.00963	0.00588
P04202	TGFB1_MOUSE	0.00000	0.00000	0.00000	0.00000	0.00000	0.00000	0.00000	0.00000	0.00000	0.00000	0.00000	0.00000
P04223	HA1K_MOUSE	0.00000	0.00000	0.00000	0.00000	0.00000	0.00000	0.00000	0.00837	0.00599	0.00744	0.01365	0.00000
P04231	HB23_MOUSE	0.00000	0.00000	0.00000	0.00000	0.00000	0.00000	0.00232	0.00140	0.00545	0.00562	0.00171	0.00531
P04247	MYG_MOUSE	1.77300	1.21328	2.10444	1.55629	1.18683	1.15700	0.18588	0.16688	0.29073	0.24547	0.14261	0.13691
P04370	MBP_MOUSE	0.00000	0.00000	0.00000	0.00000	0.00000	0.00000	0.01377	0.00354	0.02378	0.00318	0.00531	0.00068
P04441	HG2A_MOUSE	0.00000	0.00000	0.00000	0.00000	0.00000	0.00000	0.01219	0.00461	0.01428	0.01426	0.01736	0.01363
P04444	HBBZ_MOUSE	0.00000	0.00000	0.00000	0.00000	0.00000	0.00000	0.00554	0.00000	0.00000	0.00221	0.00313	0.00000
P04627	ARAF_MOUSE	0.00000	0.00000	0.00000	0.00000	0.00000	0.00000	0.00000	0.00000	0.00328	0.00109	0.00000	0.00132
P04919	B3AT_MOUSE	0.00178	0.00000	0.00127	0.00451	0.00254	0.00000	0.03696	0.01287	0.02138	0.05913	0.09335	0.00968
P04925	PRIO_MOUSE	0.00000	0.00000	0.00000	0.00000	0.00000	0.00000	0.00442	0.00373	0.00511	0.00230	0.00339	0.00222
P04939	MUP3_MOUSE	0.00000	0.00000	0.00000	0.00000	0.00000	0.00000	0.00000	0.00000	0.00012	0.00030	0.00023	0.00000
P04945	KV6AB_MOUSE	0.00000	0.00000	0.00000	0.00000	0.00000	0.00000	0.00000	0.00057	0.00000	0.00081	0.00317	0.00417
P05063	ALDOC_MOUSE	0.00081	0.00083	0.00032	0.00033	0.00045	0.00069	0.00000	0.00000	0.00116	0.00000	0.00000	0.00024
P05064	ALDOA_MOUSE	0.27817	0.19847	0.18079	0.18004	0.21568	0.21036	0.20759	0.10215	0.10412	0.11183	0.08597	0.11513
P05125	ANF_MOUSE	0.00000	0.00000	0.00000	0.00000	0.00000	0.00000	0.00986	0.00285	0.00621	0.00441	0.00254	0.00268
P05132	KAPCA_MOUSE	0.00236	0.00429	0.00373	0.00526	0.00433	0.00277	0.00916	0.00844	0.01310	0.00955	0.01445	0.01251
P05201	AATC_MOUSE	0.27636	0.27335	0.39299	0.32050	0.27746	0.28145	0.02199	0.02303	0.02391	0.02439	0.02300	0.01837
P05202	AATM_MOUSE	0.78606	0.82250	0.77606	0.77517	0.77903	0.94655	0.04909	0.09123	0.06193	0.07493	0.03822	0.06960
P05213	TBA1B_MOUSE	0.00000	0.00000	0.00000	0.00000	0.00000	0.00000	0.65389	0.81730	0.53446	0.67304	0.54813	0.79138
P05480	SRC_MOUSE	0.00000	0.00000	0.00000	0.00000	0.00000	0.00000	0.00599	0.01070	0.00531	0.00585	0.00318	0.00754
P05532	KIT_MOUSE	0.00000	0.00000	0.00000	0.00000	0.00000	0.00000	0.00000	0.00128	0.00064	0.00164	0.00000	0.00084
P05555	ITAM_MOUSE	0.00000	0.00000	0.00000	0.00000	0.00000	0.00000	0.06827	0.03134	0.08689	0.05326	0.03436	0.01969
P05977	MYL1_MOUSE	0.00000	0.00000	0.00000	0.00000	0.00000	0.00000	0.09271	0.00050	0.00110	0.00056	0.00018	0.00000
P06151	LDHA_MOUSE	0.10535	0.12754	0.19901	0.16428	0.17784	0.11896	0.12600	0.12073	0.12219	0.11724	0.10887	0.10650
P06330	HVM51_MOUSE	0.00150	1.98719	0.62407	0.00196	0.02229	0.95809	0.00609	0.00191	0.00578	0.00302	0.00299	0.00298
P06537	GCR_MOUSE	0.00000	0.00000	0.00000	0.00000	0.00000	0.00000	0.00197	0.00141	0.00286	0.00121	0.00124	0.00305
P06683	CO9_MOUSE	0.00000	0.00000	0.00000	0.00000	0.00000	0.00000	0.00764	0.00874	0.00239	0.01452	0.00204	0.00000
P06684	CO5_MOUSE	0.00291	0.00195	0.00194	0.00217	0.00249	0.00277	0.01811	0.02149	0.02354	0.02959	0.02304	0.01969
P06728	APOA4_MOUSE	0.01252	0.00667	0.01002	0.01120	0.00803	0.00802	0.01351	0.01301	0.01660	0.02691	0.02865	0.01639
P06745	G6PI_MOUSE	0.28439	0.27186	0.36607	0.26552	0.28170	0.22603	0.08426	0.07516	0.08008	0.07443	0.08648	0.08308
P06797	CATL1_MOUSE	0.00000	0.00000	0.00000	0.00000	0.00000	0.00000	0.00075	0.00644	0.00372	0.00320	0.00157	0.00173
P06800	PTPRC_MOUSE	0.00000	0.00000	0.00000	0.00000	0.00000	0.00000	0.00743	0.00805	0.01645	0.01492	0.01727	0.01595

P06801	MAOX_MOUSE	0.01058	0.00603	0.00884	0.00549	0.00884	0.00804	0.04088	0.03044	0.03769	0.02348	0.01934	0.02809
P06802	ENPP1_MOUSE	0.00000	0.00000	0.00000	0.00000	0.00000	0.00000	0.00000	0.00000	0.00000	0.00000	0.00000	0.00000
P06837	NEUM_MOUSE	0.00000	0.00000	0.00000	0.00000	0.00000	0.00000	0.00000	0.00320	0.00617	0.00500	0.00484	0.00245
P06869	UROK_MOUSE	0.00000	0.00000	0.00000	0.00000	0.00000	0.00000	0.00000	0.00042	0.00000	0.00055	0.00078	0.00036
P06909	CFAH_MOUSE	0.01539	0.01913	0.00928	0.01257	0.01092	0.01078	0.01869	0.01595	0.01305	0.01611	0.02093	0.01678
P07091	S10A4_MOUSE	0.00000	0.00000	0.00000	0.00000	0.00000	0.00000	0.00433	0.00277	0.00411	0.00376	0.00467	0.00280
P07214	SPRC_MOUSE	0.00000	0.00000	0.00000	0.00000	0.00000	0.00000	0.00000	0.00116	0.00443	0.00185	0.00103	0.00087
P07309	TTHY_MOUSE	0.03801	0.00969	0.02862	0.02693	0.02284	0.00587	0.05229	0.04371	0.08150	0.08400	0.06131	0.06318
P07310	KCRM_MOUSE	1.32109	1.22987	1.04328	0.96496	1.34152	1.30737	0.44614	0.03387	0.03961	0.03779	0.02753	0.02770
P07356	ANXA2_MOUSE	0.03968	0.13015	0.05220	0.08262	0.06282	0.10785	0.23482	0.23635	0.21510	0.21687	0.22776	0.21760
P07724	ALBU_MOUSE	4.12579	3.07294	3.60903	6.13628	5.06740	3.60449	1.21023	1.47544	1.81558	2.17664	1.76166	1.60203
P07759	SPA3K_MOUSE	0.01115	0.00276	0.00552	0.01370	0.00266	0.00278	0.68512	0.51680	0.50382	0.49478	0.65322	0.37265
P07901	HS90A_MOUSE	0.03626	0.03026	0.04117	0.03005	0.03102	0.02272	0.12413	0.12915	0.14984	0.13380	0.13189	0.14299
P08003	PDIA4_MOUSE	0.00670	0.00348	0.00484	0.00408	0.00376	0.00462	0.04714	0.04480	0.05083	0.05260	0.03800	0.05384
P08030	APT_MOUSE	0.00000	0.00000	0.00000	0.00000	0.00000	0.00000	0.00000	0.00484	0.00785	0.00611	0.00772	0.00718
P08032	SPTA1_MOUSE	0.00860	0.01318	0.01073	0.01343	0.01188	0.01083	0.01737	0.00174	0.00623	0.03348	0.06303	0.00152
P08071	TRFL_MOUSE	0.00000	0.00000	0.00000	0.00000	0.00000	0.00000	0.01290	0.01296	0.00681	0.00773	0.01854	0.01858
P08074	CBR2_MOUSE	0.00000	0.00000	0.00000	0.00000	0.00000	0.00000	1.07335	1.24528	0.48623	0.63255	0.47685	0.81269
P08103	HCK_MOUSE	0.00000	0.00000	0.00000	0.00000	0.00000	0.00000	0.00860	0.00972	0.00634	0.00620	0.00668	0.00903
P08113	ENPL_MOUSE	0.02288	0.01460	0.02873	0.02307	0.02183	0.02559	0.08119	0.07668	0.10460	0.09018	0.08206	0.08473
P08121	CO3A1_MOUSE	0.00000	0.00000	0.00000	0.00000	0.00000	0.00000	0.00000	0.00191	0.00000	0.00000	0.00085	0.00000
P08122	CO4A2_MOUSE	0.00370	0.00578	0.00407	0.00401	0.00193	0.00085	0.15555	0.16254	0.17958	0.09986	0.24488	0.16432
P08207	S10AA_MOUSE	0.00000	0.00000	0.00000	0.00000	0.00000	0.00000	0.05186	0.01713	0.05795	0.03420	0.05224	0.02076
P08226	APOE_MOUSE	0.01120	0.00605	0.00900	0.01068	0.00977	0.00574	0.01284	0.01230	0.01641	0.01630	0.01861	0.01109
P08228	SODC_MOUSE	0.00000	0.00000	0.00000	0.00000	0.00000	0.00000	0.14308	0.14138	0.18840	0.14905	0.17937	0.13314
P08249	MDHM_MOUSE	1.18973	1.01241	0.99698	0.99531	1.06942	1.52054	0.24697	0.23673	0.19283	0.17594	0.13541	0.16922
P08551	NFL_MOUSE	0.00000	0.00000	0.00000	0.00000	0.00000	0.00000	0.00000	0.00509	0.00372	0.00000	0.00000	0.00000
P08553	NFM_MOUSE	0.00000	0.00000	0.00000	0.00000	0.00000	0.00000	0.00227	0.00037	0.00766	0.00000	0.00041	0.00000
P08556	RASN_MOUSE	0.00374	0.00022	0.00231	0.00146	0.00065	0.00084	0.00368	0.01327	0.00382	0.00471	0.00296	0.00548
P08752	GNAI2_MOUSE	0.04228	0.04325	0.03726	0.03708	0.03245	0.03988	0.10940	0.15398	0.09781	0.11688	0.09355	0.14670
P08905	LYZ2_MOUSE	0.00000	0.00000	0.00000	0.00000	0.00000	0.00000	0.04733	0.04046	0.01874	0.02234	0.00440	0.03371
P09041	PGK2_MOUSE	0.00000	0.00000	0.00000	0.00000	0.00000	0.00000	0.00000	0.00000	0.00056	0.00051	0.00064	0.00000
P09055	ITB1_MOUSE	0.00051	0.00074	0.00063	0.00081	0.00073	0.00036	0.05962	0.07923	0.06221	0.06126	0.05738	0.07845
P09103	PDIA1_MOUSE	0.04023	0.03091	0.03357	0.03030	0.03666	0.03099	0.13053	0.12926	0.15722	0.15106	0.14043	0.12630

P09242	PPBT_MOUSE	0.00000	0.00000	0.00000	0.00000	0.00000	0.00000	0.00223	0.00217	0.00212	0.00281	0.00315	0.00248
P09405	NUCL_MOUSE	0.00538	0.01384	0.00668	0.00288	0.00753	0.01280	0.04277	0.03581	0.03667	0.03637	0.03757	0.04180
P09411	PGK1_MOUSE	0.21387	0.18441	0.31293	0.27560	0.24574	0.24294	0.11239	0.08664	0.09043	0.08615	0.09583	0.10210
P09470	ACE_MOUSE	0.00000	0.00000	0.00000	0.00000	0.00000	0.00000	0.05966	0.07373	0.05834	0.06152	0.06828	0.06310
P09528	FRIH_MOUSE	0.00000	0.00000	0.00000	0.00000	0.00000	0.00000	0.00238	0.00957	0.02112	0.01464	0.01101	0.01194
P09541	MYL4_MOUSE	0.01227	0.01226	0.00900	0.01322	0.01804	0.02203	0.09240	0.15138	0.09084	0.15556	0.10331	0.10155
P09542	MYL3_MOUSE	1.17670	0.49711	0.85716	0.81888	0.83030	0.51797	0.33958	0.00687	0.00969	0.01536	0.00403	0.00367
P09671	SODM_MOUSE	0.15930	0.18698	0.21283	0.14269	0.10924	0.17969	0.00586	0.04199	0.03039	0.02881	0.02366	0.01814
P09803	CADH1_MOUSE	0.00000	0.00000	0.00000	0.00000	0.00000	0.00000	0.02632	0.02904	0.03380	0.03005	0.04149	0.04020
P09813	APOA2_MOUSE	0.00000	0.00000	0.00000	0.00000	0.00000	0.00000	0.00000	0.00486	0.01448	0.03825	0.01970	0.00647
P09925	SURF1_MOUSE	0.00163	0.00073	0.00336	0.00135	0.00214	0.00146	0.00000	0.00000	0.00000	0.00000	0.00000	0.00000
P0C0A3	CHMP6_MOUSE	0.00000	0.00000	0.00000	0.00000	0.00000	0.00000	0.00000	0.00991	0.00979	0.01280	0.00569	0.00994
Q3THW5	H2AV_MOUSE	0.00000	0.00000	0.00000	0.00000	0.00000	0.00000	0.01674	0.02444	0.02263	0.01541	0.01835	0.01487
P0C605	KGP1_MOUSE	0.00000	0.00000	0.00000	0.00000	0.00000	0.00000	0.00742	0.00728	0.01072	0.00856	0.00952	0.00761
P0C8K7	SMIM1_MOUSE	0.00000	0.00000	0.00000	0.00000	0.00000	0.00000	0.00000	0.00357	0.00428	0.00346	0.00247	0.00168
P62984	RL40_MOUSE	0.00000	0.00000	0.00000	0.00000	0.00000	0.00000	0.21803	0.50992	0.43333	0.47672	0.21174	0.36201
P0DN34	NDUB1_MOUSE	0.00000	0.00000	0.00000	0.00000	0.00000	0.00000	0.00566	0.01051	0.00716	0.00765	0.00436	0.00339
P0DN91	T254C_MOUSE	0.00000	0.00000	0.00000	0.00000	0.00000	0.00000	0.00000	0.00253	0.00000	0.00000	0.00000	0.00106
P0DOV1	IFI5B_MOUSE	0.00000	0.00000	0.00000	0.00000	0.00000	0.00000	0.00000	0.00000	0.00110	0.00024	0.00055	0.00031
P0DP28	CALM3_MOUSE	0.00000	0.00000	0.00000	0.00000	0.00000	0.00000	0.48969	0.46023	0.49756	0.44673	0.55582	0.44629
P10107	ANXA1_MOUSE	0.00452	0.00357	0.00361	0.00522	0.00509	0.00577	0.19762	0.20244	0.25699	0.25505	0.28781	0.25427
P10126	EF1A1_MOUSE	0.00933	0.00962	0.01107	0.01967	0.00864	0.01145	0.13781	0.25513	0.16343	0.19346	0.16725	0.18543
P10417	BCL2_MOUSE	0.00000	0.00000	0.00000	0.00000	0.00000	0.00000	0.00000	0.00089	0.00060	0.00125	0.00176	0.00144
P10493	NID1_MOUSE	0.04544	0.03477	0.02173	0.03247	0.05569	0.02378	0.07215	0.08214	0.06785	0.06327	0.06457	0.07171
P10518	HEM2_MOUSE	0.00817	0.00460	0.01250	0.02784	0.01913	0.01425	0.00875	0.01620	0.01956	0.01729	0.01520	0.01149
P10605	CATB_MOUSE	0.00832	0.00350	0.01104	0.00677	0.00956	0.00530	0.01189	0.00703	0.00793	0.00502	0.00616	0.00430
P10630	IF4A2_MOUSE	0.00176	0.00367	0.00159	0.00473	0.00141	0.00344	0.00326	0.02637	0.02470	0.01722	0.02224	0.01531
P10637	TAU_MOUSE	0.00000	0.00000	0.00000	0.00000	0.00000	0.00000	0.01330	0.02637	0.02339	0.02317	0.01916	0.02338
P10639	THIO_MOUSE	0.00000	0.00000	0.00000	0.00000	0.00000	0.00000	0.01277	0.06796	0.02053	0.02983	0.00946	0.01320
P10649	GSTM1_MOUSE	0.12071	0.10947	0.09035	0.06401	0.10555	0.10762	0.23240	0.51344	0.32307	0.36821	0.30363	0.37539
P10711	TCEA1_MOUSE	0.00000	0.00000	0.00000	0.00000	0.00000	0.00000	0.00142	0.00284	0.00469	0.00462	0.00272	0.00542
P10810	CD14_MOUSE	0.00000	0.00000	0.00000	0.00000	0.00000	0.00000	0.00160	0.00418	0.00677	0.00000	0.00186	0.00415
P10833	RRAS_MOUSE	0.00000	0.00000	0.00000	0.00000	0.00000	0.00000	0.09455	0.11689	0.12750	0.13365	0.14236	0.13690
P10852	4F2_MOUSE	0.00000	0.00000	0.00000	0.00000	0.00000	0.00000	0.00482	0.00506	0.00606	0.00531	0.00516	0.00626

Q8CGP2	H2B1P_MOUSE	0.00000	0.00000	0.00000	0.00000	0.00000	0.00000	0.88333	1.66691	1.45789	1.38032	1.22383	1.70555
P10922	H10_MOUSE	0.00000	0.00000	0.00000	0.00000	0.00000	0.00000	0.12021	0.19756	0.16211	0.17325	0.09975	0.18898
P11031	TCP4_MOUSE	0.00000	0.00000	0.00000	0.00000	0.00000	0.00000	0.00840	0.01244	0.01139	0.01113	0.01640	0.01511
P21812	MCPT4_MOUSE	0.00000	0.00000	0.00000	0.00000	0.00000	0.00000	0.00000	0.00000	0.00329	0.00000	0.00000	0.00000
P11087	CO1A1_MOUSE	0.00000	0.00000	0.00000	0.00000	0.00000	0.00000	0.20184	0.35953	0.23389	0.15119	0.34391	0.18047
P11103	PARP1_MOUSE	0.00000	0.00000	0.00000	0.00000	0.00000	0.00000	0.01064	0.01217	0.00919	0.01406	0.00000	0.01292
P11152	LIPL_MOUSE	0.01794	0.02977	0.02388	0.01827	0.02802	0.01807	0.00211	0.00089	0.00316	0.00160	0.00252	0.00239
P11247	PERM_MOUSE	0.00000	0.00000	0.00000	0.00000	0.00000	0.00000	0.01095	0.00808	0.00456	0.00721	0.01358	0.01585
P11276	FINC_MOUSE	0.01190	0.00566	0.00662	0.01818	0.00694	0.00832	0.08449	0.05123	0.05275	0.04942	0.06830	0.05080
P11352	GPX1_MOUSE	0.00000	0.00000	0.00000	0.00000	0.00000	0.00000	0.04418	0.03629	0.04181	0.04523	0.04911	0.04623
P11404	FABPH_MOUSE	0.05203	0.00839	0.03570	0.03605	0.01708	0.00186	0.00000	0.00000	0.00000	0.00738	0.00000	0.00000
P11438	LAMP1_MOUSE	0.00000	0.00000	0.00000	0.00000	0.00000	0.00000	0.00000	0.02112	0.02026	0.01898	0.01128	0.00829
P11499	HS90B_MOUSE	0.24017	0.18022	0.22954	0.17518	0.13916	0.13343	0.21958	0.19262	0.23772	0.22665	0.23645	0.20583
P11531	DMD_MOUSE	0.11310	0.12733	0.11908	0.10112	0.11873	0.09586	0.01016	0.01045	0.00784	0.00651	0.01228	0.00895
P11589	MUP2_MOUSE	0.00000	0.00000	0.00000	0.00000	0.00000	0.00000	0.00021	0.00000	0.00000	0.00000	0.00011	0.00040
P11672	NGAL_MOUSE	0.00000	0.00000	0.00000	0.00000	0.00000	0.00000	0.00109	0.00841	0.00504	0.00392	0.00213	0.00895
P11679	K2C8_MOUSE	0.00000	0.00000	0.00000	0.00000	0.00000	0.00000	0.11835	0.15729	0.12468	0.15556	0.11802	0.14233
P11688	ITA5_MOUSE	0.00000	0.00000	0.00000	0.00000	0.00000	0.00000	0.00771	0.00361	0.01102	0.00526	0.00387	0.00564
P11714	CP2D9_MOUSE	0.00000	0.00000	0.00000	0.00000	0.00000	0.00000	0.00000	0.00000	0.00000	0.00000	0.00000	0.00000
P11835	ITB2_MOUSE	0.00000	0.00000	0.00000	0.00000	0.00000	0.00000	0.00943	0.00981	0.01420	0.01311	0.01524	0.01529
P11862	GAS2_MOUSE	0.00000	0.00000	0.00000	0.00000	0.00000	0.00000	0.00004	0.00000	0.00005	0.00015	0.00007	0.00000
P11881	ITPR1_MOUSE	0.00000	0.00000	0.00000	0.00000	0.00000	0.00000	0.00877	0.00609	0.00621	0.00702	0.00000	0.00527
P11911	CD79A_MOUSE	0.00000	0.00000	0.00000	0.00000	0.00000	0.00000	0.00000	0.00000	0.00000	0.00000	0.00000	0.00240
P11928	OAS1A_MOUSE	0.00000	0.00000	0.00000	0.00000	0.00000	0.00000	0.00000	0.00000	0.00433	0.00066	0.00278	0.00183
P11930	NUD19_MOUSE	0.00000	0.00000	0.00000	0.00000	0.00000	0.00000	0.00000	0.00004	0.00033	0.00010	0.00000	0.00000
P11983	TCPA_MOUSE	0.00227	0.00403	0.00187	0.00105	0.00209	0.00180	0.02691	0.02091	0.03227	0.02583	0.02813	0.02705
P12023	A4_MOUSE	0.00000	0.00000	0.00000	0.00000	0.00000	0.00000	0.00796	0.00796	0.01117	0.01017	0.00643	0.01143
P12242	UCP1_MOUSE	0.00000	0.00000	0.00000	0.00000	0.00000	0.00000	0.02884	0.00000	0.00000	0.00000	0.00000	0.00114
P12265	BGLR_MOUSE	0.00000	0.00000	0.00000	0.00000	0.00000	0.00000	0.00144	0.00477	0.00777	0.00343	0.00662	0.00255
P12367	KAP2_MOUSE	0.00577	0.00547	0.00532	0.00564	0.00551	0.00727	0.04186	0.09209	0.05014	0.04938	0.05193	0.05994
P12382	PFKAL_MOUSE	0.00913	0.01063	0.01125	0.01194	0.00984	0.00578	0.01484	0.01095	0.01568	0.01131	0.01585	0.01163
P12658	CALB1_MOUSE	0.00000	0.00000	0.00000	0.00000	0.00000	0.00000	0.00055	0.00005	0.00039	0.00041	0.00055	0.00000
P12710	FABPL_MOUSE	0.00000	0.00000	0.00000	0.00000	0.00000	0.00000	0.00567	0.00537	0.00585	0.00457	0.00503	0.00577
P12787	COX5A_MOUSE	0.06655	0.04123	0.07966	0.11091	0.11464	0.18349	0.03321	0.02879	0.02386	0.02907	0.02140	0.02605

P12791	CP2BA_MOUSE	0.00000	0.00000	0.00000	0.00000	0.00000	0.00000	0.04058	0.03853	0.03297	0.04171	0.04256	0.04341
P12815	PDCD6_MOUSE	0.00000	0.00000	0.00000	0.00000	0.00000	0.00000	0.01417	0.01492	0.01420	0.01249	0.01461	0.01311
P12970	RL7A_MOUSE	0.01609	0.02892	0.01885	0.01117	0.01717	0.08144	0.02386	0.02964	0.03977	0.02860	0.02774	0.03851
Q6T707	SCD4_MOUSE	0.00000	0.00000	0.00000	0.00000	0.00000	0.00000	0.00000	0.00000	0.00000	0.00000	0.00000	0.00000
P13020	GELS_MOUSE	0.01912	0.01646	0.00830	0.01487	0.01063	0.01394	0.11610	0.10489	0.13176	0.12746	0.12365	0.10289
P13412	TNNI2_MOUSE	0.00000	0.00000	0.00000	0.00000	0.00000	0.00000	0.08391	0.00000	0.00005	0.00000	0.00000	0.00000
P13541	MYH3_MOUSE	0.00266	0.00261	0.00392	0.00365	0.00245	0.00271	0.05661	0.05426	0.00000	0.06067	0.01522	0.01459
P13542	MYH8_MOUSE	0.00022	0.00130	0.00037	0.00484	0.00033	0.00042	0.35381	0.00003	0.00004	0.00003	0.00002	0.00002
P13597	ICAM1_MOUSE	0.00000	0.00000	0.00000	0.00000	0.00000	0.00000	0.03843	0.06064	0.07130	0.06923	0.05680	0.07064
P13609	SRGN_MOUSE	0.00000	0.00000	0.00000	0.00000	0.00000	0.00000	0.00000	0.00051	0.00000	0.00316	0.00000	0.00348
P13634	CAH1_MOUSE	0.00000	0.00000	0.00000	0.00000	0.00000	0.00000	0.03105	0.01051	0.01293	0.03966	0.06161	0.00647
P13707	GPDA_MOUSE	0.01054	0.00458	0.00727	0.00728	0.00610	0.00652	0.03198	0.01034	0.01832	0.00631	0.00222	0.00984
P13745	GSTA1_MOUSE	0.00004	0.00060	0.00045	0.00027	0.00070	0.00037	0.00000	0.00473	0.00173	0.00193	0.00178	0.01622
P13864	DNMT1_MOUSE	0.00000	0.00000	0.00000	0.00000	0.00000	0.00000	0.01194	0.00101	0.00498	0.00920	0.02161	0.00478
P14069	S10A6_MOUSE	0.00000	0.00000	0.00000	0.00000	0.00000	0.00000	0.01169	0.13819	0.11928	0.16225	0.05787	0.05768
P14094	AT1B1_MOUSE	0.06357	0.06780	0.03703	0.02930	0.05461	0.08222	0.02324	0.01779	0.01585	0.01730	0.00564	0.01788
P14115	RL27A_MOUSE	0.00000	0.00000	0.00000	0.00000	0.00000	0.00000	0.00791	0.02374	0.00890	0.01728	0.01017	0.02292
P14131	RS16_MOUSE	0.00000	0.00000	0.00000	0.00000	0.00000	0.00000	0.05539	0.04264	0.05798	0.06428	0.05857	0.07179
P14148	RL7_MOUSE	0.00000	0.00000	0.00000	0.00000	0.00000	0.00000	0.04874	0.05153	0.06638	0.05707	0.04704	0.06367
P14152	MDHC_MOUSE	0.88852	1.05491	1.15342	0.95855	0.97203	1.09287	0.12869	0.15515	0.10146	0.13107	0.11271	0.12932
P14206	RSSA_MOUSE	0.05046	0.03567	0.03481	0.02341	0.03983	0.03214	0.04465	0.02824	0.02758	0.03043	0.03343	0.02974
P14211	CALR_MOUSE	0.00000	0.00000	0.00000	0.00000	0.00000	0.00000	0.06669	0.09152	0.09538	0.09583	0.09962	0.08794
P14220	GLPA_MOUSE	0.00000	0.00000	0.00000	0.00000	0.00000	0.00000	0.00000	0.00000	0.00000	0.00052	0.00000	0.00000
P14246	GTR2_MOUSE	0.00000	0.00000	0.00000	0.00000	0.00000	0.00000	0.00000	0.00000	0.00000	0.00000	0.00000	0.00000
P14426	HA13_MOUSE	0.00000	0.00000	0.00000	0.00000	0.00000	0.00000	0.00510	0.00000	0.00289	0.00471	0.00000	0.00651
P14429	HA17_MOUSE	0.00000	0.00000	0.00000	0.00000	0.00000	0.00000	0.00000	0.00276	0.00198	0.00146	0.00389	0.00434
P14438	HA2U_MOUSE	0.00000	0.00000	0.00000	0.00000	0.00000	0.00000	0.00730	0.00841	0.01779	0.01470	0.01061	0.01154
P14483	HB2A_MOUSE	0.00000	0.00000	0.00000	0.00000	0.00000	0.00000	0.00025	0.00862	0.00980	0.00660	0.00215	0.00736
P14576	SRP54_MOUSE	0.00000	0.00000	0.00000	0.00000	0.00000	0.00000	0.00489	0.00746	0.00986	0.00698	0.00979	0.00658
P14602	HSPB1_MOUSE	0.03024	0.01695	0.02417	0.01153	0.01509	0.02553	0.07327	0.07046	0.05943	0.04949	0.04146	0.05428
P14685	PSMD3_MOUSE	0.00153	0.00414	0.00386	0.00232	0.00223	0.00109	0.01430	0.01317	0.01696	0.01528	0.01922	0.01649
P14733	LMNB1_MOUSE	0.00831	0.00351	0.00386	0.00490	0.00927	0.01011	0.04390	0.05457	0.07052	0.06040	0.05840	0.07125
P14824	ANXA6_MOUSE	0.21169	0.17197	0.21012	0.15656	0.29940	0.16945	0.19330	0.17054	0.19558	0.17313	0.23618	0.20616
P14869	RLA0_MOUSE	0.00157	0.00091	0.00228	0.00168	0.00327	0.00089	0.11870	0.07449	0.10517	0.09397	0.09516	0.09658

P14873	MAP1B_MOUSE	0.00000	0.00000	0.00000	0.00000	0.00000	0.00000	0.00865	0.00444	0.01330	0.00997	0.00982	0.00317
P15089	CBPA3_MOUSE	0.00000	0.00000	0.00000	0.00000	0.00000	0.00000	0.00932	0.00000	0.01190	0.00061	0.00069	0.00000
P15105	GLNA_MOUSE	0.01083	0.02067	0.01139	0.00993	0.00936	0.00677	0.00949	0.01039	0.01346	0.00786	0.00843	0.00925
P15306	TRBM_MOUSE	0.00000	0.00000	0.00000	0.00000	0.00000	0.00000	0.00000	0.02945	0.03180	0.02268	0.01261	0.01258
P15327	PMGE_MOUSE	0.00000	0.00000	0.00000	0.00000	0.00000	0.00000	0.00481	0.00246	0.00363	0.01510	0.01787	0.00023
P15379	CD44_MOUSE	0.00000	0.00000	0.00000	0.00000	0.00000	0.00000	0.01981	0.02157	0.01432	0.01404	0.01475	0.01762
P15392	CP2A4_MOUSE	0.00000	0.00000	0.00000	0.00000	0.00000	0.00000	0.00483	0.00057	0.00162	0.00225	0.00103	0.00131
P15501	SPBP_MOUSE	0.00000	0.00000	0.00000	0.00000	0.00000	0.00000	0.00000	0.00000	0.00000	0.00017	0.00036	0.00000
P15508	SPTB1_MOUSE	0.04989	0.05134	0.02673	0.05198	0.04818	0.03963	0.02424	0.00633	0.00734	0.01959	0.03550	0.00396
P15532	NDKA_MOUSE	0.00243	0.00184	0.00194	0.00226	0.00233	0.00130	0.01135	0.01581	0.01606	0.01474	0.00732	0.01398
P15626	GSTM2_MOUSE	0.05342	0.06089	0.04950	0.04346	0.04594	0.07010	0.10865	0.15965	0.11480	0.13317	0.11991	0.12714
P15864	H12_MOUSE	0.00000	0.00000	0.00000	0.00000	0.00000	0.00000	0.10040	0.32003	0.21906	0.37256	0.20845	0.35520
P16015	CAH3_MOUSE	0.00074	0.00164	0.00236	0.00183	0.00073	0.00167	0.31018	0.01961	0.18612	0.03238	0.03476	0.02380
P16045	LEG1_MOUSE	0.01767	0.00689	0.01338	0.01571	0.01068	0.00764	0.04014	0.03126	0.02688	0.03809	0.01909	0.03581
P16054	KPCE_MOUSE	0.00000	0.00000	0.00000	0.00000	0.00000	0.00000	0.00000	0.00587	0.00681	0.00000	0.00819	0.00000
P16110	LEG3_MOUSE	0.00000	0.00000	0.00000	0.00000	0.00000	0.00000	0.03345	0.04898	0.07154	0.05938	0.03365	0.03905
P16125	LDHB_MOUSE	0.50311	0.75288	0.73569	0.48289	0.63698	0.88971	0.07978	0.10174	0.08040	0.07923	0.06269	0.07455
P16254	SRP14_MOUSE	0.00000	0.00000	0.00000	0.00000	0.00000	0.00000	0.00759	0.00201	0.00651	0.00521	0.00728	0.00742
P16283	B3A3_MOUSE	0.00000	0.00000	0.00000	0.00000	0.00000	0.00000	0.00000	0.00234	0.00259	0.00250	0.00138	0.00124
P16330	CN37_MOUSE	0.00000	0.00000	0.00000	0.00000	0.00000	0.00000	0.00000	0.00566	0.00776	0.00000	0.00518	0.00208
P16331	PH4H_MOUSE	0.00000	0.00000	0.00000	0.00000	0.00000	0.00000	0.00000	0.00022	0.00019	0.00000	0.00000	0.00000
P16332	MUTA_MOUSE	0.03269	0.02449	0.02476	0.02240	0.02706	0.03114	0.00751	0.01381	0.00908	0.00955	0.00707	0.00846
P16406	AMPE_MOUSE	0.00000	0.00000	0.00000	0.00000	0.00000	0.00000	0.04307	0.05017	0.05095	0.04872	0.05356	0.05727
P16460	ASSY_MOUSE	0.00000	0.00000	0.00000	0.00000	0.00000	0.00000	0.00062	0.00570	0.00600	0.00357	0.00699	0.00446
P16546	SPTN1_MOUSE	0.20417	0.12728	0.17457	0.17758	0.15946	0.13867	0.11687	0.13151	0.17854	0.14207	0.15623	0.15012
P16675	PPGB_MOUSE	0.00000	0.00000	0.00000	0.00000	0.00000	0.00000	0.00320	0.01266	0.01088	0.00897	0.01087	0.00757
P16858	G3P_MOUSE	0.98224	0.51869	1.01981	0.72860	0.78858	0.80090	0.56288	0.36825	0.26070	0.35137	0.25157	0.36877
P17047	LAMP2_MOUSE	0.00000	0.00000	0.00000	0.00000	0.00000	0.00000	0.01423	0.00880	0.01141	0.01020	0.00962	0.00916
P17095	HMGA1_MOUSE	0.00000	0.00000	0.00000	0.00000	0.00000	0.00000	0.00000	0.00475	0.01254	0.00321	0.00524	0.00382
P17156	HSP72_MOUSE	0.00174	0.00159	0.00116	0.00169	0.00127	0.00206	0.00928	0.02057	0.01681	0.01947	0.01767	0.02012
P17182	ENOA_MOUSE	0.01978	0.01074	0.02291	0.01871	0.01169	0.01796	0.24492	0.22841	0.25146	0.22383	0.14674	0.24705
P17225	PTBP1_MOUSE	0.00000	0.00000	0.00000	0.00000	0.00000	0.00000	0.02836	0.02911	0.03491	0.03486	0.05349	0.05020
P17426	AP2A1_MOUSE	0.00318	0.00278	0.00247	0.00558	0.00239	0.00226	0.02605	0.01886	0.02001	0.01972	0.02271	0.02178
P17427	AP2A2_MOUSE	0.00246	0.00341	0.00061	0.00193	0.00128	0.00240	0.02469	0.02629	0.02828	0.02327	0.02374	0.03292

P17563	SBP1_MOUSE	0.17766	0.18321	0.14017	0.12865	0.14143	0.19348	0.49841	0.60360	0.41273	0.42781	0.51318	0.55056
P17710	HXK1_MOUSE	0.01418	0.02208	0.01823	0.02075	0.01743	0.02101	0.02334	0.02683	0.01946	0.02161	0.02164	0.02025
P17717	UDB17_MOUSE	0.00000	0.00000	0.00000	0.00000	0.00000	0.00000	0.00000	0.00000	0.00000	0.00000	0.00000	0.00000
P17742	PPIA_MOUSE	0.02645	0.02491	0.03541	0.02842	0.02695	0.03856	0.35395	0.35896	0.25657	0.31834	0.23481	0.37145
P17751	TPIS_MOUSE	0.10177	0.21466	0.27082	0.26688	0.23720	0.29151	0.08246	0.08808	0.09662	0.09369	0.07877	0.08996
P17809	GTR1_MOUSE	0.00000	0.00000	0.00000	0.00000	0.00000	0.00000	0.00052	0.00059	0.00164	0.00044	0.00125	0.00086
Q61696	HS71A_MOUSE	0.00347	0.00298	0.00224	0.00348	0.00148	0.00239	0.02606	0.03407	0.02715	0.01684	0.01333	0.02009
P17918	PCNA_MOUSE	0.00000	0.00000	0.00000	0.00000	0.00000	0.00000	0.00670	0.01020	0.00417	0.00628	0.00179	0.01073
P18052	PTPRA_MOUSE	0.00000	0.00000	0.00000	0.00000	0.00000	0.00000	0.00254	0.00149	0.00289	0.00000	0.00093	0.00497
P18242	CATD_MOUSE	0.02036	0.02472	0.02485	0.02895	0.04263	0.02615	0.04137	0.03269	0.04375	0.04387	0.02799	0.03262
P18419	SVS4_MOUSE	0.00000	0.00013	0.00084	0.11426	0.00000	0.00000	0.00000	0.00000	0.00000	0.00000	0.00000	0.00000
P18525	HVM54_MOUSE	0.00000	0.00000	0.00000	0.00000	0.00000	0.00000	0.00000	0.00453	0.00000	0.00000	0.00000	0.00000
P18572	BASI_MOUSE	0.07178	0.06416	0.05445	0.07686	0.04433	0.06950	0.00673	0.00559	0.01061	0.01137	0.00976	0.00631
P18608	HMGN1_MOUSE	0.00000	0.00000	0.00000	0.00000	0.00000	0.00000	0.00000	0.00413	0.00041	0.00294	0.00000	0.00101
P18653	KS6A1_MOUSE	0.00000	0.00000	0.00000	0.00000	0.00000	0.00000	0.01667	0.01235	0.02001	0.01454	0.02621	0.01799
P18654	KS6A3_MOUSE	0.00000	0.00000	0.00000	0.00000	0.00000	0.00000	0.00235	0.00443	0.00645	0.00519	0.00805	0.00629
P18760	COF1_MOUSE	0.00000	0.00000	0.00000	0.00000	0.00000	0.00000	0.31805	0.34702	0.32620	0.42269	0.37688	0.46048
P18828	SDC1_MOUSE	0.00000	0.00000	0.00000	0.00000	0.00000	0.00000	0.00000	0.01238	0.01296	0.00661	0.00853	0.01095
P18872	GNAO_MOUSE	0.00000	0.00000	0.00000	0.00000	0.00000	0.00000	0.03070	0.04407	0.03707	0.03211	0.01791	0.03416
P18894	OXDA_MOUSE	0.00000	0.00000	0.00000	0.00000	0.00000	0.00000	0.00000	0.00000	0.00000	0.00000	0.00000	0.00000
P19096	FAS_MOUSE	0.00000	0.00000	0.00000	0.00000	0.00000	0.00000	0.12149	0.05183	0.11041	0.06197	0.05541	0.04909
P19123	TNNC1_MOUSE	0.46473	0.43925	0.42772	0.53134	0.54139	0.53629	0.01384	0.02509	0.02645	0.02725	0.01647	0.01428
P19137	LAMA1_MOUSE	0.00000	0.00000	0.00000	0.00000	0.00000	0.00000	0.00000	0.03273	0.01226	0.01446	0.00000	0.01045
P19157	GSTP1_MOUSE	0.16160	0.14319	0.09580	0.10885	0.13759	0.13195	0.21922	0.26440	0.22580	0.25647	0.21155	0.26371
P19221	THRB_MOUSE	0.00666	0.00576	0.00361	0.00726	0.00592	0.00501	0.00882	0.00823	0.00822	0.01421	0.01059	0.00554
P19246	NFH_MOUSE	0.00000	0.00000	0.00000	0.00000	0.00000	0.00000	0.00000	0.00000	0.00329	0.00000	0.00000	0.00000
P19253	RL13A_MOUSE	0.00000	0.00000	0.00000	0.00000	0.00000	0.00000	0.00306	0.01883	0.03040	0.03481	0.01557	0.02150
P19258	MPV17_MOUSE	0.00000	0.00000	0.00000	0.00000	0.00000	0.00000	0.00000	0.00080	0.00000	0.00000	0.00000	0.00000
P19324	SERPH_MOUSE	0.03483	0.01711	0.01694	0.01912	0.02465	0.02080	0.08644	0.06084	0.09407	0.07985	0.08933	0.06661
P19536	COX5B_MOUSE	0.00164	0.00049	0.00210	0.00353	0.00151	0.00103	0.00000	0.00937	0.00974	0.01718	0.00000	0.00000
P19783	COX41_MOUSE	0.21569	0.12661	0.17647	0.21269	0.24208	0.25605	0.08387	0.09390	0.06280	0.07141	0.05957	0.04965
P19788	MGP_MOUSE	0.00000	0.00000	0.00000	0.00000	0.00000	0.00000	0.00000	0.00263	0.00000	0.00000	0.00527	0.00000
P19973	LSP1_MOUSE	0.00000	0.00000	0.00000	0.00000	0.00000	0.00000	0.00468	0.02054	0.02593	0.02255	0.01582	0.02195
P20029	BIP_MOUSE	0.18281	0.16560	0.25088	0.16474	0.16070	0.14036	0.29006	0.28239	0.29490	0.31842	0.25533	0.30356

P20108	PRDX3_MOUSE	0.42512	0.16291	0.28848	0.22482	0.25955	0.20144	0.03280	0.03346	0.03351	0.03310	0.03218	0.03004
P20152	VIME_MOUSE	0.10000	0.08798	0.07911	0.09626	0.09549	0.13471	0.62095	0.68997	0.76492	0.71236	0.64174	0.65186
P20444	KPCA_MOUSE	0.00000	0.00000	0.00000	0.00000	0.00000	0.00000	0.01423	0.01453	0.00790	0.01248	0.01030	0.01017
P20801	TNNC2_MOUSE	0.00000	0.00000	0.00000	0.00000	0.00000	0.00000	0.11612	0.00016	0.00000	0.00013	0.00004	0.00006
P20918	PLMN_MOUSE	0.00764	0.00397	0.00480	0.00646	0.00505	0.00405	0.00211	0.01137	0.00696	0.01243	0.00543	0.00395
P21107	TPM3_MOUSE	0.00000	0.00000	0.00000	0.00000	0.00000	0.00000	0.11140	0.12504	0.10378	0.13053	0.10860	0.11830
P21271	MYO5B_MOUSE	0.00000	0.00000	0.00000	0.00000	0.00000	0.00000	0.00218	0.00305	0.00409	0.00369	0.00129	0.00257
P21278	GNA11_MOUSE	0.00000	0.00000	0.00000	0.00000	0.00000	0.00000	0.00792	0.01256	0.01794	0.01330	0.01631	0.01510
P21279	GNAQ_MOUSE	0.00000	0.00000	0.00000	0.00000	0.00000	0.00000	0.04027	0.04067	0.02629	0.03827	0.02534	0.04417
P21300	ALD1_MOUSE	0.00000	0.00000	0.00000	0.00000	0.00000	0.00000	0.01351	0.00994	0.01502	0.01210	0.01429	0.00534
P21447	MDR1A_MOUSE	0.00000	0.00000	0.00000	0.00000	0.00000	0.00000	0.01322	0.01645	0.01752	0.01503	0.01412	0.01663
P21550	ENOB_MOUSE	0.12799	0.11210	0.32369	0.21351	0.17725	0.22681	0.09856	0.02329	0.02929	0.03030	0.01250	0.01387
P21614	VTDB_MOUSE	0.02039	0.00474	0.01020	0.02125	0.01418	0.00866	0.01613	0.01777	0.02354	0.02453	0.03166	0.01118
P21619	LMNB2_MOUSE	0.00289	0.00093	0.00314	0.00124	0.00086	0.00171	0.02217	0.03955	0.04152	0.03397	0.03177	0.04262
P21841	PSPC_MOUSE	0.00000	0.00000	0.00000	0.00000	0.00000	0.00000	0.03279	0.03519	0.05586	0.05638	0.03695	0.03074
P21844	CMA1_MOUSE	0.00000	0.00000	0.00000	0.00000	0.00000	0.00000	0.00000	0.00000	0.00293	0.00000	0.00000	0.00000
P21956	MFGM_MOUSE	0.00000	0.00000	0.00000	0.00000	0.00000	0.00000	0.00672	0.01588	0.01210	0.00723	0.00779	0.00916
P21958	TAP1_MOUSE	0.00000	0.00000	0.00000	0.00000	0.00000	0.00000	0.00783	0.00904	0.00823	0.00874	0.00864	0.01396
P21981	TGM2_MOUSE	0.05742	0.03813	0.03922	0.03250	0.03268	0.03660	0.18180	0.15857	0.16415	0.14752	0.14940	0.18975
P21995	EMB_MOUSE	0.00000	0.00000	0.00000	0.00000	0.00000	0.00000	0.00327	0.00221	0.00518	0.00128	0.00865	0.00506
P22315	HEMH_MOUSE	0.01670	0.01311	0.01422	0.00933	0.00902	0.01398	0.00970	0.00497	0.00690	0.00523	0.00515	0.00556
P22437	PGH1_MOUSE	0.00000	0.00000	0.00000	0.00000	0.00000	0.00000	0.03765	0.03597	0.06050	0.04812	0.04662	0.03733
P22599	A1AT2_MOUSE	0.00302	0.00126	0.00248	0.00383	0.00516	0.00035	0.06557	0.06055	0.11602	0.13828	0.11614	0.07093
P22682	CBL_MOUSE	0.00000	0.00000	0.00000	0.00000	0.00000	0.00000	0.00031	0.00000	0.00267	0.00056	0.00483	0.00000
P22892	AP1G1_MOUSE	0.00000	0.00000	0.00000	0.00000	0.00000	0.00000	0.00049	0.00979	0.00380	0.00265	0.00457	0.00373
P23116	EIF3A_MOUSE	0.00031	0.00073	0.00117	0.00039	0.00075	0.00243	0.01138	0.00936	0.01324	0.01199	0.00896	0.01177
P23198	CBX3_MOUSE	0.00255	0.00148	0.00157	0.00114	0.00142	0.00392	0.03379	0.02902	0.02473	0.02821	0.02687	0.03200
P23242	CXA1_MOUSE	0.03892	0.02888	0.03026	0.03342	0.03778	0.01639	0.00000	0.00000	0.00338	0.00147	0.00048	0.00032
P23298	KPCL_MOUSE	0.00000	0.00000	0.00000	0.00000	0.00000	0.00000	0.00449	0.00400	0.00813	0.00376	0.00638	0.00488
P23440	PDE6B_MOUSE	0.00000	0.00000	0.00000	0.00000	0.00000	0.00000	0.01176	0.00000	0.01813	0.01904	0.01824	0.03055
P23492	PNPH_MOUSE	0.00303	0.00450	0.00575	0.00544	0.00513	0.00290	0.05553	0.06005	0.05843	0.06424	0.06369	0.05986
P23506	PIMT_MOUSE	0.00000	0.00000	0.00000	0.00000	0.00000	0.00000	0.02277	0.02240	0.03107	0.02979	0.03753	0.02913
P23591	FCL_MOUSE	0.00269	0.00394	0.00283	0.00334	0.00215	0.00317	0.01703	0.01429	0.01559	0.01491	0.01322	0.01668
P23780	BGAL_MOUSE	0.00000	0.00000	0.00000	0.00000	0.00000	0.00000	0.00000	0.00000	0.00000	0.00000	0.00000	0.00081

P23927	CRYAB_MOUSE	0.08168	0.04543	0.11459	0.07551	0.08825	0.07971	0.05556	0.02493	0.03039	0.02696	0.02354	0.02073
P23953	EST1C_MOUSE	0.00463	0.00138	0.00424	0.00716	0.00416	0.00070	0.03960	0.02356	0.05734	0.06859	0.09419	0.03737
P24063	ITAL_MOUSE	0.00000	0.00000	0.00000	0.00000	0.00000	0.00000	0.00300	0.00181	0.00284	0.00381	0.00428	0.00424
P24270	CATA_MOUSE	0.03717	0.03177	0.02836	0.03174	0.03806	0.03208	0.04273	0.04614	0.04601	0.05111	0.06912	0.04754
P24369	PPIB_MOUSE	0.00000	0.00000	0.00000	0.00000	0.00000	0.00000	0.04071	0.05696	0.03960	0.04153	0.03078	0.03764
P24452	CAPG_MOUSE	0.00000	0.00000	0.00000	0.00000	0.00000	0.00000	0.04681	0.05755	0.04648	0.05317	0.03877	0.05656
P24457	CP2DB_MOUSE	0.00000	0.00000	0.00000	0.00000	0.00000	0.00000	0.00000	0.00412	0.00493	0.00412	0.00190	0.00291
P24472	GSTA4_MOUSE	0.00000	0.00000	0.00000	0.00000	0.00000	0.00000	0.04662	0.06395	0.02822	0.03728	0.03290	0.04189
P24527	LKHA4_MOUSE	0.00756	0.01682	0.01044	0.01558	0.01332	0.01370	0.03539	0.05028	0.03850	0.03884	0.04492	0.04197
P24547	IMDH2_MOUSE	0.00000	0.00000	0.00000	0.00000	0.00000	0.00000	0.00099	0.00244	0.00483	0.00400	0.00526	0.00312
P24549	AL1A1_MOUSE	0.01534	0.01253	0.01200	0.01560	0.01300	0.01292	0.66832	0.67443	0.59202	0.59767	0.70120	0.70525
P24668	MPRD_MOUSE	0.00000	0.00000	0.00000	0.00000	0.00000	0.00000	0.02581	0.04824	0.03687	0.03607	0.03483	0.04037
P25444	RS2_MOUSE	0.00276	0.00268	0.00467	0.00315	0.00368	0.00199	0.05930	0.03413	0.04910	0.03898	0.05313	0.04439
P25799	NFKB1_MOUSE	0.00000	0.00000	0.00000	0.00000	0.00000	0.00000	0.00170	0.00669	0.00566	0.00467	0.00233	0.00812
P25911	LYN_MOUSE	0.00000	0.00000	0.00000	0.00000	0.00000	0.00000	0.01201	0.01956	0.02669	0.02055	0.02937	0.02495
P25976	UBF1_MOUSE	0.00000	0.00000	0.00000	0.00000	0.00000	0.00000	0.00432	0.00780	0.00527	0.00716	0.00915	0.00840
P26039	TLN1_MOUSE	0.04784	0.03395	0.03073	0.03332	0.03441	0.02729	0.15940	0.18207	0.19424	0.18819	0.19518	0.20866
P26040	EZRI_MOUSE	0.00863	0.00695	0.00706	0.00608	0.00753	0.01212	0.05446	0.05784	0.07594	0.06366	0.06233	0.06050
P26041	MOES_MOUSE	0.01403	0.02307	0.01429	0.01630	0.01462	0.01838	0.76841	1.07445	1.07622	0.99350	0.81226	1.03251
P26043	RADI_MOUSE	0.00764	0.00910	0.01197	0.01364	0.00825	0.01113	0.05224	0.04855	0.05514	0.05325	0.06083	0.05174
P26149	3BHS2_MOUSE	0.00000	0.00000	0.00000	0.00000	0.00000	0.00000	0.00000	0.00000	0.00000	0.00000	0.00000	0.00000
P26231	CTNA1_MOUSE	0.02972	0.01571	0.01906	0.01952	0.02248	0.02580	0.05240	0.05041	0.04993	0.04931	0.05159	0.05986
P26350	PTMA_MOUSE	0.00000	0.00000	0.00000	0.00000	0.00000	0.00000	0.02487	0.16977	0.22412	0.20072	0.12254	0.11048
P26369	U2AF2_MOUSE	0.00000	0.00000	0.00000	0.00000	0.00000	0.00000	0.02395	0.01689	0.02182	0.02012	0.01981	0.02444
P26443	DHE3_MOUSE	0.07483	0.06493	0.07993	0.06166	0.06104	0.03990	0.04571	0.04630	0.03783	0.04359	0.03637	0.04424
P26516	PSMD7_MOUSE	0.00000	0.00000	0.00000	0.00000	0.00000	0.00000	0.00313	0.00424	0.00904	0.00696	0.01276	0.00391
P26618	PGFRA_MOUSE	0.00000	0.00000	0.00000	0.00000	0.00000	0.00000	0.00000	0.00000	0.00000	0.00000	0.00000	0.00000
P26638	SYSC_MOUSE	0.00229	0.00110	0.00119	0.00086	0.00078	0.00011	0.02703	0.01738	0.02938	0.02354	0.02504	0.02528
P26645	MARCS_MOUSE	0.00000	0.00000	0.00000	0.00000	0.00000	0.00000	0.00051	0.04576	0.08356	0.06627	0.04332	0.04046
P26883	FKB1A_MOUSE	0.00034	0.00016	0.00020	0.00028	0.00019	0.00000	0.00699	0.04640	0.01682	0.04345	0.00620	0.02417
P27005	S10A8_MOUSE	0.00000	0.00000	0.00000	0.00000	0.00000	0.00000	0.02467	0.01190	0.00890	0.00995	0.02381	0.01227
P27046	MA2A1_MOUSE	0.00000	0.00000	0.00000	0.00000	0.00000	0.00000	0.00462	0.00583	0.00591	0.00457	0.00339	0.00386
P27048	RSMB_MOUSE	0.00000	0.00000	0.00000	0.00000	0.00000	0.00000	0.00278	0.01270	0.00548	0.00697	0.00890	0.00473
P27546	MAP4_MOUSE	0.00998	0.01238	0.01057	0.01099	0.01500	0.01227	0.05623	0.04913	0.05603	0.05333	0.05542	0.06258

P27573	MYP0_MOUSE	0.00000	0.00000	0.00000	0.00000	0.00000	0.00000	0.05023	0.01307	0.07501	0.01043	0.02097	0.00715
P27601	GNA13_MOUSE	0.00000	0.00000	0.00000	0.00000	0.00000	0.00000	0.00846	0.01971	0.02578	0.01697	0.01962	0.02661
P27612	PLAP_MOUSE	0.00154	0.00247	0.00095	0.00204	0.00127	0.00157	0.00510	0.00955	0.00813	0.00985	0.00998	0.00844
P27659	RL3_MOUSE	0.00000	0.00000	0.00000	0.00000	0.00000	0.00000	0.03096	0.03233	0.04460	0.03885	0.04194	0.04510
P27773	PDIA3_MOUSE	0.00633	0.01329	0.01096	0.01745	0.01080	0.01348	0.25878	0.32206	0.33497	0.32230	0.24599	0.31416
P28063	PSB8_MOUSE	0.00000	0.00000	0.00000	0.00000	0.00000	0.00000	0.00841	0.01471	0.01689	0.00987	0.00377	0.01079
P28076	PSB9_MOUSE	0.00000	0.00000	0.00000	0.00000	0.00000	0.00000	0.00214	0.00354	0.00629	0.00575	0.00891	0.00531
P28271	ACOC_MOUSE	0.00621	0.00440	0.00861	0.00379	0.00351	0.00405	0.00698	0.01011	0.01139	0.00832	0.01142	0.01088
P28301	LYOX_MOUSE	0.00000	0.00000	0.00000	0.00000	0.00000	0.00000	0.01146	0.00709	0.00852	0.00377	0.01698	0.01093
P28352	APEX1_MOUSE	0.00145	0.00244	0.00232	0.00217	0.00202	0.00141	0.00103	0.00915	0.01931	0.01311	0.01637	0.00951
P28474	ADHX_MOUSE	0.03089	0.03018	0.01705	0.02379	0.02672	0.03885	0.03090	0.02573	0.03930	0.03003	0.04168	0.03353
P28481	CO2A1_MOUSE	0.00000	0.00000	0.00000	0.00000	0.00000	0.00000	0.00000	0.00000	0.00080	0.00000	0.00236	0.00000
P28650	PURA1_MOUSE	0.02796	0.01821	0.01678	0.02100	0.01845	0.01197	0.01907	0.00465	0.00406	0.00527	0.00187	0.00242
P28651	CAH8_MOUSE	0.00000	0.00000	0.00000	0.00000	0.00000	0.00000	0.01166	0.01248	0.00712	0.00998	0.01081	0.01327
P28653	PGS1_MOUSE	0.00000	0.00000	0.00000	0.00000	0.00000	0.00000	0.39199	0.16756	0.21600	0.11315	0.35490	0.28315
P28654	PGS2_MOUSE	0.00000	0.00000	0.00000	0.00000	0.00000	0.00000	0.08152	0.04432	0.06057	0.02735	0.08016	0.04484
P28656	NP1L1_MOUSE	0.00000	0.00000	0.00000	0.00000	0.00000	0.00000	0.05407	0.03847	0.05684	0.05188	0.06963	0.06915
P28658	ATX10_MOUSE	0.00000	0.00000	0.00000	0.00000	0.00000	0.00000	0.01462	0.01329	0.01578	0.01334	0.01526	0.01341
P28659	CELF1_MOUSE	0.00000	0.00000	0.00000	0.00000	0.00000	0.00000	0.00038	0.00038	0.00056	0.00034	0.00000	0.00012
P28660	NCKP1_MOUSE	0.00000	0.00000	0.00000	0.00000	0.00000	0.00000	0.00777	0.01120	0.01225	0.00981	0.01381	0.01230
P28661	SEPT4_MOUSE	0.00000	0.00000	0.00000	0.00000	0.00000	0.00000	0.01164	0.01910	0.01581	0.01498	0.01429	0.01403
P28665	MUG1_MOUSE	0.05564	0.02403	0.02810	0.04265	0.03047	0.02354	0.02828	0.02071	0.01591	0.02519	0.03510	0.00844
P28667	MRP_MOUSE	0.00000	0.00000	0.00000	0.00000	0.00000	0.00000	0.00000	0.00000	0.00416	0.00267	0.00000	0.00113
P28740	KIF2A_MOUSE	0.00000	0.00000	0.00000	0.00000	0.00000	0.00000	0.00000	0.00092	0.00188	0.00242	0.00348	0.00428
P28825	MEP1A_MOUSE	0.00000	0.00000	0.00000	0.00000	0.00000	0.00000	0.00037	0.00039	0.00052	0.00044	0.00036	0.00034
P28828	PTPRM_MOUSE	0.00000	0.00000	0.00000	0.00000	0.00000	0.00000	0.00213	0.00637	0.01161	0.01337	0.01309	0.00559
P28843	DPP4_MOUSE	0.00000	0.00000	0.00000	0.00000	0.00000	0.00000	0.03104	0.03716	0.04636	0.04218	0.03943	0.03987
P28867	KPCD_MOUSE	0.00000	0.00000	0.00000	0.00000	0.00000	0.00000	0.00447	0.00402	0.00340	0.00489	0.00253	0.00684
P29341	PABP1_MOUSE	0.00572	0.00353	0.00363	0.00223	0.00331	0.00456	0.03161	0.03767	0.02849	0.02790	0.02725	0.03533
P29351	PTN6_MOUSE	0.00000	0.00000	0.00000	0.00000	0.00000	0.00000	0.01267	0.01377	0.01751	0.01386	0.02136	0.01895
P29391	FRIL1_MOUSE	0.00000	0.00000	0.00000	0.00000	0.00000	0.00000	0.01001	0.01205	0.02011	0.02062	0.02577	0.01356
P29416	HEXA_MOUSE	0.00000	0.00000	0.00000	0.00000	0.00000	0.00000	0.00000	0.00929	0.00251	0.00268	0.00262	0.00486
P29452	CASP1_MOUSE	0.00000	0.00000	0.00000	0.00000	0.00000	0.00000	0.00059	0.00044	0.00179	0.00078	0.00177	0.00122
P29533	VCAM1_MOUSE	0.00000	0.00000	0.00000	0.00000	0.00000	0.00000	0.00590	0.00542	0.00495	0.00579	0.00000	0.00687

P29595	NEDD8_MOUSE	0.00000	0.00000	0.00000	0.00000	0.00000	0.00000	0.02120	0.01557	0.02383	0.02346	0.01582	0.01128
P29699	FETUA_MOUSE	0.03400	0.02695	0.02189	0.04288	0.02716	0.01511	0.06440	0.03653	0.09718	0.06672	0.10983	0.03770
P29758	OAT_MOUSE	0.03869	0.03142	0.15613	0.10486	0.14288	0.12512	0.04312	0.06282	0.07706	0.05803	0.08315	0.06137
P29788	VTNC_MOUSE	0.00000	0.00000	0.00000	0.00000	0.00000	0.00000	0.00889	0.00703	0.01708	0.01011	0.00649	0.01141
P30115	GSTA3_MOUSE	0.00000	0.00000	0.00000	0.00000	0.00020	0.00000	0.10352	0.14936	0.14658	0.11229	0.10607	0.18506
P30275	KCRU_MOUSE	0.00000	0.00000	0.00000	0.00000	0.00000	0.00000	0.02905	0.01724	0.02033	0.01968	0.02227	0.01506
P30412	PPIC_MOUSE	0.00000	0.00000	0.00000	0.00000	0.00000	0.00000	0.00209	0.00934	0.00129	0.00285	0.00059	0.00384
P30416	FKBP4_MOUSE	0.00000	0.00000	0.00000	0.00000	0.00000	0.00000	0.01107	0.01345	0.01232	0.01001	0.01252	0.01026
P30677	GNA14_MOUSE	0.00000	0.00000	0.00000	0.00000	0.00000	0.00000	0.00000	0.00357	0.00659	0.00113	0.00149	0.00188
P30681	HMGB2_MOUSE	0.00000	0.00000	0.00000	0.00000	0.00000	0.00000	0.00160	0.01715	0.02251	0.02589	0.01780	0.02112
P30999	CTND1_MOUSE	0.00000	0.00000	0.00000	0.00000	0.00000	0.00000	0.01927	0.03506	0.03224	0.02951	0.03539	0.03580
P31001	DESM_MOUSE	0.63848	0.56920	0.67731	0.56855	0.74781	0.78817	0.11950	0.12028	0.09168	0.11060	0.09671	0.09743
P31230	AIMP1_MOUSE	0.00000	0.00000	0.00000	0.00000	0.00000	0.00000	0.00445	0.00554	0.00562	0.00626	0.00576	0.00524
P31324	KAP3_MOUSE	0.00000	0.00000	0.00000	0.00000	0.00000	0.00000	0.00456	0.00382	0.00465	0.00124	0.00308	0.00000
P31428	DPEP1_MOUSE	0.00000	0.00000	0.00000	0.00000	0.00000	0.00000	0.06812	0.07551	0.10654	0.07024	0.08013	0.09744
P31532	SAA4_MOUSE	0.00000	0.00000	0.00000	0.00000	0.00000	0.00000	0.00540	0.00606	0.00314	0.00196	0.00296	0.00150
P31650	S6A11_MOUSE	0.00000	0.00000	0.00000	0.00000	0.00000	0.00000	0.00000	0.00019	0.00000	0.00000	0.00000	0.00000
P31725	S10A9_MOUSE	0.00000	0.00000	0.00000	0.00000	0.00000	0.00000	0.03381	0.08760	0.02150	0.05076	0.05472	0.13015
P31750	AKT1_MOUSE	0.00000	0.00000	0.00000	0.00000	0.00000	0.00000	0.00490	0.00289	0.00741	0.00628	0.00656	0.00632
P31786	ACBP_MOUSE	0.00000	0.00000	0.00000	0.00000	0.00000	0.00000	0.01992	0.00759	0.01582	0.01379	0.01075	0.00836
P31809	CEAM1_MOUSE	0.00123	0.00288	0.00232	0.00181	0.00177	0.00207	0.00000	0.00906	0.00492	0.00696	0.00642	0.00499
P31938	MP2K1_MOUSE	0.00000	0.00000	0.00000	0.00000	0.00000	0.00000	0.00405	0.00246	0.01063	0.00468	0.00580	0.00753
P32020	NLTP_MOUSE	0.00000	0.00000	0.00000	0.00000	0.00000	0.00000	0.00256	0.01721	0.02349	0.01790	0.00516	0.01716
P32067	LA_MOUSE	0.00000	0.00000	0.00000	0.00000	0.00000	0.00000	0.02013	0.01750	0.01910	0.02056	0.02444	0.02290
P32233	DRG1_MOUSE	0.00000	0.00000	0.00000	0.00000	0.00000	0.00000	0.00816	0.00636	0.00670	0.00702	0.00864	0.00689
P32261	ANT3_MOUSE	0.03272	0.02792	0.03200	0.03171	0.04935	0.02685	0.04331	0.04074	0.05810	0.05177	0.05783	0.05157
P32883	RASK_MOUSE	0.00000	0.00000	0.00000	0.00000	0.00000	0.00000	0.00000	0.00306	0.00490	0.00292	0.00148	0.00135
P32921	SYWC_MOUSE	0.01172	0.01146	0.01333	0.00839	0.00850	0.00915	0.06009	0.05956	0.04491	0.04747	0.04745	0.05712
P33267	CP2F2_MOUSE	0.00000	0.00000	0.00000	0.00000	0.00000	0.00000	0.26066	0.25906	0.13750	0.12262	0.25837	0.16917
P33434	MMP2_MOUSE	0.00000	0.00000	0.00000	0.00000	0.00000	0.00000	0.00850	0.00155	0.00764	0.00471	0.00676	0.00604
P33622	APOC3_MOUSE	0.00000	0.00000	0.00000	0.00000	0.00000	0.00000	0.01576	0.00521	0.00337	0.02459	0.00998	0.00000
P34022	RANG_MOUSE	0.00000	0.00000	0.00000	0.00000	0.00000	0.00000	0.01045	0.02181	0.01372	0.01058	0.01111	0.02090
P34152	FAK1_MOUSE	0.00000	0.00000	0.00000	0.00000	0.00000	0.00000	0.00943	0.00872	0.00696	0.00713	0.00654	0.01114
P34884	MIF_MOUSE	0.00000	0.00000	0.00000	0.00000	0.00000	0.00000	0.01887	0.02404	0.02016	0.02246	0.02113	0.02626

P34914	HYES_MOUSE	0.10000	0.11005	0.12226	0.08561	0.11288	0.11213	0.00380	0.00336	0.00285	0.00268	0.00330	0.00280
P34928	APOC1_MOUSE	0.00000	0.00000	0.00000	0.00000	0.00000	0.00000	0.00057	0.00000	0.00000	0.00117	0.00000	0.00000
P35123	UBP4_MOUSE	0.00000	0.00000	0.00000	0.00000	0.00000	0.00000	0.00000	0.00000	0.00071	0.00252	0.00228	0.00204
P35235	PTN11_MOUSE	0.00768	0.00810	0.00919	0.00826	0.00881	0.00637	0.01326	0.00812	0.01186	0.00995	0.01122	0.00940
P35242	SFTPA_MOUSE	0.00000	0.00000	0.00000	0.00000	0.00000	0.00000	0.21995	0.32692	0.24661	0.29302	0.22357	0.28494
P35276	RAB3D_MOUSE	0.00000	0.00000	0.00000	0.00000	0.00000	0.00000	0.00000	0.00143	0.00000	0.00192	0.00000	0.00180
P35278	RAB5C_MOUSE	0.00000	0.00000	0.00000	0.00000	0.00000	0.00000	0.02273	0.05293	0.04968	0.04828	0.04899	0.05957
P35279	RAB6A_MOUSE	0.00000	0.00000	0.00000	0.00000	0.00000	0.00000	0.06525	0.18188	0.09463	0.13356	0.06303	0.12892
P35282	RAB21_MOUSE	0.00931	0.00445	0.00697	0.00430	0.00349	0.00189	0.01684	0.01599	0.01809	0.01484	0.01290	0.01437
P35283	RAB12_MOUSE	0.00000	0.00000	0.00000	0.00000	0.00000	0.00000	0.02316	0.02018	0.01211	0.01530	0.01831	0.01864
P35285	RB22A_MOUSE	0.00000	0.00000	0.00000	0.00000	0.00000	0.00000	0.01253	0.01291	0.01624	0.01011	0.01079	0.01056
P35288	RAB23_MOUSE	0.00000	0.00000	0.00000	0.00000	0.00000	0.00000	0.01088	0.00000	0.00990	0.00000	0.00591	0.00349
P35293	RAB18_MOUSE	0.01812	0.01735	0.01340	0.00845	0.01118	0.01148	0.02113	0.02037	0.02186	0.02785	0.01380	0.02382
P35330	ICAM2_MOUSE	0.00000	0.00000	0.00000	0.00000	0.00000	0.00000	0.00924	0.01819	0.01376	0.01327	0.01005	0.01658
P35486	ODPA_MOUSE	0.12164	0.12812	0.15869	0.11753	0.15767	0.17654	0.07059	0.06002	0.04765	0.04878	0.03229	0.05319
P35505	FAAA_MOUSE	0.00000	0.00000	0.00000	0.00000	0.00000	0.00000	0.00126	0.00346	0.00354	0.00319	0.00094	0.00152
P35550	FBRL_MOUSE	0.00000	0.00000	0.00000	0.00000	0.00000	0.00000	0.00172	0.00395	0.00881	0.00774	0.00717	0.00433
P35564	CALX_MOUSE	0.01620	0.02324	0.02975	0.02678	0.03281	0.01913	0.09358	0.10412	0.12850	0.12461	0.10667	0.11366
P35576	G6PC_MOUSE	0.00000	0.00000	0.00000	0.00000	0.00000	0.00000	0.00000	0.00000	0.00000	0.00146	0.00000	0.00000
P35585	AP1M1_MOUSE	0.00000	0.00000	0.00000	0.00000	0.00000	0.00000	0.02673	0.01777	0.03125	0.02672	0.02721	0.03799
P35700	PRDX1_MOUSE	0.03657	0.01567	0.03842	0.01707	0.03083	0.03526	0.07235	0.12183	0.06101	0.07224	0.07084	0.07012
P35762	CD81_MOUSE	0.00000	0.00000	0.00000	0.00000	0.00000	0.00000	0.04981	0.03018	0.03872	0.03865	0.04771	0.05273
P35802	GPM6A_MOUSE	0.00000	0.00000	0.00000	0.00000	0.00000	0.00000	0.00000	0.00214	0.00503	0.00248	0.00133	0.00131
P35803	GPM6B_MOUSE	0.00000	0.00000	0.00000	0.00000	0.00000	0.00000	0.00408	0.00545	0.00438	0.00183	0.00082	0.00682
P35821	PTN1_MOUSE	0.00000	0.00000	0.00000	0.00000	0.00000	0.00000	0.00102	0.00739	0.00543	0.00474	0.00442	0.00455
P35822	PTPRK_MOUSE	0.00000	0.00000	0.00000	0.00000	0.00000	0.00000	0.00000	0.00000	0.00000	0.00000	0.00000	0.00000
P35831	PTN12_MOUSE	0.00000	0.00000	0.00000	0.00000	0.00000	0.00000	0.00000	0.00000	0.00856	0.00317	0.00176	0.00014
P35922	FMR1_MOUSE	0.00000	0.00000	0.00000	0.00000	0.00000	0.00000	0.00000	0.00037	0.00146	0.00000	0.00052	0.00000
P35979	RL12_MOUSE	0.01819	0.01438	0.02097	0.01686	0.01585	0.01170	0.04386	0.02794	0.03194	0.03343	0.03554	0.03135
P35980	RL18_MOUSE	0.00000	0.00000	0.00000	0.00000	0.00000	0.00000	0.08745	0.08429	0.07767	0.09368	0.08461	0.08789
P36371	TAP2_MOUSE	0.00000	0.00000	0.00000	0.00000	0.00000	0.00000	0.00330	0.00383	0.00994	0.00478	0.00320	0.00524
P36536	SAR1A_MOUSE	0.00000	0.00000	0.00000	0.00000	0.00000	0.00000	0.00000	0.00000	0.00330	0.00169	0.00041	0.00000
P36552	HEM6_MOUSE	0.00313	0.00157	0.00147	0.00182	0.00175	0.00152	0.00089	0.00058	0.00110	0.00118	0.00067	0.00074
P36916	GNL1_MOUSE	0.00000	0.00000	0.00000	0.00000	0.00000	0.00000	0.00000	0.00121	0.00195	0.00033	0.00057	0.00080

P36993	PPM1B_MOUSE	0.00000	0.00000	0.00000	0.00000	0.00000	0.00000	0.01539	0.01119	0.01635	0.01380	0.01287	0.01509
P37040	NCPR_MOUSE	0.00118	0.00085	0.00115	0.00065	0.00139	0.00060	0.12028	0.12116	0.08204	0.07997	0.09291	0.10752
P37804	TAGL_MOUSE	0.00410	0.00158	0.00255	0.00515	0.00186	0.00700	0.08137	0.08260	0.06724	0.07323	0.04917	0.06632
P38060	HMGCL_MOUSE	0.00372	0.00275	0.00490	0.00577	0.00487	0.00578	0.00089	0.00009	0.00053	0.00030	0.00050	0.00027
P38647	GRP75_MOUSE	0.25964	0.18378	0.18954	0.21227	0.22564	0.20098	0.05036	0.05102	0.04955	0.04835	0.03672	0.05580
P39054	DYN2_MOUSE	0.00235	0.01325	0.00214	0.00219	0.00230	0.00223	0.03909	0.02915	0.03680	0.03383	0.03751	0.03540
P39061	COIA1_MOUSE	0.00000	0.00000	0.00000	0.00000	0.00000	0.00000	0.02751	0.03064	0.03579	0.02386	0.04264	0.03274
P39447	ZO1_MOUSE	0.00165	0.00240	0.00078	0.00130	0.00080	0.00135	0.04290	0.06341	0.07684	0.06604	0.06567	0.07143
P39654	LOX15_MOUSE	0.00000	0.00000	0.00000	0.00000	0.00000	0.00000	0.00051	0.00092	0.00264	0.00097	0.00571	0.00697
P39655	LOX12_MOUSE	0.00000	0.00000	0.00000	0.00000	0.00000	0.00000	0.00276	0.00010	0.00566	0.00782	0.00232	0.00145
P39688	FYN_MOUSE	0.00000	0.00000	0.00000	0.00000	0.00000	0.00000	0.00128	0.00328	0.00129	0.00000	0.00000	0.00227
P39749	FEN1_MOUSE	0.00000	0.00000	0.00000	0.00000	0.00000	0.00000	0.00000	0.00000	0.00150	0.00000	0.00000	0.00000
P39876	TIMP3_MOUSE	0.00000	0.00000	0.00000	0.00000	0.00000	0.00000	0.01051	0.00431	0.00843	0.00738	0.00886	0.00071
P40124	CAP1_MOUSE	0.00244	0.00167	0.00120	0.00210	0.00165	0.00055	0.14055	0.16198	0.14078	0.16501	0.15359	0.17053
P40142	TKT_MOUSE	0.00336	0.00315	0.00228	0.00195	0.00142	0.00278	0.21656	0.17030	0.22566	0.19087	0.21363	0.22414
P40237	CD82_MOUSE	0.00000	0.00000	0.00000	0.00000	0.00000	0.00000	0.01857	0.01400	0.01724	0.01203	0.00817	0.01571
P40240	CD9_MOUSE	0.00000	0.00000	0.00000	0.00000	0.00000	0.00000	0.00000	0.00362	0.00463	0.00328	0.00299	0.00154
P40336	VP26A_MOUSE	0.00562	0.00884	0.01188	0.01043	0.00804	0.00834	0.01979	0.02126	0.02332	0.02136	0.02241	0.01900
P40630	TFAM_MOUSE	0.00217	0.00629	0.00260	0.00388	0.00266	0.00636	0.00166	0.00513	0.00000	0.00265	0.00428	0.01201
P40936	INMT_MOUSE	0.00000	0.00000	0.00000	0.00000	0.00000	0.00000	0.81196	1.33826	1.03710	1.15794	0.93674	1.16838
P41105	RL28_MOUSE	0.00000	0.00000	0.00000	0.00000	0.00000	0.00000	0.01350	0.01423	0.02400	0.02063	0.01694	0.01715
P41216	ACSL1_MOUSE	0.21015	0.23075	0.13670	0.23645	0.21297	0.32924	0.03994	0.02497	0.02315	0.02070	0.01895	0.02242
P41241	CSK_MOUSE	0.00000	0.00000	0.00000	0.00000	0.00000	0.00000	0.00336	0.00158	0.00557	0.00450	0.00544	0.00432
P41245	MMP9_MOUSE	0.00000	0.00000	0.00000	0.00000	0.00000	0.00000	0.00209	0.00184	0.00262	0.00553	0.01422	0.01275
P41317	MBL2_MOUSE	0.00000	0.00000	0.00000	0.00000	0.00000	0.00000	0.00000	0.00078	0.00000	0.00089	0.00074	0.00000
P41438	S19A1_MOUSE	0.00000	0.00000	0.00000	0.00000	0.00000	0.00000	0.00000	0.00000	0.00000	0.00000	0.00000	0.00000
P41731	CD63_MOUSE	0.00000	0.00000	0.00000	0.00000	0.00000	0.00000	0.00182	0.00268	0.00473	0.00553	0.00309	0.00781
P42125	ECI1_MOUSE	0.26257	0.28070	0.31733	0.19243	0.21783	0.17501	0.03757	0.04400	0.04104	0.03604	0.03056	0.03555
P42208	SEPT2_MOUSE	0.01143	0.00789	0.01122	0.00731	0.01025	0.00856	0.03692	0.03891	0.05256	0.04473	0.04228	0.04262
P42225	STAT1_MOUSE	0.00175	0.00082	0.00124	0.00100	0.00124	0.00082	0.04025	0.05365	0.04946	0.04886	0.03574	0.04451
P42227	STAT3_MOUSE	0.03511	0.01954	0.06477	0.02209	0.02776	0.01551	0.01259	0.01174	0.02283	0.01393	0.01964	0.01624
P42232	STA5B_MOUSE	0.00000	0.00000	0.00000	0.00000	0.00000	0.00000	0.00349	0.00264	0.01049	0.00542	0.00591	0.00391
P42337	PK3CA_MOUSE	0.00000	0.00000	0.00000	0.00000	0.00000	0.00000	0.00000	0.00000	0.00000	0.00000	0.00000	0.00000
P42567	EPS15_MOUSE	0.00000	0.00000	0.00000	0.00000	0.00000	0.00000	0.02085	0.02447	0.02833	0.02906	0.03441	0.03121

P42669	PURA_MOUSE	0.00000	0.00000	0.00000	0.00000	0.00000	0.00000	0.06364	0.07048	0.07122	0.07038	0.06745	0.08967
P42859	HD_MOUSE	0.00000	0.00000	0.00000	0.00000	0.00000	0.00000	0.00000	0.00450	0.00068	0.00393	0.00000	0.00064
P42932	TCPQ_MOUSE	0.01336	0.00998	0.01591	0.01610	0.01467	0.01095	0.03209	0.03281	0.03069	0.03439	0.02092	0.03464
P43023	CX6A2_MOUSE	0.00000	0.00000	0.00000	0.00000	0.00000	0.00000	0.01036	0.00294	0.00514	0.00562	0.00771	0.00166
P43024	CX6A1_MOUSE	0.00000	0.00000	0.00000	0.00000	0.00000	0.00000	0.01947	0.01056	0.00873	0.00831	0.00972	0.00649
P43025	TETN_MOUSE	0.00000	0.00000	0.00000	0.00000	0.00000	0.00000	0.00000	0.00488	0.00000	0.00162	0.00000	0.00000
P43274	H14_MOUSE	0.00000	0.00000	0.00000	0.00000	0.00000	0.00000	0.25531	0.78846	0.41058	0.68237	0.30053	0.54180
P43275	H11_MOUSE	0.00000	0.00000	0.00000	0.00000	0.00000	0.00000	0.06560	0.05406	0.06829	0.09024	0.10680	0.09553
P43276	H15_MOUSE	0.00000	0.00000	0.00000	0.00000	0.00000	0.00000	0.01538	0.05499	0.04320	0.08929	0.02371	0.07088
P43277	H13_MOUSE	0.00000	0.00000	0.00000	0.00000	0.00000	0.00000	0.00000	0.15572	0.12645	0.08014	0.06623	0.07803
P43406	ITAV_MOUSE	0.00000	0.00000	0.00000	0.00000	0.00000	0.00000	0.00820	0.00986	0.01053	0.01045	0.00985	0.01287
P45376	ALDR_MOUSE	0.10942	0.07273	0.15139	0.09183	0.08863	0.09650	0.09662	0.08654	0.09867	0.09220	0.07260	0.09858
P45377	ALD2_MOUSE	0.00000	0.00000	0.00000	0.00000	0.00000	0.00000	0.00560	0.00588	0.01018	0.00979	0.00787	0.00806
P45591	COF2_MOUSE	0.00385	0.01287	0.02610	0.03103	0.03445	0.00328	0.08087	0.04771	0.07665	0.07041	0.07221	0.05872
P45700	MA1A1_MOUSE	0.00000	0.00000	0.00000	0.00000	0.00000	0.00000	0.00000	0.00144	0.00000	0.00000	0.00120	0.00000
P45878	FKBP2_MOUSE	0.00000	0.00000	0.00000	0.00000	0.00000	0.00000	0.00139	0.00689	0.00380	0.00421	0.00110	0.00403
P45952	ACADM_MOUSE	0.36328	0.56406	0.38755	0.33069	0.50466	0.69638	0.05596	0.03878	0.03639	0.03000	0.02623	0.03003
P46061	RAGP1_MOUSE	0.00000	0.00000	0.00000	0.00000	0.00000	0.00000	0.00488	0.00536	0.00728	0.00643	0.01008	0.00791
P46062	SIPA1_MOUSE	0.00000	0.00000	0.00000	0.00000	0.00000	0.00000	0.00000	0.00156	0.00063	0.00114	0.00082	0.00038
P46412	GPX3_MOUSE	0.00000	0.00000	0.00000	0.00000	0.00000	0.00000	0.06132	0.03403	0.03693	0.03105	0.02422	0.03481
P46414	CDN1B_MOUSE	0.00000	0.00000	0.00000	0.00000	0.00000	0.00000	0.00000	0.00237	0.00468	0.00377	0.00175	0.00225
P46460	NSF_MOUSE	0.00000	0.00000	0.00000	0.00000	0.00000	0.00000	0.01079	0.00992	0.01209	0.00971	0.00879	0.00854
P46467	VPS4B_MOUSE	0.00000	0.00000	0.00000	0.00000	0.00000	0.00000	0.01042	0.01052	0.00973	0.01151	0.00825	0.00981
P46471	PRS7_MOUSE	0.00295	0.00794	0.00497	0.00443	0.00772	0.00598	0.01982	0.02948	0.02136	0.02588	0.01235	0.02652
P46638	RB11B_MOUSE	0.00000	0.00000	0.00000	0.00000	0.00000	0.00000	0.05942	0.11037	0.07827	0.10551	0.07912	0.09904
P46656	ADX_MOUSE	0.00000	0.00000	0.00000	0.00000	0.00000	0.00000	0.00192	0.00675	0.00000	0.00584	0.00417	0.00325
P46662	MERL_MOUSE	0.00000	0.00000	0.00000	0.00000	0.00000	0.00000	0.00000	0.00000	0.00000	0.00000	0.00000	0.00000
P46664	PURA2_MOUSE	0.00000	0.00000	0.00000	0.00000	0.00000	0.00000	0.02589	0.02818	0.04032	0.03129	0.04615	0.03480
P46735	MYO1B_MOUSE	0.00158	0.00056	0.00078	0.00181	0.00160	0.00100	0.06792	0.06240	0.05871	0.05113	0.06949	0.06763
P46737	BRCC3_MOUSE	0.00000	0.00000	0.00000	0.00000	0.00000	0.00000	0.00000	0.00297	0.00000	0.00000	0.00000	0.00212
P46935	NEDD4_MOUSE	0.00457	0.00615	0.00502	0.00408	0.00567	0.00511	0.01426	0.01801	0.01525	0.01325	0.01520	0.01421
P46938	YAP1_MOUSE	0.00000	0.00000	0.00000	0.00000	0.00000	0.00000	0.00000	0.00344	0.00283	0.00212	0.00038	0.00117
P46978	STT3A_MOUSE	0.00000	0.00000	0.00000	0.00000	0.00000	0.00000	0.01015	0.01107	0.01078	0.01142	0.01024	0.01015
P47199	QOR_MOUSE	0.00585	0.00641	0.00573	0.00412	0.00345	0.00587	0.00377	0.00495	0.00565	0.00257	0.00308	0.00593

P47226	TES_MOUSE	0.00000	0.00000	0.00000	0.00000	0.00000	0.00000	0.00000	0.00378	0.00290	0.00377	0.00535	0.00532
P47713	PA24A_MOUSE	0.00000	0.00000	0.00000	0.00000	0.00000	0.00000	0.00000	0.00000	0.00000	0.00000	0.00000	0.00000
P47738	ALDH2_MOUSE	0.15309	0.16731	0.10028	0.13238	0.13400	0.19994	0.48262	0.60840	0.35242	0.44399	0.37830	0.53112
P47739	AL3A1_MOUSE	0.00000	0.00000	0.00000	0.00000	0.00000	0.00000	0.01845	0.01662	0.01332	0.01353	0.01380	0.01696
P47740	AL3A2_MOUSE	0.00000	0.00000	0.00000	0.00000	0.00000	0.00000	0.00821	0.01606	0.00790	0.00968	0.00890	0.01410
P47753	CAZA1_MOUSE	0.00000	0.00000	0.00000	0.00000	0.00000	0.00000	0.02528	0.02352	0.03285	0.02876	0.03458	0.03137
P47754	CAZA2_MOUSE	0.01840	0.01286	0.01831	0.01558	0.01220	0.01371	0.08240	0.08689	0.10174	0.09405	0.10422	0.11136
P47757	CAPZB_MOUSE	0.01538	0.01222	0.01123	0.01332	0.01028	0.01272	0.02895	0.03548	0.02648	0.02902	0.02390	0.03463
P47758	SRPRB_MOUSE	0.00379	0.00326	0.00405	0.00493	0.00260	0.00308	0.05068	0.01264	0.01687	0.01661	0.01799	0.02363
P47791	GSHR_MOUSE	0.00198	0.00263	0.00238	0.00310	0.00277	0.00205	0.01425	0.01469	0.01867	0.01571	0.02415	0.01759
P47802	MTX1_MOUSE	0.00421	0.00327	0.00499	0.00705	0.00416	0.00572	0.00000	0.00154	0.00533	0.00383	0.00322	0.00201
P47809	MP2K4_MOUSE	0.00415	0.00455	0.00482	0.00280	0.00315	0.00478	0.00180	0.00083	0.00311	0.00348	0.00091	0.00294
P47811	MK14_MOUSE	0.00097	0.00126	0.00195	0.00097	0.00105	0.00112	0.00094	0.00300	0.00448	0.00374	0.00404	0.00382
P47856	GFPT1_MOUSE	0.00000	0.00000	0.00000	0.00000	0.00000	0.00000	0.00659	0.00779	0.01206	0.00934	0.01375	0.00851
P47857	PFKAM_MOUSE	0.17075	0.19663	0.22024	0.19535	0.18764	0.15939	0.03401	0.01152	0.01223	0.01203	0.01537	0.00913
P47880	IBP6_MOUSE	0.00000	0.00000	0.00000	0.00000	0.00000	0.00000	0.01342	0.00279	0.00226	0.00195	0.00285	0.00099
P47911	RL6_MOUSE	0.00401	0.00838	0.01371	0.00921	0.00758	0.00498	0.00058	0.01530	0.02501	0.02256	0.01518	0.01576
P47915	RL29_MOUSE	0.00000	0.00000	0.00000	0.00000	0.00000	0.00000	0.00000	0.01015	0.00930	0.01229	0.00135	0.00360
P47934	CACP_MOUSE	0.13852	0.20336	0.23661	0.18004	0.17872	0.13161	0.01221	0.00256	0.00654	0.00373	0.00420	0.00319
P47941	CRKL_MOUSE	0.00000	0.00000	0.00000	0.00000	0.00000	0.00000	0.00911	0.00698	0.01231	0.01229	0.01963	0.01021
P47955	RLA1_MOUSE	0.01279	0.00451	0.01614	0.00769	0.01074	0.00465	0.00712	0.00504	0.00000	0.00000	0.00739	0.00000
P47962	RL5_MOUSE	0.00521	0.00445	0.00266	0.00677	0.00288	0.00486	0.00120	0.02201	0.02612	0.02404	0.01498	0.01547
P47963	RL13_MOUSE	0.00000	0.00000	0.00000	0.00000	0.00000	0.00000	0.01399	0.03321	0.02381	0.03039	0.01642	0.02060
P47964	RL36_MOUSE	0.00000	0.00000	0.00000	0.00000	0.00000	0.00000	0.00000	0.00166	0.00599	0.00238	0.00000	0.00167
P47968	RPIA_MOUSE	0.00000	0.00000	0.00000	0.00000	0.00000	0.00000	0.00000	0.00000	0.00130	0.00113	0.00090	0.00058
P48024	EIF1_MOUSE	0.00000	0.00000	0.00000	0.00000	0.00000	0.00000	0.02381	0.01753	0.02578	0.01963	0.02259	0.01778
P48025	KSYK_MOUSE	0.00000	0.00000	0.00000	0.00000	0.00000	0.00000	0.00088	0.00338	0.00915	0.00437	0.00993	0.00747
P48036	ANXA5_MOUSE	0.03878	0.04081	0.03976	0.03602	0.05071	0.04838	0.32279	0.34390	0.29553	0.32890	0.29536	0.35966
P48193	41_MOUSE	0.00482	0.00587	0.00430	0.00578	0.00405	0.00565	0.00722	0.00711	0.01067	0.01358	0.01936	0.00640
P48428	TBCA_MOUSE	0.00000	0.00000	0.00000	0.00000	0.00000	0.00000	0.00105	0.00451	0.00643	0.00505	0.00661	0.00708
P48678	LMNA_MOUSE	0.04897	0.04898	0.04000	0.03126	0.04691	0.02581	0.11404	0.13304	0.15785	0.13272	0.11834	0.11967
P48722	HS74L_MOUSE	0.00412	0.00569	0.00508	0.00513	0.00454	0.00555	0.01327	0.01200	0.01670	0.01521	0.01810	0.01672
P48758	CBR1_MOUSE	0.01258	0.00654	0.00997	0.00964	0.00836	0.01180	0.05296	0.04323	0.04175	0.04579	0.04961	0.05566
P48771	CX7A2_MOUSE	0.00000	0.00000	0.00000	0.00000	0.00000	0.00000	0.00634	0.01171	0.00939	0.01002	0.00683	0.00808

P48774	GSTM5_MOUSE	0.00695	0.00602	0.00617	0.00504	0.00848	0.00735	0.00440	0.00769	0.00600	0.00518	0.00317	0.00587
P48787	TNNI3_MOUSE	0.52029	0.22565	0.39852	0.39707	0.62714	0.06821	0.02231	0.05452	0.06682	0.07017	0.03977	0.03925
P48962	ADT1_MOUSE	0.69177	0.62660	0.51233	1.07681	0.40713	1.10237	0.17298	0.26800	0.09987	0.17164	0.13347	0.14348
P49182	HEP2_MOUSE	0.00000	0.00000	0.00000	0.00000	0.00000	0.00000	0.00126	0.00361	0.00635	0.00321	0.00173	0.00320
P49222	EPB42_MOUSE	0.00000	0.00000	0.00000	0.00000	0.00000	0.00000	0.00070	0.00000	0.00000	0.00518	0.01704	0.00000
P49290	PERE_MOUSE	0.00000	0.00000	0.00000	0.00000	0.00000	0.00000	0.00738	0.00732	0.00697	0.00633	0.01696	0.02611
P49312	ROA1_MOUSE	0.00636	0.00985	0.00396	0.00851	0.00523	0.00813	0.05796	0.07821	0.05514	0.06411	0.06372	0.06939
P49442	INPP_MOUSE	0.00290	0.00394	0.00445	0.00352	0.00381	0.00319	0.00820	0.00562	0.00927	0.00889	0.01110	0.01075
P49443	PPM1A_MOUSE	0.00000	0.00000	0.00000	0.00000	0.00000	0.00000	0.00252	0.00769	0.00494	0.00457	0.00298	0.00515
P49446	PTPRE_MOUSE	0.00000	0.00000	0.00000	0.00000	0.00000	0.00000	0.00175	0.00205	0.00297	0.00280	0.00132	0.00244
P49586	PCY1A_MOUSE	0.00203	0.00179	0.00190	0.00179	0.00083	0.00156	0.01155	0.01007	0.00985	0.00858	0.01035	0.00928
P49615	CDK5_MOUSE	0.00000	0.00000	0.00000	0.00000	0.00000	0.00000	0.00442	0.00153	0.00329	0.00199	0.00256	0.00197
P49710	HCLS1_MOUSE	0.00000	0.00000	0.00000	0.00000	0.00000	0.00000	0.00904	0.00325	0.00882	0.00816	0.00440	0.00827
P49722	PSA2_MOUSE	0.00000	0.00000	0.00000	0.00000	0.00000	0.00000	0.02312	0.02652	0.03663	0.02693	0.03514	0.03034
P49769	PSN1_MOUSE	0.00000	0.00000	0.00000	0.00000	0.00000	0.00000	0.00000	0.00158	0.00000	0.00170	0.00000	0.00000
P49813	TMOD1_MOUSE	0.03717	0.06976	0.03271	0.02996	0.05372	0.05051	0.01601	0.01421	0.01519	0.01406	0.01363	0.01550
P49817	CAV1_MOUSE	0.00000	0.00000	0.00000	0.00000	0.00000	0.00000	0.56397	0.52892	0.82931	0.60134	0.72309	0.70973
P49935	CATH_MOUSE	0.00000	0.00000	0.00000	0.00000	0.00000	0.00000	0.03104	0.03106	0.03284	0.03792	0.02126	0.03561
P49962	SRP09_MOUSE	0.00000	0.00000	0.00000	0.00000	0.00000	0.00000	0.00218	0.00169	0.00251	0.00166	0.00000	0.00175
P50096	IMDH1_MOUSE	0.00000	0.00000	0.00000	0.00000	0.00000	0.00000	0.00298	0.00466	0.00590	0.00603	0.00478	0.00508
P50136	ODBA_MOUSE	0.02455	0.01750	0.01561	0.01315	0.01786	0.02328	0.00080	0.00415	0.00277	0.00172	0.00315	0.00340
P50171	DHB8_MOUSE	0.01279	0.01080	0.00928	0.01310	0.01320	0.01622	0.00511	0.00909	0.00302	0.00025	0.00430	0.00358
P50172	DHI1_MOUSE	0.00000	0.00000	0.00000	0.00000	0.00000	0.00000	0.05076	0.06480	0.03712	0.04562	0.04954	0.05473
P50247	SAHH_MOUSE	0.00000	0.00000	0.00000	0.00000	0.00000	0.00000	0.02420	0.03056	0.04046	0.03396	0.03356	0.03321
P50285	FMO1_MOUSE	0.00000	0.00000	0.00000	0.00000	0.00000	0.00000	0.05062	0.06537	0.05296	0.05324	0.05118	0.06402
P50295	ARY2_MOUSE	0.00000	0.00000	0.00000	0.00000	0.00000	0.00000	0.01069	0.00645	0.01566	0.00904	0.00457	0.00145
P50396	GDIA_MOUSE	0.01318	0.01767	0.01152	0.00841	0.01724	0.01791	0.04655	0.05914	0.05378	0.05594	0.04740	0.06176
P50404	SFTPD_MOUSE	0.00000	0.00000	0.00000	0.00000	0.00000	0.00000	0.00701	0.00731	0.01036	0.00735	0.01073	0.00915
P50405	PSPB_MOUSE	0.00000	0.00000	0.00000	0.00000	0.00000	0.00000	0.01149	0.02529	0.00732	0.00702	0.01224	0.00576
P50427	STS_MOUSE	0.00000	0.00000	0.00000	0.00000	0.00000	0.00000	0.00305	0.00211	0.00468	0.00381	0.00353	0.00162
P50429	ARSB_MOUSE	0.00000	0.00000	0.00000	0.00000	0.00000	0.00000	0.00000	0.00000	0.00036	0.00052	0.00029	0.00096
P50431	GLYC_MOUSE	0.00000	0.00000	0.00000	0.00000	0.00000	0.00000	0.00000	0.00000	0.00035	0.00000	0.00033	0.00032
P50462	CSRP3_MOUSE	0.11166	0.06794	0.09697	0.09131	0.08785	0.05341	0.12536	0.04637	0.01833	0.04771	0.04061	0.02860
P50516	VATA_MOUSE	0.00279	0.00380	0.00130	0.00249	0.00190	0.00305	0.02647	0.02435	0.03021	0.02578	0.02878	0.02691

P50518	VATE1_MOUSE	0.00000	0.00000	0.00000	0.00000	0.00000	0.00000	0.01644	0.01469	0.01836	0.01034	0.01510	0.01505
P50543	S10AB_MOUSE	0.00000	0.00000	0.00000	0.00000	0.00000	0.00000	0.15770	0.15524	0.14215	0.19473	0.10244	0.16721
P50544	ACADV_MOUSE	0.44235	0.40293	0.30463	0.40452	0.42136	0.46196	0.06084	0.03126	0.03660	0.03275	0.02965	0.03496
P50580	PA2G4_MOUSE	0.00000	0.00000	0.00000	0.00000	0.00000	0.00000	0.02610	0.02807	0.03090	0.03373	0.02696	0.02692
P50637	TSPO_MOUSE	0.00000	0.00000	0.00000	0.00000	0.00000	0.00000	0.00030	0.00371	0.00528	0.00120	0.00000	0.00243
P50752	TNNT2_MOUSE	0.50786	0.76488	0.63959	0.59739	0.61015	0.74399	0.01993	0.01779	0.02788	0.03378	0.02864	0.01998
P51125	ICAL_MOUSE	0.00467	0.00650	0.00337	0.00214	0.00231	0.00245	0.02242	0.02830	0.02888	0.02613	0.02641	0.02796
P51150	RAB7A_MOUSE	0.02553	0.02690	0.01524	0.02369	0.01869	0.03415	0.10081	0.14482	0.07700	0.11036	0.07774	0.11576
P51174	ACADL_MOUSE	1.06530	1.00148	1.00286	0.73649	0.94492	1.08987	0.13037	0.11787	0.10064	0.10536	0.09856	0.10407
P51410	RL9_MOUSE	0.00000	0.00000	0.00000	0.00000	0.00000	0.00000	0.08405	0.06238	0.05467	0.04909	0.07209	0.04882
P51432	PLCB3_MOUSE	0.00000	0.00000	0.00000	0.00000	0.00000	0.00000	0.01840	0.01683	0.02073	0.02124	0.02729	0.02422
P51437	CAMP_MOUSE	0.00000	0.00000	0.00000	0.00000	0.00000	0.00000	0.02913	0.04138	0.01687	0.03065	0.03335	0.05010
P51637	CAV3_MOUSE	0.00000	0.00000	0.00000	0.00000	0.00000	0.00000	0.00000	0.00161	0.00035	0.00068	0.00118	0.00000
P51660	DHB4_MOUSE	0.00429	0.00261	0.00458	0.00207	0.00179	0.00273	0.02241	0.01551	0.01853	0.01704	0.01648	0.01626
P51661	DHI2_MOUSE	0.00000	0.00000	0.00000	0.00000	0.00000	0.00000	0.00000	0.00000	0.00000	0.00000	0.00000	0.00000
P51667	MLRV_MOUSE	0.01755	0.01677	0.03279	0.04021	0.01845	0.03009	0.00846	0.00114	0.00000	0.00000	0.00000	0.00105
P51807	DYLT1_MOUSE	0.00000	0.00000	0.00000	0.00000	0.00000	0.00000	0.01418	0.00612	0.00419	0.00211	0.00161	0.00123
P51855	GSHB_MOUSE	0.00141	0.00118	0.00170	0.00113	0.00182	0.00191	0.00968	0.01355	0.01178	0.01160	0.01662	0.01485
P51859	HDGF_MOUSE	0.00000	0.00000	0.00000	0.00000	0.00000	0.00000	0.02204	0.03044	0.03769	0.03095	0.03268	0.03304
P51863	VA0D1_MOUSE	0.00000	0.00000	0.00000	0.00000	0.00000	0.00000	0.00317	0.00565	0.00276	0.00274	0.00116	0.00315
P51881	ADT2_MOUSE	0.07199	0.04605	0.04060	0.13941	0.05040	0.09725	0.04648	0.12219	0.04884	0.06265	0.03307	0.07068
P51885	LUM_MOUSE	0.00000	0.00000	0.00000	0.00000	0.00000	0.00000	0.41035	0.27292	0.30595	0.24506	0.28957	0.29874
P51906	EAA3_MOUSE	0.00000	0.00000	0.00000	0.00000	0.00000	0.00000	0.00000	0.00033	0.00033	0.00020	0.00036	0.00000
P51912	AAAT_MOUSE	0.00000	0.00000	0.00000	0.00000	0.00000	0.00000	0.01491	0.00781	0.01561	0.00844	0.01594	0.01202
P52196	THTR_MOUSE	0.00000	0.00000	0.00000	0.00000	0.00000	0.00000	0.04915	0.04622	0.04009	0.03696	0.05208	0.04747
P52332	JAK1_MOUSE	0.00000	0.00000	0.00000	0.00000	0.00000	0.00000	0.00000	0.00116	0.00454	0.00251	0.00223	0.00139
P52430	PON1_MOUSE	0.00489	0.00378	0.00786	0.00593	0.00218	0.00460	0.05749	0.09017	0.03711	0.06065	0.08862	0.08692
P52479	UBP10_MOUSE	0.00000	0.00000	0.00000	0.00000	0.00000	0.00000	0.00000	0.00000	0.00000	0.00000	0.00000	0.00130
P52480	KPYM_MOUSE	0.43077	0.36746	0.45472	0.55320	0.45139	0.57538	0.29651	0.32512	0.29458	0.24150	0.25599	0.29490
P52503	NDUS6_MOUSE	0.00000	0.00000	0.00000	0.00000	0.00000	0.00000	0.00000	0.00000	0.00000	0.00080	0.00000	0.00000
P52624	UPP1_MOUSE	0.00000	0.00000	0.00000	0.00000	0.00000	0.00000	0.00000	0.00238	0.00478	0.00345	0.00104	0.00142
P52633	STAT6_MOUSE	0.00000	0.00000	0.00000	0.00000	0.00000	0.00000	0.00000	0.00110	0.00000	0.00018	0.00000	0.00031
P52760	RIDA_MOUSE	0.00000	0.00000	0.00000	0.00000	0.00000	0.00000	0.00909	0.00517	0.00988	0.00799	0.01184	0.00711
P52795	EFNB1_MOUSE	0.00000	0.00000	0.00000	0.00000	0.00000	0.00000	0.00324	0.00325	0.00608	0.00377	0.00235	0.00596

P52800	EFNB2_MOUSE	0.00000	0.00000	0.00000	0.00000	0.00000	0.00000	0.00038	0.00158	0.00222	0.00149	0.00152	0.00161
P52825	CPT2_MOUSE	0.09979	0.11340	0.11236	0.08301	0.10937	0.07080	0.00972	0.00563	0.00689	0.00500	0.00419	0.00499
P52840	ST1A1_MOUSE	0.00000	0.00000	0.00000	0.00000	0.00000	0.00000	0.00122	0.00545	0.00060	0.00260	0.00187	0.00252
P52875	TM165_MOUSE	0.00000	0.00000	0.00000	0.00000	0.00000	0.00000	0.00000	0.00169	0.00223	0.00000	0.00102	0.00182
P53026	RL10A_MOUSE	0.00253	0.00394	0.00233	0.00216	0.00264	0.00240	0.02770	0.02244	0.02700	0.02398	0.02668	0.02698
P53395	ODB2_MOUSE	0.01160	0.01131	0.01973	0.00793	0.01150	0.01153	0.00880	0.00546	0.00621	0.00394	0.00624	0.00674
P53657	KPYR_MOUSE	0.00000	0.00000	0.00000	0.00000	0.00000	0.00000	0.00000	0.00000	0.00000	0.00036	0.00252	0.00000
P53702	CCHL_MOUSE	0.00126	0.00057	0.00161	0.00103	0.00160	0.00052	0.00000	0.00114	0.00107	0.00049	0.00042	0.00033
P53810	PIPNA_MOUSE	0.00262	0.00376	0.00268	0.00199	0.00259	0.00346	0.02637	0.03463	0.03712	0.03778	0.03592	0.03850
P53811	PIPNB_MOUSE	0.00000	0.00000	0.00000	0.00000	0.00000	0.00000	0.00552	0.00384	0.00796	0.00649	0.00790	0.00493
P53986	MOT1_MOUSE	0.00000	0.00000	0.00000	0.00000	0.00000	0.00000	0.00882	0.00507	0.01071	0.00720	0.00563	0.00284
P53994	RAB2A_MOUSE	0.01139	0.01496	0.01140	0.01389	0.01225	0.01573	0.03808	0.05024	0.04217	0.04430	0.04745	0.04945
P54071	IDHP_MOUSE	0.61458	0.87481	0.71396	0.59674	0.67474	0.67554	0.14892	0.11325	0.11064	0.10690	0.12865	0.12194
P54116	STOM_MOUSE	0.01807	0.02271	0.01344	0.00878	0.01840	0.01764	0.01247	0.02520	0.02154	0.02773	0.01771	0.01984
P54227	STMN1_MOUSE	0.00000	0.00000	0.00000	0.00000	0.00000	0.00000	0.00950	0.00608	0.00207	0.00573	0.00144	0.00593
P54310	LIPS_MOUSE	0.00000	0.00000	0.00000	0.00000	0.00000	0.00000	0.01097	0.00379	0.00934	0.00000	0.00000	0.00087
P54320	ELN_MOUSE	0.00000	0.00000	0.00000	0.00000	0.00000	0.00000	0.45415	0.31290	0.33250	0.21603	0.37546	0.39840
P54726	RD23A_MOUSE	0.00000	0.00000	0.00000	0.00000	0.00000	0.00000	0.00000	0.00126	0.00221	0.00308	0.00270	0.00100
P54728	RD23B_MOUSE	0.00000	0.00000	0.00000	0.00000	0.00000	0.00000	0.00326	0.12470	0.32742	0.11251	0.05099	0.10429
P54729	NUB1_MOUSE	0.00000	0.00000	0.00000	0.00000	0.00000	0.00000	0.00000	0.00186	0.00511	0.00341	0.00238	0.00386
P54731	FAF1_MOUSE	0.00000	0.00000	0.00000	0.00000	0.00000	0.00000	0.00072	0.00241	0.00187	0.00000	0.00108	0.00409
P54761	EPHB4_MOUSE	0.00000	0.00000	0.00000	0.00000	0.00000	0.00000	0.00828	0.00625	0.00812	0.00714	0.00615	0.01185
P54775	PRS6B_MOUSE	0.00991	0.00770	0.00819	0.00864	0.00855	0.00711	0.02356	0.01791	0.03230	0.02270	0.03865	0.02373
P54797	TNG2_MOUSE	0.00000	0.00000	0.00000	0.00000	0.00000	0.00000	0.00000	0.00000	0.00000	0.00000	0.00069	0.00000
P54822	PUR8_MOUSE	0.00401	0.00537	0.00354	0.00339	0.00374	0.00327	0.01529	0.00840	0.01467	0.01213	0.01888	0.01162
P54823	DDX6_MOUSE	0.00167	0.00133	0.00038	0.00093	0.00075	0.00044	0.00855	0.00941	0.02013	0.01460	0.01674	0.01170
P54869	HMCS2_MOUSE	0.00000	0.00000	0.00000	0.00000	0.00000	0.00000	0.00197	0.00159	0.00821	0.00581	0.00698	0.00457
P54923	ADPRH_MOUSE	0.00000	0.00000	0.00000	0.00000	0.00000	0.00000	0.00000	0.00107	0.00263	0.00274	0.00569	0.00185
P55012	S12A2_MOUSE	0.00000	0.00000	0.00000	0.00000	0.00000	0.00000	0.00025	0.00174	0.00303	0.00076	0.00000	0.00189
P55014	S12A1_MOUSE	0.00000	0.00000	0.00000	0.00000	0.00000	0.00000	0.00000	0.00000	0.00000	0.00000	0.00000	0.00000
P55065	PLTP_MOUSE	0.00000	0.00000	0.00000	0.00000	0.00000	0.00000	0.00813	0.00667	0.01019	0.00913	0.01421	0.01085
P55096	ABCD3_MOUSE	0.00323	0.00728	0.00408	0.00281	0.00522	0.00498	0.02152	0.02318	0.03027	0.02219	0.01468	0.01549
P55194	3BP1_MOUSE	0.00000	0.00000	0.00000	0.00000	0.00000	0.00000	0.00449	0.00830	0.00722	0.00892	0.00577	0.01087
P55258	RAB8A_MOUSE	0.00000	0.00000	0.00000	0.00000	0.00000	0.00000	0.00912	0.00818	0.01174	0.00947	0.00842	0.01167

P55264	ADK_MOUSE	0.01002	0.01502	0.01859	0.02416	0.01697	0.00587	0.01658	0.03928	0.04802	0.02969	0.02645	0.02832
P55284	CADH5_MOUSE	0.00446	0.00659	0.00585	0.02353	0.00440	0.00609	0.03501	0.05556	0.04970	0.04488	0.02152	0.05788
P55288	CAD11_MOUSE	0.00000	0.00000	0.00000	0.00000	0.00000	0.00000	0.00085	0.00262	0.00488	0.00356	0.00170	0.00520
P55302	AMRP_MOUSE	0.00000	0.00000	0.00000	0.00000	0.00000	0.00000	0.00162	0.01204	0.01167	0.00804	0.00991	0.01183
P55772	ENTP1_MOUSE	0.00000	0.00000	0.00000	0.00000	0.00000	0.00000	0.02959	0.04616	0.04025	0.03987	0.04450	0.04848
P55937	GOGA3_MOUSE	0.00000	0.00000	0.00000	0.00000	0.00000	0.00000	0.00279	0.00903	0.01295	0.00999	0.01039	0.00671
P56135	ATPK_MOUSE	0.00000	0.00000	0.00000	0.00000	0.00000	0.00000	0.04283	0.02913	0.03402	0.02590	0.02486	0.02298
P56212	ARP19_MOUSE	0.00000	0.00000	0.00000	0.00000	0.00000	0.00000	0.00017	0.00394	0.00495	0.00705	0.00790	0.00221
P56376	ACYP1_MOUSE	0.00000	0.00000	0.00000	0.00000	0.00000	0.00000	0.00146	0.00391	0.00000	0.00000	0.00162	0.00207
P56379	ATP68_MOUSE	0.00000	0.00000	0.00000	0.00000	0.00000	0.00000	0.00000	0.00757	0.00523	0.00703	0.00270	0.00445
P56380	AP4A_MOUSE	0.01173	0.00928	0.01375	0.01162	0.01146	0.01374	0.00211	0.00179	0.00195	0.00232	0.00000	0.00531
P56389	CDD_MOUSE	0.00000	0.00000	0.00000	0.00000	0.00000	0.00000	0.00014	0.00000	0.00000	0.00003	0.00013	0.00022
P56392	CX7A1_MOUSE	0.00000	0.00000	0.00000	0.00000	0.00000	0.00000	0.01892	0.00730	0.00586	0.00858	0.00681	0.00458
P56395	CYB5_MOUSE	0.00000	0.00000	0.00000	0.00000	0.00000	0.00000	0.06229	0.09149	0.11009	0.09514	0.11971	0.07580
P56399	UBP5_MOUSE	0.03669	0.04161	0.04412	0.05508	0.05043	0.04515	0.03014	0.01932	0.03101	0.02917	0.03232	0.03105
P56400	GP1BB_MOUSE	0.00000	0.00000	0.00000	0.00000	0.00000	0.00000	0.00000	0.00000	0.00000	0.00035	0.00000	0.00000
P56480	ATPB_MOUSE	4.31607	5.38821	5.33497	4.67573	5.36576	6.76124	0.24944	0.27108	0.24245	0.26470	0.25572	0.26115
P56528	CD38_MOUSE	0.00000	0.00000	0.00000	0.00000	0.00000	0.00000	0.01888	0.00915	0.02015	0.01124	0.02039	0.02040
P56546	CTBP2_MOUSE	0.00000	0.00000	0.00000	0.00000	0.00000	0.00000	0.00439	0.00303	0.00523	0.00390	0.00521	0.00339
P56565	S10A1_MOUSE	0.00000	0.00000	0.00000	0.00000	0.00000	0.00000	0.00000	0.00000	0.00000	0.01796	0.00000	0.00000
P56695	WFS1_MOUSE	0.00000	0.00000	0.00000	0.00000	0.00000	0.00000	0.00000	0.00881	0.00721	0.00325	0.00665	0.00187
P56812	PDCD5_MOUSE	0.00000	0.00000	0.00000	0.00000	0.00000	0.00000	0.00466	0.00429	0.00365	0.00642	0.00462	0.00476
P56857	CLD18_MOUSE	0.00000	0.00000	0.00000	0.00000	0.00000	0.00000	0.00003	0.01924	0.02263	0.01673	0.01245	0.00900
P56959	FUS_MOUSE	0.00000	0.00000	0.00000	0.00000	0.00000	0.00000	0.01177	0.02332	0.03104	0.02739	0.02273	0.02232
P57016	LAD1_MOUSE	0.00000	0.00000	0.00000	0.00000	0.00000	0.00000	0.00000	0.00000	0.00000	0.00000	0.00000	0.00000
P57716	NICA_MOUSE	0.00000	0.00000	0.00000	0.00000	0.00000	0.00000	0.00114	0.00471	0.00917	0.00549	0.01391	0.01161
P57746	VATD_MOUSE	0.00000	0.00000	0.00000	0.00000	0.00000	0.00000	0.00362	0.00763	0.00679	0.00613	0.00629	0.00747
P57759	ERP29_MOUSE	0.00000	0.00000	0.00000	0.00000	0.00000	0.00000	0.03125	0.01213	0.01954	0.01396	0.01542	0.01790
P57776	EF1D_MOUSE	0.00603	0.00444	0.00467	0.00145	0.00827	0.00459	0.02220	0.03204	0.03351	0.03485	0.02955	0.03294
P57780	ACTN4_MOUSE	0.01978	0.02020	0.01905	0.01782	0.01883	0.02731	0.19523	0.21214	0.23587	0.21841	0.21247	0.21378
P57784	RU2A_MOUSE	0.00000	0.00000	0.00000	0.00000	0.00000	0.00000	0.01038	0.00946	0.00428	0.00600	0.00394	0.00856
P58021	TM9S2_MOUSE	0.00326	0.00286	0.00219	0.00242	0.00302	0.00242	0.01204	0.00907	0.00976	0.00967	0.01006	0.00983
P58044	IDI1_MOUSE	0.00000	0.00000	0.00000	0.00000	0.00000	0.00000	0.00329	0.01171	0.01029	0.00532	0.01513	0.01085
P58059	RT21_MOUSE	0.00000	0.00000	0.00000	0.00000	0.00000	0.00000	0.00000	0.00040	0.00000	0.00000	0.00000	0.00000

P58196	PLS4_MOUSE	0.00000	0.00000	0.00000	0.00000	0.00000	0.00000	0.00383	0.01304	0.00535	0.00490	0.00468	0.01017
P58252	EF2_MOUSE	0.10781	0.07127	0.11574	0.07261	0.09350	0.08400	0.07515	0.07924	0.07580	0.07577	0.06369	0.07710
P58281	OPA1_MOUSE	0.09433	0.06611	0.09242	0.06297	0.08465	0.07022	0.01493	0.00874	0.01434	0.01326	0.01211	0.00967
P58389	PTPA_MOUSE	0.00000	0.00000	0.00000	0.00000	0.00000	0.00000	0.00880	0.01067	0.01050	0.01216	0.00820	0.01390
P58465	CTDSL_MOUSE	0.00000	0.00000	0.00000	0.00000	0.00000	0.00000	0.00308	0.00395	0.00526	0.00434	0.00445	0.00523
P58735	S26A1_MOUSE	0.00000	0.00000	0.00000	0.00000	0.00000	0.00000	0.00000	0.00000	0.00000	0.00000	0.00000	0.00000
P58771	TPM1_MOUSE	0.80484	1.00498	0.83829	1.15520	0.79004	1.71158	0.41769	0.21665	0.21082	0.24089	0.17055	0.15474
P58774	TPM2_MOUSE	0.00024	0.00080	0.00051	0.00054	0.00060	0.00144	0.08415	0.04536	0.04251	0.05255	0.03971	0.03365
P58871	TB182_MOUSE	0.00000	0.00000	0.00000	0.00000	0.00000	0.00000	0.01533	0.01910	0.02376	0.02400	0.02398	0.02671
P59017	B2L13_MOUSE	0.02348	0.01764	0.02322	0.02830	0.02687	0.01970	0.00612	0.00351	0.00367	0.00307	0.00430	0.00432
P59114	CAPAM_MOUSE	0.00000	0.00000	0.00000	0.00000	0.00000	0.00000	0.00000	0.00101	0.00214	0.00086	0.00272	0.00255
P59158	S12A3_MOUSE	0.00000	0.00000	0.00000	0.00000	0.00000	0.00000	0.00037	0.00000	0.00018	0.00113	0.00000	0.00090
P59242	CING_MOUSE	0.00000	0.00000	0.00000	0.00000	0.00000	0.00000	0.01453	0.01369	0.02457	0.01963	0.02364	0.01712
P59279	RAB2B_MOUSE	0.00000	0.00000	0.00000	0.00000	0.00000	0.00000	0.00000	0.00000	0.00000	0.00000	0.00000	0.00000
P59325	IF5_MOUSE	0.00000	0.00000	0.00000	0.00000	0.00000	0.00000	0.01351	0.00652	0.01217	0.01027	0.01531	0.00925
P59326	YTHD1_MOUSE	0.00000	0.00000	0.00000	0.00000	0.00000	0.00000	0.00000	0.00194	0.00415	0.00234	0.00390	0.00244
P59383	LRRN4_MOUSE	0.00000	0.00000	0.00000	0.00000	0.00000	0.00000	0.00000	0.00388	0.00000	0.00000	0.00000	0.00000
P59481	LMA2L_MOUSE	0.00000	0.00000	0.00000	0.00000	0.00000	0.00000	0.00117	0.00088	0.00178	0.00175	0.00000	0.00000
P59672	ANS1A_MOUSE	0.00000	0.00000	0.00000	0.00000	0.00000	0.00000	0.00467	0.00387	0.00881	0.00291	0.00000	0.00454
P59708	SF3B6_MOUSE	0.00000	0.00000	0.00000	0.00000	0.00000	0.00000	0.01319	0.01069	0.00602	0.01118	0.00513	0.01454
P59729	RIN3_MOUSE	0.00000	0.00000	0.00000	0.00000	0.00000	0.00000	0.00175	0.00166	0.00190	0.00180	0.00000	0.00257
P59764	DOCK4_MOUSE	0.00000	0.00000	0.00000	0.00000	0.00000	0.00000	0.00925	0.00591	0.01125	0.00793	0.01187	0.02198
P59999	ARPC4_MOUSE	0.00000	0.00000	0.00000	0.00000	0.00000	0.00000	0.02094	0.03580	0.02288	0.02403	0.01989	0.02737
P60060	SC61G_MOUSE	0.00000	0.00000	0.00000	0.00000	0.00000	0.00000	0.00000	0.00167	0.00035	0.00000	0.00000	0.00042
P60122	RUVB1_MOUSE	0.00000	0.00000	0.00000	0.00000	0.00000	0.00000	0.01059	0.01264	0.01330	0.00879	0.01399	0.01841
P60229	EIF3E_MOUSE	0.00000	0.00000	0.00000	0.00000	0.00000	0.00000	0.01500	0.01459	0.01504	0.01465	0.01343	0.01759
P60335	PCBP1_MOUSE	0.01542	0.01280	0.00689	0.01001	0.00921	0.00830	0.01350	0.02220	0.01933	0.01800	0.01433	0.02020
P60605	UB2G2_MOUSE	0.00000	0.00000	0.00000	0.00000	0.00000	0.00000	0.00025	0.00910	0.00226	0.00630	0.00000	0.00393
P60670	NPL4_MOUSE	0.00707	0.00250	0.00417	0.00399	0.00611	0.00570	0.01958	0.00681	0.01360	0.00672	0.00900	0.01035
P60710	ACTB_MOUSE	0.07968	0.05744	0.08021	0.05561	0.09549	0.09573	1.59983	1.81578	1.94920	1.80117	1.90440	1.79404
P60766	CDC42_MOUSE	0.00000	0.00000	0.00000	0.00000	0.00000	0.00000	0.04033	0.02258	0.02849	0.02783	0.02921	0.03393
P60824	CIRBP_MOUSE	0.00000	0.00000	0.00000	0.00000	0.00000	0.00000	0.00289	0.00573	0.00376	0.00459	0.00315	0.00770
P60843	IF4A1_MOUSE	0.00419	0.00159	0.00315	0.00236	0.00296	0.00177	0.06058	0.06845	0.07735	0.08074	0.08517	0.07151
P60867	RS20_MOUSE	0.00000	0.00000	0.00000	0.00000	0.00000	0.00000	0.01231	0.02340	0.02691	0.02513	0.01806	0.01986

P60904	DNJC5_MOUSE	0.00000	0.00000	0.00000	0.00000	0.00000	0.00000	0.00667	0.00920	0.01328	0.01062	0.01460	0.01352
P61021	RAB5B_MOUSE	0.00000	0.00000	0.00000	0.00000	0.00000	0.00000	0.01455	0.02723	0.02764	0.02321	0.03036	0.02880
P61022	CHP1_MOUSE	0.00000	0.00000	0.00000	0.00000	0.00000	0.00000	0.01330	0.00842	0.00775	0.01099	0.01004	0.01481
P61027	RAB10_MOUSE	0.00000	0.00000	0.00000	0.00000	0.00000	0.00000	0.02507	0.02253	0.03639	0.02529	0.02914	0.03969
P61028	RAB8B_MOUSE	0.00000	0.00000	0.00000	0.00000	0.00000	0.00000	0.00189	0.01058	0.00899	0.00476	0.00729	0.00709
P61082	UBC12_MOUSE	0.00000	0.00000	0.00000	0.00000	0.00000	0.00000	0.00000	0.00533	0.00739	0.00675	0.00265	0.00261
P61087	UBE2K_MOUSE	0.00000	0.00000	0.00000	0.00000	0.00000	0.00000	0.01668	0.01245	0.01003	0.01074	0.00802	0.01087
P61089	UBE2N_MOUSE	0.00000	0.00000	0.00000	0.00000	0.00000	0.00000	0.03912	0.05064	0.03110	0.03889	0.03682	0.03742
P61110	ANRE_MOUSE	0.00000	0.00000	0.00000	0.00000	0.00000	0.00000	0.00000	0.00000	0.00000	0.00000	0.00000	0.00000
P61148	FGF1_MOUSE	0.00000	0.00000	0.00000	0.00000	0.00000	0.00000	0.00489	0.00560	0.00932	0.00718	0.00643	0.00949
P61161	ARP2_MOUSE	0.00747	0.00755	0.00668	0.00512	0.00668	0.00742	0.03931	0.04687	0.04906	0.04290	0.04009	0.03830
P61164	ACTZ_MOUSE	0.01106	0.00943	0.01416	0.00516	0.00877	0.01741	0.04542	0.05902	0.03992	0.04665	0.04736	0.04973
P61202	CSN2_MOUSE	0.00338	0.00702	0.00463	0.00681	0.00668	0.00689	0.00795	0.00765	0.01043	0.00975	0.00945	0.00901
P84078	ARF1_MOUSE	0.05361	0.03857	0.03631	0.02356	0.06307	0.06101	0.15273	0.21022	0.15840	0.20333	0.19159	0.19689
P61211	ARL1_MOUSE	0.00164	0.00087	0.00217	0.00213	0.00213	0.00226	0.00801	0.01406	0.00936	0.00976	0.01141	0.00844
P61222	ABCE1_MOUSE	0.00541	0.00692	0.00438	0.00320	0.00380	0.00325	0.01814	0.01661	0.02249	0.01876	0.02729	0.02389
P61226	RAP2B_MOUSE	0.00000	0.00000	0.00000	0.00000	0.00000	0.00000	0.00062	0.00000	0.00191	0.00116	0.00137	0.00181
P61255	RL26_MOUSE	0.00000	0.00000	0.00000	0.00000	0.00000	0.00000	0.00000	0.00632	0.00523	0.00436	0.00263	0.00273
Q9CQL1	MGN2_MOUSE	0.00000	0.00000	0.00000	0.00000	0.00000	0.00000	0.03081	0.02255	0.03446	0.02705	0.03461	0.02530
P61358	RL27_MOUSE	0.00000	0.00000	0.00000	0.00000	0.00000	0.00000	0.02320	0.02404	0.03192	0.02902	0.02569	0.02782
P61458	PHS_MOUSE	0.00000	0.00000	0.00000	0.00000	0.00000	0.00000	0.00000	0.00000	0.00000	0.00000	0.00000	0.00000
P61514	RL37A_MOUSE	0.00000	0.00000	0.00000	0.00000	0.00000	0.00000	0.05011	0.03012	0.02937	0.03235	0.03946	0.03440
P61620	S61A1_MOUSE	0.00000	0.00000	0.00000	0.00000	0.00000	0.00000	0.00000	0.00903	0.00196	0.01279	0.00252	0.00558
P61750	ARF4_MOUSE	0.00000	0.00000	0.00000	0.00000	0.00000	0.00000	0.01572	0.01713	0.00825	0.01371	0.01703	0.00850
P61759	PFD3_MOUSE	0.00000	0.00000	0.00000	0.00000	0.00000	0.00000	0.00499	0.00797	0.00642	0.00620	0.01271	0.00770
P61804	DAD1_MOUSE	0.00000	0.00000	0.00000	0.00000	0.00000	0.00000	0.01458	0.02094	0.00673	0.01624	0.00433	0.01822
P61922	GABT_MOUSE	0.00512	0.00292	0.00400	0.00528	0.00446	0.00227	0.00000	0.00007	0.00010	0.00000	0.00011	0.00015
P61924	COPZ1_MOUSE	0.00000	0.00000	0.00000	0.00000	0.00000	0.00000	0.01601	0.01395	0.01645	0.01759	0.02346	0.01895
P61939	THBG_MOUSE	0.00000	0.00000	0.00000	0.00000	0.00000	0.00000	0.00000	0.00000	0.00022	0.00344	0.00000	0.00112
P61953	GBG11_MOUSE	0.00000	0.00000	0.00000	0.00000	0.00000	0.00000	0.00000	0.00000	0.00345	0.00221	0.00130	0.00117
P61957	SUMO2_MOUSE	0.00000	0.00000	0.00000	0.00000	0.00000	0.00000	0.00000	0.02164	0.01922	0.03568	0.01660	0.01962
P61961	UFM1_MOUSE	0.00000	0.00000	0.00000	0.00000	0.00000	0.00000	0.01262	0.00599	0.01598	0.00898	0.01710	0.00887
P61965	WDR5_MOUSE	0.00000	0.00000	0.00000	0.00000	0.00000	0.00000	0.00129	0.00334	0.00238	0.00318	0.00281	0.00413
P61967	AP1S1_MOUSE	0.00000	0.00000	0.00000	0.00000	0.00000	0.00000	0.00431	0.00312	0.00700	0.01023	0.00350	0.00950

P61971	NTF2_MOUSE	0.01119	0.00648	0.00544	0.00729	0.00774	0.00836	0.01016	0.00664	0.01488	0.00557	0.01676	0.00618
P61979	HNRPK_MOUSE	0.01870	0.01007	0.01488	0.01520	0.01697	0.01183	0.15436	0.13408	0.13988	0.16367	0.16051	0.14035
P61982	1433G_MOUSE	0.04350	0.03864	0.03056	0.03717	0.03641	0.03948	0.04570	0.05465	0.09661	0.08891	0.08112	0.12294
P62046	LRCH1_MOUSE	0.00000	0.00000	0.00000	0.00000	0.00000	0.00000	0.00611	0.00738	0.01098	0.00636	0.00776	0.01019
P62071	RRAS2_MOUSE	0.02014	0.01926	0.01802	0.01411	0.01593	0.02009	0.00786	0.00259	0.00958	0.00694	0.01006	0.00817
P62075	TIM13_MOUSE	0.00000	0.00000	0.00000	0.00000	0.00000	0.00000	0.00194	0.00084	0.00053	0.00042	0.00049	0.00020
P62082	RS7_MOUSE	0.00000	0.00000	0.00000	0.00000	0.00000	0.00000	0.00840	0.01193	0.01665	0.01645	0.02557	0.02212
P62137	PP1A_MOUSE	0.00000	0.00000	0.00000	0.00000	0.00000	0.00000	0.00758	0.03123	0.01939	0.01451	0.01420	0.01430
P62141	PP1B_MOUSE	0.02897	0.02544	0.02062	0.02291	0.02330	0.02277	0.07390	0.06929	0.08481	0.06985	0.07196	0.07062
P62192	PRS4_MOUSE	0.00734	0.00513	0.00755	0.00596	0.00828	0.00633	0.03309	0.02291	0.02596	0.02513	0.02584	0.02416
P62196	PRS8_MOUSE	0.01247	0.02170	0.01441	0.01243	0.01210	0.01036	0.01758	0.01845	0.02166	0.01982	0.01715	0.02172
P62242	RS8_MOUSE	0.02010	0.02809	0.01983	0.01721	0.01663	0.01950	0.04803	0.05432	0.02830	0.04699	0.03073	0.05036
P62245	RS15A_MOUSE	0.00244	0.00214	0.00128	0.00143	0.00148	0.00047	0.06897	0.05706	0.07590	0.07518	0.09097	0.05376
P62254	UB2G1_MOUSE	0.00000	0.00000	0.00000	0.00000	0.00000	0.00000	0.01101	0.00296	0.00274	0.00318	0.00200	0.00599
P62257	UBE2H_MOUSE	0.00000	0.00000	0.00000	0.00000	0.00000	0.00000	0.00221	0.00315	0.00265	0.00111	0.00291	0.00243
P62259	1433E_MOUSE	0.03061	0.06485	0.06727	0.08173	0.07620	0.07016	0.19596	0.31086	0.27559	0.29950	0.33465	0.33445
P62264	RS14_MOUSE	0.01499	0.03079	0.01979	0.01060	0.01756	0.01417	0.03727	0.04698	0.03104	0.05120	0.02854	0.04055
P62267	RS23_MOUSE	0.00000	0.00000	0.00000	0.00000	0.00000	0.00000	0.00442	0.00883	0.00464	0.00569	0.00402	0.00600
P62270	RS18_MOUSE	0.00000	0.00000	0.00000	0.00000	0.00000	0.00000	0.19336	0.10579	0.14711	0.12416	0.11996	0.11498
P62274	RS29_MOUSE	0.00000	0.00000	0.00000	0.00000	0.00000	0.00000	0.00000	0.00770	0.00876	0.00548	0.00205	0.00183
P62281	RS11_MOUSE	0.00000	0.00000	0.00000	0.00000	0.00000	0.00000	0.01933	0.02183	0.02992	0.02162	0.01952	0.02076
P62301	RS13_MOUSE	0.00000	0.00000	0.00000	0.00000	0.00000	0.00000	0.10896	0.02506	0.08470	0.06681	0.09188	0.07776
P62305	RUXE_MOUSE	0.00000	0.00000	0.00000	0.00000	0.00000	0.00000	0.00000	0.00415	0.00262	0.00462	0.00496	0.00678
P62307	RUXF_MOUSE	0.00000	0.00000	0.00000	0.00000	0.00000	0.00000	0.00000	0.00490	0.00839	0.00866	0.00473	0.00418
P62309	RUXG_MOUSE	0.00000	0.00000	0.00000	0.00000	0.00000	0.00000	0.00511	0.00297	0.00298	0.00424	0.00000	0.00000
P62311	LSM3_MOUSE	0.00000	0.00000	0.00000	0.00000	0.00000	0.00000	0.01808	0.01193	0.00565	0.01521	0.00746	0.01769
P62315	SMD1_MOUSE	0.00000	0.00000	0.00000	0.00000	0.00000	0.00000	0.00065	0.00995	0.00691	0.00761	0.00505	0.00871
P62317	SMD2_MOUSE	0.00000	0.00000	0.00000	0.00000	0.00000	0.00000	0.04519	0.04957	0.04334	0.05600	0.07209	0.06449
P62320	SMD3_MOUSE	0.00000	0.00000	0.00000	0.00000	0.00000	0.00000	0.01644	0.01121	0.02268	0.01774	0.02325	0.01991
P62331	ARF6_MOUSE	0.00000	0.00000	0.00000	0.00000	0.00000	0.00000	0.03629	0.03609	0.02465	0.03075	0.03760	0.03310
P62334	PRS10_MOUSE	0.00278	0.00245	0.00459	0.00331	0.00357	0.00298	0.01696	0.01511	0.02148	0.01933	0.02156	0.02061
P62342	SELT_MOUSE	0.00000	0.00000	0.00000	0.00000	0.00000	0.00000	0.00000	0.00205	0.00000	0.00259	0.00000	0.00000
P62627	DLRB1_MOUSE	0.00000	0.00000	0.00000	0.00000	0.00000	0.00000	0.01284	0.00894	0.01278	0.01251	0.00873	0.01001
P62631	EF1A2_MOUSE	0.08332	0.07869	0.16338	0.12659	0.09414	0.06491	0.00110	0.00282	0.00193	0.00403	0.00151	0.00108

P62702	RS4X_MOUSE	0.00766	0.01395	0.01559	0.01158	0.01724	0.01992	0.03185	0.03273	0.04173	0.03893	0.02934	0.04011
P63330	PP2AA_MOUSE	0.00935	0.01574	0.00167	0.00786	0.01529	0.01674	0.04102	0.03790	0.03485	0.03681	0.03037	0.04204
P62717	RL18A_MOUSE	0.00000	0.00000	0.00000	0.00000	0.00000	0.00000	0.01522	0.00931	0.01268	0.01146	0.00906	0.01313
P62737	ACTA_MOUSE	0.00498	0.00593	0.00514	0.00454	0.00364	0.00000	0.03415	0.07125	0.11563	0.06399	0.06984	0.07261
P62743	AP2S1_MOUSE	0.00000	0.00000	0.00000	0.00000	0.00000	0.00000	0.01379	0.02189	0.02809	0.02194	0.01692	0.02193
P62748	HPCL1_MOUSE	0.00000	0.00000	0.00000	0.00000	0.00000	0.00000	0.02591	0.02974	0.03194	0.02505	0.02822	0.02882
P62751	RL23A_MOUSE	0.00432	0.00323	0.00367	0.00313	0.00294	0.00328	0.01729	0.03475	0.04646	0.02779	0.02708	0.04261
P62754	RS6_MOUSE	0.00000	0.00000	0.00000	0.00000	0.00000	0.00000	0.03986	0.05127	0.01907	0.03813	0.01887	0.04339
P62761	VISL1_MOUSE	0.00000	0.00000	0.00000	0.00000	0.00000	0.00000	0.00299	0.00498	0.00472	0.00448	0.00275	0.00404
P62774	MTPN_MOUSE	0.00000	0.00000	0.00000	0.00000	0.00000	0.00000	0.06073	0.05641	0.06549	0.07149	0.06948	0.07231
P62806	H4_MOUSE	0.00000	0.00000	0.00000	0.00000	0.00000	0.00000	0.69637	1.35845	0.82841	1.23400	0.96495	1.43323
P62814	VATB2_MOUSE	0.00217	0.00232	0.00065	0.00169	0.00132	0.00182	0.00986	0.02135	0.01749	0.01618	0.01632	0.01717
P62821	RAB1A_MOUSE	0.01364	0.00959	0.02148	0.02171	0.02923	0.02274	0.10665	0.12118	0.12930	0.14091	0.14363	0.13792
P62827	RAN_MOUSE	0.00000	0.00000	0.00000	0.00000	0.00000	0.00000	0.02625	0.06480	0.08234	0.05138	0.04194	0.07220
P62830	RL23_MOUSE	0.01012	0.01026	0.01612	0.01338	0.01096	0.00756	0.09250	0.09137	0.11648	0.11327	0.10975	0.12480
P62835	RAP1A_MOUSE	0.00705	0.00553	0.00531	0.00981	0.00801	0.00700	0.00409	0.01965	0.01443	0.01466	0.01106	0.01890
P62843	RS15_MOUSE	0.00000	0.00000	0.00000	0.00000	0.00000	0.00000	0.00518	0.02439	0.01237	0.01720	0.00290	0.00721
P62849	RS24_MOUSE	0.00306	0.00812	0.00299	0.00440	0.00531	0.00686	0.04012	0.03345	0.03042	0.03529	0.03093	0.04196
P62852	RS25_MOUSE	0.00000	0.00000	0.00000	0.00000	0.00000	0.00000	0.01078	0.02156	0.00866	0.00876	0.00791	0.01437
P62855	RS26_MOUSE	0.00000	0.00000	0.00000	0.00000	0.00000	0.00000	0.07184	0.05825	0.04659	0.05310	0.05180	0.06241
P62858	RS28_MOUSE	0.00000	0.00000	0.00000	0.00000	0.00000	0.00000	0.03710	0.05891	0.03279	0.06622	0.02697	0.04711
P62862	RS30_MOUSE	0.00000	0.00000	0.00000	0.00000	0.00000	0.00000	0.00000	0.00000	0.00099	0.00000	0.00000	0.00221
P62869	ELOB_MOUSE	0.00000	0.00000	0.00000	0.00000	0.00000	0.00000	0.00245	0.01247	0.01410	0.01521	0.00968	0.01292
P62874	GBB1_MOUSE	0.00737	0.00758	0.00313	0.00377	0.00457	0.00601	0.09569	0.10997	0.11367	0.12488	0.11166	0.09149
P62878	RBX1_MOUSE	0.03640	0.02291	0.04381	0.01962	0.01690	0.02866	0.00000	0.00710	0.00696	0.00983	0.00631	0.00635
P62889	RL30_MOUSE	0.01523	0.01544	0.01008	0.01121	0.01061	0.01342	0.00629	0.00752	0.01434	0.01084	0.00999	0.01006
P62892	RL39_MOUSE	0.00000	0.00000	0.00000	0.00000	0.00000	0.00000	0.00000	0.00104	0.00217	0.00123	0.00121	0.00116
P62897	CYC_MOUSE	0.03084	0.02599	0.04556	0.03556	0.03630	0.04186	0.09600	0.05229	0.05742	0.03821	0.06647	0.04899
P62900	RL31_MOUSE	0.00000	0.00000	0.00000	0.00000	0.00000	0.00000	0.00988	0.02022	0.01353	0.01768	0.01738	0.02118
P62908	RS3_MOUSE	0.00979	0.01636	0.01313	0.00673	0.01251	0.01006	0.04716	0.05115	0.03967	0.04737	0.04913	0.04743
P62911	RL32_MOUSE	0.00000	0.00000	0.00000	0.00000	0.00000	0.00000	0.00000	0.00097	0.00848	0.00470	0.00000	0.00244
P62918	RL8_MOUSE	0.00000	0.00000	0.00000	0.00000	0.00000	0.00000	0.00000	0.01368	0.01287	0.01446	0.01066	0.00800
P62960	YBOX1_MOUSE	0.01244	0.00669	0.00965	0.00974	0.00522	0.00265	0.01738	0.04787	0.07704	0.07065	0.05038	0.04941
P62996	TRA2B_MOUSE	0.00394	0.00468	0.00216	0.00160	0.00346	0.00270	0.00648	0.03280	0.00858	0.00269	0.00233	0.01900

P63001	RAC1_MOUSE	0.00430	0.01208	0.00155	0.00425	0.00541	0.00495	0.09248	0.14427	0.10790	0.11853	0.13331	0.13769
P63005	LIS1_MOUSE	0.00307	0.00276	0.00473	0.00357	0.00314	0.00183	0.01366	0.03024	0.04183	0.03323	0.03743	0.03041
P63011	RAB3A_MOUSE	0.00827	0.00597	0.00848	0.00600	0.00585	0.00599	0.00801	0.00908	0.00844	0.00768	0.00331	0.00575
P63017	HSP7C_MOUSE	0.12938	0.16339	0.13238	0.15860	0.12005	0.23976	0.20312	0.22026	0.19980	0.23209	0.17106	0.22795
P63024	VAMP3_MOUSE	0.00144	0.00091	0.00122	0.00187	0.00187	0.00145	0.04208	0.04512	0.03968	0.04091	0.03619	0.05323
P63028	TCTP_MOUSE	0.00000	0.00000	0.00000	0.00000	0.00000	0.00000	0.07804	0.07893	0.05563	0.07024	0.06381	0.07329
P63037	DNJA1_MOUSE	0.00000	0.00000	0.00000	0.00000	0.00000	0.00000	0.01399	0.01359	0.01680	0.01898	0.01890	0.01476
P63038	CH60_MOUSE	0.41886	0.26068	0.29535	0.25132	0.37506	0.32831	0.06775	0.06855	0.05807	0.05719	0.06879	0.07397
P63044	VAMP2_MOUSE	0.00205	0.00116	0.00178	0.00085	0.00229	0.00306	0.00531	0.01863	0.00540	0.00900	0.00716	0.01473
P63073	IF4E_MOUSE	0.00222	0.00075	0.00116	0.00162	0.00080	0.00035	0.00130	0.00549	0.00577	0.00485	0.00366	0.00461
P63082	VATL_MOUSE	0.00000	0.00000	0.00000	0.00000	0.00000	0.00000	0.00000	0.00394	0.00000	0.00000	0.00000	0.00212
P63085	MK01_MOUSE	0.00145	0.00338	0.00242	0.00390	0.00338	0.00262	0.01471	0.01498	0.01574	0.01643	0.01265	0.01647
P63087	PP1G_MOUSE	0.00000	0.00000	0.00000	0.00000	0.00000	0.00000	0.01234	0.00921	0.01044	0.00974	0.00425	0.01203
Q6R0H7	GNAS1_MOUSE	0.00698	0.01094	0.00882	0.00457	0.00854	0.00592	0.02981	0.02153	0.03141	0.02624	0.03046	0.02620
P63101	1433Z_MOUSE	0.00000	0.00000	0.00000	0.00000	0.00000	0.00000	0.20002	0.47607	0.54544	0.47292	0.39766	0.36723
P63158	HMGB1_MOUSE	0.00000	0.00000	0.00000	0.00000	0.00000	0.00000	0.06377	0.07585	0.12059	0.09667	0.10239	0.09899
P63166	SUMO1_MOUSE	0.00000	0.00000	0.00000	0.00000	0.00000	0.00000	0.00000	0.00105	0.00253	0.00193	0.00122	0.00066
Q9D0M5	DYL2_MOUSE	0.00000	0.00000	0.00000	0.00000	0.00000	0.00000	0.00303	0.02494	0.04123	0.03520	0.02138	0.02099
P63213	GBG2_MOUSE	0.00000	0.00000	0.00000	0.00000	0.00000	0.00000	0.00000	0.02372	0.01247	0.02486	0.01159	0.02118
P63242	IF5A1_MOUSE	0.00696	0.00116	0.00528	0.00472	0.00465	0.00165	0.03092	0.02445	0.03492	0.02986	0.03126	0.02740
P63268	ACTH_MOUSE	0.00000	0.00000	0.00000	0.00000	0.00000	0.00000	0.00000	0.00062	0.00452	0.00142	0.00090	0.00092
P63276	RS17_MOUSE	0.00000	0.00000	0.00000	0.00000	0.00000	0.00000	0.06774	0.02778	0.02992	0.02427	0.04085	0.02845
P63280	UBC9_MOUSE	0.00000	0.00000	0.00000	0.00000	0.00000	0.00000	0.00723	0.00811	0.00787	0.00637	0.00607	0.00828
P63321	RALA_MOUSE	0.00000	0.00000	0.00000	0.00000	0.00000	0.00000	0.02622	0.03347	0.02504	0.02706	0.02357	0.02828
P63323	RS12_MOUSE	0.00000	0.00000	0.00000	0.00000	0.00000	0.00000	0.00558	0.00719	0.01116	0.00991	0.01405	0.00980
P63325	RS10_MOUSE	0.00000	0.00000	0.00000	0.00000	0.00000	0.00000	0.01945	0.02121	0.02155	0.01524	0.02602	0.03749
P63328	PP2BA_MOUSE	0.00000	0.00000	0.00000	0.00000	0.00000	0.00000	0.01161	0.02153	0.02337	0.02240	0.01933	0.02215
P67778	PHB_MOUSE	0.04984	0.05511	0.03964	0.03842	0.04607	0.04095	0.02116	0.02198	0.02723	0.02267	0.03212	0.02385
P67871	CSK2B_MOUSE	0.00000	0.00000	0.00000	0.00000	0.00000	0.00000	0.00494	0.00468	0.00554	0.00290	0.00543	0.00584
P67984	RL22_MOUSE	0.00094	0.00325	0.00035	0.00068	0.00375	0.00371	0.00585	0.03306	0.01558	0.02058	0.00759	0.02159
P68033	ACTC_MOUSE	0.00000	0.00000	0.00000	0.00000	0.00000	0.00000	0.50556	0.42212	0.29661	0.39543	0.27352	0.42147
P68037	UB2L3_MOUSE	0.00000	0.00000	0.00000	0.00000	0.00000	0.00000	0.03145	0.02015	0.03392	0.02828	0.03971	0.03433
P68040	RACK1_MOUSE	0.00657	0.00511	0.00656	0.00799	0.00801	0.00623	0.03607	0.05118	0.02851	0.04094	0.03549	0.03478
P68134	ACTS_MOUSE	0.00000	0.00000	0.00000	0.00000	0.00000	0.00000	2.68769	0.95659	1.07257	1.15723	1.33753	0.99757

P68181	KAPCB_MOUSE	0.00000	0.00000	0.00000	0.00000	0.00000	0.00000	0.00101	0.00691	0.00752	0.00458	0.00732	0.00766
P68254	1433T_MOUSE	0.00000	0.00000	0.00000	0.00000	0.00000	0.00000	0.07252	0.13770	0.13525	0.11463	0.11937	0.11661
P68368	TBA4A_MOUSE	0.13004	0.16767	0.11158	0.07652	0.09359	0.11236	0.07983	0.03013	0.05601	0.06386	0.05642	0.05537
P68372	TBB4B_MOUSE	0.00000	0.00000	0.00000	0.00000	0.00000	0.00000	0.48016	0.50426	0.50824	0.53826	0.48460	0.56309
P68373	TBA1C_MOUSE	0.00000	0.00000	0.00000	0.00000	0.00000	0.00000	0.00000	0.00209	0.00000	0.00616	0.00000	0.00086
P68404	KPCB_MOUSE	0.00000	0.00000	0.00000	0.00000	0.00000	0.00000	0.00247	0.00151	0.00723	0.00471	0.00439	0.00275
P68510	1433F_MOUSE	0.00876	0.01645	0.01709	0.00874	0.02673	0.00995	0.03314	0.08388	0.10497	0.11831	0.06456	0.07127
P70122	SBDS_MOUSE	0.00000	0.00000	0.00000	0.00000	0.00000	0.00000	0.01597	0.01175	0.01676	0.01315	0.01765	0.01333
P70158	ASM3A_MOUSE	0.00000	0.00000	0.00000	0.00000	0.00000	0.00000	0.00219	0.00224	0.00444	0.00312	0.00403	0.00241
P70168	IMB1_MOUSE	0.01130	0.01085	0.01773	0.02163	0.01320	0.01550	0.02558	0.02161	0.03298	0.02434	0.03570	0.02569
P70195	PSB7_MOUSE	0.00000	0.00000	0.00000	0.00000	0.00000	0.00000	0.00000	0.00104	0.00588	0.00470	0.00106	0.00100
P70202	LXN_MOUSE	0.00000	0.00000	0.00000	0.00000	0.00000	0.00000	0.01580	0.01633	0.01332	0.01951	0.01535	0.01969
P70227	ITPR3_MOUSE	0.00000	0.00000	0.00000	0.00000	0.00000	0.00000	0.00000	0.00000	0.00000	0.00000	0.00000	0.00000
P70245	EBP_MOUSE	0.00000	0.00000	0.00000	0.00000	0.00000	0.00000	0.02101	0.01093	0.01870	0.01521	0.01752	0.01508
P70261	PALD_MOUSE	0.00000	0.00000	0.00000	0.00000	0.00000	0.00000	0.01293	0.00490	0.00920	0.03002	0.00945	0.03794
P70268	PKN1_MOUSE	0.00000	0.00000	0.00000	0.00000	0.00000	0.00000	0.00664	0.00759	0.00716	0.00701	0.00759	0.00848
P70274	SEPP1_MOUSE	0.00000	0.00000	0.00000	0.00000	0.00000	0.00000	0.00544	0.00000	0.00323	0.00221	0.00565	0.00374
P70275	SEM3E_MOUSE	0.00000	0.00000	0.00000	0.00000	0.00000	0.00000	0.00000	0.00100	0.00000	0.00000	0.00284	0.00000
P70280	VAMP7_MOUSE	0.00000	0.00000	0.00000	0.00000	0.00000	0.00000	0.00000	0.00000	0.00228	0.00179	0.00000	0.00055
P70290	EM55_MOUSE	0.00000	0.00000	0.00000	0.00000	0.00000	0.00000	0.01038	0.00635	0.00641	0.00851	0.01206	0.00554
P70296	PEBP1_MOUSE	0.06994	0.04732	0.04006	0.07937	0.04303	0.05331	0.07303	0.07320	0.04426	0.06437	0.05615	0.07055
P70297	STAM1_MOUSE	0.00000	0.00000	0.00000	0.00000	0.00000	0.00000	0.01133	0.00949	0.01528	0.01846	0.01668	0.01806
P70302	STIM1_MOUSE	0.00000	0.00000	0.00000	0.00000	0.00000	0.00000	0.00576	0.00693	0.00891	0.00456	0.00661	0.00729
P70303	PYRG2_MOUSE	0.00000	0.00000	0.00000	0.00000	0.00000	0.00000	0.00363	0.00390	0.00502	0.00381	0.00451	0.00374
P70315	WASP_MOUSE	0.00000	0.00000	0.00000	0.00000	0.00000	0.00000	0.01733	0.00525	0.00000	0.00000	0.00254	0.00400
P70318	TIAR_MOUSE	0.00000	0.00000	0.00000	0.00000	0.00000	0.00000	0.00093	0.00269	0.00219	0.00050	0.00162	0.00130
P70333	HNRH2_MOUSE	0.00155	0.00148	0.00229	0.00234	0.00250	0.00271	0.01188	0.01814	0.00804	0.00669	0.00830	0.01427
P70335	ROCK1_MOUSE	0.00000	0.00000	0.00000	0.00000	0.00000	0.00000	0.00805	0.01499	0.01462	0.01443	0.00879	0.01581
P70336	ROCK2_MOUSE	0.00000	0.00000	0.00000	0.00000	0.00000	0.00000	0.02371	0.01687	0.02014	0.01891	0.01527	0.02050
P70349	HINT1_MOUSE	0.00000	0.00000	0.00000	0.00000	0.00000	0.00000	0.02580	0.03855	0.01840	0.02313	0.02701	0.02289
P70362	UFD1_MOUSE	0.00000	0.00000	0.00000	0.00000	0.00000	0.00000	0.00914	0.00314	0.00599	0.00918	0.00691	0.00671
P70372	ELAV1_MOUSE	0.00000	0.00000	0.00000	0.00000	0.00000	0.00000	0.01778	0.02207	0.02022	0.01884	0.01962	0.02097
P70387	HFE_MOUSE	0.00000	0.00000	0.00000	0.00000	0.00000	0.00000	0.00000	0.00061	0.00178	0.00000	0.00116	0.00000
P70398	USP9X_MOUSE	0.00389	0.00539	0.00574	0.00288	0.00231	0.00146	0.00780	0.00437	0.00909	0.00622	0.00861	0.00656

P70402	MYBPH_MOUSE	0.00000	0.00000	0.00000	0.00000	0.00000	0.00000	0.00289	0.00000	0.00000	0.00000	0.00000	0.00000
P70404	IDHG1_MOUSE	0.19891	0.09965	0.20284	0.20228	0.22053	0.11789	0.01671	0.01904	0.01098	0.01361	0.01178	0.01572
P70414	NAC1_MOUSE	0.00778	0.03474	0.01755	0.00863	0.01052	0.01200	0.00144	0.00000	0.00000	0.00114	0.00000	0.00050
P70419	GALT3_MOUSE	0.00000	0.00000	0.00000	0.00000	0.00000	0.00000	0.00251	0.00000	0.00461	0.00170	0.00164	0.00000
P70426	RIT1_MOUSE	0.00000	0.00000	0.00000	0.00000	0.00000	0.00000	0.00000	0.00050	0.00086	0.00059	0.00109	0.00031
P70429	EVL_MOUSE	0.00000	0.00000	0.00000	0.00000	0.00000	0.00000	0.00000	0.00000	0.00052	0.00000	0.00000	0.00000
P70441	NHRF1_MOUSE	0.00000	0.00000	0.00000	0.00000	0.00000	0.00000	0.00676	0.01569	0.01991	0.01817	0.01059	0.01148
P70445	4EBP2_MOUSE	0.00000	0.00000	0.00000	0.00000	0.00000	0.00000	0.00000	0.00000	0.00053	0.00026	0.00000	0.00000
P70452	STX4_MOUSE	0.00000	0.00000	0.00000	0.00000	0.00000	0.00000	0.00418	0.00404	0.00551	0.00360	0.00263	0.00668
P70460	VASP_MOUSE	0.00000	0.00000	0.00000	0.00000	0.00000	0.00000	0.00399	0.01041	0.01198	0.00920	0.01119	0.00994
P70663	SPRL1_MOUSE	0.00000	0.00000	0.00000	0.00000	0.00000	0.00000	0.00669	0.00263	0.00807	0.00353	0.00901	0.00586
P70670	NACAM_MOUSE	0.05058	0.08149	0.06417	0.07400	0.06104	0.05692	0.02082	0.04490	0.03393	0.04273	0.05015	0.03752
P70671	IRF3_MOUSE	0.00000	0.00000	0.00000	0.00000	0.00000	0.00000	0.00000	0.00074	0.00241	0.00116	0.00090	0.00078
P70677	CASP3_MOUSE	0.00000	0.00000	0.00000	0.00000	0.00000	0.00000	0.00126	0.00082	0.00447	0.00347	0.00245	0.00232
P70695	F16P2_MOUSE	0.00000	0.00000	0.00000	0.00000	0.00000	0.00000	0.00149	0.00000	0.00106	0.00193	0.00000	0.00187
P70698	PYRG1_MOUSE	0.00000	0.00000	0.00000	0.00000	0.00000	0.00000	0.00369	0.01089	0.01052	0.00802	0.00148	0.00429
P70699	LYAG_MOUSE	0.00000	0.00000	0.00000	0.00000	0.00000	0.00000	0.00123	0.00427	0.00525	0.00273	0.00278	0.00189
P70704	AT8A1_MOUSE	0.00000	0.00000	0.00000	0.00000	0.00000	0.00000	0.01067	0.01344	0.01699	0.01239	0.01221	0.01697
P80313	TCPH_MOUSE	0.01621	0.01252	0.01223	0.02304	0.01479	0.00937	0.04068	0.03205	0.04377	0.04033	0.04820	0.04404
P80314	TCPB_MOUSE	0.01078	0.00919	0.00772	0.01217	0.01140	0.01031	0.03233	0.03306	0.03912	0.03478	0.04414	0.03872
P80315	TCPD_MOUSE	0.01988	0.01018	0.01299	0.00704	0.00952	0.00717	0.02776	0.01674	0.02743	0.02792	0.02508	0.02845
P80316	TCPE_MOUSE	0.00195	0.00138	0.00313	0.00259	0.00336	0.00256	0.03321	0.02595	0.04901	0.03690	0.04739	0.04130
P80317	TCPZ_MOUSE	0.00307	0.00461	0.00529	0.00160	0.00349	0.00358	0.03098	0.02536	0.03344	0.03512	0.03634	0.03283
P80318	TCPG_MOUSE	0.00932	0.00667	0.00974	0.00716	0.00760	0.00601	0.02926	0.02465	0.03138	0.02670	0.03092	0.02840
P81117	NUCB2_MOUSE	0.00000	0.00000	0.00000	0.00000	0.00000	0.00000	0.01270	0.00765	0.01651	0.01439	0.01615	0.01270
P82198	BGH3_MOUSE	0.00000	0.00094	0.00071	0.00114	0.00103	0.00122	0.02007	0.00461	0.01223	0.00577	0.00988	0.01095
P82343	RENBP_MOUSE	0.00000	0.00000	0.00000	0.00000	0.00000	0.00000	0.00154	0.01345	0.00858	0.01061	0.01270	0.01156
P82347	SGCD_MOUSE	0.00982	0.01117	0.01206	0.01057	0.01287	0.00834	0.00765	0.00629	0.00238	0.00342	0.00115	0.00567
P82349	SGCB_MOUSE	0.00946	0.01327	0.00851	0.00917	0.01030	0.01177	0.00000	0.00000	0.00521	0.00200	0.00167	0.00421
P83510	TNIK_MOUSE	0.00000	0.00000	0.00000	0.00000	0.00000	0.00000	0.00000	0.00374	0.00485	0.00221	0.00093	0.00140
P83741	WNK1_MOUSE	0.00000	0.00000	0.00000	0.00000	0.00000	0.00000	0.00241	0.00662	0.00632	0.00673	0.00253	0.00330
P83887	TBG1_MOUSE	0.00000	0.00000	0.00000	0.00000	0.00000	0.00000	0.00135	0.00078	0.00205	0.00167	0.00318	0.00111
P83917	CBX1_MOUSE	0.00000	0.00000	0.00000	0.00000	0.00000	0.00000	0.00912	0.01392	0.01585	0.01167	0.01852	0.01689
P83940	ELOC_MOUSE	0.00000	0.00000	0.00000	0.00000	0.00000	0.00000	0.02690	0.02304	0.03372	0.03288	0.03253	0.02938

P84084	ARF5_MOUSE	0.01033	0.00881	0.00761	0.00497	0.00976	0.00785	0.00000	0.00887	0.00994	0.01215	0.00528	0.01086
P84089	ERH_MOUSE	0.00000	0.00000	0.00000	0.00000	0.00000	0.00000	0.01730	0.01407	0.02121	0.02326	0.02079	0.00979
P84091	AP2M1_MOUSE	0.00327	0.00586	0.00438	0.00632	0.00343	0.00749	0.01686	0.02492	0.02605	0.02147	0.01995	0.01820
P84096	RHOG_MOUSE	0.00000	0.00000	0.00000	0.00000	0.00000	0.00000	0.03507	0.03665	0.04092	0.03660	0.02651	0.03429
P84099	RL19_MOUSE	0.00000	0.00000	0.00000	0.00000	0.00000	0.00000	0.06163	0.06410	0.05854	0.06215	0.04715	0.06500
P84104	SRSF3_MOUSE	0.00000	0.00000	0.00000	0.00000	0.00000	0.00000	0.00788	0.00467	0.00069	0.00306	0.00252	0.00572
P85094	ISC2A_MOUSE	0.01166	0.01777	0.02752	0.01826	0.02938	0.01348	0.00000	0.00000	0.00000	0.00000	0.00000	0.00000
Q3TL54	TR43A_MOUSE	0.00000	0.00000	0.00000	0.00000	0.00000	0.00000	0.01707	0.00000	0.00072	0.00000	0.00000	0.00000
P97290	IC1_MOUSE	0.00084	0.00035	0.00087	0.00140	0.00090	0.00019	0.00933	0.00938	0.02041	0.01906	0.02653	0.01305
P97298	PEDF_MOUSE	0.00000	0.00000	0.00000	0.00000	0.00000	0.00000	0.00218	0.00394	0.00351	0.00432	0.00594	0.00444
P97300	NPTN_MOUSE	0.00000	0.00000	0.00000	0.00000	0.00000	0.00000	0.08448	0.00824	0.01374	0.01013	0.00716	0.00736
P97314	CSRP2_MOUSE	0.00000	0.00000	0.00000	0.00000	0.00000	0.00000	0.00128	0.00055	0.00485	0.00299	0.00159	0.00321
P97315	CSRP1_MOUSE	0.00000	0.00000	0.00000	0.00000	0.00000	0.00000	0.10564	0.14548	0.14485	0.10878	0.11087	0.12717
P97328	KHK_MOUSE	0.00000	0.00000	0.00000	0.00000	0.00000	0.00000	0.00000	0.00000	0.00000	0.00000	0.00000	0.00000
P97333	NRP1_MOUSE	0.00042	0.00256	0.00019	0.00014	0.00030	0.00064	0.11353	0.15228	0.14580	0.12394	0.12743	0.14606
P97347	RPTN_MOUSE	0.00000	0.00000	0.00000	0.00000	0.00000	0.00000	0.00705	0.00000	0.00049	0.00000	0.00000	0.00000
P97351	RS3A_MOUSE	0.02183	0.03108	0.01993	0.01933	0.01640	0.01754	0.05678	0.06507	0.09244	0.06880	0.06373	0.07386
P97352	S10AD_MOUSE	0.00000	0.00000	0.00000	0.00000	0.00000	0.00000	0.00876	0.00442	0.00962	0.00754	0.00747	0.00740
P97355	SPSY_MOUSE	0.00195	0.00831	0.00442	0.00338	0.00485	0.00450	0.00574	0.00777	0.00284	0.00306	0.00597	0.00533
P97358	TAF1B_MOUSE	0.00000	0.00000	0.00000	0.00000	0.00000	0.00000	0.02888	0.03973	0.00896	0.01749	0.03259	0.01365
P97363	SPTC2_MOUSE	0.00000	0.00000	0.00000	0.00000	0.00000	0.00000	0.01266	0.01067	0.01033	0.00910	0.00387	0.01527
P97364	SPS2_MOUSE	0.00000	0.00000	0.00000	0.00000	0.00000	0.00000	0.00020	0.00000	0.00000	0.00000	0.00000	0.00000
P97370	AT1B3_MOUSE	0.00309	0.00655	0.00430	0.00131	0.00427	0.00746	0.04593	0.04271	0.04834	0.03527	0.04676	0.04419
P97371	PSME1_MOUSE	0.00000	0.00000	0.00000	0.00000	0.00000	0.00000	0.04861	0.04906	0.06594	0.05828	0.06079	0.06136
P97372	PSME2_MOUSE	0.00000	0.00000	0.00000	0.00000	0.00000	0.00000	0.02125	0.02173	0.04328	0.03143	0.03990	0.03029
P97379	G3BP2_MOUSE	0.00000	0.00000	0.00000	0.00000	0.00000	0.00000	0.00565	0.00301	0.00729	0.00497	0.00668	0.00557
P97384	ANX11_MOUSE	0.01366	0.02692	0.00940	0.01488	0.01152	0.01256	0.11319	0.12496	0.10994	0.10689	0.10670	0.11931
P97390	VPS45_MOUSE	0.00000	0.00000	0.00000	0.00000	0.00000	0.00000	0.00582	0.00326	0.00599	0.00512	0.00469	0.00312
P97426	ECP1_MOUSE	0.00000	0.00000	0.00000	0.00000	0.00000	0.00000	0.00247	0.01574	0.00991	0.01802	0.03259	0.07594
P97429	ANXA4_MOUSE	0.01172	0.01492	0.01712	0.01012	0.01371	0.01696	0.06604	0.07407	0.10171	0.09460	0.08680	0.08151
P97434	MPRIP_MOUSE	0.00000	0.00000	0.00000	0.00000	0.00000	0.00000	0.02761	0.02882	0.03360	0.02855	0.02883	0.03271
P97443	SMYD1_MOUSE	0.06670	0.08141	0.06565	0.04923	0.05493	0.06992	0.36376	0.03635	0.01843	0.03906	0.00659	0.00619
P97447	FHL1_MOUSE	0.00000	0.00000	0.00000	0.00000	0.00000	0.00000	0.11490	0.23656	0.14440	0.16947	0.05000	0.20022
P97449	AMPN_MOUSE	0.00071	0.00168	0.00144	0.00129	0.00179	0.00155	0.00501	0.00694	0.01160	0.00559	0.00976	0.00763

P97450	ATP5J_MOUSE	0.21197	0.14007	0.12494	0.13042	0.13032	0.07486	0.01084	0.01545	0.01471	0.01296	0.01017	0.01446
P97457	MLRS_MOUSE	0.00000	0.00000	0.00000	0.00000	0.00000	0.00000	0.06783	0.00038	0.00026	0.00027	0.00014	0.00017
P97461	RS5_MOUSE	0.00000	0.00000	0.00000	0.00000	0.00000	0.00000	0.07522	0.05733	0.05783	0.06806	0.05348	0.06791
P97468	CML1_MOUSE	0.00000	0.00000	0.00000	0.00000	0.00000	0.00000	0.00000	0.00160	0.00646	0.00400	0.00396	0.00000
P97470	PP4C_MOUSE	0.00000	0.00000	0.00000	0.00000	0.00000	0.00000	0.00218	0.00789	0.00450	0.00447	0.00585	0.00602
P97471	SMAD4_MOUSE	0.00000	0.00000	0.00000	0.00000	0.00000	0.00000	0.00080	0.00000	0.00066	0.00000	0.00373	0.00178
P97478	COQ7_MOUSE	0.00442	0.00282	0.00181	0.00291	0.00189	0.00287	0.00000	0.00054	0.00000	0.00000	0.00000	0.00000
P97493	THIOM_MOUSE	0.00000	0.00000	0.00000	0.00000	0.00000	0.00000	0.00309	0.00422	0.00228	0.00249	0.00043	0.00340
P97494	GSH1_MOUSE	0.00000	0.00000	0.00000	0.00000	0.00000	0.00000	0.01956	0.01656	0.02520	0.02734	0.02540	0.02115
P97501	FMO3_MOUSE	0.00000	0.00000	0.00000	0.00000	0.00000	0.00000	0.01547	0.02286	0.00328	0.00867	0.01582	0.02085
P97742	CPT1A_MOUSE	0.00000	0.00000	0.00000	0.00000	0.00000	0.00000	0.02144	0.01641	0.02703	0.02414	0.02407	0.02457
P97760	RPB3_MOUSE	0.00000	0.00000	0.00000	0.00000	0.00000	0.00000	0.01233	0.01130	0.00842	0.01269	0.01410	0.01388
P97792	CXAR_MOUSE	0.00000	0.00000	0.00000	0.00000	0.00000	0.00000	0.01061	0.01494	0.01051	0.00863	0.01170	0.01471
P97797	SHPS1_MOUSE	0.00000	0.00000	0.00000	0.00000	0.00000	0.00000	0.00000	0.00036	0.00224	0.00000	0.00070	0.00070
P97807	FUMH_MOUSE	0.38001	0.22687	0.30806	0.34434	0.34046	0.32135	0.05689	0.06036	0.03762	0.04465	0.04421	0.04730
P97816	S100G_MOUSE	0.00000	0.00000	0.00000	0.00000	0.00000	0.00000	0.00131	0.00000	0.00000	0.00000	0.00000	0.00000
P97821	CATC_MOUSE	0.00000	0.00000	0.00000	0.00000	0.00000	0.00000	0.01024	0.00709	0.00835	0.00834	0.00649	0.00921
P97822	AN32E_MOUSE	0.00000	0.00000	0.00000	0.00000	0.00000	0.00000	0.02538	0.01147	0.01988	0.01769	0.02698	0.01764
P97823	LYPA1_MOUSE	0.00087	0.00072	0.00141	0.00039	0.00119	0.00073	0.00276	0.00925	0.00214	0.00415	0.00143	0.00813
P97855	G3BP1_MOUSE	0.00583	0.00355	0.00441	0.00424	0.00427	0.00480	0.01314	0.00755	0.01586	0.01161	0.01555	0.01504
P97858	S35B1_MOUSE	0.00000	0.00000	0.00000	0.00000	0.00000	0.00000	0.00000	0.00507	0.00381	0.00250	0.00256	0.00282
P97863	NFIB_MOUSE	0.00000	0.00000	0.00000	0.00000	0.00000	0.00000	0.00335	0.00382	0.00160	0.00174	0.00129	0.00399
P97864	CASP7_MOUSE	0.00000	0.00000	0.00000	0.00000	0.00000	0.00000	0.00000	0.00071	0.00141	0.00282	0.00146	0.00321
P97872	FMO5_MOUSE	0.00000	0.00000	0.00000	0.00000	0.00000	0.00000	0.03925	0.04067	0.03647	0.03082	0.04228	0.03682
P97873	LOXL1_MOUSE	0.00000	0.00000	0.00000	0.00000	0.00000	0.00000	0.05505	0.04464	0.02805	0.00836	0.04998	0.04502
P97927	LAMA4_MOUSE	0.00767	0.02150	0.00645	0.00793	0.00688	0.00794	0.04162	0.02972	0.03881	0.03168	0.04593	0.03733
P98078	DAB2_MOUSE	0.00000	0.00000	0.00000	0.00000	0.00000	0.00000	0.00802	0.00560	0.00437	0.00379	0.00449	0.00460
P98083	SHC1_MOUSE	0.00000	0.00000	0.00000	0.00000	0.00000	0.00000	0.00218	0.00357	0.00000	0.00000	0.00000	0.00000
P98192	GNPAT_MOUSE	0.00198	0.00237	0.00435	0.00400	0.00369	0.00355	0.00000	0.00000	0.00039	0.00086	0.00040	0.00078
P98197	AT11A_MOUSE	0.00000	0.00000	0.00000	0.00000	0.00000	0.00000	0.00013	0.00081	0.00024	0.00015	0.00114	0.00013
P99024	TBB5_MOUSE	0.02206	0.02203	0.01388	0.01522	0.00829	0.01723	0.17998	0.24189	0.19673	0.23636	0.20276	0.25305
P99026	PSB4_MOUSE	0.00000	0.00000	0.00000	0.00000	0.00000	0.00000	0.03071	0.03835	0.02441	0.03097	0.03151	0.03367
P99027	RLA2_MOUSE	0.00000	0.00000	0.00000	0.00000	0.00000	0.00000	0.07619	0.06713	0.07212	0.07247	0.07796	0.07681
P99028	QCR6_MOUSE	0.00000	0.00000	0.00000	0.00000	0.00000	0.00000	0.01256	0.01298	0.00000	0.01402	0.00000	0.00863

P99029	PRDX5_MOUSE	0.05485	0.08468	0.07498	0.11113	0.04223	0.13803	0.03536	0.03590	0.02794	0.03117	0.03027	0.03666
Q00262	STX2_MOUSE	0.00000	0.00000	0.00000	0.00000	0.00000	0.00000	0.00000	0.00324	0.00334	0.00182	0.00250	0.00080
Q00519	XDH_MOUSE	0.00272	0.00563	0.00358	0.00455	0.00370	0.00381	0.08879	0.08061	0.09095	0.07752	0.08313	0.09394
Q00560	IL6RB_MOUSE	0.00000	0.00000	0.00000	0.00000	0.00000	0.00000	0.00113	0.00191	0.00085	0.00119	0.00375	0.00129
Q00612	G6PD1_MOUSE	0.00059	0.00123	0.00089	0.00204	0.00072	0.00110	0.03520	0.02846	0.02926	0.02873	0.02544	0.03013
Q00623	APOA1_MOUSE	0.00000	0.00000	0.00000	0.00000	0.00000	0.00000	0.04545	0.03628	0.05868	0.09091	0.10111	0.02626
Q00724	RET4_MOUSE	0.00000	0.00000	0.00000	0.00000	0.00000	0.00000	0.00000	0.00000	0.00104	0.00000	0.00000	0.00038
Q00896	A1AT3_MOUSE	0.00000	0.00000	0.00000	0.00000	0.00000	0.00000	0.48536	0.30922	0.40139	0.39919	0.60874	0.26401
Q00897	A1AT4_MOUSE	0.00462	0.00324	0.00331	0.00413	0.00464	0.00531	0.04753	0.06786	0.08731	0.07238	0.06489	0.07694
Q00898	A1AT5_MOUSE	0.00229	0.00497	0.00186	0.00286	0.00167	0.00311	0.13756	0.15381	0.20509	0.03914	0.15085	0.03032
Q00899	TYY1_MOUSE	0.00000	0.00000	0.00000	0.00000	0.00000	0.00000	0.00386	0.00183	0.00000	0.00156	0.00291	0.00259
Q00915	RET1_MOUSE	0.00000	0.00000	0.00000	0.00000	0.00000	0.00000	0.02641	0.02225	0.02977	0.02741	0.02114	0.02693
Q00PI9	HNRL2_MOUSE	0.00506	0.00540	0.00421	0.00438	0.00411	0.00426	0.01437	0.01550	0.02210	0.01648	0.02169	0.02291
Q01147	CREB1_MOUSE	0.00000	0.00000	0.00000	0.00000	0.00000	0.00000	0.00309	0.00000	0.00000	0.00226	0.00000	0.00000
Q01149	CO1A2_MOUSE	0.00000	0.00000	0.00000	0.00000	0.00000	0.00000	0.08231	0.14262	0.08853	0.05062	0.08223	0.02876
Q01279	EGFR_MOUSE	0.00000	0.00000	0.00000	0.00000	0.00000	0.00000	0.00313	0.00324	0.00384	0.00161	0.00499	0.00127
Q01405	SC23A_MOUSE	0.00409	0.00265	0.00603	0.00206	0.00334	0.00669	0.01264	0.00941	0.01156	0.01002	0.01180	0.01177
Q01730	RSU1_MOUSE	0.00607	0.00451	0.00616	0.00188	0.00603	0.00418	0.11867	0.08363	0.06957	0.09641	0.09979	0.11318
Q01768	NDKB_MOUSE	0.02589	0.02414	0.02716	0.04151	0.03427	0.01051	0.09586	0.10401	0.11313	0.11179	0.08175	0.07155
Q01853	TERA_MOUSE	0.15036	0.12577	0.14433	0.09980	0.15491	0.12519	0.06795	0.08049	0.09052	0.09062	0.08660	0.09198
Q02013	AQP1_MOUSE	0.00000	0.00000	0.00000	0.00000	0.00000	0.00000	0.29441	0.29948	0.25204	0.31406	0.24387	0.31583
Q02053	UBA1_MOUSE	0.11904	0.11644	0.10113	0.09477	0.09431	0.07838	0.06232	0.05481	0.07374	0.06678	0.06994	0.06749
Q02248	CTNB1_MOUSE	0.00260	0.00301	0.00104	0.00245	0.00128	0.00173	0.02693	0.03648	0.04838	0.03514	0.04808	0.04405
Q02257	PLAK_MOUSE	0.00470	0.00853	0.00725	0.01043	0.01101	0.00980	0.00744	0.02038	0.01767	0.01971	0.01954	0.01893
Q02357	ANK1_MOUSE	0.00131	0.00106	0.00091	0.01042	0.00197	0.00137	0.01178	0.00085	0.00462	0.01401	0.02666	0.00089
Q02566	MYH6_MOUSE	7.58049	7.92201	7.09070	7.45486	7.29235	7.55283	0.38773	0.32159	0.37635	0.46589	0.38222	0.31019
Q02788	CO6A2_MOUSE	0.04524	0.05979	0.02329	0.04837	0.03030	0.02972	0.08781	0.07375	0.07252	0.04652	0.06795	0.06596
Q02819	NUCB1_MOUSE	0.00000	0.00000	0.00000	0.00000	0.00000	0.00000	0.02663	0.02510	0.03475	0.03339	0.04063	0.02929
Q02858	TIE2_MOUSE	0.00000	0.00000	0.00000	0.00000	0.00000	0.00000	0.00174	0.00000	0.00142	0.00040	0.00242	0.00356
Q02956	KPCZ_MOUSE	0.00000	0.00000	0.00000	0.00000	0.00000	0.00000	0.00896	0.00686	0.00881	0.00682	0.00841	0.00809
Q03249	GALT_MOUSE	0.00000	0.00000	0.00000	0.00000	0.00000	0.00000	0.00000	0.00223	0.00190	0.00184	0.00258	0.00312
Q03265	ATPA_MOUSE	2.72824	3.07081	2.10549	2.72402	2.43569	3.27114	0.26160	0.23646	0.17410	0.22481	0.19741	0.23994
Q03734	SPA3M_MOUSE	0.00025	0.00062	0.00019	0.00036	0.00031	0.00000	0.00293	0.00175	0.00606	0.00629	0.00780	0.00535
Q03958	PFD6_MOUSE	0.00000	0.00000	0.00000	0.00000	0.00000	0.00000	0.00151	0.00000	0.00000	0.00000	0.00000	0.00000

Q04207	TF65_MOUSE	0.00000	0.00000	0.00000	0.00000	0.00000	0.00000	0.00000	0.00472	0.00485	0.00424	0.00554	0.00674
Q04447	KCRB_MOUSE	0.05492	0.04763	0.07248	0.05683	0.05935	0.05273	0.05887	0.06655	0.08069	0.07742	0.07714	0.06955
Q04646	ATNG_MOUSE	0.00000	0.00000	0.00000	0.00000	0.00000	0.00000	0.00276	0.00000	0.00000	0.00000	0.00117	0.00385
Q04690	NF1_MOUSE	0.00000	0.00000	0.00000	0.00000	0.00000	0.00000	0.00641	0.01461	0.00820	0.00387	0.00388	0.03622
Q04736	YES_MOUSE	0.00000	0.00000	0.00000	0.00000	0.00000	0.00000	0.00313	0.00922	0.02005	0.01007	0.02713	0.01752
Q04750	TOP1_MOUSE	0.00000	0.00000	0.00000	0.00000	0.00000	0.00000	0.00095	0.00473	0.00541	0.00421	0.00420	0.00482
Q04857	CO6A1_MOUSE	0.13514	0.25153	0.18090	0.18048	0.12376	0.11213	0.22635	0.13170	0.13179	0.09783	0.13079	0.10458
Q05144	RAC2_MOUSE	0.00000	0.00000	0.00000	0.00000	0.00000	0.00000	0.00062	0.00169	0.00180	0.00196	0.00124	0.00670
Q05186	RCN1_MOUSE	0.00000	0.00000	0.00000	0.00000	0.00000	0.00000	0.00692	0.01398	0.02042	0.01972	0.02258	0.01937
Q05306	COAA1_MOUSE	0.00000	0.00000	0.00000	0.00000	0.00000	0.00000	0.03892	0.00000	0.00033	0.00000	0.00554	0.00000
Q05421	CP2E1_MOUSE	0.00000	0.00000	0.00000	0.00000	0.00000	0.00000	0.00000	0.00000	0.00043	0.00000	0.00000	0.00000
Q05512	MARK2_MOUSE	0.00000	0.00000	0.00000	0.00000	0.00000	0.00000	0.00000	0.00088	0.00221	0.00110	0.00184	0.00066
Q05793	PGBM_MOUSE	0.07157	0.08459	0.07386	0.07776	0.06097	0.03312	0.12970	0.12121	0.11947	0.08285	0.17787	0.14350
Q05816	FABP5_MOUSE	0.00779	0.00369	0.00485	0.00500	0.00580	0.00566	0.01394	0.01090	0.02267	0.01473	0.00713	0.00898
Q05920	PYC_MOUSE	0.03751	0.02528	0.03938	0.02710	0.03158	0.03000	0.04601	0.02936	0.03321	0.02325	0.02731	0.02620
Q05BC3	EMAL1_MOUSE	0.00000	0.00000	0.00000	0.00000	0.00000	0.00000	0.00218	0.01315	0.01108	0.00955	0.00524	0.01040
Q05D44	IF2P_MOUSE	0.00000	0.00000	0.00000	0.00000	0.00000	0.00000	0.00000	0.00095	0.00568	0.00429	0.00291	0.00313
Q06138	CAB39_MOUSE	0.00283	0.00073	0.00172	0.00206	0.00221	0.00178	0.02540	0.03488	0.02343	0.02502	0.02911	0.02774
Q06185	ATP5I_MOUSE	0.00000	0.00000	0.00000	0.00000	0.00000	0.00000	0.07811	0.06215	0.05325	0.06115	0.03330	0.05213
Q06318	UTER_MOUSE	0.00000	0.00000	0.00000	0.00000	0.00000	0.00000	0.08933	0.06458	0.03823	0.03369	0.05459	0.05066
Q06335	APLP2_MOUSE	0.00000	0.00000	0.00000	0.00000	0.00000	0.00000	0.00000	0.00000	0.00064	0.00000	0.00162	0.00151
Q06770	CBG_MOUSE	0.00143	0.00146	0.00059	0.00181	0.00161	0.00131	0.00206	0.00110	0.00578	0.01679	0.01368	0.00743
Q06890	CLUS_MOUSE	0.00774	0.00528	0.00571	0.00475	0.00583	0.00482	0.08051	0.07856	0.08303	0.07813	0.09780	0.08290
Q07076	ANXA7_MOUSE	0.02112	0.01738	0.01532	0.01599	0.02197	0.02069	0.03682	0.06766	0.05227	0.05034	0.05552	0.04714
Q07113	MPRI_MOUSE	0.00000	0.00000	0.00000	0.00000	0.00000	0.00000	0.00214	0.00359	0.00074	0.00220	0.00000	0.00154
Q07417	ACADS_MOUSE	0.02868	0.01865	0.05803	0.03535	0.03243	0.02368	0.01503	0.01497	0.01505	0.01211	0.00760	0.00815
Q07456	AMBP_MOUSE	0.00000	0.00000	0.00000	0.00000	0.00000	0.00000	0.00000	0.00764	0.00000	0.00000	0.00296	0.00000
Q07797	LG3BP_MOUSE	0.00000	0.00000	0.00000	0.00000	0.00000	0.00000	0.00466	0.00453	0.00935	0.00680	0.00964	0.00462
Q07813	BAX_MOUSE	0.00000	0.00000	0.00000	0.00000	0.00000	0.00000	0.00423	0.01232	0.01277	0.01174	0.01096	0.01241
Q08024	PEBB_MOUSE	0.00000	0.00000	0.00000	0.00000	0.00000	0.00000	0.00000	0.00427	0.00102	0.00184	0.00000	0.00000
Q08091	CNN1_MOUSE	0.00000	0.00000	0.00000	0.00000	0.00000	0.00000	0.10321	0.07512	0.06679	0.05651	0.09707	0.06700
Q08093	CNN2_MOUSE	0.00000	0.00000	0.00000	0.00000	0.00000	0.00000	0.02572	0.01178	0.03442	0.02313	0.03292	0.03702
Q08189	TGM3_MOUSE	0.00000	0.00000	0.00000	0.00000	0.00000	0.00000	0.08895	0.00009	0.00011	0.00008	0.00005	0.00005
Q08481	PECA1_MOUSE	0.00000	0.00000	0.00000	0.00000	0.00000	0.00000	0.02216	0.03224	0.04215	0.03165	0.04047	0.03812

Q08857	CD36_MOUSE	0.08533	0.28514	0.19435	0.12813	0.25367	0.17451	0.09783	0.14440	0.11338	0.12856	0.14043	0.16604
Q08879	FBLN1_MOUSE	0.00000	0.00000	0.00000	0.00000	0.00000	0.00000	0.00985	0.00548	0.00784	0.00603	0.00511	0.00600
Q08943	SSRP1_MOUSE	0.00000	0.00000	0.00000	0.00000	0.00000	0.00000	0.00000	0.00811	0.00290	0.01063	0.00319	0.00000
Q09014	NCF1_MOUSE	0.00000	0.00000	0.00000	0.00000	0.00000	0.00000	0.00759	0.00274	0.00545	0.00524	0.00934	0.00553
Q09324	GCNT1_MOUSE	0.00000	0.00000	0.00000	0.00000	0.00000	0.00000	0.00000	0.00000	0.00000	0.00000	0.00000	0.00000
Q09PK2	APRV1_MOUSE	0.00000	0.00000	0.00000	0.00000	0.00000	0.00000	0.02573	0.00000	0.00020	0.00000	0.00036	0.00000
Q0P557	MIEAP_MOUSE	0.00000	0.00000	0.00000	0.00000	0.00000	0.00000	0.00000	0.00277	0.00518	0.00392	0.00297	0.00219
Q0P678	ZCH18_MOUSE	0.00000	0.00000	0.00000	0.00000	0.00000	0.00000	0.00000	0.00247	0.00230	0.00412	0.00000	0.00608
Q0VGB7	PP4R2_MOUSE	0.00000	0.00000	0.00000	0.00000	0.00000	0.00000	0.00000	0.00106	0.00209	0.00309	0.00144	0.00000
Q0VGY8	TANC1_MOUSE	0.00000	0.00000	0.00000	0.00000	0.00000	0.00000	0.00034	0.00072	0.00231	0.00082	0.00247	0.00131
Q11011	PSA_MOUSE	0.00293	0.00344	0.00496	0.00335	0.00329	0.00356	0.01329	0.01937	0.01854	0.01758	0.02152	0.01821
Q11136	PEPD_MOUSE	0.00000	0.00000	0.00000	0.00000	0.00000	0.00000	0.01054	0.00751	0.01382	0.01182	0.01641	0.01228
Q14B46	RTKN2_MOUSE	0.00000	0.00000	0.00000	0.00000	0.00000	0.00000	0.00000	0.00000	0.00095	0.00000	0.00109	0.00000
Q14C51	PTCD3_MOUSE	0.00174	0.00176	0.00342	0.00099	0.00298	0.00210	0.00998	0.00177	0.00445	0.00852	0.00937	0.00355
Q1HFZ0	NSUN2_MOUSE	0.00000	0.00000	0.00000	0.00000	0.00000	0.00000	0.00558	0.00415	0.00180	0.00402	0.00000	0.00106
Q1XH17	TRI72_MOUSE	0.10729	0.06693	0.15648	0.09718	0.11505	0.07894	0.00892	0.00085	0.00239	0.00146	0.00164	0.00071
Q2KN98	CYTSA_MOUSE	0.00000	0.00000	0.00000	0.00000	0.00000	0.00000	0.00041	0.00322	0.00161	0.00072	0.00096	0.00167
Q2TPA8	HSDL2_MOUSE	0.05308	0.05772	0.03879	0.03483	0.05239	0.05947	0.02109	0.01030	0.01401	0.01343	0.01265	0.01279
Q2VPQ9	EAF6_MOUSE	0.00000	0.00000	0.00000	0.00000	0.00000	0.00000	0.00132	0.00204	0.00153	0.00357	0.00217	0.00000
Q31125	S39A7_MOUSE	0.00000	0.00000	0.00000	0.00000	0.00000	0.00000	0.00000	0.00299	0.00829	0.00594	0.00352	0.00259
Q3B7Z2	OSBP1_MOUSE	0.00390	0.00325	0.00505	0.00339	0.00586	0.00474	0.00432	0.00630	0.00979	0.00424	0.00632	0.00548
Q3SXD3	HDDC2_MOUSE	0.00000	0.00000	0.00000	0.00000	0.00000	0.00000	0.00127	0.00000	0.00307	0.00218	0.00475	0.00270
Q3T9X0	GTR9_MOUSE	0.00000	0.00000	0.00000	0.00000	0.00000	0.00000	0.00000	0.00000	0.00000	0.00000	0.00000	0.00000
Q3TAS6	EMC10_MOUSE	0.00000	0.00000	0.00000	0.00000	0.00000	0.00000	0.00525	0.00608	0.00414	0.00503	0.00429	0.00557
Q3TBD2	HMHA1_MOUSE	0.00000	0.00000	0.00000	0.00000	0.00000	0.00000	0.00000	0.00118	0.00366	0.00242	0.00244	0.00371
Q3TBT3	STING_MOUSE	0.00000	0.00000	0.00000	0.00000	0.00000	0.00000	0.00422	0.00363	0.00000	0.00232	0.00272	0.00289
Q3TC72	FAHD2_MOUSE	0.02430	0.01959	0.03422	0.02050	0.02121	0.02325	0.00138	0.00466	0.00427	0.00122	0.00011	0.00219
Q3TC93	H1BP3_MOUSE	0.00000	0.00000	0.00000	0.00000	0.00000	0.00000	0.00124	0.00401	0.00282	0.00367	0.00603	0.00361
Q3TCJ1	ABRX2_MOUSE	0.00000	0.00000	0.00000	0.00000	0.00000	0.00000	0.00033	0.00170	0.00552	0.00357	0.00235	0.00174
Q3TCN2	PLBL2_MOUSE	0.00000	0.00000	0.00000	0.00000	0.00000	0.00000	0.00251	0.00242	0.00797	0.00207	0.00521	0.00492
Q3TDN2	FAF2_MOUSE	0.00000	0.00000	0.00000	0.00000	0.00000	0.00000	0.00415	0.00936	0.01049	0.01151	0.00799	0.00388
Q3TDQ1	STT3B_MOUSE	0.00000	0.00000	0.00000	0.00000	0.00000	0.00000	0.02300	0.01512	0.02211	0.01863	0.01908	0.01583
Q3TEA8	HP1B3_MOUSE	0.00134	0.00541	0.00395	0.00439	0.00365	0.00405	0.02960	0.03044	0.03728	0.03090	0.03809	0.03703
Q3TFD2	PCAT1_MOUSE	0.00000	0.00000	0.00000	0.00000	0.00000	0.00000	0.00550	0.00586	0.00990	0.00774	0.00648	0.00783

Q3TGF2	F107B_MOUSE	0.00000	0.00000	0.00000	0.00000	0.00000	0.00000	0.00000	0.00086	0.00225	0.00265	0.00321	0.00252
Q3THE2	ML12B_MOUSE	0.00000	0.00000	0.00000	0.00000	0.00000	0.00000	0.15319	0.20056	0.13838	0.17020	0.18358	0.19174
Q3THF9	CQ10B_MOUSE	0.00000	0.00000	0.00000	0.00000	0.00000	0.00000	0.00222	0.00064	0.00053	0.00089	0.00000	0.00000
Q3THG9	AASD1_MOUSE	0.00000	0.00000	0.00000	0.00000	0.00000	0.00000	0.00000	0.00000	0.00592	0.00444	0.00896	0.00337
Q3THK3	T2FA_MOUSE	0.00000	0.00000	0.00000	0.00000	0.00000	0.00000	0.00209	0.00123	0.00037	0.00120	0.00000	0.00196
Q3THK7	GUAA_MOUSE	0.00345	0.00263	0.00215	0.00210	0.00245	0.00301	0.00267	0.00910	0.00571	0.00474	0.00488	0.00535
Q3THS6	METK2_MOUSE	0.00000	0.00000	0.00000	0.00000	0.00000	0.00000	0.01262	0.01187	0.01144	0.01335	0.01425	0.01308
Q3TIR3	RIC8A_MOUSE	0.00000	0.00000	0.00000	0.00000	0.00000	0.00000	0.00000	0.00482	0.00533	0.00602	0.00307	0.00321
Q3TIX9	SNUT2_MOUSE	0.00000	0.00000	0.00000	0.00000	0.00000	0.00000	0.00000	0.00119	0.00000	0.00230	0.00100	0.00131
Q3TJ91	L2GL2_MOUSE	0.00000	0.00000	0.00000	0.00000	0.00000	0.00000	0.00422	0.00993	0.00511	0.00377	0.00454	0.00880
Q3TJD7	PDLI7_MOUSE	0.00000	0.00000	0.00000	0.00000	0.00000	0.00000	0.01854	0.00947	0.00674	0.00554	0.00973	0.00869
Q3TJZ6	FA98A_MOUSE	0.00000	0.00000	0.00000	0.00000	0.00000	0.00000	0.00602	0.00492	0.00187	0.00420	0.00321	0.00402
Q3TL44	NLRX1_MOUSE	0.00292	0.00237	0.00216	0.00199	0.00368	0.00264	0.00362	0.00060	0.00823	0.00563	0.00837	0.00465
Q3TLH4	PRC2C_MOUSE	0.00000	0.00000	0.00000	0.00000	0.00000	0.00000	0.00148	0.00000	0.00327	0.00211	0.00142	0.00338
Q3TLP5	ECHD2_MOUSE	0.00255	0.00312	0.00306	0.00271	0.00214	0.00369	0.00787	0.00435	0.00083	0.00000	0.00625	0.00527
Q3TMH2	SCRN3_MOUSE	0.00000	0.00000	0.00000	0.00000	0.00000	0.00000	0.00855	0.00546	0.00564	0.00668	0.01024	0.00443
Q3TMP8	TM38A_MOUSE	0.00000	0.00000	0.00000	0.00000	0.00000	0.00000	0.00326	0.00000	0.00000	0.00000	0.00000	0.00000
Q3TNA1	XYLB_MOUSE	0.00000	0.00000	0.00000	0.00000	0.00000	0.00000	0.00009	0.00000	0.00003	0.00000	0.00000	0.00000
Q3TPX4	EXOC5_MOUSE	0.00000	0.00000	0.00000	0.00000	0.00000	0.00000	0.00588	0.00437	0.00199	0.00418	0.00426	0.00621
Q3TUH1	TAM41_MOUSE	0.00000	0.00000	0.00000	0.00000	0.00000	0.00000	0.00000	0.00000	0.00000	0.00000	0.00000	0.00000
Q3TW96	UAP1L_MOUSE	0.00000	0.00000	0.00000	0.00000	0.00000	0.00000	0.01797	0.02025	0.01893	0.01781	0.01709	0.01954
Q3TWL2	PP4P1_MOUSE	0.00000	0.00000	0.00000	0.00000	0.00000	0.00000	0.00073	0.00463	0.00242	0.00103	0.00000	0.00265
Q3TWW8	SRSF6_MOUSE	0.00000	0.00000	0.00000	0.00000	0.00000	0.00000	0.00000	0.00491	0.00759	0.00457	0.00293	0.00299
Q3TXS7	PSMD1_MOUSE	0.01914	0.03884	0.01821	0.01711	0.02028	0.01316	0.01171	0.01396	0.01749	0.01482	0.02077	0.01415
Q3TYS2	CYBC1_MOUSE	0.00000	0.00000	0.00000	0.00000	0.00000	0.00000	0.00162	0.00338	0.00372	0.00223	0.00143	0.00316
Q3TZZ7	ESYT2_MOUSE	0.00062	0.00071	0.00086	0.00104	0.00117	0.00117	0.01015	0.02220	0.02207	0.01754	0.02260	0.02405
Q3U0S6	RAIN_MOUSE	0.00000	0.00000	0.00000	0.00000	0.00000	0.00000	0.00458	0.01050	0.01236	0.00960	0.01013	0.01195
Q3U0V1	FUBP2_MOUSE	0.00000	0.00000	0.00000	0.00000	0.00000	0.00000	0.01902	0.01652	0.01933	0.01647	0.01660	0.01896
Q3U0V2	TRADD_MOUSE	0.00000	0.00000	0.00000	0.00000	0.00000	0.00000	0.00590	0.00560	0.00841	0.00697	0.00433	0.00783
Q3U186	SYRM_MOUSE	0.00000	0.00000	0.00000	0.00000	0.00000	0.00000	0.00000	0.00000	0.00000	0.00000	0.00000	0.00000
Q3U1F9	PHAG1_MOUSE	0.00000	0.00000	0.00000	0.00000	0.00000	0.00000	0.00408	0.00592	0.00646	0.00652	0.00752	0.00861
Q3U1J4	DDB1_MOUSE	0.00982	0.01219	0.00981	0.00801	0.00941	0.00674	0.01287	0.01550	0.01679	0.01542	0.01961	0.01517
Q3U3V8	XRRA1_MOUSE	0.00000	0.00000	0.00000	0.00000	0.00000	0.00000	0.00273	0.02715	0.00421	0.00000	0.00052	0.01525
Q3U4I7	PYRD2_MOUSE	0.00000	0.00000	0.00000	0.00000	0.00000	0.00000	0.00000	0.00405	0.00203	0.00000	0.00000	0.00171

Q3U5Q7	CMPK2_MOUSE	0.00000	0.00000	0.00000	0.00000	0.00000	0.00000	0.00117	0.00623	0.00460	0.00357	0.00392	0.00678
Q3U7R1	ESYT1_MOUSE	0.00894	0.00818	0.00460	0.00645	0.00666	0.00743	0.03090	0.02351	0.04280	0.03526	0.04008	0.03540
Q3U7U3	FBX7_MOUSE	0.00000	0.00000	0.00000	0.00000	0.00000	0.00000	0.00000	0.00084	0.00309	0.00337	0.00593	0.00127
Q3U962	CO5A2_MOUSE	0.00000	0.00000	0.00000	0.00000	0.00000	0.00000	0.01082	0.00031	0.00526	0.00280	0.00909	0.00573
Q3U9G9	LBR_MOUSE	0.00000	0.00000	0.00000	0.00000	0.00000	0.00000	0.01738	0.01597	0.01950	0.01624	0.02429	0.02010
Q3UA37	QRIC1_MOUSE	0.00000	0.00000	0.00000	0.00000	0.00000	0.00000	0.00237	0.00125	0.00270	0.00261	0.00333	0.00322
Q3UBX0	TM109_MOUSE	0.00000	0.00000	0.00000	0.00000	0.00000	0.00000	0.00673	0.01928	0.00827	0.00727	0.00313	0.01267
Q3UDE2	TTL12_MOUSE	0.00000	0.00000	0.00000	0.00000	0.00000	0.00000	0.00277	0.00395	0.00929	0.00450	0.00659	0.00354
Q3UE37	UBE2Z_MOUSE	0.00000	0.00000	0.00000	0.00000	0.00000	0.00000	0.00000	0.00253	0.00047	0.00140	0.00145	0.00105
Q3UEB3	PUF60_MOUSE	0.00107	0.00077	0.00030	0.00149	0.00103	0.00102	0.01456	0.01173	0.02267	0.02084	0.02234	0.01823
Q3UEG6	AGT2_MOUSE	0.00000	0.00000	0.00000	0.00000	0.00000	0.00000	0.00011	0.00029	0.00038	0.00033	0.00031	0.00018
Q3UFF7	LYPL1_MOUSE	0.00000	0.00000	0.00000	0.00000	0.00000	0.00000	0.00181	0.00030	0.00073	0.00069	0.00102	0.00000
Q3UFK8	FRMD8_MOUSE	0.00000	0.00000	0.00000	0.00000	0.00000	0.00000	0.00313	0.00723	0.00543	0.00266	0.01430	0.00609
Q9DA80	RSH3B_MOUSE	0.00000	0.00000	0.00000	0.00000	0.00000	0.00000	0.00000	0.00000	0.00000	0.00000	0.00000	0.00000
Q3UGC7	EI3JA_MOUSE	0.00000	0.00000	0.00000	0.00000	0.00000	0.00000	0.00696	0.01511	0.01099	0.01295	0.01362	0.01546
Q3UGR5	HDHD2_MOUSE	0.00083	0.00015	0.00150	0.00116	0.00082	0.00048	0.00698	0.00744	0.00972	0.00838	0.00863	0.00675
Q3UH60	DIP2B_MOUSE	0.00000	0.00000	0.00000	0.00000	0.00000	0.00000	0.00592	0.00192	0.00000	0.00083	0.00095	0.00000
Q3UH68	LIMC1_MOUSE	0.00843	0.00671	0.00863	0.00492	0.00682	0.00605	0.03927	0.04084	0.05116	0.05344	0.04882	0.04628
Q3UHD6	SNX27_MOUSE	0.00164	0.00178	0.00151	0.00085	0.00128	0.00821	0.00000	0.00217	0.00198	0.00353	0.00326	0.00649
Q3UHJ0	AAK1_MOUSE	0.00000	0.00000	0.00000	0.00000	0.00000	0.00000	0.00025	0.00200	0.00246	0.00285	0.00174	0.00321
Q3UHX2	HAP28_MOUSE	0.00000	0.00000	0.00000	0.00000	0.00000	0.00000	0.01118	0.01130	0.00710	0.01128	0.00927	0.01213
Q3UIA2	RHG17_MOUSE	0.00000	0.00000	0.00000	0.00000	0.00000	0.00000	0.00277	0.00569	0.00752	0.00377	0.00469	0.00504
Q3UIJ9	MYZAP_MOUSE	0.00132	0.00161	0.00118	0.00112	0.00077	0.00142	0.01172	0.01671	0.01120	0.01481	0.00834	0.01327
Q3UIL6	PKHA7_MOUSE	0.00000	0.00000	0.00000	0.00000	0.00000	0.00000	0.00031	0.00045	0.00059	0.00026	0.00027	0.00000
Q3UIR3	DTX3L_MOUSE	0.00000	0.00000	0.00000	0.00000	0.00000	0.00000	0.00000	0.00275	0.00344	0.00364	0.00170	0.00166
Q3UIU2	NDUB6_MOUSE	0.00000	0.00000	0.00000	0.00000	0.00000	0.00000	0.02138	0.01507	0.01404	0.01478	0.01411	0.01632
Q3UJB9	EDC4_MOUSE	0.00000	0.00000	0.00000	0.00000	0.00000	0.00000	0.00032	0.00000	0.00199	0.00070	0.00161	0.00064
Q3UJP5	CH037_MOUSE	0.00000	0.00000	0.00000	0.00000	0.00000	0.00000	0.00000	0.00000	0.00112	0.00013	0.00014	0.00000
Q3UJU9	RMD3_MOUSE	0.00000	0.00000	0.00000	0.00000	0.00000	0.00000	0.00311	0.00400	0.00340	0.00335	0.00000	0.00508
Q3UKJ7	SMU1_MOUSE	0.00000	0.00000	0.00000	0.00000	0.00000	0.00000	0.00703	0.00456	0.00691	0.00435	0.00461	0.00397
Q3ULD5	MCCB_MOUSE	0.03361	0.02803	0.04064	0.03130	0.03305	0.02811	0.01541	0.00903	0.01336	0.01020	0.01051	0.00927
Q3ULJ0	GPD1L_MOUSE	0.00000	0.00000	0.00000	0.00000	0.00000	0.00000	0.00000	0.01221	0.01413	0.01165	0.00251	0.00680
Q3ULW8	PARP3_MOUSE	0.00000	0.00000	0.00000	0.00000	0.00000	0.00000	0.00565	0.00346	0.00301	0.00353	0.00165	0.00493
Q3UM45	PP1R7_MOUSE	0.00000	0.00000	0.00000	0.00000	0.00000	0.00000	0.02703	0.02047	0.02895	0.02488	0.03235	0.02762

Q9D3R6	KATL2_MOUSE	0.00000	0.00000	0.00000	0.00000	0.00000	0.00000	0.00693	0.01969	0.00336	0.00925	0.00000	0.00682
Q3UMF0	COBL1_MOUSE	0.00000	0.00000	0.00000	0.00000	0.00000	0.00000	0.00000	0.00122	0.00330	0.00181	0.00092	0.00066
Q3UMR5	MCU_MOUSE	0.00000	0.00000	0.00000	0.00000	0.00000	0.00000	0.00522	0.00306	0.00633	0.00391	0.00728	0.00467
Q3UMT1	PP12C_MOUSE	0.00000	0.00000	0.00000	0.00000	0.00000	0.00000	0.00000	0.00279	0.00514	0.00302	0.00261	0.00092
Q3UMU9	HDGR2_MOUSE	0.00000	0.00000	0.00000	0.00000	0.00000	0.00000	0.00510	0.00276	0.00881	0.00465	0.00900	0.00497
Q3UMY5	EMAL4_MOUSE	0.00000	0.00000	0.00000	0.00000	0.00000	0.00000	0.01380	0.01092	0.01572	0.01395	0.01548	0.01479
Q3UNX5	ACSM3_MOUSE	0.00000	0.00000	0.00000	0.00000	0.00000	0.00000	0.00000	0.00001	0.00001	0.00000	0.00000	0.00002
Q3UNZ8	QORL2_MOUSE	0.00000	0.00000	0.00000	0.00000	0.00000	0.00000	0.00067	0.00056	0.00063	0.00020	0.00063	0.00079
Q3UP75	UD3A1_MOUSE	0.00000	0.00000	0.00000	0.00000	0.00000	0.00000	0.00451	0.00723	0.00233	0.00350	0.00275	0.00520
Q3UP87	ELNE_MOUSE	0.00000	0.00000	0.00000	0.00000	0.00000	0.00000	0.00000	0.00799	0.00306	0.00543	0.00519	0.00464
Q3UPF5	ZCCHV_MOUSE	0.00000	0.00000	0.00000	0.00000	0.00000	0.00000	0.00336	0.00179	0.00261	0.00454	0.00102	0.00528
Q3UPH1	PRRC1_MOUSE	0.00000	0.00000	0.00000	0.00000	0.00000	0.00000	0.00551	0.00513	0.00766	0.00502	0.00660	0.00681
Q3UPL0	SC31A_MOUSE	0.00000	0.00000	0.00000	0.00000	0.00000	0.00000	0.01847	0.01535	0.01918	0.01609	0.01909	0.01682
Q3UPY5	GLBL2_MOUSE	0.00000	0.00000	0.00000	0.00000	0.00000	0.00000	0.00000	0.00000	0.00000	0.00000	0.00000	0.00000
Q3UQ28	PXDN_MOUSE	0.00000	0.00000	0.00000	0.00000	0.00000	0.00000	0.00353	0.00245	0.00381	0.00140	0.00171	0.00265
Q3UQ44	IQGA2_MOUSE	0.00000	0.00000	0.00000	0.00000	0.00000	0.00000	0.00326	0.00515	0.00268	0.00323	0.00000	0.00143
Q3UQ84	SYTM_MOUSE	0.00345	0.00236	0.00265	0.00347	0.00215	0.00251	0.00000	0.00000	0.00000	0.00000	0.00000	0.00000
Q3UQN2	FCHO2_MOUSE	0.00000	0.00000	0.00000	0.00000	0.00000	0.00000	0.00242	0.00303	0.00591	0.00370	0.00533	0.00473
Q3URD3	SLMAP_MOUSE	0.01443	0.01500	0.01721	0.01348	0.01728	0.01612	0.01435	0.00923	0.01180	0.01090	0.01249	0.01165
Q3URE1	ACSF3_MOUSE	0.00873	0.02254	0.00843	0.00621	0.00562	0.00331	0.00120	0.00000	0.00138	0.00000	0.00180	0.00000
Q3USB7	PLCL1_MOUSE	0.00000	0.00000	0.00000	0.00000	0.00000	0.00000	0.00000	0.00103	0.00000	0.00373	0.00000	0.00000
Q3UUI3	THEM4_MOUSE	0.00000	0.00000	0.00000	0.00000	0.00000	0.00000	0.00000	0.00029	0.00012	0.00052	0.00123	0.00000
Q3UV17	K22O_MOUSE	0.00000	0.00000	0.00000	0.00000	0.00000	0.00000	0.00000	0.00603	0.00661	0.00494	0.00435	0.00376
Q3UV70	PDP1_MOUSE	0.00470	0.00324	0.00365	0.00281	0.00303	0.00145	0.00000	0.00000	0.00237	0.00168	0.00000	0.00090
Q3UVK0	ERMP1_MOUSE	0.00000	0.00000	0.00000	0.00000	0.00000	0.00000	0.00000	0.00056	0.00051	0.00054	0.00030	0.00031
Q3UVL4	VPS51_MOUSE	0.00000	0.00000	0.00000	0.00000	0.00000	0.00000	0.01961	0.01118	0.00831	0.02988	0.00893	0.00595
Q3UW53	NIBA1_MOUSE	0.00000	0.00000	0.00000	0.00000	0.00000	0.00000	0.01221	0.01730	0.01758	0.01519	0.01656	0.01946
Q3UX10	TBAL3_MOUSE	0.00000	0.00000	0.00000	0.00000	0.00000	0.00000	0.02132	0.01015	0.01887	0.01618	0.01586	0.02037
Q3UYC0	PPM1H_MOUSE	0.00000	0.00000	0.00000	0.00000	0.00000	0.00000	0.00000	0.00000	0.00000	0.00000	0.00000	0.00000
Q3UYV9	NCBP1_MOUSE	0.00000	0.00000	0.00000	0.00000	0.00000	0.00000	0.00139	0.00248	0.00263	0.00345	0.00394	0.00418
Q3UZ39	LRRF1_MOUSE	0.00000	0.00000	0.00000	0.00000	0.00000	0.00000	0.00160	0.00889	0.01880	0.01416	0.00883	0.00839
Q3UZA1	CPZIP_MOUSE	0.00000	0.00000	0.00000	0.00000	0.00000	0.00000	0.00257	0.00478	0.00704	0.00643	0.00322	0.00633
Q3UZZ6	ST1D1_MOUSE	0.00000	0.00000	0.00000	0.00000	0.00000	0.00000	0.00177	0.00318	0.00068	0.00159	0.00105	0.00184
Q3V0K9	PLSI_MOUSE	0.00000	0.00000	0.00000	0.00000	0.00000	0.00000	0.00357	0.00239	0.00292	0.00458	0.00312	0.00296

Q3V1L4	5NTC_MOUSE	0.00040	0.00000	0.00129	0.00055	0.00113	0.00062	0.00040	0.00000	0.00116	0.00000	0.00078	0.00000
Q3V384	AFG1L_MOUSE	0.00940	0.06520	0.01016	0.03811	0.00799	0.00917	0.00000	0.00191	0.00000	0.00000	0.00000	0.00000
Q3V3R4	ITA1_MOUSE	0.00036	0.00028	0.00081	0.00293	0.00082	0.00057	0.02281	0.03081	0.03326	0.03046	0.03018	0.02919
Q49B93	SC5AC_MOUSE	0.00000	0.00000	0.00000	0.00000	0.00000	0.00000	0.00000	0.00000	0.00000	0.00000	0.00000	0.00000
Q4ACU6	SHAN3_MOUSE	0.00000	0.00000	0.00000	0.00000	0.00000	0.00000	0.01015	0.00565	0.01081	0.00701	0.01255	0.00927
Q4FZC9	SYNE3_MOUSE	0.00000	0.00000	0.00000	0.00000	0.00000	0.00000	0.03312	0.00000	0.03140	0.04167	0.07024	0.00808
Q9WVA2	TIM8A_MOUSE	0.00000	0.00000	0.00000	0.00000	0.00000	0.00000	0.00264	0.00287	0.00084	0.00442	0.00115	0.00146
Q4KML4	ABRAL_MOUSE	0.00000	0.00000	0.00000	0.00000	0.00000	0.00000	0.03112	0.02101	0.02204	0.02521	0.03160	0.02330
Q4LDD4	ARAP1_MOUSE	0.00000	0.00000	0.00000	0.00000	0.00000	0.00000	0.02080	0.00572	0.01611	0.01247	0.01704	0.01808
Q4PZA2	ECE1_MOUSE	0.00000	0.00000	0.00000	0.00000	0.00000	0.00000	0.00472	0.01197	0.01485	0.01087	0.01482	0.01397
Q4VA53	PDS5B_MOUSE	0.00000	0.00000	0.00000	0.00000	0.00000	0.00000	0.00150	0.00405	0.00777	0.00527	0.00531	0.00475
Q4VAA2	CDV3_MOUSE	0.00000	0.00000	0.00000	0.00000	0.00000	0.00000	0.00000	0.00080	0.00181	0.00259	0.00142	0.00110
Q4VBD2	TAPT1_MOUSE	0.00000	0.00000	0.00000	0.00000	0.00000	0.00000	0.00000	0.00179	0.00000	0.00185	0.00000	0.00000
Q501J6	DDX17_MOUSE	0.00000	0.00000	0.00000	0.00000	0.00000	0.00000	0.08837	0.11853	0.08101	0.09410	0.09750	0.12709
Q501J7	PHAR4_MOUSE	0.00000	0.00000	0.00000	0.00000	0.00000	0.00000	0.00000	0.00000	0.00301	0.00139	0.00138	0.00106
Q505B7	ARCH_MOUSE	0.00000	0.00000	0.00000	0.00000	0.00000	0.00000	0.00254	0.00549	0.00338	0.00207	0.00256	0.00414
Q505F5	LRC47_MOUSE	0.00000	0.00000	0.00000	0.00000	0.00000	0.00000	0.00685	0.00286	0.00535	0.00453	0.00348	0.00518
Q52KI8	SRRM1_MOUSE	0.00000	0.00000	0.00000	0.00000	0.00000	0.00000	0.00587	0.00662	0.00374	0.00688	0.00673	0.00833
Q569Z5	DDX46_MOUSE	0.00000	0.00000	0.00000	0.00000	0.00000	0.00000	0.00407	0.01092	0.00620	0.01340	0.00000	0.00000
Q569Z6	TR150_MOUSE	0.00000	0.00000	0.00000	0.00000	0.00000	0.00000	0.00000	0.00465	0.01245	0.00976	0.00447	0.00435
Q570Y9	DPTOR_MOUSE	0.00000	0.00000	0.00000	0.00000	0.00000	0.00000	0.00370	0.00298	0.00466	0.00229	0.00404	0.00278
Q571E4	GALNS_MOUSE	0.00000	0.00000	0.00000	0.00000	0.00000	0.00000	0.00378	0.00000	0.00239	0.00000	0.00000	0.00000
Q571I9	A16A1_MOUSE	0.00000	0.00000	0.00000	0.00000	0.00000	0.00000	0.00000	0.00160	0.00154	0.00301	0.00102	0.00123
Q58A65	JIP4_MOUSE	0.00131	0.00093	0.00053	0.00113	0.00126	0.00224	0.00730	0.00943	0.01267	0.00713	0.00904	0.01070
Q59J78	NDUF2_MOUSE	0.00389	0.00760	0.00914	0.00859	0.00774	0.01314	0.00257	0.00298	0.00286	0.00337	0.00000	0.00333
Q5DTX6	JCAD_MOUSE	0.00000	0.00000	0.00000	0.00000	0.00000	0.00000	0.00431	0.00536	0.00751	0.00664	0.00607	0.00679
Q5EBG6	HSPB6_MOUSE	0.00054	0.00270	0.00359	0.00471	0.00164	0.00681	0.00305	0.00027	0.00056	0.00042	0.00000	0.00029
Q5EG47	AAPK1_MOUSE	0.00000	0.00000	0.00000	0.00000	0.00000	0.00000	0.00467	0.01202	0.00687	0.00896	0.00999	0.00601
Q5F201	CFA52_MOUSE	0.00000	0.00000	0.00000	0.00000	0.00000	0.00000	0.00241	0.00100	0.00186	0.00333	0.00242	0.00596
Q5FW53	MBPHL_MOUSE	0.00000	0.00000	0.00000	0.00000	0.00000	0.00000	0.00000	0.00640	0.00841	0.01070	0.01365	0.01093
Q5FW60	MUP20_MOUSE	0.00000	0.00000	0.00000	0.00000	0.00000	0.00000	0.00000	0.00000	0.00000	0.00000	0.00042	0.00000
Q5FWI3	CEIP2_MOUSE	0.00000	0.00000	0.00000	0.00000	0.00000	0.00000	0.01848	0.02827	0.03571	0.02912	0.02900	0.02827
Q5FWK3	RHG01_MOUSE	0.00000	0.00000	0.00000	0.00000	0.00000	0.00000	0.04844	0.07659	0.08041	0.07354	0.08228	0.07672
Q5M8N0	CNRP1_MOUSE	0.00000	0.00000	0.00000	0.00000	0.00000	0.00000	0.04244	0.00821	0.01676	0.02043	0.00644	0.01758

Q5M8N4	D39U1_MOUSE	0.00853	0.00563	0.00793	0.00481	0.00480	0.01085	0.00000	0.00046	0.00095	0.00000	0.00000	0.00114
Q5NCF2	TPPC1_MOUSE	0.00000	0.00000	0.00000	0.00000	0.00000	0.00000	0.00000	0.00083	0.00216	0.00161	0.00104	0.00119
Q5RL79	KTAP2_MOUSE	0.00000	0.00000	0.00000	0.00000	0.00000	0.00000	0.00270	0.00593	0.00612	0.00651	0.00561	0.00618
Q5SRX1	TM1L2_MOUSE	0.00050	0.00085	0.00073	0.00046	0.00075	0.00077	0.00488	0.00519	0.00833	0.00635	0.01423	0.00775
Q5SS90	CG057_MOUSE	0.00000	0.00000	0.00000	0.00000	0.00000	0.00000	0.00198	0.00000	0.00000	0.00000	0.00000	0.00000
Q5SSZ5	TENS3_MOUSE	0.00000	0.00000	0.00000	0.00000	0.00000	0.00000	0.00860	0.00712	0.01557	0.01140	0.02133	0.01532
Q5SUF2	LC7L3_MOUSE	0.00000	0.00000	0.00000	0.00000	0.00000	0.00000	0.00000	0.00288	0.00442	0.00363	0.00216	0.00148
Q5SUR0	PUR4_MOUSE	0.00000	0.00000	0.00000	0.00000	0.00000	0.00000	0.00000	0.00228	0.00355	0.00573	0.00468	0.00071
Q5SVR0	TBC9B_MOUSE	0.00000	0.00000	0.00000	0.00000	0.00000	0.00000	0.00276	0.00112	0.00381	0.00154	0.00324	0.00166
Q5SW19	CLU_MOUSE	0.00814	0.01154	0.00715	0.00844	0.01506	0.01205	0.00000	0.00000	0.00078	0.00000	0.00000	0.00000
Q5SWT3	S2535_MOUSE	0.00000	0.00000	0.00000	0.00000	0.00000	0.00000	0.00562	0.00147	0.00461	0.00276	0.00361	0.00362
Q5SWU9	ACACA_MOUSE	0.00000	0.00000	0.00000	0.00000	0.00000	0.00000	0.01569	0.00703	0.01545	0.00779	0.00768	0.00781
Q5SWY8	SC5AA_MOUSE	0.00000	0.00000	0.00000	0.00000	0.00000	0.00000	0.00000	0.00000	0.00000	0.00000	0.00000	0.00000
Q5SX39	MYH4_MOUSE	0.00320	0.00297	0.00164	0.00291	0.00242	0.00308	0.07337	0.00016	0.00004	0.00005	0.00005	0.00013
Q5SX40	MYH1_MOUSE	0.00152	0.00120	0.00295	0.00104	0.00142	0.00247	0.82445	0.12195	0.09753	0.13099	0.09138	0.07809
Q5SXY1	CYTSB_MOUSE	0.00000	0.00000	0.00000	0.00000	0.00000	0.00000	0.00000	0.00000	0.00000	0.00000	0.00000	0.00000
Q5SYD0	MYO1D_MOUSE	0.00000	0.00000	0.00000	0.00000	0.00000	0.00000	0.02311	0.02226	0.02155	0.01952	0.02776	0.02269
Q5U3K5	RABL6_MOUSE	0.00000	0.00000	0.00000	0.00000	0.00000	0.00000	0.00000	0.00312	0.00137	0.00169	0.00175	0.00141
Q5U458	DJC11_MOUSE	0.00935	0.04419	0.00360	0.00942	0.00501	0.01100	0.00000	0.00448	0.00369	0.00345	0.00272	0.00446
Q5U4C1	GASP1_MOUSE	0.00000	0.00000	0.00000	0.00000	0.00000	0.00000	0.00414	0.00437	0.00000	0.00000	0.00884	0.02046
Q5U5V2	HYKK_MOUSE	0.00000	0.00000	0.00000	0.00000	0.00000	0.00000	0.00000	0.00198	0.00000	0.00140	0.00123	0.00095
Q5XG73	ACBD5_MOUSE	0.00000	0.00000	0.00000	0.00000	0.00000	0.00000	0.00110	0.00189	0.00142	0.00085	0.00000	0.00000
Q5XJY5	COPD_MOUSE	0.00177	0.00179	0.00175	0.00233	0.00117	0.00172	0.15659	0.11845	0.16401	0.14186	0.19928	0.16229
Q5XJY6	FANCB_MOUSE	0.00000	0.00000	0.00000	0.00000	0.00000	0.00000	0.00000	0.00203	0.00119	0.00274	0.00176	0.00171
Q5XKE0	MYPC2_MOUSE	0.00000	0.00000	0.00000	0.00000	0.00000	0.00000	0.10023	0.00111	0.00199	0.00228	0.00328	0.00297
Q5XKN4	JAGN1_MOUSE	0.00000	0.00000	0.00000	0.00000	0.00000	0.00000	0.00000	0.00494	0.00260	0.00067	0.00061	0.00389
Q60590	A1AG1_MOUSE	0.00000	0.00000	0.00000	0.00000	0.00000	0.00000	0.01754	0.01969	0.01719	0.01738	0.02701	0.01167
Q60597	ODO1_MOUSE	0.95968	0.93086	1.12043	0.85408	1.00299	0.96793	0.08327	0.05165	0.06464	0.05937	0.06049	0.05570
Q60598	SRC8_MOUSE	0.00000	0.00000	0.00000	0.00000	0.00000	0.00000	0.04249	0.04735	0.05164	0.05065	0.03445	0.05114
Q60604	ADSV_MOUSE	0.00000	0.00000	0.00000	0.00000	0.00000	0.00000	0.00000	0.00015	0.00007	0.00013	0.00000	0.00000
Q60605	MYL6_MOUSE	0.00000	0.00000	0.00000	0.00000	0.00000	0.00000	0.22957	0.21425	0.25640	0.21350	0.23972	0.22867
Q60631	GRB2_MOUSE	0.00000	0.00000	0.00000	0.00000	0.00000	0.00000	0.00598	0.00702	0.00474	0.00529	0.00520	0.00531
Q60634	FLOT2_MOUSE	0.00188	0.00487	0.00189	0.00272	0.00226	0.00423	0.01381	0.01588	0.01948	0.01509	0.01832	0.01795
Q60648	SAP3_MOUSE	0.00000	0.00000	0.00000	0.00000	0.00000	0.00000	0.00000	0.00000	0.00013	0.00000	0.00008	0.00016

Q60649	CLPB_MOUSE	0.00000	0.00000	0.00000	0.00000	0.00000	0.00000	0.00000	0.00000	0.00000	0.00000	0.00000	0.00000
Q60668	HNRPD_MOUSE	0.00000	0.00000	0.00000	0.00000	0.00000	0.00000	0.10717	0.07942	0.07808	0.09616	0.07232	0.10973
Q60675	LAMA2_MOUSE	0.11086	0.19791	0.10916	0.15632	0.12065	0.08985	0.02383	0.01186	0.00832	0.00689	0.00946	0.00813
Q60676	PPP5_MOUSE	0.00554	0.00588	0.00504	0.00772	0.00474	0.01130	0.02664	0.02143	0.02154	0.02122	0.01461	0.02262
Q60692	PSB6_MOUSE	0.00000	0.00000	0.00000	0.00000	0.00000	0.00000	0.00000	0.00869	0.00519	0.00963	0.00000	0.00383
Q60710	SAMH1_MOUSE	0.00000	0.00000	0.00000	0.00000	0.00000	0.00000	0.03395	0.03342	0.04751	0.02978	0.05409	0.04147
Q60714	S27A1_MOUSE	0.00000	0.00000	0.00000	0.00000	0.00000	0.00000	0.00507	0.00109	0.00449	0.00194	0.00190	0.00079
Q60715	P4HA1_MOUSE	0.00000	0.00000	0.00000	0.00000	0.00000	0.00000	0.00427	0.00358	0.00895	0.00698	0.00259	0.00305
Q60737	CSK21_MOUSE	0.00561	0.00460	0.00531	0.00408	0.00469	0.00377	0.01954	0.01300	0.01923	0.01745	0.02014	0.01667
Q60738	ZNT1_MOUSE	0.00000	0.00000	0.00000	0.00000	0.00000	0.00000	0.00000	0.00109	0.00217	0.00120	0.00292	0.00034
Q60739	BAG1_MOUSE	0.00000	0.00000	0.00000	0.00000	0.00000	0.00000	0.00229	0.00625	0.00474	0.00542	0.00170	0.00561
Q60749	KHDR1_MOUSE	0.00000	0.00000	0.00000	0.00000	0.00000	0.00000	0.02764	0.01699	0.01926	0.01973	0.02689	0.02037
Q60759	GCDH_MOUSE	0.07114	0.02831	0.01993	0.02792	0.02065	0.02719	0.00095	0.00114	0.00061	0.00023	0.00041	0.00031
Q60770	STXB3_MOUSE	0.00000	0.00000	0.00000	0.00000	0.00000	0.00000	0.00895	0.00809	0.00631	0.00620	0.00687	0.00742
Q60780	GAS7_MOUSE	0.00000	0.00000	0.00000	0.00000	0.00000	0.00000	0.00204	0.00022	0.00022	0.00000	0.00027	0.00000
Q60790	RASA3_MOUSE	0.00000	0.00000	0.00000	0.00000	0.00000	0.00000	0.00652	0.00285	0.00528	0.00436	0.00614	0.00494
Q60825	NPT2A_MOUSE	0.00000	0.00000	0.00000	0.00000	0.00000	0.00000	0.00069	0.00000	0.00046	0.00079	0.00125	0.00000
Q60829	PPR1B_MOUSE	0.00000	0.00000	0.00000	0.00000	0.00000	0.00000	0.00000	0.00000	0.00000	0.00000	0.00000	0.00000
Q60847	COCA1_MOUSE	0.00000	0.00000	0.00000	0.00000	0.00000	0.00000	0.06694	0.05706	0.04452	0.02294	0.06545	0.05023
Q60854	SPB6_MOUSE	0.01416	0.01603	0.01089	0.01504	0.01700	0.01841	0.17351	0.11289	0.11741	0.11220	0.11485	0.13577
Q60864	STIP1_MOUSE	0.00699	0.00730	0.00941	0.01136	0.00682	0.00716	0.02110	0.01797	0.02179	0.02029	0.01801	0.02018
Q60865	CAPR1_MOUSE	0.00000	0.00000	0.00000	0.00000	0.00000	0.00000	0.01197	0.01066	0.01142	0.01047	0.01073	0.01208
Q60866	PTER_MOUSE	0.00000	0.00000	0.00000	0.00000	0.00000	0.00000	0.00000	0.00000	0.00000	0.00000	0.00000	0.00000
Q60870	REEP5_MOUSE	0.00000	0.00000	0.00000	0.00000	0.00000	0.00000	0.00000	0.00825	0.00832	0.00529	0.00251	0.00361
Q8BMJ3	IF1AX_MOUSE	0.00000	0.00000	0.00000	0.00000	0.00000	0.00000	0.00000	0.00362	0.00600	0.00508	0.00256	0.00288
Q60875	ARHG2_MOUSE	0.00000	0.00000	0.00000	0.00000	0.00000	0.00000	0.00133	0.00560	0.00852	0.00591	0.01028	0.00923
Q60902	EP15R_MOUSE	0.00411	0.00486	0.00260	0.00275	0.00234	0.00353	0.01238	0.01394	0.01761	0.01669	0.01254	0.01698
Q60928	GGT1_MOUSE	0.00000	0.00000	0.00000	0.00000	0.00000	0.00000	0.00000	0.00013	0.00000	0.00000	0.00000	0.00019
Q60930	VDAC2_MOUSE	0.14383	0.05773	0.13858	0.15335	0.12314	0.08867	0.05115	0.06814	0.03198	0.04619	0.03363	0.04625
Q60931	VDAC3_MOUSE	0.11725	0.07720	0.09655	0.08346	0.10084	0.13440	0.06210	0.05201	0.02598	0.03769	0.03990	0.04741
Q60932	VDAC1_MOUSE	0.35024	0.23032	0.40474	0.39291	0.39341	0.29100	0.11226	0.09733	0.07205	0.08679	0.08742	0.09278
Q60936	COQ8A_MOUSE	0.02253	0.01388	0.02098	0.01687	0.01108	0.02324	0.00135	0.00000	0.00000	0.00038	0.00000	0.00000
Q60953	PML_MOUSE	0.00000	0.00000	0.00000	0.00000	0.00000	0.00000	0.00270	0.00367	0.00441	0.00356	0.00413	0.00442
Q60960	IMA5_MOUSE	0.00059	0.00055	0.00053	0.00038	0.00067	0.00041	0.00088	0.00263	0.00402	0.00000	0.00188	0.00166

Q60967	PAPS1_MOUSE	0.00000	0.00000	0.00000	0.00000	0.00000	0.00000	0.00000	0.00000	0.00079	0.00000	0.00000	0.00000
Q60972	RBBP4_MOUSE	0.00000	0.00000	0.00000	0.00000	0.00000	0.00000	0.01615	0.01325	0.02433	0.02494	0.01427	0.02002
Q60973	RBBP7_MOUSE	0.00000	0.00000	0.00000	0.00000	0.00000	0.00000	0.02688	0.03164	0.03035	0.02809	0.03205	0.03219
Q60991	CP7B1_MOUSE	0.00000	0.00000	0.00000	0.00000	0.00000	0.00000	0.00000	0.00000	0.00000	0.00103	0.00000	0.00000
Q60994	ADIPO_MOUSE	0.01935	0.01694	0.01044	0.02266	0.01471	0.02189	0.00950	0.01474	0.01970	0.01669	0.01121	0.00725
Q61001	LAMA5_MOUSE	0.00727	0.02276	0.01646	0.00959	0.00701	0.00970	0.21464	0.14625	0.17433	0.11231	0.24160	0.18082
Q61029	LAP2B_MOUSE	0.00266	0.00657	0.00417	0.00493	0.00286	0.00338	0.03458	0.03320	0.04704	0.03723	0.05711	0.05195
Q61033	LAP2A_MOUSE	0.00000	0.00000	0.00000	0.00000	0.00000	0.00000	0.00331	0.00245	0.00369	0.00237	0.00578	0.00412
Q61035	HARS1_MOUSE	0.00000	0.00000	0.00000	0.00000	0.00000	0.00000	0.00609	0.00998	0.01294	0.00812	0.00171	0.01325
Q61074	PPM1G_MOUSE	0.00000	0.00000	0.00000	0.00000	0.00000	0.00000	0.00000	0.00357	0.00218	0.00310	0.00123	0.00534
Q61081	CDC37_MOUSE	0.00476	0.00664	0.00250	0.00290	0.00383	0.00623	0.03575	0.02773	0.04553	0.05017	0.03334	0.03939
Q61087	LAMB3_MOUSE	0.00000	0.00000	0.00000	0.00000	0.00000	0.00000	0.04373	0.03915	0.05069	0.03375	0.05734	0.05397
Q61092	LAMC2_MOUSE	0.00000	0.00000	0.00000	0.00000	0.00000	0.00000	0.04529	0.02063	0.03398	0.02051	0.06836	0.03776
Q61093	CY24B_MOUSE	0.00000	0.00000	0.00000	0.00000	0.00000	0.00000	0.00884	0.01267	0.00999	0.00974	0.01154	0.01796
Q61102	ABCB7_MOUSE	0.00000	0.00000	0.00000	0.00000	0.00000	0.00000	0.00449	0.00444	0.00536	0.00419	0.00161	0.00238
Q61103	REQU_MOUSE	0.00000	0.00000	0.00000	0.00000	0.00000	0.00000	0.00362	0.00151	0.00412	0.00373	0.00408	0.00420
Q61107	GBP4_MOUSE	0.00000	0.00000	0.00000	0.00000	0.00000	0.00000	0.00000	0.00000	0.00247	0.00170	0.00035	0.00044
Q61114	BPIB1_MOUSE	0.00000	0.00000	0.00000	0.00000	0.00000	0.00000	0.00179	0.00000	0.00449	0.00179	0.00507	0.00389
Q61129	CFAI_MOUSE	0.00000	0.00000	0.00000	0.00000	0.00000	0.00000	0.00000	0.00000	0.00066	0.00199	0.00171	0.00062
Q61133	GSTT2_MOUSE	0.00000	0.00000	0.00000	0.00000	0.00000	0.00000	0.00980	0.00770	0.00753	0.00890	0.00583	0.00896
Q61146	OCLN_MOUSE	0.00000	0.00000	0.00000	0.00000	0.00000	0.00000	0.00419	0.00839	0.01145	0.00920	0.01477	0.00758
Q61147	CERU_MOUSE	0.00564	0.00209	0.00467	0.00454	0.00306	0.00239	0.01355	0.01565	0.01847	0.02324	0.02420	0.01569
Q61151	2A5E_MOUSE	0.00060	0.00127	0.00070	0.00029	0.00144	0.00153	0.00030	0.00000	0.00034	0.00060	0.00101	0.00084
Q61165	SL9A1_MOUSE	0.00000	0.00000	0.00000	0.00000	0.00000	0.00000	0.00000	0.00204	0.00195	0.00127	0.00192	0.00158
Q61166	MARE1_MOUSE	0.00441	0.00750	0.00549	0.00379	0.00612	0.00545	0.01480	0.01606	0.01970	0.02118	0.01456	0.01910
Q61171	PRDX2_MOUSE	0.04809	0.03861	0.06557	0.07182	0.07180	0.06794	0.23185	0.08888	0.12812	0.21909	0.33768	0.14849
Q61187	TS101_MOUSE	0.00000	0.00000	0.00000	0.00000	0.00000	0.00000	0.00347	0.00174	0.00379	0.00275	0.00417	0.00266
Q61189	ICLN_MOUSE	0.00000	0.00000	0.00000	0.00000	0.00000	0.00000	0.00119	0.00000	0.00333	0.00000	0.00398	0.00253
Q61191	HCFC1_MOUSE	0.00000	0.00000	0.00000	0.00000	0.00000	0.00000	0.00577	0.00361	0.00848	0.00520	0.00000	0.00626
Q61206	PA1B2_MOUSE	0.00000	0.00000	0.00000	0.00000	0.00000	0.00000	0.04603	0.03209	0.04141	0.03327	0.04417	0.03802
Q61210	ARHG1_MOUSE	0.00000	0.00000	0.00000	0.00000	0.00000	0.00000	0.01005	0.01443	0.02093	0.01668	0.01538	0.01984
Q61211	EIF2D_MOUSE	0.00000	0.00000	0.00000	0.00000	0.00000	0.00000	0.00000	0.00132	0.01201	0.00546	0.00000	0.00000
Q61233	PLSL_MOUSE	0.00475	0.00379	0.00399	0.00753	0.00660	0.00458	0.05396	0.05952	0.06412	0.07388	0.07018	0.08432
Q61234	SNTA1_MOUSE	0.01986	0.01513	0.01521	0.01155	0.01936	0.02350	0.00484	0.00280	0.00463	0.00267	0.00341	0.00329

Q61235	SNTB2_MOUSE	0.00000	0.00000	0.00000	0.00000	0.00000	0.00000	0.01961	0.01449	0.01923	0.01703	0.02051	0.01999
Q61247	A2AP_MOUSE	0.00000	0.00000	0.00000	0.00000	0.00000	0.00000	0.00963	0.00607	0.00920	0.00890	0.01243	0.00536
Q61249	IGBP1_MOUSE	0.00000	0.00000	0.00000	0.00000	0.00000	0.00000	0.00210	0.00689	0.00555	0.00517	0.00437	0.00444
Q61263	SOAT1_MOUSE	0.00000	0.00000	0.00000	0.00000	0.00000	0.00000	0.00123	0.00565	0.00728	0.00368	0.00441	0.00430
Q61282	PGCA_MOUSE	0.00000	0.00000	0.00000	0.00000	0.00000	0.00000	0.06290	0.00000	0.00000	0.00075	0.02394	0.00000
Q61288	ACVL1_MOUSE	0.00000	0.00000	0.00000	0.00000	0.00000	0.00000	0.00479	0.00340	0.00480	0.00350	0.00475	0.00459
Q61290	CAC1E_MOUSE	0.00000	0.00000	0.00000	0.00000	0.00000	0.00000	0.00628	0.00276	0.01101	0.00731	0.00612	0.00687
Q61292	LAMB2_MOUSE	0.02939	0.05052	0.02810	0.04466	0.03338	0.02111	0.13075	0.11823	0.11799	0.07867	0.13768	0.13182
Q61301	CTNA2_MOUSE	0.00000	0.00000	0.00000	0.00000	0.00000	0.00000	0.01321	0.00733	0.01107	0.00925	0.01369	0.01047
Q61316	HSP74_MOUSE	0.03074	0.03077	0.02973	0.02659	0.03624	0.03050	0.03057	0.03365	0.04036	0.03925	0.02787	0.03872
Q61334	BAP29_MOUSE	0.00000	0.00000	0.00000	0.00000	0.00000	0.00000	0.00000	0.00154	0.00286	0.00202	0.00169	0.00083
Q61335	BAP31_MOUSE	0.00423	0.00108	0.00217	0.00141	0.00049	0.00000	0.00706	0.02797	0.02950	0.02142	0.02002	0.01874
Q61362	CH3L1_MOUSE	0.00000	0.00000	0.00000	0.00000	0.00000	0.00000	0.01857	0.01166	0.01962	0.01588	0.01689	0.02455
Q61391	NEP_MOUSE	0.00000	0.00000	0.00000	0.00000	0.00000	0.00000	0.01914	0.02069	0.02670	0.02648	0.02913	0.03057
Q61398	PCOC1_MOUSE	0.00000	0.00000	0.00000	0.00000	0.00000	0.00000	0.00106	0.00410	0.00000	0.00000	0.00036	0.00045
Q61411	RASH_MOUSE	0.00000	0.00000	0.00000	0.00000	0.00000	0.00000	0.04331	0.02262	0.03141	0.03176	0.03506	0.03254
Q61425	HCDH_MOUSE	0.15789	0.11288	0.24154	0.28067	0.33046	0.17295	0.05877	0.03397	0.04036	0.02936	0.04796	0.04071
Q61469	PLPP1_MOUSE	0.00000	0.00000	0.00000	0.00000	0.00000	0.00000	0.00000	0.00332	0.00585	0.00341	0.00154	0.00146
Q61490	CD166_MOUSE	0.00000	0.00000	0.00000	0.00000	0.00000	0.00000	0.03433	0.04523	0.04407	0.04388	0.05262	0.04816
Q61503	5NTD_MOUSE	0.00000	0.00000	0.00000	0.00000	0.00000	0.00000	0.02872	0.02147	0.02365	0.02995	0.02918	0.02593
Q61508	ECM1_MOUSE	0.00000	0.00000	0.00000	0.00000	0.00000	0.00000	0.00658	0.00804	0.00416	0.00514	0.00108	0.00133
Q61510	TRI25_MOUSE	0.00000	0.00000	0.00000	0.00000	0.00000	0.00000	0.00578	0.01396	0.00750	0.00970	0.00660	0.00907
Q61543	GSLG1_MOUSE	0.01230	0.00843	0.01380	0.01544	0.01089	0.00947	0.00395	0.00340	0.00502	0.00322	0.00354	0.00273
Q61545	EWS_MOUSE	0.00000	0.00000	0.00000	0.00000	0.00000	0.00000	0.01206	0.01642	0.01167	0.01525	0.00656	0.00750
Q61550	RAD21_MOUSE	0.00000	0.00000	0.00000	0.00000	0.00000	0.00000	0.00000	0.00131	0.00081	0.00000	0.00000	0.00196
Q61553	FSCN1_MOUSE	0.00000	0.00000	0.00000	0.00000	0.00000	0.00000	0.00528	0.00525	0.00725	0.00657	0.00372	0.00437
Q61554	FBN1_MOUSE	0.04244	0.08226	0.06446	0.03992	0.05792	0.03285	0.00442	0.01861	0.02047	0.00899	0.01145	0.00691
Q61581	IBP7_MOUSE	0.00000	0.00000	0.00000	0.00000	0.00000	0.00000	0.00166	0.00170	0.00208	0.00230	0.00231	0.00223
Q61584	FXR1_MOUSE	0.00144	0.00098	0.00129	0.00116	0.00072	0.00169	0.00244	0.00620	0.00310	0.00791	0.00132	0.00213
Q61586	GPAT1_MOUSE	0.00000	0.00000	0.00000	0.00000	0.00000	0.00000	0.00000	0.00000	0.00057	0.00000	0.00000	0.00000
Q61595	KTN1_MOUSE	0.00209	0.00340	0.00199	0.00250	0.00224	0.00107	0.00288	0.00171	0.00438	0.00274	0.00440	0.00229
Q61598	GDIB_MOUSE	0.03323	0.04020	0.03190	0.03726	0.04192	0.03124	0.05894	0.06647	0.07046	0.06773	0.07204	0.06560
Q61599	GDIR2_MOUSE	0.00000	0.00000	0.00000	0.00000	0.00000	0.00000	0.08920	0.07313	0.09647	0.10274	0.10878	0.12901
Q61646	HPT_MOUSE	0.00000	0.00000	0.00000	0.00000	0.00000	0.00000	0.01778	0.05501	0.01044	0.00893	0.01168	0.02135

Q61655	DD19A_MOUSE	0.00000	0.00000	0.00000	0.00000	0.00000	0.00000	0.00239	0.00897	0.00494	0.00418	0.00598	0.00575
Q61656	DDX5_MOUSE	0.00000	0.00000	0.00000	0.00000	0.00000	0.00000	0.01216	0.02032	0.01560	0.01789	0.01682	0.01976
Q61686	CBX5_MOUSE	0.00000	0.00000	0.00000	0.00000	0.00000	0.00000	0.00000	0.01383	0.00696	0.00658	0.00638	0.00566
Q61699	HS105_MOUSE	0.00000	0.00000	0.00000	0.00000	0.00000	0.00000	0.01422	0.01793	0.01286	0.01458	0.01437	0.01522
Q61702	ITIH1_MOUSE	0.00131	0.00119	0.00116	0.00157	0.00126	0.00137	0.00662	0.01487	0.01029	0.01377	0.02711	0.00862
Q61703	ITIH2_MOUSE	0.00000	0.00000	0.00000	0.00000	0.00000	0.00000	0.00297	0.00189	0.00322	0.00501	0.00868	0.00099
Q61733	RT31_MOUSE	0.00000	0.00000	0.00000	0.00000	0.00000	0.00000	0.00000	0.00000	0.00000	0.00000	0.00000	0.00000
Q61735	CD47_MOUSE	0.00000	0.00000	0.00000	0.00000	0.00000	0.00000	0.02841	0.06414	0.05638	0.06175	0.03066	0.05981
Q61739	ITA6_MOUSE	0.00332	0.00387	0.00253	0.00253	0.00217	0.00305	0.01223	0.00727	0.01305	0.00999	0.01276	0.01374
Q61753	SERA_MOUSE	0.00000	0.00000	0.00000	0.00000	0.00000	0.00000	0.00000	0.00000	0.00000	0.00000	0.00000	0.00000
Q61767	3BHS4_MOUSE	0.00000	0.00000	0.00000	0.00000	0.00000	0.00000	0.00019	0.00010	0.00005	0.00009	0.00008	0.00000
Q61768	KINH_MOUSE	0.02262	0.02276	0.01655	0.01561	0.02102	0.01849	0.02039	0.01610	0.01801	0.01677	0.01631	0.01971
Q61781	K1C14_MOUSE	0.00000	0.00000	0.00000	0.00000	0.00000	0.00000	0.01859	0.00088	0.00231	0.00000	0.00077	0.00398
Q61789	LAMA3_MOUSE	0.00000	0.00000	0.00000	0.00000	0.00000	0.00000	0.05915	0.03805	0.04797	0.03044	0.07980	0.05763
Q61792	LASP1_MOUSE	0.00000	0.00000	0.00000	0.00000	0.00000	0.00000	0.00177	0.03255	0.02715	0.03193	0.01499	0.02533
Q61830	MRC1_MOUSE	0.00548	0.00304	0.00337	0.00249	0.00236	0.00343	0.00647	0.00370	0.00491	0.00226	0.00246	0.00197
Q61838	PZP_MOUSE	0.04816	0.02267	0.02722	0.07146	0.03243	0.02451	0.19444	0.05237	0.09457	0.26379	0.33407	0.05131
Q61847	MEP1B_MOUSE	0.00000	0.00000	0.00000	0.00000	0.00000	0.00000	0.00000	0.00000	0.00000	0.00000	0.00000	0.00139
Q61878	PRG2_MOUSE	0.00000	0.00000	0.00000	0.00000	0.00000	0.00000	0.01185	0.04864	0.01495	0.03216	0.01921	0.06513
Q61879	MYH10_MOUSE	0.00431	0.00464	0.00673	0.00584	0.00762	0.00448	0.09785	0.09595	0.10254	0.09607	0.11549	0.11063
Q61937	NPM_MOUSE	0.00422	0.00679	0.00660	0.00649	0.00748	0.00607	0.05170	0.03256	0.04297	0.03965	0.05324	0.04306
Q61990	PCBP2_MOUSE	0.00019	0.00021	0.00076	0.00045	0.00011	0.00053	0.05247	0.06260	0.07038	0.07399	0.05552	0.08328
Q62000	MIME_MOUSE	0.00012	0.00013	0.00128	0.00178	0.00228	0.00399	0.12788	0.12494	0.15201	0.11599	0.15350	0.13198
Q62009	POSTN_MOUSE	0.01267	0.01917	0.01525	0.01389	0.01320	0.00977	0.12806	0.07129	0.09442	0.06543	0.15279	0.11800
Q62048	PEA15_MOUSE	0.00000	0.00000	0.00000	0.00000	0.00000	0.00000	0.02807	0.03071	0.04129	0.04319	0.05381	0.03225
Q62074	KPCI_MOUSE	0.00000	0.00000	0.00000	0.00000	0.00000	0.00000	0.00709	0.01071	0.01083	0.00939	0.00952	0.01032
Q62077	PLCG1_MOUSE	0.00000	0.00000	0.00000	0.00000	0.00000	0.00000	0.00033	0.00259	0.00149	0.00036	0.00415	0.00163
Q62086	PON2_MOUSE	0.00000	0.00000	0.00000	0.00000	0.00000	0.00000	0.00416	0.00399	0.01104	0.00822	0.01150	0.00637
Q62087	PON3_MOUSE	0.00000	0.00000	0.00000	0.00000	0.00000	0.00000	0.07024	0.06184	0.06845	0.05845	0.05244	0.08212
Q62093	SRSF2_MOUSE	0.00400	0.00149	0.00450	0.00665	0.00311	0.00431	0.03250	0.04863	0.03767	0.04761	0.05653	0.03624
Q62095	DDX3Y_MOUSE	0.00000	0.00000	0.00000	0.00000	0.00000	0.00000	0.00636	0.00721	0.01065	0.00842	0.01190	0.00766
Q62148	AL1A2_MOUSE	0.00000	0.00000	0.00000	0.00000	0.00000	0.00000	0.00661	0.00746	0.01439	0.00889	0.00845	0.00816
Q62151	RAGE_MOUSE	0.00000	0.00000	0.00000	0.00000	0.00000	0.00000	0.44220	0.91392	0.64256	0.67273	0.53278	0.69009
Q62165	DAG1_MOUSE	0.00569	0.00692	0.01003	0.00898	0.01100	0.00596	0.02793	0.03017	0.03073	0.02923	0.02574	0.02992

Q62167	DDX3X_MOUSE	0.00367	0.00704	0.00469	0.00638	0.00385	0.00433	0.03153	0.03013	0.02867	0.02558	0.02438	0.02879
Q62186	SSRD_MOUSE	0.00000	0.00000	0.00000	0.00000	0.00000	0.00000	0.07310	0.04831	0.08453	0.06672	0.08471	0.06635
Q62188	DPYL3_MOUSE	0.00254	0.00383	0.00352	0.00321	0.00406	0.00370	0.04301	0.03987	0.04225	0.03666	0.04651	0.04276
Q62189	SNRPA_MOUSE	0.00000	0.00000	0.00000	0.00000	0.00000	0.00000	0.00519	0.00552	0.00819	0.00571	0.01100	0.00873
Q62219	TGFI1_MOUSE	0.00000	0.00000	0.00000	0.00000	0.00000	0.00000	0.01041	0.02697	0.02471	0.02029	0.02262	0.02395
Q62234	MYOM1_MOUSE	0.46123	0.46206	0.37754	0.38119	0.48552	0.44529	0.02474	0.00280	0.00539	0.00577	0.00453	0.00365
Q62241	RU1C_MOUSE	0.00000	0.00000	0.00000	0.00000	0.00000	0.00000	0.00178	0.00785	0.00882	0.00979	0.00589	0.01157
Q62261	SPTB2_MOUSE	0.25261	0.21113	0.19059	0.15814	0.21030	0.17292	0.14108	0.13387	0.15480	0.14518	0.14655	0.14819
Q62264	THRSP_MOUSE	0.00000	0.00000	0.00000	0.00000	0.00000	0.00000	0.00000	0.00000	0.00786	0.00000	0.00000	0.00000
Q62283	TSN7_MOUSE	0.00000	0.00000	0.00000	0.00000	0.00000	0.00000	0.00000	0.00920	0.01659	0.01388	0.00994	0.00759
Q62312	TGFR2_MOUSE	0.00000	0.00000	0.00000	0.00000	0.00000	0.00000	0.00000	0.00071	0.00000	0.00000	0.00000	0.00336
Q62313	TGON1_MOUSE	0.00000	0.00000	0.00000	0.00000	0.00000	0.00000	0.00000	0.00000	0.00136	0.00000	0.00000	0.00017
Q62318	TIF1B_MOUSE	0.00000	0.00000	0.00000	0.00000	0.00000	0.00000	0.03177	0.03200	0.03651	0.03203	0.03443	0.04606
Q62348	TSN_MOUSE	0.00000	0.00000	0.00000	0.00000	0.00000	0.00000	0.01452	0.01280	0.01909	0.01877	0.02372	0.01822
Q62351	TFR1_MOUSE	0.00000	0.00000	0.00000	0.00000	0.00000	0.00000	0.00125	0.00109	0.00156	0.00130	0.00179	0.00053
Q62376	RU17_MOUSE	0.00000	0.00000	0.00000	0.00000	0.00000	0.00000	0.01134	0.01308	0.01686	0.01368	0.01258	0.01302
Q62393	TPD52_MOUSE	0.00000	0.00000	0.00000	0.00000	0.00000	0.00000	0.00717	0.01012	0.00818	0.00552	0.00758	0.01111
Q62417	SRBS1_MOUSE	0.09127	0.05072	0.04075	0.04748	0.04978	0.05071	0.01141	0.01502	0.01856	0.01410	0.02039	0.01585
Q62418	DBNL_MOUSE	0.00291	0.00295	0.00227	0.00199	0.00306	0.00426	0.02727	0.03163	0.03986	0.04184	0.03839	0.03921
Q62419	SH3G1_MOUSE	0.00000	0.00000	0.00000	0.00000	0.00000	0.00000	0.00659	0.00957	0.00781	0.00986	0.00679	0.01314
Q62422	OSTF1_MOUSE	0.00000	0.00000	0.00000	0.00000	0.00000	0.00000	0.02107	0.01474	0.02150	0.02139	0.02741	0.02488
Q62425	NDUA4_MOUSE	0.00000	0.00000	0.00000	0.00000	0.00000	0.00000	0.00110	0.15237	0.08495	0.01920	0.03092	0.14102
Q62433	NDRG1_MOUSE	0.00000	0.00000	0.00000	0.00000	0.00000	0.00000	0.00347	0.00258	0.00508	0.00346	0.00428	0.00283
Q62446	FKBP3_MOUSE	0.00191	0.00311	0.00378	0.00276	0.00145	0.00114	0.00441	0.00440	0.00682	0.00657	0.00546	0.00530
Q62448	IF4G2_MOUSE	0.00023	0.00020	0.00048	0.00039	0.00085	0.00040	0.01197	0.00668	0.00961	0.01006	0.01081	0.01166
Q62465	VAT1_MOUSE	0.00460	0.00374	0.00340	0.00314	0.00302	0.00423	0.08925	0.10053	0.08966	0.09335	0.08455	0.09886
Q62468	VILI_MOUSE	0.00000	0.00000	0.00000	0.00000	0.00000	0.00000	0.00006	0.00005	0.00005	0.00000	0.00005	0.00003
Q62469	ITA2_MOUSE	0.00000	0.00000	0.00000	0.00000	0.00000	0.00000	0.00933	0.00810	0.01585	0.01412	0.01639	0.01623
Q62470	ITA3_MOUSE	0.00000	0.00000	0.00000	0.00000	0.00000	0.00000	0.01894	0.02138	0.02068	0.01613	0.02288	0.02145
Q62523	ZYX_MOUSE	0.00000	0.00000	0.00000	0.00000	0.00000	0.00000	0.00164	0.04071	0.06791	0.05377	0.02905	0.03024
Q63810	CANB1_MOUSE	0.00000	0.00000	0.00000	0.00000	0.00000	0.00000	0.01153	0.01029	0.00418	0.00718	0.00842	0.00350
Q63844	MK03_MOUSE	0.00000	0.00000	0.00000	0.00000	0.00000	0.00000	0.03710	0.03438	0.04995	0.03979	0.04818	0.03579
Q63850	NUP62_MOUSE	0.00000	0.00000	0.00000	0.00000	0.00000	0.00000	0.00452	0.00180	0.00289	0.00286	0.00354	0.00595
Q63870	CO7A1_MOUSE	0.00000	0.00000	0.00000	0.00000	0.00000	0.00000	0.01074	0.00000	0.00000	0.00000	0.00000	0.00108

Q63918	CAVN2_MOUSE	0.04591	0.03328	0.03218	0.02928	0.04410	0.03378	0.25435	0.29299	0.38924	0.32721	0.42030	0.34794
Q63932	MP2K2_MOUSE	0.00223	0.00151	0.00217	0.00203	0.00157	0.00213	0.00375	0.01167	0.01757	0.01360	0.00825	0.01489
Q63961	EGLN_MOUSE	0.00000	0.00000	0.00000	0.00000	0.00000	0.00000	0.00434	0.01111	0.00662	0.00854	0.00499	0.01276
Q63ZW7	INADL_MOUSE	0.00000	0.00000	0.00000	0.00000	0.00000	0.00000	0.00764	0.01004	0.01445	0.00951	0.01970	0.01486
Q64010	CRK_MOUSE	0.00000	0.00000	0.00000	0.00000	0.00000	0.00000	0.01532	0.01162	0.01304	0.01507	0.01485	0.01624
Q64012	RALY_MOUSE	0.00000	0.00000	0.00000	0.00000	0.00000	0.00000	0.00104	0.00881	0.00961	0.00720	0.00409	0.00441
Q640N1	AEBP1_MOUSE	0.00000	0.00000	0.00000	0.00000	0.00000	0.00000	0.00135	0.00181	0.00273	0.00042	0.00000	0.00304
Q64105	SPRE_MOUSE	0.01671	0.01183	0.01424	0.01675	0.01292	0.01720	0.03178	0.02400	0.02710	0.02973	0.02331	0.04041
Q64133	AOFA_MOUSE	0.00247	0.00332	0.00145	0.00222	0.00152	0.00172	0.02947	0.02431	0.04114	0.02852	0.03613	0.03026
Q64152	BTF3_MOUSE	0.00000	0.00000	0.00000	0.00000	0.00000	0.00000	0.01556	0.00933	0.01275	0.01088	0.01115	0.01322
Q64213	SF01_MOUSE	0.00000	0.00000	0.00000	0.00000	0.00000	0.00000	0.00000	0.00301	0.00207	0.00325	0.00159	0.00493
Q64282	IFIT1_MOUSE	0.00000	0.00000	0.00000	0.00000	0.00000	0.00000	0.00000	0.00000	0.00330	0.00091	0.00176	0.00000
Q64310	SURF4_MOUSE	0.00352	0.00638	0.00955	0.00571	0.00827	0.00715	0.01533	0.02155	0.01121	0.01283	0.01602	0.01143
Q64314	CD34_MOUSE	0.00000	0.00000	0.00000	0.00000	0.00000	0.00000	0.00000	0.00825	0.01321	0.00667	0.00459	0.00436
Q64324	STXB2_MOUSE	0.00000	0.00000	0.00000	0.00000	0.00000	0.00000	0.00625	0.00599	0.01061	0.00918	0.00845	0.00924
Q64331	MYO6_MOUSE	0.00000	0.00000	0.00000	0.00000	0.00000	0.00000	0.01469	0.01158	0.01649	0.01528	0.01413	0.01505
Q64337	SQSTM_MOUSE	0.00000	0.00000	0.00000	0.00000	0.00000	0.00000	0.00189	0.00238	0.00628	0.00282	0.00174	0.00398
Q64339	ISG15_MOUSE	0.00000	0.00000	0.00000	0.00000	0.00000	0.00000	0.00995	0.00891	0.00836	0.01070	0.01676	0.00807
Q64345	IFIT3_MOUSE	0.00000	0.00000	0.00000	0.00000	0.00000	0.00000	0.00000	0.00000	0.00000	0.00000	0.00000	0.00926
Q64373	B2CL1_MOUSE	0.00000	0.00000	0.00000	0.00000	0.00000	0.00000	0.00150	0.00657	0.00109	0.00163	0.00000	0.00360
Q64374	RGN_MOUSE	0.00000	0.00000	0.00000	0.00000	0.00000	0.00000	0.00056	0.00000	0.00000	0.00000	0.00074	0.00000
Q64378	FKBP5_MOUSE	0.00000	0.00000	0.00000	0.00000	0.00000	0.00000	0.00641	0.00495	0.00772	0.00574	0.00900	0.00459
Q64433	CH10_MOUSE	0.00000	0.00000	0.00000	0.00000	0.00000	0.00000	0.02252	0.01642	0.01312	0.01399	0.01194	0.01390
Q64435	UD16_MOUSE	0.00000	0.00000	0.00000	0.00000	0.00000	0.00000	0.01600	0.02030	0.01184	0.01229	0.01453	0.01726
Q64437	ADH7_MOUSE	0.00000	0.00000	0.00000	0.00000	0.00000	0.00000	0.02880	0.00719	0.01148	0.00807	0.01931	0.00855
Q64442	DHSO_MOUSE	0.00187	0.00322	0.00197	0.00205	0.00375	0.00214	0.03518	0.02685	0.03926	0.04184	0.04336	0.03611
Q64444	CAH4_MOUSE	0.00000	0.00000	0.00000	0.00000	0.00000	0.00000	0.02387	0.01679	0.02718	0.01978	0.03587	0.02615
Q64449	MRC2_MOUSE	0.00000	0.00000	0.00000	0.00000	0.00000	0.00000	0.02181	0.00425	0.01991	0.00945	0.01055	0.00674
Q64462	CP4B1_MOUSE	0.00000	0.00000	0.00000	0.00000	0.00000	0.00000	0.10502	0.07599	0.06073	0.05564	0.07093	0.06423
Q64471	GSTT1_MOUSE	0.00501	0.00576	0.00506	0.00529	0.00439	0.00497	0.04763	0.05438	0.06497	0.05604	0.06400	0.05296
Q64511	TOP2B_MOUSE	0.00000	0.00000	0.00000	0.00000	0.00000	0.00000	0.01163	0.01096	0.01180	0.01108	0.01408	0.01254
Q64514	TPP2_MOUSE	0.00207	0.00565	0.00250	0.00243	0.00215	0.00352	0.01195	0.00847	0.00769	0.00865	0.00804	0.01078
Q64516	GLPK_MOUSE	0.00354	0.00774	0.00383	0.00501	0.00542	0.00289	0.00012	0.00004	0.00015	0.00005	0.00000	0.00005
Q64518	AT2A3_MOUSE	0.00023	0.00000	0.00000	0.00028	0.00018	0.00000	0.04699	0.02994	0.03075	0.03399	0.03802	0.03523

Q64521	GPDM_MOUSE	0.00306	0.00296	0.00300	0.00361	0.00321	0.00183	0.02789	0.02228	0.02114	0.01720	0.01611	0.02152
Q6GSS7	H2A2A_MOUSE	0.00000	0.00000	0.00000	0.00000	0.00000	0.00000	0.42951	0.40373	0.52569	0.59347	0.61256	0.77121
Q64669	NQO1_MOUSE	0.01769	0.01995	0.01014	0.00736	0.01237	0.01339	0.00681	0.00664	0.00724	0.00863	0.00939	0.00764
Q64674	SPEE_MOUSE	0.00000	0.00000	0.00000	0.00000	0.00000	0.00000	0.00221	0.00891	0.00528	0.00508	0.00816	0.00441
Q64704	STX3_MOUSE	0.00000	0.00000	0.00000	0.00000	0.00000	0.00000	0.00000	0.00696	0.00248	0.00281	0.00069	0.00291
Q64726	ZA2G_MOUSE	0.00000	0.00000	0.00000	0.00000	0.00000	0.00000	0.00754	0.00941	0.00458	0.00880	0.00335	0.00546
Q64727	VINC_MOUSE	0.09728	0.06755	0.08944	0.08318	0.08866	0.08893	0.14730	0.23054	0.19930	0.21839	0.18677	0.22980
Q64735	CR1L_MOUSE	0.00033	0.00000	0.00047	0.00033	0.00017	0.00021	0.00082	0.00787	0.01773	0.01191	0.00893	0.00708
Q64737	PUR2_MOUSE	0.00000	0.00000	0.00000	0.00000	0.00000	0.00000	0.01052	0.01111	0.01009	0.00957	0.00901	0.00938
Q64FW2	RETST_MOUSE	0.00000	0.00000	0.00000	0.00000	0.00000	0.00000	0.00000	0.00142	0.00339	0.00252	0.00164	0.00397
Q68EF0	RAB3I_MOUSE	0.00000	0.00000	0.00000	0.00000	0.00000	0.00000	0.00000	0.00000	0.00000	0.00000	0.00000	0.00000
Q68FD5	CLH1_MOUSE	0.03523	0.04310	0.04526	0.04210	0.03814	0.03824	0.15878	0.18248	0.19968	0.18030	0.19559	0.17688
Q68FE6	RIPR1_MOUSE	0.00000	0.00000	0.00000	0.00000	0.00000	0.00000	0.00532	0.00385	0.00446	0.00405	0.00509	0.00461
Q68FF6	GIT1_MOUSE	0.00000	0.00000	0.00000	0.00000	0.00000	0.00000	0.00164	0.00181	0.00210	0.00150	0.00335	0.00176
Q68FH4	GALK2_MOUSE	0.00000	0.00000	0.00000	0.00000	0.00000	0.00000	0.00910	0.00909	0.00749	0.00741	0.00874	0.00920
Q68FL4	SAHH3_MOUSE	0.00000	0.00000	0.00000	0.00000	0.00000	0.00000	0.00000	0.01175	0.01391	0.00993	0.00648	0.00697
Q68FL6	SYMC_MOUSE	0.00366	0.00416	0.00261	0.00298	0.00423	0.00255	0.01496	0.01189	0.00880	0.01014	0.01139	0.01319
Q69Z37	SAM9L_MOUSE	0.00000	0.00000	0.00000	0.00000	0.00000	0.00000	0.00000	0.00000	0.00000	0.00000	0.00000	0.00000
Q69Z99	ZN512_MOUSE	0.00000	0.00000	0.00000	0.00000	0.00000	0.00000	0.00000	0.00000	0.00044	0.00000	0.00162	0.00034
Q69ZN7	MYOF_MOUSE	0.00000	0.00000	0.00000	0.00000	0.00000	0.00000	0.04064	0.04892	0.04669	0.04847	0.04938	0.04627
Q69ZX8	ABLM3_MOUSE	0.00000	0.00000	0.00000	0.00000	0.00000	0.00000	0.00640	0.00756	0.00656	0.00744	0.01259	0.00331
Q6A026	PDS5A_MOUSE	0.00000	0.00000	0.00000	0.00000	0.00000	0.00000	0.00000	0.00286	0.00530	0.00444	0.00328	0.00639
Q6A028	SWP70_MOUSE	0.00000	0.00000	0.00000	0.00000	0.00000	0.00000	0.00637	0.01207	0.00889	0.01066	0.00829	0.01338
Q6A068	CDC5L_MOUSE	0.00000	0.00000	0.00000	0.00000	0.00000	0.00000	0.00695	0.00620	0.01011	0.00617	0.00646	0.01208
Q6A0A9	F120A_MOUSE	0.00000	0.00000	0.00000	0.00000	0.00000	0.00000	0.00078	0.00530	0.00827	0.00558	0.00547	0.00703
Q6A0D4	RFTN1_MOUSE	0.00000	0.00000	0.00000	0.00000	0.00000	0.00000	0.00000	0.00000	0.00149	0.00000	0.00000	0.00000
Q6A4J8	UBP7_MOUSE	0.00000	0.00000	0.00000	0.00000	0.00000	0.00000	0.01165	0.00634	0.01437	0.00837	0.01378	0.00826
Q6A4L0	S22AD_MOUSE	0.00000	0.00000	0.00000	0.00000	0.00000	0.00000	0.00014	0.00037	0.00000	0.00039	0.00060	0.00000
Q6AW69	CGNL1_MOUSE	0.00000	0.00000	0.00000	0.00000	0.00000	0.00000	0.00304	0.00419	0.00393	0.00359	0.00447	0.00427
Q6DFW4	NOP58_MOUSE	0.00000	0.00000	0.00000	0.00000	0.00000	0.00000	0.00448	0.00518	0.00760	0.00477	0.01145	0.00793
Q6DIC0	SMCA2_MOUSE	0.00000	0.00000	0.00000	0.00000	0.00000	0.00000	0.00231	0.00629	0.00204	0.00213	0.00086	0.00276
Q6DID3	SCAF8_MOUSE	0.00000	0.00000	0.00000	0.00000	0.00000	0.00000	0.00139	0.00297	0.00513	0.00247	0.00488	0.00567
Q6DVA0	LEMD2_MOUSE	0.00000	0.00000	0.00000	0.00000	0.00000	0.00000	0.00000	0.00158	0.00000	0.00000	0.00000	0.00000
Q6DYE8	ENPP3_MOUSE	0.00000	0.00000	0.00000	0.00000	0.00000	0.00000	0.00000	0.00000	0.00000	0.00000	0.00000	0.00000

Q6EDY6	CARL1_MOUSE	0.00000	0.00000	0.00000	0.00000	0.00000	0.00000	0.00660	0.00948	0.00745	0.00644	0.00891	0.00954
Q6GQT9	NOMO1_MOUSE	0.00078	0.00218	0.00087	0.00776	0.00091	0.00135	0.00336	0.00555	0.00873	0.00807	0.00553	0.00580
Q6GV12	KDSR_MOUSE	0.00000	0.00000	0.00000	0.00000	0.00000	0.00000	0.00000	0.01124	0.00117	0.00000	0.00160	0.00404
Q6GYP7	RGPA1_MOUSE	0.00000	0.00000	0.00000	0.00000	0.00000	0.00000	0.02583	0.01495	0.02938	0.01937	0.02499	0.01890
Q6IRU2	TPM4_MOUSE	0.01870	0.00936	0.02047	0.01770	0.01409	0.01384	0.11677	0.12824	0.15677	0.16726	0.13171	0.13251
Q6IRU5	CLCB_MOUSE	0.00000	0.00000	0.00000	0.00000	0.00000	0.00000	0.00113	0.01730	0.02193	0.02424	0.00944	0.00995
Q6KAR6	EXOC3_MOUSE	0.00000	0.00000	0.00000	0.00000	0.00000	0.00000	0.00000	0.00038	0.00107	0.00000	0.00000	0.00000
Q6NSQ9	G6PC3_MOUSE	0.00000	0.00000	0.00000	0.00000	0.00000	0.00000	0.00103	0.01117	0.00050	0.00248	0.00380	0.00380
Q6NSR8	PEPL1_MOUSE	0.00055	0.00420	0.00125	0.00065	0.00016	0.00000	0.00000	0.01148	0.00000	0.00322	0.00250	0.00149
Q6NV83	SR140_MOUSE	0.00000	0.00000	0.00000	0.00000	0.00000	0.00000	0.00172	0.00456	0.00403	0.00227	0.00515	0.00569
Q6NVE8	WDR44_MOUSE	0.00000	0.00000	0.00000	0.00000	0.00000	0.00000	0.00264	0.00090	0.00411	0.00165	0.00438	0.00254
Q6NVF9	CPSF6_MOUSE	0.00000	0.00000	0.00000	0.00000	0.00000	0.00000	0.00740	0.00608	0.00000	0.00791	0.00698	0.00697
Q6NY15	TSG10_MOUSE	0.00000	0.00000	0.00000	0.00000	0.00000	0.00000	0.00000	0.00428	0.00000	0.00183	0.00000	0.00156
Q6NZB0	DNJC8_MOUSE	0.00000	0.00000	0.00000	0.00000	0.00000	0.00000	0.00000	0.00469	0.00152	0.00105	0.00057	0.00044
Q6NZC7	S23IP_MOUSE	0.00000	0.00000	0.00000	0.00000	0.00000	0.00000	0.00000	0.00000	0.00000	0.00068	0.00000	0.00070
Q6NZJ6	IF4G1_MOUSE	0.00188	0.00103	0.00156	0.00214	0.00317	0.00190	0.01599	0.01039	0.01650	0.01487	0.01683	0.01461
Q6P069	SORCN_MOUSE	0.00000	0.00000	0.00000	0.00000	0.00000	0.00000	0.02074	0.02318	0.02658	0.02276	0.02898	0.01970
Q6P1B1	XPP1_MOUSE	0.00434	0.00353	0.00316	0.00297	0.00485	0.00315	0.01042	0.00707	0.00820	0.00887	0.00896	0.00925
Q6P1F6	2ABA_MOUSE	0.00132	0.00152	0.00183	0.00389	0.00228	0.00742	0.01001	0.01034	0.00932	0.00980	0.00963	0.00872
Q6P3A8	ODBB_MOUSE	0.00657	0.00275	0.01102	0.00298	0.00378	0.03162	0.00000	0.00000	0.00016	0.00000	0.00023	0.00017
Q6P3D0	NUD16_MOUSE	0.00000	0.00000	0.00000	0.00000	0.00000	0.00000	0.00062	0.00221	0.00000	0.00075	0.00000	0.00000
Q6P4T2	U520_MOUSE	0.00000	0.00000	0.00000	0.00000	0.00000	0.00000	0.02079	0.01868	0.01860	0.01768	0.01910	0.02293
Q6P542	ABCF1_MOUSE	0.00150	0.00150	0.00089	0.00128	0.00107	0.00168	0.07153	0.05478	0.09893	0.06456	0.13372	0.06542
Q6P5D4	CP135_MOUSE	0.00000	0.00000	0.00000	0.00000	0.00000	0.00000	0.00000	0.00267	0.00134	0.00142	0.00273	0.00074
Q6P5E4	UGGG1_MOUSE	0.00000	0.00000	0.00000	0.00000	0.00000	0.00000	0.02174	0.02434	0.02273	0.02446	0.02286	0.02296
Q6P5F9	XPO1_MOUSE	0.01396	0.00147	0.01053	0.00955	0.00990	0.00790	0.00787	0.01249	0.01656	0.01484	0.01290	0.00970
Q6P5H2	NEST_MOUSE	0.00000	0.00000	0.00000	0.00000	0.00000	0.00000	0.00545	0.01155	0.01088	0.00861	0.00381	0.00985
Q6P6M5	PX11C_MOUSE	0.00000	0.00000	0.00000	0.00000	0.00000	0.00000	0.00000	0.00000	0.00000	0.00000	0.00000	0.00409
Q6P8I4	PCNP_MOUSE	0.00000	0.00000	0.00000	0.00000	0.00000	0.00000	0.00000	0.00116	0.00641	0.00464	0.00320	0.00156
Q6P8J2	SAT2_MOUSE	0.00000	0.00000	0.00000	0.00000	0.00000	0.00000	0.00000	0.00000	0.00000	0.00000	0.00000	0.00000
Q6P8J7	KCRS_MOUSE	1.48756	1.48302	1.39633	1.42533	1.42860	1.45937	0.05127	0.05535	0.03288	0.05818	0.03229	0.03217
Q6P8X1	SNX6_MOUSE	0.00000	0.00000	0.00000	0.00000	0.00000	0.00000	0.01702	0.01468	0.02089	0.01935	0.01918	0.01521
Q6P9J5	KANK4_MOUSE	0.00000	0.00000	0.00000	0.00000	0.00000	0.00000	0.00592	0.00540	0.00807	0.00617	0.00944	0.00770
Q6P9J9	ANO6_MOUSE	0.00000	0.00000	0.00000	0.00000	0.00000	0.00000	0.00533	0.00640	0.00937	0.00496	0.00700	0.00701

Q6P9Q4	FHOD1_MOUSE	0.00000	0.00000	0.00000	0.00000	0.00000	0.00000	0.01574	0.01869	0.01785	0.01680	0.01384	0.01575
Q6P9Q6	FKB15_MOUSE	0.00000	0.00000	0.00000	0.00000	0.00000	0.00000	0.00449	0.00586	0.00560	0.00605	0.00562	0.00735
Q6P9R2	OXSR1_MOUSE	0.00000	0.00000	0.00000	0.00000	0.00000	0.00000	0.01296	0.01144	0.01189	0.01242	0.01149	0.01062
Q6P9R4	ARHGI_MOUSE	0.00000	0.00000	0.00000	0.00000	0.00000	0.00000	0.00212	0.00526	0.00000	0.00000	0.00303	0.00000
Q6PA06	ATLA2_MOUSE	0.00000	0.00000	0.00000	0.00000	0.00000	0.00000	0.00175	0.00105	0.00124	0.00203	0.00086	0.00154
Q6PAM1	TXLNA_MOUSE	0.00000	0.00000	0.00000	0.00000	0.00000	0.00000	0.00000	0.00156	0.00337	0.00290	0.00138	0.00092
Q6PAR5	GAPD1_MOUSE	0.00000	0.00000	0.00000	0.00000	0.00000	0.00000	0.00000	0.00420	0.00185	0.00000	0.00307	0.00128
Q6PAV2	HERC4_MOUSE	0.00000	0.00000	0.00000	0.00000	0.00000	0.00000	0.00000	0.00024	0.00244	0.00087	0.00145	0.00135
Q6PB44	PTN23_MOUSE	0.00000	0.00000	0.00000	0.00000	0.00000	0.00000	0.00122	0.00322	0.00264	0.00248	0.00137	0.00357
Q6PB66	LPPRC_MOUSE	0.06967	0.12371	0.07715	0.07365	0.08173	0.08286	0.01005	0.00605	0.00676	0.00674	0.00793	0.00696
Q6PB93	GALT2_MOUSE	0.00000	0.00000	0.00000	0.00000	0.00000	0.00000	0.00579	0.00431	0.00591	0.00466	0.00692	0.00524
Q6PCP5	MFF_MOUSE	0.00000	0.00000	0.00000	0.00000	0.00000	0.00000	0.00226	0.00586	0.00718	0.00661	0.00898	0.00712
Q6PD03	2A5A_MOUSE	0.00345	0.00728	0.00405	0.00682	0.00440	0.00626	0.00842	0.00692	0.00468	0.00513	0.00169	0.00786
Q6PDG5	SMRC2_MOUSE	0.00000	0.00000	0.00000	0.00000	0.00000	0.00000	0.00355	0.00930	0.00597	0.00793	0.00510	0.00884
Q6PDH0	PHLB1_MOUSE	0.00000	0.00000	0.00000	0.00000	0.00000	0.00000	0.00663	0.00555	0.00467	0.00198	0.00667	0.00567
Q6PDI5	ECM29_MOUSE	0.02205	0.03157	0.01208	0.01702	0.01512	0.03432	0.00000	0.00213	0.01435	0.00354	0.00000	0.00000
Q6PDI6	MINY2_MOUSE	0.00000	0.00000	0.00000	0.00000	0.00000	0.00000	0.00000	0.00000	0.00202	0.00092	0.00060	0.00054
Q6PDL0	DC1L2_MOUSE	0.00000	0.00000	0.00000	0.00000	0.00000	0.00000	0.01489	0.01526	0.01633	0.01446	0.01305	0.01242
Q6PDM2	SRSF1_MOUSE	0.00000	0.00000	0.00000	0.00000	0.00000	0.00000	0.01375	0.01981	0.02703	0.01957	0.02120	0.02627
Q6PDN3	MYLK_MOUSE	0.00000	0.00000	0.00000	0.00000	0.00000	0.00000	0.05483	0.04547	0.04618	0.04860	0.05075	0.05377
Q6PDQ2	CHD4_MOUSE	0.00000	0.00000	0.00000	0.00000	0.00000	0.00000	0.00658	0.01533	0.01167	0.01074	0.01555	0.01287
Q6PE01	SNR40_MOUSE	0.00000	0.00000	0.00000	0.00000	0.00000	0.00000	0.00168	0.00237	0.00332	0.00149	0.00340	0.00156
Q6PEE3	RIR2B_MOUSE	0.00000	0.00000	0.00000	0.00000	0.00000	0.00000	0.00000	0.00299	0.00000	0.00000	0.00000	0.00000
Q6PER3	MARE3_MOUSE	0.00157	0.00167	0.00141	0.00175	0.00264	0.00213	0.00518	0.00732	0.00839	0.00685	0.00547	0.00699
Q6PFD9	NUP98_MOUSE	0.00000	0.00000	0.00000	0.00000	0.00000	0.00000	0.00000	0.00315	0.00402	0.00455	0.00174	0.00573
Q6PFR5	TRA2A_MOUSE	0.00000	0.00000	0.00000	0.00000	0.00000	0.00000	0.00270	0.01197	0.00463	0.00743	0.00731	0.01086
Q6PGB6	NAA50_MOUSE	0.00000	0.00000	0.00000	0.00000	0.00000	0.00000	0.00000	0.00340	0.00157	0.00298	0.00132	0.00000
Q6PGF7	EXOC8_MOUSE	0.00000	0.00000	0.00000	0.00000	0.00000	0.00000	0.00000	0.00240	0.00342	0.00214	0.00084	0.00145
Q6PGH1	BUD31_MOUSE	0.00000	0.00000	0.00000	0.00000	0.00000	0.00000	0.00370	0.00206	0.00312	0.00254	0.00393	0.00346
Q6PGL7	WASC2_MOUSE	0.00000	0.00000	0.00000	0.00000	0.00000	0.00000	0.00575	0.00421	0.00628	0.00334	0.00562	0.00582
Q6PHN9	RAB35_MOUSE	0.00237	0.00107	0.00176	0.00243	0.00116	0.00154	0.01796	0.01828	0.02089	0.02016	0.01620	0.02133
Q6PHU5	SORT_MOUSE	0.00000	0.00000	0.00000	0.00000	0.00000	0.00000	0.00772	0.01311	0.01492	0.01080	0.01518	0.01522
Q6PHZ2	KCC2D_MOUSE	0.01285	0.01421	0.02409	0.01250	0.01405	0.01507	0.03145	0.02927	0.02792	0.02813	0.02692	0.03206
Q6PIE5	AT1A2_MOUSE	0.01252	0.01437	0.00638	0.00921	0.00815	0.01030	0.00940	0.00578	0.00633	0.00182	0.00753	0.00534

Q6PIP5	NUDC1_MOUSE	0.00000	0.00000	0.00000	0.00000	0.00000	0.00000	0.00000	0.00664	0.00451	0.00568	0.00000	0.00000
Q6PIU9	YJ005_MOUSE	0.00460	0.00183	0.00090	0.00066	0.00119	0.00075	0.01157	0.00875	0.00608	0.00773	0.00660	0.00926
Q6Q477	AT2B4_MOUSE	0.00350	0.00229	0.00313	0.00353	0.00329	0.00151	0.00461	0.00759	0.00887	0.00721	0.00193	0.00619
Q6Q899	DDX58_MOUSE	0.00000	0.00000	0.00000	0.00000	0.00000	0.00000	0.00869	0.00831	0.01027	0.00857	0.01144	0.00879
Q6R891	NEB2_MOUSE	0.00000	0.00000	0.00000	0.00000	0.00000	0.00000	0.00000	0.00035	0.00160	0.00000	0.00000	0.00180
Q6RHR9	MAGI1_MOUSE	0.00000	0.00000	0.00000	0.00000	0.00000	0.00000	0.00505	0.00175	0.00449	0.00276	0.00482	0.00492
Q6URW6	MYH14_MOUSE	0.01448	0.01425	0.00965	0.01790	0.00995	0.01132	0.38966	0.47341	0.43151	0.40908	0.40362	0.42654
Q6WKZ7	NOSTN_MOUSE	0.00000	0.00000	0.00000	0.00000	0.00000	0.00000	0.00158	0.00332	0.00232	0.00087	0.00000	0.00255
Q6WVG3	KCD12_MOUSE	0.00000	0.00000	0.00000	0.00000	0.00000	0.00000	0.02147	0.01607	0.02184	0.01861	0.01997	0.02678
Q6ZPF4	FMNL3_MOUSE	0.00000	0.00000	0.00000	0.00000	0.00000	0.00000	0.00511	0.00242	0.00439	0.00348	0.00430	0.00326
Q6ZPJ3	UBE2O_MOUSE	0.00000	0.00000	0.00000	0.00000	0.00000	0.00000	0.00000	0.00000	0.00083	0.00094	0.00259	0.00080
Q6ZPQ6	PITM2_MOUSE	0.00000	0.00000	0.00000	0.00000	0.00000	0.00000	0.00000	0.00090	0.00064	0.00153	0.00092	0.00083
Q6ZQ08	CNOT1_MOUSE	0.00000	0.00000	0.00000	0.00000	0.00000	0.00000	0.02380	0.02885	0.02387	0.02876	0.02352	0.02557
Q6ZQ38	CAND1_MOUSE	0.00178	0.00097	0.00282	0.00193	0.00153	0.00111	0.02675	0.01835	0.02995	0.02401	0.03508	0.02736
Q6ZQ82	RHG26_MOUSE	0.00000	0.00000	0.00000	0.00000	0.00000	0.00000	0.00000	0.00174	0.00092	0.00261	0.00544	0.00135
Q6ZQI3	MLEC_MOUSE	0.00000	0.00000	0.00000	0.00000	0.00000	0.00000	0.00650	0.01396	0.01018	0.01323	0.00993	0.01232
Q6ZQK5	ACAP2_MOUSE	0.00000	0.00000	0.00000	0.00000	0.00000	0.00000	0.01115	0.01129	0.00834	0.00801	0.00734	0.01154
Q6ZQM8	UD17C_MOUSE	0.00000	0.00000	0.00000	0.00000	0.00000	0.00000	0.01054	0.01849	0.01070	0.01290	0.01109	0.01092
Q6ZWM4	LSM8_MOUSE	0.00000	0.00000	0.00000	0.00000	0.00000	0.00000	0.00386	0.00341	0.00250	0.00419	0.00483	0.00233
Q6ZWN5	RS9_MOUSE	0.00000	0.00000	0.00000	0.00000	0.00000	0.00000	0.01446	0.03074	0.02164	0.02294	0.01334	0.02147
Q6ZWQ0	SYNE2_MOUSE	0.00000	0.00000	0.00000	0.00000	0.00000	0.00000	0.00364	0.00373	0.00642	0.00381	0.00604	0.00518
Q6ZWQ7	SPCS3_MOUSE	0.00000	0.00000	0.00000	0.00000	0.00000	0.00000	0.00582	0.00833	0.00404	0.00474	0.00255	0.00659
Q6ZWR6	SYNE1_MOUSE	0.00000	0.00000	0.00000	0.00000	0.00000	0.00000	0.01655	0.03481	0.04093	0.03634	0.05728	0.04106
Q6ZWU9	RS27_MOUSE	0.00000	0.00000	0.00000	0.00000	0.00000	0.00000	0.16489	0.04089	0.03796	0.04362	0.18993	0.03509
Q6ZWV3	RL10_MOUSE	0.00000	0.00000	0.00000	0.00000	0.00000	0.00000	0.02258	0.01557	0.01323	0.01143	0.00961	0.01374
Q6ZWV7	RL35_MOUSE	0.00000	0.00000	0.00000	0.00000	0.00000	0.00000	0.00000	0.00706	0.00809	0.00765	0.00247	0.00379
Q6ZWX6	IF2A_MOUSE	0.00000	0.00000	0.00000	0.00000	0.00000	0.00000	0.00860	0.01297	0.02419	0.01998	0.02401	0.01628
Q6ZWY3	RS27L_MOUSE	0.00000	0.00000	0.00000	0.00000	0.00000	0.00000	0.01292	0.01109	0.01462	0.01229	0.01121	0.01344
Q70IV5	SYNEM_MOUSE	0.00000	0.00000	0.00000	0.00000	0.00000	0.00000	0.01583	0.01441	0.02127	0.01640	0.01810	0.01837
Q70KF4	CMYA5_MOUSE	0.01074	0.01113	0.00886	0.00869	0.00672	0.00717	0.00852	0.02227	0.03425	0.02615	0.02431	0.02948
Q71LX4	TLN2_MOUSE	0.08249	0.07519	0.07704	0.06482	0.06811	0.06091	0.02103	0.01877	0.01956	0.01742	0.02135	0.01941
Q71RI9	KAT3_MOUSE	0.01828	0.01917	0.02302	0.01111	0.01787	0.01643	0.00860	0.00802	0.00107	0.00296	0.00250	0.00443
Q75N62	GIMA8_MOUSE	0.00000	0.00000	0.00000	0.00000	0.00000	0.00000	0.00000	0.00000	0.00085	0.00000	0.00040	0.00034
Q76LS9	MINY1_MOUSE	0.00000	0.00000	0.00000	0.00000	0.00000	0.00000	0.00000	0.00160	0.00665	0.00410	0.00439	0.00204

Q76MZ3	2AAA_MOUSE	0.00499	0.00231	0.02644	0.00452	0.00932	0.00617	0.04847	0.04111	0.06514	0.05676	0.07265	0.05087
Q78HU7	GLPC_MOUSE	0.00000	0.00000	0.00000	0.00000	0.00000	0.00000	0.01062	0.00000	0.00241	0.00730	0.01336	0.00420
Q78IK2	ATPMD_MOUSE	0.00000	0.00000	0.00000	0.00000	0.00000	0.00000	0.04129	0.04633	0.03463	0.04854	0.02977	0.02244
Q78IK4	MIC27_MOUSE	0.01990	0.02467	0.01576	0.04948	0.01895	0.04284	0.00000	0.00604	0.00479	0.00558	0.00233	0.00572
Q78IQ7	S39A4_MOUSE	0.00000	0.00000	0.00000	0.00000	0.00000	0.00000	0.00904	0.00764	0.00685	0.00758	0.00321	0.00924
Q78IS1	TMED3_MOUSE	0.00000	0.00000	0.00000	0.00000	0.00000	0.00000	0.01134	0.00936	0.01440	0.01119	0.01311	0.00987
Q78JE5	FBX22_MOUSE	0.00000	0.00000	0.00000	0.00000	0.00000	0.00000	0.00078	0.00411	0.00183	0.00503	0.00224	0.00513
Q78JN3	ECI3_MOUSE	0.00000	0.00000	0.00000	0.00000	0.00000	0.00000	0.00000	0.00000	0.00000	0.00000	0.00000	0.00000
Q78JT3	3HAO_MOUSE	0.00000	0.00000	0.00000	0.00000	0.00000	0.00000	0.00000	0.00007	0.00006	0.00003	0.00008	0.00016
Q78KK3	S22AI_MOUSE	0.00000	0.00000	0.00000	0.00000	0.00000	0.00000	0.00000	0.00000	0.00000	0.00000	0.00000	0.00000
Q78PY7	SND1_MOUSE	0.00089	0.00310	0.00107	0.00109	0.00184	0.00209	0.02798	0.04804	0.06150	0.05302	0.05376	0.04736
Q78YZ6	SCOC_MOUSE	0.00000	0.00000	0.00000	0.00000	0.00000	0.00000	0.00047	0.00000	0.00226	0.00217	0.00287	0.00104
Q78ZA7	NP1L4_MOUSE	0.00742	0.00657	0.00473	0.00241	0.00439	0.00262	0.02989	0.04359	0.04359	0.04469	0.04325	0.04393
Q791T5	MTCH1_MOUSE	0.00000	0.00000	0.00000	0.00000	0.00000	0.00000	0.00000	0.00360	0.00000	0.00180	0.00162	0.00438
Q791V5	MTCH2_MOUSE	0.02429	0.03419	0.03544	0.04302	0.03469	0.05535	0.01087	0.01464	0.01114	0.01318	0.02225	0.01205
Q7M6Y3	PICAL_MOUSE	0.00000	0.00000	0.00000	0.00000	0.00000	0.00000	0.02709	0.01137	0.02208	0.01811	0.02985	0.01417
Q7M6Z4	KIF27_MOUSE	0.00000	0.00000	0.00000	0.00000	0.00000	0.00000	0.10118	0.00460	0.02148	0.05211	0.12681	0.00423
Q7M757	BRC3L_MOUSE	0.00000	0.00000	0.00000	0.00000	0.00000	0.00000	0.00153	0.01370	0.00811	0.01081	0.00471	0.00602
Q7SIG6	ASAP2_MOUSE	0.00000	0.00000	0.00000	0.00000	0.00000	0.00000	0.00064	0.00195	0.00166	0.00208	0.00057	0.00000
Q7TMB8	CYFP1_MOUSE	0.00000	0.00000	0.00000	0.00000	0.00000	0.00000	0.01673	0.02042	0.01453	0.01343	0.01610	0.01676
Q7TMF3	NDUAC_MOUSE	0.14034	0.10020	0.10182	0.08248	0.10343	0.07312	0.00664	0.00341	0.00374	0.00223	0.00322	0.00241
Q7TMK9	HNRPQ_MOUSE	0.00000	0.00000	0.00000	0.00000	0.00000	0.00000	0.02619	0.02138	0.02736	0.02438	0.03413	0.02831
Q7TMM9	TBB2A_MOUSE	0.00000	0.00000	0.00000	0.00000	0.00000	0.00000	0.07178	0.08224	0.08436	0.08058	0.06723	0.10309
Q7TMR0	PCP_MOUSE	0.00000	0.00000	0.00000	0.00000	0.00000	0.00000	0.00287	0.00000	0.00056	0.00071	0.00000	0.00383
Q7TMS5	ABCG2_MOUSE	0.00000	0.00000	0.00000	0.00000	0.00000	0.00000	0.00000	0.00000	0.00027	0.00020	0.00000	0.00012
Q7TMY8	HUWE1_MOUSE	0.00218	0.00150	0.00231	0.00164	0.00080	0.00257	0.00644	0.00510	0.00669	0.00673	0.00754	0.01019
Q7TN29	SMAP2_MOUSE	0.00000	0.00000	0.00000	0.00000	0.00000	0.00000	0.00000	0.00000	0.00000	0.00000	0.00000	0.00000
Q7TNC4	LC7L2_MOUSE	0.00000	0.00000	0.00000	0.00000	0.00000	0.00000	0.01529	0.01022	0.01598	0.01382	0.01181	0.01230
Q7TNE1	SUCHY_MOUSE	0.00000	0.00000	0.00000	0.00000	0.00000	0.00000	0.00000	0.00064	0.00000	0.00000	0.00000	0.00000
Q7TNG5	EMAL2_MOUSE	0.00716	0.00531	0.00259	0.00456	0.00421	0.01073	0.03740	0.02282	0.02305	0.01653	0.01061	0.01235
Q7TNG8	LDHD_MOUSE	0.00000	0.00000	0.00000	0.00000	0.00000	0.00000	0.00020	0.00000	0.00000	0.00004	0.00000	0.00004
Q7TNP2	2AAB_MOUSE	0.00000	0.00000	0.00000	0.00000	0.00000	0.00000	0.00000	0.00000	0.00000	0.00000	0.00000	0.00000
Q7TNV0	DEK_MOUSE	0.00000	0.00000	0.00000	0.00000	0.00000	0.00000	0.02402	0.05295	0.01670	0.02479	0.01199	0.02686
Q7TPD0	INT3_MOUSE	0.00000	0.00000	0.00000	0.00000	0.00000	0.00000	0.00294	0.00054	0.00396	0.00266	0.00432	0.00343

Q7TPR4	ACTN1_MOUSE	0.04380	0.03212	0.02462	0.02449	0.03542	0.03820	0.09309	0.07767	0.09460	0.08811	0.08476	0.08577
Q7TPV4	MBB1A_MOUSE	0.00000	0.00000	0.00000	0.00000	0.00000	0.00000	0.00356	0.00377	0.00659	0.00383	0.00414	0.00446
Q7TPW1	NEXN_MOUSE	0.00507	0.00171	0.00237	0.00307	0.00538	0.00426	0.00000	0.00183	0.00343	0.00431	0.00247	0.00306
Q7TQ48	SRCA_MOUSE	0.62070	0.51275	0.76808	0.44009	0.74757	0.63688	0.04417	0.01858	0.02095	0.02390	0.02038	0.01706
Q7TQ95	LNP_MOUSE	0.00000	0.00000	0.00000	0.00000	0.00000	0.00000	0.00133	0.00267	0.00296	0.00314	0.00166	0.00313
Q7TQF7	AMPH_MOUSE	0.00000	0.00000	0.00000	0.00000	0.00000	0.00000	0.00290	0.00522	0.00354	0.00431	0.00568	0.00493
Q7TQH0	ATX2L_MOUSE	0.00000	0.00000	0.00000	0.00000	0.00000	0.00000	0.00000	0.00000	0.00296	0.00577	0.00311	0.00112
Q7TQI3	OTUB1_MOUSE	0.00914	0.01002	0.00462	0.00530	0.00405	0.00726	0.01940	0.02193	0.02801	0.02622	0.01749	0.02507
Q7TR96	O1013_MOUSE	0.00000	0.00000	0.00000	0.00000	0.00000	0.00000	0.02080	0.10595	0.12678	0.13325	0.08610	0.04958
Q7TSE6	ST38L_MOUSE	0.00000	0.00000	0.00000	0.00000	0.00000	0.00000	0.00112	0.00127	0.00269	0.00171	0.00232	0.00186
Q7TSI3	PP6R1_MOUSE	0.00000	0.00000	0.00000	0.00000	0.00000	0.00000	0.00000	0.00149	0.00289	0.00242	0.00203	0.00162
Q7TSJ2	MAP6_MOUSE	0.00000	0.00000	0.00000	0.00000	0.00000	0.00000	0.00000	0.00249	0.00206	0.00278	0.00199	0.00086
Q7TSQ8	PDPR_MOUSE	0.00043	0.00059	0.00125	0.00027	0.00050	0.00027	0.00192	0.00448	0.00440	0.00280	0.00438	0.00259
Q7TST5	LAMP3_MOUSE	0.00000	0.00000	0.00000	0.00000	0.00000	0.00000	0.00000	0.00399	0.00251	0.00000	0.00114	0.00178
Q7TSV4	PGM2_MOUSE	0.00000	0.00000	0.00000	0.00000	0.00000	0.00000	0.01255	0.02532	0.02781	0.02353	0.01508	0.02195
Q7TT37	ELP1_MOUSE	0.00000	0.00000	0.00000	0.00000	0.00000	0.00000	0.00515	0.00838	0.00507	0.00948	0.00558	0.01040
Q7TT50	MRCKB_MOUSE	0.00000	0.00000	0.00000	0.00000	0.00000	0.00000	0.00571	0.00459	0.00740	0.00629	0.00771	0.00619
Q80SU6	NPT2C_MOUSE	0.00000	0.00000	0.00000	0.00000	0.00000	0.00000	0.00000	0.00000	0.00000	0.00000	0.00000	0.00000
Q80SU7	GVIN1_MOUSE	0.00000	0.00000	0.00000	0.00000	0.00000	0.00000	0.00486	0.00305	0.00650	0.00516	0.00310	0.00372
Q80SW1	SAHH2_MOUSE	0.00180	0.00187	0.00199	0.00108	0.00175	0.00181	0.00000	0.00234	0.00000	0.00000	0.00000	0.00142
Q80SY3	VA0D2_MOUSE	0.00000	0.00000	0.00000	0.00000	0.00000	0.00000	0.00000	0.00000	0.00000	0.00000	0.00000	0.00000
Q80SZ7	GBG5_MOUSE	0.00000	0.00000	0.00000	0.00000	0.00000	0.00000	0.00000	0.00789	0.00605	0.00758	0.00290	0.00373
Q80TA6	MTMRC_MOUSE	0.00000	0.00000	0.00000	0.00000	0.00000	0.00000	0.00000	0.00349	0.00000	0.00000	0.00192	0.00000
Q80TH2	ERBIN_MOUSE	0.00205	0.00526	0.00265	0.00269	0.00230	0.00173	0.00686	0.00908	0.01052	0.01003	0.01067	0.01259
Q80TL7	MON2_MOUSE	0.00000	0.00000	0.00000	0.00000	0.00000	0.00000	0.00000	0.00155	0.00244	0.00276	0.00000	0.00143
Q80TV8	CLAP1_MOUSE	0.00000	0.00000	0.00000	0.00000	0.00000	0.00000	0.00239	0.00111	0.00393	0.00210	0.00352	0.00364
Q80TY0	FNBP1_MOUSE	0.00000	0.00000	0.00000	0.00000	0.00000	0.00000	0.00515	0.00631	0.00634	0.00611	0.00484	0.00688
Q80U63	MFN2_MOUSE	0.00375	0.00346	0.00226	0.00175	0.00170	0.00270	0.00336	0.00042	0.00286	0.00242	0.00196	0.00226
Q80U93	NU214_MOUSE	0.00000	0.00000	0.00000	0.00000	0.00000	0.00000	0.00524	0.00444	0.00375	0.00360	0.00156	0.00369
Q80UG5	SEPT9_MOUSE	0.00000	0.00000	0.00000	0.00000	0.00000	0.00000	0.01185	0.02043	0.02500	0.01462	0.01037	0.01277
Q80UJ7	RB3GP_MOUSE	0.00000	0.00000	0.00000	0.00000	0.00000	0.00000	0.00000	0.00000	0.00091	0.00051	0.00054	0.00000
Q80UM7	MOGS_MOUSE	0.00000	0.00000	0.00000	0.00000	0.00000	0.00000	0.00531	0.00659	0.00527	0.00438	0.00368	0.00641
Q80UP5	AN13A_MOUSE	0.00000	0.00000	0.00000	0.00000	0.00000	0.00000	0.00187	0.00537	0.00763	0.00770	0.00594	0.00448
Q80UU9	PGRC2_MOUSE	0.00000	0.00000	0.00000	0.00000	0.00000	0.00000	0.00000	0.00528	0.01212	0.00601	0.00436	0.00590

Q80UW5	MRCKG_MOUSE	0.00000	0.00000	0.00000	0.00000	0.00000	0.00000	0.00638	0.00856	0.00711	0.00763	0.00670	0.00888
Q80UW8	RPAB1_MOUSE	0.00000	0.00000	0.00000	0.00000	0.00000	0.00000	0.00000	0.00000	0.00000	0.00000	0.00093	0.00085
Q80V42	CBPM_MOUSE	0.00000	0.00000	0.00000	0.00000	0.00000	0.00000	0.00744	0.00954	0.00696	0.00697	0.00758	0.00666
Q80V70	MEGF6_MOUSE	0.00000	0.00000	0.00000	0.00000	0.00000	0.00000	0.00000	0.00229	0.00493	0.00246	0.00369	0.00100
Q80VD1	FA98B_MOUSE	0.00000	0.00000	0.00000	0.00000	0.00000	0.00000	0.00246	0.00283	0.00351	0.00291	0.00137	0.00338
Q80VJ2	SRA1_MOUSE	0.00000	0.00000	0.00000	0.00000	0.00000	0.00000	0.00000	0.00000	0.00050	0.00116	0.00000	0.00000
Q80VJ3	DNPH1_MOUSE	0.00000	0.00000	0.00000	0.00000	0.00000	0.00000	0.00000	0.00000	0.00000	0.00000	0.00000	0.00330
Q80VP0	TCPR1_MOUSE	0.00000	0.00000	0.00000	0.00000	0.00000	0.00000	0.00318	0.00474	0.00472	0.00330	0.00623	0.00480
Q80VP1	EPN1_MOUSE	0.00000	0.00000	0.00000	0.00000	0.00000	0.00000	0.00419	0.00583	0.00787	0.00406	0.00985	0.00626
Q80VQ0	AL3B1_MOUSE	0.00000	0.00000	0.00000	0.00000	0.00000	0.00000	0.00883	0.01624	0.01738	0.02133	0.01246	0.02084
Q80VQ1	LRRC1_MOUSE	0.00000	0.00000	0.00000	0.00000	0.00000	0.00000	0.00395	0.00166	0.00508	0.00215	0.00555	0.00539
Q80W04	TMCC2_MOUSE	0.00000	0.00000	0.00000	0.00000	0.00000	0.00000	0.00135	0.00393	0.00394	0.00288	0.00504	0.00300
Q80W21	GSTM7_MOUSE	0.01304	0.01390	0.00948	0.01966	0.01460	0.03306	0.00236	0.00632	0.00184	0.00481	0.00000	0.00420
Q80W22	THNS2_MOUSE	0.00000	0.00000	0.00000	0.00000	0.00000	0.00000	0.00045	0.00229	0.00159	0.00088	0.00196	0.00156
Q80W54	FACE1_MOUSE	0.00384	0.00287	0.00330	0.00328	0.00204	0.00415	0.01583	0.01441	0.01937	0.01549	0.01824	0.01478
Q80W93	HYDIN_MOUSE	0.00055	0.00108	0.00088	0.00145	0.00084	0.00111	0.00000	0.01033	0.00609	0.01293	0.00645	0.00611
Q80WB5	NTAQ1_MOUSE	0.00000	0.00000	0.00000	0.00000	0.00000	0.00000	0.00000	0.00072	0.00516	0.00197	0.00217	0.00177
Q80WC7	AGFG2_MOUSE	0.00000	0.00000	0.00000	0.00000	0.00000	0.00000	0.00081	0.00825	0.00310	0.00218	0.00306	0.00037
Q80WG5	LRC8A_MOUSE	0.00000	0.00000	0.00000	0.00000	0.00000	0.00000	0.00000	0.00000	0.00054	0.00000	0.00039	0.00024
Q80WJ7	LYRIC_MOUSE	0.00000	0.00000	0.00000	0.00000	0.00000	0.00000	0.01143	0.01229	0.00624	0.00960	0.00782	0.00952
Q80WK2	OSTB_MOUSE	0.00000	0.00000	0.00000	0.00000	0.00000	0.00000	0.00000	0.00000	0.00000	0.00000	0.00000	0.00000
Q80WQ2	VAC14_MOUSE	0.00000	0.00000	0.00000	0.00000	0.00000	0.00000	0.07665	0.01173	0.02051	0.01138	0.02377	0.01476
Q80WR1	TSN18_MOUSE	0.00000	0.00000	0.00000	0.00000	0.00000	0.00000	0.00203	0.00602	0.00180	0.00272	0.00095	0.00260
Q80WW9	DDRGK_MOUSE	0.00000	0.00000	0.00000	0.00000	0.00000	0.00000	0.00000	0.00190	0.00472	0.00333	0.00264	0.00511
Q80X19	COEA1_MOUSE	0.00000	0.00000	0.00000	0.00000	0.00000	0.00000	0.00585	0.00337	0.00874	0.00215	0.01392	0.00864
Q80X41	VRK1_MOUSE	0.00000	0.00000	0.00000	0.00000	0.00000	0.00000	0.00377	0.00472	0.00436	0.00239	0.01338	0.00419
Q80X50	UBP2L_MOUSE	0.00000	0.00000	0.00000	0.00000	0.00000	0.00000	0.00984	0.00857	0.00960	0.00833	0.00869	0.00846
Q80X85	RT07_MOUSE	0.00000	0.00000	0.00000	0.00000	0.00000	0.00000	0.00000	0.00022	0.00000	0.00000	0.00000	0.00000
Q80X90	FLNB_MOUSE	0.03543	0.01861	0.00983	0.01228	0.01067	0.01677	0.03086	0.02715	0.03235	0.02887	0.03174	0.03075
Q80X95	RRAGA_MOUSE	0.00000	0.00000	0.00000	0.00000	0.00000	0.00000	0.00000	0.00308	0.00513	0.00392	0.00207	0.00151
Q80XA6	REPS2_MOUSE	0.00000	0.00000	0.00000	0.00000	0.00000	0.00000	0.00000	0.00000	0.00129	0.00000	0.00000	0.00000
Q80XI4	PI42B_MOUSE	0.00080	0.00067	0.00077	0.00049	0.00049	0.00066	0.00477	0.00435	0.00746	0.00676	0.00621	0.00750
Q80XL6	ACD11_MOUSE	0.00000	0.00000	0.00000	0.00000	0.00000	0.00000	0.00000	0.00000	0.00002	0.00000	0.00000	0.00000
Q80XN0	BDH_MOUSE	0.09843	0.12299	0.14871	0.08946	0.14379	0.08936	0.00040	0.00099	0.00000	0.00017	0.00009	0.00016

Q80XR2	AT2C1_MOUSE	0.00000	0.00000	0.00000	0.00000	0.00000	0.00000	0.01668	0.07213	0.03590	0.05605	0.00000	0.06797
Q80XU3	NUCKS_MOUSE	0.00000	0.00000	0.00000	0.00000	0.00000	0.00000	0.00000	0.00610	0.00614	0.00665	0.00672	0.00661
Q80Y14	GLRX5_MOUSE	0.01216	0.01296	0.00915	0.00933	0.00721	0.01000	0.00624	0.00452	0.00420	0.00365	0.00210	0.00612
Q80YA7	DPP8_MOUSE	0.00000	0.00000	0.00000	0.00000	0.00000	0.00000	0.00659	0.01436	0.00876	0.00506	0.00000	0.00861
Q80YC5	FA12_MOUSE	0.00000	0.00000	0.00000	0.00000	0.00000	0.00000	0.00499	0.00361	0.00540	0.00589	0.00788	0.00309
Q80YV4	PANK4_MOUSE	0.00342	0.00522	0.00205	0.01497	0.00305	0.00247	0.00000	0.00000	0.00000	0.00000	0.00000	0.00000
Q80ZJ1	RAP2A_MOUSE	0.00000	0.00000	0.00000	0.00000	0.00000	0.00000	0.00000	0.01709	0.01050	0.01211	0.01495	0.01477
Q810A7	DDX42_MOUSE	0.00000	0.00000	0.00000	0.00000	0.00000	0.00000	0.00000	0.00000	0.00000	0.00062	0.00171	0.00080
Q810B6	ANFY1_MOUSE	0.00000	0.00000	0.00000	0.00000	0.00000	0.00000	0.00162	0.00370	0.00551	0.00518	0.00632	0.00448
Q811D0	DLG1_MOUSE	0.00116	0.00096	0.00087	0.00083	0.00094	0.00040	0.00186	0.00487	0.00804	0.00524	0.00768	0.00528
Q811I0	ATPF1_MOUSE	0.00250	0.00329	0.00362	0.00293	0.00218	0.00317	0.00000	0.00020	0.00029	0.00000	0.00032	0.00000
Q8BFP9	PDK1_MOUSE	0.01863	0.02029	0.01459	0.01483	0.01462	0.01105	0.00178	0.00511	0.00090	0.00085	0.00000	0.00292
Q8BFQ4	WDR82_MOUSE	0.00000	0.00000	0.00000	0.00000	0.00000	0.00000	0.00807	0.01172	0.01678	0.01047	0.01364	0.01637
Q8BFQ8	GALD1_MOUSE	0.00000	0.00000	0.00000	0.00000	0.00000	0.00000	0.00416	0.00247	0.00845	0.00571	0.00838	0.00722
Q8BFR4	GNS_MOUSE	0.00000	0.00000	0.00000	0.00000	0.00000	0.00000	0.00000	0.00804	0.00545	0.00239	0.00175	0.00000
Q8BFR5	EFTU_MOUSE	0.09974	0.17577	0.08721	0.13907	0.10424	0.22076	0.03212	0.02891	0.02633	0.03100	0.02845	0.03150
Q8BFS6	CPPED_MOUSE	0.00859	0.00611	0.00347	0.00318	0.00258	0.00280	0.00000	0.00358	0.00156	0.00195	0.00052	0.00037
Q8BFW7	LPP_MOUSE	0.00085	0.00166	0.00143	0.00189	0.00095	0.00228	0.04305	0.05378	0.04869	0.04953	0.03428	0.05503
Q8BFY6	PEF1_MOUSE	0.00082	0.00418	0.00177	0.00211	0.00053	0.00362	0.00353	0.00248	0.00710	0.00421	0.00572	0.00567
Q8BFY9	TNPO1_MOUSE	0.00000	0.00000	0.00000	0.00000	0.00000	0.00000	0.01042	0.00796	0.00909	0.00959	0.01106	0.01123
Q8BFZ9	ERLN2_MOUSE	0.00000	0.00000	0.00000	0.00000	0.00000	0.00000	0.00390	0.01332	0.01266	0.01422	0.00788	0.01138
Q8BG05	ROA3_MOUSE	0.00886	0.00800	0.00678	0.00991	0.00829	0.00727	0.19558	0.14625	0.16448	0.15643	0.17950	0.15295
Q8BG07	PLD4_MOUSE	0.00000	0.00000	0.00000	0.00000	0.00000	0.00000	0.00000	0.00138	0.00385	0.00269	0.00384	0.00252
Q8BG32	PSD11_MOUSE	0.00333	0.00634	0.00561	0.00610	0.00464	0.00463	0.01705	0.01287	0.01951	0.01719	0.01857	0.01718
Q8BG51	MIRO1_MOUSE	0.00000	0.00000	0.00000	0.00000	0.00000	0.00000	0.00521	0.00705	0.00302	0.00286	0.00351	0.00385
Q8BG67	EFR3A_MOUSE	0.00000	0.00000	0.00000	0.00000	0.00000	0.00000	0.00000	0.00000	0.00064	0.00051	0.00071	0.00000
Q8BG81	PDIP3_MOUSE	0.00000	0.00000	0.00000	0.00000	0.00000	0.00000	0.00400	0.00735	0.00224	0.00865	0.00000	0.00710
Q8BG95	MYPT2_MOUSE	0.00196	0.00100	0.00323	0.00381	0.00378	0.00198	0.00000	0.00523	0.00789	0.00773	0.00532	0.00265
Q8BGA8	ACSM5_MOUSE	0.00000	0.00000	0.00000	0.00000	0.00000	0.00000	0.00000	0.00000	0.00000	0.00000	0.00000	0.00000
Q8BGB5	LIMD2_MOUSE	0.00000	0.00000	0.00000	0.00000	0.00000	0.00000	0.00416	0.00942	0.01088	0.00736	0.01452	0.01066
Q8BGB7	ENOPH_MOUSE	0.00000	0.00000	0.00000	0.00000	0.00000	0.00000	0.02438	0.00835	0.03457	0.02704	0.03807	0.02467
Q8BGB8	COQ4_MOUSE	0.00000	0.00000	0.00000	0.00000	0.00000	0.00000	0.00000	0.00000	0.00000	0.00000	0.00000	0.00000
Q8BGC0	HTSF1_MOUSE	0.00000	0.00000	0.00000	0.00000	0.00000	0.00000	0.00000	0.00395	0.00643	0.00251	0.00891	0.00700
Q8BGC4	PTGR3_MOUSE	0.01973	0.02398	0.01377	0.01554	0.01612	0.02751	0.01038	0.01023	0.01149	0.00973	0.00911	0.01096

Q8BGD9	IF4B_MOUSE	0.00000	0.00000	0.00000	0.00000	0.00000	0.00000	0.01905	0.02389	0.02969	0.02914	0.03102	0.02768
Q8BGH2	SAM50_MOUSE	0.06798	0.06659	0.06807	0.06300	0.07432	0.06920	0.01447	0.01210	0.01366	0.01101	0.01229	0.01289
Q8BGH7	C42S2_MOUSE	0.00000	0.00000	0.00000	0.00000	0.00000	0.00000	0.00809	0.01020	0.01171	0.00855	0.01477	0.00706
Q8BGQ1	SPE39_MOUSE	0.00000	0.00000	0.00000	0.00000	0.00000	0.00000	0.00123	0.00055	0.00142	0.00065	0.00130	0.00074
Q8BGQ7	SYAC_MOUSE	0.00415	0.01281	0.00270	0.00427	0.00307	0.00316	0.00061	0.00591	0.00738	0.00691	0.00806	0.00956
Q8BGR6	ARL15_MOUSE	0.00000	0.00000	0.00000	0.00000	0.00000	0.00000	0.00345	0.00070	0.00550	0.00125	0.00519	0.00167
Q8BGR9	UBCP1_MOUSE	0.00000	0.00000	0.00000	0.00000	0.00000	0.00000	0.00275	0.00073	0.00036	0.00173	0.00138	0.00055
Q8BGS1	E41L5_MOUSE	0.00000	0.00000	0.00000	0.00000	0.00000	0.00000	0.02921	0.03174	0.03501	0.02966	0.03698	0.03412
Q8BGS7	CEPT1_MOUSE	0.00000	0.00000	0.00000	0.00000	0.00000	0.00000	0.00178	0.00462	0.00363	0.00248	0.00306	0.00656
Q8BGU5	CCNY_MOUSE	0.00000	0.00000	0.00000	0.00000	0.00000	0.00000	0.00136	0.00326	0.00539	0.00210	0.00537	0.00295
Q8BGX0	TRI23_MOUSE	0.00000	0.00000	0.00000	0.00000	0.00000	0.00000	0.00222	0.01840	0.00360	0.01344	0.01068	0.01309
Q8BGX2	TIM29_MOUSE	0.00000	0.00000	0.00000	0.00000	0.00000	0.00000	0.02159	0.00446	0.00000	0.00000	0.00000	0.00000
Q8BH00	AL8A1_MOUSE	0.00000	0.00000	0.00000	0.00000	0.00000	0.00000	0.00025	0.00032	0.00003	0.00027	0.00027	0.00011
Q8BH04	PCKGM_MOUSE	0.00000	0.00000	0.00000	0.00000	0.00000	0.00000	0.01035	0.01090	0.01398	0.01202	0.01401	0.01512
Q8BH24	TM9S4_MOUSE	0.00000	0.00000	0.00000	0.00000	0.00000	0.00000	0.00043	0.00171	0.00340	0.00402	0.00577	0.00316
Q8BH35	CO8B_MOUSE	0.00000	0.00000	0.00000	0.00000	0.00000	0.00000	0.00038	0.00066	0.00151	0.00000	0.00000	0.00000
Q8BH43	WASF2_MOUSE	0.00000	0.00000	0.00000	0.00000	0.00000	0.00000	0.06069	0.04104	0.00885	0.02074	0.01137	0.02431
Q8BH44	COR2B_MOUSE	0.00000	0.00000	0.00000	0.00000	0.00000	0.00000	0.00155	0.00124	0.00274	0.00156	0.00328	0.00209
Q8BH58	TIPRL_MOUSE	0.00316	0.00315	0.00373	0.00225	0.00431	0.00190	0.00216	0.00349	0.00658	0.00495	0.00589	0.00438
Q8BH59	CMC1_MOUSE	0.17879	0.15432	0.25799	0.19707	0.15313	0.13865	0.02798	0.01919	0.01282	0.01924	0.01523	0.01540
Q8BH61	F13A_MOUSE	0.00706	0.00686	0.00746	0.00780	0.00797	0.00778	0.01900	0.01363	0.01441	0.01522	0.01209	0.01636
Q8BH64	EHD2_MOUSE	0.10386	0.09238	0.10606	0.10042	0.11179	0.09134	0.21441	0.32130	0.34561	0.28896	0.34083	0.33428
Q8BH69	SPS1_MOUSE	0.00000	0.00000	0.00000	0.00000	0.00000	0.00000	0.00182	0.01454	0.00355	0.00471	0.00328	0.00696
Q8BH74	NU107_MOUSE	0.00000	0.00000	0.00000	0.00000	0.00000	0.00000	0.00000	0.00166	0.00318	0.00239	0.00099	0.00156
Q8BH79	ANO10_MOUSE	0.00000	0.00000	0.00000	0.00000	0.00000	0.00000	0.00316	0.00368	0.00415	0.00260	0.00225	0.00290
Q8BH86	GLUCM_MOUSE	0.03497	0.02444	0.02866	0.03399	0.02822	0.04529	0.00117	0.00139	0.00257	0.00211	0.00217	0.00166
Q8BH95	ECHM_MOUSE	0.08583	0.04425	0.06741	0.07146	0.06865	0.05734	0.07368	0.07478	0.07915	0.05873	0.05984	0.06446
Q8BH97	RCN3_MOUSE	0.00000	0.00000	0.00000	0.00000	0.00000	0.00000	0.01214	0.00802	0.01584	0.01122	0.01256	0.01075
Q8BHA3	DTD2_MOUSE	0.00000	0.00000	0.00000	0.00000	0.00000	0.00000	0.00000	0.00132	0.00200	0.00174	0.00090	0.00051
Q8BHC0	LYVE1_MOUSE	0.00000	0.00000	0.00000	0.00000	0.00000	0.00000	0.00000	0.01549	0.00468	0.00658	0.00000	0.00952
Q8BHC7	RHBL4_MOUSE	0.00000	0.00000	0.00000	0.00000	0.00000	0.00000	0.00075	0.00229	0.00000	0.00091	0.00088	0.00122
Q8BHD7	PTBP3_MOUSE	0.00000	0.00000	0.00000	0.00000	0.00000	0.00000	0.00758	0.00380	0.01803	0.01481	0.01798	0.02067
Q8BHF7	PGPS1_MOUSE	0.00000	0.00000	0.00000	0.00000	0.00000	0.00000	0.00212	0.00390	0.00518	0.00239	0.00191	0.00206
Q8BHG1	NRDC_MOUSE	0.00000	0.00000	0.00000	0.00000	0.00000	0.00000	0.00480	0.00214	0.00420	0.00309	0.00449	0.00443

Q8BHG2	CZIB_MOUSE	0.00000	0.00000	0.00000	0.00000	0.00000	0.00000	0.00392	0.00395	0.00580	0.00412	0.00516	0.00462
Q8BHI7	ELOV5_MOUSE	0.00000	0.00000	0.00000	0.00000	0.00000	0.00000	0.00842	0.00805	0.00600	0.00508	0.00439	0.00448
Q8BHJ5	TBL1R_MOUSE	0.00000	0.00000	0.00000	0.00000	0.00000	0.00000	0.00000	0.00055	0.00000	0.00020	0.00000	0.00043
Q8BHL4	RAI3_MOUSE	0.00000	0.00000	0.00000	0.00000	0.00000	0.00000	0.23638	0.22855	0.19219	0.17165	0.10268	0.17327
Q8BHL5	ELMO2_MOUSE	0.00000	0.00000	0.00000	0.00000	0.00000	0.00000	0.00086	0.00274	0.00653	0.00600	0.00287	0.00439
Q8BHL8	PSMF1_MOUSE	0.00000	0.00000	0.00000	0.00000	0.00000	0.00000	0.00000	0.00000	0.00165	0.00397	0.00356	0.00246
Q8BHN0	PPM1L_MOUSE	0.00000	0.00000	0.00000	0.00000	0.00000	0.00000	0.00000	0.00000	0.00127	0.00000	0.00125	0.00190
Q8BHN3	GANAB_MOUSE	0.00130	0.00158	0.00155	0.00252	0.00123	0.00158	0.02682	0.02919	0.02905	0.02836	0.02771	0.02537
Q8BHZ0	CYRIA_MOUSE	0.00000	0.00000	0.00000	0.00000	0.00000	0.00000	0.01067	0.01647	0.01276	0.01150	0.01320	0.01806
Q8BI08	MAL2_MOUSE	0.00000	0.00000	0.00000	0.00000	0.00000	0.00000	0.00184	0.01748	0.00578	0.00422	0.00302	0.01721
Q8BI29	SARG_MOUSE	0.00000	0.00000	0.00000	0.00000	0.00000	0.00000	0.01074	0.01434	0.01835	0.01295	0.02121	0.02076
Q8BIJ6	SYIM_MOUSE	0.05969	0.04481	0.06914	0.05600	0.06245	0.05474	0.03125	0.02937	0.03867	0.02969	0.03533	0.03638
Q8BIJ7	RUFY1_MOUSE	0.00000	0.00000	0.00000	0.00000	0.00000	0.00000	0.00000	0.00209	0.00484	0.00577	0.00117	0.00324
Q8BIK4	DOCK9_MOUSE	0.00000	0.00000	0.00000	0.00000	0.00000	0.00000	0.00849	0.00590	0.00836	0.00468	0.00708	0.00709
Q8BIW1	PRUN1_MOUSE	0.00000	0.00000	0.00000	0.00000	0.00000	0.00000	0.00079	0.00000	0.00033	0.00000	0.00000	0.00054
Q8BJ03	COX15_MOUSE	0.00000	0.00000	0.00000	0.00000	0.00000	0.00000	0.00000	0.00000	0.00000	0.00000	0.00000	0.00000
Q8BJ64	CHDH_MOUSE	0.00000	0.00000	0.00000	0.00000	0.00000	0.00000	0.00301	0.00157	0.00192	0.00225	0.00000	0.00338
Q8BJ71	NUP93_MOUSE	0.00000	0.00000	0.00000	0.00000	0.00000	0.00000	0.00102	0.00218	0.00478	0.00395	0.00754	0.00648
Q8BJS4	SUN2_MOUSE	0.00271	0.00200	0.00131	0.00186	0.00253	0.00237	0.01032	0.01020	0.01461	0.01125	0.01303	0.01437
Q8BJU0	SGTA_MOUSE	0.00093	0.00080	0.00111	0.00081	0.00081	0.00067	0.00282	0.00538	0.00712	0.00583	0.00392	0.00494
Q8BJU2	TSN9_MOUSE	0.00000	0.00000	0.00000	0.00000	0.00000	0.00000	0.01348	0.01514	0.01131	0.01072	0.00827	0.01655
Q8BJW6	EIF2A_MOUSE	0.00000	0.00000	0.00000	0.00000	0.00000	0.00000	0.00302	0.00100	0.00258	0.00185	0.00095	0.00259
Q8BJY1	PSMD5_MOUSE	0.00690	0.00943	0.00641	0.00286	0.00590	0.00783	0.01395	0.01272	0.02024	0.01757	0.02234	0.01771
Q8BJZ3	LFG3_MOUSE	0.00000	0.00000	0.00000	0.00000	0.00000	0.00000	0.00000	0.00374	0.00220	0.00300	0.00261	0.00171
Q8BK62	OLFL3_MOUSE	0.00000	0.00000	0.00000	0.00000	0.00000	0.00000	0.00179	0.00298	0.00236	0.00219	0.00109	0.00244
Q8BK63	KC1A_MOUSE	0.00000	0.00000	0.00000	0.00000	0.00000	0.00000	0.00289	0.00036	0.00207	0.00133	0.00111	0.00000
Q8BK64	AHSA1_MOUSE	0.00000	0.00000	0.00000	0.00000	0.00000	0.00000	0.00994	0.01110	0.01206	0.00813	0.01472	0.00983
Q8BK67	RCC2_MOUSE	0.00000	0.00000	0.00000	0.00000	0.00000	0.00000	0.00327	0.00269	0.00484	0.00400	0.00619	0.00520
Q8BK72	RT27_MOUSE	0.00353	0.00434	0.00453	0.00371	0.00386	0.00261	0.00010	0.00000	0.00023	0.00013	0.00053	0.00036
Q8BKC5	IPO5_MOUSE	0.03337	0.02494	0.01416	0.02448	0.01244	0.01178	0.02602	0.02259	0.02252	0.02693	0.02322	0.02911
Q8BKE6	CP20A_MOUSE	0.00000	0.00000	0.00000	0.00000	0.00000	0.00000	0.00000	0.00285	0.00686	0.00311	0.00511	0.00184
Q8BKG3	PTK7_MOUSE	0.00000	0.00000	0.00000	0.00000	0.00000	0.00000	0.00448	0.01091	0.00934	0.00907	0.00724	0.01048
Q8BKX1	BAIP2_MOUSE	0.00000	0.00000	0.00000	0.00000	0.00000	0.00000	0.00029	0.00081	0.00189	0.00079	0.00147	0.00231
Q8BKZ9	ODPX_MOUSE	0.07508	0.04029	0.06371	0.06862	0.08331	0.05521	0.00739	0.00914	0.01846	0.00525	0.00418	0.00766

Q8BL66	EEA1_MOUSE	0.00868	0.02154	0.00581	0.01351	0.00412	0.00646	0.01055	0.00759	0.01237	0.00826	0.00904	0.00915
Q8BL97	SRSF7_MOUSE	0.00000	0.00000	0.00000	0.00000	0.00000	0.00000	0.10085	0.09318	0.08187	0.07777	0.09594	0.09546
Q8BLF1	NCEH1_MOUSE	0.00265	0.00867	0.00696	0.00689	0.00426	0.00857	0.01034	0.01544	0.01196	0.01067	0.01204	0.01052
Q8BMA6	SRP68_MOUSE	0.00000	0.00000	0.00000	0.00000	0.00000	0.00000	0.00609	0.00423	0.00654	0.00447	0.00917	0.00684
Q8BMB3	IF4E2_MOUSE	0.00000	0.00000	0.00000	0.00000	0.00000	0.00000	0.00000	0.00255	0.00296	0.00232	0.00000	0.00240
Q8BMC1	VATG3_MOUSE	0.00000	0.00000	0.00000	0.00000	0.00000	0.00000	0.00000	0.00000	0.00000	0.00000	0.00000	0.00000
Q8BMD8	SCMC1_MOUSE	0.00000	0.00000	0.00000	0.00000	0.00000	0.00000	0.00101	0.00673	0.00480	0.00425	0.00518	0.00341
Q8BMF3	MAON_MOUSE	0.00958	0.00649	0.01996	0.01060	0.01199	0.00555	0.00000	0.00000	0.00000	0.00000	0.00000	0.00000
Q8BMF4	ODP2_MOUSE	0.65553	0.40225	0.67679	0.49929	0.59765	0.40969	0.05715	0.03560	0.04424	0.03529	0.04761	0.05039
Q8BMG7	RBGPR_MOUSE	0.00000	0.00000	0.00000	0.00000	0.00000	0.00000	0.00000	0.00028	0.00000	0.00000	0.00079	0.00000
Q8BMJ2	SYLC_MOUSE	0.02119	0.01410	0.00659	0.00828	0.00869	0.00889	0.00980	0.00912	0.00536	0.00830	0.01102	0.01001
Q8BMK4	CKAP4_MOUSE	0.00264	0.00190	0.00326	0.00190	0.00097	0.00416	0.01227	0.01557	0.01888	0.01850	0.01393	0.01795
Q8BML9	SYQ_MOUSE	0.00315	0.00229	0.00372	0.00460	0.00343	0.00284	0.01150	0.01048	0.01205	0.01260	0.00953	0.01411
Q8BMP6	GCP60_MOUSE	0.00000	0.00000	0.00000	0.00000	0.00000	0.00000	0.00391	0.00594	0.00996	0.00824	0.00686	0.00399
Q8BMS1	ECHA_MOUSE	1.76867	2.87358	2.11595	1.91218	1.93977	1.72322	0.07854	0.04605	0.06494	0.05839	0.06489	0.05926
Q8BMS4	COQ3_MOUSE	0.02883	0.01662	0.01391	0.01818	0.01635	0.01995	0.00000	0.00000	0.00015	0.00000	0.00000	0.00000
Q8BMS9	RASF2_MOUSE	0.00000	0.00000	0.00000	0.00000	0.00000	0.00000	0.00054	0.00137	0.00232	0.00220	0.00122	0.00307
Q8BND5	QSOX1_MOUSE	0.00208	0.00235	0.00229	0.00466	0.00277	0.00252	0.00582	0.00310	0.00432	0.00743	0.00620	0.00337
Q8BNI4	DERL2_MOUSE	0.00000	0.00000	0.00000	0.00000	0.00000	0.00000	0.00212	0.00000	0.00097	0.00123	0.00000	0.00090
Q8BP40	PPA6_MOUSE	0.00000	0.00000	0.00000	0.00000	0.00000	0.00000	0.00047	0.00092	0.00148	0.00046	0.00148	0.00049
Q8BP47	SYNC_MOUSE	0.00469	0.00349	0.00391	0.00337	0.00333	0.00343	0.01671	0.02415	0.01722	0.01978	0.01712	0.01879
Q8BP67	RL24_MOUSE	0.00000	0.00000	0.00000	0.00000	0.00000	0.00000	0.02608	0.03306	0.02370	0.02662	0.01835	0.01898
Q8BP71	RFOX2_MOUSE	0.00000	0.00000	0.00000	0.00000	0.00000	0.00000	0.00000	0.00000	0.00050	0.00000	0.00000	0.00086
Q8BP92	RCN2_MOUSE	0.00071	0.00048	0.00130	0.00049	0.00135	0.00074	0.04175	0.03533	0.03895	0.03543	0.04876	0.04542
Q8BPB5	FBLN3_MOUSE	0.00000	0.00000	0.00000	0.00000	0.00000	0.00000	0.01159	0.01261	0.01673	0.01442	0.01919	0.01306
Q8BPM0	DAAM1_MOUSE	0.00000	0.00000	0.00000	0.00000	0.00000	0.00000	0.00327	0.00191	0.00327	0.00287	0.00127	0.00433
Q8BPU7	ELMO1_MOUSE	0.00000	0.00000	0.00000	0.00000	0.00000	0.00000	0.00412	0.00546	0.00926	0.00754	0.00916	0.01002
Q8BQ47	CNPY4_MOUSE	0.00000	0.00000	0.00000	0.00000	0.00000	0.00000	0.00160	0.00625	0.00872	0.00718	0.00544	0.00884
Q8BQZ4	RLGPB_MOUSE	0.00000	0.00000	0.00000	0.00000	0.00000	0.00000	0.00000	0.00000	0.00087	0.00000	0.00000	0.00016
Q8BR07	BICD1_MOUSE	0.00000	0.00000	0.00000	0.00000	0.00000	0.00000	0.01185	0.00549	0.00719	0.00851	0.00970	0.00785
Q8BR63	F177A_MOUSE	0.00000	0.00000	0.00000	0.00000	0.00000	0.00000	0.00122	0.00387	0.00279	0.00197	0.00141	0.00331
Q8BR90	CE051_MOUSE	0.00000	0.00000	0.00000	0.00000	0.00000	0.00000	0.00000	0.00000	0.00134	0.00000	0.00383	0.00000
Q8BR92	PALM2_MOUSE	0.00000	0.00000	0.00000	0.00000	0.00000	0.00000	0.00481	0.00700	0.00660	0.00751	0.00480	0.01196
Q8BRF7	SCFD1_MOUSE	0.00000	0.00000	0.00000	0.00000	0.00000	0.00000	0.00599	0.01668	0.01906	0.01017	0.00950	0.00976

Q8BRN9	C2D1B_MOUSE	0.00000	0.00000	0.00000	0.00000	0.00000	0.00000	0.00047	0.00196	0.00234	0.00154	0.00326	0.00118
Q8BSE0	RMD2_MOUSE	0.00000	0.00000	0.00000	0.00000	0.00000	0.00000	0.01174	0.00565	0.00779	0.00630	0.01387	0.00671
Q8BSL7	ARF2_MOUSE	0.00000	0.00000	0.00000	0.00000	0.00000	0.00000	0.00000	0.00874	0.00045	0.00113	0.00000	0.00168
Q8BSY0	ASPH_MOUSE	0.05638	0.05474	0.04262	0.03744	0.03791	0.04977	0.03055	0.02704	0.02413	0.02493	0.01916	0.02574
Q8BT60	CPNE3_MOUSE	0.00000	0.00000	0.00000	0.00000	0.00000	0.00000	0.00411	0.02518	0.01172	0.01235	0.01311	0.01809
Q8BTI8	SRRM2_MOUSE	0.00000	0.00000	0.00000	0.00000	0.00000	0.00000	0.00210	0.00261	0.00875	0.00623	0.00579	0.00465
Q8BTJ4	ENPP4_MOUSE	0.00000	0.00000	0.00000	0.00000	0.00000	0.00000	0.00000	0.00880	0.01234	0.00984	0.01051	0.01131
Q8BTM8	FLNA_MOUSE	0.04168	0.04013	0.03094	0.03521	0.03224	0.04231	0.20257	0.19194	0.20313	0.21575	0.19692	0.19785
Q8BTS4	NUP54_MOUSE	0.00000	0.00000	0.00000	0.00000	0.00000	0.00000	0.00198	0.00409	0.00310	0.00289	0.00000	0.00425
Q8BTU1	CFA20_MOUSE	0.00000	0.00000	0.00000	0.00000	0.00000	0.00000	0.00000	0.00625	0.00193	0.00437	0.00185	0.00313
Q8BTV2	CPSF7_MOUSE	0.00000	0.00000	0.00000	0.00000	0.00000	0.00000	0.00000	0.00129	0.00130	0.00241	0.00116	0.00102
Q8BTY1	KAT1_MOUSE	0.00569	0.00502	0.00748	0.00384	0.00633	0.00388	0.00313	0.00031	0.00000	0.00000	0.00000	0.00015
Q8BTZ7	GMPPB_MOUSE	0.00000	0.00000	0.00000	0.00000	0.00000	0.00000	0.00926	0.00662	0.00783	0.00760	0.00755	0.00908
Q8BU14	SEC62_MOUSE	0.00000	0.00000	0.00000	0.00000	0.00000	0.00000	0.00000	0.00157	0.00317	0.00248	0.00109	0.00110
Q8BU30	SYIC_MOUSE	0.00068	0.00075	0.00130	0.00085	0.00086	0.00109	0.00656	0.00665	0.00644	0.00753	0.00564	0.00500
Q8BU31	RAP2C_MOUSE	0.00000	0.00000	0.00000	0.00000	0.00000	0.00000	0.00456	0.00464	0.00534	0.00526	0.00771	0.00331
Q8BU33	HACL2_MOUSE	0.00000	0.00000	0.00000	0.00000	0.00000	0.00000	0.00000	0.00228	0.00096	0.00051	0.00093	0.00090
Q8BUK6	HOOK3_MOUSE	0.00449	0.00341	0.00215	0.00119	0.00272	0.00168	0.10706	0.30031	0.26152	0.12586	0.15795	0.28959
Q8BUR4	DOCK1_MOUSE	0.00000	0.00000	0.00000	0.00000	0.00000	0.00000	0.00057	0.00229	0.00444	0.00332	0.00428	0.00257
Q8BUV3	GEPH_MOUSE	0.00000	0.00000	0.00000	0.00000	0.00000	0.00000	0.00720	0.00548	0.00389	0.00480	0.00348	0.00673
Q8BV49	IFIX_MOUSE	0.00000	0.00000	0.00000	0.00000	0.00000	0.00000	0.00140	0.00172	0.00308	0.00277	0.00299	0.00336
Q8BVA4	LMOD1_MOUSE	0.00000	0.00000	0.00000	0.00000	0.00000	0.00000	0.00563	0.00403	0.00384	0.00192	0.00325	0.00000
Q8BVE3	VATH_MOUSE	0.00000	0.00000	0.00000	0.00000	0.00000	0.00000	0.02281	0.02188	0.03328	0.02809	0.03592	0.02575
Q8BVI4	DHPR_MOUSE	0.03113	0.01954	0.02477	0.01670	0.01962	0.02090	0.02719	0.03938	0.02517	0.02786	0.04594	0.03443
Q8BVL3	SNX17_MOUSE	0.00000	0.00000	0.00000	0.00000	0.00000	0.00000	0.00177	0.00099	0.00410	0.00350	0.00715	0.00333
Q8BVQ5	PPME1_MOUSE	0.00000	0.00000	0.00000	0.00000	0.00000	0.00000	0.00000	0.00444	0.00300	0.00122	0.00000	0.00246
Q8BVY0	RL1D1_MOUSE	0.00000	0.00000	0.00000	0.00000	0.00000	0.00000	0.00264	0.00310	0.00464	0.00412	0.00209	0.00309
Q8BW75	AOFB_MOUSE	0.05567	0.04306	0.04260	0.05276	0.06101	0.04571	0.05545	0.04997	0.07321	0.06545	0.07549	0.06591
Q8BWF0	SSDH_MOUSE	0.04212	0.04760	0.05319	0.02485	0.04355	0.04219	0.10112	0.09086	0.05460	0.05895	0.04748	0.07973
Q8BWG8	ARRB1_MOUSE	0.00000	0.00000	0.00000	0.00000	0.00000	0.00000	0.01843	0.01976	0.02481	0.02027	0.02286	0.02419
Q8BWM0	PGES2_MOUSE	0.00617	0.00650	0.00683	0.00430	0.00712	0.00761	0.00260	0.00059	0.00279	0.00162	0.00367	0.00136
Q8BWN8	ACOT4_MOUSE	0.00000	0.00000	0.00000	0.00000	0.00000	0.00000	0.00000	0.00000	0.00000	0.00000	0.00000	0.00000
Q8BWQ6	VP35L_MOUSE	0.00000	0.00000	0.00000	0.00000	0.00000	0.00000	0.00121	0.00067	0.00199	0.00070	0.00124	0.00067
Q8BWT1	THIM_MOUSE	0.78669	0.82259	0.64085	0.65165	0.75044	1.12734	0.13182	0.08557	0.09954	0.09299	0.09288	0.09804

Q8BWU5	OSGEP_MOUSE	0.00000	0.00000	0.00000	0.00000	0.00000	0.00000	0.00640	0.00000	0.00000	0.00000	0.00308	0.00000
Q8BWW9	PKN2_MOUSE	0.00000	0.00000	0.00000	0.00000	0.00000	0.00000	0.00556	0.00464	0.00502	0.00641	0.00381	0.00556
Q8BWY2	CLC1A_MOUSE	0.00000	0.00000	0.00000	0.00000	0.00000	0.00000	0.00000	0.00154	0.00000	0.00000	0.00073	0.00000
Q8BWY3	ERF1_MOUSE	0.00000	0.00000	0.00000	0.00000	0.00000	0.00000	0.01353	0.01053	0.00843	0.00978	0.00569	0.00853
Q8BWZ3	NAA25_MOUSE	0.00000	0.00000	0.00000	0.00000	0.00000	0.00000	0.00000	0.00000	0.00000	0.00028	0.00000	0.00062
Q8BX02	KANK2_MOUSE	0.00107	0.00043	0.00127	0.00083	0.00330	0.00115	0.01121	0.02754	0.03195	0.02533	0.02877	0.03191
Q8BX70	VP13C_MOUSE	0.00408	0.01179	0.00746	0.01243	0.00627	0.00706	0.01150	0.00935	0.01165	0.00976	0.01123	0.00983
Q8BXK8	AGAP1_MOUSE	0.00000	0.00000	0.00000	0.00000	0.00000	0.00000	0.00000	0.00464	0.00639	0.00578	0.00290	0.00233
Q8BXK9	CLIC5_MOUSE	0.00392	0.00383	0.00304	0.00562	0.00337	0.00480	0.15760	0.14209	0.20262	0.15068	0.21116	0.20657
Q8BXR9	OSBL6_MOUSE	0.00000	0.00000	0.00000	0.00000	0.00000	0.00000	0.00503	0.00788	0.00811	0.00485	0.01144	0.01003
Q8BXZ1	TMX3_MOUSE	0.00345	0.00340	0.00229	0.00137	0.00214	0.00405	0.00092	0.00430	0.01248	0.00643	0.00326	0.00840
Q8BY87	UBP47_MOUSE	0.00000	0.00000	0.00000	0.00000	0.00000	0.00000	0.00138	0.00341	0.00308	0.00201	0.00119	0.00281
Q8BY89	CTL2_MOUSE	0.00066	0.00091	0.00048	0.00040	0.00046	0.00105	0.01486	0.02340	0.02515	0.01817	0.02187	0.02065
Q8BYA0	TBCD_MOUSE	0.00000	0.00000	0.00000	0.00000	0.00000	0.00000	0.00496	0.00229	0.00367	0.00459	0.00432	0.00482
Q8BYF6	SC5A8_MOUSE	0.00000	0.00000	0.00000	0.00000	0.00000	0.00000	0.00000	0.00000	0.00022	0.00012	0.00000	0.00000
Q8BYK6	YTHD3_MOUSE	0.00000	0.00000	0.00000	0.00000	0.00000	0.00000	0.00098	0.00088	0.00219	0.00039	0.00217	0.00000
Q8BYM7	RSH4A_MOUSE	0.00000	0.00000	0.00000	0.00000	0.00000	0.00000	0.00033	0.00038	0.00068	0.00121	0.00157	0.00181
Q8BYY4	TT39B_MOUSE	0.00000	0.00000	0.00000	0.00000	0.00000	0.00000	0.00040	0.00350	0.00000	0.00066	0.00148	0.00047
Q8BZ03	KPCD2_MOUSE	0.00000	0.00000	0.00000	0.00000	0.00000	0.00000	0.00239	0.00396	0.00617	0.00448	0.00680	0.00656
Q8BZ09	ODC_MOUSE	0.00000	0.00000	0.00000	0.00000	0.00000	0.00000	0.00000	0.00145	0.00025	0.00031	0.00000	0.00102
Q8BZ98	DYN3_MOUSE	0.00000	0.00000	0.00000	0.00000	0.00000	0.00000	0.00844	0.00460	0.00983	0.00741	0.01015	0.01207
Q8BZF8	PGM5_MOUSE	0.02487	0.02176	0.01838	0.01195	0.02074	0.02114	0.15020	0.13728	0.07331	0.10613	0.10808	0.10798
Q8BZW8	NHLC2_MOUSE	0.00585	0.00303	0.00290	0.00432	0.00296	0.00265	0.01409	0.00372	0.01026	0.00615	0.00531	0.00855
Q8BZZ3	WWP1_MOUSE	0.00000	0.00000	0.00000	0.00000	0.00000	0.00000	0.00681	0.00180	0.00970	0.00676	0.01080	0.00806
Q8C052	MAP1S_MOUSE	0.00000	0.00000	0.00000	0.00000	0.00000	0.00000	0.00000	0.00000	0.00256	0.00132	0.00092	0.00071
Q8C079	STRP1_MOUSE	0.00000	0.00000	0.00000	0.00000	0.00000	0.00000	0.00439	0.00077	0.00513	0.00348	0.00634	0.00489
Q8C0C7	SYFA_MOUSE	0.00414	0.00887	0.00344	0.00279	0.00272	0.00332	0.00176	0.00319	0.00285	0.00308	0.00304	0.00339
Q8C0D4	RHG12_MOUSE	0.00000	0.00000	0.00000	0.00000	0.00000	0.00000	0.00000	0.00000	0.00179	0.00088	0.00000	0.00122
Q8C0D5	EFL1_MOUSE	0.00000	0.00000	0.00000	0.00000	0.00000	0.00000	0.00000	0.00000	0.00233	0.00437	0.00284	0.00480
Q8C0E2	VP26B_MOUSE	0.00000	0.00000	0.00000	0.00000	0.00000	0.00000	0.00910	0.00896	0.00873	0.00957	0.01024	0.00792
Q8C0I1	ADAS_MOUSE	0.00000	0.00000	0.00000	0.00000	0.00000	0.00000	0.00381	0.00219	0.00486	0.00272	0.00809	0.00533
Q8C0L0	TMX4_MOUSE	0.00339	0.00333	0.00271	0.00263	0.00271	0.00290	0.00649	0.00667	0.00724	0.00570	0.00532	0.00573
Q8C0L6	PAOX_MOUSE	0.00000	0.00000	0.00000	0.00000	0.00000	0.00000	0.00042	0.00058	0.00107	0.00134	0.00000	0.00232
Q8C129	LCAP_MOUSE	0.00293	0.00142	0.00299	0.00395	0.00179	0.00254	0.00255	0.00662	0.00718	0.00539	0.00502	0.00838

Q8C142	ARH_MOUSE	0.00000	0.00000	0.00000	0.00000	0.00000	0.00000	0.00271	0.00280	0.00272	0.00307	0.00275	0.00255
Q8C163	EXOG_MOUSE	0.00000	0.00000	0.00000	0.00000	0.00000	0.00000	0.00000	0.00000	0.00124	0.00033	0.00000	0.00016
Q8C165	P20D1_MOUSE	0.00000	0.00000	0.00000	0.00000	0.00000	0.00000	0.00003	0.00000	0.00002	0.00000	0.00003	0.00002
Q8C166	CPNE1_MOUSE	0.00000	0.00000	0.00000	0.00000	0.00000	0.00000	0.01343	0.01687	0.01553	0.01512	0.01858	0.01539
Q8C1A5	THOP1_MOUSE	0.00522	0.00307	0.03948	0.01969	0.00843	0.00623	0.00989	0.01237	0.01259	0.01649	0.02102	0.01243
Q8C1B7	SEP11_MOUSE	0.00000	0.00000	0.00000	0.00000	0.00000	0.00000	0.01844	0.02585	0.03960	0.02688	0.03042	0.02105
Q8C1E7	TACAN_MOUSE	0.00000	0.00000	0.00000	0.00000	0.00000	0.00000	0.00000	0.00000	0.00000	0.00000	0.00064	0.00000
Q8C2E7	WASC5_MOUSE	0.00000	0.00000	0.00000	0.00000	0.00000	0.00000	0.00208	0.00279	0.00330	0.00253	0.00282	0.00306
Q8C2Q3	RBM14_MOUSE	0.00000	0.00000	0.00000	0.00000	0.00000	0.00000	0.00000	0.00536	0.00423	0.00668	0.00238	0.00247
Q8C3J5	DOCK2_MOUSE	0.00000	0.00000	0.00000	0.00000	0.00000	0.00000	0.00000	0.00159	0.00490	0.00285	0.00122	0.00000
Q8C3K6	SC5A1_MOUSE	0.00000	0.00000	0.00000	0.00000	0.00000	0.00000	0.00000	0.00000	0.00000	0.00000	0.00000	0.00000
Q8C3W1	CA198_MOUSE	0.00000	0.00000	0.00000	0.00000	0.00000	0.00000	0.00000	0.00000	0.00000	0.00237	0.00678	0.00184
Q8C3X4	GUF1_MOUSE	0.00000	0.00000	0.00000	0.00000	0.00000	0.00000	0.00000	0.00000	0.00366	0.00000	0.00000	0.00000
Q8C3X8	LMF2_MOUSE	0.00000	0.00000	0.00000	0.00000	0.00000	0.00000	0.00498	0.00517	0.01437	0.00889	0.01103	0.00649
Q8C522	ENDD1_MOUSE	0.00000	0.00000	0.00000	0.00000	0.00000	0.00000	0.00982	0.01645	0.01154	0.01191	0.00841	0.01402
Q8C5H8	NAKD2_MOUSE	0.00000	0.00000	0.00000	0.00000	0.00000	0.00000	0.00861	0.00562	0.00545	0.00539	0.00718	0.00485
Q8C5Q4	GRSF1_MOUSE	0.00000	0.00000	0.00000	0.00000	0.00000	0.00000	0.00838	0.00450	0.00538	0.00668	0.00804	0.00293
Q8C5W0	CLMN_MOUSE	0.00000	0.00000	0.00000	0.00000	0.00000	0.00000	0.00000	0.00167	0.00000	0.00000	0.00000	0.00559
Q8C650	SEP10_MOUSE	0.00000	0.00000	0.00000	0.00000	0.00000	0.00000	0.00747	0.00892	0.01296	0.00631	0.00158	0.00188
Q8C6B2	RTKN_MOUSE	0.00000	0.00000	0.00000	0.00000	0.00000	0.00000	0.00000	0.00098	0.00205	0.00078	0.00179	0.00000
Q8C6G8	WDR26_MOUSE	0.00000	0.00000	0.00000	0.00000	0.00000	0.00000	0.00000	0.00172	0.00247	0.00235	0.00320	0.00192
Q8C6K9	CO6A6_MOUSE	0.01394	0.03189	0.01600	0.01886	0.01264	0.01426	0.00154	0.00000	0.00118	0.00000	0.00000	0.00000
Q8C6S9	CFA54_MOUSE	0.00000	0.00000	0.00000	0.00000	0.00000	0.00000	0.00319	0.00000	0.00274	0.00230	0.00536	0.00576
Q8C6U2	S66A3_MOUSE	0.00000	0.00000	0.00000	0.00000	0.00000	0.00000	0.00344	0.00255	0.00550	0.00354	0.00419	0.00439
Q8C7D2	CRBN_MOUSE	0.00000	0.00000	0.00000	0.00000	0.00000	0.00000	0.00000	0.00139	0.00026	0.00000	0.00000	0.00026
Q8C7K6	PCYXL_MOUSE	0.00000	0.00000	0.00000	0.00000	0.00000	0.00000	0.00621	0.00530	0.00458	0.00461	0.00518	0.00446
Q8C7R4	UBA6_MOUSE	0.00000	0.00000	0.00000	0.00000	0.00000	0.00000	0.00168	0.00594	0.00459	0.00394	0.00140	0.00738
Q8C854	MYEF2_MOUSE	0.00000	0.00000	0.00000	0.00000	0.00000	0.00000	0.00583	0.00870	0.00694	0.00430	0.00661	0.00482
Q8C878	UBA3_MOUSE	0.00000	0.00000	0.00000	0.00000	0.00000	0.00000	0.00361	0.00933	0.01174	0.00720	0.01325	0.01297
Q8C8T8	TSR2_MOUSE	0.00000	0.00000	0.00000	0.00000	0.00000	0.00000	0.00504	0.00479	0.00204	0.00180	0.00000	0.00341
Q8C8U0	LIPB1_MOUSE	0.00000	0.00000	0.00000	0.00000	0.00000	0.00000	0.00506	0.00486	0.00655	0.00677	0.00456	0.00831
Q8C996	TM163_MOUSE	0.00000	0.00000	0.00000	0.00000	0.00000	0.00000	0.00346	0.00525	0.00839	0.00529	0.00458	0.00286
Q8CAA7	PGM2L_MOUSE	0.00000	0.00000	0.00000	0.00000	0.00000	0.00000	0.00000	0.00000	0.00000	0.00000	0.00000	0.00000
Q8CAK1	CAF17_MOUSE	0.00000	0.00000	0.00000	0.00000	0.00000	0.00000	0.00000	0.00000	0.00000	0.00000	0.00000	0.00000

Q8CAQ8	MIC60_MOUSE	0.20783	0.14578	0.18226	0.17959	0.15615	0.17452	0.04251	0.04239	0.03724	0.03832	0.03042	0.03607
Q8CAY6	THIC_MOUSE	0.00000	0.00000	0.00000	0.00000	0.00000	0.00000	0.01083	0.01206	0.01760	0.00499	0.00944	0.00604
Q8CBA2	SLFN5_MOUSE	0.00000	0.00000	0.00000	0.00000	0.00000	0.00000	0.00092	0.00160	0.01452	0.00635	0.01009	0.00830
Q8CBE3	WDR37_MOUSE	0.00000	0.00000	0.00000	0.00000	0.00000	0.00000	0.00501	0.00629	0.00131	0.00338	0.00214	0.00513
Q8CBW3	ABI1_MOUSE	0.00000	0.00000	0.00000	0.00000	0.00000	0.00000	0.01251	0.01233	0.01574	0.01364	0.01661	0.01565
Q8CBY8	DCTN4_MOUSE	0.00000	0.00000	0.00000	0.00000	0.00000	0.00000	0.00601	0.01043	0.00871	0.00878	0.00952	0.01267
Q8CC35	SYNPO_MOUSE	0.00580	0.00483	0.00461	0.00743	0.00555	0.00431	0.01752	0.01215	0.01472	0.01201	0.00610	0.01499
Q8CC86	PNCB_MOUSE	0.00000	0.00000	0.00000	0.00000	0.00000	0.00000	0.01091	0.01173	0.01652	0.02799	0.01026	0.01431
Q8CC88	VWA8_MOUSE	0.06502	0.09318	0.07146	0.06151	0.08390	0.08014	0.00078	0.00003	0.00030	0.00103	0.00016	0.00069
Q8CCF0	PRP31_MOUSE	0.00000	0.00000	0.00000	0.00000	0.00000	0.00000	0.00290	0.00069	0.00501	0.00209	0.00614	0.00656
Q8CCH2	NHLC3_MOUSE	0.00000	0.00000	0.00000	0.00000	0.00000	0.00000	0.00000	0.00000	0.00000	0.00000	0.00000	0.00070
Q8CCJ3	UFL1_MOUSE	0.00000	0.00000	0.00000	0.00000	0.00000	0.00000	0.00768	0.00160	0.00559	0.00426	0.00491	0.00159
Q8CCK0	H2AW_MOUSE	0.00000	0.00000	0.00000	0.00000	0.00000	0.00000	0.01275	0.00790	0.01212	0.00984	0.00891	0.01241
Q8CCS6	PABP2_MOUSE	0.00000	0.00000	0.00000	0.00000	0.00000	0.00000	0.00759	0.00663	0.00506	0.00473	0.00444	0.00782
Q8CD10	MICU2_MOUSE	0.00000	0.00000	0.00000	0.00000	0.00000	0.00000	0.00000	0.00000	0.00284	0.00000	0.00296	0.00252
Q8CDA1	SAC2_MOUSE	0.00000	0.00000	0.00000	0.00000	0.00000	0.00000	0.00000	0.00000	0.00000	0.00000	0.00000	0.00000
Q8CDJ8	STON1_MOUSE	0.00000	0.00000	0.00000	0.00000	0.00000	0.00000	0.00000	0.00195	0.00207	0.00184	0.00078	0.00109
Q8CDM8	F16B1_MOUSE	0.00000	0.00000	0.00000	0.00000	0.00000	0.00000	0.00000	0.00069	0.00000	0.00000	0.00015	0.00036
Q8CDN6	TXNL1_MOUSE	0.00074	0.00103	0.00080	0.00125	0.00171	0.00160	0.01609	0.01936	0.02292	0.03133	0.02297	0.02282
Q8CEZ4	MB214_MOUSE	0.00000	0.00000	0.00000	0.00000	0.00000	0.00000	0.00000	0.00048	0.00000	0.00081	0.00111	0.00114
Q8CFB4	GBP5_MOUSE	0.00000	0.00000	0.00000	0.00000	0.00000	0.00000	0.00043	0.00012	0.00063	0.00021	0.00054	0.00031
Q8CFD4	SNX8_MOUSE	0.00000	0.00000	0.00000	0.00000	0.00000	0.00000	0.00000	0.00000	0.00000	0.00000	0.00028	0.00000
Q8CFE3	RCOR1_MOUSE	0.00000	0.00000	0.00000	0.00000	0.00000	0.00000	0.00182	0.00000	0.00310	0.00000	0.00000	0.00000
Q8CFE4	SCYL2_MOUSE	0.00000	0.00000	0.00000	0.00000	0.00000	0.00000	0.00239	0.00085	0.00394	0.00222	0.00455	0.00324
Q8CFI0	NED4L_MOUSE	0.00000	0.00000	0.00000	0.00000	0.00000	0.00000	0.00610	0.01135	0.00739	0.00651	0.00660	0.00992
Q8CFI7	RPB2_MOUSE	0.00000	0.00000	0.00000	0.00000	0.00000	0.00000	0.00000	0.00111	0.00000	0.00000	0.00000	0.00000
Q8CFJ7	S2545_MOUSE	0.00000	0.00000	0.00000	0.00000	0.00000	0.00000	0.00311	0.00536	0.00316	0.00361	0.00000	0.00000
Q8CFX1	G6PE_MOUSE	0.00000	0.00000	0.00000	0.00000	0.00000	0.00000	0.01273	0.02163	0.01676	0.01735	0.01124	0.01955
Q8CG03	PDE5A_MOUSE	0.00000	0.00000	0.00000	0.00000	0.00000	0.00000	0.01753	0.01897	0.01766	0.02020	0.01860	0.01761
Q8CG19	LTBP1_MOUSE	0.00000	0.00000	0.00000	0.00000	0.00000	0.00000	0.00142	0.00326	0.00584	0.00182	0.00570	0.00244
Q8CG50	RAB43_MOUSE	0.00000	0.00000	0.00000	0.00000	0.00000	0.00000	0.00000	0.00000	0.00000	0.00000	0.00000	0.00000
Q8CG72	ADPRS_MOUSE	0.00000	0.00000	0.00000	0.00000	0.00000	0.00000	0.00369	0.00652	0.00555	0.00490	0.00712	0.00718
Q8CG76	ARK72_MOUSE	0.01418	0.00758	0.01806	0.00810	0.00784	0.00931	0.01753	0.01416	0.01970	0.01727	0.01565	0.01455
Q8CG79	ASPP2_MOUSE	0.00000	0.00000	0.00000	0.00000	0.00000	0.00000	0.00957	0.00135	0.00625	0.00379	0.00637	0.00467

Q8CGA0	PPM1F_MOUSE	0.00000	0.00000	0.00000	0.00000	0.00000	0.00000	0.00779	0.00780	0.01521	0.01086	0.01221	0.00752
Q8CGB6	TNS2_MOUSE	0.00000	0.00000	0.00000	0.00000	0.00000	0.00000	0.03287	0.04359	0.03751	0.02895	0.03912	0.04198
Q8CGC7	SYEP_MOUSE	0.00811	0.01380	0.01140	0.01347	0.00837	0.00686	0.00845	0.00615	0.00786	0.00734	0.00845	0.00727
Q8CGF1	RHG29_MOUSE	0.00000	0.00000	0.00000	0.00000	0.00000	0.00000	0.00000	0.00022	0.00065	0.00000	0.00027	0.00026
Q8CGF7	TCRG1_MOUSE	0.00000	0.00000	0.00000	0.00000	0.00000	0.00000	0.00278	0.00000	0.00454	0.00252	0.00102	0.00284
Q8CGK3	LONM_MOUSE	0.07567	0.04836	0.06407	0.04665	0.07394	0.05749	0.02300	0.01803	0.01637	0.01697	0.01877	0.02203
Q8CGN5	PLIN1_MOUSE	0.00000	0.00000	0.00000	0.00000	0.00000	0.00000	0.02292	0.00131	0.03256	0.00140	0.00017	0.00186
Q8CGY8	OGT1_MOUSE	0.00127	0.00016	0.00056	0.00106	0.00044	0.00119	0.00000	0.00000	0.00382	0.00267	0.00101	0.00152
Q8CH18	CCAR1_MOUSE	0.00000	0.00000	0.00000	0.00000	0.00000	0.00000	0.00000	0.00119	0.00419	0.00092	0.00181	0.00315
Q8CH25	SLTM_MOUSE	0.00000	0.00000	0.00000	0.00000	0.00000	0.00000	0.00153	0.00602	0.00143	0.00261	0.00025	0.00403
Q8CHD8	RFIP3_MOUSE	0.00000	0.00000	0.00000	0.00000	0.00000	0.00000	0.00000	0.00000	0.00000	0.00000	0.00000	0.00000
Q8CHE4	PHLP1_MOUSE	0.00000	0.00000	0.00000	0.00000	0.00000	0.00000	0.00000	0.00000	0.00000	0.00000	0.00000	0.00000
Q8CHH9	SEPT8_MOUSE	0.00000	0.00000	0.00000	0.00000	0.00000	0.00000	0.01161	0.01504	0.02725	0.01731	0.02198	0.01396
Q8CHK3	MBOA7_MOUSE	0.00000	0.00000	0.00000	0.00000	0.00000	0.00000	0.00190	0.00476	0.00185	0.00260	0.00239	0.00293
Q8CHP5	PYM1_MOUSE	0.00000	0.00000	0.00000	0.00000	0.00000	0.00000	0.00000	0.00103	0.00238	0.00497	0.00388	0.00363
Q8CHP8	PGP_MOUSE	0.01364	0.00790	0.00981	0.01264	0.01071	0.01141	0.00000	0.00318	0.00632	0.00456	0.00257	0.00435
Q8CHR6	DPYD_MOUSE	0.00000	0.00000	0.00000	0.00000	0.00000	0.00000	0.00000	0.00000	0.00228	0.00000	0.00000	0.00000
Q8CHS8	VP37A_MOUSE	0.00000	0.00000	0.00000	0.00000	0.00000	0.00000	0.00000	0.00000	0.00000	0.00000	0.00000	0.00000
Q8CHT0	AL4A1_MOUSE	0.04492	0.05043	0.02914	0.02705	0.03886	0.05543	0.03741	0.04422	0.04328	0.03580	0.03930	0.03813
Q8CHU3	EPN2_MOUSE	0.00000	0.00000	0.00000	0.00000	0.00000	0.00000	0.00409	0.00853	0.01211	0.01090	0.01147	0.01226
Q8CHW4	EI2BE_MOUSE	0.00000	0.00000	0.00000	0.00000	0.00000	0.00000	0.00000	0.00051	0.00069	0.00000	0.00115	0.00181
Q8CI08	SLAI2_MOUSE	0.00000	0.00000	0.00000	0.00000	0.00000	0.00000	0.00471	0.00455	0.00399	0.00468	0.00478	0.00297
Q8CI12	SMTL2_MOUSE	0.00000	0.00000	0.00000	0.00000	0.00000	0.00000	0.00000	0.00000	0.00294	0.00000	0.00130	0.00000
Q8CI32	BAG5_MOUSE	0.00253	0.00140	0.00080	0.00182	0.00082	0.00131	0.00237	0.00297	0.00423	0.00365	0.00570	0.00448
Q8CI51	PDLI5_MOUSE	0.01348	0.00225	0.01470	0.01575	0.00591	0.00359	0.03027	0.02302	0.02830	0.02336	0.03103	0.01941
Q8CI59	STEA3_MOUSE	0.00000	0.00000	0.00000	0.00000	0.00000	0.00000	0.00085	0.00000	0.00212	0.00000	0.00177	0.00000
Q8CI78	RMND1_MOUSE	0.00000	0.00000	0.00000	0.00000	0.00000	0.00000	0.00000	0.00354	0.00644	0.00409	0.00676	0.00704
Q8CI94	PYGB_MOUSE	0.13760	0.14390	0.29421	0.23837	0.21903	0.20918	0.04774	0.03479	0.05182	0.04684	0.04926	0.04374
Q8CIB5	FERM2_MOUSE	0.00719	0.00717	0.01266	0.00659	0.00744	0.00578	0.14650	0.14411	0.17219	0.15116	0.16899	0.14891
Q8CIE6	COPA_MOUSE	0.00000	0.00000	0.00000	0.00000	0.00000	0.00000	0.03098	0.02502	0.03694	0.02908	0.03838	0.02949
Q8CIF4	BTD_MOUSE	0.00000	0.00000	0.00000	0.00000	0.00000	0.00000	0.00000	0.00000	0.00000	0.00000	0.00000	0.00000
Q8CIH5	PLCG2_MOUSE	0.00000	0.00000	0.00000	0.00000	0.00000	0.00000	0.01157	0.01230	0.01071	0.01205	0.00644	0.01752
Q8CIM7	CP2DQ_MOUSE	0.00000	0.00000	0.00000	0.00000	0.00000	0.00000	0.01120	0.00630	0.01394	0.01016	0.02131	0.01380
Q8CIN4	PAK2_MOUSE	0.00000	0.00000	0.00000	0.00000	0.00000	0.00000	0.01941	0.02675	0.03646	0.03006	0.03515	0.02473

Q8CIZ8	VWF_MOUSE	0.00000	0.00000	0.00000	0.00000	0.00000	0.00000	0.03420	0.03058	0.03539	0.03124	0.02773	0.02742
Q8CJ40	CROCC_MOUSE	0.00000	0.00000	0.00000	0.00000	0.00000	0.00000	0.00000	0.00913	0.00000	0.00000	0.00000	0.00087
Q8CJ53	CIP4_MOUSE	0.00000	0.00000	0.00000	0.00000	0.00000	0.00000	0.00303	0.00622	0.01342	0.00541	0.00844	0.00647
Q8CJG0	AGO2_MOUSE	0.00000	0.00000	0.00000	0.00000	0.00000	0.00000	0.00538	0.00277	0.00520	0.00375	0.00685	0.00347
Q8JZK9	HMCS1_MOUSE	0.00000	0.00000	0.00000	0.00000	0.00000	0.00000	0.00000	0.00000	0.00049	0.00000	0.00000	0.00000
Q8JZM2	PR15L_MOUSE	0.00000	0.00000	0.00000	0.00000	0.00000	0.00000	0.01434	0.00410	0.01363	0.00195	0.00152	0.00106
Q8JZN5	ACAD9_MOUSE	0.02182	0.01307	0.01931	0.01883	0.01832	0.02690	0.01770	0.01521	0.01649	0.01181	0.02047	0.01469
Q8JZN7	MIRO2_MOUSE	0.00028	0.00100	0.00053	0.00099	0.00082	0.00015	0.00000	0.00000	0.00000	0.00000	0.00000	0.00000
Q8JZQ2	AFG32_MOUSE	0.01025	0.00666	0.00989	0.00926	0.00905	0.00782	0.08172	0.07662	0.08997	0.05033	0.07517	0.06132
Q8JZQ9	EIF3B_MOUSE	0.00134	0.00365	0.00124	0.00192	0.00193	0.00190	0.02221	0.03011	0.03057	0.02289	0.02438	0.02874
Q8JZR0	ACSL5_MOUSE	0.00000	0.00000	0.00000	0.00000	0.00000	0.00000	0.01524	0.01197	0.01499	0.01445	0.01456	0.01311
Q8JZU2	TXTP_MOUSE	0.00000	0.00000	0.00000	0.00000	0.00000	0.00000	0.01044	0.00739	0.00959	0.00377	0.00224	0.00393
Q8JZU6	PXDC1_MOUSE	0.00000	0.00000	0.00000	0.00000	0.00000	0.00000	0.00000	0.00262	0.00137	0.00000	0.00000	0.00183
Q8JZV9	BDH2_MOUSE	0.00000	0.00000	0.00000	0.00000	0.00000	0.00000	0.00000	0.00111	0.00022	0.00000	0.00019	0.00000
Q8JZZ0	UD3A2_MOUSE	0.00000	0.00000	0.00000	0.00000	0.00000	0.00000	0.00000	0.00000	0.00001	0.00000	0.00000	0.00001
Q8K003	TMA7_MOUSE	0.00000	0.00000	0.00000	0.00000	0.00000	0.00000	0.00000	0.00139	0.00076	0.00042	0.00042	0.00067
Q8K010	OPLA_MOUSE	0.00721	0.00267	0.01129	0.00438	0.00298	0.00293	0.01445	0.01261	0.01964	0.01762	0.01695	0.01699
Q8K012	FBP1L_MOUSE	0.00000	0.00000	0.00000	0.00000	0.00000	0.00000	0.00869	0.01089	0.01170	0.00963	0.01122	0.01085
Q8K019	BCLF1_MOUSE	0.00000	0.00000	0.00000	0.00000	0.00000	0.00000	0.00000	0.00099	0.00310	0.00136	0.00093	0.00135
Q8K021	SCAM1_MOUSE	0.00000	0.00000	0.00000	0.00000	0.00000	0.00000	0.00711	0.00491	0.00552	0.00523	0.00578	0.00544
Q8K064	F174B_MOUSE	0.00000	0.00000	0.00000	0.00000	0.00000	0.00000	0.00000	0.00304	0.00102	0.00219	0.00200	0.00121
Q8K0B2	LMBD1_MOUSE	0.00000	0.00000	0.00000	0.00000	0.00000	0.00000	0.00287	0.00143	0.00505	0.00178	0.00648	0.00308
Q8K0C4	CP51A_MOUSE	0.00000	0.00000	0.00000	0.00000	0.00000	0.00000	0.00529	0.00000	0.00070	0.00155	0.00221	0.00000
Q8K0C9	GMDS_MOUSE	0.00000	0.00000	0.00000	0.00000	0.00000	0.00000	0.00453	0.00357	0.00553	0.00442	0.00657	0.00560
Q8K0D5	EFGM_MOUSE	0.02792	0.01835	0.01851	0.02028	0.01778	0.01352	0.00000	0.00043	0.00041	0.00073	0.00115	0.00103
Q8K0E3	SC5AB_MOUSE	0.00000	0.00000	0.00000	0.00000	0.00000	0.00000	0.00000	0.00000	0.00000	0.00000	0.00000	0.00000
Q8K0E8	FIBB_MOUSE	0.10323	0.04382	0.04963	0.10384	0.06935	0.06221	0.12974	0.05780	0.06472	0.07810	0.12879	0.04147
Q8K0H1	S47A1_MOUSE	0.00000	0.00000	0.00000	0.00000	0.00000	0.00000	0.00000	0.00000	0.00000	0.00000	0.00000	0.00000
Q8K0H5	TAF10_MOUSE	0.00000	0.00000	0.00000	0.00000	0.00000	0.00000	0.00000	0.00000	0.00026	0.00000	0.00000	0.00021
Q8K0L3	ACSM2_MOUSE	0.00000	0.00000	0.00000	0.00000	0.00000	0.00000	0.00000	0.00000	0.00000	0.00000	0.00001	0.00000
Q8K0L9	ZBT20_MOUSE	0.00000	0.00000	0.00000	0.00000	0.00000	0.00000	0.00023	0.00018	0.00162	0.00052	0.00106	0.00087
Q8K0T0	RTN1_MOUSE	0.00000	0.00000	0.00000	0.00000	0.00000	0.00000	0.00000	0.00432	0.00634	0.00369	0.00060	0.00156
Q8K0U4	HS12A_MOUSE	0.00000	0.00000	0.00000	0.00000	0.00000	0.00000	0.01657	0.00950	0.01628	0.01365	0.01428	0.01524
Q8K0Z7	TACO1_MOUSE	0.01079	0.00727	0.00861	0.01244	0.00933	0.00514	0.00000	0.00000	0.00000	0.00000	0.00000	0.00000

Q8K124	PKHO2_MOUSE	0.00000	0.00000	0.00000	0.00000	0.00000	0.00000	0.00412	0.00000	0.00696	0.00482	0.01454	0.00537
Q8K157	GALM_MOUSE	0.00000	0.00000	0.00000	0.00000	0.00000	0.00000	0.00000	0.00168	0.00077	0.00121	0.00074	0.00089
Q8K182	CO8A_MOUSE	0.00000	0.00000	0.00000	0.00000	0.00000	0.00000	0.00999	0.00717	0.00198	0.00063	0.00675	0.00010
Q8K183	PDXK_MOUSE	0.00000	0.00000	0.00000	0.00000	0.00000	0.00000	0.01088	0.01447	0.01714	0.01536	0.01392	0.01206
Q8K1B8	URP2_MOUSE	0.00000	0.00000	0.00000	0.00000	0.00000	0.00000	0.01609	0.00995	0.02151	0.02669	0.01831	0.02078
Q8K1E0	STX5_MOUSE	0.00000	0.00000	0.00000	0.00000	0.00000	0.00000	0.00625	0.00715	0.00466	0.00599	0.00534	0.00471
Q8K1J6	TRNT1_MOUSE	0.00000	0.00000	0.00000	0.00000	0.00000	0.00000	0.01304	0.01540	0.01795	0.01487	0.02234	0.02090
Q8K1L5	PP1RB_MOUSE	0.00000	0.00000	0.00000	0.00000	0.00000	0.00000	0.00000	0.00000	0.00094	0.00093	0.00046	0.00000
Q8K1M6	DNM1L_MOUSE	0.01224	0.01583	0.02270	0.01903	0.02238	0.02337	0.01246	0.01356	0.01417	0.01321	0.01379	0.01431
Q8K1N2	PHLB2_MOUSE	0.00000	0.00000	0.00000	0.00000	0.00000	0.00000	0.00549	0.00473	0.00715	0.00528	0.00564	0.00273
Q8K1R3	PNPT1_MOUSE	0.00185	0.00130	0.00140	0.00126	0.00133	0.00124	0.00000	0.00000	0.00000	0.00000	0.00128	0.00000
Q8K1R7	NEK9_MOUSE	0.00000	0.00000	0.00000	0.00000	0.00000	0.00000	0.00477	0.00378	0.00571	0.00504	0.00578	0.00516
Q8K1X1	WDR11_MOUSE	0.00000	0.00000	0.00000	0.00000	0.00000	0.00000	0.01124	0.00936	0.01208	0.00810	0.00516	0.01159
Q8K1Z0	COQ9_MOUSE	0.03680	0.03050	0.03328	0.05316	0.04531	0.01272	0.00118	0.00555	0.00589	0.00561	0.00132	0.00248
Q8K212	PACS1_MOUSE	0.00000	0.00000	0.00000	0.00000	0.00000	0.00000	0.00455	0.00240	0.00487	0.00208	0.00446	0.00244
Q8K221	ARFP2_MOUSE	0.00000	0.00000	0.00000	0.00000	0.00000	0.00000	0.00000	0.00000	0.00000	0.00000	0.00116	0.00114
Q8K273	MMGT1_MOUSE	0.00000	0.00000	0.00000	0.00000	0.00000	0.00000	0.00000	0.00141	0.00000	0.00107	0.00000	0.00000
Q8K274	KT3K_MOUSE	0.00000	0.00000	0.00000	0.00000	0.00000	0.00000	0.00000	0.00090	0.00185	0.00169	0.00084	0.00087
Q8K297	GT251_MOUSE	0.00000	0.00000	0.00000	0.00000	0.00000	0.00000	0.00128	0.00198	0.00568	0.00305	0.00307	0.00547
Q8K2A1	GULP1_MOUSE	0.00000	0.00000	0.00000	0.00000	0.00000	0.00000	0.00000	0.00745	0.00123	0.00577	0.00000	0.00182
Q8K2B3	SDHA_MOUSE	0.75981	0.53408	0.95580	0.91757	0.94046	0.55475	0.02651	0.02855	0.03355	0.03161	0.01819	0.02727
Q8K2C6	SIR5_MOUSE	0.01523	0.01159	0.01071	0.01453	0.01525	0.01534	0.00113	0.00000	0.00298	0.00118	0.00124	0.00109
Q8K2C9	HACD3_MOUSE	0.00000	0.00000	0.00000	0.00000	0.00000	0.00000	0.03036	0.01566	0.03944	0.02790	0.04813	0.03061
Q8K2F8	LS14A_MOUSE	0.00000	0.00000	0.00000	0.00000	0.00000	0.00000	0.01098	0.01063	0.00819	0.00925	0.01165	0.00695
Q8K2H2	OTU6B_MOUSE	0.00000	0.00000	0.00000	0.00000	0.00000	0.00000	0.00254	0.01060	0.00259	0.00366	0.00710	0.00567
Q8K2I1	FNTB_MOUSE	0.00000	0.00000	0.00000	0.00000	0.00000	0.00000	0.01960	0.00326	0.00355	0.00490	0.00274	0.00448
Q8K2I3	FMO2_MOUSE	0.00638	0.00997	0.00275	0.00401	0.00365	0.00372	0.27292	0.26832	0.29190	0.26542	0.26598	0.29807
Q8K2K6	AGFG1_MOUSE	0.00000	0.00000	0.00000	0.00000	0.00000	0.00000	0.00956	0.01000	0.01733	0.00941	0.01948	0.01404
Q8K2L8	TPC12_MOUSE	0.00000	0.00000	0.00000	0.00000	0.00000	0.00000	0.00595	0.01351	0.00941	0.01402	0.01655	0.01164
Q8K2M0	RM38_MOUSE	0.00000	0.00000	0.00000	0.00000	0.00000	0.00000	0.00000	0.00000	0.00000	0.00000	0.00000	0.00000
Q8K2Q7	BROX_MOUSE	0.00000	0.00000	0.00000	0.00000	0.00000	0.00000	0.00559	0.00000	0.00402	0.00378	0.00199	0.00407
Q8K2Q9	SHOT1_MOUSE	0.00000	0.00000	0.00000	0.00000	0.00000	0.00000	0.00000	0.00308	0.01128	0.00564	0.00638	0.00362
Q8K2T1	NMRL1_MOUSE	0.00000	0.00000	0.00000	0.00000	0.00000	0.00000	0.00098	0.00000	0.00389	0.00076	0.00331	0.00202
Q8K2Y3	EVA1B_MOUSE	0.00000	0.00000	0.00000	0.00000	0.00000	0.00000	0.02668	0.02184	0.02498	0.02634	0.03975	0.03983

Q8K310	MATR3_MOUSE	0.00297	0.00380	0.00413	0.00550	0.00409	0.00616	0.01802	0.01762	0.02682	0.02472	0.01763	0.02404
Q8K337	I5P2_MOUSE	0.00000	0.00000	0.00000	0.00000	0.00000	0.00000	0.00351	0.00571	0.00959	0.00650	0.00751	0.00703
Q8K353	CYTM1_MOUSE	0.00000	0.00000	0.00000	0.00000	0.00000	0.00000	0.00000	0.00000	0.00547	0.01612	0.00150	0.00569
Q8K354	CBR3_MOUSE	0.00000	0.00000	0.00000	0.00000	0.00000	0.00000	0.01475	0.01413	0.01466	0.01393	0.01241	0.01320
Q8K370	ACD10_MOUSE	0.03121	0.02095	0.02955	0.01722	0.02925	0.03481	0.00703	0.00583	0.00958	0.00774	0.00855	0.00866
Q8K3A0	HSC20_MOUSE	0.00347	0.00531	0.00298	0.00230	0.00470	0.00301	0.00650	0.00755	0.00000	0.00287	0.00000	0.00670
Q8K3C3	LZIC_MOUSE	0.00000	0.00000	0.00000	0.00000	0.00000	0.00000	0.00372	0.00737	0.00328	0.00412	0.00127	0.00204
Q8K3H0	DP13A_MOUSE	0.00174	0.00202	0.00114	0.00132	0.00752	0.00148	0.00672	0.00875	0.01027	0.00797	0.00670	0.01008
Q8K3J1	NDUS8_MOUSE	0.03365	0.05372	0.01038	0.01040	0.01708	0.03442	0.01060	0.01537	0.01203	0.00818	0.00894	0.01000
Q8K411	PREP_MOUSE	0.01180	0.00705	0.01193	0.01167	0.01464	0.00669	0.00670	0.00448	0.00545	0.00471	0.00527	0.00484
Q8K440	ABC8B_MOUSE	0.00000	0.00000	0.00000	0.00000	0.00000	0.00000	0.00360	0.00358	0.00155	0.00384	0.00492	0.00467
Q8K4F5	ABHDB_MOUSE	0.00000	0.00000	0.00000	0.00000	0.00000	0.00000	0.00181	0.00312	0.00401	0.00265	0.00235	0.00310
Q8K4G1	LTBP4_MOUSE	0.00008	0.00196	0.00163	0.00000	0.00055	0.00137	0.00000	0.00267	0.00577	0.00000	0.00356	0.00000
Q8K4G5	ABLM1_MOUSE	0.00832	0.00556	0.00996	0.00666	0.00702	0.00428	0.01371	0.01186	0.01794	0.00910	0.00658	0.01190
Q8K4H1	KFA_MOUSE	0.00000	0.00000	0.00000	0.00000	0.00000	0.00000	0.00000	0.00000	0.00000	0.00000	0.00000	0.00000
Q8K4K6	PANK1_MOUSE	0.00000	0.00000	0.00000	0.00000	0.00000	0.00000	0.00000	0.00000	0.00000	0.00000	0.00000	0.00000
Q8K4L3	SVIL_MOUSE	0.01090	0.02087	0.00786	0.01213	0.00875	0.01156	0.01774	0.02496	0.02812	0.02671	0.02870	0.02700
Q8K4Q8	COL12_MOUSE	0.00000	0.00000	0.00000	0.00000	0.00000	0.00000	0.00797	0.00313	0.02677	0.01932	0.01962	0.01171
Q8K4X7	PLCD_MOUSE	0.00000	0.00000	0.00000	0.00000	0.00000	0.00000	0.00182	0.00239	0.00502	0.00338	0.00316	0.00433
Q8K4Z3	NNRE_MOUSE	0.02874	0.02174	0.03278	0.01144	0.02593	0.01556	0.05850	0.06969	0.04612	0.04666	0.03426	0.05662
Q8K4Z5	SF3A1_MOUSE	0.00000	0.00000	0.00000	0.00000	0.00000	0.00000	0.01781	0.00899	0.01103	0.00718	0.00902	0.00923
Q8K558	TRML1_MOUSE	0.00000	0.00000	0.00000	0.00000	0.00000	0.00000	0.00000	0.00000	0.00096	0.00123	0.00000	0.00000
Q8K5B2	MCFD2_MOUSE	0.00000	0.00000	0.00000	0.00000	0.00000	0.00000	0.00000	0.00181	0.00948	0.00833	0.01261	0.00731
Q8N7N5	DCAF8_MOUSE	0.00000	0.00000	0.00000	0.00000	0.00000	0.00000	0.00000	0.00468	0.00096	0.00152	0.00000	0.00000
Q8QZR5	ALAT1_MOUSE	0.00348	0.00685	0.00611	0.00324	0.00336	0.00388	0.00099	0.00929	0.00422	0.00205	0.00233	0.00590
Q8QZS1	HIBCH_MOUSE	0.01804	0.03007	0.04480	0.03367	0.07287	0.05793	0.02050	0.01744	0.01610	0.01077	0.01732	0.01453
Q8QZT1	THIL_MOUSE	0.58055	0.53354	0.52899	0.55167	0.50157	0.47484	0.04115	0.06156	0.05865	0.05173	0.03487	0.05494
Q8QZW3	F151A_MOUSE	0.00000	0.00000	0.00000	0.00000	0.00000	0.00000	0.00000	0.00258	0.00414	0.00711	0.00901	0.00474
Q8QZY1	EIF3L_MOUSE	0.01211	0.01108	0.01285	0.00628	0.01095	0.00384	0.02518	0.02031	0.01850	0.02128	0.02145	0.02270
Q8QZY2	GLCTK_MOUSE	0.00000	0.00000	0.00000	0.00000	0.00000	0.00000	0.00742	0.00000	0.00885	0.00769	0.00174	0.00000
Q8QZY6	TSN14_MOUSE	0.00000	0.00000	0.00000	0.00000	0.00000	0.00000	0.00176	0.00043	0.00138	0.00211	0.00000	0.00143
Q8QZY9	SF3B4_MOUSE	0.00000	0.00000	0.00000	0.00000	0.00000	0.00000	0.00097	0.00361	0.00497	0.00372	0.00605	0.00503
Q8R001	MARE2_MOUSE	0.00000	0.00000	0.00000	0.00000	0.00000	0.00000	0.00552	0.00705	0.01012	0.00558	0.00612	0.00701
Q8R010	AIMP2_MOUSE	0.00168	0.00272	0.00516	0.00343	0.00386	0.00217	0.00046	0.00092	0.00189	0.00234	0.00207	0.00157

Q8R016	BLMH_MOUSE	0.00545	0.00473	0.00432	0.00630	0.00553	0.00662	0.01720	0.01342	0.01381	0.01828	0.01572	0.01546
Q8R034	APC13_MOUSE	0.00000	0.00000	0.00000	0.00000	0.00000	0.00000	0.00834	0.00265	0.00000	0.00265	0.00000	0.00160
Q8R050	ERF3A_MOUSE	0.00244	0.00388	0.00166	0.00216	0.00201	0.00503	0.00584	0.00516	0.00523	0.00413	0.00663	0.00322
Q8R059	GALE_MOUSE	0.00000	0.00000	0.00000	0.00000	0.00000	0.00000	0.00207	0.00088	0.00165	0.00445	0.00000	0.00310
Q8R081	HNRPL_MOUSE	0.00792	0.01179	0.00806	0.00910	0.00791	0.00785	0.03661	0.02779	0.04764	0.03475	0.04605	0.03756
Q8R086	SUOX_MOUSE	0.00591	0.00174	0.00752	0.00171	0.00499	0.00240	0.00360	0.00225	0.00381	0.00202	0.00125	0.00456
Q8R0F8	FAHD1_MOUSE	0.00097	0.00139	0.00132	0.00205	0.00169	0.00468	0.00110	0.00121	0.00108	0.00162	0.00000	0.00287
Q8R0F9	S14L4_MOUSE	0.00000	0.00000	0.00000	0.00000	0.00000	0.00000	0.00944	0.02337	0.02810	0.02229	0.01986	0.02051
Q8R0G9	NU133_MOUSE	0.00000	0.00000	0.00000	0.00000	0.00000	0.00000	0.00023	0.00234	0.00000	0.00000	0.00000	0.00000
Q8R0I0	ACE2_MOUSE	0.00000	0.00000	0.00000	0.00000	0.00000	0.00000	0.00000	0.00000	0.00000	0.00000	0.00000	0.00000
Q8R0N6	HOT_MOUSE	0.00908	0.00583	0.00405	0.00344	0.00928	0.00445	0.00031	0.00025	0.00017	0.00010	0.00011	0.00011
Q8R0W0	EPIPL_MOUSE	0.00000	0.00000	0.00000	0.00000	0.00000	0.00000	0.00758	0.01251	0.01081	0.01236	0.00810	0.01064
Q8R0X7	SGPL1_MOUSE	0.00000	0.00000	0.00000	0.00000	0.00000	0.00000	0.00559	0.00486	0.00826	0.00596	0.00932	0.00577
Q8R0Y6	AL1L1_MOUSE	0.00000	0.00000	0.00000	0.00000	0.00000	0.00000	0.01025	0.01105	0.01606	0.00865	0.01399	0.01034
Q8R0Y8	S2542_MOUSE	0.00000	0.00000	0.00000	0.00000	0.00000	0.00000	0.00000	0.00000	0.00000	0.00000	0.00000	0.00000
Q8R123	FAD1_MOUSE	0.00000	0.00000	0.00000	0.00000	0.00000	0.00000	0.00000	0.00000	0.00000	0.00000	0.00000	0.00000
Q8R138	TM119_MOUSE	0.00000	0.00000	0.00000	0.00000	0.00000	0.00000	0.00393	0.00314	0.00315	0.00265	0.00436	0.00331
Q8R146	APEH_MOUSE	0.00300	0.00274	0.00225	0.00347	0.00185	0.00059	0.01741	0.01516	0.01778	0.01847	0.02391	0.01575
Q8R164	BPHL_MOUSE	0.03121	0.01943	0.02784	0.02478	0.02932	0.02258	0.01340	0.01335	0.01330	0.00949	0.01481	0.01015
Q8R180	ERO1A_MOUSE	0.00000	0.00000	0.00000	0.00000	0.00000	0.00000	0.00836	0.00841	0.00991	0.01069	0.00921	0.00890
Q8R1A4	DOCK7_MOUSE	0.00000	0.00000	0.00000	0.00000	0.00000	0.00000	0.00534	0.00462	0.00558	0.00477	0.00567	0.00651
Q8R1B4	EIF3C_MOUSE	0.00328	0.00417	0.00595	0.00477	0.00361	0.00313	0.01167	0.01562	0.01484	0.01458	0.01306	0.01476
Q8R1F1	NIBA2_MOUSE	0.00000	0.00000	0.00000	0.00000	0.00000	0.00000	0.19018	0.29556	0.23390	0.22891	0.24148	0.24045
Q8R1G2	CMBL_MOUSE	0.00362	0.00123	0.00226	0.00396	0.00391	0.00008	0.00000	0.00366	0.00556	0.00309	0.00243	0.00163
Q8R1G6	PDLI2_MOUSE	0.00000	0.00000	0.00000	0.00000	0.00000	0.00000	0.05099	0.05532	0.04288	0.04661	0.04610	0.05560
Q8R1H0	HOP_MOUSE	0.00000	0.00000	0.00000	0.00000	0.00000	0.00000	0.05296	0.13111	0.07165	0.03848	0.09199	0.12313
Q8R1I1	QCR9_MOUSE	0.00000	0.00000	0.00000	0.00000	0.00000	0.00000	0.03189	0.04791	0.02874	0.03234	0.03593	0.03339
Q8R1N4	NUDC3_MOUSE	0.00000	0.00000	0.00000	0.00000	0.00000	0.00000	0.00589	0.00327	0.00543	0.00486	0.00651	0.00357
Q8R1Q8	DC1L1_MOUSE	0.00781	0.00550	0.00638	0.00818	0.00665	0.00652	0.00991	0.01184	0.01503	0.01273	0.01219	0.01124
Q8R1Q9	RBSK_MOUSE	0.00000	0.00000	0.00000	0.00000	0.00000	0.00000	0.00000	0.00886	0.02140	0.02184	0.02369	0.02339
Q8R1T4	S35A3_MOUSE	0.00000	0.00000	0.00000	0.00000	0.00000	0.00000	0.00000	0.00000	0.00000	0.00000	0.00000	0.00000
Q8R1V4	TMED4_MOUSE	0.00000	0.00000	0.00000	0.00000	0.00000	0.00000	0.00000	0.00518	0.00044	0.00385	0.00200	0.00469
Q8R242	DIAC_MOUSE	0.00000	0.00000	0.00000	0.00000	0.00000	0.00000	0.00000	0.00217	0.00230	0.00040	0.00132	0.00085
Q8R2Q4	RRF2M_MOUSE	0.00706	0.00541	0.00697	0.01054	0.00731	0.00758	0.00000	0.00000	0.00000	0.00000	0.00000	0.00000

Q8R2Q8	BST2_MOUSE	0.00000	0.00000	0.00000	0.00000	0.00000	0.00000	0.00000	0.01246	0.00768	0.00658	0.00452	0.00401
Q8R2U0	SEH1_MOUSE	0.00000	0.00000	0.00000	0.00000	0.00000	0.00000	0.00558	0.00509	0.00652	0.00569	0.00877	0.00623
Q8R2U6	NUDT4_MOUSE	0.00000	0.00000	0.00000	0.00000	0.00000	0.00000	0.00000	0.00000	0.00000	0.00197	0.00059	0.00000
Q8R2Y2	MUC18_MOUSE	0.00000	0.00000	0.00000	0.00000	0.00000	0.00000	0.04750	0.04230	0.05112	0.05002	0.05939	0.05002
Q8R2Y8	PTH2_MOUSE	0.00284	0.00114	0.00149	0.00170	0.00221	0.00107	0.01037	0.00527	0.00994	0.00837	0.01023	0.00731
Q8R317	UBQL1_MOUSE	0.00000	0.00000	0.00000	0.00000	0.00000	0.00000	0.01451	0.01741	0.01717	0.01328	0.01812	0.01935
Q8R326	PSPC1_MOUSE	0.00000	0.00000	0.00000	0.00000	0.00000	0.00000	0.01071	0.00351	0.00918	0.00804	0.00894	0.00963
Q8R332	NUP58_MOUSE	0.00000	0.00000	0.00000	0.00000	0.00000	0.00000	0.00271	0.00035	0.00638	0.00640	0.00766	0.00692
Q8R361	RFIP5_MOUSE	0.00000	0.00000	0.00000	0.00000	0.00000	0.00000	0.01587	0.04284	0.00000	0.00000	0.00000	0.04293
Q8R3B1	PLCD1_MOUSE	0.00279	0.00283	0.00191	0.00371	0.00273	0.00398	0.00850	0.00715	0.01102	0.00672	0.01098	0.01089
Q8R3D1	TBC13_MOUSE	0.00000	0.00000	0.00000	0.00000	0.00000	0.00000	0.00000	0.00000	0.00000	0.00000	0.00000	0.00000
Q8R3E3	WIPI1_MOUSE	0.00000	0.00000	0.00000	0.00000	0.00000	0.00000	0.00000	0.00337	0.00555	0.00360	0.00196	0.00438
Q8R3G1	PP1R8_MOUSE	0.00000	0.00000	0.00000	0.00000	0.00000	0.00000	0.00000	0.00000	0.00000	0.00098	0.00000	0.00076
Q8R3G9	TSN8_MOUSE	0.00000	0.00000	0.00000	0.00000	0.00000	0.00000	0.01991	0.03093	0.04529	0.03247	0.01654	0.03070
Q8R3H7	HS2ST_MOUSE	0.00000	0.00000	0.00000	0.00000	0.00000	0.00000	0.00000	0.00061	0.00078	0.00109	0.00045	0.00034
Q8R3P0	ACY2_MOUSE	0.00000	0.00000	0.00000	0.00000	0.00000	0.00000	0.00029	0.00000	0.00000	0.00022	0.00014	0.00000
Q8R3S6	EXOC1_MOUSE	0.00000	0.00000	0.00000	0.00000	0.00000	0.00000	0.00270	0.00000	0.00315	0.00196	0.00000	0.00400
Q8R3T5	STXB6_MOUSE	0.00000	0.00000	0.00000	0.00000	0.00000	0.00000	0.00000	0.00135	0.00032	0.00039	0.00000	0.00060
Q8R404	MIC13_MOUSE	0.00000	0.00000	0.00000	0.00000	0.00000	0.00000	0.00261	0.02536	0.01340	0.00651	0.01087	0.02406
Q8R411	MYCT1_MOUSE	0.00000	0.00000	0.00000	0.00000	0.00000	0.00000	0.00000	0.00326	0.00000	0.00000	0.00000	0.00000
Q8R420	ABCA3_MOUSE	0.00000	0.00000	0.00000	0.00000	0.00000	0.00000	0.00437	0.00860	0.00619	0.00682	0.00222	0.00401
Q8R429	AT2A1_MOUSE	0.00000	0.00000	0.00000	0.00000	0.00000	0.00000	0.05181	0.00284	0.00048	0.00081	0.00000	0.00017
Q8R4E4	MYOZ3_MOUSE	0.00000	0.00000	0.00000	0.00000	0.00000	0.00000	0.00335	0.00000	0.00000	0.00000	0.00000	0.00000
Q8R4H2	ARHGC_MOUSE	0.00000	0.00000	0.00000	0.00000	0.00000	0.00000	0.00624	0.00759	0.00960	0.00786	0.00850	0.00757
Q8R4K2	IRAK4_MOUSE	0.00000	0.00000	0.00000	0.00000	0.00000	0.00000	0.00884	0.00000	0.00764	0.00427	0.00315	0.00126
Q8R4N0	CLYBL_MOUSE	0.00865	0.01173	0.01509	0.01509	0.01681	0.00913	0.00000	0.00000	0.00000	0.00111	0.00000	0.00000
Q8R4S0	PP14C_MOUSE	0.00078	0.00078	0.00035	0.00050	0.00050	0.00000	0.00000	0.00000	0.00165	0.00149	0.00102	0.00000
Q8R4Y4	STAB1_MOUSE	0.00000	0.00000	0.00000	0.00000	0.00000	0.00000	0.00000	0.00085	0.00181	0.00066	0.00073	0.00044
Q8R502	LRC8C_MOUSE	0.00000	0.00000	0.00000	0.00000	0.00000	0.00000	0.00780	0.01867	0.01415	0.01132	0.00811	0.01092
Q8R519	ACMSD_MOUSE	0.00000	0.00000	0.00000	0.00000	0.00000	0.00000	0.00009	0.00010	0.00075	0.00032	0.00064	0.00053
Q8R550	SH3K1_MOUSE	0.00000	0.00000	0.00000	0.00000	0.00000	0.00000	0.00370	0.00275	0.00551	0.00755	0.00490	0.00810
Q8R570	SNP47_MOUSE	0.00000	0.00000	0.00000	0.00000	0.00000	0.00000	0.00215	0.00122	0.00197	0.00275	0.00112	0.00291
Q8R574	KPRB_MOUSE	0.00000	0.00000	0.00000	0.00000	0.00000	0.00000	0.00760	0.00305	0.00776	0.00576	0.00612	0.00910
Q8R5A3	AB1IP_MOUSE	0.00000	0.00000	0.00000	0.00000	0.00000	0.00000	0.00138	0.00224	0.00429	0.00501	0.00291	0.00456

Q8R5C5	ACTY_MOUSE	0.00000	0.00000	0.00000	0.00000	0.00000	0.00000	0.01679	0.02045	0.01441	0.01026	0.01894	0.01385
Q8R5G7	ARAP3_MOUSE	0.00000	0.00000	0.00000	0.00000	0.00000	0.00000	0.00000	0.00310	0.00427	0.00413	0.00595	0.00454
Q8R5H1	UBP15_MOUSE	0.00795	0.00093	0.00246	0.00392	0.00149	0.00151	0.00390	0.00321	0.00395	0.00327	0.00422	0.00359
Q8R5I6	GSTM4_MOUSE	0.00112	0.00103	0.00109	0.00167	0.00188	0.00059	0.00110	0.00365	0.00000	0.00200	0.00000	0.00322
Q8R5J9	PRAF3_MOUSE	0.00000	0.00000	0.00000	0.00000	0.00000	0.00000	0.02272	0.03429	0.00807	0.01361	0.00421	0.02162
Q8R5M0	GIPC3_MOUSE	0.00000	0.00000	0.00000	0.00000	0.00000	0.00000	0.00000	0.00000	0.00120	0.00063	0.00096	0.00000
Q8R5M8	CADM1_MOUSE	0.00000	0.00000	0.00000	0.00000	0.00000	0.00000	0.01304	0.01600	0.01453	0.01813	0.01657	0.02473
Q8VBT0	TMX1_MOUSE	0.00000	0.00000	0.00000	0.00000	0.00000	0.00000	0.01751	0.00779	0.00485	0.00483	0.00872	0.00501
Q8VBT9	ASPC1_MOUSE	0.00000	0.00000	0.00000	0.00000	0.00000	0.00000	0.00094	0.00096	0.00080	0.00179	0.00128	0.00186
Q8VBV7	CSN8_MOUSE	0.00000	0.00000	0.00000	0.00000	0.00000	0.00000	0.01691	0.01205	0.01196	0.01742	0.01835	0.01829
Q8VBW6	ULA1_MOUSE	0.00528	0.00924	0.00599	0.00628	0.00496	0.00417	0.00699	0.00804	0.01344	0.01210	0.01560	0.01213
Q8VBZ3	CLPT1_MOUSE	0.01048	0.01745	0.02024	0.00491	0.01126	0.00302	0.01095	0.00943	0.01295	0.01053	0.02034	0.01340
Q8VC12	HUTU_MOUSE	0.00000	0.00000	0.00000	0.00000	0.00000	0.00000	0.01583	0.01068	0.01653	0.01421	0.01624	0.01477
Q8VC28	AK1CD_MOUSE	0.00000	0.00000	0.00000	0.00000	0.00000	0.00000	0.00000	0.00090	0.00318	0.00151	0.00152	0.00146
Q8VC30	TKFC_MOUSE	0.00000	0.00000	0.00000	0.00000	0.00000	0.00000	0.00002	0.00012	0.00038	0.00029	0.00011	0.00006
Q8VC60	GLB1L_MOUSE	0.00000	0.00000	0.00000	0.00000	0.00000	0.00000	0.00000	0.00000	0.00198	0.00000	0.00112	0.00000
Q8VC69	S22A6_MOUSE	0.00000	0.00000	0.00000	0.00000	0.00000	0.00000	0.00000	0.00000	0.00000	0.00000	0.00000	0.00000
Q8VCA8	SCRN2_MOUSE	0.00109	0.00246	0.00072	0.00109	0.00119	0.00149	0.00423	0.00246	0.00643	0.00578	0.00788	0.00601
Q8VCC1	PGDH_MOUSE	0.00000	0.00000	0.00000	0.00000	0.00000	0.00000	0.01401	0.02607	0.01605	0.01465	0.01303	0.01689
Q8VCC2	EST1_MOUSE	0.00000	0.00000	0.00000	0.00000	0.00000	0.00000	0.00000	0.00252	0.00184	0.00271	0.00302	0.00206
Q8VCC9	SPON1_MOUSE	0.00000	0.00000	0.00000	0.00000	0.00000	0.00000	0.00631	0.01099	0.01010	0.00709	0.00843	0.00997
Q8VCE2	GPN1_MOUSE	0.00000	0.00000	0.00000	0.00000	0.00000	0.00000	0.00000	0.00195	0.00000	0.00000	0.00000	0.00000
Q8VCF0	MAVS_MOUSE	0.00656	0.00505	0.00643	0.00676	0.00697	0.00561	0.01213	0.00253	0.00598	0.00402	0.00305	0.00202
Q8VCH0	THIKB_MOUSE	0.00000	0.00000	0.00000	0.00000	0.00000	0.00000	0.00000	0.00000	0.00000	0.00000	0.00000	0.00000
Q8VCH7	RDH10_MOUSE	0.00000	0.00000	0.00000	0.00000	0.00000	0.00000	0.00000	0.00038	0.00063	0.00052	0.00042	0.00080
Q8VCH8	UBXN4_MOUSE	0.00000	0.00000	0.00000	0.00000	0.00000	0.00000	0.00689	0.00463	0.00262	0.00369	0.00240	0.00211
Q8VCI0	PLBL1_MOUSE	0.00000	0.00000	0.00000	0.00000	0.00000	0.00000	0.00490	0.00478	0.00626	0.00610	0.00687	0.00737
Q8VCI5	PEX19_MOUSE	0.00993	0.00705	0.00770	0.00493	0.01015	0.00391	0.00000	0.00267	0.00194	0.00178	0.00337	0.00117
Q8VCM7	FIBG_MOUSE	0.01340	0.00867	0.00962	0.02513	0.01564	0.01223	0.09917	0.03407	0.04354	0.05986	0.09736	0.02647
Q8VCM8	NCLN_MOUSE	0.00000	0.00000	0.00000	0.00000	0.00000	0.00000	0.00552	0.00351	0.00573	0.00508	0.00487	0.00451
Q8VCN5	CGL_MOUSE	0.00000	0.00000	0.00000	0.00000	0.00000	0.00000	0.00051	0.00000	0.00195	0.00064	0.00116	0.00059
Q8VCN6	CD99_MOUSE	0.00000	0.00000	0.00000	0.00000	0.00000	0.00000	0.00607	0.00000	0.01818	0.00611	0.01627	0.00516
Q8VCN9	TBCC_MOUSE	0.00000	0.00000	0.00000	0.00000	0.00000	0.00000	0.00079	0.00166	0.00190	0.00165	0.00211	0.00248
Q8VCP9	CLC14_MOUSE	0.00000	0.00000	0.00000	0.00000	0.00000	0.00000	0.01160	0.02348	0.03128	0.02385	0.03945	0.02519

Q8VCR7	ABHEB_MOUSE	0.00000	0.00000	0.00000	0.00000	0.00000	0.00000	0.00826	0.01194	0.01072	0.00869	0.01366	0.00973
Q8VCT3	AMPB_MOUSE	0.00212	0.00394	0.00256	0.00223	0.00298	0.00382	0.02020	0.02904	0.03042	0.02753	0.02269	0.02342
Q8VCT4	EST1D_MOUSE	0.00246	0.00385	0.00507	0.00333	0.00494	0.00249	0.18808	0.29539	0.15218	0.17731	0.16579	0.23362
Q8VCV1	AB17C_MOUSE	0.00000	0.00000	0.00000	0.00000	0.00000	0.00000	0.00000	0.00079	0.00104	0.00045	0.00000	0.00000
Q8VCW8	ACSF2_MOUSE	0.03210	0.03060	0.01817	0.01451	0.02769	0.02305	0.01057	0.01627	0.01180	0.01321	0.01842	0.01890
Q8VD04	GRAP1_MOUSE	0.00000	0.00000	0.00000	0.00000	0.00000	0.00000	0.00000	0.00000	0.00000	0.00000	0.00000	0.00000
Q8VD75	HIP1_MOUSE	0.00000	0.00000	0.00000	0.00000	0.00000	0.00000	0.00553	0.00616	0.01054	0.00749	0.01154	0.00894
Q8VDC0	SYLM_MOUSE	0.00000	0.00000	0.00000	0.00000	0.00000	0.00000	0.00000	0.00000	0.00000	0.00000	0.00000	0.00000
Q8VDC1	FYCO1_MOUSE	0.00514	0.00722	0.00565	0.00426	0.00513	0.00382	0.00183	0.00238	0.00629	0.00417	0.00452	0.00462
Q8VDD5	MYH9_MOUSE	0.05910	0.05741	0.04268	0.05140	0.05735	0.05030	0.23677	0.21928	0.24273	0.25988	0.21960	0.24585
Q8VDF3	DAPK2_MOUSE	0.00000	0.00000	0.00000	0.00000	0.00000	0.00000	0.00446	0.00175	0.00492	0.00483	0.00659	0.00466
Q8VDG5	PPCS_MOUSE	0.00000	0.00000	0.00000	0.00000	0.00000	0.00000	0.00000	0.00246	0.00291	0.00208	0.00000	0.00174
Q8VDJ3	VIGLN_MOUSE	0.00658	0.00560	0.00618	0.00616	0.00515	0.00541	0.02438	0.02628	0.02878	0.02524	0.02728	0.02836
Q8VDK1	NIT1_MOUSE	0.00201	0.00188	0.00304	0.00186	0.00333	0.00249	0.00964	0.00862	0.01351	0.01030	0.01078	0.01021
Q8VDL4	ADPGK_MOUSE	0.00000	0.00000	0.00000	0.00000	0.00000	0.00000	0.00913	0.01332	0.01223	0.01054	0.01571	0.01364
Q8VDM4	PSMD2_MOUSE	0.00217	0.00323	0.00357	0.00545	0.00247	0.00206	0.03549	0.04663	0.06318	0.05436	0.05662	0.05307
Q8VDM6	HNRL1_MOUSE	0.00000	0.00000	0.00000	0.00000	0.00000	0.00000	0.01875	0.02431	0.03009	0.02284	0.00680	0.03208
Q8VDN2	AT1A1_MOUSE	0.15344	0.18349	0.11755	0.11996	0.15091	0.18714	0.05956	0.07276	0.05669	0.06356	0.05128	0.06934
Q8VDP3	MICA1_MOUSE	0.00000	0.00000	0.00000	0.00000	0.00000	0.00000	0.00635	0.00343	0.00818	0.00444	0.00575	0.00460
Q8VDP4	CCAR2_MOUSE	0.00000	0.00000	0.00000	0.00000	0.00000	0.00000	0.01023	0.00432	0.01007	0.00683	0.00466	0.00899
Q8VDP6	CDIPT_MOUSE	0.00000	0.00000	0.00000	0.00000	0.00000	0.00000	0.00409	0.01246	0.00531	0.00546	0.00000	0.00542
Q8VDQ1	PTGR2_MOUSE	0.02224	0.00749	0.01585	0.01134	0.01609	0.01052	0.01068	0.01098	0.01527	0.00917	0.01316	0.01036
Q8VDQ8	SIR2_MOUSE	0.00000	0.00000	0.00000	0.00000	0.00000	0.00000	0.00701	0.00675	0.00771	0.00822	0.00475	0.00823
Q8VDR5	TM267_MOUSE	0.00000	0.00000	0.00000	0.00000	0.00000	0.00000	0.00139	0.00000	0.00269	0.00000	0.00000	0.00000
Q8VDR9	DOCK6_MOUSE	0.00000	0.00000	0.00000	0.00000	0.00000	0.00000	0.00110	0.00000	0.00178	0.00148	0.00067	0.00113
Q8VDT1	SC5A9_MOUSE	0.00000	0.00000	0.00000	0.00000	0.00000	0.00000	0.00000	0.00000	0.00000	0.00000	0.00000	0.00000
Q8VDW0	DX39A_MOUSE	0.00000	0.00000	0.00000	0.00000	0.00000	0.00000	0.00000	0.00157	0.00049	0.00064	0.00225	0.00195
Q8VE22	RT23_MOUSE	0.00296	0.00276	0.00371	0.00290	0.00230	0.00269	0.00000	0.00000	0.00000	0.00000	0.00000	0.00000
Q8VE37	RCC1_MOUSE	0.00000	0.00000	0.00000	0.00000	0.00000	0.00000	0.00825	0.00455	0.00508	0.00460	0.00405	0.00500
Q8VE38	OXND1_MOUSE	0.00190	0.00445	0.00590	0.00542	0.00803	0.00585	0.00000	0.00000	0.00086	0.00000	0.00000	0.00035
Q8VE47	UBA5_MOUSE	0.00000	0.00000	0.00000	0.00000	0.00000	0.00000	0.00683	0.01146	0.01077	0.01163	0.01733	0.01226
Q8VE62	PAIP1_MOUSE	0.00000	0.00000	0.00000	0.00000	0.00000	0.00000	0.00000	0.00512	0.00227	0.00348	0.00152	0.00358
Q8VE70	PDC10_MOUSE	0.00000	0.00000	0.00000	0.00000	0.00000	0.00000	0.00811	0.00518	0.01032	0.01214	0.01008	0.01302
Q8VE88	F1142_MOUSE	0.00000	0.00000	0.00000	0.00000	0.00000	0.00000	0.00282	0.00225	0.00298	0.00262	0.00366	0.00323

Q8VE95	CH082_MOUSE	0.00000	0.00000	0.00000	0.00000	0.00000	0.00000	0.00122	0.00067	0.00248	0.00187	0.01124	0.00229
Q8VE97	SRSF4_MOUSE	0.00000	0.00000	0.00000	0.00000	0.00000	0.00000	0.00167	0.00228	0.00536	0.00437	0.00281	0.00200
Q8VEB1	GRK5_MOUSE	0.00000	0.00000	0.00000	0.00000	0.00000	0.00000	0.00157	0.00131	0.00000	0.00000	0.00359	0.00000
Q8VEB4	PAG15_MOUSE	0.00000	0.00000	0.00000	0.00000	0.00000	0.00000	0.00225	0.00683	0.00373	0.00379	0.00266	0.00232
Q8VED9	LEGL_MOUSE	0.00000	0.00000	0.00000	0.00000	0.00000	0.00000	0.00172	0.00322	0.00515	0.00243	0.00281	0.00288
Q8VEE1	LMCD1_MOUSE	0.00000	0.00000	0.00000	0.00000	0.00000	0.00000	0.00801	0.00776	0.00195	0.00705	0.00000	0.00364
Q8VEE4	RFA1_MOUSE	0.00000	0.00000	0.00000	0.00000	0.00000	0.00000	0.00342	0.00000	0.00205	0.00366	0.00000	0.00684
Q8VEH3	ARL8A_MOUSE	0.00000	0.00000	0.00000	0.00000	0.00000	0.00000	0.02374	0.01421	0.01389	0.01491	0.01727	0.00951
Q8VEK0	CC50A_MOUSE	0.00000	0.00000	0.00000	0.00000	0.00000	0.00000	0.00000	0.00623	0.00604	0.00681	0.00295	0.00405
Q8VEK3	HNRPU_MOUSE	0.00000	0.00000	0.00000	0.00000	0.00000	0.00000	0.06054	0.08080	0.06741	0.07380	0.04695	0.08350
Q8VEM8	MPCP_MOUSE	0.16449	0.25013	0.30286	0.21600	0.18786	0.21665	0.11739	0.11493	0.12931	0.12409	0.11802	0.11289
Q8VH51	RBM39_MOUSE	0.00000	0.00000	0.00000	0.00000	0.00000	0.00000	0.01488	0.01489	0.01139	0.01351	0.01392	0.01554
Q8VHE0	SEC63_MOUSE	0.00000	0.00000	0.00000	0.00000	0.00000	0.00000	0.00000	0.00305	0.00141	0.00074	0.00268	0.00057
Q8VHG0	FMO4_MOUSE	0.00000	0.00000	0.00000	0.00000	0.00000	0.00000	0.00000	0.00000	0.00000	0.00000	0.00000	0.00022
Q8VHK1	CSKI2_MOUSE	0.00000	0.00000	0.00000	0.00000	0.00000	0.00000	0.00000	0.00098	0.00261	0.00134	0.00000	0.00133
Q8VHL1	SETD7_MOUSE	0.00000	0.00000	0.00000	0.00000	0.00000	0.00000	0.00331	0.00464	0.00621	0.00541	0.00493	0.00722
Q8VHR5	P66B_MOUSE	0.00000	0.00000	0.00000	0.00000	0.00000	0.00000	0.00000	0.00000	0.00706	0.00632	0.00321	0.00301
Q8VHX6	FLNC_MOUSE	0.09825	0.10894	0.11036	0.08097	0.08557	0.06241	0.10820	0.01390	0.01362	0.01238	0.01034	0.00749
Q8VHY0	CSPG4_MOUSE	0.00000	0.00000	0.00000	0.00000	0.00000	0.00000	0.00972	0.00667	0.02008	0.00765	0.02224	0.01272
Q8VI36	PAXI_MOUSE	0.00000	0.00000	0.00000	0.00000	0.00000	0.00000	0.01990	0.02442	0.02842	0.02021	0.02012	0.02571
Q8VI47	MRP2_MOUSE	0.00000	0.00000	0.00000	0.00000	0.00000	0.00000	0.00000	0.00000	0.00000	0.00000	0.00000	0.00042
Q8VIJ6	SFPQ_MOUSE	0.00322	0.00447	0.00217	0.00233	0.00306	0.00309	0.08487	0.04160	0.06876	0.05464	0.09109	0.06658
Q8VIM4	BSND_MOUSE	0.00000	0.00000	0.00000	0.00000	0.00000	0.00000	0.00000	0.00000	0.00000	0.00000	0.00000	0.00000
Q8WTY4	CPIN1_MOUSE	0.00000	0.00000	0.00056	0.00018	0.00007	0.00000	0.00000	0.00000	0.00113	0.00177	0.00080	0.00075
Q91V01	MBOA5_MOUSE	0.00000	0.00000	0.00000	0.00000	0.00000	0.00000	0.00996	0.00829	0.00624	0.00785	0.00265	0.00621
Q91V04	TRAM1_MOUSE	0.00000	0.00000	0.00000	0.00000	0.00000	0.00000	0.00330	0.00420	0.00026	0.00111	0.00000	0.00261
Q91V12	BACH_MOUSE	0.00000	0.00000	0.00000	0.00000	0.00000	0.00000	0.02897	0.03584	0.03645	0.04043	0.03210	0.03595
Q91V16	ETFR1_MOUSE	0.00000	0.00000	0.00000	0.00000	0.00000	0.00000	0.00086	0.00000	0.00000	0.00000	0.00000	0.00029
Q91V41	RAB14_MOUSE	0.01923	0.01742	0.01627	0.01419	0.02657	0.01867	0.03262	0.03605	0.03715	0.03630	0.02215	0.04446
Q91V61	SFXN3_MOUSE	0.00208	0.00362	0.00079	0.00093	0.00139	0.00150	0.02200	0.02508	0.02513	0.02803	0.02140	0.03067
Q91V64	ISOC1_MOUSE	0.00387	0.00332	0.00413	0.00262	0.00297	0.00311	0.00535	0.01089	0.01642	0.01524	0.01411	0.01455
Q91V76	CK054_MOUSE	0.00000	0.00000	0.00000	0.00000	0.00000	0.00000	0.00007	0.00013	0.00013	0.00007	0.00023	0.00014
Q91V88	NPNT_MOUSE	0.00000	0.00000	0.00000	0.00000	0.00000	0.00000	0.11516	0.09540	0.13217	0.08089	0.17160	0.15243
Q91V92	ACLY_MOUSE	0.00000	0.00000	0.00000	0.00000	0.00000	0.00000	0.07777	0.04418	0.06914	0.04277	0.04577	0.04877

Q91VA0	ACSM1_MOUSE	0.00000	0.00000	0.00000	0.00000	0.00000	0.00000	0.00001	0.00000	0.00000	0.00000	0.00032	0.00005
Q91VA6	PDIP2_MOUSE	0.00138	0.00054	0.00095	0.00044	0.00087	0.00055	0.00008	0.00007	0.00006	0.00007	0.00000	0.00000
Q91VC3	IF4A3_MOUSE	0.00000	0.00000	0.00000	0.00000	0.00000	0.00000	0.04240	0.01218	0.02225	0.01417	0.01006	0.01270
Q91VC4	PLVAP_MOUSE	0.00000	0.00000	0.00000	0.00000	0.00000	0.00000	0.05812	0.05485	0.03932	0.03993	0.03809	0.05386
Q91VC7	PP14A_MOUSE	0.00000	0.00000	0.00000	0.00000	0.00000	0.00000	0.03226	0.07808	0.03647	0.05390	0.04390	0.04516
Q91VD9	NDUS1_MOUSE	0.52576	0.48272	0.42574	0.41013	0.47567	0.61510	0.04336	0.02476	0.03741	0.03167	0.03560	0.03150
Q91VE0	S27A4_MOUSE	0.00000	0.00000	0.00000	0.00000	0.00000	0.00000	0.00033	0.00000	0.00061	0.00061	0.00115	0.00056
Q91VF5	EMID1_MOUSE	0.00000	0.00000	0.00000	0.00000	0.00000	0.00000	0.00000	0.00051	0.00199	0.00042	0.00106	0.00000
Q91VH2	SNX9_MOUSE	0.00000	0.00000	0.00000	0.00000	0.00000	0.00000	0.00791	0.00923	0.01386	0.01167	0.00973	0.01141
Q91VH6	MEMO1_MOUSE	0.00108	0.00161	0.00144	0.00160	0.00158	0.00129	0.00219	0.00415	0.00533	0.00625	0.00267	0.00367
Q91VI7	RINI_MOUSE	0.02659	0.02349	0.01872	0.01649	0.01376	0.02748	0.02589	0.02941	0.04024	0.03746	0.04165	0.03136
Q91VJ2	CAVN3_MOUSE	0.00000	0.00000	0.00000	0.00000	0.00000	0.00000	0.00266	0.01173	0.00444	0.00452	0.00717	0.00464
Q91VJ4	STK38_MOUSE	0.00000	0.00000	0.00000	0.00000	0.00000	0.00000	0.00683	0.00713	0.00687	0.00548	0.00760	0.00702
Q91VK1	BZW2_MOUSE	0.00000	0.00000	0.00000	0.00000	0.00000	0.00000	0.00146	0.00000	0.00000	0.00000	0.00000	0.00074
Q91VM5	RMXL1_MOUSE	0.00000	0.00000	0.00000	0.00000	0.00000	0.00000	0.01040	0.01382	0.02305	0.01809	0.01530	0.01341
Q91VM9	IPYR2_MOUSE	0.04070	0.04090	0.02889	0.03399	0.03463	0.03734	0.02371	0.02879	0.02792	0.02082	0.02267	0.02501
Q91VN4	MIC25_MOUSE	0.00119	0.00411	0.00274	0.00174	0.00139	0.00234	0.02146	0.00389	0.00325	0.00282	0.00901	0.00366
Q91VR2	ATPG_MOUSE	0.04172	0.00222	0.06988	0.10618	0.02938	0.02729	0.04589	0.06051	0.04540	0.05574	0.04288	0.04898
Q91VR5	DDX1_MOUSE	0.00478	0.01229	0.00159	0.00089	0.00066	0.00128	0.01130	0.00831	0.01156	0.00816	0.01041	0.00925
Q91VS7	MGST1_MOUSE	0.00000	0.00000	0.00000	0.00000	0.00000	0.00000	0.00194	0.01495	0.00208	0.00811	0.01407	0.01453
Q91VT4	CBR4_MOUSE	0.00853	0.01228	0.01356	0.01290	0.00941	0.01314	0.00104	0.00321	0.00412	0.00252	0.00221	0.00153
Q91VW3	SH3L3_MOUSE	0.00000	0.00000	0.00000	0.00000	0.00000	0.00000	0.02104	0.02568	0.02272	0.02393	0.01983	0.02887
Q91VZ6	SMAP1_MOUSE	0.00000	0.00000	0.00000	0.00000	0.00000	0.00000	0.00968	0.00582	0.00311	0.00310	0.00125	0.00444
Q91W10	S39A8_MOUSE	0.00000	0.00000	0.00000	0.00000	0.00000	0.00000	0.00000	0.00000	0.00102	0.00000	0.00000	0.00000
Q91W43	GCSP_MOUSE	0.00000	0.00000	0.00000	0.00000	0.00000	0.00000	0.00000	0.00000	0.00000	0.00000	0.00000	0.00000
Q91W50	CSDE1_MOUSE	0.00000	0.00000	0.00000	0.00000	0.00000	0.00000	0.01220	0.01075	0.00943	0.01173	0.00816	0.01062
Q91W53	GOGA7_MOUSE	0.00000	0.00000	0.00000	0.00000	0.00000	0.00000	0.00089	0.00077	0.00256	0.00126	0.00128	0.00112
Q91W89	MA2C1_MOUSE	0.00058	0.00005	0.00135	0.00071	0.00031	0.00008	0.00000	0.00000	0.00479	0.00222	0.00365	0.00312
Q91W90	TXND5_MOUSE	0.00182	0.00149	0.00166	0.00151	0.00183	0.00102	0.02462	0.01822	0.03864	0.02612	0.03842	0.02942
Q91W92	BORG5_MOUSE	0.00000	0.00000	0.00000	0.00000	0.00000	0.00000	0.05867	0.05444	0.07211	0.05845	0.06458	0.05958
Q91WC0	SETD3_MOUSE	0.00000	0.00000	0.00000	0.00000	0.00000	0.00000	0.00168	0.00242	0.00179	0.00166	0.00185	0.00051
Q91WD5	NDUS2_MOUSE	0.00904	0.00975	0.01262	0.01308	0.02504	0.02120	0.03048	0.02208	0.02731	0.02079	0.02306	0.01746
Q91WG0	EST2C_MOUSE	0.00000	0.00000	0.00000	0.00000	0.00000	0.00000	0.00007	0.00012	0.00304	0.00140	0.00009	0.00032
Q91WJ8	FUBP1_MOUSE	0.00000	0.00000	0.00000	0.00000	0.00000	0.00000	0.00669	0.00888	0.01249	0.00788	0.00691	0.01028

Q91WK0	LRRF2_MOUSE	0.00000	0.00000	0.00000	0.00000	0.00000	0.00000	0.00000	0.00635	0.00194	0.00244	0.00145	0.00571
Q91WK2	EIF3H_MOUSE	0.00156	0.00182	0.00115	0.00030	0.00073	0.00151	0.00333	0.00377	0.00738	0.00476	0.00607	0.00355
Q91WK5	GCSH_MOUSE	0.00000	0.00000	0.00000	0.00000	0.00000	0.00000	0.00000	0.00355	0.00521	0.00176	0.00686	0.00513
Q91WM2	HDHD5_MOUSE	0.00000	0.00000	0.00000	0.00000	0.00000	0.00000	0.00000	0.00043	0.00056	0.00054	0.00000	0.00000
Q91WN1	DNJC9_MOUSE	0.00000	0.00000	0.00000	0.00000	0.00000	0.00000	0.00466	0.00265	0.00683	0.00571	0.00692	0.00735
Q91WN4	KMO_MOUSE	0.00000	0.00000	0.00000	0.00000	0.00000	0.00000	0.00000	0.00000	0.00000	0.00102	0.00000	0.00000
Q91WP6	SPA3N_MOUSE	0.00000	0.00000	0.00000	0.00000	0.00000	0.00000	0.01890	0.01580	0.01366	0.01811	0.01494	0.01656
Q91WQ3	SYYC_MOUSE	0.00311	0.00164	0.00121	0.00093	0.00211	0.00031	0.02053	0.01511	0.02276	0.01928	0.01791	0.01482
Q91WR5	AK1CL_MOUSE	0.00000	0.00000	0.00000	0.00000	0.00000	0.00000	0.00000	0.00011	0.00095	0.00026	0.00012	0.00014
Q91WS0	CISD1_MOUSE	0.00000	0.00000	0.00000	0.00000	0.00000	0.00000	0.02003	0.02165	0.01921	0.02282	0.03058	0.02216
Q91WT8	RBM47_MOUSE	0.00000	0.00000	0.00000	0.00000	0.00000	0.00000	0.00000	0.00445	0.00000	0.00000	0.00000	0.00000
Q91WT9	CBS_MOUSE	0.00000	0.00000	0.00000	0.00000	0.00000	0.00000	0.00000	0.00000	0.00000	0.00000	0.00000	0.00000
Q91WU0	EST1F_MOUSE	0.00000	0.00000	0.00000	0.00000	0.00000	0.00000	0.01023	0.01168	0.01594	0.01231	0.01023	0.00801
Q91WU5	AS3MT_MOUSE	0.00285	0.00207	0.00270	0.00293	0.00144	0.00258	0.00307	0.00381	0.00400	0.00228	0.00300	0.00356
Q91WV0	NC2B_MOUSE	0.00000	0.00000	0.00000	0.00000	0.00000	0.00000	0.00438	0.00349	0.00267	0.00363	0.00261	0.00507
Q91WV7	SLC31_MOUSE	0.00000	0.00000	0.00000	0.00000	0.00000	0.00000	0.00000	0.00003	0.00000	0.00002	0.00000	0.00005
Q91X17	UROM_MOUSE	0.00000	0.00000	0.00000	0.00000	0.00000	0.00000	0.00000	0.00000	0.00000	0.00000	0.00000	0.00000
Q91X20	ASH2L_MOUSE	0.00000	0.00000	0.00000	0.00000	0.00000	0.00000	0.00000	0.00000	0.00207	0.00026	0.00104	0.00115
Q91X52	DCXR_MOUSE	0.00000	0.00000	0.00000	0.00000	0.00000	0.00000	0.00000	0.01644	0.01390	0.01277	0.00764	0.01358
Q91X72	HEMO_MOUSE	0.06571	0.02150	0.03441	0.06467	0.03708	0.03018	0.09731	0.09560	0.09870	0.09805	0.10012	0.04571
Q91X91	NADC_MOUSE	0.00000	0.00000	0.00000	0.00000	0.00000	0.00000	0.00000	0.00007	0.00021	0.00000	0.00000	0.00008
Q91X97	NCALD_MOUSE	0.00000	0.00000	0.00000	0.00000	0.00000	0.00000	0.01840	0.01819	0.01552	0.01640	0.01957	0.01794
Q91XA9	CHIA_MOUSE	0.00000	0.00000	0.00000	0.00000	0.00000	0.00000	0.00824	0.00655	0.00598	0.00394	0.00753	0.00886
Q91XD2	LIMS2_MOUSE	0.00000	0.00000	0.00000	0.00000	0.00000	0.00000	0.01022	0.00406	0.00995	0.00300	0.00501	0.00498
Q91XD6	VPS36_MOUSE	0.00000	0.00000	0.00000	0.00000	0.00000	0.00000	0.00183	0.00000	0.00587	0.00663	0.00000	0.00114
Q91XE0	GLYAT_MOUSE	0.00000	0.00000	0.00000	0.00000	0.00000	0.00000	0.00000	0.00000	0.00000	0.00000	0.00000	0.00000
Q91XE4	ACY3_MOUSE	0.00380	0.00407	0.00199	0.00392	0.00319	0.00479	0.00370	0.00741	0.00748	0.00177	0.00470	0.00454
Q91XE8	TM205_MOUSE	0.00000	0.00000	0.00000	0.00000	0.00000	0.00000	0.06548	0.07014	0.06443	0.06934	0.04510	0.06478
Q91XF0	PNPO_MOUSE	0.00000	0.00000	0.00000	0.00000	0.00000	0.00000	0.00115	0.00083	0.00225	0.00093	0.00181	0.00000
Q91XU3	PI42C_MOUSE	0.00000	0.00000	0.00000	0.00000	0.00000	0.00000	0.00178	0.00370	0.00469	0.00485	0.00383	0.00337
Q91XV3	BASP1_MOUSE	0.00000	0.00000	0.00000	0.00000	0.00000	0.00000	0.00054	0.00236	0.00952	0.00782	0.00434	0.00395
Q91XY4	PCDG4_MOUSE	0.00000	0.00000	0.00000	0.00000	0.00000	0.00000	0.00000	0.00261	0.00250	0.00066	0.00093	0.00392
Q91Y63	S13A3_MOUSE	0.00000	0.00000	0.00000	0.00000	0.00000	0.00000	0.00274	0.00000	0.00000	0.00000	0.00000	0.00784
Q91Y97	ALDOB_MOUSE	0.00000	0.00000	0.00000	0.00000	0.00000	0.00000	0.00005	0.00001	0.00007	0.00002	0.00001	0.00002

Q91YD6	VILL_MOUSE	0.00000	0.00000	0.00000	0.00000	0.00000	0.00000	0.00045	0.00036	0.00018	0.00000	0.00015	0.00000
Q91YD9	WASL_MOUSE	0.00000	0.00000	0.00000	0.00000	0.00000	0.00000	0.00000	0.00000	0.00058	0.00011	0.00000	0.00000
Q91YE6	IPO9_MOUSE	0.00000	0.00000	0.00000	0.00000	0.00000	0.00000	0.01000	0.00675	0.01123	0.01032	0.01278	0.01078
Q91YE8	SYNP2_MOUSE	0.02188	0.02598	0.01700	0.01653	0.02314	0.02264	0.00855	0.00261	0.00918	0.00590	0.00298	0.00193
Q91YH5	ATLA3_MOUSE	0.00000	0.00000	0.00000	0.00000	0.00000	0.00000	0.04194	0.03485	0.04731	0.04034	0.03883	0.04342
Q91YI0	ARLY_MOUSE	0.00000	0.00000	0.00000	0.00000	0.00000	0.00000	0.01453	0.01635	0.01628	0.01668	0.01355	0.01749
Q91YJ2	SNX4_MOUSE	0.00000	0.00000	0.00000	0.00000	0.00000	0.00000	0.00366	0.00514	0.00665	0.00646	0.00415	0.00622
Q91YJ5	IF2M_MOUSE	0.00000	0.00000	0.00000	0.00000	0.00000	0.00000	0.00000	0.00000	0.00078	0.00070	0.00000	0.00000
Q91YN5	UAP1_MOUSE	0.00000	0.00000	0.00000	0.00000	0.00000	0.00000	0.00048	0.00042	0.00127	0.00125	0.00137	0.00147
Q91YP0	L2HDH_MOUSE	0.00405	0.01371	0.00437	0.00471	0.00463	0.01004	0.00981	0.00234	0.01470	0.00895	0.01255	0.00827
Q91YP3	DEOC_MOUSE	0.00000	0.00000	0.00000	0.00000	0.00000	0.00000	0.00114	0.00199	0.00255	0.00190	0.00184	0.00171
Q91YQ5	RPN1_MOUSE	0.00725	0.00870	0.00473	0.00418	0.00569	0.00612	0.04600	0.04160	0.04831	0.04349	0.04714	0.04135
Q91YR1	TWF1_MOUSE	0.00000	0.00000	0.00000	0.00000	0.00000	0.00000	0.03686	0.02895	0.03662	0.03374	0.03702	0.03471
Q91YR7	PRP6_MOUSE	0.00000	0.00000	0.00000	0.00000	0.00000	0.00000	0.00000	0.00176	0.00420	0.00279	0.00125	0.00113
Q91YR9	PTGR1_MOUSE	0.00000	0.00000	0.00000	0.00000	0.00000	0.00000	0.03340	0.01417	0.01017	0.00875	0.01458	0.01397
Q91YS8	KCC1A_MOUSE	0.00000	0.00000	0.00000	0.00000	0.00000	0.00000	0.01081	0.01300	0.01531	0.01475	0.01782	0.01559
Q91YT0	NDUV1_MOUSE	0.46404	0.35171	0.43877	0.42467	0.29496	0.33344	0.03185	0.01568	0.01600	0.01901	0.01193	0.01654
Q91YT8	CSCL1_MOUSE	0.00000	0.00000	0.00000	0.00000	0.00000	0.00000	0.00000	0.00000	0.00196	0.00000	0.00072	0.00059
Q91YW3	DNJC3_MOUSE	0.00000	0.00000	0.00000	0.00000	0.00000	0.00000	0.00513	0.00514	0.00715	0.00505	0.00718	0.00430
Q91YY4	ATPF2_MOUSE	0.00000	0.00000	0.00000	0.00000	0.00000	0.00000	0.00000	0.00000	0.00000	0.00000	0.00000	0.00000
Q91Z53	GRHPR_MOUSE	0.00849	0.00494	0.01148	0.00537	0.00465	0.00391	0.01049	0.00676	0.01140	0.00845	0.00943	0.00814
Q91Z67	SRGP2_MOUSE	0.00000	0.00000	0.00000	0.00000	0.00000	0.00000	0.00185	0.00160	0.00134	0.00271	0.00185	0.00326
Q91Z83	MYH7_MOUSE	0.06501	0.12968	0.12003	0.07547	0.08404	0.07936	0.00572	0.00931	0.00835	0.00546	0.00000	0.00493
Q91ZA3	PCCA_MOUSE	0.05961	0.04890	0.04563	0.05406	0.03674	0.06358	0.02551	0.03012	0.02425	0.02034	0.01764	0.03107
Q91ZE0	TMLH_MOUSE	0.00000	0.00000	0.00000	0.00000	0.00000	0.00000	0.00000	0.00000	0.00000	0.00000	0.00000	0.00000
Q91ZH7	ABHD3_MOUSE	0.00000	0.00000	0.00000	0.00000	0.00000	0.00000	0.00000	0.00000	0.00000	0.00000	0.00000	0.00000
Q91ZJ5	UGPA_MOUSE	0.01736	0.00414	0.01475	0.00963	0.00902	0.00723	0.01929	0.01145	0.02059	0.01260	0.02506	0.01586
Q91ZR1	RAB4B_MOUSE	0.00000	0.00000	0.00000	0.00000	0.00000	0.00000	0.00000	0.00467	0.00471	0.00266	0.00342	0.00444
Q91ZR2	SNX18_MOUSE	0.00000	0.00000	0.00000	0.00000	0.00000	0.00000	0.00000	0.00316	0.00360	0.00127	0.00144	0.00110
Q91ZU9	NMD3B_MOUSE	0.00000	0.00000	0.00000	0.00000	0.00000	0.00000	0.00729	0.00576	0.00307	0.00304	0.00000	0.00776
Q91ZW3	SMCA5_MOUSE	0.00000	0.00000	0.00000	0.00000	0.00000	0.00000	0.00228	0.00287	0.00434	0.00414	0.00385	0.00502
Q91ZX7	LRP1_MOUSE	0.00026	0.00000	0.00044	0.00067	0.00036	0.00014	0.01799	0.02025	0.02814	0.02083	0.01897	0.01861
Q920A5	RISC_MOUSE	0.00000	0.00000	0.00000	0.00000	0.00000	0.00000	0.00161	0.00336	0.00093	0.00254	0.00239	0.00443
Q920B9	SP16H_MOUSE	0.00000	0.00000	0.00000	0.00000	0.00000	0.00000	0.00787	0.00732	0.00475	0.00223	0.00127	0.00521

Q920E5	FPPS_MOUSE	0.00000	0.00000	0.00000	0.00000	0.00000	0.00000	0.00717	0.00336	0.00858	0.00681	0.00399	0.00523
Q920Q4	VPS16_MOUSE	0.00000	0.00000	0.00000	0.00000	0.00000	0.00000	0.00017	0.00000	0.00880	0.00401	0.00224	0.00498
Q920Q6	MSI2H_MOUSE	0.00659	0.00428	0.00568	0.00489	0.00549	0.00723	0.00890	0.00923	0.00724	0.00884	0.00773	0.00947
Q920R6	VPP4_MOUSE	0.00000	0.00000	0.00000	0.00000	0.00000	0.00000	0.00069	0.00247	0.00106	0.00391	0.00227	0.00309
Q921C5	BICD2_MOUSE	0.00000	0.00000	0.00000	0.00000	0.00000	0.00000	0.00482	0.00652	0.00454	0.00826	0.00198	0.00932
Q921F2	TADBP_MOUSE	0.00000	0.00000	0.00000	0.00000	0.00000	0.00000	0.00940	0.01756	0.02286	0.02453	0.02940	0.01512
Q921F4	HNRLL_MOUSE	0.00000	0.00000	0.00000	0.00000	0.00000	0.00000	0.01030	0.01415	0.01380	0.01284	0.01311	0.01546
Q921G7	ETFD_MOUSE	0.18000	0.12004	0.16111	0.14449	0.12754	0.07947	0.00640	0.02994	0.02281	0.03023	0.01674	0.01675
Q921H8	THIKA_MOUSE	0.00475	0.01060	0.00598	0.00675	0.00690	0.00963	0.02121	0.02320	0.02228	0.02270	0.02329	0.02490
Q9CQZ0	ORML2_MOUSE	0.00000	0.00000	0.00000	0.00000	0.00000	0.00000	0.00000	0.00000	0.00319	0.00053	0.00030	0.00000
Q921I1	TRFE_MOUSE	0.50740	0.26456	0.32069	0.46785	0.35553	0.25104	0.09629	0.10184	0.09035	0.12703	0.09558	0.06086
Q921I9	EXOS4_MOUSE	0.00000	0.00000	0.00000	0.00000	0.00000	0.00000	0.00366	0.00000	0.00208	0.00375	0.00276	0.00164
Q921J2	RHEB_MOUSE	0.00000	0.00000	0.00000	0.00000	0.00000	0.00000	0.00597	0.00000	0.00267	0.01584	0.00346	0.00000
Q921L3	TMCO1_MOUSE	0.00000	0.00000	0.00000	0.00000	0.00000	0.00000	0.00095	0.00000	0.00153	0.00000	0.00000	0.00000
Q921M3	SF3B3_MOUSE	0.00000	0.00000	0.00000	0.00000	0.00000	0.00000	0.02774	0.03301	0.03347	0.02914	0.03216	0.03528
Q921M4	GOGA2_MOUSE	0.00000	0.00000	0.00000	0.00000	0.00000	0.00000	0.00487	0.00247	0.00502	0.00552	0.00588	0.00444
Q921M7	CYRIB_MOUSE	0.00945	0.00305	0.01083	0.00301	0.02017	0.00239	0.01294	0.01266	0.02397	0.02041	0.02490	0.02596
Q921S7	RM37_MOUSE	0.00933	0.00630	0.00640	0.00613	0.00954	0.00564	0.00176	0.00076	0.00126	0.00109	0.00000	0.00259
Q921T2	TOIP1_MOUSE	0.00130	0.00365	0.00143	0.00090	0.00311	0.00137	0.01065	0.00956	0.01234	0.01176	0.01331	0.01331
Q921U8	SMTN_MOUSE	0.00000	0.00000	0.00000	0.00000	0.00000	0.00000	0.01657	0.00702	0.00748	0.00375	0.01039	0.00945
Q921W4	QORL1_MOUSE	0.00000	0.00000	0.00000	0.00000	0.00000	0.00000	0.00042	0.00000	0.00230	0.00000	0.00057	0.00058
Q921X9	PDIA5_MOUSE	0.00000	0.00000	0.00000	0.00000	0.00000	0.00000	0.00013	0.00127	0.00141	0.00150	0.00382	0.00201
Q921Y0	MOB1A_MOUSE	0.00000	0.00000	0.00000	0.00000	0.00000	0.00000	0.00953	0.01018	0.01169	0.01039	0.00544	0.00997
Q921Z5	TFIP8_MOUSE	0.00000	0.00000	0.00000	0.00000	0.00000	0.00000	0.00040	0.00035	0.00057	0.00029	0.00044	0.00000
Q922B1	MACD1_MOUSE	0.05402	0.03393	0.05981	0.05566	0.04766	0.03142	0.01098	0.00711	0.00000	0.00551	0.00244	0.00000
Q922B2	SYDC_MOUSE	0.00486	0.00532	0.00730	0.00699	0.00863	0.00446	0.02223	0.02350	0.02475	0.02669	0.02842	0.02727
Q922D4	PP6R3_MOUSE	0.00000	0.00000	0.00000	0.00000	0.00000	0.00000	0.00599	0.00529	0.00513	0.00535	0.00594	0.00590
Q922D8	C1TC_MOUSE	0.00434	0.00250	0.00312	0.00511	0.00228	0.00336	0.01948	0.02056	0.02078	0.01937	0.01896	0.02123
Q922E4	PCY2_MOUSE	0.00000	0.00000	0.00000	0.00000	0.00000	0.00000	0.00000	0.00000	0.00540	0.00000	0.00247	0.00179
Q922F4	TBB6_MOUSE	0.01337	0.01091	0.00774	0.01684	0.01439	0.01924	0.00000	0.00395	0.00131	0.00223	0.00000	0.00104
Q922H2	PDK3_MOUSE	0.00000	0.00000	0.00000	0.00000	0.00000	0.00000	0.00000	0.00128	0.00318	0.00258	0.00213	0.00180
Q922H4	GMPPA_MOUSE	0.00000	0.00000	0.00000	0.00000	0.00000	0.00000	0.00226	0.00000	0.00421	0.00433	0.00139	0.00363
Q922J3	CLIP1_MOUSE	0.01380	0.01665	0.00980	0.01065	0.00999	0.00797	0.01925	0.03344	0.03961	0.04118	0.03949	0.02700
Q922J6	TSN2_MOUSE	0.00000	0.00000	0.00000	0.00000	0.00000	0.00000	0.00000	0.00000	0.00266	0.00000	0.00000	0.00000

Q922P9	GLYR1_MOUSE	0.00000	0.00000	0.00000	0.00000	0.00000	0.00000	0.01034	0.00340	0.00875	0.00699	0.01117	0.00942
Q922Q1	MARC2_MOUSE	0.00209	0.00451	0.00231	0.00256	0.00307	0.00416	0.00449	0.00551	0.00372	0.00387	0.00099	0.00452
Q922Q4	P5CR2_MOUSE	0.00000	0.00000	0.00000	0.00000	0.00000	0.00000	0.00000	0.00087	0.00258	0.00140	0.00088	0.00055
Q922Q8	LRC59_MOUSE	0.00000	0.00000	0.00000	0.00000	0.00000	0.00000	0.01008	0.01077	0.00641	0.00735	0.00532	0.00645
Q922Q9	CHID1_MOUSE	0.00000	0.00000	0.00000	0.00000	0.00000	0.00000	0.00262	0.00158	0.00239	0.00115	0.00219	0.00136
Q922R8	PDIA6_MOUSE	0.00349	0.02535	0.01589	0.00736	0.00759	0.00259	0.03525	0.03448	0.05090	0.04680	0.04234	0.04513
Q922Y1	UBXN1_MOUSE	0.00000	0.00000	0.00000	0.00000	0.00000	0.00000	0.01219	0.00884	0.01152	0.01035	0.01083	0.01001
Q922Z0	OXDD_MOUSE	0.00000	0.00000	0.00000	0.00000	0.00000	0.00000	0.00000	0.00000	0.00000	0.00000	0.00000	0.00000
Q923B0	GGACT_MOUSE	0.00000	0.00000	0.00000	0.00000	0.00000	0.00000	0.00000	0.00000	0.00000	0.00000	0.00000	0.00000
Q923B6	STEA4_MOUSE	0.01312	0.00404	0.00274	0.00312	0.00326	0.00163	0.00000	0.00000	0.00192	0.00000	0.00000	0.00000
Q923D2	BLVRB_MOUSE	0.00000	0.00000	0.00000	0.00000	0.00000	0.00000	0.01894	0.01717	0.02388	0.04064	0.04054	0.01554
Q923D4	SF3B5_MOUSE	0.00000	0.00000	0.00000	0.00000	0.00000	0.00000	0.00000	0.00038	0.00321	0.00164	0.00000	0.00152
Q923G2	RPAB3_MOUSE	0.00000	0.00000	0.00000	0.00000	0.00000	0.00000	0.00228	0.00184	0.00265	0.00291	0.00253	0.00131
Q923I7	SC5A2_MOUSE	0.00000	0.00000	0.00000	0.00000	0.00000	0.00000	0.00000	0.01141	0.01347	0.01218	0.00760	0.00618
Q923Q2	STA13_MOUSE	0.00000	0.00000	0.00000	0.00000	0.00000	0.00000	0.03115	0.00367	0.00657	0.00334	0.00797	0.00676
Q923T9	KCC2G_MOUSE	0.00409	0.00552	0.00309	0.00213	0.00269	0.00379	0.00696	0.00542	0.00533	0.00347	0.00626	0.00683
Q923X1	AGRL4_MOUSE	0.00000	0.00000	0.00000	0.00000	0.00000	0.00000	0.00543	0.00615	0.00865	0.00633	0.00761	0.00652
Q923X4	GLRX2_MOUSE	0.00000	0.00000	0.00000	0.00000	0.00000	0.00000	0.00000	0.00000	0.00000	0.00000	0.00000	0.00000
Q924C1	XPO5_MOUSE	0.00000	0.00000	0.00000	0.00000	0.00000	0.00000	0.00000	0.00000	0.00000	0.00224	0.00176	0.00000
Q924D0	RT4I1_MOUSE	0.01921	0.01142	0.02426	0.00971	0.01539	0.01109	0.00000	0.00000	0.00000	0.00000	0.00000	0.00000
Q924K8	MTA3_MOUSE	0.00000	0.00000	0.00000	0.00000	0.00000	0.00000	0.00142	0.00289	0.00321	0.00258	0.00278	0.00246
Q924L1	LTMD1_MOUSE	0.00000	0.00000	0.00000	0.00000	0.00000	0.00000	0.00211	0.00064	0.00165	0.00000	0.00060	0.00131
Q924M7	MPI_MOUSE	0.07415	0.04517	0.07195	0.05459	0.07355	0.04841	0.00754	0.00363	0.00692	0.00661	0.00458	0.00589
Q924N4	S12A6_MOUSE	0.00000	0.00000	0.00000	0.00000	0.00000	0.00000	0.00000	0.00000	0.00000	0.00000	0.00000	0.00000
Q924T2	RT02_MOUSE	0.00000	0.00000	0.00000	0.00000	0.00000	0.00000	0.00000	0.00000	0.00000	0.00000	0.00000	0.00000
Q924X2	CPT1B_MOUSE	0.01295	0.02450	0.00747	0.02046	0.01074	0.04933	0.00295	0.00055	0.00176	0.00305	0.00000	0.00103
Q924Z4	CERS2_MOUSE	0.00000	0.00000	0.00000	0.00000	0.00000	0.00000	0.00476	0.00400	0.00791	0.00645	0.00647	0.00492
Q925B0	PAWR_MOUSE	0.00000	0.00000	0.00000	0.00000	0.00000	0.00000	0.00000	0.00729	0.00713	0.00879	0.00805	0.00792
Q925F2	ESAM_MOUSE	0.00000	0.00000	0.00000	0.00000	0.00000	0.00000	0.03971	0.03580	0.05247	0.04012	0.04939	0.05100
Q925I1	ATAD3_MOUSE	0.00248	0.00113	0.00460	0.00431	0.00292	0.00135	0.00817	0.00118	0.00174	0.00383	0.01878	0.00675
Q925N0	SFXN5_MOUSE	0.00000	0.00000	0.00000	0.00000	0.00000	0.00000	0.00000	0.00258	0.00000	0.00000	0.00000	0.00349
Q925N2	SFXN2_MOUSE	0.00000	0.00000	0.00000	0.00000	0.00000	0.00000	0.00000	0.00218	0.00000	0.00000	0.00000	0.00000
Q93092	TALDO_MOUSE	0.00000	0.00000	0.00000	0.00000	0.00000	0.00000	0.01495	0.05984	0.07981	0.06646	0.06085	0.05180
Q99020	ROAA_MOUSE	0.00000	0.00000	0.00000	0.00000	0.00000	0.00000	0.03935	0.03900	0.03017	0.03799	0.02897	0.03761

Q99388	CSPRS_MOUSE	0.00000	0.00000	0.00000	0.00000	0.00000	0.00000	0.01119	0.00757	0.00869	0.00880	0.01183	0.01144
Q99J08	S14L2_MOUSE	0.00000	0.00000	0.00000	0.00000	0.00000	0.00000	0.00000	0.02847	0.01872	0.01815	0.00866	0.01270
Q99J23	GHDC_MOUSE	0.00000	0.00000	0.00000	0.00000	0.00000	0.00000	0.00000	0.00059	0.00090	0.00000	0.00000	0.00049
Q99J25	MRM1_MOUSE	0.00000	0.00000	0.00000	0.00000	0.00000	0.00000	0.05834	0.00412	0.00553	0.00443	0.01814	0.00281
Q99J27	ACATN_MOUSE	0.00000	0.00000	0.00000	0.00000	0.00000	0.00000	0.00000	0.00000	0.00000	0.00000	0.00000	0.00000
Q99J36	THUM1_MOUSE	0.00000	0.00000	0.00000	0.00000	0.00000	0.00000	0.00311	0.00159	0.00513	0.00324	0.00121	0.00342
Q99J39	DCMC_MOUSE	0.01418	0.01299	0.00914	0.01006	0.00739	0.00901	0.00360	0.00374	0.00000	0.00212	0.00245	0.00320
Q99J45	NRBP_MOUSE	0.00000	0.00000	0.00000	0.00000	0.00000	0.00000	0.00135	0.00058	0.00244	0.00225	0.00131	0.00102
Q99J47	DRS7B_MOUSE	0.00000	0.00000	0.00000	0.00000	0.00000	0.00000	0.00230	0.00428	0.00086	0.00264	0.00000	0.00373
Q99J56	DERL1_MOUSE	0.00000	0.00000	0.00000	0.00000	0.00000	0.00000	0.00154	0.00476	0.00374	0.00250	0.00134	0.00572
Q99J77	SIAS_MOUSE	0.00000	0.00000	0.00000	0.00000	0.00000	0.00000	0.01223	0.01878	0.01672	0.01760	0.00860	0.01362
Q99J93	IFM2_MOUSE	0.00000	0.00000	0.00000	0.00000	0.00000	0.00000	0.01699	0.01936	0.00320	0.02367	0.00125	0.00000
Q99J99	THTM_MOUSE	0.00387	0.00294	0.00282	0.00387	0.00379	0.00261	0.00095	0.00432	0.00259	0.00283	0.00076	0.00134
Q99JB2	STML2_MOUSE	0.00208	0.00278	0.00218	0.00155	0.00238	0.00166	0.01027	0.00975	0.01177	0.01146	0.01132	0.01107
Q99JB7	AMNLS_MOUSE	0.00000	0.00000	0.00000	0.00000	0.00000	0.00000	0.00131	0.00000	0.00000	0.00000	0.00132	0.00000
Q99JB8	PACN3_MOUSE	0.01514	0.01548	0.01037	0.00793	0.01233	0.01622	0.01102	0.00963	0.00532	0.00699	0.00354	0.00779
Q99JF5	MVD1_MOUSE	0.00000	0.00000	0.00000	0.00000	0.00000	0.00000	0.00000	0.00000	0.00000	0.00000	0.00000	0.00000
Q99JF8	PSIP1_MOUSE	0.00000	0.00000	0.00000	0.00000	0.00000	0.00000	0.00756	0.00649	0.00703	0.00506	0.00921	0.00982
Q99JG3	ANX13_MOUSE	0.00000	0.00000	0.00000	0.00000	0.00000	0.00000	0.00559	0.00104	0.00265	0.00221	0.00359	0.00253
Q99JH8	ERD21_MOUSE	0.00000	0.00000	0.00000	0.00000	0.00000	0.00000	0.00852	0.00629	0.01139	0.01016	0.01163	0.00777
Q99JI1	MSTN1_MOUSE	0.00000	0.00000	0.00000	0.00000	0.00000	0.00000	0.00000	0.00060	0.00063	0.00126	0.00062	0.00041
Q99JI4	PSMD6_MOUSE	0.00382	0.00399	0.00375	0.00303	0.00471	0.00497	0.01825	0.01373	0.02303	0.02031	0.02205	0.01967
Q99JI6	RAP1B_MOUSE	0.01195	0.00916	0.00753	0.00568	0.00612	0.00475	0.04750	0.05265	0.05406	0.06659	0.05714	0.06972
Q99JP6	HOME3_MOUSE	0.00000	0.00000	0.00000	0.00000	0.00000	0.00000	0.00150	0.00563	0.00808	0.00490	0.00958	0.00857
Q99JR1	SFXN1_MOUSE	0.00000	0.00000	0.00000	0.00000	0.00000	0.00000	0.01154	0.01073	0.01148	0.00534	0.00773	0.00807
Q99JR5	TINAL_MOUSE	0.00164	0.00151	0.00124	0.00133	0.00232	0.00161	0.04541	0.04413	0.04295	0.02827	0.04795	0.05177
Q99JR8	SMRD2_MOUSE	0.00000	0.00000	0.00000	0.00000	0.00000	0.00000	0.00333	0.00130	0.00469	0.00123	0.00556	0.00256
Q99JW2	ACY1_MOUSE	0.00000	0.00000	0.00000	0.00000	0.00000	0.00000	0.00000	0.00171	0.00000	0.00000	0.00000	0.00104
Q99JW4	LIMS1_MOUSE	0.00000	0.00000	0.00000	0.00000	0.00000	0.00000	0.01488	0.04597	0.01738	0.03107	0.01251	0.03104
Q99JW5	EPCAM_MOUSE	0.00000	0.00000	0.00000	0.00000	0.00000	0.00000	0.01071	0.00786	0.00739	0.00735	0.01381	0.01230
Q99JX3	GORS2_MOUSE	0.00000	0.00000	0.00000	0.00000	0.00000	0.00000	0.00875	0.01752	0.01039	0.01296	0.01019	0.01502
Q99JX4	EIF3M_MOUSE	0.00000	0.00000	0.00000	0.00000	0.00000	0.00000	0.00698	0.00837	0.00992	0.00847	0.01215	0.00752
Q99JX7	NXF1_MOUSE	0.00000	0.00000	0.00000	0.00000	0.00000	0.00000	0.00000	0.00000	0.00210	0.00123	0.00000	0.00000
Q99JY0	ECHB_MOUSE	0.48759	0.35029	0.43028	0.37308	0.39294	0.41038	0.08924	0.04718	0.05549	0.05314	0.05241	0.04787

Q99JY3	GIMA4_MOUSE	0.00603	0.00913	0.01335	0.01547	0.02125	0.00823	0.08778	0.07972	0.11799	0.10463	0.12842	0.10399
Q99JY9	ARP3_MOUSE	0.00536	0.00551	0.00523	0.00741	0.00238	0.00144	0.06713	0.07627	0.07188	0.07867	0.06694	0.07115
Q99K01	PDXD1_MOUSE	0.00000	0.00000	0.00000	0.00000	0.00000	0.00000	0.00120	0.00277	0.00644	0.00441	0.00614	0.00382
Q99K23	UFSP2_MOUSE	0.00000	0.00000	0.00000	0.00000	0.00000	0.00000	0.00000	0.00084	0.00202	0.00090	0.00000	0.00000
Q99K28	ARFG2_MOUSE	0.00000	0.00000	0.00000	0.00000	0.00000	0.00000	0.00301	0.00199	0.00423	0.00155	0.00374	0.00382
Q99K30	ES8L2_MOUSE	0.00000	0.00000	0.00000	0.00000	0.00000	0.00000	0.01251	0.01148	0.01580	0.01193	0.01681	0.01633
Q99K41	EMIL1_MOUSE	0.00029	0.00041	0.00037	0.00054	0.00017	0.00000	0.06343	0.04507	0.04709	0.02741	0.06878	0.06789
Q99K48	NONO_MOUSE	0.00000	0.00000	0.00000	0.00000	0.00000	0.00000	0.03182	0.04266	0.02797	0.04465	0.03525	0.04280
Q99K51	PLST_MOUSE	0.00332	0.00287	0.00288	0.00296	0.00301	0.00417	0.10053	0.11472	0.11264	0.10839	0.10033	0.11557
Q99K67	AASS_MOUSE	0.00000	0.00000	0.00000	0.00000	0.00000	0.00000	0.00005	0.00002	0.00021	0.00021	0.00016	0.00003
Q99K70	RRAGC_MOUSE	0.00000	0.00000	0.00000	0.00000	0.00000	0.00000	0.00584	0.00937	0.00859	0.00842	0.00735	0.00917
Q99KB8	GLO2_MOUSE	0.00000	0.00000	0.00000	0.00000	0.00000	0.00000	0.00030	0.00274	0.00356	0.00250	0.00200	0.00152
Q99KC7	SMAGP_MOUSE	0.00000	0.00000	0.00000	0.00000	0.00000	0.00000	0.00000	0.00000	0.00291	0.00000	0.00287	0.00000
Q99KC8	VMA5A_MOUSE	0.00695	0.00595	0.00627	0.00419	0.00489	0.00460	0.01862	0.01981	0.02434	0.02245	0.01595	0.02155
Q99KD5	UN45A_MOUSE	0.00000	0.00000	0.00000	0.00000	0.00000	0.00000	0.00441	0.00441	0.00441	0.00609	0.00454	0.00347
Q99KF1	TMED9_MOUSE	0.00314	0.00377	0.00197	0.00427	0.00445	0.00454	0.00246	0.00924	0.01476	0.00942	0.00427	0.00821
Q99KH8	STK24_MOUSE	0.00000	0.00000	0.00000	0.00000	0.00000	0.00000	0.01733	0.01631	0.01549	0.01491	0.01635	0.01661
Q99KI0	ACON_MOUSE	3.95162	3.72043	3.39566	3.75236	3.98764	2.97736	0.16959	0.09640	0.09272	0.10445	0.09484	0.09048
Q99KI3	EMC3_MOUSE	0.00000	0.00000	0.00000	0.00000	0.00000	0.00000	0.00365	0.00691	0.00375	0.00629	0.00226	0.00853
Q99KJ8	DCTN2_MOUSE	0.02354	0.02092	0.01956	0.02159	0.01641	0.02148	0.05298	0.05071	0.05941	0.05389	0.05524	0.06725
Q99KK7	DPP3_MOUSE	0.00815	0.00490	0.00540	0.00684	0.00657	0.00443	0.01399	0.01181	0.01763	0.01593	0.01786	0.01779
Q99KN9	EPN4_MOUSE	0.00000	0.00000	0.00000	0.00000	0.00000	0.00000	0.00504	0.00858	0.01155	0.00986	0.00644	0.00841
Q99KP3	CRYL1_MOUSE	0.00000	0.00000	0.00000	0.00000	0.00000	0.00000	0.01824	0.02770	0.02022	0.02054	0.03109	0.03142
Q99KP6	PRP19_MOUSE	0.00000	0.00000	0.00000	0.00000	0.00000	0.00000	0.00781	0.00861	0.01427	0.01207	0.01545	0.01226
Q99KQ4	NAMPT_MOUSE	0.02794	0.02232	0.02972	0.02027	0.03133	0.02685	0.01955	0.01708	0.02567	0.01598	0.03309	0.02309
Q99KR3	LACB2_MOUSE	0.00000	0.00000	0.00000	0.00000	0.00000	0.00000	0.00000	0.00200	0.00461	0.00236	0.00220	0.00134
Q99KR7	PPIF_MOUSE	0.00000	0.00000	0.00000	0.00000	0.00000	0.00000	0.00000	0.00000	0.00000	0.00000	0.00000	0.00000
Q99KS6	ADPRM_MOUSE	0.00000	0.00000	0.00000	0.00000	0.00000	0.00000	0.00000	0.00000	0.00118	0.00000	0.00000	0.00000
Q99KV1	DJB11_MOUSE	0.00197	0.00133	0.00256	0.00195	0.00265	0.00226	0.02052	0.01498	0.02060	0.01540	0.01951	0.01556
Q99KW9	TIP_MOUSE	0.00000	0.00000	0.00000	0.00000	0.00000	0.00000	0.00042	0.00000	0.00205	0.00052	0.00000	0.00000
Q99KY4	GAK_MOUSE	0.00000	0.00000	0.00000	0.00000	0.00000	0.00000	0.00519	0.00477	0.00847	0.00201	0.00769	0.00710
Q99L04	DHRS1_MOUSE	0.00399	0.00245	0.00241	0.00226	0.00360	0.00128	0.00000	0.00063	0.00586	0.00392	0.00229	0.00217
Q99L13	3HIDH_MOUSE	0.05613	0.03008	0.02638	0.03468	0.03308	0.03500	0.02128	0.01966	0.02237	0.01895	0.02062	0.02069
Q99L20	GSTT3_MOUSE	0.00000	0.00000	0.00000	0.00000	0.00000	0.00000	0.02251	0.02920	0.03176	0.02690	0.03406	0.03140

Q99L43	CDS2_MOUSE	0.00393	0.00533	0.00726	0.00776	0.00475	0.00709	0.04802	0.03935	0.03772	0.04218	0.03776	0.04466
Q99L45	IF2B_MOUSE	0.00000	0.00000	0.00000	0.00000	0.00000	0.00000	0.01510	0.01039	0.01043	0.00973	0.00936	0.00964
Q99L47	F10A1_MOUSE	0.00000	0.00000	0.00000	0.00000	0.00000	0.00000	0.02463	0.03830	0.02013	0.02772	0.02150	0.02922
Q99L60	VATC2_MOUSE	0.00000	0.00000	0.00000	0.00000	0.00000	0.00000	0.00000	0.00000	0.00000	0.00000	0.00000	0.00000
Q99L88	SNTB1_MOUSE	0.00000	0.00000	0.00000	0.00000	0.00000	0.00000	0.00479	0.00723	0.00406	0.00342	0.00460	0.00478
Q99LB2	DHRS4_MOUSE	0.00945	0.00981	0.00962	0.00542	0.01192	0.00706	0.01495	0.00648	0.00950	0.01003	0.00798	0.00971
Q99LB6	MAT2B_MOUSE	0.00000	0.00000	0.00000	0.00000	0.00000	0.00000	0.00296	0.00078	0.00307	0.00256	0.00358	0.00275
Q99LB7	SARDH_MOUSE	0.00000	0.00000	0.00000	0.00000	0.00000	0.00000	0.00039	0.00010	0.00019	0.00003	0.00005	0.00011
Q99LC3	NDUAA_MOUSE	0.14226	0.12524	0.18662	0.16818	0.14912	0.20787	0.01916	0.01000	0.01422	0.01273	0.01142	0.01083
Q99LC5	ETFA_MOUSE	1.22079	0.64266	1.25194	0.81125	0.96389	0.80340	0.06712	0.04321	0.04740	0.04671	0.04374	0.04935
Q99LD4	CSN1_MOUSE	0.00000	0.00000	0.00000	0.00000	0.00000	0.00000	0.01675	0.01218	0.01600	0.01325	0.01643	0.01358
Q99LD8	DDAH2_MOUSE	0.00000	0.00000	0.00000	0.00000	0.00000	0.00000	0.04191	0.02989	0.04216	0.04485	0.04426	0.04918
Q99LD9	EI2BB_MOUSE	0.00000	0.00000	0.00000	0.00000	0.00000	0.00000	0.00185	0.00018	0.00422	0.00378	0.00421	0.00313
Q99LF4	RTCB_MOUSE	0.00272	0.00178	0.00370	0.00189	0.00282	0.00046	0.00509	0.01418	0.01175	0.00906	0.01321	0.01558
Q99LG2	TNPO2_MOUSE	0.00000	0.00000	0.00000	0.00000	0.00000	0.00000	0.00000	0.00485	0.00074	0.00169	0.00170	0.00141
Q99LI8	HGS_MOUSE	0.00000	0.00000	0.00000	0.00000	0.00000	0.00000	0.00000	0.00508	0.00380	0.00385	0.00259	0.00748
Q99LJ0	CT2NL_MOUSE	0.00000	0.00000	0.00000	0.00000	0.00000	0.00000	0.00000	0.00029	0.00123	0.00000	0.00075	0.00049
Q99LJ1	FUCO_MOUSE	0.00000	0.00000	0.00000	0.00000	0.00000	0.00000	0.00052	0.00048	0.00172	0.00026	0.00063	0.00024
Q99LP6	GRPE1_MOUSE	0.00000	0.00000	0.00000	0.00000	0.00000	0.00000	0.00352	0.00185	0.00435	0.00339	0.00428	0.00331
Q99LR1	ABD12_MOUSE	0.00000	0.00000	0.00000	0.00000	0.00000	0.00000	0.00321	0.00215	0.00566	0.00394	0.00667	0.00475
Q99LX0	PARK7_MOUSE	0.03186	0.03395	0.02924	0.04739	0.03986	0.05993	0.04693	0.04022	0.04425	0.04087	0.05809	0.04193
Q99LY9	NDUS5_MOUSE	0.03080	0.01756	0.03745	0.02743	0.02741	0.02373	0.00115	0.00979	0.00421	0.00904	0.00591	0.00452
Q99M01	SYFM_MOUSE	0.00000	0.00000	0.00000	0.00000	0.00000	0.00000	0.00000	0.00000	0.00000	0.00000	0.00000	0.00000
Q99M08	CD003_MOUSE	0.00000	0.00000	0.00000	0.00000	0.00000	0.00000	0.00000	0.00344	0.00000	0.00138	0.00000	0.00087
Q99M28	RNPS1_MOUSE	0.00000	0.00000	0.00000	0.00000	0.00000	0.00000	0.00073	0.00388	0.00873	0.01247	0.01149	0.01091
Q99M51	NCK1_MOUSE	0.00000	0.00000	0.00000	0.00000	0.00000	0.00000	0.00335	0.00578	0.00472	0.00328	0.00097	0.00387
Q99M87	DNJA3_MOUSE	0.00635	0.01066	0.00482	0.00713	0.00760	0.00555	0.00526	0.00641	0.00048	0.00184	0.00171	0.00114
Q99MK8	ARBK1_MOUSE	0.00000	0.00000	0.00000	0.00000	0.00000	0.00000	0.00120	0.00237	0.00415	0.00598	0.00379	0.00496
Q99MN1	SYK_MOUSE	0.00479	0.00616	0.00680	0.00537	0.00499	0.00487	0.00989	0.01065	0.01700	0.01206	0.01413	0.01015
Q99MN9	PCCB_MOUSE	0.06117	0.08099	0.07435	0.07723	0.07722	0.08154	0.01928	0.03055	0.01238	0.01481	0.01417	0.01956
Q99MQ4	ASPN_MOUSE	0.00000	0.00000	0.00000	0.00000	0.00000	0.00000	0.03343	0.00859	0.01246	0.00897	0.01234	0.00787
Q99MR6	SRRT_MOUSE	0.00000	0.00000	0.00000	0.00000	0.00000	0.00000	0.00490	0.00400	0.00636	0.00639	0.00723	0.00713
Q99MR8	MCCA_MOUSE	0.10680	0.11618	0.09852	0.10600	0.13088	0.09927	0.01715	0.01255	0.01293	0.01014	0.01311	0.01163
Q99MV7	RNF17_MOUSE	0.00000	0.00000	0.00000	0.00000	0.00000	0.00000	0.00000	0.01698	0.00000	0.00000	0.01768	0.01739

Q99MZ6	MYO7B_MOUSE	0.00000	0.00000	0.00000	0.00000	0.00000	0.00000	0.00000	0.00000	0.00000	0.00000	0.00000	0.00000
Q99MZ7	PECR_MOUSE	0.00000	0.00000	0.00000	0.00000	0.00000	0.00000	0.00000	0.00000	0.00000	0.00000	0.00000	0.00004
Q99N05	M4A4D_MOUSE	0.00000	0.00000	0.00000	0.00000	0.00000	0.00000	0.00000	0.00000	0.00000	0.00000	0.00000	0.00000
Q99N93	RM16_MOUSE	0.00000	0.00000	0.00000	0.00000	0.00000	0.00000	0.00000	0.00000	0.00000	0.00000	0.00000	0.00000
Q99NB1	ACS2L_MOUSE	0.12419	0.13908	0.14731	0.11604	0.13554	0.15168	0.03001	0.02131	0.01504	0.01353	0.02248	0.02209
Q99NB9	SF3B1_MOUSE	0.00000	0.00000	0.00000	0.00000	0.00000	0.00000	0.01199	0.00806	0.01241	0.01063	0.01332	0.01247
Q99NH0	ANR17_MOUSE	0.00000	0.00000	0.00000	0.00000	0.00000	0.00000	0.00000	0.00051	0.00469	0.00136	0.00457	0.00088
Q99P31	HPBP1_MOUSE	0.00000	0.00000	0.00000	0.00000	0.00000	0.00000	0.00174	0.00183	0.00498	0.00266	0.00293	0.00379
Q99P58	RB27B_MOUSE	0.00000	0.00000	0.00000	0.00000	0.00000	0.00000	0.00000	0.00000	0.00368	0.00494	0.00000	0.00524
Q99P72	RTN4_MOUSE	0.00379	0.00381	0.00614	0.00472	0.00376	0.00187	0.06898	0.07049	0.10417	0.09035	0.08286	0.08266
Q99P88	NU155_MOUSE	0.00000	0.00000	0.00000	0.00000	0.00000	0.00000	0.00473	0.00431	0.00786	0.00569	0.00826	0.00522
Q99PG0	AAAD_MOUSE	0.00000	0.00000	0.00000	0.00000	0.00000	0.00000	0.00000	0.00000	0.00000	0.00000	0.00000	0.00000
Q99PG2	OGFR_MOUSE	0.00000	0.00000	0.00000	0.00000	0.00000	0.00000	0.00000	0.00087	0.00791	0.01124	0.00390	0.00334
Q99PL5	RRBP1_MOUSE	0.00179	0.00163	0.00159	0.00159	0.00148	0.00161	0.01397	0.01860	0.02624	0.01929	0.01590	0.01846
Q99PL6	UBXN6_MOUSE	0.00000	0.00000	0.00000	0.00000	0.00000	0.00000	0.00326	0.00280	0.00212	0.00397	0.00440	0.00547
Q99PP9	TRI16_MOUSE	0.00000	0.00000	0.00000	0.00000	0.00000	0.00000	0.00370	0.01465	0.01532	0.01751	0.00890	0.01249
Q99PR8	HSPB2_MOUSE	0.00000	0.00000	0.00000	0.00000	0.00000	0.00000	0.00177	0.00000	0.00000	0.00000	0.00000	0.00000
Q99PT1	GDIR1_MOUSE	0.05888	0.04831	0.05362	0.03437	0.05172	0.04672	0.24083	0.28876	0.31029	0.28077	0.28902	0.27792
Q99PV0	PRP8_MOUSE	0.00345	0.00133	0.00303	0.00438	0.00270	0.00033	0.00870	0.00761	0.00862	0.00742	0.00805	0.00611
Q99PW4	PRPK_MOUSE	0.00000	0.00000	0.00000	0.00000	0.00000	0.00000	0.00000	0.00417	0.00000	0.00000	0.00000	0.00000
Q9CPP6	NDUA5_MOUSE	0.00000	0.00000	0.00000	0.00000	0.00000	0.00000	0.02050	0.00780	0.01328	0.01212	0.01230	0.01022
Q9CPQ1	COX6C_MOUSE	0.00000	0.00000	0.00000	0.00000	0.00000	0.00000	0.01165	0.00514	0.01338	0.00806	0.01047	0.00546
Q9CPQ3	TOM22_MOUSE	0.00000	0.00000	0.00000	0.00000	0.00000	0.00000	0.00401	0.00566	0.01339	0.00690	0.01273	0.00969
Q9CPQ8	ATP5L_MOUSE	0.17967	0.13923	0.14406	0.11605	0.16054	0.13589	0.14690	0.09764	0.11686	0.09043	0.09149	0.11974
Q9CPR4	RL17_MOUSE	0.00577	0.00687	0.00421	0.00780	0.00525	0.00147	0.04359	0.03877	0.04156	0.04357	0.04124	0.03668
Q9CPR5	RM15_MOUSE	0.00000	0.00000	0.00000	0.00000	0.00000	0.00000	0.00000	0.00195	0.00277	0.00000	0.00189	0.00263
Q9CPT3	NANP_MOUSE	0.00000	0.00000	0.00000	0.00000	0.00000	0.00000	0.00000	0.00088	0.00075	0.00058	0.00194	0.00043
Q9CPU0	LGUL_MOUSE	0.00000	0.00000	0.00000	0.00000	0.00000	0.00000	0.01902	0.01641	0.01109	0.01767	0.01465	0.01364
Q9CPU2	NDUB2_MOUSE	0.00000	0.00000	0.00000	0.00000	0.00000	0.00000	0.00000	0.00000	0.00000	0.00000	0.00000	0.00000
Q9CPU4	MGST3_MOUSE	0.05588	0.06112	0.10665	0.08646	0.04866	0.08865	0.00857	0.02000	0.01693	0.01669	0.00657	0.01700
Q9CPV4	GLOD4_MOUSE	0.00228	0.00144	0.00228	0.00286	0.00160	0.00331	0.02029	0.02287	0.03212	0.02318	0.03624	0.03140
Q9CPW4	ARPC5_MOUSE	0.00000	0.00000	0.00000	0.00000	0.00000	0.00000	0.01330	0.02488	0.02610	0.02167	0.02953	0.02566
Q9CPX6	ATG3_MOUSE	0.00000	0.00000	0.00000	0.00000	0.00000	0.00000	0.01444	0.01581	0.01779	0.01192	0.00824	0.01575
Q9CPX8	QCR10_MOUSE	0.00000	0.00000	0.00000	0.00000	0.00000	0.00000	0.00057	0.00000	0.00013	0.00016	0.00023	0.00000

Q9CPY7	AMPL_MOUSE	0.02626	0.01979	0.01915	0.01940	0.01986	0.01378	0.01826	0.00969	0.02048	0.01602	0.02119	0.01245
Q9CPZ6	ORML3_MOUSE	0.00000	0.00000	0.00000	0.00000	0.00000	0.00000	0.00000	0.00000	0.00100	0.00012	0.00000	0.00000
Q9CQ10	CHMP3_MOUSE	0.00000	0.00000	0.00000	0.00000	0.00000	0.00000	0.00340	0.00382	0.00147	0.00366	0.00100	0.00333
Q9CQ19	MYL9_MOUSE	0.00000	0.00000	0.00000	0.00000	0.00000	0.00000	0.01985	0.04109	0.03607	0.05368	0.01506	0.03534
Q9CQ20	M1IP1_MOUSE	0.00000	0.00000	0.00000	0.00000	0.00000	0.00000	0.00232	0.00451	0.00610	0.00539	0.00565	0.00387
Q9CQ21	MCTS2_MOUSE	0.00000	0.00000	0.00000	0.00000	0.00000	0.00000	0.00483	0.00240	0.00154	0.00110	0.00256	0.00117
Q9CQ22	LTOR1_MOUSE	0.00000	0.00000	0.00000	0.00000	0.00000	0.00000	0.00796	0.00641	0.00548	0.00606	0.00000	0.00852
Q9CQ48	NUDC2_MOUSE	0.00000	0.00000	0.00000	0.00000	0.00000	0.00000	0.00100	0.00281	0.00000	0.00237	0.00117	0.00331
Q9CQ54	NDUC2_MOUSE	0.01405	0.01837	0.02240	0.04469	0.02805	0.04390	0.01566	0.00478	0.01187	0.00864	0.00762	0.01033
Q9CQ60	6PGL_MOUSE	0.00612	0.00436	0.00518	0.00706	0.00544	0.00396	0.01163	0.00321	0.02036	0.01475	0.02759	0.02116
Q9CQ62	DECR_MOUSE	0.24520	0.31931	0.18292	0.15964	0.25986	0.31814	0.07997	0.03488	0.04802	0.03753	0.04760	0.03886
Q9CQ65	MTAP_MOUSE	0.00435	0.00217	0.00399	0.00380	0.00524	0.00328	0.02376	0.02401	0.02522	0.02028	0.02460	0.02670
Q9CQ69	QCR8_MOUSE	0.00000	0.00000	0.00000	0.00000	0.00000	0.00000	0.01046	0.00433	0.00593	0.00404	0.00306	0.00194
Q9CQ75	NDUA2_MOUSE	0.00000	0.00000	0.00000	0.00000	0.00000	0.00000	0.00205	0.00066	0.00221	0.00323	0.00224	0.00580
Q9CQ80	VPS25_MOUSE	0.00000	0.00000	0.00000	0.00000	0.00000	0.00000	0.00000	0.00197	0.00430	0.00824	0.00377	0.00537
Q9CQ89	CUTA_MOUSE	0.00000	0.00000	0.00000	0.00000	0.00000	0.00000	0.00812	0.00856	0.00828	0.00764	0.00346	0.01224
Q9CQ91	NDUA3_MOUSE	0.00000	0.00000	0.00000	0.00000	0.00000	0.00000	0.00488	0.00419	0.00494	0.00425	0.00252	0.00768
Q9CQ92	FIS1_MOUSE	0.01620	0.00917	0.00974	0.00792	0.01319	0.01365	0.00777	0.00548	0.01083	0.00749	0.00504	0.00768
Q9CQA3	SDHB_MOUSE	0.21181	0.06318	0.12175	0.11388	0.20319	0.12282	0.05114	0.04148	0.03004	0.02896	0.02299	0.03975
Q9CQB5	CISD2_MOUSE	0.00546	0.00656	0.00381	0.00273	0.00431	0.00616	0.00527	0.00432	0.00444	0.00471	0.00448	0.00338
Q9CQC7	NDUB4_MOUSE	0.00000	0.00000	0.00000	0.00000	0.00000	0.00000	0.01797	0.01674	0.01516	0.01359	0.01834	0.01536
Q9CQC8	SPG21_MOUSE	0.00000	0.00000	0.00000	0.00000	0.00000	0.00000	0.00000	0.00000	0.00000	0.00000	0.00000	0.00000
Q9CQC9	SAR1B_MOUSE	0.00000	0.00000	0.00000	0.00000	0.00000	0.00000	0.02005	0.02806	0.01262	0.01605	0.01089	0.01555
Q9CQD1	RAB5A_MOUSE	0.00000	0.00000	0.00000	0.00000	0.00000	0.00000	0.02017	0.03759	0.02193	0.02641	0.02621	0.02106
Q9CQE1	NPS3B_MOUSE	0.00000	0.00000	0.00000	0.00000	0.00000	0.00000	0.00880	0.00727	0.00793	0.00814	0.02064	0.01025
Q9CQE3	RT17_MOUSE	0.00000	0.00000	0.00000	0.00000	0.00000	0.00000	0.00000	0.00000	0.00000	0.00000	0.00000	0.00000
Q9CQE5	RGS10_MOUSE	0.00000	0.00000	0.00000	0.00000	0.00000	0.00000	0.00000	0.00000	0.00679	0.00389	0.00000	0.00000
Q9CQE8	RTRAF_MOUSE	0.00000	0.00000	0.00000	0.00000	0.00000	0.00000	0.00939	0.01056	0.01511	0.01125	0.01378	0.01278
Q9CQF3	CPSF5_MOUSE	0.00000	0.00000	0.00000	0.00000	0.00000	0.00000	0.00306	0.00614	0.00908	0.00698	0.00645	0.00818
Q9CQF8	RT63_MOUSE	0.00000	0.00000	0.00000	0.00000	0.00000	0.00000	0.00000	0.00000	0.00041	0.00063	0.00000	0.00000
Q9CQF9	PCYOX_MOUSE	0.00158	0.00073	0.00133	0.00132	0.00111	0.00108	0.01165	0.02038	0.01855	0.01298	0.00959	0.01230
Q9CQG1	CHAC2_MOUSE	0.00000	0.00000	0.00000	0.00000	0.00000	0.00000	0.00000	0.00000	0.00048	0.00000	0.00000	0.00000
Q9CQG9	TM100_MOUSE	0.00000	0.00000	0.00000	0.00000	0.00000	0.00000	0.00000	0.00236	0.00297	0.00248	0.00152	0.00110
Q9CQH0	PDZ1I_MOUSE	0.00000	0.00000	0.00000	0.00000	0.00000	0.00000	0.00000	0.00000	0.00000	0.00000	0.00000	0.00000

Q9CQH3	NDUB5_MOUSE	0.00000	0.00000	0.00000	0.00000	0.00000	0.00000	0.01582	0.01183	0.00763	0.01033	0.00646	0.01100
Q9CQH7	BT3L4_MOUSE	0.00000	0.00000	0.00000	0.00000	0.00000	0.00000	0.00219	0.00726	0.00875	0.00869	0.01031	0.00935
Q9CQI3	GMFB_MOUSE	0.00000	0.00000	0.00000	0.00000	0.00000	0.00000	0.01991	0.02716	0.01639	0.02040	0.01043	0.01884
Q9CQI6	COTL1_MOUSE	0.00000	0.00000	0.00000	0.00000	0.00000	0.00000	0.01819	0.01872	0.01846	0.02278	0.01706	0.02498
Q9CQI7	RU2B_MOUSE	0.00000	0.00000	0.00000	0.00000	0.00000	0.00000	0.00000	0.00000	0.00000	0.00376	0.00477	0.00389
Q9CQJ6	DENR_MOUSE	0.01353	0.00906	0.00941	0.00600	0.00788	0.00312	0.00480	0.00580	0.00609	0.00639	0.00644	0.00544
Q9CQJ8	NDUB9_MOUSE	0.17942	0.07592	0.19687	0.15037	0.09789	0.09606	0.01883	0.00653	0.00883	0.00801	0.00349	0.00382
Q9CQM5	TXD17_MOUSE	0.00459	0.00467	0.00309	0.00495	0.00379	0.00295	0.00844	0.00704	0.00201	0.00493	0.00000	0.00151
Q9CQM9	GLRX3_MOUSE	0.00937	0.00900	0.01338	0.00709	0.00858	0.00741	0.01171	0.00732	0.01197	0.00869	0.01311	0.00760
Q9CQN1	TRAP1_MOUSE	0.04699	0.03578	0.03161	0.03263	0.03562	0.04514	0.01157	0.00774	0.01681	0.01071	0.01618	0.01128
Q9CQN6	TM14C_MOUSE	0.00000	0.00000	0.00000	0.00000	0.00000	0.00000	0.00628	0.00804	0.00904	0.00759	0.00614	0.00790
Q9CQP2	TPPC2_MOUSE	0.00000	0.00000	0.00000	0.00000	0.00000	0.00000	0.00000	0.00562	0.00393	0.00197	0.00515	0.00305
Q9CQQ7	AT5F1_MOUSE	0.11157	0.03976	0.12872	0.10198	0.08511	0.04831	0.03404	0.03271	0.04224	0.03683	0.03769	0.02818
Q9CQR2	RS21_MOUSE	0.00000	0.00000	0.00000	0.00000	0.00000	0.00000	0.01448	0.01147	0.00819	0.01188	0.00588	0.01495
Q9CQR4	ACO13_MOUSE	0.00324	0.00261	0.00317	0.00239	0.00223	0.00323	0.00245	0.00770	0.00261	0.00227	0.00468	0.00132
Q9CQR6	PPP6_MOUSE	0.00000	0.00000	0.00000	0.00000	0.00000	0.00000	0.01366	0.01479	0.01864	0.01464	0.01653	0.02002
Q9CQS8	SC61B_MOUSE	0.00000	0.00000	0.00000	0.00000	0.00000	0.00000	0.00000	0.00949	0.02093	0.01436	0.00860	0.00775
Q9CQU3	RER1_MOUSE	0.00000	0.00000	0.00000	0.00000	0.00000	0.00000	0.01664	0.01456	0.01803	0.01609	0.01535	0.01514
Q9CQV8	1433B_MOUSE	0.00000	0.00000	0.00000	0.00000	0.00000	0.00000	0.08329	0.14996	0.17774	0.17218	0.09412	0.13492
Q9CQW1	YKT6_MOUSE	0.00000	0.00000	0.00000	0.00000	0.00000	0.00000	0.00659	0.00858	0.00813	0.00784	0.00750	0.00603
Q9CQW9	IFM3_MOUSE	0.00000	0.00000	0.00000	0.00000	0.00000	0.00000	0.01341	0.01349	0.02398	0.01434	0.02527	0.00952
Q9CQX2	CYB5B_MOUSE	0.02500	0.01330	0.01417	0.01751	0.01004	0.01099	0.00266	0.01479	0.02023	0.01662	0.01617	0.02416
Q9CQX8	RT36_MOUSE	0.00290	0.00175	0.00436	0.00458	0.00382	0.00084	0.00000	0.00532	0.00000	0.00124	0.00000	0.00161
Q9CQY5	MAGT1_MOUSE	0.00000	0.00000	0.00000	0.00000	0.00000	0.00000	0.00000	0.00661	0.00253	0.00763	0.00384	0.00733
Q9CQY6	UQCC2_MOUSE	0.00000	0.00000	0.00000	0.00000	0.00000	0.00000	0.00000	0.00580	0.00000	0.00000	0.00000	0.00000
Q9CQZ1	HSBP1_MOUSE	0.00000	0.00000	0.00000	0.00000	0.00000	0.00000	0.00041	0.01032	0.00043	0.00618	0.00557	0.00527
Q9CQZ5	NDUA6_MOUSE	0.06017	0.02102	0.05045	0.05064	0.05505	0.02731	0.00000	0.00356	0.00483	0.00312	0.00159	0.00164
Q9CQZ6	NDUB3_MOUSE	0.00000	0.00000	0.00000	0.00000	0.00000	0.00000	0.00000	0.00275	0.00265	0.00458	0.00206	0.00111
Q9CR00	PSMD9_MOUSE	0.00000	0.00000	0.00000	0.00000	0.00000	0.00000	0.00045	0.00239	0.00311	0.00332	0.00000	0.00287
Q9CR09	UFC1_MOUSE	0.00000	0.00000	0.00000	0.00000	0.00000	0.00000	0.00000	0.00000	0.00272	0.00371	0.00000	0.00349
Q9CR16	PPID_MOUSE	0.00000	0.00000	0.00000	0.00000	0.00000	0.00000	0.02383	0.01629	0.01506	0.01390	0.01276	0.01916
Q9CR20	IR3IP_MOUSE	0.00000	0.00000	0.00000	0.00000	0.00000	0.00000	0.01771	0.01253	0.01091	0.01280	0.00741	0.01439
Q9CR21	ACPM_MOUSE	0.13119	0.13550	0.20791	0.16282	0.17308	0.11530	0.00000	0.00628	0.00498	0.00412	0.00103	0.00145
Q9CR26	VTA1_MOUSE	0.00000	0.00000	0.00000	0.00000	0.00000	0.00000	0.01306	0.01087	0.02221	0.01264	0.01960	0.01450

Q9CR29	CCD43_MOUSE	0.00000	0.00000	0.00000	0.00000	0.00000	0.00000	0.00000	0.00000	0.00244	0.00000	0.00201	0.00327
Q9CR39	WIPI3_MOUSE	0.00000	0.00000	0.00000	0.00000	0.00000	0.00000	0.00000	0.00000	0.00000	0.00000	0.00000	0.00000
Q9CR41	HYPK_MOUSE	0.00000	0.00000	0.00000	0.00000	0.00000	0.00000	0.00000	0.00649	0.00088	0.00193	0.00308	0.00368
Q9CR51	VATG1_MOUSE	0.00000	0.00000	0.00000	0.00000	0.00000	0.00000	0.03282	0.02556	0.02119	0.02383	0.02182	0.02369
Q9CR57	RL14_MOUSE	0.00000	0.00000	0.00000	0.00000	0.00000	0.00000	0.01664	0.04537	0.02076	0.04117	0.02604	0.03789
Q9CR61	NDUB7_MOUSE	0.02456	0.01781	0.01729	0.06027	0.01121	0.05897	0.00834	0.00569	0.00237	0.00596	0.00521	0.00654
Q9CR62	M2OM_MOUSE	0.06939	0.06039	0.08517	0.05650	0.07124	0.06103	0.03612	0.04323	0.04264	0.03648	0.03539	0.03941
Q9CR67	TMM33_MOUSE	0.00000	0.00000	0.00000	0.00000	0.00000	0.00000	0.00041	0.00372	0.00187	0.00199	0.00130	0.00592
Q9CR68	UCRI_MOUSE	0.06897	0.11890	0.05427	0.11307	0.08393	0.14925	0.03908	0.03944	0.03259	0.03116	0.02408	0.02966
Q9CR86	CHSP1_MOUSE	0.00000	0.00000	0.00000	0.00000	0.00000	0.00000	0.00250	0.00705	0.00635	0.01028	0.01486	0.00621
Q9CR95	NECP1_MOUSE	0.00000	0.00000	0.00000	0.00000	0.00000	0.00000	0.01324	0.00524	0.00812	0.00784	0.00832	0.00369
Q9CRA0	NAR4_MOUSE	0.00000	0.00000	0.00000	0.00000	0.00000	0.00000	0.00000	0.00211	0.00369	0.00000	0.00094	0.00072
Q9CRA7	ATP5S_MOUSE	0.00000	0.00000	0.00000	0.00000	0.00000	0.00000	0.00000	0.00000	0.00000	0.00000	0.00000	0.00000
Q9CRB2	NHP2_MOUSE	0.00000	0.00000	0.00000	0.00000	0.00000	0.00000	0.00000	0.00000	0.00000	0.00000	0.00000	0.00000
Q9CRB6	TPPP3_MOUSE	0.00000	0.00000	0.00000	0.00000	0.00000	0.00000	0.16881	0.17261	0.13995	0.13314	0.13282	0.14410
Q9CRB8	MTFP1_MOUSE	0.00064	0.00056	0.00075	0.00130	0.00061	0.00079	0.00260	0.00000	0.00000	0.00000	0.00000	0.00000
Q9CRB9	MIC19_MOUSE	0.12889	0.10118	0.13418	0.10586	0.15580	0.14641	0.01583	0.02739	0.02424	0.02627	0.01628	0.02888
Q9CRC9	GNPI2_MOUSE	0.00000	0.00000	0.00000	0.00000	0.00000	0.00000	0.00431	0.00652	0.00838	0.00897	0.01158	0.00549
Q9CRD0	OCAD1_MOUSE	0.00396	0.00430	0.00229	0.00268	0.00316	0.00252	0.01850	0.01736	0.01308	0.01067	0.01359	0.01620
Q9CRD2	EMC2_MOUSE	0.00000	0.00000	0.00000	0.00000	0.00000	0.00000	0.00433	0.00732	0.00454	0.00453	0.00181	0.00212
Q9CRY7	GDPD1_MOUSE	0.00000	0.00000	0.00000	0.00000	0.00000	0.00000	0.01704	0.00990	0.01175	0.01214	0.01179	0.01566
Q9CS42	PRPS2_MOUSE	0.00000	0.00000	0.00000	0.00000	0.00000	0.00000	0.02013	0.02620	0.03155	0.02874	0.02522	0.02662
Q9CSN1	SNW1_MOUSE	0.00000	0.00000	0.00000	0.00000	0.00000	0.00000	0.00609	0.00758	0.00416	0.00519	0.00922	0.00570
Q9CSU0	RPR1B_MOUSE	0.00000	0.00000	0.00000	0.00000	0.00000	0.00000	0.00563	0.00424	0.00914	0.00794	0.00930	0.01019
Q9CT10	RANB3_MOUSE	0.00000	0.00000	0.00000	0.00000	0.00000	0.00000	0.00000	0.00184	0.00457	0.00461	0.01018	0.00636
Q9CU62	SMC1A_MOUSE	0.00000	0.00000	0.00000	0.00000	0.00000	0.00000	0.01721	0.01013	0.01418	0.00871	0.01276	0.00876
Q9CVB6	ARPC2_MOUSE	0.00000	0.00000	0.00000	0.00000	0.00000	0.00000	0.01007	0.02506	0.02942	0.02800	0.02497	0.02049
Q9CVD2	ATX3_MOUSE	0.00000	0.00000	0.00000	0.00000	0.00000	0.00000	0.00000	0.00128	0.00000	0.00000	0.00099	0.00083
Q9CW03	SMC3_MOUSE	0.00000	0.00000	0.00000	0.00000	0.00000	0.00000	0.00878	0.02101	0.01981	0.01394	0.01444	0.01840
Q9CW46	RAVR1_MOUSE	0.00000	0.00000	0.00000	0.00000	0.00000	0.00000	0.00230	0.00129	0.00448	0.00262	0.00505	0.00520
Q9CWD8	NUBPL_MOUSE	0.00092	0.00111	0.00174	0.00136	0.00200	0.00155	0.00122	0.00178	0.00064	0.00000	0.00000	0.00170
Q9CWE0	MFR1L_MOUSE	0.00000	0.00000	0.00000	0.00000	0.00000	0.00000	0.01914	0.00344	0.00590	0.00447	0.00474	0.00370
Q9CWJ9	PUR9_MOUSE	0.01579	0.01695	0.01661	0.01110	0.01185	0.01252	0.03393	0.01825	0.03140	0.02640	0.03722	0.03034
Q9CWK8	SNX2_MOUSE	0.00000	0.00000	0.00000	0.00000	0.00000	0.00000	0.02349	0.02556	0.03398	0.03233	0.02146	0.03220

Q9CWL8	CTBL1_MOUSE	0.00000	0.00000	0.00000	0.00000	0.00000	0.00000	0.00761	0.00064	0.00279	0.00162	0.00086	0.00201
Q9CWP6	MSPD2_MOUSE	0.00000	0.00000	0.00000	0.00000	0.00000	0.00000	0.00509	0.00721	0.00669	0.00874	0.00836	0.00833
Q9CWS0	DDAH1_MOUSE	0.00000	0.00000	0.00000	0.00000	0.00000	0.00000	0.01540	0.01462	0.01946	0.01585	0.01250	0.01420
Q9CWU6	UQCC1_MOUSE	0.00000	0.00000	0.00000	0.00000	0.00000	0.00000	0.00626	0.00331	0.00535	0.00436	0.00600	0.00436
Q9CWW6	PIN4_MOUSE	0.00000	0.00000	0.00000	0.00000	0.00000	0.00000	0.00457	0.00000	0.00663	0.00601	0.00376	0.00293
Q9CWZ3	RBM8A_MOUSE	0.00000	0.00000	0.00000	0.00000	0.00000	0.00000	0.00159	0.00826	0.00635	0.00987	0.01068	0.00722
Q9CWZ7	SNAG_MOUSE	0.00000	0.00000	0.00000	0.00000	0.00000	0.00000	0.00837	0.00697	0.00735	0.00564	0.00778	0.00439
Q9CX00	IST1_MOUSE	0.00000	0.00000	0.00000	0.00000	0.00000	0.00000	0.00000	0.00360	0.00530	0.00415	0.00214	0.00202
Q9CX13	CNIH4_MOUSE	0.00000	0.00000	0.00000	0.00000	0.00000	0.00000	0.00076	0.00677	0.00000	0.00152	0.00056	0.00267
Q9CX34	SGT1_MOUSE	0.00091	0.00136	0.00167	0.00124	0.00076	0.00104	0.00611	0.00823	0.01013	0.01072	0.00863	0.01110
Q9CX56	PSMD8_MOUSE	0.00000	0.00000	0.00000	0.00000	0.00000	0.00000	0.00455	0.00666	0.01069	0.01002	0.00977	0.00763
Q9CX60	LBH_MOUSE	0.00000	0.00000	0.00000	0.00000	0.00000	0.00000	0.00291	0.00102	0.01170	0.00029	0.01230	0.01352
Q9CX86	ROA0_MOUSE	0.00000	0.00000	0.00000	0.00000	0.00000	0.00000	0.03348	0.03557	0.02583	0.03056	0.02754	0.03182
Q9CX99	GRAP_MOUSE	0.00000	0.00000	0.00000	0.00000	0.00000	0.00000	0.00000	0.00303	0.00413	0.00000	0.00446	0.00000
Q9CXA2	T3HPD_MOUSE	0.00000	0.00000	0.00000	0.00000	0.00000	0.00000	0.00140	0.00330	0.00122	0.00290	0.00331	0.00186
Q9CXD6	MCUR1_MOUSE	0.00000	0.00000	0.00000	0.00000	0.00000	0.00000	0.00000	0.00000	0.00000	0.00000	0.00000	0.00000
Q9CXE7	TMED5_MOUSE	0.00000	0.00000	0.00000	0.00000	0.00000	0.00000	0.01100	0.01046	0.00996	0.00945	0.00582	0.01049
Q9CXI0	COQ5_MOUSE	0.01054	0.01650	0.02201	0.01294	0.01529	0.01268	0.00000	0.00146	0.00064	0.00000	0.00000	0.00000
Q9CXJ4	MITOS_MOUSE	0.00000	0.00000	0.00000	0.00000	0.00000	0.00000	0.00000	0.00000	0.00000	0.00000	0.00000	0.00000
Q9CXR1	DHRS7_MOUSE	0.00000	0.00000	0.00000	0.00000	0.00000	0.00000	0.01697	0.00681	0.00908	0.00579	0.01067	0.00907
Q9CXS4	CENPV_MOUSE	0.00292	0.00524	0.00133	0.00350	0.00237	0.00278	0.01635	0.01512	0.01694	0.01457	0.01507	0.01547
Q9CXT8	MPPB_MOUSE	0.00000	0.00000	0.00000	0.00000	0.00000	0.00000	0.00560	0.00357	0.00000	0.00815	0.00000	0.00000
Q9CXW3	CYBP_MOUSE	0.00000	0.00000	0.00000	0.00000	0.00000	0.00000	0.00473	0.01334	0.01315	0.01077	0.00885	0.00997
Q9CXW4	RL11_MOUSE	0.00000	0.00000	0.00000	0.00000	0.00000	0.00000	0.03426	0.04395	0.03188	0.03649	0.02595	0.04016
Q9CXY6	ILF2_MOUSE	0.00000	0.00000	0.00000	0.00000	0.00000	0.00000	0.01492	0.02148	0.02756	0.02334	0.01586	0.02224
Q9CXY9	GPI8_MOUSE	0.00000	0.00000	0.00000	0.00000	0.00000	0.00000	0.00000	0.00378	0.00000	0.00000	0.00027	0.00000
Q9CXZ1	NDUS4_MOUSE	0.00000	0.00000	0.00000	0.00000	0.00000	0.00000	0.00000	0.00678	0.00208	0.00277	0.00186	0.00595
Q9CY27	TECR_MOUSE	0.00000	0.00000	0.00000	0.00000	0.00000	0.00000	0.01793	0.01996	0.02636	0.02092	0.01772	0.01807
Q9CY50	SSRA_MOUSE	0.00000	0.00000	0.00000	0.00000	0.00000	0.00000	0.00308	0.00093	0.00697	0.00566	0.00628	0.00820
Q9CY57	CHTOP_MOUSE	0.00000	0.00000	0.00000	0.00000	0.00000	0.00000	0.00000	0.00492	0.00384	0.00221	0.00194	0.00371
Q9CY58	PAIRB_MOUSE	0.00596	0.01531	0.01032	0.01040	0.01019	0.01127	0.01420	0.01948	0.02505	0.01902	0.02097	0.02579
Q9CY62	RN181_MOUSE	0.00000	0.00000	0.00000	0.00000	0.00000	0.00000	0.00000	0.00000	0.00108	0.00000	0.00000	0.00051
Q9CY64	BIEA_MOUSE	0.00000	0.00000	0.00000	0.00000	0.00000	0.00000	0.01423	0.01070	0.02058	0.01275	0.01845	0.01686
Q9CY66	GAR1_MOUSE	0.00000	0.00000	0.00000	0.00000	0.00000	0.00000	0.00000	0.00136	0.00229	0.00109	0.00100	0.00102

Q9CY73	RM44_MOUSE	0.00142	0.00098	0.00191	0.00198	0.00167	0.00150	0.00113	0.00000	0.00000	0.00069	0.00000	0.00000
Q9CYA6	ZCHC8_MOUSE	0.00000	0.00000	0.00000	0.00000	0.00000	0.00000	0.00000	0.00322	0.00268	0.00239	0.00448	0.00531
Q9CYG7	TOM34_MOUSE	0.00000	0.00000	0.00000	0.00000	0.00000	0.00000	0.00952	0.00724	0.00793	0.00644	0.01099	0.01136
Q9CYH2	PXL2A_MOUSE	0.00000	0.00000	0.00000	0.00000	0.00000	0.00000	0.01344	0.00912	0.01019	0.00772	0.00957	0.01182
Q9CYI4	LUC7L_MOUSE	0.00000	0.00000	0.00000	0.00000	0.00000	0.00000	0.00000	0.00000	0.00151	0.00058	0.00000	0.00000
Q9CYL5	GAPR1_MOUSE	0.00000	0.00000	0.00000	0.00000	0.00000	0.00000	0.00000	0.00283	0.00000	0.00383	0.00400	0.00000
Q9CYN2	SPCS2_MOUSE	0.00000	0.00000	0.00000	0.00000	0.00000	0.00000	0.00829	0.01894	0.00586	0.00809	0.01134	0.00592
Q9CYR0	SSBP_MOUSE	0.00302	0.00119	0.00348	0.00338	0.00216	0.00110	0.00000	0.00468	0.00247	0.00322	0.00309	0.00232
Q9CYR6	AGM1_MOUSE	0.00279	0.00397	0.00212	0.00135	0.00187	0.00156	0.00133	0.00380	0.00574	0.00393	0.00203	0.00216
Q9CYW4	HDHD3_MOUSE	0.00000	0.00000	0.00000	0.00000	0.00000	0.00000	0.00355	0.00284	0.00214	0.00154	0.00236	0.00176
Q9CYZ2	TPD54_MOUSE	0.00179	0.00105	0.00107	0.00157	0.00263	0.00262	0.01893	0.01483	0.02842	0.02206	0.03384	0.02705
Q9CZ04	CSN7A_MOUSE	0.00280	0.00113	0.00374	0.00236	0.00188	0.00150	0.00609	0.00642	0.00743	0.00628	0.00625	0.00664
Q9CZ13	QCR1_MOUSE	0.32972	0.32948	0.39775	0.46259	0.43037	0.51319	0.06995	0.05241	0.05353	0.05768	0.04993	0.04579
Q9CZ28	SNF8_MOUSE	0.00000	0.00000	0.00000	0.00000	0.00000	0.00000	0.00000	0.00000	0.00798	0.00502	0.00543	0.00816
Q9CZ30	OLA1_MOUSE	0.00668	0.00148	0.00317	0.00208	0.00491	0.00201	0.01677	0.01285	0.01589	0.01413	0.01666	0.01462
Q9CZ42	NNRD_MOUSE	0.00199	0.00182	0.00268	0.00144	0.00137	0.00178	0.02196	0.03062	0.01265	0.02265	0.02441	0.02331
Q9CZ44	NSF1C_MOUSE	0.01225	0.00711	0.00603	0.00611	0.00709	0.00763	0.01788	0.02184	0.02686	0.02769	0.02596	0.02935
Q9CZ69	CKLF6_MOUSE	0.00000	0.00000	0.00000	0.00000	0.00000	0.00000	0.00000	0.00000	0.00000	0.00000	0.00190	0.00144
Q9CZB0	C560_MOUSE	0.01143	0.00559	0.02786	0.01809	0.00716	0.00335	0.01699	0.01106	0.01016	0.01071	0.01144	0.00968
Q9CZC8	SCRN1_MOUSE	0.00000	0.00000	0.00000	0.00000	0.00000	0.00000	0.00359	0.00627	0.01029	0.00802	0.00864	0.00898
Q9CZD3	GARS_MOUSE	0.00285	0.00376	0.00265	0.00170	0.00206	0.00306	0.00319	0.00650	0.01098	0.00232	0.01010	0.00866
Q9CZE3	RAB32_MOUSE	0.00000	0.00000	0.00000	0.00000	0.00000	0.00000	0.00373	0.00673	0.00442	0.00616	0.00646	0.00586
Q9CZH7	MXRA7_MOUSE	0.00000	0.00000	0.00000	0.00000	0.00000	0.00000	0.00000	0.01474	0.01543	0.01562	0.01196	0.00865
Q9CZJ2	HS12B_MOUSE	0.00000	0.00000	0.00000	0.00000	0.00000	0.00000	0.01882	0.02289	0.02191	0.01840	0.02427	0.02378
Q9CZM2	RL15_MOUSE	0.00469	0.00806	0.00890	0.00333	0.00501	0.00435	0.01915	0.02687	0.01952	0.01793	0.02431	0.02120
Q9CZN7	GLYM_MOUSE	0.00851	0.00409	0.00548	0.00190	0.00486	0.00674	0.00585	0.00362	0.00414	0.00357	0.00517	0.00332
Q9CZP5	BCS1_MOUSE	0.00038	0.00109	0.00171	0.00075	0.00015	0.00015	0.00736	0.00113	0.00323	0.00348	0.00063	0.00358
Q9CZR8	EFTS_MOUSE	0.00000	0.00000	0.00000	0.00000	0.00000	0.00000	0.00000	0.00000	0.00000	0.00000	0.00000	0.00000
Q9CZS1	AL1B1_MOUSE	0.00820	0.00904	0.00634	0.00929	0.01084	0.01217	0.00287	0.00512	0.00425	0.00665	0.00427	0.00382
Q9CZU3	MTREX_MOUSE	0.00000	0.00000	0.00000	0.00000	0.00000	0.00000	0.00187	0.00313	0.00434	0.00291	0.00388	0.00546
Q9CZU6	CISY_MOUSE	0.31501	0.39078	0.21453	0.60274	0.23487	0.50947	0.11487	0.09158	0.06208	0.06926	0.05686	0.06075
Q9CZW4	ACSL3_MOUSE	0.00000	0.00000	0.00000	0.00000	0.00000	0.00000	0.00623	0.00280	0.00660	0.00433	0.00408	0.00505
Q9CZW5	TOM70_MOUSE	0.00320	0.00375	0.00424	0.00888	0.00554	0.00585	0.01031	0.01092	0.01378	0.01202	0.01448	0.01204
Q9CZX8	RS19_MOUSE	0.00000	0.00000	0.00000	0.00000	0.00000	0.00000	0.03867	0.05015	0.04069	0.04486	0.03686	0.05171

Q9CZY3	UB2V1_MOUSE	0.00000	0.00000	0.00000	0.00000	0.00000	0.00000	0.02633	0.03516	0.02986	0.03288	0.02325	0.03505
Q9D020	5NT3A_MOUSE	0.03249	0.01420	0.02425	0.02848	0.04098	0.03166	0.00273	0.00133	0.00121	0.00122	0.00130	0.00108
Q9D023	MPC2_MOUSE	0.00000	0.00000	0.00000	0.00000	0.00000	0.00000	0.00429	0.00515	0.00875	0.00388	0.00452	0.00401
Q9D024	CCD47_MOUSE	0.00356	0.00139	0.00207	0.00218	0.00222	0.00260	0.01130	0.01154	0.00411	0.00456	0.00805	0.00540
Q9D051	ODPB_MOUSE	0.65452	0.46948	0.61874	0.46883	0.43865	0.50018	0.12420	0.10476	0.10275	0.09435	0.08843	0.10192
Q9D0A3	ARPIN_MOUSE	0.00000	0.00000	0.00000	0.00000	0.00000	0.00000	0.00000	0.00243	0.00386	0.00477	0.00465	0.00195
Q9D0B6	PBDC1_MOUSE	0.00000	0.00000	0.00000	0.00000	0.00000	0.00000	0.00447	0.00216	0.00449	0.00450	0.00227	0.00197
Q9D0E1	HNRPM_MOUSE	0.01104	0.01617	0.01322	0.01736	0.02048	0.02052	0.05288	0.04099	0.05256	0.04592	0.04622	0.05989
Q9D0F3	LMAN1_MOUSE	0.00493	0.00341	0.00449	0.00297	0.00301	0.00422	0.00924	0.01309	0.01678	0.01171	0.01315	0.01168
Q9D0F9	PGM1_MOUSE	0.08728	0.07524	0.08657	0.09158	0.07979	0.06926	0.02206	0.02605	0.02574	0.02841	0.00981	0.02617
Q9D0I9	SYRC_MOUSE	0.00299	0.00301	0.00411	0.00393	0.00280	0.00160	0.01465	0.01089	0.01114	0.01166	0.01037	0.01454
Q9D0J4	ARL2_MOUSE	0.00000	0.00000	0.00000	0.00000	0.00000	0.00000	0.00545	0.00135	0.00530	0.00370	0.00664	0.00424
Q9D0J8	PTMS_MOUSE	0.00000	0.00000	0.00000	0.00000	0.00000	0.00000	0.00000	0.03838	0.03830	0.04035	0.01587	0.01942
Q9D0K2	SCOT1_MOUSE	0.17316	0.19530	0.23213	0.13072	0.26752	0.22033	0.03209	0.04377	0.03197	0.02929	0.03035	0.03744
Q9D0L7	ARM10_MOUSE	0.00000	0.00000	0.00000	0.00000	0.00000	0.00000	0.00609	0.00729	0.00849	0.00728	0.00730	0.01064
Q9D0L8	MCES_MOUSE	0.00000	0.00000	0.00000	0.00000	0.00000	0.00000	0.00000	0.00000	0.00284	0.00230	0.00257	0.00407
Q9D0M1	KPRA_MOUSE	0.00000	0.00000	0.00000	0.00000	0.00000	0.00000	0.00348	0.00128	0.00467	0.00367	0.00370	0.00532
Q9D0M3	CY1_MOUSE	0.25791	0.17415	0.24272	0.28598	0.22717	0.13629	0.03719	0.02394	0.02655	0.03011	0.02916	0.02318
Q9D0R2	SYTC_MOUSE	0.00591	0.02238	0.00602	0.00403	0.00582	0.00547	0.02162	0.01781	0.02192	0.01523	0.02116	0.01865
Q9D0R8	LSM12_MOUSE	0.00000	0.00000	0.00000	0.00000	0.00000	0.00000	0.00000	0.00000	0.00208	0.00513	0.00162	0.00458
Q9D0S9	HINT2_MOUSE	0.00773	0.00377	0.01032	0.00695	0.00601	0.00457	0.02293	0.01635	0.02192	0.01841	0.02568	0.01774
Q9D0T1	NH2L1_MOUSE	0.00000	0.00000	0.00000	0.00000	0.00000	0.00000	0.00000	0.00606	0.00654	0.00749	0.00362	0.00486
Q9D0W5	PPIL1_MOUSE	0.00000	0.00000	0.00000	0.00000	0.00000	0.00000	0.00000	0.00437	0.00434	0.00589	0.00124	0.00117
Q9D154	ILEUA_MOUSE	0.00000	0.00000	0.00000	0.00000	0.00000	0.00000	0.03625	0.02388	0.03744	0.02908	0.03087	0.03259
Q9D172	GAL3A_MOUSE	0.13564	0.02880	0.14384	0.10906	0.08009	0.05198	0.03204	0.02169	0.02896	0.02558	0.03060	0.02211
Q9D1A2	CNDP2_MOUSE	0.00708	0.01052	0.00374	0.00665	0.00576	0.00702	0.03017	0.02953	0.03845	0.03218	0.03227	0.03359
Q9D1C8	VPS28_MOUSE	0.00000	0.00000	0.00000	0.00000	0.00000	0.00000	0.00359	0.01148	0.01182	0.00955	0.00543	0.00917
Q9D1D4	TMEDA_MOUSE	0.00083	0.00073	0.00241	0.00190	0.00242	0.00151	0.02424	0.04421	0.02855	0.03663	0.02753	0.04269
Q9D1G1	RAB1B_MOUSE	0.01435	0.01600	0.00862	0.01211	0.01584	0.01671	0.05800	0.04199	0.06722	0.06754	0.06598	0.06203
Q9D1G5	LRC57_MOUSE	0.00000	0.00000	0.00000	0.00000	0.00000	0.00000	0.00170	0.00274	0.00459	0.00287	0.00199	0.00414
Q9D1H6	NDUF4_MOUSE	0.00265	0.00100	0.00192	0.00165	0.00158	0.00170	0.00000	0.00000	0.00000	0.00104	0.00000	0.00000
Q9D1H9	MFAP4_MOUSE	0.00000	0.00000	0.00000	0.00000	0.00000	0.00000	0.22531	0.11431	0.24411	0.15808	0.27974	0.19046
Q9D1I5	MCEE_MOUSE	0.00000	0.00000	0.00000	0.00000	0.00000	0.00000	0.00078	0.00000	0.00128	0.00000	0.00000	0.00159
Q9D1I6	RM14_MOUSE	0.00000	0.00000	0.00000	0.00000	0.00000	0.00000	0.00000	0.00000	0.00000	0.00000	0.00000	0.00000

Q9D1J1	NECP2_MOUSE	0.00000	0.00000	0.00000	0.00000	0.00000	0.00000	0.01025	0.01204	0.01725	0.01351	0.01638	0.01565
Q9D1J3	SARNP_MOUSE	0.00000	0.00000	0.00000	0.00000	0.00000	0.00000	0.01208	0.01703	0.02364	0.02260	0.02054	0.01629
Q9D1K2	VATF_MOUSE	0.00000	0.00000	0.00000	0.00000	0.00000	0.00000	0.00648	0.00368	0.00000	0.00243	0.00132	0.00183
Q9D1L0	CHCH2_MOUSE	0.00808	0.00401	0.00889	0.00759	0.00552	0.00102	0.00000	0.00000	0.00108	0.00050	0.00181	0.00000
Q9D1L9	LTOR5_MOUSE	0.00000	0.00000	0.00000	0.00000	0.00000	0.00000	0.00868	0.00851	0.00950	0.00835	0.01180	0.00760
Q9D1M0	SEC13_MOUSE	0.00769	0.00536	0.00454	0.00541	0.00553	0.00456	0.00390	0.00690	0.01251	0.00953	0.01705	0.01160
Q9D1N2	HDBP1_MOUSE	0.00000	0.00000	0.00000	0.00000	0.00000	0.00000	0.00000	0.00460	0.00244	0.00159	0.00352	0.00323
Q9D1N9	RM21_MOUSE	0.00000	0.00000	0.00000	0.00000	0.00000	0.00000	0.00000	0.00000	0.00000	0.00000	0.00000	0.00000
Q9D1P0	RM13_MOUSE	0.00000	0.00000	0.00000	0.00000	0.00000	0.00000	0.03248	0.00000	0.00000	0.00000	0.01073	0.00000
Q9D1P4	CHRD1_MOUSE	0.00000	0.00000	0.00000	0.00000	0.00000	0.00000	0.00075	0.00214	0.00616	0.00304	0.00366	0.00204
Q9D1Q6	ERP44_MOUSE	0.00526	0.00200	0.00324	0.00357	0.00336	0.00417	0.00667	0.01561	0.01316	0.01271	0.00731	0.00992
Q9D1R9	RL34_MOUSE	0.00000	0.00000	0.00000	0.00000	0.00000	0.00000	0.00000	0.00574	0.00597	0.00520	0.00400	0.00248
Q9D1X0	NOL3_MOUSE	0.01432	0.01331	0.01975	0.01880	0.01681	0.01803	0.03331	0.00744	0.03101	0.02012	0.02421	0.02382
Q9D281	NXP20_MOUSE	0.00000	0.00000	0.00000	0.00000	0.00000	0.00000	0.00489	0.00847	0.01106	0.00474	0.00363	0.00694
Q9D287	SPF27_MOUSE	0.00000	0.00000	0.00000	0.00000	0.00000	0.00000	0.00138	0.00205	0.00386	0.00325	0.00126	0.00140
Q9D289	TPC6B_MOUSE	0.00000	0.00000	0.00000	0.00000	0.00000	0.00000	0.00034	0.00033	0.00117	0.00097	0.00101	0.00039
Q9D2G2	ODO2_MOUSE	0.00462	0.00590	0.00203	0.00926	0.00371	0.01658	0.10820	0.13324	0.09703	0.09966	0.12838	0.11619
Q9D2N4	DTNA_MOUSE	0.00530	0.00849	0.00846	0.00310	0.00394	0.00737	0.01894	0.00599	0.00768	0.00733	0.01187	0.01101
Q9D2N9	VP33A_MOUSE	0.00000	0.00000	0.00000	0.00000	0.00000	0.00000	0.00699	0.00218	0.00828	0.00333	0.00953	0.01206
Q9D2V7	CORO7_MOUSE	0.00000	0.00000	0.00000	0.00000	0.00000	0.00000	0.00499	0.00433	0.01377	0.00879	0.01578	0.01336
Q9D2V8	MFS10_MOUSE	0.00000	0.00000	0.00000	0.00000	0.00000	0.00000	0.00304	0.00182	0.00716	0.00401	0.00273	0.00144
Q9D358	PPAC_MOUSE	0.00000	0.00000	0.00000	0.00000	0.00000	0.00000	0.01370	0.01122	0.01337	0.01791	0.01110	0.01332
Q9D379	HYEP_MOUSE	0.00000	0.00000	0.00000	0.00000	0.00000	0.00000	0.03361	0.03064	0.03554	0.03390	0.03837	0.03039
Q9D394	RUFY3_MOUSE	0.00000	0.00000	0.00000	0.00000	0.00000	0.00000	0.00000	0.00000	0.00000	0.00000	0.00017	0.00000
Q9D3D9	ATPD_MOUSE	0.00000	0.00000	0.00000	0.00000	0.00000	0.00000	0.00535	0.02430	0.03453	0.02808	0.01564	0.01339
Q9D3E6	STAG1_MOUSE	0.00000	0.00000	0.00000	0.00000	0.00000	0.00000	0.00000	0.00000	0.00000	0.00098	0.00105	0.00000
Q9D3G5	STX11_MOUSE	0.00000	0.00000	0.00000	0.00000	0.00000	0.00000	0.00304	0.00735	0.00408	0.00553	0.00143	0.00671
Q9D404	OXSM_MOUSE	0.00684	0.00555	0.00569	0.00553	0.00677	0.00333	0.00000	0.00218	0.00266	0.00233	0.00179	0.00143
Q9D4H1	EXOC2_MOUSE	0.00000	0.00000	0.00000	0.00000	0.00000	0.00000	0.00566	0.00492	0.00647	0.00540	0.00778	0.00671
Q9D517	PLCC_MOUSE	0.00000	0.00000	0.00000	0.00000	0.00000	0.00000	0.00544	0.00344	0.00758	0.00561	0.00612	0.00491
Q9D554	SF3A3_MOUSE	0.00000	0.00000	0.00000	0.00000	0.00000	0.00000	0.00326	0.01017	0.00945	0.01023	0.00861	0.01155
Q9D5T0	ATAD1_MOUSE	0.00000	0.00000	0.00000	0.00000	0.00000	0.00000	0.00194	0.00274	0.00506	0.00386	0.00257	0.00367
Q9D5V5	CUL5_MOUSE	0.00297	0.00244	0.00406	0.00308	0.00553	0.00186	0.00278	0.00175	0.00353	0.00226	0.00426	0.00372
Q9D5V6	SYAP1_MOUSE	0.00152	0.00087	0.00052	0.00282	0.00158	0.00081	0.00000	0.00550	0.00577	0.00535	0.00186	0.00336

Q9D620	RFIP1_MOUSE	0.00000	0.00000	0.00000	0.00000	0.00000	0.00000	0.00319	0.00892	0.00613	0.00876	0.00806	0.01079
Q9D662	SC23B_MOUSE	0.00000	0.00000	0.00000	0.00000	0.00000	0.00000	0.00554	0.00585	0.00963	0.00609	0.01074	0.00826
Q9D666	SUN1_MOUSE	0.00000	0.00000	0.00000	0.00000	0.00000	0.00000	0.00599	0.00525	0.00564	0.00360	0.00634	0.00413
Q9D687	S6A19_MOUSE	0.00000	0.00000	0.00000	0.00000	0.00000	0.00000	0.00000	0.00009	0.00000	0.00000	0.00000	0.00000
Q9D6F9	TBB4A_MOUSE	0.00087	0.00055	0.00041	0.00052	0.00042	0.00065	0.01090	0.02643	0.02361	0.03441	0.03534	0.03834
Q9D6J5	NDUB8_MOUSE	0.08547	0.11697	0.12721	0.16703	0.10620	0.12106	0.01761	0.00853	0.01349	0.01063	0.00990	0.00972
Q9D6J6	NDUV2_MOUSE	0.18466	0.13835	0.16094	0.12669	0.15723	0.10753	0.00671	0.00174	0.00399	0.00471	0.00605	0.00522
Q9D6K5	SYJ2B_MOUSE	0.00000	0.00000	0.00000	0.00000	0.00000	0.00000	0.00330	0.00452	0.00692	0.00515	0.00607	0.00552
Q9D6K8	FUND2_MOUSE	0.00000	0.00000	0.00000	0.00000	0.00000	0.00000	0.00905	0.00851	0.00000	0.00000	0.00000	0.00000
Q9D6P8	CALL3_MOUSE	0.00000	0.00000	0.00000	0.00000	0.00000	0.00000	0.00274	0.00000	0.00000	0.00000	0.00000	0.00000
Q9D6R2	IDH3A_MOUSE	0.03587	0.02224	0.04943	0.02495	0.02585	0.01990	0.08213	0.06817	0.07718	0.07393	0.09673	0.07077
Q9D6S7	RRFM_MOUSE	0.00462	0.00386	0.00664	0.00427	0.00466	0.00611	0.00162	0.00572	0.00172	0.00311	0.00555	0.00435
Q9D6U8	F162A_MOUSE	0.00000	0.00000	0.00000	0.00000	0.00000	0.00000	0.01767	0.01835	0.01501	0.01898	0.01254	0.01679
Q9D6Y7	MSRA_MOUSE	0.00735	0.00380	0.00522	0.00888	0.00356	0.00363	0.00756	0.01201	0.01012	0.00846	0.01264	0.00945
Q9D6Y9	GLGB_MOUSE	0.00409	0.00539	0.00735	0.00638	0.00557	0.00314	0.00365	0.00525	0.00841	0.00419	0.00504	0.00365
Q9D6Z1	NOP56_MOUSE	0.00000	0.00000	0.00000	0.00000	0.00000	0.00000	0.00677	0.00779	0.00745	0.00699	0.00731	0.00947
Q9D706	RPAP3_MOUSE	0.00000	0.00000	0.00000	0.00000	0.00000	0.00000	0.00000	0.00000	0.00188	0.00139	0.00000	0.00000
Q9D708	S10AG_MOUSE	0.00000	0.00000	0.00000	0.00000	0.00000	0.00000	0.04170	0.02626	0.05641	0.07478	0.08365	0.02704
Q9D711	PIR_MOUSE	0.00000	0.00000	0.00000	0.00000	0.00000	0.00000	0.00000	0.00798	0.00482	0.00801	0.01070	0.00368
Q9D7A8	ARMC1_MOUSE	0.00204	0.00315	0.00424	0.00815	0.00732	0.00670	0.00131	0.00233	0.00162	0.00220	0.00190	0.00253
Q9D7B1	DUS2L_MOUSE	0.00000	0.00000	0.00000	0.00000	0.00000	0.00000	0.01375	0.00000	0.01328	0.01112	0.00718	0.00342
Q9D7B6	ACAD8_MOUSE	0.01834	0.02339	0.01108	0.01539	0.01359	0.03655	0.00335	0.00500	0.00223	0.00125	0.00067	0.00146
Q9D7E4	CS025_MOUSE	0.00000	0.00000	0.00000	0.00000	0.00000	0.00000	0.00000	0.00045	0.00153	0.00060	0.00101	0.00094
Q9D7G0	PRPS1_MOUSE	0.00000	0.00000	0.00000	0.00000	0.00000	0.00000	0.00474	0.00403	0.00582	0.00536	0.00540	0.00412
Q9D7J9	ECHD3_MOUSE	0.00000	0.00000	0.00000	0.00000	0.00000	0.00000	0.00127	0.00228	0.00248	0.00064	0.00000	0.00049
Q9D7L8	TMIG1_MOUSE	0.00000	0.00000	0.00000	0.00000	0.00000	0.00000	0.00000	0.01193	0.00000	0.00000	0.00000	0.00000
Q9D7M1	GID8_MOUSE	0.00000	0.00000	0.00000	0.00000	0.00000	0.00000	0.00280	0.00288	0.00251	0.00292	0.00377	0.00227
Q9D7N3	RT09_MOUSE	0.00213	0.00104	0.00336	0.00355	0.00166	0.00175	0.00093	0.00000	0.00000	0.00000	0.00090	0.00000
Q9D7N9	APMAP_MOUSE	0.00000	0.00000	0.00000	0.00000	0.00000	0.00000	0.01292	0.01916	0.01703	0.01640	0.01718	0.01606
Q9D7P7	CLIC3_MOUSE	0.00000	0.00000	0.00000	0.00000	0.00000	0.00000	0.03476	0.05415	0.06538	0.04764	0.04707	0.05225
Q9D7P9	SPB12_MOUSE	0.00000	0.00000	0.00000	0.00000	0.00000	0.00000	0.02860	0.00009	0.00000	0.00034	0.00000	0.00000
Q9D7S9	CHMP5_MOUSE	0.00000	0.00000	0.00000	0.00000	0.00000	0.00000	0.00771	0.01154	0.01032	0.01067	0.01532	0.02194
Q9D7V9	NAAA_MOUSE	0.00000	0.00000	0.00000	0.00000	0.00000	0.00000	0.00000	0.00089	0.00512	0.00281	0.00154	0.00144
Q9D7X3	DUS3_MOUSE	0.01779	0.00853	0.01414	0.00899	0.01025	0.01033	0.02552	0.02099	0.02402	0.02419	0.02184	0.02381

Q9D819	IPYR_MOUSE	0.01447	0.00926	0.01308	0.00897	0.01301	0.01682	0.03442	0.01965	0.02059	0.02212	0.02381	0.01704
Q9D824	FIP1_MOUSE	0.00000	0.00000	0.00000	0.00000	0.00000	0.00000	0.00138	0.00000	0.00156	0.00381	0.00171	0.00182
Q9D826	SOX_MOUSE	0.00000	0.00000	0.00000	0.00000	0.00000	0.00000	0.00007	0.00016	0.00000	0.00000	0.00005	0.00004
Q9D832	DNJB4_MOUSE	0.00560	0.00359	0.00453	0.00246	0.00554	0.00392	0.01349	0.00842	0.01251	0.01210	0.01939	0.01309
Q9D855	QCR7_MOUSE	0.00000	0.00000	0.00000	0.00000	0.00000	0.00000	0.04131	0.02221	0.02629	0.02488	0.02184	0.01988
Q9D880	TIM50_MOUSE	0.00958	0.01582	0.01148	0.00997	0.01462	0.00713	0.01916	0.00599	0.01081	0.00720	0.01164	0.00432
Q9D883	U2AF1_MOUSE	0.00000	0.00000	0.00000	0.00000	0.00000	0.00000	0.00109	0.00354	0.00502	0.00514	0.00284	0.00668
Q9D892	ITPA_MOUSE	0.00000	0.00000	0.00000	0.00000	0.00000	0.00000	0.01714	0.01811	0.01866	0.01630	0.01777	0.01927
Q9D898	ARP5L_MOUSE	0.00000	0.00000	0.00000	0.00000	0.00000	0.00000	0.00659	0.00418	0.01090	0.00693	0.01054	0.00718
Q9D8B3	CHM4B_MOUSE	0.00000	0.00000	0.00000	0.00000	0.00000	0.00000	0.00000	0.01458	0.02241	0.01683	0.00588	0.00759
Q9D8B4	NDUAB_MOUSE	0.00000	0.00000	0.00000	0.00000	0.00000	0.00000	0.01507	0.01674	0.01607	0.01332	0.01356	0.01641
Q9D8E6	RL4_MOUSE	0.01454	0.02655	0.00656	0.01439	0.00857	0.01727	0.04132	0.04008	0.03265	0.03358	0.03051	0.04286
Q9D8N0	EF1G_MOUSE	0.02559	0.01324	0.03096	0.03323	0.01361	0.02244	0.03413	0.03363	0.03660	0.03507	0.02971	0.03382
Q9D8N2	DEN10_MOUSE	0.00000	0.00000	0.00000	0.00000	0.00000	0.00000	0.00077	0.00082	0.00050	0.00000	0.00060	0.00149
Q9D8S3	ARFG3_MOUSE	0.00000	0.00000	0.00000	0.00000	0.00000	0.00000	0.00740	0.00765	0.00737	0.00734	0.00633	0.00961
Q9D8T2	GSDMD_MOUSE	0.00000	0.00000	0.00000	0.00000	0.00000	0.00000	0.00000	0.00629	0.00404	0.00384	0.00227	0.00319
Q9D8T7	SLIRP_MOUSE	0.00000	0.00000	0.00000	0.00000	0.00000	0.00000	0.00000	0.00000	0.00000	0.00000	0.00000	0.00000
Q9D8U8	SNX5_MOUSE	0.00000	0.00000	0.00000	0.00000	0.00000	0.00000	0.02725	0.02734	0.01028	0.02097	0.00886	0.02287
Q9D8V0	HM13_MOUSE	0.00000	0.00000	0.00000	0.00000	0.00000	0.00000	0.01252	0.01264	0.01234	0.01297	0.01262	0.01576
Q9D8W5	PSD12_MOUSE	0.00493	0.00662	0.00582	0.00480	0.00639	0.00472	0.02823	0.03547	0.02300	0.03087	0.02721	0.02738
Q9D8X1	CUTC_MOUSE	0.00000	0.00000	0.00000	0.00000	0.00000	0.00000	0.00000	0.00000	0.00115	0.00029	0.00000	0.00000
Q9D8Y0	EFHD2_MOUSE	0.00000	0.00000	0.00000	0.00000	0.00000	0.00000	0.00397	0.01472	0.01135	0.02074	0.01281	0.01154
Q9D8Y7	TP8L2_MOUSE	0.00000	0.00000	0.00000	0.00000	0.00000	0.00000	0.00024	0.00000	0.00191	0.00000	0.00364	0.00060
Q9D906	ATG7_MOUSE	0.00000	0.00000	0.00000	0.00000	0.00000	0.00000	0.00000	0.00454	0.00586	0.00473	0.00452	0.00212
Q9D939	ST1C2_MOUSE	0.00000	0.00000	0.00000	0.00000	0.00000	0.00000	0.00000	0.00000	0.00051	0.00015	0.00032	0.00000
Q9D952	EVPL_MOUSE	0.00000	0.00000	0.00000	0.00000	0.00000	0.00000	0.01290	0.00871	0.00974	0.01153	0.02260	0.00394
Q9D958	SPCS1_MOUSE	0.00000	0.00000	0.00000	0.00000	0.00000	0.00000	0.00636	0.00483	0.00604	0.00613	0.00242	0.00560
Q9D964	GATM_MOUSE	0.00000	0.00000	0.00000	0.00000	0.00000	0.00000	0.00010	0.00010	0.00006	0.00008	0.00003	0.00008
Q9D967	MGDP1_MOUSE	0.00744	0.02270	0.00717	0.00490	0.00596	0.00603	0.00187	0.00345	0.00504	0.00355	0.00216	0.00289
Q9D9V3	ECHD1_MOUSE	0.00177	0.00151	0.00179	0.00323	0.00133	0.00150	0.01124	0.00117	0.00876	0.00285	0.00098	0.00328
Q9DAI2	IFT22_MOUSE	0.00000	0.00000	0.00000	0.00000	0.00000	0.00000	0.00047	0.00057	0.00205	0.00127	0.00287	0.00143
Q9DAK9	PHP14_MOUSE	0.00000	0.00000	0.00000	0.00000	0.00000	0.00000	0.00000	0.00786	0.01058	0.01040	0.00334	0.00405
Q9DAM5	TPC_MOUSE	0.00000	0.00000	0.00000	0.00000	0.00000	0.00000	0.00000	0.00000	0.00000	0.00000	0.00000	0.00000
Q9DAR7	DCPS_MOUSE	0.00000	0.00000	0.00000	0.00000	0.00000	0.00000	0.01802	0.01134	0.02313	0.01836	0.02812	0.02272

Q9DAS9	GBG12_MOUSE	0.00418	0.00201	0.00267	0.00190	0.00297	0.00415	0.06133	0.03608	0.03553	0.03776	0.03190	0.05832
Q9DAT5	MTU1_MOUSE	0.00000	0.00000	0.00000	0.00000	0.00000	0.00000	0.00000	0.00000	0.00000	0.00000	0.00000	0.00000
Q9DAU1	CNPY3_MOUSE	0.00000	0.00000	0.00000	0.00000	0.00000	0.00000	0.00000	0.00266	0.00511	0.00471	0.00200	0.00170
Q9DAV9	TM38B_MOUSE	0.00000	0.00000	0.00000	0.00000	0.00000	0.00000	0.00435	0.00491	0.00081	0.00160	0.00000	0.00000
Q9DAW9	CNN3_MOUSE	0.00000	0.00000	0.00000	0.00000	0.00000	0.00000	0.02090	0.04061	0.02590	0.01885	0.03049	0.03207
Q9DB05	SNAA_MOUSE	0.00000	0.00000	0.00000	0.00000	0.00000	0.00000	0.00376	0.01485	0.01865	0.01380	0.01133	0.01189
Q9DB15	RM12_MOUSE	0.00000	0.00000	0.00000	0.00000	0.00000	0.00000	0.00000	0.00000	0.00000	0.00000	0.00000	0.00000
Q9DB20	ATPO_MOUSE	0.07947	0.11811	0.29598	0.19245	0.12623	0.10601	0.02060	0.02934	0.01749	0.02488	0.02776	0.03782
Q9DB26	PHYD1_MOUSE	0.00261	0.00078	0.00271	0.00170	0.00266	0.00103	0.00000	0.00084	0.00314	0.00156	0.00191	0.00106
Q9DB29	IAH1_MOUSE	0.00360	0.00459	0.00287	0.00356	0.00314	0.00237	0.01858	0.01596	0.01150	0.01422	0.00439	0.01905
Q9DB73	NB5R1_MOUSE	0.00000	0.00000	0.00000	0.00000	0.00000	0.00000	0.00000	0.00559	0.00352	0.00891	0.00329	0.00524
Q9DB77	QCR2_MOUSE	1.71037	1.40410	1.57563	1.23060	1.25827	1.15269	0.08464	0.06611	0.07770	0.07207	0.06892	0.06162
Q9DBB8	DHDH_MOUSE	0.00076	0.00077	0.00127	0.00044	0.00118	0.00094	0.00825	0.00798	0.01399	0.00808	0.00791	0.00969
Q9DBB9	CPN2_MOUSE	0.00000	0.00000	0.00000	0.00000	0.00000	0.00000	0.00511	0.00561	0.00599	0.00483	0.00477	0.00455
Q9DBC3	CMTR1_MOUSE	0.00000	0.00000	0.00000	0.00000	0.00000	0.00000	0.00000	0.00000	0.00058	0.00024	0.00000	0.00000
Q9DBC7	KAP0_MOUSE	0.01776	0.01031	0.00655	0.01284	0.01174	0.01573	0.02983	0.03327	0.04432	0.03693	0.03262	0.03916
Q9DBD0	ICA_MOUSE	0.00000	0.00000	0.00000	0.00000	0.00000	0.00000	0.00445	0.00325	0.00612	0.00598	0.00864	0.00533
Q9DBE0	CSAD_MOUSE	0.00000	0.00000	0.00000	0.00000	0.00000	0.00000	0.00500	0.00669	0.00505	0.00329	0.00370	0.00386
Q9DBE8	ALG2_MOUSE	0.00000	0.00000	0.00000	0.00000	0.00000	0.00000	0.00437	0.00747	0.00627	0.00443	0.00663	0.00819
Q9DBF1	AL7A1_MOUSE	0.04411	0.01414	0.01354	0.01097	0.01153	0.01049	0.02460	0.02989	0.03267	0.02827	0.04041	0.02569
Q9DBG3	AP2B1_MOUSE	0.00053	0.00091	0.00030	0.00041	0.00036	0.00037	0.02794	0.03629	0.03240	0.03536	0.02711	0.03832
Q9DBG5	PLIN3_MOUSE	0.01115	0.00861	0.00736	0.01011	0.00738	0.01445	0.01332	0.01159	0.01663	0.01549	0.01845	0.01786
Q9DBG6	RPN2_MOUSE	0.00492	0.00573	0.00391	0.00253	0.00351	0.00448	0.04327	0.03309	0.04374	0.03329	0.04089	0.03690
Q9DBG9	TX1B3_MOUSE	0.00000	0.00000	0.00000	0.00000	0.00000	0.00000	0.00922	0.00719	0.00403	0.00804	0.00333	0.01036
Q9DBH5	LMAN2_MOUSE	0.00000	0.00000	0.00000	0.00000	0.00000	0.00000	0.01834	0.01453	0.02144	0.01932	0.02104	0.01487
Q9DBJ1	PGAM1_MOUSE	0.07872	0.06457	0.07242	0.07011	0.08430	0.07147	0.05798	0.04684	0.07759	0.06110	0.07083	0.05601
Q9DBJ3	BI2L1_MOUSE	0.00000	0.00000	0.00000	0.00000	0.00000	0.00000	0.01204	0.00463	0.00554	0.00338	0.00509	0.00410
Q9DBK0	ACO12_MOUSE	0.00000	0.00000	0.00000	0.00000	0.00000	0.00000	0.00000	0.00000	0.00000	0.00000	0.00000	0.00000
Q9DBL1	ACDSB_MOUSE	0.02015	0.00744	0.02264	0.01110	0.01619	0.01366	0.00452	0.01289	0.00984	0.00649	0.01089	0.01376
Q9DBL7	COASY_MOUSE	0.00000	0.00000	0.00000	0.00000	0.00000	0.00000	0.00018	0.00021	0.00193	0.00000	0.00009	0.00036
Q9DBL9	ABHD5_MOUSE	0.00000	0.00000	0.00000	0.00000	0.00000	0.00000	0.00150	0.00000	0.00160	0.00000	0.00000	0.00000
Q9DBM2	ECHP_MOUSE	0.00000	0.00000	0.00000	0.00000	0.00000	0.00000	0.00072	0.00064	0.00081	0.00035	0.00078	0.00068
Q9DBN5	LONP2_MOUSE	0.00000	0.00000	0.00000	0.00000	0.00000	0.00000	0.00000	0.00000	0.00000	0.00000	0.00000	0.00000
Q9DBP0	NPT2B_MOUSE	0.00000	0.00000	0.00000	0.00000	0.00000	0.00000	0.00000	0.00270	0.00729	0.00608	0.00225	0.00422

Q9DBP5	KCY_MOUSE	0.00000	0.00000	0.00000	0.00000	0.00000	0.00000	0.02362	0.02348	0.02305	0.02339	0.01671	0.02098
Q9DBR1	XRN2_MOUSE	0.00000	0.00000	0.00000	0.00000	0.00000	0.00000	0.00000	0.00187	0.00309	0.00248	0.00253	0.00114
Q9DBR3	ARMC8_MOUSE	0.00000	0.00000	0.00000	0.00000	0.00000	0.00000	0.00000	0.00098	0.00329	0.00246	0.00360	0.00299
Q9DBR4	APBB2_MOUSE	0.00000	0.00000	0.00000	0.00000	0.00000	0.00000	0.00000	0.00290	0.00000	0.00000	0.00000	0.00026
Q9DBR7	MYPT1_MOUSE	0.00219	0.00186	0.00219	0.00286	0.00235	0.00226	0.00833	0.01627	0.01653	0.01816	0.01141	0.01582
Q9DBS1	TMM43_MOUSE	0.00000	0.00000	0.00000	0.00000	0.00000	0.00000	0.02087	0.01761	0.02182	0.01926	0.02015	0.01819
Q9DBS2	TPRGL_MOUSE	0.00000	0.00000	0.00000	0.00000	0.00000	0.00000	0.00769	0.00394	0.00651	0.00930	0.00749	0.00668
Q9DBS5	KLC4_MOUSE	0.00000	0.00000	0.00000	0.00000	0.00000	0.00000	0.00000	0.00000	0.00574	0.00347	0.00294	0.00206
Q9DBS9	OSBL3_MOUSE	0.00000	0.00000	0.00000	0.00000	0.00000	0.00000	0.00000	0.00210	0.00120	0.00346	0.00124	0.00135
Q9DBT9	M2GD_MOUSE	0.00000	0.00000	0.00000	0.00000	0.00000	0.00000	0.00009	0.00005	0.00004	0.00015	0.00005	0.00000
Q9DBV4	MXRA8_MOUSE	0.00000	0.00000	0.00000	0.00000	0.00000	0.00000	0.00000	0.00344	0.00490	0.00370	0.00278	0.00152
Q9DBX1	RGCC_MOUSE	0.00000	0.00000	0.00000	0.00000	0.00000	0.00000	0.01754	0.01565	0.01735	0.01393	0.02097	0.02241
Q9DBX3	SUSD2_MOUSE	0.00000	0.00000	0.00000	0.00000	0.00000	0.00000	0.00000	0.00012	0.00138	0.00109	0.00020	0.00031
Q9DBX6	CP2S1_MOUSE	0.00000	0.00000	0.00000	0.00000	0.00000	0.00000	0.00986	0.01014	0.01512	0.01229	0.01000	0.01016
Q9DBZ5	EIF3K_MOUSE	0.00000	0.00000	0.00000	0.00000	0.00000	0.00000	0.00906	0.01259	0.00269	0.00623	0.00876	0.00577
Q9DC07	LNEBL_MOUSE	0.01694	0.01018	0.00870	0.00354	0.01234	0.00863	0.07201	0.08743	0.07392	0.08426	0.05760	0.10827
Q9DC11	PXDC2_MOUSE	0.00000	0.00000	0.00000	0.00000	0.00000	0.00000	0.00745	0.01499	0.01559	0.01851	0.01687	0.01717
Q9DC16	ERGI1_MOUSE	0.00000	0.00000	0.00000	0.00000	0.00000	0.00000	0.00483	0.00351	0.00416	0.00397	0.00512	0.00561
Q9DC23	DJC10_MOUSE	0.00000	0.00000	0.00000	0.00000	0.00000	0.00000	0.00559	0.00573	0.00000	0.00000	0.00000	0.00000
Q9DC50	OCTC_MOUSE	0.00000	0.00000	0.00000	0.00000	0.00000	0.00000	0.00000	0.00013	0.00014	0.00000	0.00007	0.00014
Q9DC51	GNAI3_MOUSE	0.00880	0.00646	0.00631	0.00451	0.00678	0.00330	0.02150	0.02421	0.01269	0.01908	0.01088	0.01666
Q9DC61	MPPA_MOUSE	0.00466	0.00396	0.00367	0.00397	0.00400	0.00240	0.00000	0.00000	0.00091	0.00000	0.00000	0.00000
Q9DC69	NDUA9_MOUSE	0.28109	0.40636	0.40682	0.39193	0.49617	0.40665	0.01329	0.00395	0.01003	0.00723	0.00923	0.00786
Q9DC70	NDUS7_MOUSE	0.00000	0.00000	0.00000	0.00000	0.00000	0.00000	0.01845	0.01644	0.01076	0.01251	0.00652	0.01169
Q9DCD0	6PGD_MOUSE	0.00876	0.00613	0.00545	0.00543	0.00565	0.00744	0.10057	0.06347	0.09163	0.07793	0.10237	0.07197
Q9DCD6	GBRAP_MOUSE	0.00000	0.00000	0.00000	0.00000	0.00000	0.00000	0.00000	0.00154	0.00335	0.00268	0.00132	0.00098
Q9DCF9	SSRG_MOUSE	0.00000	0.00000	0.00000	0.00000	0.00000	0.00000	0.01798	0.01435	0.01000	0.01403	0.00753	0.01572
Q9DCG6	PBLD1_MOUSE	0.00000	0.00000	0.00000	0.00000	0.00000	0.00000	0.00000	0.00000	0.00000	0.00000	0.00000	0.00000
Q9DCG9	TR112_MOUSE	0.00000	0.00000	0.00000	0.00000	0.00000	0.00000	0.00252	0.00346	0.00119	0.00363	0.00000	0.00388
Q9DCH4	EIF3F_MOUSE	0.00000	0.00000	0.00000	0.00000	0.00000	0.00000	0.00395	0.00764	0.00755	0.00651	0.00520	0.00749
Q9DCJ5	NDUA8_MOUSE	0.02606	0.01448	0.03814	0.03089	0.02544	0.01446	0.03042	0.01445	0.01155	0.01552	0.01543	0.01277
Q9DCJ9	NPL_MOUSE	0.00000	0.00000	0.00000	0.00000	0.00000	0.00000	0.00000	0.00124	0.00000	0.00000	0.00000	0.00000
Q9DCL8	IPP2_MOUSE	0.00000	0.00000	0.00000	0.00000	0.00000	0.00000	0.00626	0.01385	0.00530	0.00781	0.00634	0.00644
Q9DCL9	PUR6_MOUSE	0.02241	0.01773	0.02621	0.02023	0.02300	0.02596	0.02787	0.03314	0.01731	0.03317	0.02232	0.02583

Q9DCM0	ETHE1_MOUSE	0.00729	0.00127	0.00422	0.00373	0.00415	0.00391	0.00143	0.00385	0.00546	0.00530	0.00444	0.00387
Q9DCM2	GSTK1_MOUSE	0.06582	0.06002	0.05946	0.05112	0.05586	0.04211	0.04328	0.04173	0.02840	0.03056	0.04673	0.04094
Q9DCN1	NUD12_MOUSE	0.00000	0.00000	0.00000	0.00000	0.00000	0.00000	0.00105	0.00029	0.00172	0.00069	0.00079	0.00125
Q9DCN2	NB5R3_MOUSE	0.02086	0.00647	0.01051	0.01348	0.01566	0.01263	0.14302	0.11733	0.13773	0.13100	0.13841	0.13185
Q9DCQ2	ASPD_MOUSE	0.00000	0.00000	0.00000	0.00000	0.00000	0.00000	0.00000	0.00000	0.00000	0.00000	0.00000	0.00000
Q9DCR2	AP3S1_MOUSE	0.00000	0.00000	0.00000	0.00000	0.00000	0.00000	0.00000	0.00316	0.00370	0.00462	0.00317	0.00358
Q9DCS2	MTL26_MOUSE	0.00000	0.00000	0.00000	0.00000	0.00000	0.00000	0.00000	0.00169	0.00648	0.00381	0.00268	0.00000
Q9DCS3	MECR_MOUSE	0.00981	0.00834	0.00716	0.01083	0.00820	0.00762	0.00133	0.00043	0.00124	0.00237	0.00133	0.00106
Q9DCS9	NDUBA_MOUSE	0.15917	0.12687	0.16865	0.15052	0.20901	0.20978	0.02599	0.01167	0.01545	0.01779	0.01091	0.01444
Q9DCT1	AKCL2_MOUSE	0.00247	0.00375	0.00408	0.00425	0.00219	0.00366	0.00344	0.00269	0.00102	0.00126	0.00398	0.00413
Q9DCT2	NDUS3_MOUSE	0.31617	0.31304	0.39226	0.20760	0.42836	0.36080	0.02295	0.01559	0.01913	0.01571	0.01472	0.01629
Q9DCT5	SDF2_MOUSE	0.00000	0.00000	0.00000	0.00000	0.00000	0.00000	0.00000	0.00000	0.00000	0.00000	0.00000	0.00000
Q9DCT8	CRIP2_MOUSE	0.04729	0.01988	0.06862	0.04853	0.04858	0.02672	0.47558	0.54561	0.38838	0.43883	0.30327	0.51172
Q9DCU2	PLLP_MOUSE	0.00000	0.00000	0.00000	0.00000	0.00000	0.00000	0.00000	0.00355	0.00338	0.00395	0.00351	0.00264
Q9DCU6	RM04_MOUSE	0.00000	0.00000	0.00000	0.00000	0.00000	0.00000	0.00000	0.00000	0.00000	0.00000	0.00000	0.00000
Q9DCU9	HOGA1_MOUSE	0.00000	0.00000	0.00000	0.00000	0.00000	0.00000	0.00000	0.00000	0.00000	0.00000	0.00273	0.00000
Q9DCV4	RMD1_MOUSE	0.02391	0.02956	0.01176	0.01555	0.02112	0.03154	0.01295	0.00799	0.00856	0.01001	0.01016	0.01025
Q9DCW4	ETFB_MOUSE	0.52728	0.85506	0.77440	0.88818	0.73537	0.72821	0.04638	0.04461	0.03046	0.03730	0.03276	0.03400
Q9DCX2	ATP5H_MOUSE	0.74174	0.34923	0.71042	0.50435	0.55281	0.37263	0.02653	0.03560	0.04350	0.04113	0.03370	0.03852
Q9DCX8	IYD1_MOUSE	0.00000	0.00000	0.00000	0.00000	0.00000	0.00000	0.00000	0.00000	0.00000	0.00000	0.00000	0.00000
Q9DCY0	KEG1_MOUSE	0.00000	0.00000	0.00000	0.00000	0.00000	0.00000	0.00021	0.00039	0.00001	0.00028	0.00018	0.00013
Q9DCZ4	MIC26_MOUSE	0.03129	0.03817	0.01769	0.03769	0.01753	0.04209	0.01189	0.01642	0.00577	0.01125	0.00876	0.01015
Q9DD02	HIKES_MOUSE	0.00000	0.00000	0.00000	0.00000	0.00000	0.00000	0.00000	0.00200	0.00214	0.00190	0.00088	0.00092
Q9DD06	RARR2_MOUSE	0.00000	0.00000	0.00000	0.00000	0.00000	0.00000	0.00082	0.00250	0.00236	0.00233	0.00369	0.00349
Q9DD18	DTD1_MOUSE	0.00000	0.00000	0.00000	0.00000	0.00000	0.00000	0.00000	0.00241	0.00420	0.00354	0.00187	0.00139
Q9DD20	MET7B_MOUSE	0.00000	0.00000	0.00000	0.00000	0.00000	0.00000	0.00000	0.00000	0.00000	0.00000	0.00000	0.00000
Q9EP69	SAC1_MOUSE	0.00756	0.00263	0.00464	0.00641	0.00240	0.00283	0.01756	0.01286	0.01823	0.01543	0.01940	0.01597
Q9EP71	RAI14_MOUSE	0.00000	0.00000	0.00000	0.00000	0.00000	0.00000	0.00889	0.00882	0.00887	0.00939	0.00641	0.00899
Q9EP72	EMC7_MOUSE	0.00000	0.00000	0.00000	0.00000	0.00000	0.00000	0.00000	0.00125	0.00264	0.00079	0.00116	0.00058
Q9EP89	LACTB_MOUSE	0.00215	0.00289	0.00245	0.00274	0.00226	0.00116	0.00190	0.00267	0.00615	0.00478	0.00286	0.00032
Q9EPB4	ASC_MOUSE	0.00000	0.00000	0.00000	0.00000	0.00000	0.00000	0.00250	0.00000	0.00126	0.00106	0.00269	0.00150
Q9EPB5	SERHL_MOUSE	0.00236	0.00164	0.00285	0.00336	0.00189	0.00152	0.00077	0.00049	0.00076	0.00031	0.00000	0.00026
Q9EPC1	PARVA_MOUSE	0.00356	0.02483	0.00784	0.01016	0.00542	0.00899	0.04769	0.06268	0.05655	0.06249	0.06386	0.06113
Q9EPJ9	ARFG1_MOUSE	0.00000	0.00000	0.00000	0.00000	0.00000	0.00000	0.00146	0.00319	0.00197	0.00423	0.00073	0.00485

Q9EPK2	XRP2_MOUSE	0.00000	0.00000	0.00000	0.00000	0.00000	0.00000	0.00468	0.00565	0.00627	0.00510	0.00511	0.00639
Q9EPK8	TRPV4_MOUSE	0.00000	0.00000	0.00000	0.00000	0.00000	0.00000	0.00000	0.00000	0.00000	0.00000	0.00000	0.00000
Q9EPL8	IPO7_MOUSE	0.00000	0.00000	0.00000	0.00000	0.00000	0.00000	0.00832	0.01244	0.00735	0.00891	0.01088	0.01065
Q9EPL9	ACOX3_MOUSE	0.00000	0.00000	0.00000	0.00000	0.00000	0.00000	0.00196	0.00214	0.00647	0.00617	0.00490	0.00698
Q9EPQ2	RPGR1_MOUSE	0.00000	0.00000	0.00000	0.00000	0.00000	0.00000	0.00217	0.02591	0.00305	0.00573	0.00000	0.00959
Q9EPQ7	STAR5_MOUSE	0.00000	0.00000	0.00000	0.00000	0.00000	0.00000	0.00084	0.00051	0.00049	0.00053	0.00034	0.00038
Q9EPT5	SO2A1_MOUSE	0.00000	0.00000	0.00000	0.00000	0.00000	0.00000	0.02187	0.05107	0.04452	0.03366	0.04690	0.05052
Q9EPU0	RENT1_MOUSE	0.00310	0.00315	0.00267	0.00221	0.00252	0.00230	0.01107	0.00866	0.01050	0.01038	0.01204	0.01075
Q9EPU4	CPSF1_MOUSE	0.00000	0.00000	0.00000	0.00000	0.00000	0.00000	0.00000	0.00000	0.00102	0.00112	0.00000	0.00104
Q9EQ06	DHB11_MOUSE	0.00145	0.00161	0.00226	0.00204	0.00169	0.00084	0.01231	0.03186	0.01622	0.02234	0.02642	0.02566
Q9EQ20	MMSA_MOUSE	0.31744	0.25839	0.28782	0.22326	0.32025	0.27328	0.08128	0.08712	0.06810	0.05709	0.06869	0.07534
Q9EQ32	BCAP_MOUSE	0.00000	0.00000	0.00000	0.00000	0.00000	0.00000	0.00000	0.00000	0.00147	0.00056	0.00052	0.00096
Q9EQ80	NIF3L_MOUSE	0.00000	0.00000	0.00000	0.00000	0.00000	0.00000	0.00245	0.00367	0.00238	0.00274	0.00424	0.00335
Q9EQF6	DPYL5_MOUSE	0.00000	0.00000	0.00000	0.00000	0.00000	0.00000	0.00359	0.00576	0.00490	0.00491	0.00628	0.00451
Q9EQG3	SCEL_MOUSE	0.00000	0.00000	0.00000	0.00000	0.00000	0.00000	0.00313	0.00307	0.00514	0.00454	0.00686	0.00310
Q9EQG7	ENPP5_MOUSE	0.00000	0.00000	0.00000	0.00000	0.00000	0.00000	0.00000	0.00000	0.00117	0.00235	0.00000	0.00000
Q9EQG9	CERT_MOUSE	0.00000	0.00000	0.00000	0.00000	0.00000	0.00000	0.00298	0.00347	0.00158	0.00359	0.00118	0.00232
Q9EQH2	ERAP1_MOUSE	0.00000	0.00000	0.00000	0.00000	0.00000	0.00000	0.00318	0.00563	0.00915	0.00695	0.00845	0.00798
Q9EQH3	VPS35_MOUSE	0.01531	0.02108	0.02805	0.01633	0.01172	0.02249	0.01915	0.02846	0.02731	0.02554	0.02914	0.02412
Q9EQJ9	MAGI3_MOUSE	0.00000	0.00000	0.00000	0.00000	0.00000	0.00000	0.00169	0.00642	0.01035	0.00480	0.01183	0.00613
Q9EQK5	MVP_MOUSE	0.00868	0.01102	0.01162	0.00931	0.01145	0.01053	0.04001	0.03776	0.04655	0.04520	0.04409	0.04679
Q9EQP2	EHD4_MOUSE	0.02957	0.02715	0.02479	0.02437	0.02519	0.02852	0.21938	0.27428	0.16490	0.17333	0.14883	0.21817
Q9EQS3	MYCBP_MOUSE	0.00000	0.00000	0.00000	0.00000	0.00000	0.00000	0.00672	0.00469	0.00937	0.00767	0.01056	0.00575
Q9EQU5	SET_MOUSE	0.00000	0.00000	0.00000	0.00000	0.00000	0.00000	0.06514	0.05640	0.06073	0.06577	0.04079	0.07581
Q9EQW7	KI13A_MOUSE	0.00000	0.00000	0.00000	0.00000	0.00000	0.00000	0.00000	0.00000	0.00144	0.00052	0.00000	0.00000
Q9EQX4	AIF1L_MOUSE	0.00000	0.00000	0.00000	0.00000	0.00000	0.00000	0.01145	0.00000	0.01193	0.00990	0.01278	0.00614
Q9ER00	STX12_MOUSE	0.04428	0.00210	0.00272	0.00187	0.00227	0.00266	0.01141	0.00760	0.01480	0.01235	0.01649	0.01391
Q9ER38	TOR3A_MOUSE	0.00000	0.00000	0.00000	0.00000	0.00000	0.00000	0.00333	0.00198	0.00389	0.00348	0.00547	0.00394
Q9ER39	TOR1A_MOUSE	0.00000	0.00000	0.00000	0.00000	0.00000	0.00000	0.00095	0.00487	0.00282	0.00153	0.00000	0.00070
Q9ER41	TOR1B_MOUSE	0.00000	0.00000	0.00000	0.00000	0.00000	0.00000	0.00000	0.00478	0.00173	0.00240	0.00096	0.00155
Q9ER72	SYCC_MOUSE	0.00000	0.00000	0.00000	0.00000	0.00000	0.00000	0.00000	0.00041	0.00000	0.00000	0.00171	0.00091
Q9ER88	RT29_MOUSE	0.00000	0.00000	0.00000	0.00000	0.00000	0.00000	0.00034	0.00000	0.00000	0.00030	0.00000	0.00000
Q9ERB0	SNP29_MOUSE	0.00000	0.00000	0.00000	0.00000	0.00000	0.00000	0.00233	0.00188	0.00277	0.00326	0.00386	0.00157
Q9ERD7	TBB3_MOUSE	0.00156	0.00211	0.00554	0.00241	0.00124	0.00244	0.00000	0.00000	0.00098	0.00000	0.00000	0.00000

Q9ERE7	MESD_MOUSE	0.00000	0.00000	0.00000	0.00000	0.00000	0.00000	0.00285	0.00354	0.00672	0.00502	0.00584	0.00482
Q9ERF3	WDR61_MOUSE	0.00000	0.00000	0.00000	0.00000	0.00000	0.00000	0.01840	0.00653	0.02192	0.01003	0.02369	0.03052
Q9ERG0	LIMA1_MOUSE	0.00000	0.00000	0.00000	0.00000	0.00000	0.00000	0.02066	0.03323	0.02428	0.02626	0.01984	0.02515
Q9ERG2	STRN3_MOUSE	0.00080	0.00107	0.00128	0.00087	0.00054	0.00113	0.00403	0.00389	0.01014	0.00594	0.00885	0.00702
Q9ERI2	RB27A_MOUSE	0.00000	0.00000	0.00000	0.00000	0.00000	0.00000	0.00273	0.00161	0.00437	0.00449	0.00256	0.00254
Q9ERI6	RDH14_MOUSE	0.00187	0.00056	0.00135	0.00073	0.00064	0.00047	0.00086	0.00152	0.00055	0.00149	0.00073	0.00151
Q9ERK4	XPO2_MOUSE	0.00468	0.08522	0.00284	0.03001	0.00289	0.03557	0.00945	0.01228	0.01309	0.01428	0.01102	0.01565
Q9ERL7	GMFG_MOUSE	0.00000	0.00000	0.00000	0.00000	0.00000	0.00000	0.00000	0.00667	0.00165	0.00110	0.00559	0.00603
Q9ERL9	GCYA1_MOUSE	0.00000	0.00000	0.00000	0.00000	0.00000	0.00000	0.00659	0.01195	0.01159	0.01173	0.01097	0.01586
Q9ERN0	SCAM2_MOUSE	0.00000	0.00000	0.00000	0.00000	0.00000	0.00000	0.01610	0.01214	0.01658	0.01635	0.01771	0.01751
Q9ERR7	SEP15_MOUSE	0.00000	0.00000	0.00000	0.00000	0.00000	0.00000	0.01125	0.00597	0.01228	0.01225	0.01222	0.01269
Q9ERS2	NDUAD_MOUSE	0.20454	0.17854	0.20547	0.18202	0.21932	0.21236	0.01162	0.01753	0.00824	0.01002	0.00433	0.01601
Q9ERS5	PKHA2_MOUSE	0.00000	0.00000	0.00000	0.00000	0.00000	0.00000	0.00281	0.00172	0.00177	0.00122	0.00458	0.00508
Q9ERT9	PPR1A_MOUSE	0.00000	0.00000	0.00000	0.00000	0.00000	0.00000	0.00000	0.00000	0.00000	0.00000	0.00000	0.00072
Q9ERU3	ZNF22_MOUSE	0.00000	0.00000	0.00000	0.00000	0.00000	0.00000	0.00606	0.00224	0.00319	0.00293	0.00127	0.00303
Q9ERU9	RBP2_MOUSE	0.00000	0.00000	0.00000	0.00000	0.00000	0.00000	0.00656	0.00542	0.01269	0.00802	0.01032	0.00780
Q9ES28	ARHG7_MOUSE	0.00000	0.00000	0.00000	0.00000	0.00000	0.00000	0.00381	0.00296	0.00765	0.00535	0.01015	0.00627
Q9ES46	PARVB_MOUSE	0.01380	0.01902	0.01898	0.02285	0.01338	0.02598	0.02060	0.01097	0.01575	0.01349	0.01522	0.01438
Q9ES52	SHIP1_MOUSE	0.00000	0.00000	0.00000	0.00000	0.00000	0.00000	0.00000	0.00029	0.00257	0.00074	0.00137	0.00378
Q9ES56	TPPC4_MOUSE	0.00000	0.00000	0.00000	0.00000	0.00000	0.00000	0.01196	0.00947	0.00962	0.00999	0.00903	0.01140
Q9ES64	USH1C_MOUSE	0.00000	0.00000	0.00000	0.00000	0.00000	0.00000	0.00000	0.00000	0.00000	0.00000	0.00000	0.00000
Q9ES74	NEK7_MOUSE	0.00000	0.00000	0.00000	0.00000	0.00000	0.00000	0.00345	0.00000	0.00283	0.00000	0.00276	0.00299
Q9ES97	RTN3_MOUSE	0.00877	0.01010	0.00056	0.00051	0.00534	0.00215	0.00984	0.02670	0.03188	0.02254	0.02017	0.01715
Q9ESB3	HRG_MOUSE	0.00000	0.00000	0.00000	0.00000	0.00000	0.00000	0.00730	0.00441	0.00731	0.00908	0.00655	0.01103
Q9ESE1	LRBA_MOUSE	0.00000	0.00000	0.00000	0.00000	0.00000	0.00000	0.02445	0.06807	0.05892	0.07948	0.02084	0.06820
Q9ESG4	CLTRN_MOUSE	0.00000	0.00000	0.00000	0.00000	0.00000	0.00000	0.00000	0.00000	0.00686	0.00325	0.00311	0.00000
Q9ESL4	M3K20_MOUSE	0.00357	0.00154	0.00358	0.00172	0.00248	0.00150	0.00260	0.00842	0.00665	0.00440	0.00760	0.00500
Q9EST1	GSDMA_MOUSE	0.00000	0.00000	0.00000	0.00000	0.00000	0.00000	0.00000	0.00000	0.00000	0.00000	0.00000	0.00000
Q9EST5	AN32B_MOUSE	0.00000	0.00000	0.00000	0.00000	0.00000	0.00000	0.02868	0.01736	0.03059	0.02295	0.03819	0.03191
Q9ESW4	AGK_MOUSE	0.01152	0.00789	0.00928	0.01072	0.00676	0.00732	0.00424	0.00204	0.00427	0.00156	0.00510	0.00347
Q9ESW8	PGPI_MOUSE	0.00000	0.00000	0.00000	0.00000	0.00000	0.00000	0.00000	0.00000	0.00000	0.00000	0.00000	0.00000
Q9ESX5	DKC1_MOUSE	0.00000	0.00000	0.00000	0.00000	0.00000	0.00000	0.00464	0.00192	0.00570	0.00373	0.00641	0.00582
Q9ESZ8	GTF2I_MOUSE	0.00000	0.00000	0.00000	0.00000	0.00000	0.00000	0.00061	0.00455	0.00125	0.00665	0.00201	0.00301
Q9ET01	PYGL_MOUSE	0.00006	0.00018	0.00009	0.00037	0.00013	0.00008	0.01105	0.01006	0.01259	0.00617	0.00891	0.00747

Q9ET22	DPP2_MOUSE	0.00000	0.00000	0.00000	0.00000	0.00000	0.00000	0.00592	0.00721	0.00621	0.00703	0.00364	0.01137
Q9ET30	TM9S3_MOUSE	0.00000	0.00000	0.00000	0.00000	0.00000	0.00000	0.00414	0.00401	0.00918	0.00516	0.00687	0.00537
Q9ET54	PALLD_MOUSE	0.00000	0.00000	0.00000	0.00000	0.00000	0.00000	0.01431	0.01360	0.01120	0.01230	0.00984	0.01303
Q9ET78	JPH2_MOUSE	0.01854	0.03445	0.01476	0.02673	0.02047	0.02073	0.00226	0.00111	0.00196	0.00107	0.00083	0.00087
Q9JHH9	COPZ2_MOUSE	0.00000	0.00000	0.00000	0.00000	0.00000	0.00000	0.00408	0.00310	0.00649	0.00373	0.00715	0.00591
Q9JHI5	IVD_MOUSE	0.05062	0.03170	0.04118	0.02984	0.04705	0.05572	0.04552	0.13288	0.10229	0.08399	0.10030	0.07979
Q9JHJ0	TMOD3_MOUSE	0.00000	0.00000	0.00000	0.00000	0.00000	0.00000	0.02601	0.03174	0.04183	0.03819	0.03473	0.04699
Q9JHK5	PLEK_MOUSE	0.00000	0.00000	0.00000	0.00000	0.00000	0.00000	0.00456	0.00398	0.00353	0.00673	0.00663	0.00419
Q9JHL1	NHRF2_MOUSE	0.00000	0.00000	0.00000	0.00000	0.00000	0.00000	0.03357	0.03698	0.04916	0.04002	0.04499	0.03761
Q9JHQ0	ANXA9_MOUSE	0.00000	0.00000	0.00000	0.00000	0.00000	0.00000	0.00765	0.00754	0.01163	0.00510	0.00838	0.00928
Q9JHQ5	LZTL1_MOUSE	0.00000	0.00000	0.00000	0.00000	0.00000	0.00000	0.00036	0.00000	0.00222	0.00074	0.00241	0.00073
Q9JHR7	IDE_MOUSE	0.00210	0.00520	0.00311	0.00208	0.00247	0.00182	0.01260	0.00812	0.00734	0.00746	0.00851	0.01079
Q9JHS4	CLPX_MOUSE	0.00000	0.00000	0.00000	0.00000	0.00000	0.00000	0.00000	0.00000	0.00000	0.00000	0.00000	0.00000
Q9JHU2	PALMD_MOUSE	0.00266	0.00145	0.00259	0.00204	0.00168	0.00212	0.00000	0.00000	0.00000	0.00000	0.00000	0.00000
Q9JHU4	DYHC1_MOUSE	0.05583	0.06860	0.04703	0.05512	0.04812	0.05701	0.06868	0.06555	0.06518	0.06419	0.06812	0.06862
Q9JHU9	INO1_MOUSE	0.00000	0.00000	0.00000	0.00000	0.00000	0.00000	0.00374	0.00403	0.00467	0.00292	0.00426	0.00447
Q9JHW2	NIT2_MOUSE	0.02153	0.03445	0.01571	0.01908	0.01333	0.01752	0.03799	0.03991	0.03640	0.03405	0.01741	0.04647
Q9JI10	STK3_MOUSE	0.00000	0.00000	0.00000	0.00000	0.00000	0.00000	0.00668	0.01136	0.01309	0.00751	0.00699	0.01437
Q9JI33	NET4_MOUSE	0.00000	0.00000	0.00000	0.00000	0.00000	0.00000	0.00000	0.00163	0.00289	0.00221	0.00099	0.00109
Q9JI46	NUDT3_MOUSE	0.00000	0.00000	0.00000	0.00000	0.00000	0.00000	0.00000	0.00577	0.00165	0.00192	0.00239	0.00316
Q9JI48	PLAC8_MOUSE	0.00000	0.00000	0.00000	0.00000	0.00000	0.00000	0.01039	0.00896	0.01307	0.01263	0.00554	0.02352
Q9JI75	NQO2_MOUSE	0.01150	0.00724	0.00821	0.00427	0.00580	0.00720	0.02599	0.01609	0.01505	0.01587	0.02180	0.01611
Q9JI91	ACTN2_MOUSE	2.17497	2.19004	1.63950	1.58789	2.00750	1.87618	0.11060	0.03776	0.03483	0.04074	0.03331	0.02498
Q9JIF0	ANM1_MOUSE	0.00856	0.00734	0.00478	0.00245	0.00259	0.00390	0.01272	0.01951	0.02188	0.01798	0.01609	0.01936
Q9JIF7	COPB_MOUSE	0.00524	0.00553	0.00431	0.00174	0.00448	0.00447	0.01352	0.01301	0.01814	0.01484	0.01675	0.01627
Q9JIF9	MYOTI_MOUSE	0.00000	0.00000	0.00000	0.00000	0.00000	0.00000	0.00245	0.00099	0.00145	0.00150	0.00211	0.00148
Q9JIG7	CCD22_MOUSE	0.00000	0.00000	0.00000	0.00000	0.00000	0.00000	0.00000	0.00702	0.00859	0.00707	0.00728	0.00617
Q9JII5	DAZP1_MOUSE	0.00000	0.00000	0.00000	0.00000	0.00000	0.00000	0.01492	0.03102	0.02698	0.01778	0.02569	0.02670
Q9JII6	AK1A1_MOUSE	0.00000	0.00000	0.00000	0.00000	0.00000	0.00000	0.04098	0.03487	0.06557	0.05221	0.07015	0.04858
Q9JIK5	DDX21_MOUSE	0.00000	0.00000	0.00000	0.00000	0.00000	0.00000	0.00743	0.00638	0.00593	0.00786	0.00582	0.00735
Q9JIL4	NHRF3_MOUSE	0.00000	0.00000	0.00000	0.00000	0.00000	0.00000	0.00015	0.00173	0.00017	0.00156	0.00086	0.00008
Q9JIQ3	DBLOH_MOUSE	0.00132	0.00207	0.00179	0.00096	0.00202	0.00181	0.00098	0.01010	0.01119	0.00883	0.00943	0.00861
Q9JIS8	S12A4_MOUSE	0.00000	0.00000	0.00000	0.00000	0.00000	0.00000	0.00103	0.00146	0.00450	0.00377	0.00203	0.00167
Q9JIW9	RALB_MOUSE	0.00000	0.00000	0.00000	0.00000	0.00000	0.00000	0.02761	0.04093	0.04743	0.04493	0.04777	0.05166

Q9JIX8	ACINU_MOUSE	0.00000	0.00000	0.00000	0.00000	0.00000	0.00000	0.00601	0.00974	0.01268	0.00861	0.01348	0.01275
Q9JIY7	NAT8_MOUSE	0.00000	0.00000	0.00000	0.00000	0.00000	0.00000	0.00000	0.00000	0.00000	0.00000	0.00000	0.00000
Q9JIZ9	PLS3_MOUSE	0.00000	0.00000	0.00000	0.00000	0.00000	0.00000	0.00000	0.00257	0.00056	0.00138	0.00109	0.00098
Q9JJ28	FLII_MOUSE	0.02111	0.00047	0.00112	0.02578	0.00183	0.01732	0.01419	0.01192	0.01820	0.01354	0.01452	0.01754
Q9JJ59	ABCB9_MOUSE	0.00000	0.00000	0.00000	0.00000	0.00000	0.00000	0.02993	0.00000	0.00000	0.00000	0.00000	0.00000
Q9JJC6	RIPL1_MOUSE	0.00000	0.00000	0.00000	0.00000	0.00000	0.00000	0.00372	0.00391	0.00386	0.00186	0.00358	0.00390
Q9JJH1	RNAS4_MOUSE	0.00000	0.00000	0.00000	0.00000	0.00000	0.00000	0.00446	0.00877	0.00432	0.00484	0.00644	0.00645
Q9JJI8	RL38_MOUSE	0.00000	0.00000	0.00000	0.00000	0.00000	0.00000	0.01497	0.02283	0.01665	0.02459	0.02627	0.02376
Q9JJK2	LANC2_MOUSE	0.00000	0.00000	0.00000	0.00000	0.00000	0.00000	0.00000	0.00338	0.00232	0.00652	0.01522	0.00879
Q9JJR8	TMM9B_MOUSE	0.00000	0.00000	0.00000	0.00000	0.00000	0.00000	0.00000	0.00000	0.00000	0.00000	0.00000	0.00000
Q9JJU8	SH3L1_MOUSE	0.00000	0.00000	0.00000	0.00000	0.00000	0.00000	0.01074	0.02021	0.02794	0.03017	0.03516	0.03309
Q9JJW0	PXMP4_MOUSE	0.00000	0.00000	0.00000	0.00000	0.00000	0.00000	0.00000	0.00000	0.00026	0.00000	0.00000	0.00000
Q9JJW5	MYOZ2_MOUSE	0.17835	0.19651	0.19560	0.18741	0.24198	0.23663	0.00471	0.00628	0.00277	0.00446	0.00606	0.00332
Q9JJX6	P2RX4_MOUSE	0.00000	0.00000	0.00000	0.00000	0.00000	0.00000	0.00087	0.00522	0.00115	0.00273	0.00229	0.00248
Q9JJZ2	TBA8_MOUSE	0.02707	0.02476	0.01879	0.02180	0.02019	0.01929	0.00377	0.00144	0.00206	0.00195	0.00000	0.00000
Q9JK37	MYOZ1_MOUSE	0.00000	0.00000	0.00000	0.00000	0.00000	0.00000	0.06800	0.00000	0.00008	0.00006	0.00006	0.00002
Q9JK42	PDK2_MOUSE	0.05535	0.06621	0.07562	0.05051	0.05520	0.05170	0.00846	0.00264	0.00000	0.00346	0.00000	0.00203
Q9JK48	SHLB1_MOUSE	0.00112	0.00078	0.00239	0.00096	0.00152	0.00274	0.00487	0.00515	0.00480	0.00580	0.00468	0.00523
Q9JK53	PRELP_MOUSE	0.00273	0.00438	0.00290	0.00445	0.00293	0.00448	0.05969	0.06673	0.07588	0.05432	0.06581	0.05879
Q9JK81	MYG1_MOUSE	0.00000	0.00000	0.00000	0.00000	0.00000	0.00000	0.00634	0.00521	0.00874	0.00759	0.00992	0.00921
Q9JK83	PAR6B_MOUSE	0.00000	0.00000	0.00000	0.00000	0.00000	0.00000	0.00626	0.00775	0.00999	0.00783	0.01053	0.00680
Q9JK91	MLH1_MOUSE	0.00000	0.00000	0.00000	0.00000	0.00000	0.00000	0.12359	0.01039	0.09719	0.00419	0.11897	0.00000
Q9JKB1	UCHL3_MOUSE	0.00616	0.01852	0.06602	0.01136	0.01226	0.02133	0.00779	0.00983	0.01008	0.01286	0.01055	0.01129
Q9JKB3	YBOX3_MOUSE	0.00039	0.00072	0.00038	0.00035	0.00041	0.00023	0.00138	0.00014	0.00184	0.00000	0.00089	0.00075
Q9JKC8	AP3M1_MOUSE	0.00000	0.00000	0.00000	0.00000	0.00000	0.00000	0.00624	0.00000	0.00000	0.00000	0.00054	0.00000
Q9JKF1	IQGA1_MOUSE	0.00541	0.01096	0.00374	0.00491	0.00457	0.00574	0.12083	0.11794	0.11988	0.11812	0.12362	0.13110
Q9JKF7	RM39_MOUSE	0.00000	0.00000	0.00000	0.00000	0.00000	0.00000	0.00000	0.00000	0.00000	0.00000	0.00000	0.00000
Q9JKK7	TMOD2_MOUSE	0.00000	0.00000	0.00000	0.00000	0.00000	0.00000	0.00000	0.00465	0.00209	0.00510	0.00000	0.00217
Q9JKL4	NDUF3_MOUSE	0.00000	0.00000	0.00000	0.00000	0.00000	0.00000	0.00000	0.00000	0.00552	0.00183	0.00057	0.00000
Q9JKR6	HYOU1_MOUSE	0.00119	0.00079	0.00144	0.00093	0.00136	0.00198	0.06045	0.06019	0.06675	0.05795	0.06740	0.06376
Q9JKS4	LDB3_MOUSE	0.27434	0.22493	0.19926	0.24394	0.28001	0.31224	0.01245	0.00601	0.00542	0.00625	0.00279	0.00358
Q9JKS5	HABP4_MOUSE	0.00000	0.00000	0.00000	0.00000	0.00000	0.00000	0.00000	0.00000	0.00180	0.00000	0.00073	0.00057
Q9JKV1	ADRM1_MOUSE	0.00000	0.00000	0.00000	0.00000	0.00000	0.00000	0.00273	0.00189	0.00554	0.00409	0.00219	0.00637
Q9JKX6	NUDT5_MOUSE	0.00000	0.00000	0.00000	0.00000	0.00000	0.00000	0.00184	0.00353	0.01194	0.00533	0.00725	0.00592

Q9JKY5	HIP1R_MOUSE	0.00000	0.00000	0.00000	0.00000	0.00000	0.00000	0.00317	0.00382	0.00532	0.00317	0.00199	0.00443
Q9JL16	ISG20_MOUSE	0.00000	0.00000	0.00000	0.00000	0.00000	0.00000	0.00270	0.00105	0.00164	0.00236	0.00134	0.00205
Q9JL26	FMNL1_MOUSE	0.00000	0.00000	0.00000	0.00000	0.00000	0.00000	0.00239	0.00238	0.00343	0.00340	0.00428	0.00483
Q9JL35	HMGN5_MOUSE	0.00000	0.00000	0.00000	0.00000	0.00000	0.00000	0.00201	0.00869	0.01567	0.01109	0.01338	0.00964
Q9JL62	GLTP_MOUSE	0.00000	0.00000	0.00000	0.00000	0.00000	0.00000	0.02617	0.02586	0.04963	0.02549	0.03043	0.04305
Q9JLB0	MPP6_MOUSE	0.00000	0.00000	0.00000	0.00000	0.00000	0.00000	0.00000	0.00000	0.00065	0.00065	0.00000	0.00164
Q9JLB2	MPP5_MOUSE	0.00000	0.00000	0.00000	0.00000	0.00000	0.00000	0.00566	0.01079	0.01278	0.00820	0.01426	0.01117
Q9JLB4	CUBN_MOUSE	0.00000	0.00000	0.00000	0.00000	0.00000	0.00000	0.00874	0.00971	0.00825	0.01022	0.00691	0.01043
Q9JLF6	TGM1_MOUSE	0.00000	0.00000	0.00000	0.00000	0.00000	0.00000	0.00396	0.00000	0.00136	0.00000	0.00432	0.00115
Q9JLI6	SCLY_MOUSE	0.00000	0.00000	0.00000	0.00000	0.00000	0.00000	0.00112	0.00145	0.00000	0.00069	0.00000	0.00149
Q9JLI8	SART3_MOUSE	0.00000	0.00000	0.00000	0.00000	0.00000	0.00000	0.00000	0.00000	0.00141	0.00618	0.00000	0.00000
Q9JLJ2	AL9A1_MOUSE	0.00590	0.00648	0.00606	0.00607	0.00846	0.00679	0.03598	0.08741	0.04540	0.07386	0.03768	0.05991
Q9JLJ5	ELOV1_MOUSE	0.00000	0.00000	0.00000	0.00000	0.00000	0.00000	0.01989	0.04500	0.01697	0.02934	0.02101	0.01964
Q9JLN9	MTOR_MOUSE	0.00000	0.00000	0.00000	0.00000	0.00000	0.00000	0.09830	0.04871	0.06829	0.04785	0.06395	0.06840
Q9JLQ0	CD2AP_MOUSE	0.00000	0.00000	0.00000	0.00000	0.00000	0.00000	0.00311	0.00405	0.01499	0.00769	0.02044	0.01125
Q9JLQ2	GIT2_MOUSE	0.00000	0.00000	0.00000	0.00000	0.00000	0.00000	0.00401	0.00283	0.00692	0.00313	0.00177	0.00575
Q9JLR9	HIG1A_MOUSE	0.00000	0.00000	0.00000	0.00000	0.00000	0.00000	0.00616	0.00857	0.00145	0.00358	0.00185	0.00172
Q9JLT2	TREA_MOUSE	0.00000	0.00000	0.00000	0.00000	0.00000	0.00000	0.00000	0.00005	0.00000	0.00000	0.00000	0.00013
Q9JLT4	TRXR2_MOUSE	0.00794	0.00822	0.01453	0.00909	0.01339	0.00991	0.02118	0.03239	0.02375	0.01892	0.00730	0.00000
Q9JLV1	BAG3_MOUSE	0.01013	0.00564	0.00335	0.00795	0.00522	0.00930	0.01256	0.00979	0.00843	0.00849	0.00683	0.00990
Q9JLV5	CUL3_MOUSE	0.00000	0.00000	0.00000	0.00000	0.00000	0.00000	0.00132	0.01376	0.01184	0.00986	0.00654	0.01456
Q9JLV6	PNKP_MOUSE	0.00000	0.00000	0.00000	0.00000	0.00000	0.00000	0.00501	0.00365	0.00633	0.00491	0.00372	0.00710
Q9JLZ3	AUHM_MOUSE	0.00000	0.00000	0.00000	0.00000	0.00000	0.00000	0.00195	0.00390	0.00387	0.00266	0.00346	0.00123
Q9JLZ8	SIGIR_MOUSE	0.00000	0.00000	0.00000	0.00000	0.00000	0.00000	0.00000	0.00000	0.00000	0.00000	0.00000	0.00000
Q9JM13	RABX5_MOUSE	0.00000	0.00000	0.00000	0.00000	0.00000	0.00000	0.00167	0.00081	0.00155	0.00159	0.00085	0.00228
Q9JM14	NT5C_MOUSE	0.00000	0.00000	0.00000	0.00000	0.00000	0.00000	0.01015	0.01009	0.01118	0.01200	0.01082	0.01088
Q9JM62	REEP6_MOUSE	0.00000	0.00000	0.00000	0.00000	0.00000	0.00000	0.00465	0.00879	0.00000	0.00000	0.00241	0.00700
Q9JM63	KCJ10_MOUSE	0.00000	0.00000	0.00000	0.00000	0.00000	0.00000	0.00000	0.00013	0.00000	0.00000	0.00000	0.00000
Q9JM76	ARPC3_MOUSE	0.00000	0.00000	0.00000	0.00000	0.00000	0.00000	0.00062	0.01057	0.01791	0.01397	0.00697	0.01160
Q9JM83	CALM4_MOUSE	0.00000	0.00000	0.00000	0.00000	0.00000	0.00000	0.01157	0.00000	0.00102	0.00000	0.00000	0.00134
Q9JM96	BORG4_MOUSE	0.00000	0.00000	0.00000	0.00000	0.00000	0.00000	0.00663	0.01064	0.01170	0.01185	0.01160	0.01124
Q9JMA1	UBP14_MOUSE	0.00742	0.00650	0.00787	0.02240	0.00585	0.00801	0.00539	0.01129	0.01370	0.01102	0.00725	0.01052
Q9JMA9	S6A14_MOUSE	0.00000	0.00000	0.00000	0.00000	0.00000	0.00000	0.00676	0.00846	0.01000	0.00891	0.00765	0.00921
Q9JMC3	DNJA4_MOUSE	0.00000	0.00000	0.00000	0.00000	0.00000	0.00000	0.00468	0.00764	0.00301	0.00491	0.00809	0.00742

Q9JMD0	ZN207_MOUSE	0.00000	0.00000	0.00000	0.00000	0.00000	0.00000	0.00000	0.00000	0.00000	0.00000	0.00000	0.00000
Q9JMD3	STA10_MOUSE	0.00000	0.00000	0.00000	0.00000	0.00000	0.00000	0.00123	0.00585	0.00529	0.00568	0.00350	0.00527
Q9JME5	AP3B2_MOUSE	0.00000	0.00000	0.00000	0.00000	0.00000	0.00000	0.00000	0.00066	0.00199	0.00108	0.00212	0.00152
Q9JME7	TPC2L_MOUSE	0.00000	0.00000	0.00000	0.00000	0.00000	0.00000	0.00000	0.00000	0.00000	0.00000	0.00106	0.00151
Q9JMG1	EDF1_MOUSE	0.00000	0.00000	0.00000	0.00000	0.00000	0.00000	0.00000	0.00915	0.00199	0.00126	0.00000	0.00000
Q9JMG4	NKAI4_MOUSE	0.00000	0.00000	0.00000	0.00000	0.00000	0.00000	0.00000	0.00253	0.00557	0.00202	0.00262	0.00344
Q9JMG7	HDGR3_MOUSE	0.00000	0.00000	0.00000	0.00000	0.00000	0.00000	0.00550	0.00647	0.01013	0.00688	0.00555	0.00895
Q9JMH6	TRXR1_MOUSE	0.00166	0.00196	0.00204	0.00109	0.00292	0.00149	0.00623	0.00798	0.01335	0.01214	0.01365	0.00965
Q9JMH9	MY18A_MOUSE	0.01273	0.01415	0.01091	0.00782	0.01070	0.00922	0.00742	0.00597	0.00868	0.00657	0.00876	0.00725
Q9NYQ2	HAOX2_MOUSE	0.00000	0.00000	0.00000	0.00000	0.00000	0.00000	0.00165	0.00000	0.00038	0.00033	0.00000	0.00000
Q9QUH0	GLRX1_MOUSE	0.00000	0.00000	0.00000	0.00000	0.00000	0.00000	0.00612	0.00278	0.00418	0.00276	0.00213	0.00204
Q9QUI0	RHOA_MOUSE	0.03241	0.02312	0.02346	0.01619	0.02144	0.01652	0.00000	0.02594	0.03039	0.01589	0.00676	0.00887
Q9QUJ7	ACSL4_MOUSE	0.00000	0.00000	0.00000	0.00000	0.00000	0.00000	0.02723	0.02189	0.02869	0.02506	0.02217	0.02451
Q9QUM0	ITA2B_MOUSE	0.00000	0.00000	0.00000	0.00000	0.00000	0.00000	0.00298	0.00000	0.00855	0.00902	0.00551	0.00274
Q9QUM9	PSA6_MOUSE	0.00000	0.00000	0.00000	0.00000	0.00000	0.00000	0.01514	0.01697	0.02038	0.01791	0.01733	0.01821
Q9QUP5	HPLN1_MOUSE	0.00000	0.00000	0.00000	0.00000	0.00000	0.00000	0.02007	0.00072	0.00000	0.00030	0.01068	0.00000
Q9QUR6	PPCE_MOUSE	0.01929	0.02238	0.03504	0.02089	0.01915	0.01700	0.05342	0.06507	0.05095	0.06013	0.05248	0.06903
Q9QUR7	PIN1_MOUSE	0.00000	0.00000	0.00000	0.00000	0.00000	0.00000	0.00000	0.00519	0.00444	0.01130	0.00424	0.00104
Q9QVP4	MLRA_MOUSE	0.18220	0.10302	0.07211	0.17229	0.22760	0.13460	0.09115	0.08253	0.08278	0.12557	0.08526	0.07186
Q9QVP9	FAK2_MOUSE	0.00000	0.00000	0.00000	0.00000	0.00000	0.00000	0.00000	0.00124	0.00529	0.00495	0.00716	0.00705
Q9QWR8	NAGAB_MOUSE	0.00000	0.00000	0.00000	0.00000	0.00000	0.00000	0.01637	0.01902	0.01743	0.01298	0.01387	0.01430
Q9QWV4	MLF1_MOUSE	0.00000	0.00000	0.00000	0.00000	0.00000	0.00000	0.00378	0.00524	0.00998	0.00563	0.00920	0.00766
Q9QX60	DGUOK_MOUSE	0.00693	0.00365	0.00372	0.00223	0.00522	0.00387	0.00928	0.01296	0.01169	0.00944	0.01418	0.01130
Q9QXA5	LSM4_MOUSE	0.00000	0.00000	0.00000	0.00000	0.00000	0.00000	0.09208	0.02011	0.08340	0.06539	0.02929	0.08584
Q9QXC1	FETUB_MOUSE	0.00000	0.00000	0.00000	0.00000	0.00000	0.00000	0.00000	0.00220	0.00451	0.00221	0.00092	0.00000
Q9QXD1	ACOX2_MOUSE	0.00000	0.00000	0.00000	0.00000	0.00000	0.00000	0.01370	0.00962	0.01459	0.01145	0.01309	0.01153
Q9QXD6	F16P1_MOUSE	0.00000	0.00000	0.00000	0.00000	0.00000	0.00000	0.00000	0.00000	0.00001	0.00000	0.00000	0.00002
Q9QXD8	LIMD1_MOUSE	0.00000	0.00000	0.00000	0.00000	0.00000	0.00000	0.00218	0.00150	0.00473	0.00466	0.00394	0.00000
Q9QXE0	HACL1_MOUSE	0.00000	0.00000	0.00000	0.00000	0.00000	0.00000	0.00000	0.00000	0.00000	0.00000	0.00000	0.00250
Q9QXE7	TBL1X_MOUSE	0.00000	0.00000	0.00000	0.00000	0.00000	0.00000	0.00000	0.00209	0.00396	0.00299	0.00568	0.00179
Q9QXG4	ACSA_MOUSE	0.00000	0.00000	0.00000	0.00000	0.00000	0.00000	0.01255	0.00696	0.00548	0.00280	0.00294	0.00638
Q9QXK3	COPG2_MOUSE	0.00000	0.00000	0.00000	0.00000	0.00000	0.00000	0.00561	0.01499	0.00989	0.00867	0.00000	0.01030
Q9QXN0	SHRM3_MOUSE	0.00000	0.00000	0.00000	0.00000	0.00000	0.00000	0.00000	0.00000	0.00258	0.00127	0.00134	0.00000
Q9QXN5	MIOX_MOUSE	0.00000	0.00000	0.00000	0.00000	0.00000	0.00000	0.00000	0.00000	0.00067	0.00000	0.00000	0.00000

Q9QXS1	PLEC_MOUSE	0.21986	0.27727	0.20913	0.22790	0.21017	0.21278	0.11315	0.10168	0.11346	0.10867	0.10957	0.11136
Q9QXS6	DREB_MOUSE	0.00000	0.00000	0.00000	0.00000	0.00000	0.00000	0.00000	0.00000	0.00000	0.00000	0.00000	0.00000
Q9QXT0	CNPY2_MOUSE	0.00000	0.00000	0.00000	0.00000	0.00000	0.00000	0.02212	0.02114	0.02127	0.02704	0.02063	0.02463
Q9QXX4	CMC2_MOUSE	0.05194	0.03843	0.05801	0.04874	0.05523	0.05698	0.00583	0.00924	0.00992	0.00814	0.00430	0.01038
Q9QXY6	EHD3_MOUSE	0.00000	0.00000	0.00000	0.00000	0.00000	0.00000	0.00287	0.00736	0.00725	0.00573	0.00540	0.00734
Q9QXZ0	MACF1_MOUSE	0.00474	0.00022	0.00315	0.00020	0.00015	0.00307	0.03314	0.02783	0.03623	0.03601	0.03872	0.03856
Q9QXZ6	SO1A1_MOUSE	0.00000	0.00000	0.00000	0.00000	0.00000	0.00000	0.00000	0.00000	0.00000	0.00795	0.00367	0.00404
Q9QY30	ABCBB_MOUSE	0.00000	0.00000	0.00000	0.00000	0.00000	0.00000	0.00772	0.00745	0.00864	0.00883	0.00915	0.01036
Q9QY76	VAPB_MOUSE	0.00000	0.00000	0.00000	0.00000	0.00000	0.00000	0.01477	0.01281	0.02605	0.02203	0.01718	0.01756
Q9QYA2	TOM40_MOUSE	0.01690	0.02222	0.01567	0.01030	0.01371	0.01229	0.00881	0.00804	0.00504	0.00379	0.00191	0.00363
Q9QYB1	CLIC4_MOUSE	0.03754	0.04531	0.02782	0.02875	0.03775	0.03039	0.11042	0.12757	0.13513	0.11767	0.12331	0.14686
Q9QYB5	ADDG_MOUSE	0.00000	0.00000	0.00000	0.00000	0.00000	0.00000	0.04221	0.02722	0.03621	0.03808	0.04415	0.04324
Q9QYB8	ADDB_MOUSE	0.00000	0.00000	0.00000	0.00000	0.00000	0.00000	0.00000	0.00000	0.00000	0.00000	0.00000	0.00000
Q9QYC0	ADDA_MOUSE	0.00732	0.00494	0.00483	0.00583	0.00461	0.00586	0.02313	0.02099	0.02325	0.02299	0.02204	0.02658
Q9QYG0	NDRG2_MOUSE	0.11514	0.14587	0.18642	0.17759	0.13830	0.17575	0.03232	0.01120	0.01834	0.01407	0.01809	0.00980
Q9QYI3	DNJC7_MOUSE	0.00000	0.00000	0.00000	0.00000	0.00000	0.00000	0.00604	0.00563	0.00398	0.00450	0.00441	0.00583
Q9QYI5	DNJB2_MOUSE	0.00000	0.00000	0.00000	0.00000	0.00000	0.00000	0.00407	0.00852	0.00358	0.00754	0.00264	0.00680
Q9QYJ0	DNJA2_MOUSE	0.01960	0.01793	0.02209	0.01610	0.02112	0.02309	0.02212	0.01625	0.01399	0.01835	0.01115	0.02187
Q9QYJ3	DNJB1_MOUSE	0.00000	0.00000	0.00000	0.00000	0.00000	0.00000	0.00837	0.00501	0.00702	0.00581	0.00815	0.00819
Q9QYR9	ACOT2_MOUSE	0.00000	0.00000	0.00000	0.00000	0.00000	0.00000	0.03910	0.04575	0.04993	0.03740	0.03977	0.04780
Q9QYS9	QKI_MOUSE	0.00000	0.00000	0.00000	0.00000	0.00000	0.00000	0.00577	0.00773	0.00425	0.00463	0.00246	0.00425
Q9QYY0	GAB1_MOUSE	0.00000	0.00000	0.00000	0.00000	0.00000	0.00000	0.00091	0.00251	0.00059	0.00000	0.00000	0.00000
Q9QZ06	TOLIP_MOUSE	0.00302	0.00445	0.00330	0.00286	0.00284	0.00381	0.02360	0.02268	0.02380	0.01917	0.02227	0.02140
Q9QZ08	NAGK_MOUSE	0.00000	0.00000	0.00000	0.00000	0.00000	0.00000	0.00588	0.00559	0.00268	0.00513	0.00200	0.00606
Q9QZ23	NFU1_MOUSE	0.00000	0.00000	0.00000	0.00000	0.00000	0.00000	0.00707	0.00282	0.00627	0.00649	0.00670	0.00665
Q9QZ47	TNNT3_MOUSE	0.00000	0.00000	0.00000	0.00000	0.00000	0.00000	0.08458	0.00005	0.00007	0.00012	0.00002	0.00000
Q9QZ73	DCNL1_MOUSE	0.00114	0.00103	0.00085	0.00066	0.00122	0.00108	0.00527	0.00365	0.00518	0.00523	0.00619	0.00586
Q9QZ85	IIGP1_MOUSE	0.00000	0.00000	0.00000	0.00000	0.00000	0.00000	0.00042	0.00084	0.00099	0.00149	0.00000	0.00000
Q9QZ88	VPS29_MOUSE	0.00000	0.00000	0.00000	0.00000	0.00000	0.00000	0.01272	0.02000	0.01401	0.01989	0.00829	0.01788
Q9QZB7	ARP10_MOUSE	0.00000	0.00000	0.00000	0.00000	0.00000	0.00000	0.01110	0.01280	0.01250	0.00956	0.01236	0.01384
Q9QZB9	DCTN5_MOUSE	0.00000	0.00000	0.00000	0.00000	0.00000	0.00000	0.00336	0.00422	0.00142	0.00298	0.00236	0.00395
Q9QZD8	DIC_MOUSE	0.00000	0.00000	0.00000	0.00000	0.00000	0.00000	0.00000	0.00624	0.00670	0.00180	0.00143	0.00310
Q9QZD9	EIF3I_MOUSE	0.00000	0.00000	0.00000	0.00000	0.00000	0.00000	0.02426	0.01315	0.01688	0.01676	0.01320	0.01415
Q9QZE5	COPG1_MOUSE	0.00027	0.00000	0.00026	0.00007	0.00124	0.00127	0.02528	0.02033	0.03201	0.02813	0.03113	0.02723

Q9QZE7	TSNAX_MOUSE	0.00000	0.00000	0.00000	0.00000	0.00000	0.00000	0.00059	0.00871	0.00685	0.00397	0.00938	0.00610
Q9QZM0	UBQL2_MOUSE	0.00000	0.00000	0.00000	0.00000	0.00000	0.00000	0.00076	0.00464	0.00485	0.00425	0.00374	0.00511
Q9QZN3	FBX8_MOUSE	0.00000	0.00000	0.00000	0.00000	0.00000	0.00000	0.04411	0.00000	0.04544	0.02471	0.05463	0.02351
Q9QZN4	FBX6_MOUSE	0.00000	0.00000	0.00000	0.00000	0.00000	0.00000	0.00044	0.00068	0.00533	0.00366	0.00136	0.00053
Q9QZQ1	AFAD_MOUSE	0.00000	0.00000	0.00000	0.00000	0.00000	0.00000	0.01462	0.02160	0.02445	0.01990	0.01903	0.02413
Q9QZQ8	H2AY_MOUSE	0.00049	0.00087	0.00088	0.00088	0.00651	0.00054	0.10362	0.09070	0.07093	0.07456	0.09036	0.09235
Q9QZR9	CO4A4_MOUSE	0.00000	0.00000	0.00000	0.00000	0.00000	0.00000	0.18822	0.09571	0.08083	0.10080	0.15276	0.11025
Q9QZS0	CO4A3_MOUSE	0.00000	0.00000	0.00000	0.00000	0.00000	0.00000	0.04776	0.04817	0.02610	0.01670	0.04205	0.04368
Q9QZS3	NUMB_MOUSE	0.00000	0.00000	0.00000	0.00000	0.00000	0.00000	0.00054	0.00555	0.00953	0.00696	0.00442	0.00513
Q9QZW0	AT11C_MOUSE	0.00000	0.00000	0.00000	0.00000	0.00000	0.00000	0.00000	0.00000	0.00183	0.00000	0.00198	0.00158
Q9R020	ZRAB2_MOUSE	0.00000	0.00000	0.00000	0.00000	0.00000	0.00000	0.00000	0.00054	0.00267	0.00106	0.00141	0.00108
Q9R022	DJC12_MOUSE	0.00000	0.00000	0.00000	0.00000	0.00000	0.00000	0.00000	0.00052	0.00000	0.00000	0.00000	0.00000
Q9R059	FHL3_MOUSE	0.00000	0.00000	0.00000	0.00000	0.00000	0.00000	0.00016	0.00000	0.00000	0.00000	0.00000	0.00000
Q9R062	GLYG_MOUSE	0.02511	0.01839	0.03260	0.02070	0.02601	0.02926	0.05511	0.06493	0.07306	0.07912	0.06499	0.07774
Q9R069	BCAM_MOUSE	0.00616	0.01025	0.00477	0.00357	0.00737	0.00724	0.09967	0.14835	0.13523	0.12473	0.10718	0.12495
Q9R078	AAKB1_MOUSE	0.00000	0.00000	0.00000	0.00000	0.00000	0.00000	0.00000	0.00000	0.00159	0.00025	0.00052	0.00000
Q9R099	TBL2_MOUSE	0.00000	0.00000	0.00000	0.00000	0.00000	0.00000	0.00000	0.00094	0.00251	0.00169	0.00102	0.00098
Q9R0A0	PEX14_MOUSE	0.00000	0.00000	0.00000	0.00000	0.00000	0.00000	0.00209	0.00276	0.00371	0.00364	0.00000	0.00275
Q9R0E2	PLOD1_MOUSE	0.00000	0.00000	0.00000	0.00000	0.00000	0.00000	0.00146	0.00277	0.00288	0.00137	0.00392	0.00170
Q9R0G6	COMP_MOUSE	0.00000	0.00000	0.00000	0.00000	0.00000	0.00000	0.02364	0.00319	0.00336	0.00400	0.00571	0.00167
Q9R0H0	ACOX1_MOUSE	0.01556	0.01618	0.01489	0.01221	0.02744	0.01686	0.00491	0.00355	0.00525	0.00482	0.00552	0.00351
Q9R0M4	PODXL_MOUSE	0.00000	0.00000	0.00000	0.00000	0.00000	0.00000	0.02887	0.02691	0.03349	0.02746	0.04214	0.03687
Q9R0M5	TPK1_MOUSE	0.00000	0.00000	0.00000	0.00000	0.00000	0.00000	0.00000	0.00000	0.00156	0.00015	0.00102	0.00000
Q9R0M6	RAB9A_MOUSE	0.00000	0.00000	0.00000	0.00000	0.00000	0.00000	0.00502	0.00566	0.00860	0.00693	0.00840	0.00484
Q9R0N0	GALK1_MOUSE	0.00000	0.00000	0.00000	0.00000	0.00000	0.00000	0.00644	0.01193	0.00775	0.00914	0.00494	0.01183
Q9R0P3	ESTD_MOUSE	0.02808	0.01744	0.03693	0.01584	0.02542	0.02723	0.01913	0.02434	0.02545	0.02396	0.03169	0.02055
Q9R0P4	SMAP_MOUSE	0.00000	0.00000	0.00000	0.00000	0.00000	0.00000	0.00582	0.00400	0.01468	0.01236	0.01206	0.01064
Q9R0P5	DEST_MOUSE	0.00000	0.00000	0.00000	0.00000	0.00000	0.00000	0.11442	0.16401	0.13695	0.13296	0.09222	0.14599
Q9R0P6	SC11A_MOUSE	0.00000	0.00000	0.00000	0.00000	0.00000	0.00000	0.01023	0.00994	0.00910	0.00769	0.00755	0.00860
Q9R0P9	UCHL1_MOUSE	0.00000	0.00000	0.00000	0.00000	0.00000	0.00000	0.00746	0.00134	0.00829	0.00493	0.00361	0.00314
Q9R0Q3	TMED2_MOUSE	0.00000	0.00000	0.00000	0.00000	0.00000	0.00000	0.00000	0.00943	0.01600	0.00820	0.00371	0.00458
Q9R0Q6	ARC1A_MOUSE	0.00128	0.00162	0.00487	0.00271	0.00473	0.00170	0.00532	0.00442	0.00456	0.00445	0.00656	0.00585
Q9R0Q7	TEBP_MOUSE	0.00422	0.00235	0.00465	0.00207	0.00470	0.00373	0.02412	0.01492	0.02661	0.02135	0.02798	0.02085
Q9R0U0	SRS10_MOUSE	0.00000	0.00000	0.00000	0.00000	0.00000	0.00000	0.00613	0.00659	0.00789	0.00622	0.00629	0.00739

Q9R0X4	ACOT9_MOUSE	0.00070	0.00282	0.00458	0.00665	0.00188	0.00251	0.01452	0.01104	0.01372	0.01186	0.01405	0.01025
Q9R0Y5	KAD1_MOUSE	0.10958	0.12080	0.19925	0.27790	0.10686	0.22485	0.05328	0.00921	0.02120	0.01512	0.01875	0.01308
Q9R111	GUAD_MOUSE	0.00060	0.00139	0.00059	0.00357	0.00162	0.00077	0.01783	0.01644	0.01759	0.01487	0.01642	0.00989
Q9R112	SQOR_MOUSE	0.01124	0.00678	0.01038	0.00715	0.00871	0.00890	0.01801	0.02400	0.01605	0.01707	0.01333	0.01972
Q9R190	MTA2_MOUSE	0.00000	0.00000	0.00000	0.00000	0.00000	0.00000	0.00596	0.00997	0.00356	0.00629	0.00121	0.00537
Q9R1C7	PR40A_MOUSE	0.00000	0.00000	0.00000	0.00000	0.00000	0.00000	0.02060	0.00811	0.00600	0.00708	0.00664	0.00772
Q9R1J0	NSDHL_MOUSE	0.00000	0.00000	0.00000	0.00000	0.00000	0.00000	0.00090	0.00160	0.00323	0.00279	0.00348	0.00296
Q9R1K9	CETN2_MOUSE	0.00000	0.00000	0.00000	0.00000	0.00000	0.00000	0.00812	0.00974	0.00578	0.00770	0.00810	0.00754
Q9R1P0	PSA4_MOUSE	0.00000	0.00000	0.00000	0.00000	0.00000	0.00000	0.06979	0.05781	0.03731	0.05085	0.04132	0.04931
Q9R1P1	PSB3_MOUSE	0.01451	0.00980	0.03093	0.01205	0.00965	0.00931	0.04354	0.03799	0.04679	0.04690	0.06543	0.04521
Q9R1P3	PSB2_MOUSE	0.00000	0.00000	0.00000	0.00000	0.00000	0.00000	0.03449	0.01216	0.01987	0.01449	0.02706	0.01570
Q9R1P4	PSA1_MOUSE	0.00704	0.00569	0.00833	0.00663	0.00506	0.00556	0.01263	0.01755	0.02116	0.02901	0.01879	0.02400
Q9R1Q7	PLP2_MOUSE	0.00000	0.00000	0.00000	0.00000	0.00000	0.00000	0.02455	0.03429	0.02565	0.02943	0.00812	0.06533
Q9R1Q8	TAGL3_MOUSE	0.00000	0.00000	0.00000	0.00000	0.00000	0.00000	0.00093	0.00126	0.00359	0.00220	0.00157	0.00279
Q9R1T2	SAE1_MOUSE	0.00000	0.00000	0.00000	0.00000	0.00000	0.00000	0.00361	0.00621	0.01004	0.00702	0.00892	0.00613
Q9R1T4	SEPT6_MOUSE	0.00000	0.00000	0.00000	0.00000	0.00000	0.00000	0.00092	0.00319	0.00786	0.00577	0.00699	0.00317
Q9R1W5	CALRL_MOUSE	0.00000	0.00000	0.00000	0.00000	0.00000	0.00000	0.00551	0.03600	0.02667	0.02770	0.01357	0.02354
Q9R1Z8	VINEX_MOUSE	0.00000	0.00000	0.00000	0.00000	0.00000	0.00000	0.03057	0.05324	0.06265	0.04134	0.04280	0.05717
Q9R233	TPSN_MOUSE	0.00000	0.00000	0.00000	0.00000	0.00000	0.00000	0.01166	0.00743	0.01659	0.01319	0.01092	0.01904
Q9R257	HEBP1_MOUSE	0.00000	0.00000	0.00000	0.00000	0.00000	0.00000	0.00000	0.00000	0.00067	0.00000	0.00000	0.00000
Q9R269	PEPL_MOUSE	0.00000	0.00000	0.00000	0.00000	0.00000	0.00000	0.02693	0.02079	0.03069	0.02303	0.01678	0.01888
Q9WTI7	MYO1C_MOUSE	0.01766	0.04224	0.01512	0.02093	0.02069	0.02770	0.10185	0.11847	0.10553	0.10154	0.10382	0.12687
Q9WTK5	NFKB2_MOUSE	0.00000	0.00000	0.00000	0.00000	0.00000	0.00000	0.00000	0.00000	0.00000	0.00000	0.00000	0.00000
Q9WTL2	RAB25_MOUSE	0.00000	0.00000	0.00000	0.00000	0.00000	0.00000	0.00000	0.00000	0.00112	0.00000	0.00000	0.00000
Q9WTL7	LYPA2_MOUSE	0.00000	0.00000	0.00000	0.00000	0.00000	0.00000	0.00266	0.00322	0.00385	0.00268	0.00577	0.00320
Q9WTM5	RUVB2_MOUSE	0.00000	0.00000	0.00000	0.00000	0.00000	0.00000	0.00723	0.00723	0.01000	0.01014	0.00827	0.00989
Q9WTP6	KAD2_MOUSE	0.01603	0.00900	0.01321	0.00723	0.01087	0.00869	0.00808	0.00839	0.00545	0.00650	0.00537	0.00613
Q9WTP7	KAD3_MOUSE	0.03911	0.02382	0.02191	0.02040	0.01727	0.03129	0.04294	0.03997	0.03360	0.03148	0.03090	0.03851
Q9WTQ5	AKA12_MOUSE	0.00000	0.00000	0.00000	0.00000	0.00000	0.00000	0.04140	0.04643	0.07592	0.08080	0.08012	0.09017
Q9WTQ8	TIM23_MOUSE	0.00000	0.00000	0.00000	0.00000	0.00000	0.00000	0.00856	0.00738	0.00367	0.00635	0.00515	0.00693
Q9WTR5	CAD13_MOUSE	0.03225	0.04477	0.01777	0.02595	0.02821	0.05880	0.01165	0.01147	0.02608	0.01404	0.01142	0.01576
Q9WTU6	MK09_MOUSE	0.00000	0.00000	0.00000	0.00000	0.00000	0.00000	0.01193	0.01554	0.02138	0.01839	0.00496	0.01927
Q9WTX5	SKP1_MOUSE	0.00618	0.00653	0.00946	0.01174	0.00603	0.00727	0.03417	0.02737	0.02686	0.03267	0.03059	0.03012
Q9WTX6	CUL1_MOUSE	0.00000	0.00000	0.00000	0.00000	0.00000	0.00000	0.00767	0.00423	0.00437	0.00415	0.00184	0.00383

Q9WTY4	AQP5_MOUSE	0.00000	0.00000	0.00000	0.00000	0.00000	0.00000	0.00877	0.04703	0.03965	0.05115	0.03010	0.04661
Q9WU40	MAN1_MOUSE	0.00000	0.00000	0.00000	0.00000	0.00000	0.00000	0.00291	0.00140	0.00456	0.00152	0.00575	0.00293
Q9WU78	PDC6I_MOUSE	0.05167	0.05625	0.03923	0.03067	0.04141	0.01954	0.03749	0.03129	0.03809	0.03335	0.03209	0.03163
Q9WU79	PROD_MOUSE	0.00157	0.00028	0.00041	0.00010	0.00028	0.00048	0.00064	0.00011	0.00000	0.00000	0.00018	0.00010
Q9WUA2	SYFB_MOUSE	0.00000	0.00000	0.00000	0.00000	0.00000	0.00000	0.01103	0.00888	0.00870	0.00761	0.01008	0.00886
Q9WUA3	PFKAP_MOUSE	0.00842	0.00818	0.00631	0.00407	0.00457	0.00845	0.02719	0.02545	0.03033	0.02761	0.03333	0.02740
Q9WUB3	PYGM_MOUSE	0.52417	0.47258	1.08050	0.73538	0.75926	0.46605	0.10584	0.01645	0.01939	0.02176	0.01881	0.01803
Q9WUB7	CLCKA_MOUSE	0.00000	0.00000	0.00000	0.00000	0.00000	0.00000	0.00225	0.00531	0.00186	0.00434	0.00000	0.00421
Q9WUD1	CHIP_MOUSE	0.00000	0.00000	0.00000	0.00000	0.00000	0.00000	0.00000	0.00250	0.00295	0.00204	0.00095	0.00102
Q9WUK2	IF4H_MOUSE	0.00000	0.00000	0.00000	0.00000	0.00000	0.00000	0.01773	0.03195	0.01460	0.01432	0.00665	0.01588
Q9WUL7	ARL3_MOUSE	0.00000	0.00000	0.00000	0.00000	0.00000	0.00000	0.00931	0.01006	0.00622	0.00745	0.00726	0.00783
Q9WUM3	COR1B_MOUSE	0.00000	0.00000	0.00000	0.00000	0.00000	0.00000	0.02065	0.04370	0.03967	0.03867	0.03492	0.03134
Q9WUM4	COR1C_MOUSE	0.00091	0.00181	0.00058	0.00151	0.00142	0.00257	0.02381	0.02165	0.02460	0.01959	0.02706	0.02463
Q9WUM5	SUCA_MOUSE	0.12148	0.29033	0.21907	0.31285	0.21956	0.16340	0.01674	0.01790	0.01964	0.01887	0.02029	0.01802
Q9WUN2	TBK1_MOUSE	0.00000	0.00000	0.00000	0.00000	0.00000	0.00000	0.00280	0.00242	0.00210	0.00233	0.00136	0.00000
Q9WUP7	UCHL5_MOUSE	0.00000	0.00000	0.00000	0.00000	0.00000	0.00000	0.00419	0.00737	0.00000	0.00377	0.00000	0.00489
Q9WUQ2	PREB_MOUSE	0.00000	0.00000	0.00000	0.00000	0.00000	0.00000	0.00339	0.00203	0.00654	0.00522	0.00383	0.00676
Q9WUR2	ECI2_MOUSE	0.05797	0.06300	0.04974	0.07936	0.08343	0.07388	0.01103	0.00760	0.01411	0.00895	0.01162	0.01137
Q9WUR9	KAD4_MOUSE	0.00000	0.00000	0.00000	0.00000	0.00000	0.00000	0.00000	0.00000	0.00000	0.00000	0.00000	0.00000
Q9WUZ9	ENTP5_MOUSE	0.00000	0.00000	0.00000	0.00000	0.00000	0.00000	0.00000	0.00000	0.00007	0.00000	0.00007	0.00000
Q9WV32	ARC1B_MOUSE	0.00000	0.00000	0.00000	0.00000	0.00000	0.00000	0.00604	0.01555	0.02197	0.01733	0.01643	0.00973
Q9WV34	MPP2_MOUSE	0.00000	0.00000	0.00000	0.00000	0.00000	0.00000	0.00036	0.00050	0.00172	0.00151	0.00192	0.00286
Q9WV35	ABEC2_MOUSE	0.02642	0.02226	0.03193	0.01675	0.03200	0.04449	0.04083	0.00571	0.00385	0.00330	0.00301	0.00000
Q9WV54	ASAH1_MOUSE	0.00000	0.00000	0.00000	0.00000	0.00000	0.00000	0.03721	0.05456	0.04209	0.03930	0.05004	0.03933
Q9WV55	VAPA_MOUSE	0.00341	0.00378	0.00296	0.00236	0.00273	0.00328	0.03667	0.03657	0.03553	0.03816	0.04485	0.04087
Q9WV60	GSK3B_MOUSE	0.00006	0.00034	0.00011	0.00022	0.00010	0.00000	0.00609	0.00398	0.00659	0.00609	0.00666	0.00524
Q9WV69	DEMA_MOUSE	0.00000	0.00000	0.00000	0.00000	0.00000	0.00000	0.00036	0.00046	0.00047	0.00067	0.00064	0.00000
Q9WV80	SNX1_MOUSE	0.00204	0.06808	0.00227	0.00224	0.00196	0.00167	0.00972	0.01250	0.01871	0.01209	0.01315	0.01508
Q9WV85	NDK3_MOUSE	0.00000	0.00000	0.00000	0.00000	0.00000	0.00000	0.00000	0.00341	0.00373	0.00264	0.00085	0.00173
Q9WV91	FPRP_MOUSE	0.00000	0.00000	0.00000	0.00000	0.00000	0.00000	0.00673	0.01651	0.02415	0.01649	0.02769	0.01331
Q9WV92	E41L3_MOUSE	0.00000	0.00000	0.00000	0.00000	0.00000	0.00000	0.00417	0.00509	0.00408	0.00345	0.00323	0.00351
Q9WVA3	BUB3_MOUSE	0.00000	0.00000	0.00000	0.00000	0.00000	0.00000	0.00206	0.01261	0.00781	0.00967	0.00646	0.00939
Q9WVA4	TAGL2_MOUSE	0.01019	0.00491	0.00690	0.00531	0.00873	0.01058	0.30631	0.37398	0.33790	0.36066	0.26813	0.42226
Q9WVB0	RBPMS_MOUSE	0.00093	0.00082	0.00089	0.00166	0.00062	0.00018	0.00877	0.00474	0.00858	0.00701	0.00909	0.00786

Q9WVC3	CAV2_MOUSE	0.00000	0.00000	0.00000	0.00000	0.00000	0.00000	0.02637	0.05537	0.06541	0.05646	0.06219	0.05916
Q9WVD5	ORNT1_MOUSE	0.00000	0.00000	0.00000	0.00000	0.00000	0.00000	0.00147	0.00123	0.00019	0.00020	0.00000	0.00112
Q9WVE8	PACN2_MOUSE	0.00880	0.00915	0.00558	0.00581	0.00591	0.00909	0.02406	0.03392	0.03704	0.03337	0.03423	0.03075
Q9WVH9	FBLN5_MOUSE	0.00133	0.00052	0.00075	0.00018	0.00083	0.00243	0.02319	0.06883	0.03651	0.02583	0.03986	0.05495
Q9WVJ2	PSD13_MOUSE	0.01449	0.01693	0.01375	0.01704	0.01543	0.01489	0.01638	0.01365	0.02112	0.01815	0.02211	0.01600
Q9WVJ3	CBPQ_MOUSE	0.00091	0.00025	0.00051	0.00081	0.00077	0.00081	0.00321	0.00090	0.00868	0.00696	0.00729	0.00513
Q9WVJ9	FBLN4_MOUSE	0.00000	0.00000	0.00000	0.00000	0.00000	0.00000	0.00000	0.00529	0.00000	0.00000	0.00000	0.00000
Q9WVK0	ATRAP_MOUSE	0.00000	0.00000	0.00000	0.00000	0.00000	0.00000	0.00000	0.00265	0.00000	0.00151	0.00000	0.00213
Q9WVK4	EHD1_MOUSE	0.02717	0.01772	0.02502	0.01963	0.02976	0.02510	0.15354	0.13723	0.15537	0.13814	0.15384	0.15284
Q9WVL0	MAAI_MOUSE	0.02074	0.01541	0.01377	0.01000	0.01111	0.01195	0.01948	0.01408	0.01588	0.01301	0.01279	0.01028
Q9WVL3	S12A7_MOUSE	0.01188	0.01175	0.01142	0.01114	0.00773	0.01273	0.00000	0.00000	0.00000	0.00000	0.00467	0.00000
Q9WVM8	AADAT_MOUSE	0.00000	0.00000	0.00000	0.00000	0.00000	0.00000	0.00000	0.00000	0.00000	0.00000	0.00000	0.00000
Q9WVQ5	MTNB_MOUSE	0.00471	0.00282	0.00295	0.00372	0.00386	0.00363	0.00486	0.00976	0.00847	0.00800	0.00332	0.01115
Q9WVR4	FXR2_MOUSE	0.00000	0.00000	0.00000	0.00000	0.00000	0.00000	0.00000	0.00374	0.00000	0.00124	0.00000	0.00127
Q9WVT6	CAH14_MOUSE	0.00000	0.00000	0.00000	0.00000	0.00000	0.00000	0.00546	0.00934	0.01326	0.00799	0.01626	0.01326
Q9Z0E6	GBP2_MOUSE	0.00000	0.00000	0.00000	0.00000	0.00000	0.00000	0.02263	0.01935	0.02913	0.02361	0.02439	0.02261
Q9Z0F7	SYUG_MOUSE	0.00000	0.00000	0.00000	0.00000	0.00000	0.00000	0.00271	0.00083	0.00455	0.00263	0.00134	0.00112
Q9Z0F8	ADA17_MOUSE	0.00000	0.00000	0.00000	0.00000	0.00000	0.00000	0.00000	0.00079	0.00253	0.00248	0.00410	0.00255
Q9Z0G9	CLD3_MOUSE	0.00000	0.00000	0.00000	0.00000	0.00000	0.00000	0.00000	0.00000	0.00275	0.00208	0.00157	0.00146
Q9Z0H4	CELF2_MOUSE	0.00000	0.00000	0.00000	0.00000	0.00000	0.00000	0.00658	0.00116	0.01104	0.00630	0.01128	0.01058
Q9Z0K8	VNN1_MOUSE	0.00000	0.00000	0.00000	0.00000	0.00000	0.00000	0.00000	0.00000	0.00000	0.00078	0.00000	0.00000
Q9Z0M5	LICH_MOUSE	0.00000	0.00000	0.00000	0.00000	0.00000	0.00000	0.00191	0.00479	0.00188	0.00103	0.00103	0.00459
Q9Z0M6	AGRE5_MOUSE	0.00000	0.00000	0.00000	0.00000	0.00000	0.00000	0.00862	0.01032	0.01148	0.01004	0.01029	0.01266
Q9Z0N1	IF2G_MOUSE	0.00010	0.00000	0.00052	0.00030	0.00007	0.00000	0.00857	0.00765	0.01073	0.00872	0.01065	0.00857
Q9Z0N2	IF2H_MOUSE	0.00008	0.00000	0.00003	0.00000	0.00037	0.00025	0.00068	0.00169	0.00551	0.00176	0.00000	0.00343
Q9Z0P4	PALM_MOUSE	0.00000	0.00000	0.00000	0.00000	0.00000	0.00000	0.00374	0.01427	0.01756	0.01189	0.01051	0.01369
Q9Z0P5	TWF2_MOUSE	0.00599	0.00802	0.00543	0.00825	0.06488	0.00556	0.01760	0.01336	0.02374	0.01678	0.02115	0.01789
Q9Z0S1	BPNT1_MOUSE	0.00361	0.00197	0.00318	0.00180	0.00249	0.00085	0.01336	0.01749	0.02448	0.01894	0.02042	0.01959
Q9Z0S2	LHX2_MOUSE	0.00000	0.00000	0.00000	0.00000	0.00000	0.00000	0.10399	0.07224	0.07796	0.14859	0.21804	0.03390
Q9Z0T9	ITB6_MOUSE	0.00000	0.00000	0.00000	0.00000	0.00000	0.00000	0.02909	0.07831	0.02419	0.03144	0.03019	0.05285
Q9Z0U1	ZO2_MOUSE	0.00000	0.00000	0.00000	0.00000	0.00000	0.00000	0.01076	0.02878	0.02932	0.03000	0.03364	0.02395
Q9Z0W3	NU160_MOUSE	0.00000	0.00000	0.00000	0.00000	0.00000	0.00000	0.00031	0.00000	0.00116	0.00063	0.00104	0.00066
Q9Z0X1	AIFM1_MOUSE	0.06131	0.05085	0.09590	0.05768	0.10223	0.04744	0.02145	0.01887	0.01281	0.01498	0.01503	0.01196
Q9Z0X4	PDE3A_MOUSE	0.00000	0.00000	0.00000	0.00000	0.00000	0.00000	0.00481	0.01252	0.00878	0.01101	0.00882	0.01179

Q9Z126	PLF4_MOUSE	0.00000	0.00000	0.00000	0.00000	0.00000	0.00000	0.00000	0.00113	0.00603	0.00445	0.00164	0.00223
Q9Z130	HNRDL_MOUSE	0.00000	0.00000	0.00000	0.00000	0.00000	0.00000	0.00875	0.01281	0.01968	0.01648	0.01558	0.01928
Q9Z1B3	PLCB1_MOUSE	0.00000	0.00000	0.00000	0.00000	0.00000	0.00000	0.00069	0.00151	0.00272	0.00077	0.00236	0.00148
Q9Z1D1	EIF3G_MOUSE	0.00000	0.00000	0.00000	0.00000	0.00000	0.00000	0.01121	0.00982	0.01270	0.00942	0.01049	0.01029
Q9Z1E4	GYS1_MOUSE	0.01957	0.01607	0.03693	0.02851	0.02116	0.02472	0.04031	0.01466	0.01492	0.01578	0.00554	0.00636
Q9Z1F9	SAE2_MOUSE	0.00000	0.00000	0.00000	0.00000	0.00000	0.00000	0.02365	0.02971	0.03439	0.03453	0.04768	0.03984
Q9Z1G3	VATC1_MOUSE	0.00000	0.00000	0.00000	0.00000	0.00000	0.00000	0.00000	0.00000	0.00067	0.00048	0.00000	0.00000
Q9Z1G4	VPP1_MOUSE	0.00000	0.00000	0.00000	0.00000	0.00000	0.00000	0.00164	0.00480	0.00093	0.00269	0.00000	0.00098
Q9Z1J3	NFS1_MOUSE	0.00747	0.00650	0.00597	0.00654	0.00742	0.00459	0.00072	0.00391	0.00220	0.00180	0.00192	0.00116
Q9Z1K5	ARI1_MOUSE	0.00000	0.00000	0.00000	0.00000	0.00000	0.00000	0.00000	0.00367	0.00427	0.00313	0.00142	0.00149
Q9Z1M8	RED_MOUSE	0.00000	0.00000	0.00000	0.00000	0.00000	0.00000	0.00000	0.00384	0.00308	0.00255	0.00415	0.00345
Q9Z1N5	DX39B_MOUSE	0.00209	0.00116	0.00241	0.00158	0.00295	0.00239	0.03255	0.02831	0.04451	0.03628	0.03922	0.03810
Q9Z1P6	NDUA7_MOUSE	0.00000	0.00000	0.00000	0.00000	0.00000	0.00000	0.00000	0.00000	0.00000	0.00000	0.00000	0.00000
Q9Z1P7	KANK3_MOUSE	0.00000	0.00000	0.00000	0.00000	0.00000	0.00000	0.00230	0.00873	0.00911	0.00996	0.00464	0.01036
Q9Z1Q2	ABHGA_MOUSE	0.00000	0.00000	0.00000	0.00000	0.00000	0.00000	0.00000	0.00000	0.00081	0.00000	0.00137	0.00033
Q9Z1Q5	CLIC1_MOUSE	0.03921	0.02243	0.03259	0.01120	0.03569	0.01399	0.10642	0.09510	0.11502	0.11775	0.10376	0.13292
Q9Z1Q9	SYVC_MOUSE	0.00221	0.00351	0.00260	0.00398	0.00276	0.00083	0.03480	0.03085	0.04818	0.03672	0.03646	0.03746
Q9Z1R2	BAG6_MOUSE	0.00000	0.00000	0.00000	0.00000	0.00000	0.00000	0.00909	0.00416	0.00755	0.00338	0.00375	0.00486
Q9Z1T1	AP3B1_MOUSE	0.00000	0.00000	0.00000	0.00000	0.00000	0.00000	0.01046	0.00978	0.01126	0.01019	0.00848	0.01304
Q9Z1X4	ILF3_MOUSE	0.00000	0.00000	0.00000	0.00000	0.00000	0.00000	0.01207	0.00920	0.01185	0.01249	0.00683	0.01472
Q9Z1Y4	TRIP6_MOUSE	0.00000	0.00000	0.00000	0.00000	0.00000	0.00000	0.00000	0.00079	0.00232	0.00172	0.00000	0.00094
Q9Z1Z0	USO1_MOUSE	0.00643	0.00987	0.00636	0.00363	0.01099	0.00543	0.00876	0.00993	0.01401	0.01106	0.01137	0.01037
Q9Z1Z2	STRAP_MOUSE	0.00694	0.00917	0.00416	0.00527	0.01001	0.00603	0.01823	0.01355	0.02160	0.02006	0.02115	0.01855
Q9Z204	HNRPC_MOUSE	0.00000	0.00000	0.00000	0.00000	0.00000	0.00000	0.03404	0.03030	0.02450	0.03071	0.02755	0.03568
Q9Z239	PLM_MOUSE	0.00000	0.00000	0.00000	0.00000	0.00000	0.00000	0.00000	0.00660	0.00993	0.00891	0.00465	0.00380
Q9Z247	FKBP9_MOUSE	0.00000	0.00000	0.00000	0.00000	0.00000	0.00000	0.00000	0.00140	0.00512	0.00517	0.00266	0.00710
Q9Z261	CLD7_MOUSE	0.00000	0.00000	0.00000	0.00000	0.00000	0.00000	0.00000	0.00000	0.00305	0.00100	0.00032	0.00551
Q9Z266	SNAPN_MOUSE	0.00000	0.00000	0.00000	0.00000	0.00000	0.00000	0.00075	0.00201	0.00210	0.00323	0.00208	0.00448
Q9Z277	BAZ1B_MOUSE	0.00000	0.00000	0.00000	0.00000	0.00000	0.00000	0.00298	0.00230	0.00317	0.00308	0.00437	0.00408
Q9Z280	PLD1_MOUSE	0.00000	0.00000	0.00000	0.00000	0.00000	0.00000	0.00000	0.00000	0.00067	0.00000	0.00080	0.00000
Q9Z2A5	ATE1_MOUSE	0.00000	0.00000	0.00000	0.00000	0.00000	0.00000	0.00271	0.00235	0.00513	0.00295	0.00603	0.00383
Q9Z2C5	MTM1_MOUSE	0.00000	0.00000	0.00000	0.00000	0.00000	0.00000	0.00064	0.00206	0.00145	0.00000	0.00000	0.00000
Q9Z2C6	UPK1B_MOUSE	0.00000	0.00000	0.00000	0.00000	0.00000	0.00000	0.00035	0.00000	0.00100	0.00033	0.00084	0.00049
Q9Z2D1	MTMR2_MOUSE	0.00000	0.00000	0.00000	0.00000	0.00000	0.00000	0.00000	0.00108	0.00000	0.00030	0.00111	0.00040

Q9Z2D6	MECP2_MOUSE	0.00147	0.00675	0.00198	0.00250	0.00274	0.00274	0.03366	0.04773	0.05484	0.04933	0.04635	0.04286
Q9Z2G6	SE1L1_MOUSE	0.00000	0.00000	0.00000	0.00000	0.00000	0.00000	0.00000	0.00061	0.00089	0.00076	0.00000	0.00000
Q9Z2H5	E41L1_MOUSE	0.00000	0.00000	0.00000	0.00000	0.00000	0.00000	0.00000	0.00171	0.00000	0.00119	0.01679	0.00904
Q9Z2H7	GIPC2_MOUSE	0.00000	0.00000	0.00000	0.00000	0.00000	0.00000	0.01399	0.00703	0.00381	0.00830	0.00632	0.00448
Q9Z2I0	LETM1_MOUSE	0.05513	0.04126	0.05912	0.03313	0.03847	0.04156	0.01656	0.01513	0.00991	0.01002	0.00936	0.01193
Q9Z2I8	SUCB2_MOUSE	0.05851	0.03298	0.05682	0.03642	0.05160	0.04502	0.02644	0.02339	0.02217	0.02135	0.02292	0.02543
Q9Z2I9	SUCB1_MOUSE	0.43121	0.29574	0.30563	0.29150	0.39264	0.36628	0.03511	0.03354	0.02806	0.02341	0.02635	0.02713
Q9Z2J0	S23A1_MOUSE	0.00000	0.00000	0.00000	0.00000	0.00000	0.00000	0.00000	0.00000	0.00000	0.00000	0.00000	0.00000
Q9Z2L7	CRLF3_MOUSE	0.00000	0.00000	0.00000	0.00000	0.00000	0.00000	0.00000	0.00235	0.00330	0.00300	0.00321	0.00166
Q9Z2M7	PMM2_MOUSE	0.00000	0.00000	0.00000	0.00000	0.00000	0.00000	0.00402	0.01139	0.02655	0.02397	0.01334	0.03388
Q9Z2N8	ACL6A_MOUSE	0.00000	0.00000	0.00000	0.00000	0.00000	0.00000	0.01317	0.01057	0.01461	0.01512	0.01924	0.01796
Q9Z2P8	VAMP5_MOUSE	0.00000	0.00000	0.00000	0.00000	0.00000	0.00000	0.00000	0.00513	0.00113	0.00082	0.00000	0.00460
Q9Z2U0	PSA7_MOUSE	0.01927	0.01984	0.02154	0.02001	0.01842	0.02665	0.03671	0.03335	0.02269	0.03406	0.02089	0.03675
Q9Z2U1	PSA5_MOUSE	0.00000	0.00000	0.00000	0.00000	0.00000	0.00000	0.04225	0.03916	0.05379	0.04698	0.06485	0.04661
Q9Z2V4	PCKGC_MOUSE	0.00000	0.00000	0.00000	0.00000	0.00000	0.00000	0.00003	0.00017	0.00023	0.00020	0.00012	0.00019
Q9Z2V5	HDAC6_MOUSE	0.00000	0.00000	0.00000	0.00000	0.00000	0.00000	0.00000	0.00000	0.00019	0.00020	0.00018	0.00036
Q9Z2W0	DNPEP_MOUSE	0.00879	0.00884	0.01250	0.01154	0.00796	0.00845	0.00793	0.01254	0.01947	0.01262	0.01358	0.01627
Q9Z2W1	STK25_MOUSE	0.00000	0.00000	0.00000	0.00000	0.00000	0.00000	0.00239	0.00427	0.00429	0.00202	0.00424	0.00391
Q9Z2X1	HNRPF_MOUSE	0.00481	0.00326	0.00484	0.00311	0.00403	0.00263	0.05755	0.04388	0.07198	0.06171	0.08761	0.07926
Q9Z2Y8	PLPHP_MOUSE	0.01009	0.00673	0.00834	0.00804	0.00957	0.00703	0.00920	0.01369	0.01407	0.01165	0.01418	0.01503
Q9Z2Z6	MCAT_MOUSE	0.00200	0.00627	0.00673	0.00594	0.00678	0.00737	0.01283	0.01912	0.00866	0.00760	0.00134	0.01268
Q9Z306	S22A4_MOUSE	0.00000	0.00000	0.00000	0.00000	0.00000	0.00000	0.00000	0.00000	0.00000	0.00000	0.00000	0.00000
Q9Z315	SNUT1_MOUSE	0.00000	0.00000	0.00000	0.00000	0.00000	0.00000	0.00254	0.00420	0.00535	0.00291	0.00520	0.00373
O55111	DSG2_MOUSE	0.03359	0.03626	0.02043	0.03848	0.03767	0.02539	0.00000	0.00000	0.00000	0.00000	0.00000	0.00000
Q9ESD7	DYSF_MOUSE	0.01874	0.01953	0.01190	0.02300	0.01680	0.01611	0.00000	0.00000	0.00000	0.00000	0.00000	0.00000
Q62407	SPEG_MOUSE	0.00795	0.00867	0.00419	0.00938	0.00712	0.00852	0.00000	0.00000	0.00000	0.00000	0.00000	0.00000
Q0II04	NEBL_MOUSE	0.03890	0.02381	0.05232	0.05075	0.03466	0.02401	0.00000	0.00000	0.00000	0.00000	0.00000	0.00000
Q8C8R3	ANK2_MOUSE	0.00906	0.00950	0.00941	0.01084	0.00871	0.00484	0.00000	0.00000	0.00000	0.00000	0.00000	0.00000
Q80XB4	NRAP_MOUSE	0.02369	0.04040	0.03125	0.03241	0.03649	0.03535	0.00000	0.00000	0.00000	0.00000	0.00000	0.00000
Q6ZQ73	CAND2_MOUSE	0.01471	0.01620	0.01643	0.01521	0.01442	0.01263	0.00000	0.00000	0.00000	0.00000	0.00000	0.00000
Q9CWF2	TBB2B_MOUSE	0.00585	0.00655	0.00309	0.00901	0.00682	0.00532	0.00000	0.00000	0.00000	0.00000	0.00000	0.00000
O70373	XIRP1_MOUSE	0.01213	0.01118	0.01010	0.00805	0.00639	0.00769	0.00000	0.00000	0.00000	0.00000	0.00000	0.00000
P15116	CADH2_MOUSE	0.04340	0.03676	0.03303	0.02571	0.03028	0.03715	0.00000	0.00000	0.00000	0.00000	0.00000	0.00000
Q99MR9	PPR3A_MOUSE	0.00876	0.00607	0.00661	0.01135	0.00640	0.00894	0.00000	0.00000	0.00000	0.00000	0.00000	0.00000

Q3UIZ8	MYLK3_MOUSE	0.00923	0.01359	0.00779	0.01028	0.00702	0.00847	0.00000	0.00000	0.00000	0.00000	0.00000	0.00000
P05214	TBA3_MOUSE	0.00075	0.00153	0.00108	0.00240	0.00088	0.00143	0.00000	0.00000	0.00000	0.00000	0.00000	0.00000
Q61738	ITA7_MOUSE	0.01437	0.01967	0.00955	0.00818	0.01541	0.01248	0.00000	0.00000	0.00000	0.00000	0.00000	0.00000
Q65CL1	CTNA3_MOUSE	0.01184	0.01481	0.00818	0.00850	0.01127	0.00661	0.00000	0.00000	0.00000	0.00000	0.00000	0.00000
E9Q4Z2	ACACB_MOUSE	0.00639	0.01150	0.00781	0.01398	0.00684	0.01170	0.00000	0.00000	0.00000	0.00000	0.00000	0.00000
P07758	A1AT1_MOUSE	0.01097	0.00886	0.00764	0.01624	0.01718	0.00392	0.00000	0.00000	0.00000	0.00000	0.00000	0.00000
Q91ZU6	DYST_MOUSE	0.00590	0.00560	0.00464	0.00535	0.00644	0.00580	0.00000	0.00000	0.00000	0.00000	0.00000	0.00000
P62715	PP2AB_MOUSE	0.00446	0.00111	0.00422	0.00278	0.00215	0.00274	0.00000	0.00000	0.00000	0.00000	0.00000	0.00000
F2Z472	SVS3A_MOUSE	0.01253	0.00702	0.01755	0.34739	0.00604	0.01356	0.00000	0.00000	0.00000	0.00000	0.00000	0.00000
Q8K009	AL1L2_MOUSE	0.00513	0.01358	0.00255	0.00540	0.00314	0.00293	0.00000	0.00000	0.00000	0.00000	0.00000	0.00000
Q811U4	MFN1_MOUSE	0.02196	0.02036	0.02643	0.03259	0.02283	0.01711	0.00000	0.00000	0.00000	0.00000	0.00000	0.00000
Q9QZF2	GPC1_MOUSE	0.00587	0.00500	0.00717	0.00689	0.00531	0.00374	0.00000	0.00000	0.00000	0.00000	0.00000	0.00000
Q8BWB1	SYP2L_MOUSE	0.00476	0.00593	0.00480	0.00330	0.00737	0.00374	0.00000	0.00000	0.00000	0.00000	0.00000	0.00000
Q8BRK8	AAPK2_MOUSE	0.00806	0.00389	0.00653	0.00580	0.00488	0.00308	0.00000	0.00000	0.00000	0.00000	0.00000	0.00000
Q8CGY6	UN45B_MOUSE	0.00344	0.00623	0.00579	0.00482	0.00523	0.00518	0.00000	0.00000	0.00000	0.00000	0.00000	0.00000
P30933	SVS5_MOUSE	0.00057	0.00151	0.00452	0.13018	0.00086	0.00103	0.00000	0.00000	0.00000	0.00000	0.00000	0.00000
P62962	PROF1_MOUSE	0.02842	0.02172	0.01375	0.02371	0.02776	0.06677	0.00000	0.00000	0.00000	0.00000	0.00000	0.00000
O89020	AFAM_MOUSE	0.00098	0.00078	0.00111	0.00249	0.00195	0.00238	0.00000	0.00000	0.00000	0.00000	0.00000	0.00000
Q9JI39	ABCBA_MOUSE	0.00235	0.00464	0.00831	0.00721	0.01406	0.00830	0.00000	0.00000	0.00000	0.00000	0.00000	0.00000
O70433	FHL2_MOUSE	0.00737	0.00458	0.00910	0.01200	0.01252	0.00588	0.00000	0.00000	0.00000	0.00000	0.00000	0.00000
Q76LL6	FHOD3_MOUSE	0.00041	0.00086	0.00095	0.00088	0.00118	0.00075	0.00000	0.00000	0.00000	0.00000	0.00000	0.00000
Q8R1S0	COQ6_MOUSE	0.01269	0.01014	0.02072	0.01325	0.01597	0.01508	0.00000	0.00000	0.00000	0.00000	0.00000	0.00000
P55292	DSC2_MOUSE	0.01872	0.02078	0.01494	0.01617	0.01567	0.01995	0.00000	0.00000	0.00000	0.00000	0.00000	0.00000
Q8BGK2	ARHL1_MOUSE	0.04974	0.04614	0.03785	0.03329	0.03350	0.04023	0.00000	0.00000	0.00000	0.00000	0.00000	0.00000
Q3UHZ5	LMOD2_MOUSE	0.00929	0.00536	0.00780	0.00868	0.00813	0.00371	0.00000	0.00000	0.00000	0.00000	0.00000	0.00000
Q8VD26	TM143_MOUSE	0.00759	0.00628	0.01016	0.00874	0.00711	0.00901	0.00000	0.00000	0.00000	0.00000	0.00000	0.00000
Q8C7H1	MMAA_MOUSE	0.00415	0.00264	0.00436	0.00387	0.00374	0.00263	0.00000	0.00000	0.00000	0.00000	0.00000	0.00000
Q8BVZ1	PLIN5_MOUSE	0.00413	0.00213	0.00199	0.00197	0.00259	0.00279	0.00000	0.00000	0.00000	0.00000	0.00000	0.00000
Q4U4S6	XIRP2_MOUSE	0.00086	0.00106	0.00107	0.00730	0.00087	0.01009	0.00000	0.00000	0.00000	0.00000	0.00000	0.00000
Q62059	CSPG2_MOUSE	0.01772	0.04531	0.02625	0.03222	0.03106	0.04834	0.00000	0.00000	0.00000	0.00000	0.00000	0.00000
P19639	GSTM3_MOUSE	0.00060	0.00107	0.00139	0.00038	0.00020	0.00011	0.00000	0.00000	0.00000	0.00000	0.00000	0.00000
Q3UFY7	5NT3B_MOUSE	0.00525	0.00191	0.00694	0.00207	0.00243	0.00221	0.00000	0.00000	0.00000	0.00000	0.00000	0.00000
Q9ER35	FN3K_MOUSE	0.01215	0.00199	0.00248	0.00221	0.00184	0.00135	0.00000	0.00000	0.00000	0.00000	0.00000	0.00000
Q9D0L4	ADCK1_MOUSE	0.01753	0.01323	0.00795	0.00650	0.00725	0.00698	0.00000	0.00000	0.00000	0.00000	0.00000	0.00000

P0DP26	CALM1_MOUSE	0.04293	0.06117	0.03337	0.04652	0.03732	0.06627	0.00000	0.00000	0.00000	0.00000	0.00000	0.00000
Q8CHS7	DRS7C_MOUSE	0.00511	0.00604	0.00540	0.00617	0.00576	0.00773	0.00000	0.00000	0.00000	0.00000	0.00000	0.00000
P98156	VLDLR_MOUSE	0.00169	0.00239	0.00151	0.00145	0.00221	0.00248	0.00000	0.00000	0.00000	0.00000	0.00000	0.00000
Q6PAM0	AAKB2_MOUSE	0.00172	0.00245	0.00211	0.00355	0.00103	0.00264	0.00000	0.00000	0.00000	0.00000	0.00000	0.00000
P35385	HSPB7_MOUSE	0.00511	0.00510	0.00328	0.01084	0.00358	0.00178	0.00000	0.00000	0.00000	0.00000	0.00000	0.00000
P62880	GBB2_MOUSE	0.02197	0.01640	0.01460	0.01067	0.02649	0.02444	0.00000	0.00000	0.00000	0.00000	0.00000	0.00000
Q80X76	SPA3F_MOUSE	0.00033	0.00152	0.00033	0.00032	0.00027	0.00045	0.00000	0.00000	0.00000	0.00000	0.00000	0.00000
Q99JR6	NMNA3_MOUSE	0.00298	0.00306	0.00488	0.00381	0.00296	0.00208	0.00000	0.00000	0.00000	0.00000	0.00000	0.00000
Q91WC3	ACSL6_MOUSE	0.00712	0.00284	0.00256	0.00300	0.00362	0.00212	0.00000	0.00000	0.00000	0.00000	0.00000	0.00000
Q99MS7	EH1L1_MOUSE	0.00252	0.00313	0.00233	0.00225	0.00243	0.00254	0.00000	0.00000	0.00000	0.00000	0.00000	0.00000
O70548	TELT_MOUSE	0.01481	0.01504	0.01489	0.01566	0.02080	0.00749	0.00000	0.00000	0.00000	0.00000	0.00000	0.00000
P07934	PHKG1_MOUSE	0.00194	0.00386	0.00087	0.00304	0.00150	0.00143	0.00000	0.00000	0.00000	0.00000	0.00000	0.00000
P16627	HS71L_MOUSE	0.00034	0.00053	0.00033	0.00036	0.00034	0.00037	0.00000	0.00000	0.00000	0.00000	0.00000	0.00000
Q9CWX2	CIA30_MOUSE	0.00261	0.00262	0.00394	0.00416	0.00240	0.00210	0.00000	0.00000	0.00000	0.00000	0.00000	0.00000
Q9CR24	NUDT8_MOUSE	0.00650	0.00787	0.00533	0.00623	0.00677	0.00323	0.00000	0.00000	0.00000	0.00000	0.00000	0.00000
P56501	UCP3_MOUSE	0.01200	0.02201	0.02396	0.02800	0.01470	0.00653	0.00000	0.00000	0.00000	0.00000	0.00000	0.00000
Q6PD31	TRAK1_MOUSE	0.00269	0.00157	0.00102	0.00158	0.00105	0.00166	0.00000	0.00000	0.00000	0.00000	0.00000	0.00000
Q8CEK3	SPIKL_MOUSE	0.00008	0.00022	0.00035	0.03220	0.00007	0.00000	0.00000	0.00000	0.00000	0.00000	0.00000	0.00000
P56391	CX6B1_MOUSE	0.38151	0.18198	0.29293	0.15187	0.26038	0.16611	0.00000	0.00000	0.00000	0.00000	0.00000	0.00000
A6H611	MIPEP_MOUSE	0.00986	0.00711	0.00540	0.00508	0.00150	0.00165	0.00000	0.00000	0.00000	0.00000	0.00000	0.00000
Q8R3Q6	CCD58_MOUSE	0.00809	0.02243	0.04191	0.12814	0.19365	0.01024	0.00000	0.00000	0.00000	0.00000	0.00000	0.00000
Q3TVI8	PBIP1_MOUSE	0.00000	0.00027	0.00015	0.00006	0.00098	0.00013	0.00000	0.00000	0.00000	0.00000	0.00000	0.00000
Q8R2G4	NAR3_MOUSE	0.01524	0.04061	0.01140	0.02159	0.02717	0.02694	0.00000	0.00000	0.00000	0.00000	0.00000	0.00000
Q3U0B3	DHR11_MOUSE	0.00243	0.01855	0.00577	0.02881	0.00502	0.03058	0.00000	0.00000	0.00000	0.00000	0.00000	0.00000
A2A884	ZEP3_MOUSE	0.00157	0.00265	0.00148	0.00187	0.00174	0.00869	0.00000	0.00000	0.00000	0.00000	0.00000	0.00000
Q9CPS6	HINT3_MOUSE	0.00029	0.00000	0.00031	0.00032	0.00054	0.00058	0.00000	0.00000	0.00000	0.00000	0.00000	0.00000
P82350	SGCA_MOUSE	0.01169	0.01608	0.00911	0.01028	0.00443	0.00873	0.00000	0.00000	0.00000	0.00000	0.00000	0.00000
Q8BFZ1	TECRL_MOUSE	0.03434	0.00659	0.00676	0.00674	0.00730	0.00697	0.00000	0.00000	0.00000	0.00000	0.00000	0.00000
Q9WUZ7	SH3BG_MOUSE	0.00030	0.00000	0.00009	0.00036	0.00028	0.00035	0.00000	0.00000	0.00000	0.00000	0.00000	0.00000
Q61207	SAP_MOUSE	0.02945	0.03181	0.03458	0.03723	0.04069	0.03345	0.00000	0.00000	0.00000	0.00000	0.00000	0.00000
P70170	ABCC9_MOUSE	0.00185	0.00460	0.00374	0.00257	0.00104	0.00137	0.00000	0.00000	0.00000	0.00000	0.00000	0.00000
P42230	STA5A_MOUSE	0.00715	0.00113	0.00122	0.00948	0.00414	0.00313	0.00000	0.00000	0.00000	0.00000	0.00000	0.00000
Q99MI1	RB6I2_MOUSE	0.00150	0.00164	0.00133	0.00117	0.00187	0.00179	0.00000	0.00000	0.00000	0.00000	0.00000	0.00000
Q91VW5	GOGA4_MOUSE	0.00016	0.00317	0.00031	0.00050	0.00046	0.00021	0.00000	0.00000	0.00000	0.00000	0.00000	0.00000

Q91YM4	FAKD4_MOUSE	0.00090	0.00136	0.00170	0.00284	0.00144	0.00160	0.00000	0.00000	0.00000	0.00000	0.00000	0.00000
Q3UTJ2	SRBS2_MOUSE	0.00494	0.00489	0.00618	0.00810	0.00538	0.00827	0.00000	0.00000	0.00000	0.00000	0.00000	0.00000
Q6IR34	GPSM1_MOUSE	0.00149	0.00156	0.00152	0.00098	0.00046	0.00105	0.00000	0.00000	0.00000	0.00000	0.00000	0.00000
Q9CQT1	MTNA_MOUSE	0.00000	0.00029	0.00059	0.00064	0.00050	0.00031	0.00000	0.00000	0.00000	0.00000	0.00000	0.00000
Q6NVE9	PPTC7_MOUSE	0.00366	0.00130	0.00256	0.00320	0.00217	0.00331	0.00000	0.00000	0.00000	0.00000	0.00000	0.00000
Q9DCZ1	GMPR1_MOUSE	0.00315	0.00009	0.00275	0.00332	0.00448	0.00114	0.00000	0.00000	0.00000	0.00000	0.00000	0.00000
Q9R013	CATF_MOUSE	0.00088	0.00063	0.00047	0.00028	0.00095	0.00114	0.00000	0.00000	0.00000	0.00000	0.00000	0.00000
Q9CXU9	EIF1B_MOUSE	0.00376	0.00167	0.00271	0.00082	0.00230	0.00159	0.00000	0.00000	0.00000	0.00000	0.00000	0.00000
E9Q9K5	TRDN_MOUSE	0.00373	0.00550	0.00302	0.00495	0.00536	0.00262	0.00000	0.00000	0.00000	0.00000	0.00000	0.00000
Q9CYT6	CAP2_MOUSE	0.00742	0.00548	0.00298	0.00538	0.00459	0.00665	0.00000	0.00000	0.00000	0.00000	0.00000	0.00000
Q9D8H7	OMA1_MOUSE	0.00034	0.00128	0.00268	0.00113	0.00083	0.00087	0.00000	0.00000	0.00000	0.00000	0.00000	0.00000
P21126	UBL4A_MOUSE	0.00430	0.01037	0.00457	0.00754	0.01071	0.00307	0.00000	0.00000	0.00000	0.00000	0.00000	0.00000
A2AGL3	RYR3_MOUSE	0.00108	0.00140	0.00106	0.00121	0.00164	0.00156	0.00000	0.00000	0.00000	0.00000	0.00000	0.00000
Q9JIK9	RT34_MOUSE	0.00035	0.00149	0.00135	0.00125	0.00127	0.00106	0.00000	0.00000	0.00000	0.00000	0.00000	0.00000
Q6ZQB6	VIP2_MOUSE	0.00188	0.00238	0.00218	0.00199	0.00203	0.00241	0.00000	0.00000	0.00000	0.00000	0.00000	0.00000
Q8BTX9	HSDL1_MOUSE	0.00056	0.00000	0.00046	0.00112	0.00010	0.00021	0.00000	0.00000	0.00000	0.00000	0.00000	0.00000
Q9WV96	T10B_MOUSE	0.00052	0.00038	0.00042	0.00015	0.00057	0.00010	0.00000	0.00000	0.00000	0.00000	0.00000	0.00000
Q5ND29	RILP_MOUSE	0.02159	0.00014	0.00032	0.00255	0.01613	0.00823	0.00000	0.00000	0.00000	0.00000	0.00000	0.00000
Q9D7J4	COX20_MOUSE	0.01549	0.01144	0.01451	0.00711	0.01096	0.00741	0.00000	0.00000	0.00000	0.00000	0.00000	0.00000
Q8BKE9	IFT74_MOUSE	0.00128	0.00214	0.00131	0.00102	0.00232	0.00186	0.00000	0.00000	0.00000	0.00000	0.00000	0.00000
Q5H8C4	VP13A_MOUSE	0.16984	0.09104	0.09023	0.05183	0.07122	0.07673	0.00000	0.00000	0.00000	0.00000	0.00000	0.00000
Q6QD59	SEC20_MOUSE	0.00173	0.00169	0.00134	0.00168	0.00133	0.00178	0.00000	0.00000	0.00000	0.00000	0.00000	0.00000
Q8BH55	THNS1_MOUSE	0.00367	0.00169	0.00175	0.00335	0.00084	0.00070	0.00000	0.00000	0.00000	0.00000	0.00000	0.00000
Q8BYM8	SYCM_MOUSE	0.00369	0.00259	0.00327	0.00206	0.00322	0.00333	0.00000	0.00000	0.00000	0.00000	0.00000	0.00000
Q8C0M9	ASGL1_MOUSE	0.00433	0.00322	0.00179	0.00217	0.00341	0.00372	0.00000	0.00000	0.00000	0.00000	0.00000	0.00000
P48725	PCNT_MOUSE	0.01195	0.00701	0.01303	0.00758	0.00783	0.01626	0.00000	0.00000	0.00000	0.00000	0.00000	0.00000
Q8C0L9	GPCP1_MOUSE	0.00286	0.00150	0.00411	0.00129	0.00212	0.00146	0.00000	0.00000	0.00000	0.00000	0.00000	0.00000
A2ARZ3	FSIP2_MOUSE	0.00990	0.00954	0.00854	0.01229	0.00901	0.00583	0.00000	0.00000	0.00000	0.00000	0.00000	0.00000
Q8R104	SIR3_MOUSE	0.00387	0.00280	0.00358	0.00240	0.00360	0.00360	0.00000	0.00000	0.00000	0.00000	0.00000	0.00000
Q920M5	CORO6_MOUSE	0.00043	0.00166	0.00163	0.00080	0.00062	0.00213	0.00000	0.00000	0.00000	0.00000	0.00000	0.00000
Q61400	CEAMA_MOUSE	0.00301	0.00083	0.00316	0.00357	0.00095	0.00289	0.00000	0.00000	0.00000	0.00000	0.00000	0.00000
P49945	FRIL2_MOUSE	0.00305	0.00392	0.00616	0.00448	0.00369	0.00232	0.00000	0.00000	0.00000	0.00000	0.00000	0.00000
Q5SSK3	TEFM_MOUSE	0.00928	0.00375	0.00356	0.00254	0.00435	0.00352	0.00000	0.00000	0.00000	0.00000	0.00000	0.00000
Q9D7H3	RTCA_MOUSE	0.00125	0.00248	0.00147	0.00206	0.00151	0.00126	0.00000	0.00000	0.00000	0.00000	0.00000	0.00000

P62983	RS27A_MOUSE	0.21281	0.20518	0.17202	0.16028	0.13094	0.14994	0.00000	0.00000	0.00000	0.00000	0.00000	0.00000
Q8BZ52	FSD2_MOUSE	0.00064	0.00027	0.00052	0.00049	0.00081	0.00011	0.00000	0.00000	0.00000	0.00000	0.00000	0.00000
Q3ULF4	SPG7_MOUSE	0.00190	0.00151	0.00084	0.00142	0.00100	0.00075	0.00000	0.00000	0.00000	0.00000	0.00000	0.00000
O35660	GSTM6_MOUSE	0.00254	0.00526	0.00365	0.00324	0.00337	0.00329	0.00000	0.00000	0.00000	0.00000	0.00000	0.00000
Q69ZN6	GNPTA_MOUSE	0.00510	0.00821	0.00432	0.00353	0.00303	0.00594	0.00000	0.00000	0.00000	0.00000	0.00000	0.00000
Q8BJZ4	RT35_MOUSE	0.00144	0.00381	0.00156	0.00190	0.00163	0.00210	0.00000	0.00000	0.00000	0.00000	0.00000	0.00000
Q8CHC4	SYNJ1_MOUSE	0.00690	0.00732	0.00637	0.00669	0.00632	0.00525	0.00000	0.00000	0.00000	0.00000	0.00000	0.00000
Q9D273	MMAB_MOUSE	0.01204	0.00624	0.00584	0.00272	0.00856	0.00671	0.00000	0.00000	0.00000	0.00000	0.00000	0.00000
O70571	PDK4_MOUSE	0.00094	0.00101	0.00086	0.00054	0.00164	0.00000	0.00000	0.00000	0.00000	0.00000	0.00000	0.00000
Q9WUF3	C8AP2_MOUSE	0.00804	0.00804	0.00722	0.00519	0.00584	0.00753	0.00000	0.00000	0.00000	0.00000	0.00000	0.00000
Q8BKY8	MTEF2_MOUSE	0.01366	0.00910	0.00944	0.00893	0.00880	0.00787	0.00000	0.00000	0.00000	0.00000	0.00000	0.00000
Q6P549	SHIP2_MOUSE	0.00302	0.00356	0.00168	0.00153	0.00264	0.00234	0.00000	0.00000	0.00000	0.00000	0.00000	0.00000
Q99N96	RM01_MOUSE	0.00457	0.00294	0.00240	0.00326	0.00262	0.00282	0.00000	0.00000	0.00000	0.00000	0.00000	0.00000
P14427	HA14_MOUSE	0.00228	0.00185	0.00489	0.00098	0.00136	0.00552	0.00000	0.00000	0.00000	0.00000	0.00000	0.00000
Q3UHB1	NT5D3_MOUSE	0.00075	0.00058	0.00105	0.00050	0.00059	0.00092	0.00000	0.00000	0.00000	0.00000	0.00000	0.00000
Q9ERP3	TRI54_MOUSE	0.00500	0.00484	0.00372	0.00167	0.00555	0.00350	0.00000	0.00000	0.00000	0.00000	0.00000	0.00000
Q8VBT1	TXLNB_MOUSE	0.00213	0.00477	0.00164	0.00104	0.00181	0.00248	0.00000	0.00000	0.00000	0.00000	0.00000	0.00000
